# Understanding dynamic voltammetry in a dissolving microdroplet†

**DOI:** 10.1039/d4an00299g

**Published:** 2024-04-30

**Authors:** Ashutosh Rana, Christophe Renault, Jeffrey E. Dick

**Affiliations:** a Department of Chemistry, Purdue University West Lafayette IN 47907 USA jdick@purdue.edu crenault@purdue.edu; b Elmore Family School of Electrical and Computer Engineering, Purdue University West Lafayette IN 47907 USA

## Abstract

Droplet evaporation and dissolution phenomena are pervasive in both natural and artificial systems, playing crucial roles in various applications. Understanding the intricate processes involved in the evaporation and dissolution of sessile droplets is of paramount importance for applications such as inkjet printing, surface coating, and nanoparticle deposition, *etc*. In this study, we present a demonstration of electrochemical investigation of the dissolution behaviour in sub-nL droplets down to sub-pL volume. Droplets on an electrode have been studied for decades in the field of electrochemistry to understand the phase transfer of ions at the oil–water interface, accelerated reaction rates in microdroplets, *etc*. However, the impact of microdroplet dissolution on the redox activity of confined molecules within the droplet has not been explored previously. As a proof-of-principle, we examine the dissolution kinetics of 1,2-dichloroethane droplets (DCE) spiked with 155 μM decamethylferrocene within an aqueous phase on an ultramicroelectrode (*r* = 6.3 μm). The aqueous phase serves as an infinite sink, enabling the dissolution of DCE droplets while also facilitating convenient electrical contact with the reference/counter electrode (Ag/AgCl 1 M KCl). Through comprehensive voltammetric analysis, we unravel the impact of droplet dissolution on electrochemical response as the droplet reaches minuscule volumes. We validate our experimental findings by finite element modelling, which shows deviations from the experimental results as the droplet accesses negligible volumes, suggesting the presence of complex dissolution modes.

## Abstract 2

We quantified the dissolution rate of sub-nL oil microdroplets in a bulk aqueous phase based on the evolution of cyclic voltammograms recorded for a redox mediation confined in the microdroplet.

## Graphical abstract

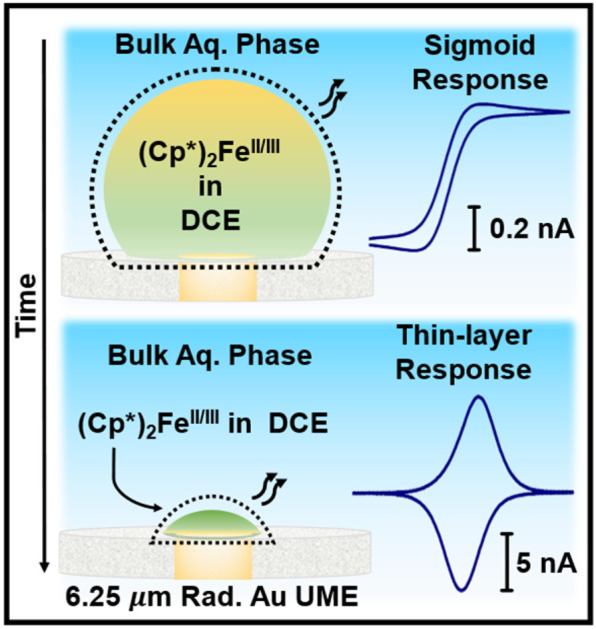

## Introduction

Droplets of sub-femtoliter volumes pervade nature; however, their physicochemical properties are strikingly unknown because of limitations in rigorous quantitative measurements. The past decade has witnessed growing consensus that chemical reactions proceed differently in droplets, which have the ability to accelerate reactions by up to 10^7^.^1–4^ Furthermore, droplets play a pivotal role in diverse scientific disciplines encompassing both physical and biological processes. The evaporation and dissolution of sessile droplets assumes a critical position in a wide array of industrial applications, ranging from agrochemical spraying of plants and desalination to DNA synthesis, evaporative lithography, inkjet printing, protein crystallography, and surface patterning.^5^ The study of dissolution kinetics in picoliter (pL) volumes, or smaller, presents significant challenges due to the small size of the droplets. Accurately measuring the dissolution rates and size changes of pL droplets requires techniques with high spatial and temporal resolution. The rapid nature of the process necessitates precise and high time resolution to capture dynamic changes effectively. The accelerated dissolution rates observed in pL droplets can be attributed to their high surface area-to-volume ratio, efficient diffusion, as well as the presence of concentration gradients and convective flows.^6^ Previous reports have emphasized the importance of droplet size in understanding how chemistry evolves within such small volumes.^7^ Therefore, studying the dissolution and evaporation of droplets may hold the key to unraveling the origins of life.^8^

**Fig. 1:**
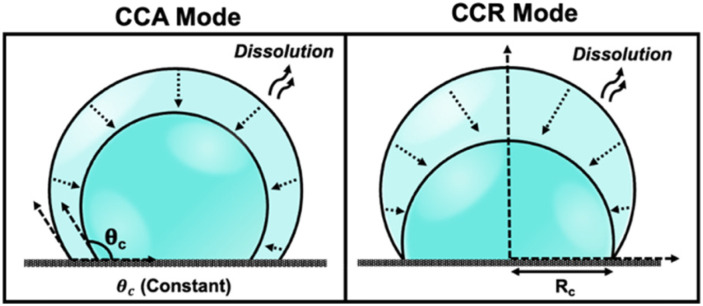
Schematic depicting various modes of droplet dissolution: Constant Contact Radius Mode (CCR) and Constant Contact Angle Mode (CCA).

The physics of droplet evaporation is a diffusion-limited phenomenon, where the diffusive transport of the droplet constituents in the form of vapor from the droplet into the atmosphere governs the evaporation process.^9^ A less studied, but completely analogous, system is that of a liquid droplet surrounded by another liquid. Herein, the dissolution process is also diffusion-limited, involving the diffusive transport of droplet constituents into the surrounding liquid phase. Both processes of evaporation and dissolution of droplets are purely diffusion-controlled and, therefore, can be explained by the same set of equations. The theoretical framework of the dissolution laws can be found in the ESI pg. no. S10.† In nature, however, droplets are rarely static and undergo dramatic changes as a result of evaporation or dissolution. Tracking the changes in the volume and lifetime of evaporating droplets depends on the mode of dissolution of the droplets. The most simple and extensively studied models in the field of evaporation and dissolution are the constant contact angle (CCA) and constant contact radius (CCR) modes.^10,11^ In CCA mode, the contact line is free to move, resulting in changes to the contact radius over time, while the contact angle (*θ*_c_) remains constant throughout the dissolution process. Whereas, in CCR mode, which is typically observed on rough substrates, involves a pinned contact line and a fixed contact radius (*R*_c_) that remains unchanged throughout the evaporation process. This leads to a uniform wetted area on the substrate, and eventually, the contact angle decreases to zero. The schematic shown in Fig. 1 describes the two extreme modes of dissolution *i.e.*, CCA and CCR mode of evaporation/dissolution. However, in reality, droplet dissolution does not occur exclusively in either of these modes but typically involves a combination of these extreme modes, referred to as the “mixed modes.” Several different kinds of mixed modes of dissolution have been reported like the stick-slide (SS) and stick-jump (SJ) modes.^5,12–14^ SS mode is characterized by a sequential combination of a CCR phase followed by a CCA phase.^15–20^ In this mode, droplets initially undergo evaporation/dissolution in the CCR mode until a specific contact angle is reached. Subsequently, the evaporation/dissolution process transitions to the CCA mode while maintaining a constant contact angle. The CCA mode may persist until the droplet is completely dissolved or may be followed by one or more subsequent stick-slide cycles. Stick-jump mode involves multiple (theoretically infinite) CCR phases separated by rapid (theoretically instantaneous) jump phases.^13–15,21^

Significant efforts have been dedicated in the field of electrochemistry, particularly in the study of droplets on an electrode in a bi-phasic system. These scenarios include instances where an oil droplet is placed on an electrode submerged in a bulk aqueous phase (or *vice versa*) or in the form of an oil–water emulsion where oil/water droplets stochastically collide with the electrode surface.^22–25^ Over the decades, electrochemistry has played a crucial role in investigating the redox activity of molecules confined within these droplets, elucidating the mechanisms of phase transfer of ions across the oil–water interface, and enabling the parsing out of partitioning kinetics, Gibbs free energy for the transfer of ions across the liquid|liquid interface, accelerated reaction rates in tiny microdroplets, *etc*.^1–3,22,24,26–30^ However, the impact of microdroplet dissolution on the redox activity of confined molecules within the droplet has not been explored previously. In this study, we present an electrochemical methodology for a thorough analysis of effect of dissolution of oil droplets down to sub-pL volume on the redox activity of confined molecules in the droplet. To validate the concept, we monitor the dissolution of a droplet composed of 1,2-dichloroethane (DCE) on a gold ultramicroelectrode (UME) in a aqueous bulk phase. The voltammetry of decamethylferrocene ((Cp*)_2_Fe^II^) undergoes significant changes as the DCE droplet dissolves. The voltammetry transition from an initial sigmoid (indicating bulk-like condition) to duck-shaped intermediates to a pair of bell-shaped voltammograms as the droplet dissolves with time. Using numerical simulations, we comprehensively capture the voltammetric evolution during the dissolution of the droplet. Accurate modeling of droplet dissolution requires a comprehensive understanding and integration of the pertinent physical mechanisms that govern the evaporation process occurring from the droplet's free surface into the surrounding atmosphere. Previous studies have revealed that droplets do not undergo dissolution through extreme modes, such as CCA or CCR. Instead, they exhibit a more intricate mode of dissolution, such as SS or SJ modes, when they are dissolving/evaporating on smooth substrates like glass.^31^ However, the numerical model proposed in this work is based on the use of extreme modes of dissolution to predict the geometry of the droplet dissolving on a Au UME. The Au UME comprises an exposed Au disk with a radius of 6.3 μm, enclosed within a glass sheath. The dissolution mode of the droplet is segmented into two stages: initially, it follows the CCA mode on the glass sheath, which is succeeded by the CCR mode as the three-phase contact boundary approaches the Au/glass interface. The simulation results, which rely on these simplified dissolution modes, can accurately capture the intricate changes observed on the voltammogram as the droplet dissolves over time in the CCA phase, and some discrepancies are observed in the CCR phase as the droplet shrinks to negligible volumes. This work establishes a platform for studying the dissolution of microdroplets using electrochemistry, offering advantages over conventional measurement tools like dynamic light scattering, atomic force microscopy, and optical microscopy in terms of temporal resolution, cost-effectiveness, quantitative precision, and overall convenience.

## Results & discussion

**Fig. 2:**
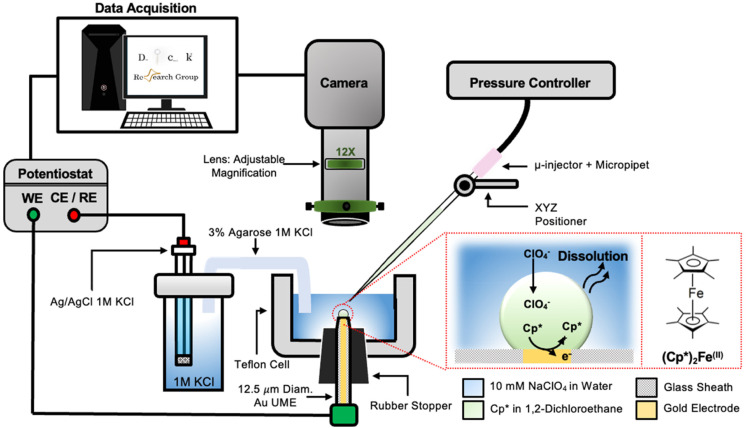
Cross sectional schematic of the setup used to visualize droplet dissolution while simultaneously recording their electrochemical response. A 6.3 μm radius gold microelectrode is held vertically in a Teflon cell (grey color) containing about 3 mL of 10 mM NaClO_4_ aqueous solution (light blue color). The gold micro disk is imaged with the camera positioned above the cell. The microinjection system is used to deliver about 0.55 nL of 155 μM (Cp*)_2_Fe^(II)^ in DCE just on top of the gold micro disk. The resulting DCE droplet is shown in green on the scheme. The electrochemical response is measured in a two-electrode configuration with a Ag/AgCl in 1 M KCl reference/counter electrode placed in a separate container and connected to the Teflon cell *via* a salt bridge. The reactions occurring inside the droplet during an anodic potential sweep are shown inside the red dotted frame. The chemical structure of the reduced form of redox molecule, (Cp*)_2_Fe^(II)^, is provided on the right. The DCE constantly dissolves into the water phase (wavy arrows) while the (Cp*)_2_Fe^(II)^ cannot escape the droplet and gets oxidized at the surface of the microelectrode. Charge balance inside the droplet is maintained by the transfer of ClO_4_^−^ across the water|DCE interface.

The setup used to simultaneously monitor the optical and electrochemical responses of a sub-nL droplet is shown in Fig. 2. A 6.3 μm radius (see optical micrograph Fig. S1†) disk gold electrode is held vertically inside a Teflon cell filled with ∼3 mL of 10 mM NaClO_4_ aqueous solution. A micro-injection system composed of a micropipette filled with (Cp*)_2_Fe^II/III^ in DCE, a XYZ micro-positioner and a compressor is used to dispense DCE above the center of gold micro-disk. The camera positioned above the cell allows both alignment of the micropipette with the gold micro-disk and monitoring of the droplet size as a function of time. Bright field optical micrograph of the surface of the electrode are acquired in reflectance with diffuse white light illumination. The electrochemical measurement is performed in a two-electrode configuration with the reference/counter electrode located in a separate reservoir containing 1 M KCl. This reservoir is connected to the cell *via* an agarose salt bridge. The electrochemical reactions inside the droplet are shown within the red frame in Fig. 2. The cyclic voltammogram recorded for the stock solution of (Cp*)_2_Fe^II/III^ used in the preparation of oil droplets reveals that the DCE phase initially contains a total of 155 μM of [(Cp*)_2_Fe^II^ + (Cp*)_2_Fe^III^], as determined through electrochemical measurements (see Fig. S2†). The chemical structure of (Cp*)_2_Fe^II^ is shown in the red dotted frame in Fig. 2. This molecule was chosen because it is particularly insoluble in water and expected to partition strongly in the DCE droplet. This molecule can be reversibly oxidized, with *n* = 1 electron, to (Cp*)_2_Fe^III^. A typical cyclic voltammogram of (Cp*)_2_Fe^II^ in bulk DCE is shown in Fig. S3.† As the potential is swept positively the oxidation of (Cp*)_2_Fe^II^ occurs at the surface of the gold electrode (yellow color in Fig. 2). Electro-neutrality inside the droplet is maintained by transfer of ClO_4_^−^ anions from the water phase into the DCE droplet.^32–34^

**Fig. 3:**
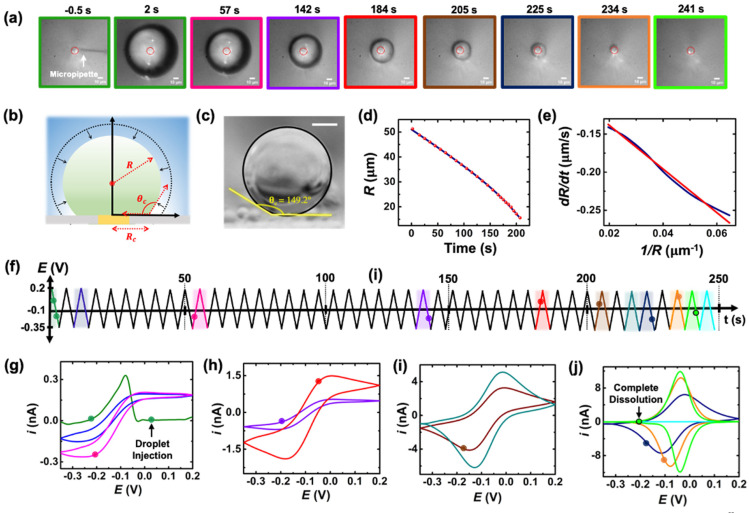
(a) Optical micrographs recorded during the dissolution of a 51 μm radius droplet containing 155 μM of (Cp*)_2_Fe^II^ in a 10 mM NaClO_4_ aqueous bulk phase. The solid red line incrusted in the micrographs indicates the position of the gold-disk electrode. The scale bar is 10 μm. (b) Side-view schematic of the droplet sitting on the electrode. (c) Side-view optical micrograph of a DCE droplet on a glass surface and immersed in water. The scale bar represents 10 μm. (d) Apparent droplet's radius as a function of time measured from the optical micrographs in (a). The data points shown in red represent the apparent radius of the droplet measured using the acquired micrographs. The solid blue curve represents a polynomial fit used to obtain a smooth representation of the data points. (e) Normal velocity of the water|DCE as a function of the inverse of the droplet's radius. The solid navy curve is a linear fit on the data, slope = −2.4 ± 0.003 cm^2^ s^−1^, *R*^2^ = 0.992. (f) Potential ramp applied during the dissolution of the droplet shown in (a). The colored segments in the potential ramp function correspond to the colored segments of the voltammogram in (g)–(j). Panels (g)–(j) show cyclic voltammograms recorded simultaneously with the optical micrographs during the dissolution of the DCE droplet at a scan rate of 0.2 V s^−1^. The color code used for the cyclic voltammograms matches the color code in (f). The points on the cyclic voltammogram indicate the exact time when the frames in (a) were taken. The voltammograms were smoothed using adjacent weighted averaging with 19 points per window and a Savitzky–Golay algorithm with 32 points per window, and a periodic boundary condition. See Fig. S4† for an overlay of experimentally obtained CV and noise filtered CV of the purple curve in (h).

A series of optical micrographs recorded during a typical experiment is shown in Fig. 3(a). The time, *t*, is referenced at 0 s when the DCE droplet is injected onto the gold micro-disk. The bright spot at the center of the first micrograph (*t* = −0.5 s, in Fig. 3(a)) is the surface of the gold disk electrode, in absence of the DCE droplet. A red solid circle was placed on all the micrographs to show the position of the electrode. The electrode is surrounded by a glass sheath. The micropipette can be seen on the right of the gold disk on the first micrograph. The injection of the droplet is performed between the first and second optical micrographs in Fig. 3(a). The injection is completed within about 1 s. A movie of the entire experiment showing the shrinking of the droplet is provided in ESI† (optical micrographs on the left side of Movie S1†). The series of micrograph shows that the DCE droplet shrinks and eventually completely dissolves into the water phase within 241 ± 2 s. The error value was determined based on error in time associated with the injection time and complete dissolution of the droplet. The initial apparent radius of the droplet is 51 ± 3 μm (second micrograph in Fig. 3(a)). The geometrical relation between the apparent droplet radius *R*, contact radius *R*_c_ and contact angle *θ*_c_ is shown in Fig. 3(b). For micron-sized droplets surface tension is stronger than gravitational forces (bond number = 2.98 × 10^−4^, see ESI pg. no. S6†) and thus the droplet always keeps a spherical curvature. Independent contact angle measurements for DCE droplets sitting on glass and immersed in water (see side-view optical micrograph Fig. 3(c)) provides a value of *θ*_c_ = 149 ± 3° (details about the measurement are provided in ESI pg. no. S8 and Fig. S5†). For *θ*_c_ ≥ 90°, the apparent radius corresponds to the actual radius of the droplet as shown in Fig. 3(b). The values of *R*_c_ and the volume of the droplet, *V*, can be calculated using basic geometric relation provided in ESI pg. no. S9.† For an initial value of *R* = 51 μm and *θ*_c_ = 149°, we estimate *R*_c_ = 26 μm and *V* = 0.55 nL.

Upon close examination of the optical micrographs in Fig. 3(a), it is apparent that the gold disk electrode remains entirely covered by the DCE droplet until complete dissolution. Additional insights into the dissolution dynamics, including the three-phase boundary surrounding the Au UME throughout the entire dissolution process, can be observed in Movie S1.† Thus, we concluded that the three-phase boundary must stop moving at the gold|glass interface, where both difference in surface tension between those two materials (glass|gold interface) and topographic defects may act as pinning centers thereby, allowing the remaining of the dissolution must occur in CCR mode.^5^Fig. 3(d) shows the radius of the droplet measured from the optical micrographs in Fig. 3(a) as a function of time shown by the red points and the navy curve is a fit to the point. Fig. 3(e) shows the normal velocity of the water|DCE boundary computed from differentiation of the fit (blue curve) in Fig. 3(d). The velocity (magnitude) increases from 0.14 μm s^−1^ up to 0.25 μm s^−1^ as the droplet radius drops from 51 μm to 15 μm.^15^ The velocity is plotted as a function of 1/*R*. The red curve is a linear adjustment of the diffusion-limited model of dissolution in CCA mode for a spherical sessile droplet (see details in ESI pg. no S11 and S12 for the slope value and diffusion coefficient calculation†). An excellent agreement is obtained between *t* = 0 s and *t* = 207 s. This last time (207 s, at which the we transition from a CCA mode to CCR mode of dissolution) corresponds to a droplet radius of 15 μm and a contact radius of 8 μm where the droplet is forced to switch from CCA to CCR mode. Note that the complete dissolution of the droplet takes ∼241 s but Fig. 3(d) and (e) accounts for only the first mode of dissolution *i.e.*, CCA. For a diffusion-limited dissolution process the eqn (S10)† establishes a link between the slope of the curve and the diffusion coefficient of DCE in water for CCA mode of dissolution. We obtain a value of *D*_DCE_ = 4.5 × 10^−6^ cm^2^ s^−1^. The obtained value is consistent with the values of *D*_DCE_ in water, 5–10 × 10^−6^ cm^2^ s^−1^, reported in the literature.^35,36^

During the entire course of dissolution the potential of the UME is continuously scanned at a scan rate of 0.2 V s^−1^ between −0.35 V and 0.2 V as shown in Fig. 3(f). This potential window is approximately centered around the apparent standard potential of the redox couple (Cp*)_2_Fe^II^/(Cp*)_2_Fe^III^ in bulk DCE, *E*′° = −0.10 V (see Fig. S3†). The duration of one cyclic voltammogram is 5.5 s. A total of 45 cyclic voltammograms are recorded during the experiment in Fig. 3, where the 45^th^ voltammogram marks the complete dissolution of the droplet. The colored points and segments in Fig. 3(g)–(j) correspond to the time when the optical micrographs in Fig. 3(a) were recorded. Recording of the micrographs and the voltammograms is started before injection of the droplet to measure a baseline current response. The injection appears as a sudden increase of current as shown by the green voltammogram in Fig. 3(g). Complete dissolution of the DCE droplet (∼242 s) appears as a sudden decrease of current as shown by the light green voltammogram in Fig. 3(j). The voltammograms recorded after 242 s (cyan curve in Fig. 3(j)) shows only charge/discharge of the electrochemical double layer and no detectable redox activity. Interestingly, the shape and intensity of the voltammograms constantly evolve as a function of droplet size, transitioning from an initial sigmoidal shape with anodic and cathodic current plateaus near 0.15/−0.1 nA (dark blue cyclic voltammograms in Fig. 3(g)) to duck-shaped intermediates and finally to a Gaussian-shaped pair of peaks reaching about ±10 nA close to the complete dissolution of the DCE droplet (light green cyclic voltammograms in Fig. 3(j)). The correlation between the variation of the response in voltammetry and the reduction of the droplet size was observed for several tens of droplets and is reproducible. Movie S1† (155 μM (Cp*)_2_Fe^II^; raw data with no noise filter of the data shown in Fig. 3) and Movie S2† (initial concentration (Cp*)_2_Fe^II/III^ was 1 mM) shows a side-by-side evolution of the voltammetric response and droplet size as a function of time for two completely independent experiments. The nuanced variations in the voltammograms result from the dynamic changes in the concentration of (Cp*)_2_Fe^II/III^ molecules confined within the droplet as it gradually shrinks over time. Consequently, we observe a sigmoid-shaped voltammogram when the droplet size is relatively large, resembling bulk-like conditions.^37^ However, as the concentration depletes in the proximity of the electrode, the voltammograms transition from a sigmoid shape to exhibit a peaking behavior. As the droplet further reduces in size to tiny volumes, a transition into a thin-layer regime occurs, where all the redox molecules within the droplet can be probed when the size of the droplet and the diffusive field are similar. This transition leads to the emergence of a bell-shaped pair of peaks. Quantitative insight and understand the intricate changes in voltammetry in the dissolving droplet was obtained using numerical simulation.

A 2D-axial time-dependent simulation was used to represent the voltammetric experiment. Diffusion of the (Cp*)_2_Fe^II^ and (Cp*)_2_Fe^III^ species inside the droplet was simulated by solving Fick's second law with a commercial Finite-Element Method package (COMSOL Multiphysics). The flux of redox species at the electrode boundary was described using the Butler–Volmer formalism for electron transfer rate. The current at the microelectrode was calculated by integrating the flux of species over the entire surface of the electrode. The simulation incorporated finite redox kinetics (*k*_0_ = 0.01 m s^−1^) for the redox couple, (Cp*)_2_Fe^II^/(Cp*)_2_Fe^III^. Dissolution of the DCE droplet was modeled using a deforming mesh and an Arbitrary Lagrangian–Eulerian method. The rate of deformation of the mesh is set by the rate of dissolution of the DCE into water. The mesh was deformed using simple geometric relationships (eqn (S18), (S19), (S25) and (S27) in ESI pg. no. S13 and S14†) that allows us to preserve the spherical geometry of the droplet and simultaneous account for the dissolution modes *i.e.*, CCA mode on the glass sheath and CCR mode as the three-phase boundary gets pinned at the Au|glass boundary (see ESI pg. no. S13 and S14† for the detailed derivation of mesh velocities).

The simulation was conducted in two phases, considering the extreme modes of dissolution, *i.e.*, CCA and CCR. In the first part, a CCA mode of dissolution was utilized, where the radius of the droplet changed with time, and the contact angle was fixed at 150°. Throughout this phase, the simulation parameterized the movement of the oil/water interface based on the measured radius values obtained from optics. The CCA mode was applied until the three-phase boundary reached the Au/glass interface and became pinned. Following this, the CCR mode was employed to depict droplet shrinking, accounting for changing contact angles and maintaining a fixed contact radius. It's essential to note that the only input parameter utilized in the entire simulation is the adjustment of the droplet geometry, specifically the droplet radius, against optical measurements in the first part of the simulation (CCA phase). In the absence of any partitioning of ferrocenyl species across the oil|water interface, the (Cp*)_2_Fe^II^ and (Cp*)_2_Fe^III^ species were confined during the dissolution of the droplet. This was done by adding a flux of both species directed toward the droplet and proportional to the velocity of the boundary and local concentration of species as the water|DCE boundary moves with time (shrinking of the droplet). The resistance of the solution and the intake of perchlorate ions inside the droplet was modeled by incorporating concentration-dependent ohmic drop and junction potential loss, respectively (see ESI† for the details of calculation for junction potential). After, it was ensured that the mass of ferrocenyl species was conserved in the droplet, a slow partition of the redox species from the DCE droplet to the bulk water phase was included in the model using a first order kinetic (*vide infra*). Details about the conservation of ferrocenyl species and slow partitioning are provided in ESI (pg. no. S16–19).† The detailed COMSOL report is provided in the ESI.†

**Fig. 4:**
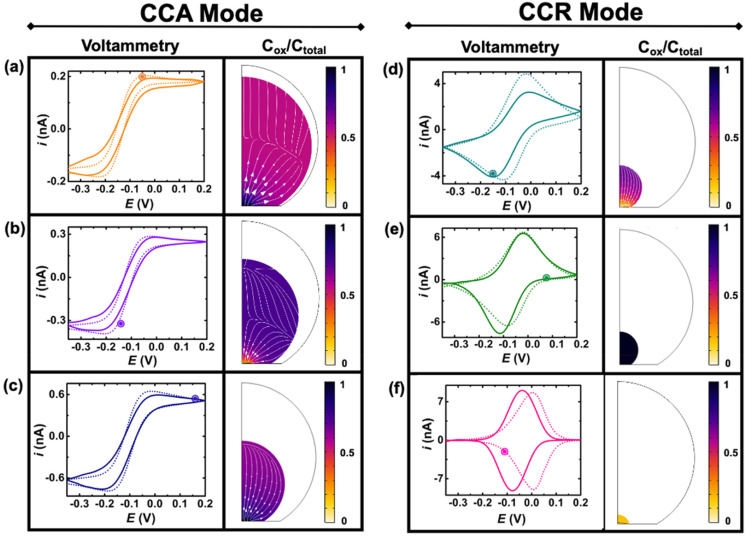
Comparison of experimental (continuous lines) and simulated (dashed lines) cyclic voltammograms during the dissolution of a DCE droplet. The color profiles represents the ratio [(Cp*)_2_ Fe^III^]/([(Cp*)_2_ Fe^II^] + [(Cp*)_2_ Fe^III^]), which is denoted as *C*_ox_/*C*_total_ for the sake of clarity. The arrows describe the flux of (Cp*)_2_Fe^III^. The concentration profiles and fluxes are taken at the time indicated by a circle on the voltammogram. The panels (a), (b), (c), (d), (e) and (f) correspond to the 6^th^ (*t* = 29 s, *C*_total_ = 0.197 mM), 17^th^ (*t* = 92 s, *C*_total_ = 0.373 mM), 26^th^ (*t* = 139 s, *C*_total_ = 0.719 mM) 38^th^ (*t* = 208 s, *C*_total_ = 4.76 mM), 41^st^ (*t* = 223 s, *C*_total_ = 8.22 mM), 43^rd^ (*t* = 235 s, *C*_total_ = 98 mM) cyclic voltammograms, respectively. The parameters of the simulation are: initial concentration of redox couple 155 μM, initial Ox/Red ratio 20/80, diffusion coefficients of (Cp*)_2_Fe^II^ and (Cp*)_2_Fe^III^ in DCE are 8.8 × 10^−6^ and 5.3 × 10^−6^ cm^2^ s^−1^, respectively, radius of electrode 6.3 μm, standard apparent potential −0.04 V, standard rate constant for electron transfer (*k*_0_ = 0.01 m s^−1^) and symmetry coefficient (*α* = 0.5), first order rate of loss for (Cp*)_2_Fe^III^ and (Cp*)_2_Fe^II^, 1.20 × 10^−6^ cm s^−1^ and 0.08 × 10^−6^ cm s^−1^. The initial serial resistance is set at 0.1 Gohm. The transition from CCA to CCR mode is set for a contact radius of 8 μm near the gold|glass interface.

Fig. 4 shows experimental (continuous lines) and simulated (dashed lines) cyclic voltammograms with normalized (Cp*)_2_Fe^III^ concentration (colormap) and flux (arrows) profiles at the time indicated by the marked point on the voltammograms. The concentration of (Cp*)_2_Fe^III^ (*C*_ox_) is normalized by the total concentration (*C*_total_) of ferrocenyl species and thus represents the ratio of oxidized form inside the droplet. The first and second columns show simulations during the CCA and CCR modes, respectively. An excellent agreement is obtained between the experiment and the simulation from the initial time until 236 s, when the volume of the droplet has shrunk from an initial volume of 0.55 nL to 0.5 pL. The simulation captures the appearance of peaks on the sigmoidal voltammograms as the droplet shrinks. A thin-layer regime with a bell-shaped voltammogram was also predicted when the size of the electrode and that of the diffusive field are similar. Movie S3 in ESI† shows side-by-side the voltammetry and concentration profile at all the times. At the beginning of the experiment (Fig. 3(a)) the droplet is about ten times larger than the electrode and the concentration gradient is primarily localized at the vicinity of the electrode and is barely reaching the water|DCE boundary. The voltammogram is thus adopting the sigmoidal shape typically measured in bulk for UMEs.^37^ It is nonetheless important to recognize that the voltammogram is sensitive to the change of volume of the droplet (and concentration). The smaller the droplet and the larger the current of both the anodic and cathodic plateaus/peaks. This can be easily understood with mass conservation. For a given amount of redox molecules trapped in the droplet, reducing the volume of this latter will inversely increase the average concentration of the former. The flux profiles shows a diffusional flux of both species starting from the water|DCE interface toward the center of the doplet (see flux lines in Fig. 4(a)–(c)). This flux is always positive and leads to an increase in concentration of the species. The increase in the total concentration of redox species (*C*_total_) during the dissolution of the droplet is mentioned in the Fig. 4 caption. On the other hand the flux of (Cp*)_2_Fe^III^ near the electrode can be either positive or negative depending on the potential of the electrode with respect to the apparent standard potential of the redox couple. However, it clear from CCR panel of Fig. 4 that there is a significant deviation between the simulated and experimental voltammograms in terms of the position and intensity of the redox peaks as the droplet shrinks to tiny volumes (Fig. 4(d)–(f)). We believe these discrepancies arise due to the simplistic assumptions in the formulation of the model like existence of extreme modes of dissolution, especially during the CCR phase. Moreover, in this work, it was assumed that diffusion is solely responsible as the transport mechanism of redox-active molecules. This assumption holds true when the concentration is low and sufficient supporting electrolyte is present, representing a scenario when the droplet is large. However, as the concentration ramps up, electromigration may also influence the transport phenomena, which hasn't been accounted for and may correlate with the discrepancies in the simulation results in the CCR mode. These difference may give rise to incongruencies between simulation and experiment in the cyclic voltammetry, especially when the droplet is smaller. However, it is crucial to emphasize on the fact that electrochemistry is sensitive to the volume changes at that scale. Further investigation into the complex modes of dissolution during the final moments of the droplet and incorporation of more complex electromigration phenomenon in the simulation represents a future avenue of our work.

**Fig. 5:**
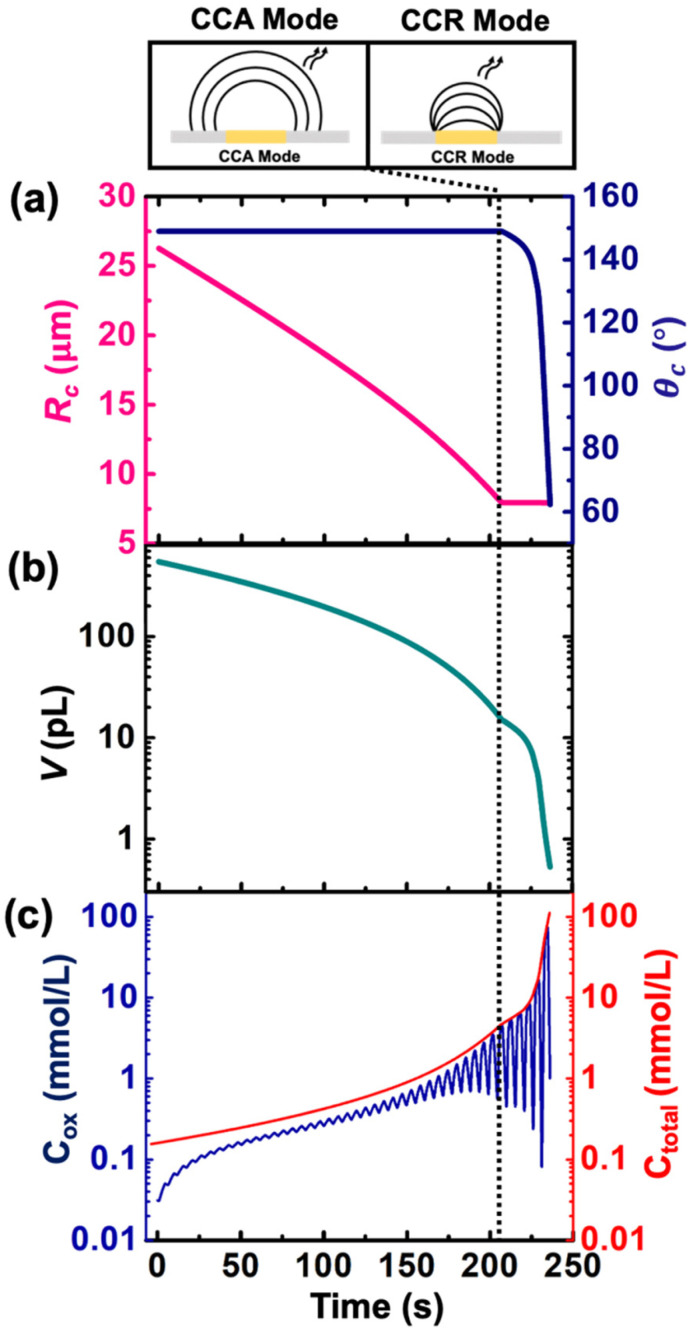
(a) Contact radius (left *Y* axis) and contact angle (right *Y* axis) used for the simulation of a DCE droplet dissolution in (b) simulated volume of the droplet as a function of time. (c) Simulated concentration of (Cp*)_2_ Fe^III^ (left axis) denoted as *C*_ox_ and total concentration of redox active species (right axis) denoted as *C*_total_ during the dissolution of the droplet. The vertical dotted line represents the transition from CCA to CCR mode.

Despite the simplistic assumptions and discrepancies in the predictions based on simulation in the CCR phase, the model allows us to determine several geometrical and electrochemical parameters like contact radius, contact angle, volume of the droplet, concentration of (Cp*)_2_Fe^II^/(Cp*)_2_Fe^III^ species, charge, partitioning kinetics of(Cp*)_2_Fe^II^/(Cp*)_2_Fe^III^ species, junction potential loss at a moving oil|water interface *etc.* as a function of time, which are not directly accessible from the experimental results. In CCA and CCR mode of dissolution, the velocity of the water|DCE interface was tracked based on the contact radius and contact angle, respectively. These quantities are plotted in Fig. 5(a) left and right *Y*-axis, respectively. The contact radius was calculated based on the micrographs recorded during the dissolution of the droplet (Fig. 3(e) and eqn (S5)†) and directly fed into the simulation allowing us to predict correctly the cyclic voltammograms during the entire CCA dissolution mode. It is crucial to note that this is the only free parameter introduced into the simulation to model the geometry of the dissolving droplet in the CCA phase. Note that after 207 s, the contact radius does not change due to pinning of the contact line at the Au|glass interface. Thus, the radius of the droplet as a function of time could also be an adjustable parameter of the simulation used to determine the kinetics of the droplet dissolution without the need of the optical measurement. In fact, in our experimental setup, the contact angle could not be measured directly from top-view optical observation. Therefore, to accurately predict the electrochemical signal during the CCR mode of dissolution, a manual adjustment based on the contact angle as a function of time was conducted in our simulation to reproduce correctly the electrochemical signal during the CCR mode of dissolution (contact angle remains constant (149°) during the CCA mode *i.e.*, from *t* = 0 s to 207 s). When referring to manual adjustments, we mean an informed input based on the theoretical equations used to model the dynamics of droplet dissolution. While the contact angle drops from 149° to 120° at an increasing rate, a constant rate of ∼16° s^−1^ is observed between 125° and 65°. This linear behavior is expected in CCR mode.^15^ It is crucial to realise that a total of 44 voltammograms were experimentally recorded before the complete dissolution of the droplet. The numerical simulation was performed until the 43^rd^ voltammogram due to our inability to account for the complex mechanisms associated with the complete dissolution of the droplet on the 44^th^ voltammogram. Therefore, despite Fig. 5(a) shows the final contact angle as 65°, in reality the droplet accesses contact angles approaching zero degrees during the final moments of its lifetime. A comparison of the theta *vs.* time used to capture the droplet geometry in the CCR phase is shown in Fig. 5(a) with a model for diffusion-limited dissolution of sessile droplet in CCR mode gives a value of *D*_DCE_ = 3 × 10^−6^ cm^2^ s^−1^ (see details in ESI pg. no. S11 and S12†). This value of is in agreement with the reported values in the literature.^35,36^ Note that there is a significant difference in the calculated value of *D*_DCE_ in water during the CCA and CCR phase. This discrepancy may be attributed to our inability to capture the electrochemistry during the CCR phase due to the simplistic assumption that the droplets dissolve in pure modes of dissolution, or the error in the measurement of the initial droplet radius. The equations used (eqn (S10) and (S13)†) to determine the diffusion coefficient are highly sensitive to the initial radius, and even a small error in initial radius manifests as a significant change in diffusion coefficients.

Fig. 5(b) and (c) shows the volume and concentration of (Cp*)_2_Fe^II/III^ species in the droplet as a function of time. The volume of the droplet was calculated based on the eqn (S3)† and geometrical parameters shown in Fig. 5(a). We emphasize the power of electrochemistry in being able to track the volume reduction across 3 orders of magnitude, requiring a log-scale. The volume decreases faster with time. A change of rate is seen at ∼207 s (vertical dotted black line) when the transition from CCA to CCR mode occurs. The effect of change in volume is evident on the concentration of ferrocenyl species confined in the droplet, as shown in Fig. 5(c). The total concentration is raising from an initial 155 μM up to almost 100 mM. This change of concentration by almost 3 orders of magnitude corresponds to the accumulation of the redox species as the volume shrinks. The *C*_ox_ ([(Cp*)_2_Fe^III^]), shown in blue on Fig. 5(c) is oscillatory in nature due to the nature of the voltage sweep. However, the overall concentration (*C*_total_) increases with time (solid red curve of Fig. 5(c)). Note that during CCR mode of dissolution, the oscillation in the *C*_ox_ curves touches the *C*_total_ ([(Cp*)_2_Fe^III^]+ [(Cp*)_2_Fe^II^]) curve shown in red. This is because the dimensions of electrode and diffusive fields are similar, thereby exhibiting thin-layer behaviour. In such a condition, all the redox molecules confined in the droplet can respond to the voltage sweep. This shows the ability of the methodology detailed in this work to be used as a sensing modality based on enrichment in analyte concentration in the dissolving droplet and represents another future avenue of our work. Nonetheless, it is interesting to ask: what is the minimum volume that can lead to a detectable electrochemical signal, or, in other words, when does the electrochemistry shut down? To answer this question, we draw our attention to the lifetime of the droplet.

Table 1 shows the lifetime of droplets measured using electrochemistry, optics and dissolution law (see pg. S20 in ESI† for the lifetime calculations using dissolution laws). The electrochemistry lifetime represents the time difference when the current rises above the baseline (at the injection point) and drops back to the baseline. The optical lifetime is determined by observing intensity contrast at injection until no variation in intensity (above baseline) is discernible in the micrographs. The theoretical lifetime of the droplet is determined using MATLAB, as shown in eqn (S33) and (S34) in the ESI pg. no. S20 and S21.† The last column is the theoretical lifetime based on the physico-chemical parameters found in the literature, the initial size of the droplet, and a diffusion-limited model. Experiments with two different initial concentrations of ferrocenyl compounds and different initial radii of the droplet are reported in Table 1. The experimental lifetimes are always shorter than the theoretical lifetime (between 3–25%, depending on the initial size of the droplet). These differences primarily arise from the use of extreme modes for dissolution to describe the shrinking volume of DCE droplets. In addition, the electrochemical lifetime is always longer than the optical lifetime during a point in the droplet's life where the volume is changing most quickly. While a longer theoretical lifetime may reflect our incapacity to observe the very last moment of the droplet dissolution (also error on initial size plus other phenomena like convection), it appears that electrochemistry allows tracking the dissolution process further than optical microscopy under our experimental conditions. In fact, the electrochemical signal grows larger as the droplet shrinks and concentrates the redox species. We hypothesize that the maximum concentrations must be limited by the solubility limit of the species. Beyond that concentration, the species are expected to precipitate. However, it must be noted that the time at which the solubility limit is reached is convoluted with the final volume that the droplet may access before complete dissolution. We measured the limit of solubility of (Cp*)_2_Fe^II^ in DCE to be around 125 mM. In the simulation results shown in Fig. 4, this limit is reached for a final volume of 0.5 pL. Moreover, we observed the presence of insoluble deposits at the electrode surface after the complete dissolution of the droplet across multiple experiments involving the use of higher initial concentration of (Cp*)_2_Fe^II^ species (see Movie S2†) in the DCE droplet.^37^

**Table 1:** Comparison of the lifetime of the droplets using optical measurement 
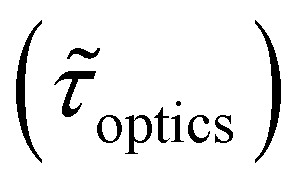
, electrochemistry 
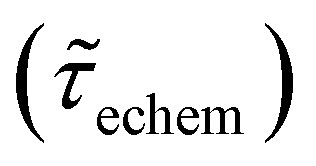
 and the dissolution laws 
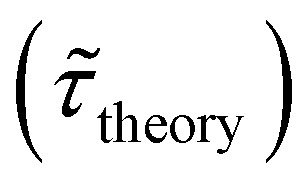
 as a function of initial radius of the droplet (*R*_*i*_) and total initial concentration of the (Cp*)_2_Fe^(II)^ and (Cp*)_2_Fe^(III)^ ([(Cp*)_2_Fe^(III)^ + (Cp*)_2_Fe^(II)^] = [C_Total_]) in DCE droplets

[*C*_total_] (mM)	*R*_*i*_ (μm)	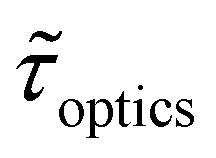 (s)	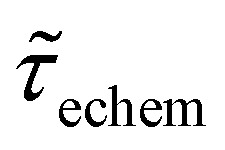 (s)	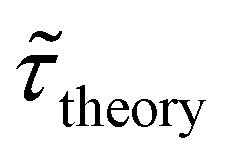 (s)
1	37	137	140	145
1	38	139	141	152
0.1	39	151	154	161
0.1	42	173	179	186
0.1	48	235	245	274
0.1	55	241	250	320
0.1	58	290	298	355

**Fig. 6:**
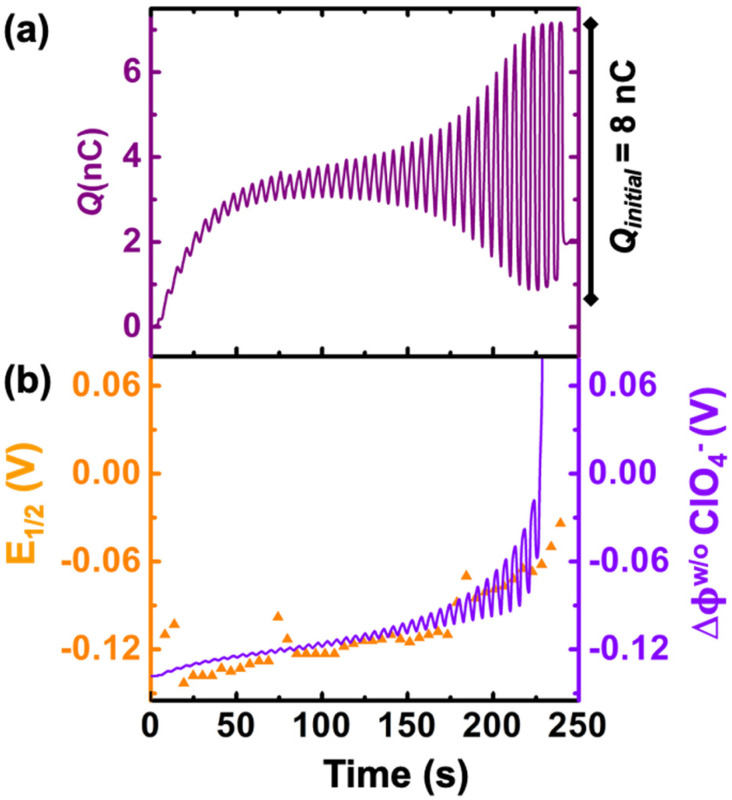
(a) Charge obtained by integrating the area under the experimental cyclic voltammograms. (b) experimental half-wave potential (*E*_1/2_, shown in orange) and simulated apparent standard potential corrected from the junction potential loss (*E*′°, shown in violet) during the course of the droplet dissolution.

We now examine other electrochemical quantities informing about the size of the droplet. Fig. 6(a) shows the charge (*Q*) calculated by integrating the area under the current of the cyclic voltammograms. The charge increases and presents oscillations that are also increasing in amplitude. Both the average charge and the amplitude of the oscillation tends toward a constant value between the 41^st^–42^nd^ cycles at about 220 s. The amplitude of the oscillation reaches a maximum of *Q*_max_ = 6.4 nC. The oscillations are cause by the cyclic potential ramp centered around the standard potential drawing alternatively anodic and cathodic currents. As the droplet shrinks, more of the volume can be probed during a single cyclic voltammogram. In the last three cycles all the volume is probed corresponding to the thin layer regime observed on the cyclic voltammograms in Fig. 3(j), 4(e) and (f). Thus, the maximum charge is proportional to the total content of (Cp*)_2_Fe^II/III^ inside the droplet. The electrochemical response is expected to reach a thin layer regime when the height of the droplet obeys 
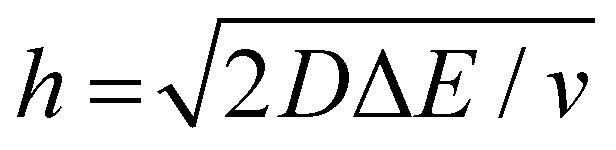
, where Δ*E*/*v* represents the duration of one potential sweep and thus the time during which oxidized/reduced species can explore the droplet by diffusion.^38^ Under our experimental condition, a value of *h* = 50 μm is calculated. Using Fig. 6(a), we deduce that the droplet height is approximately 50 μm at 220 s, in agreement with the full numerical simulation shown in Fig. 4(e). Once 
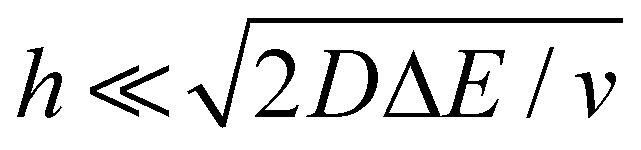
 mass transport is much faster than the time scale of the experiment. The voltammetric response resembles a pure thin-layer behavior bringing no information on the size of the droplet. In order to gain temporal resolution and track the dissolution of smaller droplet the scan rate can be increased. Another important piece of information brought by Fig. 6(a) is the total amount, *N*_tot_, of (Cp*)_2_Fe^III^ and (Cp*)_2_Fe^II^ species detected inside the droplet. According to Faraday's law the charge detected in the droplet *Q*_max_ = *nFN*_tot_, where *F* is the Fraday's constant (96 485 C mol^−1^). In our case we measured *N*_tot_ = 66 fmol while based on the initial concentration (155 μM) and volume of (0.55 nM) we expect 83 fmol. The difference indicates that 20% of the redox species were “lost” during the experiment. This loss is included in our simulation by using a first-order kinetic of transfer through the water|DCE interface. The first-order rate constants for (Cp*)_2_Fe^III^ and (Cp*)_2_Fe^II^ species are 1.20 × 10^−6^ cm s^−1^ and 0.08 × 10^−6^ cm s^−1^, respectively. A larger value for (Cp*)_2_Fe^III^ than (Cp*)_2_Fe^II^ is necessary to conserve the proportions of the anodic and cathodic peak in voltammetry. We hypothesize that the positively charged (Cp*)_2_Fe^III^ is more soluble in water than the neutral (Cp*)_2_Fe^II^ species.

Fig. 6(b) shows the half-wave potential, *E*_1/2_, (orange symbols, left axis) measured for all the 44 cyclic voltammograms recorded during the dissolution of the droplet. *E*_1/2_ was identified to the inflexion point for sigmoidal voltammograms and the average value of the peak potentials when pairs of anodic and cathodic peaks are appearing. The value of *E*_1/2_ shifts by +140 mV over the course of the experiment. We explain this shift as a result of junction potential at the water|DCE interface. Fig. 6(b) shows on the right *Y*-axis, the value of the simulated apparent standard potential corrected for the junction potential. The junction potential is computed assuming that perchlorate anions are the only ionic species crossing the interface to compensate the charge caused by the oxidation of (Cp*)_2_Fe^II^ to (Cp*)_2_Fe^III^ (see ESI pg. no. S16–S19 for detailed discussion†). We observed that in absence of counterion the electrochemical response disappears after few cyclic voltammograms suggesting that (Cp*)_2_Fe^III^ is forced to leave the droplet to maintain electroneutrality (see Fig. S12†). The electrochemical model describes correctly the average increase (more positive) of the apparent standard potential as the droplet shrinks.

The oscillations in the model are caused by the continuous fluctuation of the concentration of (Cp*)_2_Fe^III^. The experimental *E*_1/2_ values are not “instantaneous” measurements but rather a convolution of the potential shift over the duration (5.5 s) of an entire voltammogram. Thus, they do not display oscillations. The junction potential is expected to shift by 60 mV per decade of variation of perchlorate (and thus (Cp*)_2_Fe^III^) concentration inside the droplet. Based on Fig. 5(c), the total concentration is raising from an initial 155 μM up to almost 100 mM. This change of concentration by almost 3 orders of magnitude corresponds to the accumulation of the redox as the volume shrinks. This variation of concentration by three decades is correctly describing the potential shift from 0 s up to 236 s. Beyond this time extreme concentrations are reached and the shift of potential cannot be correctly predicted based by solely on the transfer of perchlorate ions (see Fig. 4(f)). We hypothesize that other ions (Na^+^ and (Cp*)_2_Fe^III^) could start transferring at the interface and other reactions such as ion pairing and precipitation may displace the equilibrium.

## Conclusion

In conclusion, this pioneering study investigates the dissolution behavior of sub-nL volume DCE droplets down to sub-pL volumes using electrochemistry. The comprehensive electrochemical analysis enables us to quantify the rate of droplet dissolution based on intricate changes observed in the nature of voltammograms, transitioning from sigmoid to “peaky” to thin-layer profiles. Additionally, this study sheds light on the ultimate fate of the droplet, allowing us to probe volume changes down to the sub-pL range and accurately track droplet lifetimes. The experimental findings are supported by numerical simulation. In principle, this methodology can be scaled to track the dissolution kinetics of nanodroplets by utilizing nanometer-sized electrodes. Furthermore, this study establishes a platform for testing wetting theories at the nanometer scale. The developed methodology surpasses other conventionally used measurement techniques like optics in terms of temporal resolution, cost, quantitative ability, and convenience. In a broader sense, this work contributes to the growing field of evaporating/ dissolving droplets, with application ranging from biosensing to microfluidic devices.^39–41^ Future research can delve into further understanding of complex dissolution phenomena and explore tailored strategies for understanding droplet dissolution behavior below the diffraction limit of light.

## Conflicts of interest

There are no conflicts to declare.

## Supplementary Material

AN-149-D4AN00299G-s001

AN-149-D4AN00299G-s002

AN-149-D4AN00299G-s003

AN-149-D4AN00299G-s004

AN-149-D4AN00299G-s005

## References

[cit1] Song X., Basheer C., Zare R. N. (2023). Making Ammonia from Nitrogen and Water Microdroplets. Proc. Natl. Acad. Sci. U. S. A..

[cit2] Lee J. K., Samanta D., Nam H. G., Zare R. N. (2019). Micrometer-Sized Water Droplets Induce Spontaneous Reduction. J. Am. Chem. Soc..

[cit3] Mehrgardi M. A., Mofidfar M., Zare R. N. (2022). Sprayed Water Microdroplets Are Able to Generate Hydrogen Peroxide Spontaneously. J. Am. Chem. Soc..

[cit4] Chen B., Xia Y., He R., Sang H., Zhang W., Li J., Chen L., Wang P., Gun S., Yin Y., Hu L., Song M., Liang Y., Wang Y., Jiang G., Zare R. N. (2022). Water–solid contact electrification causes hydrogen peroxide production from hydroxyl radical recombination in sprayed microdroplets. Proc. Natl. Acad. Sci. U. S. A..

[cit5] Wilson S. K., Ambrosio H.-M. D. (2022). Evaporation of Sessile Droplets. Annu. Rev. Fluid Mech..

[cit6] Hu H., Larson R. G. (2002). Evaporation of a Sessile Droplet on a Substrate. J. Phys. Chem. B.

[cit7] Jung Y., Marcus R. A. (2007). On the Theory of Organic Catalysis “on Water. J. Am. Chem. Soc..

[cit8] Holden D. T., As N., Morato M., Cooks R. G. (2022). Aqueous Microdroplets Enable Abiotic Synthesis and Chain Extension of Unique Peptide Isomers from Free Amino Acids. Proc. Natl. Acad. Sci. U. S. A..

[cit9] Popov Y. O. (2005). Evaporative Deposition Patterns: Spatial Dimensions of the Deposit. Phys. Rev. E: Stat., Nonlinear, Soft Matter Phys..

[cit10] Picknett R. G., Bexon R. (1977). The Evaporation of Sessile or Pendant Drops in Still Air. J. Colloid Interface Sci..

[cit11] Birdi K. S., Vu D. T., Winter A. (1989). A Study of the Evaporation Rates of Small Water Drops Placed on a Solid Surface. J. Phys. Chem..

[cit12] Dietrich E., Kooij E. S., Zhang X., Zandvliet H. J. W., Lohse D. (2015). Stick-Jump Mode in Surface Droplet Dissolution. Langmuir.

[cit13] Moffat J. R., Sefiane K., Shanahan M. E. R. (2009). Effect of TiO2 Nanoparticles on Contact Line Stick-Slip Behavior of Volatile Drops. J. Phys. Chem. B.

[cit14] Debuisson D., Merlen A., Senez V., Arscott S. (2016). Stick-Jump (SJ) Evaporation of Strongly Pinned Nanoliter Volume Sessile Water Droplets on Quick Drying, Micropatterned Surfaces. Langmuir.

[cit15] Stauber J. M., Wilson S. K., Duffy B. R., Sefiane K. (2014). On the Lifetimes of Evaporating Droplets. J. Fluid Mech..

[cit16] Bourges-Monnier C., Shanahan M. E. R. (1995). Influence of Evaporation on Contact Angle. Langmuir.

[cit17] Soolaman D. M., Yu H. Z. (2005). Water Microdroplets on Molecularly Tailored Surfaces: Correlation between Wetting Hysteresis and Evaporation Mode Switching. J. Phys. Chem. B.

[cit18] Semenov S., Starov V. M., Rubio R. G., Agogo H., Velarde M. G. (2012). Evaporation of Sessile Water Droplets in Presence of
Contact Angle Hysteresis. Math. Modell. Nat. Phenom..

[cit19] Nguyen T. A. H., Nguyen A. V., Hampton M. A., Xu Z. P., Huang L., Rudolph V. (2012). Theoretical and Experimental Analysis of Droplet Evaporation on Solid Surfaces. Chem. Eng. Sci..

[cit20] Dash S., Garimella S. V. (2013). Droplet Evaporation Dynamics on a Superhydrophobic Surface with Negligible Hysteresis. Langmuir.

[cit21] Adachi E., Dimitrov A. S., Nagayama K. (1995). Stripe Patterns Formed on a Glass Surface during Droplet Evaporation. Langmuir.

[cit22] Terry Weatherly C. K., Glasscott M. W., Dick J. E. (2020). Voltammetric Analysis of Redox Reactions and Ion Transfer in Water Microdroplets. Langmuir.

[cit23] Hermes M., Scholz F. (2000). The Electrochemical Oxidation of White Phosphorus at a Three-Phase Junction. Electrochem. Commun..

[cit24] Aoki K., Tasakorn P., Chen J. (2003). Electrode Reactions at Sub-Micron Oil | Water | Electrode Interfaces. J. Electroanal. Chem..

[cit25] Lovrić M. (2000). Diffusion from a Three-Phase Junction into a Hemispherical Droplet. Electrochem. Commun..

[cit26] Terry Weatherly C. K., Ren H., Edwards M. A., Wang L., White H. S. (2019). Coupled Electron- and Phase-Transfer Reactions at a Three-Phase Interface. J. Am. Chem. Soc..

[cit27] Fulian Q., Ball J. C., Marken F., Compton R. G., Fisher A. C. (2000). Voltammetry of Electroactive Oil Droplets. Part I: Numerical Modelling for Three Mechanistic Models Using the Dual Reciprocity Finite Element Method. Electroanalysis.

[cit28] Scholz F., Komorsky-Lovrić Š., Lovrić M. (2000). A new access to Gibbs energies of transfer of ions across liquid|liquid interfaces and a new method to study electrochemical processes at well-defined three-phase junctions. Electrochem. Commun..

[cit29] Lovrić M., Scholz F. (1997). A model for the propagation of a redox reaction through microcrystals. J. Solid State Electrochem..

[cit30] Scholz F. , Schroeder U. and Gulaboski R., Electrochemistry of Immobilized Particles and Droplets, Springer, 2015. 10.1007/978-3-319-10843-8

[cit31] Armstrong S., McHale G., Ledesma-Aguilar R., Wells G. G. (2019). Pinning-Free Evaporation of Sessile Droplets of Water from Solid Surfaces. Langmuir.

[cit32] Samec Z., Mareček V., Koryta J., Khalil M. W. (1977). Investigation of ion transfer across the interface between two immiscible electrolyte solutions by cyclic voltammetry. J. Electroanal. Chem. Interfacial Electrochem..

[cit33] Wandlowski T., Marecek V., Samec Z. (1990). Galvani potential scales for water—nitrobenzene and water-1,2-dichloroethane interfaces. Electrochim. Acta.

[cit34] Ding Z., Fermín D. J., Brevet P.-F., Girault H. H. (1998). Spectroelectrochemical Approaches to Heterogeneous Electron Transfer Reactions at the Polarised Water 1,2-Dichloroethane Interfaces. J. Electroanal. Chem..

[cit35] Chiao F. F. , Currie R. C., Chiao F. F., McKone T. E. and Hsieh D. P. H., Intermedia Transfer Factors for Contaminants Found at Hazardous Waste Sites 1,2 DICHLOROETHANE (DCA), Department of Environmental Toxicology University of California Davis, California, 1994

[cit36] Chemical Properties for Calculation of Impact to Ground Water Soil Remediation Standards, New Jersey Department of Environmental Protection, 2008. 10.7282/T3Z0385G

[cit37] Reyes-Morales J., Glasscott M. W., Pendergast A. D., Goines S., Dick J. E. (2022). The oxidation of ferrocene in sessile toluene macro-and microdroplets: An opto-electrochemical study. J. Electroanal. Chem..

[cit38] Bard A. J. and Faulkner L. R., Electrochemical Methods : Fundamentals and Applications, John Wiley & Sons, New York, 2001

[cit39] Dak P., Ebrahimi A., Alam M. A. (2015). Non-faradaic impedance characterization of a an evaporating droplet for microfluidic and biosensing application. Lab Chip.

[cit40] Ebrahimi A., Dak P., Salm E., Dash S., Garimella S. V., Bashir R., Alam M. A. (2013). Nanotextured superhydrophobic electrodes enable detection of attomolar-scale DNA concentration within a droplet by non-faradaic impedance spectroscopy. Lab Chip.

[cit41] Ebrahimi A., Csonka L. N., Alam M. A. (2018). Analysing thermal stability of cell membrane of salmonella using time-multiplexed impedance sensing. Biophys. J..

