# Supplementary material for: Understanding dynamic voltammetry in a dissolving microdroplet
Source: Analyst. 2024 Apr 30;149(15):3939–50. doi: 10.1039/d4an00299g (PMC11262062; doi:10.1039/d4an00299g)
Supplement: AN-149-D4AN00299G-s001 [file AN-149-D4AN00299G-s001.pdf]

Electronic Supplementary Material (ESI) for Analyst.
This journal is © The Royal Society of Chemistry 2024

COMSOL MULTIPHYSICS®

# Understanding Dynamic Voltammetry in a Dissolving Microdroplet

# Contents


1. **Global Definitions**
   1.1. Parameters
2. **Component 1**
   2.1. Definitions
   2.2. Geometry 1
   2.3. Transport of Diluted Species in droplet
   2.4. Moving Mesh
   2.5. Meshes
3. **Study 1 (CV 1 to 39)**
   3.1. Time Dependent
   3.2. Solver Configurations
4. **Study 2 (CV 40 to 44)**
   4.1. Time Dependent
   4.2. Solver Configurations
5. **Study 3 (CV 21 to 30)**
   5.1. Time Dependent
   5.2. Solver Configurations
6. **Study 4 (CV 31 to 38)**
   6.1. Time Dependent
   6.2. Solver Configurations
7. **Study 5 (CV 39 to 44)**
   7.1. Time Dependent
   7.2. Solver Configurations
8. **Results**
   8.1. Datasets
   8.2. Derived Values
   8.3. Tables
   8.4. Plot Groups

# 1 Global Definitions

## 1.1 PARAMETERS

### PARAMETERS 1

| Name | Expression | Value | Description |
|---|---|---|---|
| f | F_const/R_const/T | 38.941 1/V | |
| T | 298[K] | 298 K | temperature |
| r_elec | 6.35[um] | 6.35E−6 m | radius of the electrode |
| C0 | 0.155[mM] | 0.155 mol/m³ | initial concentration of redox (ox+red) |
| C0_ox_ini | C0*0.2 | 0.031 mol/m³ | initial conc. of ox in the cell |
| C0_red_ini | C0*0.8 | 0.124 mol/m³ | initial conc. of red in the cell |
| Dred | 8.8e-6[cm^2/s] | 8.8E−10 m²/s | diffusion coefficient of DmFc in DCE |
| Dox | 5.3e-6 [cm^2/s] | 5.3E−10 m²/s | diffusion coefficient of DmFc+ in DCE |
| alp | 0.5 | 0.5 | transfer coefficient for DmFc+/DmFc |
| k0 | 0.01[m/s] | 0.01 m/s | standard rate constant of electron transfer for DmFc+/DmFc |
| E0 | -0.04[V] | −0.04 V | apparent standard potential for DmFc+/DmFc |
| sr | 0.2[V/s] | 0.2 V/s | scan rate |
| Ei | -0.35[V] | −0.35 V | starting potential |
| Ef | 0.2[V] | 0.2 V | vertex potential |
| nb | 44 | 44 | number of CVs |
| t_tot | nb*2*abs(Ei - Ef)/sr | 242 s | duration of the experiment |
| t_cv | t_tot/nb | 5.5 s | duration of one CV |
| L | 51[um] | 5.1E−5 m | initial radius of the droplet |
| a | 149[deg] | 2.6005 rad | contact angle in degree |
| hg | -L*cos(a) | 4.3716E−5 m | initial height of the spherical cap |
| rc | L*cos((a - pi/2)) | 2.6267E−5 m | initial contact radius of |

| Name | Expression | Value | Description |
|---|---|---|---|
| | | | the droplet |
| C0_ClO4 | 0.01 [M] | 10 mol/m³ | initial concentration of ClO4 |
| Rs0 | 1E8[ohm] | 1E8 Ω | initial resistance of the cell |
| kloss_red | 0.08e-8 [m/s] | 8E−10 m/s | rate of DmFc tranfert from DCE to water |
| kloss_ox | 1.2e-8 [m/s] | 1.2E−8 m/s | rate of DmFc+ tranfert from DCE to water |
| D_water | 6E-6 [cm^2/s] | 6E−10 m²/s | diffusion coeff of DCE in water |
| C0_water | 55 [mol/L] | 55000 mol/m³ | concentration of pure water |
| Csat_DCEinWater | 90[mM] | 90 mol/m³ | concentration of DCE in water at saturation |
| Kp_DCE | C0_water/Csat_DCEinWater | 611.11 | partitioning coefficient between water and DCE |
| Vang | -0.043[rad/s] | −0.043 rad/s | angular dissolution rate in CCR mode |
| Vang_deg | -2.5[deg/s] | −0.043633 rad/s | angular dissolution rate in CCR mode |
| t_c | 200[s] | 200 s | transion time from CCA to CCR |
| V_ini | V_sphere-V_cap | 5.4755E−13 m³ | initial volume for contact angle larger than 90 deg |
| V_sphere | ((4/3)*pi*L^3) | 5.5565E−13 m³ | volume of sphere |
| V_cap | ((1/6)*pi*(L - hg)*(3*rc^2 + (L - hg)^2)) | 8.0971E−15 m³ | volume of small cap (contact angle smaller than 90 deg) |
| n_ini | 8.48246935651237E-14 [mol] | 8.4825E−14 mol | |
| E0ini | E0 + (R_const*T/F_const)*log( (C0_ox_ini)/C0_ClO4 ) | −0.18833 V | |

# 2 Component 1

| | |
|---|---|
| Date | Apr 3, 2023 2:12:39 PM |

SETTINGS

| Description | Value |
|---|---|
| Unit system | Same as global system (SI) |
| Geometry shape function | Automatic |

SPATIAL FRAME COORDINATES

| First | Second | Third |
|---|---|---|
| r | phi | z |

MATERIAL FRAME COORDINATES

| First | Second | Third |
|---|---|---|
| R | PHI | Z |

GEOMETRY FRAME COORDINATES

| First | Second | Third |
|---|---|---|
| Rg | PHIg | Zg |

MESH FRAME COORDINATES

| First | Second | Third |
|---|---|---|
| Rm | PHIm | Zm |

## 2.1 DEFINITIONS

### 2.1.1 Variables

**global variable**

SELECTION

| | |
|---|---|
| Geometric entity level | Entire model |

| Name | Expression | Unit | Description |
|---|---|---|---|
| n_Ox | intop2(cOx) | mol | amount of DmFc+ inside the droplet |
| n_Red | intop2(cRed) | mol | amount of DmFc inside the droplet |
| Vol | intop2(1*1*1) | $m^3$ | volume of the droplet |

| Name | Expression | Unit | Description |
| --- | --- | --- | --- |
| Surf | intop3(1*1) | m² | surface of the droplet (DCE/water boundary) |
| h | maxop1(z) | m | droplet's height |
| Rc | maxop1(r) | m | droplet's radius |
| r_c | maxop2(r) | m | contact radius |
| tetha | 2*atan(h/r_c) | rad | contact angle |
| Vr_CCR | ( (r_c/sin(tetha)) * ( 1 - cos(tetha) ) -z )*((z/sin(tetha))*Vang(t)) /r | m/s | 'r' component of mesh velocity in CCR |
| Vz_CCR | (z/sin(tetha))*Vang(t) | m/s | 'z' component of mesh velocity in CCR |
| Vr_CCA | Vn(t)*(r/Rc) | m/s | 'r' component of mesh velocity in CCA |
| Vz_CCA | Vn(t)*(z/Rc) | m/s | 'z' component of mesh velocity in CCA |
| DE | Rs0*(C0_ox_ini/(n_Ox/Vol + C0_ox_ini))*current | V | ohmic loss inside the droplet as a function of [DmFc+] |
| E0j | E0 + (R_const*T/F_const)*log( (n_Ox/Vol + C0_ox_ini)/C0_ClO4 ) | V | standard potential corrected from junction potential at water/DCE interface |
| current | F_const*Dred*intop1(cRedz) | A | current at the electrode |

### 2.1.2 Functions

#### Triangle 1

| | |
| --- | --- |
| Function name | tri1 |
| Function type | Triangle |

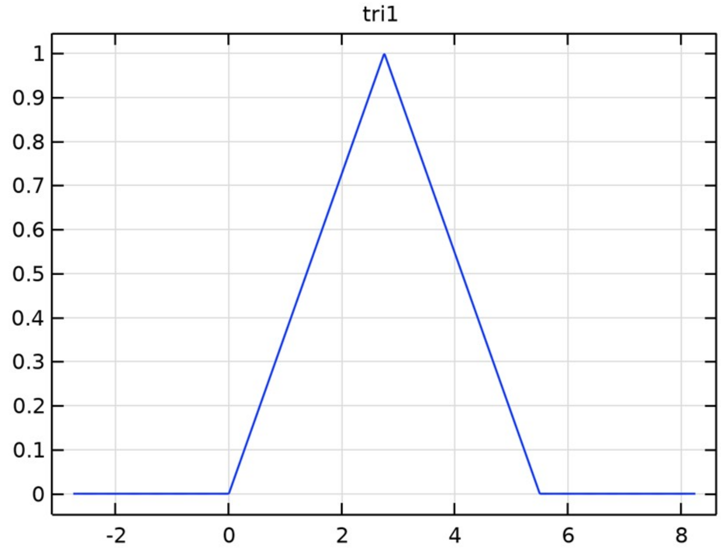


*Triangle 1*

PARAMETERS

| Description | Value |
|---|---|
| Lower limit | 0 |
| Upper limit | t_tot/nb |

SMOOTHING

| Description | Value |
|---|---|
| Size of transition zone | 0.001 |

**potential ramp**

| | |
|---|---|
| Function name | pot |
| Function type | Analytic |

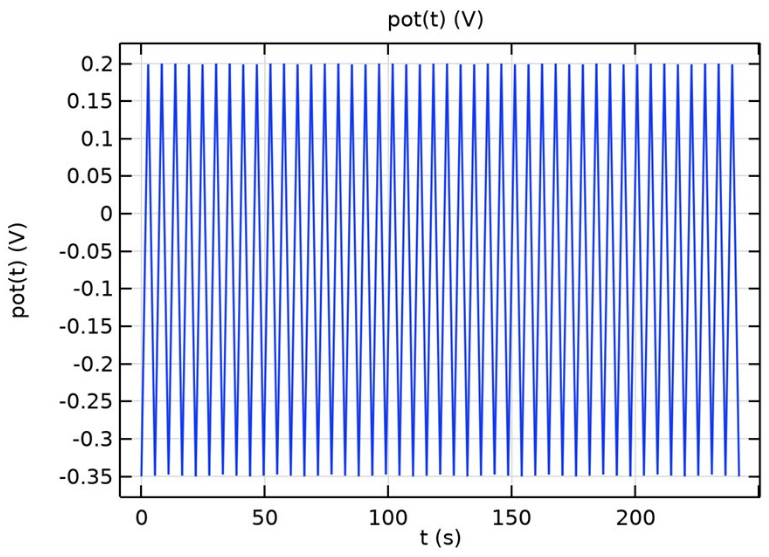


*potential ramp*

DEFINITION

| Description | Value |
|---|---|
| Expression | Ei + sign(Ef - Ei)*abs(Ei - Ef)*tri1(t) |
| Arguments | t |

PERIODIC EXTENSION

| Description | Value |
|---|---|
| Make periodic | On |
| Lower limit | 0 |
| Upper limit | t_tot/nb |

UNITS

| Description | Value |
|---|---|
| Arguments | s |
| Function | V |

**reduction rate**

| | |
|---|---|
| Function name | kred |
| Function type | Analytic |

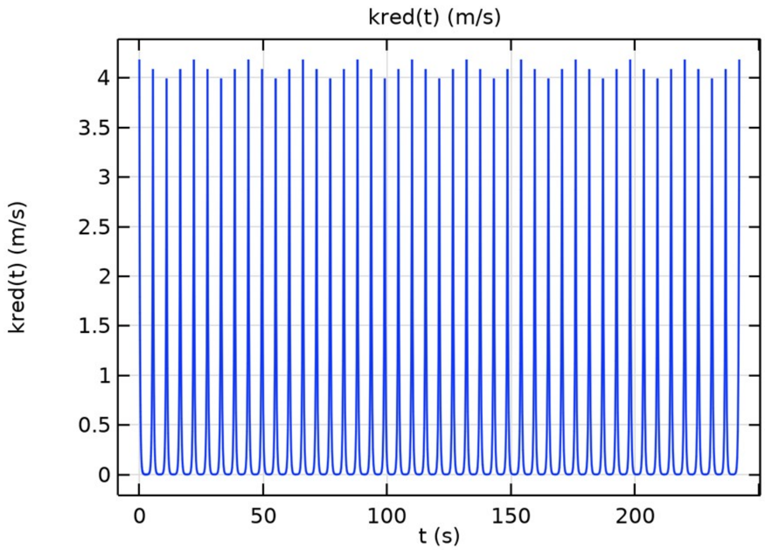


*reduction rate*

DEFINITION

| Description | Value |
| --- | --- |
| Expression | k0*exp(-alp*f*(pot(t) - E0)) |
| Arguments | t |

UNITS

| Description | Value |
| --- | --- |
| Arguments | s |
| Function | m/s |

### oxidation rate

| | |
| --- | --- |
| Function name | kox |
| Function type | Analytic |

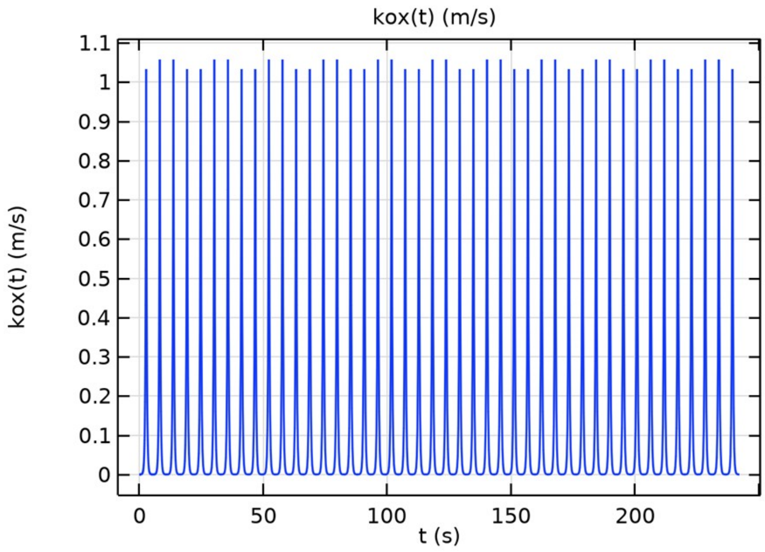


*oxidation rate*

DEFINITION

| Description | Value |
|---|---|
| Expression | k0*exp((1 - alp)*f*(pot(t) - E0)) |
| Arguments | t |

UNITS

| Description | Value |
|---|---|
| Arguments | s |
| Function | m/s |

### Interpolation o/w velocity CCA

| | |
|---|---|
| Function names | Vn |
| Function type | Interpolation |

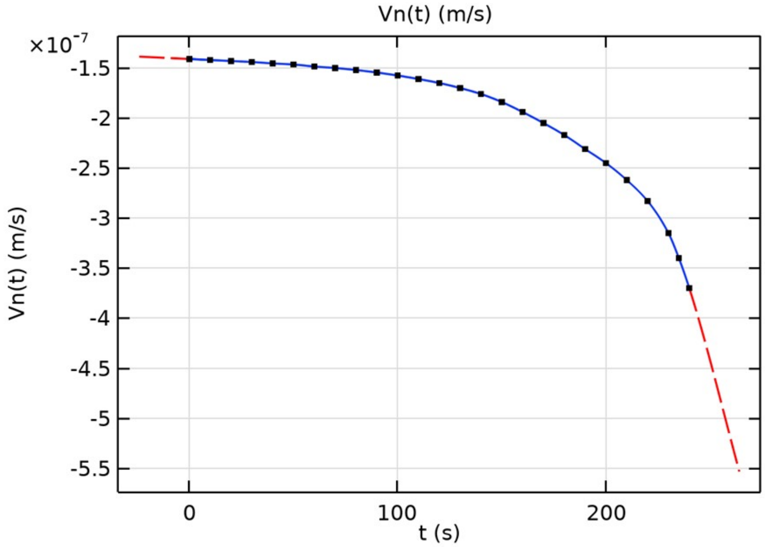


*Interpolation o/w velocity CCA*

### INTERPOLATION AND EXTRAPOLATION

| Description | Value |
|---|---|
| Interpolation | Piecewise cubic |
| Extrapolation | Nearest function |

### UNITS

| Description | Value |
|---|---|
| Arguments | s |
| Function | m/s |

## Interpolation o/w velocity CCR

| | |
|---|---|
| Function names | Vang |
| Function type | Interpolation |

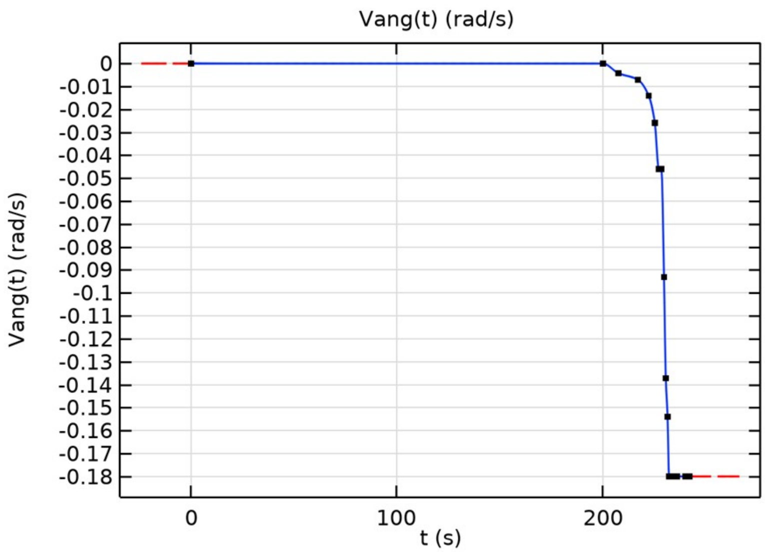


*Interpolation o/w velocity CCR*

### INTERPOLATION AND EXTRAPOLATION

| Description | Value |
|---|---|
| Interpolation | Piecewise cubic |
| Extrapolation | Nearest function |

### UNITS

| Description | Value |
|---|---|
| Arguments | s |
| Function | rad/s |

## o/w velocity from video

| | |
|---|---|
| Function name | Vn_video |
| Function type | Analytic |

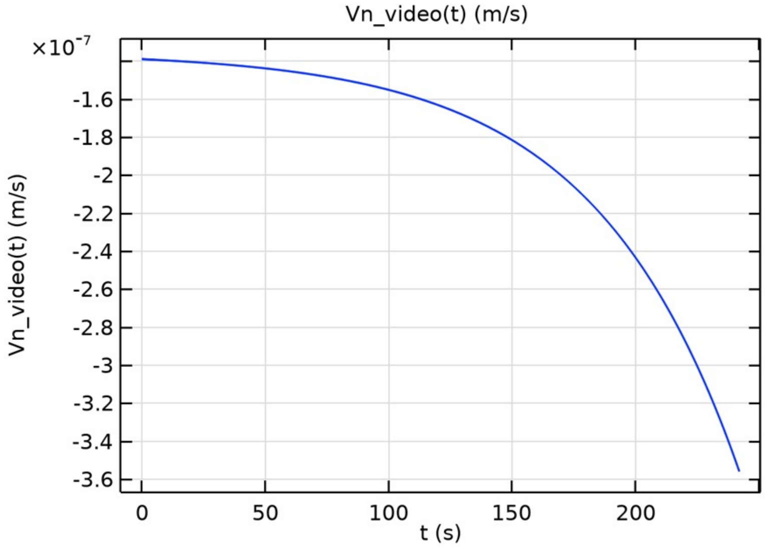


*o/w velocity from video*

DEFINITION

| Description | Value |
|---|---|
| Expression | -1e-6*((0.21279/58.87117)*exp(t/58.87117) + (579735/4282415)*exp(t/4282415)) |
| Arguments | t |

UNITS

| Description | Value |
|---|---|
| Arguments | s |
| Function | m/s |

### o/w radius from video

| | |
|---|---|
| Function name | Rn_video |
| Function type | Analytic |

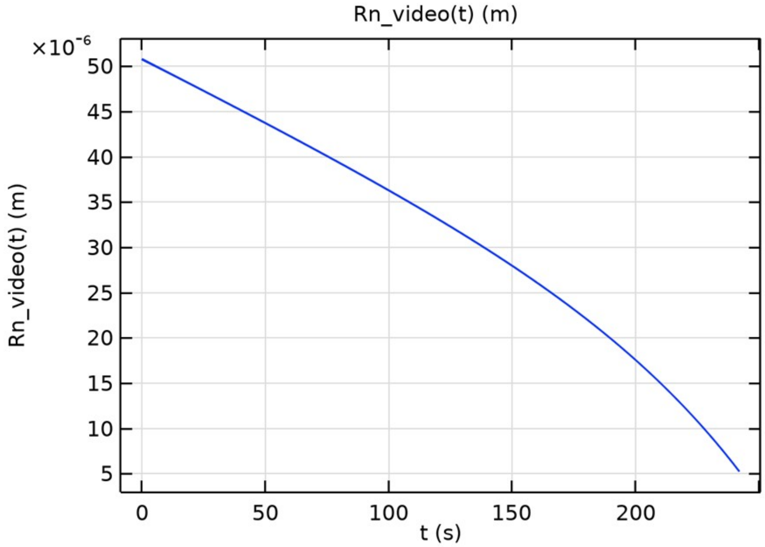


*o/w radius from video*

### DEFINITION

| Description | Value |
|---|---|
| Expression | 1e-6*(579786 - 0.21279*exp(t/58.87117) - 579735*exp(t/4282415)) |
| Arguments | t |

### UNITS

| Description | Value |
|---|---|
| Arguments | s |
| Function | m |

## contact radius in CCA

| | |
|---|---|
| Function name | R_contact |
| Function type | Analytic |

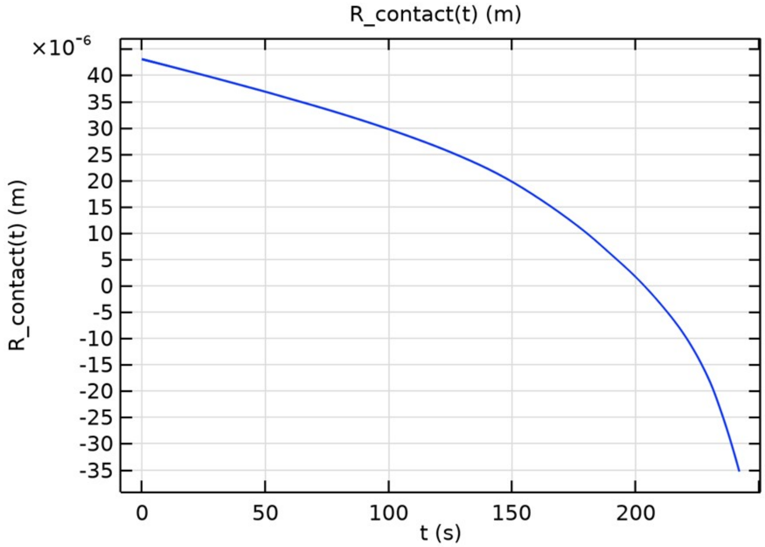


*contact radius in CCA*

DEFINITION

| Description | Value |
|---|---|
| Expression | (L + Vn(t)*t)*cos(a - 90) |
| Arguments | t |

UNITS

| Description | Value |
|---|---|
| Arguments | s |
| Function | m |

**compensation for mass loss**

| | |
|---|---|
| Function names | loss |
| Function type | Interpolation |

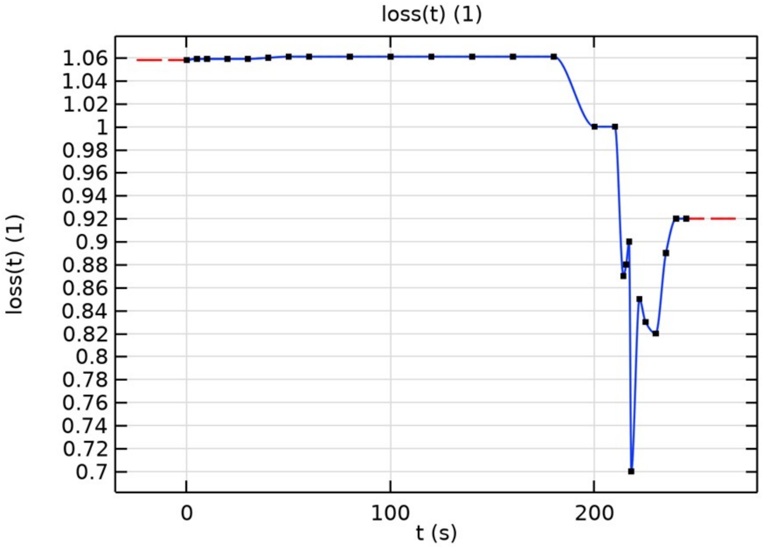


*compensation for mass loss*

INTERPOLATION AND EXTRAPOLATION

| Description | Value |
|---|---|
| Interpolation | Piecewise cubic |

UNITS

| Description | Value |
|---|---|
| Arguments | s |
| Function | 1 |

### 2.1.3 Nonlocal Couplings

**Integration over electrode surface**

| | |
|---|---|
| Coupling type | Integration |
| Operator name | intop1 |

SELECTION

| | |
|---|---|
| Geometric entity level | Boundary |
| Selection | Geometry geom1: Dimension 1: Boundary 2 |

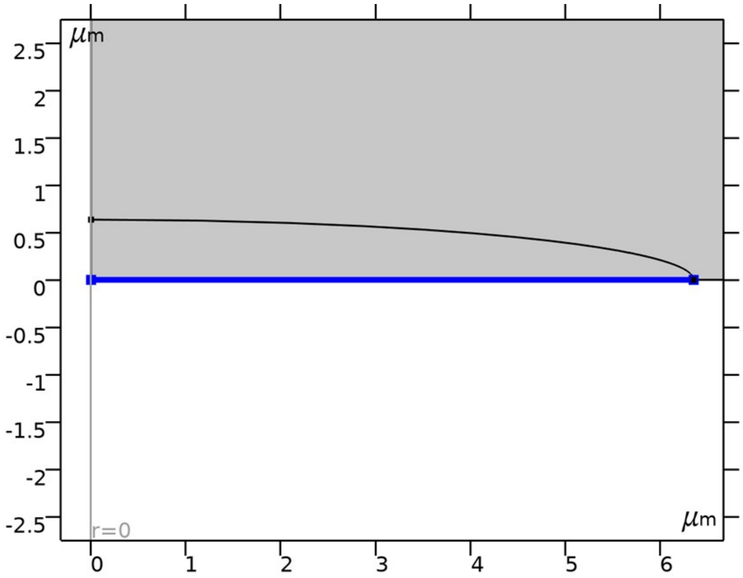


*Selection*

### ADVANCED

| Description | Value |
| --- | --- |
| Compute integral in revolved geometry | On |

## Integration over droplet volume

| | |
| --- | --- |
| Coupling type | Integration |
| Operator name | intop2 |

### SELECTION

| | |
| --- | --- |
| Geometric entity level | Domain |
| Selection | Geometry geom1: Dimension 2: Domains 1–2 |

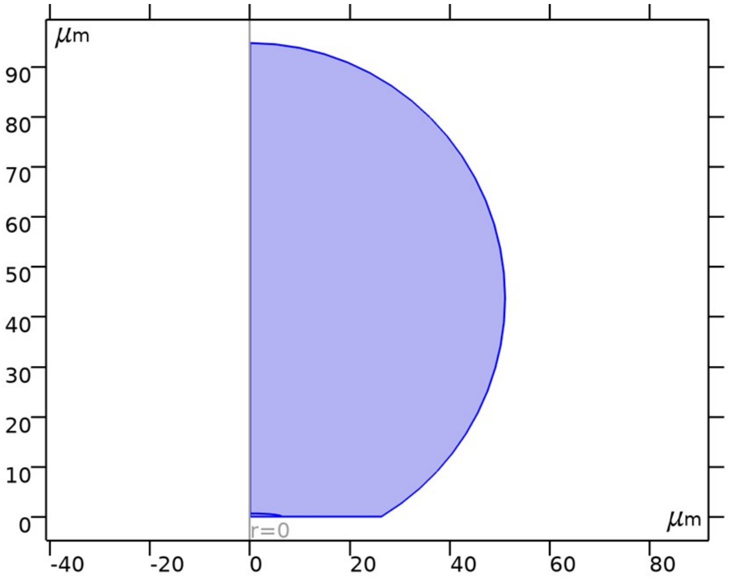


*Selection*

### ADVANCED

| Description | Value |
|---|---|
| Compute integral in revolved geometry | On |

## Integration over o/w surface

| | |
|---|---|
| Coupling type | Integration |
| Operator name | intop3 |

### SELECTION

| | |
|---|---|
| Geometric entity level | Boundary |
| Selection | Geometry geom1: Dimension 1: Boundaries 6–7 |

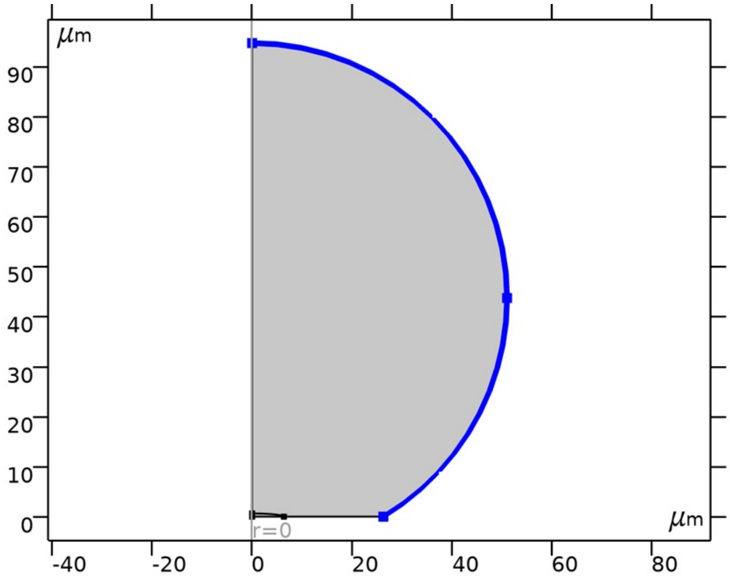


*Selection*

### ADVANCED

| Description | Value |
|---|---|
| Compute integral in revolved geometry | On |

## o/w arc

| | |
|---|---|
| Coupling type | Maximum |
| Operator name | maxop1 |

### SELECTION

| | |
|---|---|
| Geometric entity level | Boundary |
| Selection | Geometry geom1: Dimension 1: Boundaries 6–7 |

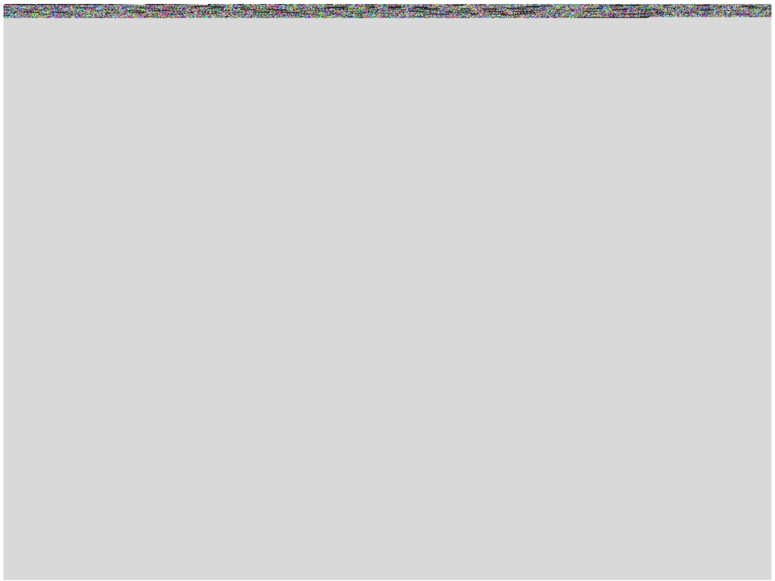

*Selection*

### contact line

| | |
|---|---|
| Coupling type | Maximum |
| Operator name | maxop2 |

#### SELECTION

| | |
|---|---|
| Geometric entity level | Point |
| Selection | Geometry geom1: Dimension 0: Point 5 |

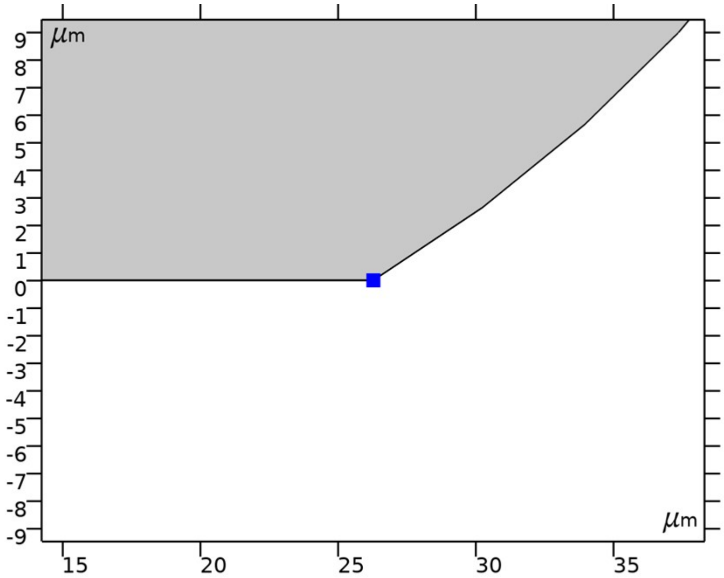


*Selection*

### 2.1.4 Coordinate Systems

#### Boundary System 1

| | |
|---|---|
| Coordinate system type | Boundary system |
| Tag | sys1 |

COORDINATE NAMES

| First | Second | Third |
|---|---|---|
| t1 | to | n |

## 2.2 GEOMETRY 1

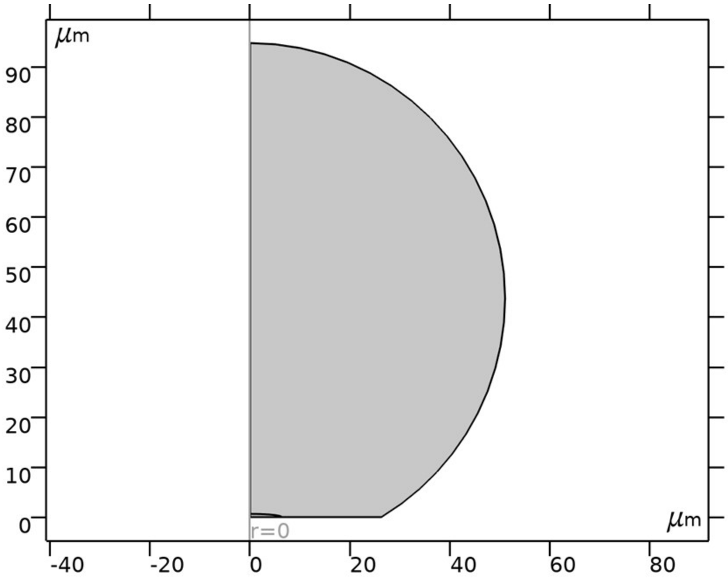


*Geometry 1*

UNITS

| | |
|---|---|
| Length unit | µm |
| Angular unit | rad |

GEOMETRY STATISTICS

| Description | Value |
|---|---|
| Space dimension | 2 |
| Number of domains | 2 |
| Number of boundaries | 7 |
| Number of vertices | 6 |

### 2.2.1 electrode (ls1)

SETTINGS

| Description | Value |
|---|---|
| Specify | Coordinates |
| Coordinates | {0, 0} |
| Specify | Coordinates |
| Coordinates | {r_elec, 0} |

### 2.2.2 droplet (c1)

POSITION

| Description | Value |
|---|---|
| Position | {0, hg} |

SIZE AND SHAPE

| Description | Value |
|---|---|
| Radius | L |
| Sector angle | 360 |

### 2.2.3 fixed diffusion layer (e1)

POSITION

| Description | Value |
|---|---|
| Position | {0, 0} |

SIZE AND SHAPE

| Description | Value |
|---|---|
| a-semiaxis | 1.0*r_elec |
| b-semiaxis | 0.1*r_elec |
| Sector angle | 360 |

### 2.2.4 Rectangle for subtraction (r1)

POSITION

| Description | Value |
|---|---|
| Position | {-10*L, -10*L} |

SIZE

| Description | Value |
|---|---|
| Width | 20*L |
| Height | 10*L |

### 2.2.5 Rectangle for subtraction 1 (r2)

POSITION

| Description | Value |
|---|---|
| Position | {-L, L} |
| Base | Center |

SIZE

| Description | Value |
|---|---|
| Width | 2*L |
| Height | 2*L |

## 2.3 TRANSPORT OF DILUTED SPECIES IN DROPLET

USED PRODUCTS

| |
|---|
| COMSOL Multiphysics |
| Chemical Reaction Engineering Module |

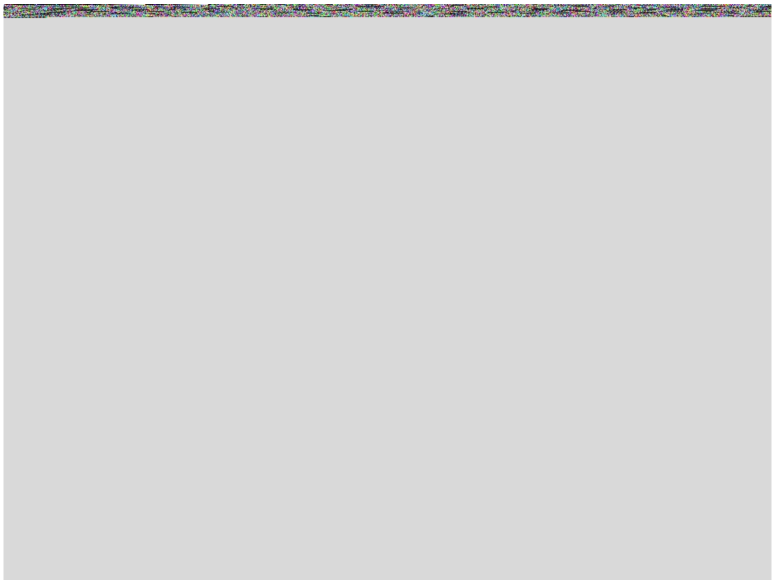

*Transport of Diluted Species in droplet*

SELECTION

| | |
|---|---|
| Geometric entity level | Domain |
| Selection | Geometry geom1: Dimension 2: Domains 1–2 |

EQUATIONS

$$\frac{\partial c_i}{\partial t}+\nabla\cdot\mathbf{J}_i=R_i$$

$$\mathbf{J}_i=-D_i\nabla c_i$$

### 2.3.1 Interface Settings

#### Discretization

SETTINGS

| Description | Value |
|---|---|
| Concentration | Linear |

#### Transport Mechanisms

SETTINGS

| Description | Value |
|---|---|
| Convection | Off |
| Migration in electric field | Off |
| Mass transfer in porous media | Off |

### 2.3.2 Variables

| Name | Expression | Unit | Description | Selection | Details |
|---|---|---|---|---|---|
| tds.R_cOx | 0 | mol/(m³·s) | Total rate expression | Domains 1–2 | + operation |
| tds.cP_cOx | 0 | mol/kg | Concentration species absorbed to the solid | Domains 1–2 | + operation |
| tds.cP_cOx | 0 | mol/kg | Concentration species absorbed to the solid | Boundaries 1–7 | + operation |
| tds.KP_cOx | 0 | m³/kg | Adsorption isotherm, first concentration derivative | Domains 1–2 | + operation |
| tds.KP_cOx | 0 | m³/kg | Adsorption isotherm, first concentration derivative | Boundaries 1–7 | + operation |
| tds.R_cRed | 0 | mol/(m³·s) | Total rate expression | Domains 1–2 | + operation |
| tds.cP_cRed | 0 | mol/kg | Concentration species absorbed to the solid | Domains 1–2 | + operation |
| tds.cP_cRed | 0 | mol/kg | Concentration species absorbed to the solid | Boundaries 1–7 | + operation |
| tds.KP_cRed | 0 | m³/kg | Adsorption isotherm, first | Domains 1–2 | + operation |

| Name | Expression | Unit | Description | Selection | Details |
|---|---|---|---|---|---|
| | | | concentration derivative | | |
| tds.KP_cRed | 0 | $m^3/kg$ | Adsorption isotherm, first concentration derivative | Boundaries 1–7 | + operation |
| tds.poro | 1 | 1 | Porosity | Domains 1–2 | |
| tds.theta_g | 0 | 1 | Gas volume fraction | Domains 1–2 | |
| tds.theta | tds.poro | 1 | Mobile fluid volume fraction | Domains 1–2 | |
| tds.nr | nr | 1 | Normal vector, r component | Boundary 5 | |
| tds.nphi | 0 | 1 | Normal vector, phi component | Boundary 5 | |
| tds.nz | nz | 1 | Normal vector, z component | Boundary 5 | |
| tds.nr | dnr | 1 | Normal vector, r component | Boundaries 1–4, 6–7 | |
| tds.nphi | 0 | 1 | Normal vector, phi component | Boundaries 1–4, 6–7 | |
| tds.nz | dnz | 1 | Normal vector, z component | Boundaries 1–4, 6–7 | |
| tds.nrmesh | nrmesh | 1 | Normal vector (mesh), r component | Boundary 5 | |
| tds.nphimesh | 0 | 1 | Normal vector (mesh), phi component | Boundary 5 | |
| tds.nzmesh | nzmesh | 1 | Normal vector (mesh), z component | Boundary 5 | |
| tds.nrmesh | dnrmesh | 1 | Normal vector (mesh), r component | Boundaries 1–4, 6–7 | |
| tds.nphimesh | 0 | 1 | Normal vector (mesh), phi component | Boundaries 1–4, 6–7 | |
| tds.nzmesh | dnzmesh | 1 | Normal vector (mesh), z component | Boundaries 1–4, 6–7 | |
| tds.nrc | root.nrc/tds.ncLen | 1 | Normal vector, r | Boundaries 1– | |

| Name | Expression | Unit | Description | Selection | Details |
|---|---|---|---|---|---|
| | | | component | 7 | |
| tds.nphic | 0 | 1 | Normal vector, phi component | Boundaries 1–7 | |
| tds.nzc | root.nzc/tds.ncLen | 1 | Normal vector, z component | Boundaries 1–7 | |
| tds.ncLen | sqrt(root.nrc^2+root.nzc^2+eps) | 1 | Help variable | Boundaries 1–7 | |

### 2.3.3 Transport Properties 1

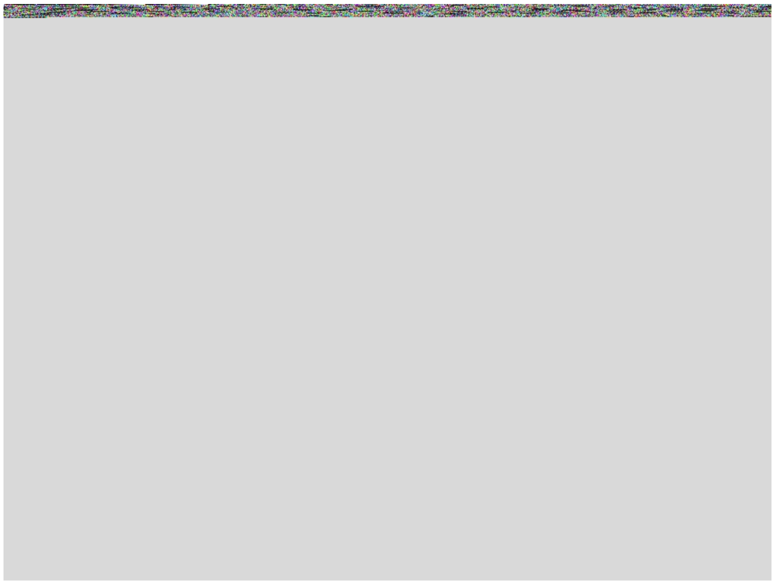

*Transport Properties 1*

SELECTION

| | |
|---|---|
| Geometric entity level | Domain |
| Selection | Geometry geom1: Dimension 2: All domains |

EQUATIONS

$$\frac{\partial c_i}{\partial t} + \nabla \cdot \mathbf{J}_i = R_i$$

$$\mathbf{J}_i = -D_i \nabla c_i$$

#### Diffusion

SETTINGS

| Description | Value |
|---|---|
| Source | Material |
| Material | None |

| Description | Value |
| --- | --- |
| Diffusion coefficient | User defined |
| Diffusion coefficient | {{Dox, 0, 0}, {0, Dox, 0}, {0, 0, Dox}} |
| Diffusion coefficient | User defined |
| Diffusion coefficient | {{Dred, 0, 0}, {0, Dred, 0}, {0, 0, Dred}} |

### Coordinate System Selection

#### SETTINGS

| Description | Value |
| --- | --- |
| Coordinate system | Global coordinate system |

### Model Input

#### SETTINGS

| Description | Value |
| --- | --- |
| Temperature | User defined |
| Temperature | T |

### Variables

| Name | Expression | Unit | Description | Selection | Details |
| --- | --- | --- | --- | --- | --- |
| domflux.cOxr | 2*tds.dflux_cOxr*pi*r | mol/(m·s) | Domain flux, r component | Domains 1–2 | |
| domflux.cOxz | 2*tds.dflux_cOxz*pi*r | mol/(m·s) | Domain flux, z component | Domains 1–2 | |
| domflux.cRedr | 2*tds.dflux_cRedr*pi*r | mol/(m·s) | Domain flux, r component | Domains 1–2 | |
| domflux.cRedz | 2*tds.dflux_cRedz*pi*r | mol/(m·s) | Domain flux, z component | Domains 1–2 | |
| tds.ndflux_cOx | tds.bndFlux_cOx | mol/(m²·s) | Normal diffusive flux | Boundaries 2, 4–7 | |
| tds.ntflux_cOx | tds.bndFlux_cOx | mol/(m²·s) | Normal total flux | Boundaries 2, 4–7 | |
| tds.ndflux_cRed | tds.bndFlux_cRed | mol/(m²·s) | Normal diffusive flux | Boundaries 2, 4–7 | |
| tds.ntflux_cRed | tds.bndFlux_cRed | mol/(m²·s) | Normal total flux | Boundaries 2, 4–7 | |
| tds.D_cOxrr | Dox | m²/s | Diffusion coefficient, rr component | Domains 1–2 | |
| tds.D_cOxphir | 0 | m²/s | Diffusion coefficient, phir | Domains 1–2 | |

| Name | Expression | Unit | Description | Selection | Details |
|---|---|---|---|---|---|
| | | | component | | |
| tds.D_cOxzr | 0 | m²/s | Diffusion coefficient, zr component | Domains 1–2 | |
| tds.D_cOxrphi | 0 | m²/s | Diffusion coefficient, rphi component | Domains 1–2 | |
| tds.D_cOxphiphi | Dox | m²/s | Diffusion coefficient, phiphi component | Domains 1–2 | |
| tds.D_cOxzphi | 0 | m²/s | Diffusion coefficient, zphi component | Domains 1–2 | |
| tds.D_cOxrz | 0 | m²/s | Diffusion coefficient, rz component | Domains 1–2 | |
| tds.D_cOxphiz | 0 | m²/s | Diffusion coefficient, phiz component | Domains 1–2 | |
| tds.D_cOxzz | Dox | m²/s | Diffusion coefficient, zz component | Domains 1–2 | |
| tds.D_cRedrr | Dred | m²/s | Diffusion coefficient, rr component | Domains 1–2 | |
| tds.D_cRedphir | 0 | m²/s | Diffusion coefficient, phir component | Domains 1–2 | |
| tds.D_cRedzr | 0 | m²/s | Diffusion coefficient, zr component | Domains 1–2 | |
| tds.D_cRedrphi | 0 | m²/s | Diffusion coefficient, rphi component | Domains 1–2 | |
| tds.D_cRedphiphi | Dred | m²/s | Diffusion coefficient, phiphi component | Domains 1–2 | |
| tds.D_cRedzphi | 0 | m²/s | Diffusion coefficient, zphi component | Domains 1–2 | |
| tds.D_cRedrz | 0 | m²/s | Diffusion | Domains 1–2 | |

| Name | Expression | Unit | Description | Selection | Details |
|---|---|---|---|---|---|
| | | | coefficient, rz component | | |
| tds.D_cRedphiz | 0 | m²/s | Diffusion coefficient, phiz component | Domains 1–2 | |
| tds.D_cRedzz | Dred | m²/s | Diffusion coefficient, zz component | Domains 1–2 | |
| tds.Dav_cOx | 0.5*(tds.D_cOxrr+tds.D_cOxzz) | m²/s | Average diffusion coefficient | Domains 1–2 | |
| tds.Dav_cRed | 0.5*(tds.D_cRedrr+tds.D_cRedzz) | m²/s | Average diffusion coefficient | Domains 1–2 | |
| tds.tflux_cOxr | tds.dflux_cOxr | mol/(m²·s) | Total flux, r component | Domains 1–2 | + operation |
| tds.tflux_cOxphi | tds.dflux_cOxphi | mol/(m²·s) | Total flux, phi component | Domains 1–2 | + operation |
| tds.tflux_cOxz | tds.dflux_cOxz | mol/(m²·s) | Total flux, z component | Domains 1–2 | + operation |
| tds.dfluxMag_cOx | sqrt(tds.dflux_cOxr^2+tds.dflux_cOxphi^2+tds.dflux_cOxz^2) | mol/(m²·s) | Diffusive flux magnitude | Domains 1–2 | |
| tds.tfluxMag_cOx | sqrt(tds.tflux_cOxr^2+tds.tflux_cOxphi^2+tds.tflux_cOxz^2) | mol/(m²·s) | Total flux magnitude | Domains 1–2 | |
| tds.dpflux_cOxr | 0 | mol/(m²·s) | Dispersive flux, r component | Domains 1–2 | |
| tds.dpflux_cOxphi | 0 | mol/(m²·s) | Dispersive flux, phi component | Domains 1–2 | |
| tds.dpflux_cOxz | 0 | mol/(m²·s) | Dispersive flux, z component | Domains 1–2 | |
| tds.tflux_cRedr | tds.dflux_cRedr | mol/(m²·s) | Total flux, r component | Domains 1–2 | + operation |
| tds.tflux_cRedphi | tds.dflux_cRedphi | mol/(m²·s) | Total flux, phi component | Domains 1–2 | + operation |
| tds.tflux_cRedz | tds.dflux_cRedz | mol/(m²·s) | Total flux, z component | Domains 1–2 | + operation |
| tds.dfluxMag_cRed | sqrt(tds.dflux_cRedr^2+tds.dflux_cRedphi^2+tds.dflux_cRedz^2) | mol/(m²·s) | Diffusive flux magnitude | Domains 1–2 | |
| tds.tfluxMag_cRe | sqrt(tds.tflux_cRedr | mol/(m²·s) | Total flux | Domains 1–2 | |

| Name | Expression | Unit | Description | Selection | Details |
|---|---|---|---|---|---|
| d | ^2+tds.tflux_cRedphi^2+tds.tflux_cRedz^2) | | magnitude | | |
| tds.dpflux_cRedr | 0 | mol/(m²·s) | Dispersive flux, r component | Domains 1–2 | |
| tds.dpflux_cRedphi | 0 | mol/(m²·s) | Dispersive flux, phi component | Domains 1–2 | |
| tds.dpflux_cRedz | 0 | mol/(m²·s) | Dispersive flux, z component | Domains 1–2 | |
| cOxrt | cOxrTIME-cOxrr*d(r,TIME)-cOxrz*d(z,TIME) | mol/(m⁴·s) | Gradient of cOx, r component, first time derivative | Domains 1–2 | |
| cOxzt | cOxzTIME-cOxzr*d(r,TIME)-cOxzz*d(z,TIME) | mol/(m⁴·s) | Gradient of cOx, z component, first time derivative | Domains 1–2 | |
| cOxrtt | d(cOxrTIME-cOxrr*d(r,TIME)-cOxrz*d(z,TIME),TIME)-d(cOxrTIME-cOxrr*d(r,TIME)-cOxrz*d(z,TIME),r)*d(r,TIME)-d(cOxrTIME-cOxrr*d(r,TIME)-cOxrz*d(z,TIME),z)*d(z,TIME) | mol/(m⁴·s²) | Gradient of cOx, r component, second time derivative | Domains 1–2 | |
| cOxztt | d(cOxzTIME-cOxzr*d(r,TIME)-cOxzz*d(z,TIME),TIME)-d(cOxzTIME-cOxzr*d(r,TIME)-cOxzz*d(z,TIME),r)*d(r,TIME)-d(cOxzTIME-cOxzr*d(r,TIME)-cOxzz*d(z,TIME),z)*d(z,TIME) | mol/(m⁴·s²) | Gradient of cOx, z component, second time derivative | Domains 1–2 | |
| cOxt | cOxTIME-cOxr*d(r,TIME)-cOxz*d(z,TIME) | mol/(m³·s) | Concentration, first time derivative | Domains 1–2 | |
| cOxtt | d(cOxTIME-cOxr*d(r,TIME)-cOxz*d(z,TIME),TIME | mol/(m³·s²) | Concentration, second time derivative | Domains 1–2 | |

| Name | Expression | Unit | Description | Selection | Details |
|---|---|---|---|---|---|
| | )-d(cOxTIME-cOxr*d(r,TIME)-cOxz*d(z,TIME),r)*d(r,TIME)-d(cOxTIME-cOxr*d(r,TIME)-cOxz*d(z,TIME),z)*d(z,TIME) | | | | |
| tds.dflux_cOxr | -tds.D_cOxrr*cOxr-tds.D_cOxrz*cOxz | mol/(m²·s) | Diffusive flux, r component | Domains 1–2 | |
| tds.dflux_cOxphi | -tds.D_cOxphir*cOxr-tds.D_cOxphiz*cOxz | mol/(m²·s) | Diffusive flux, phi component | Domains 1–2 | |
| tds.dflux_cOxz | -tds.D_cOxzr*cOxr-tds.D_cOxzz*cOxz | mol/(m²·s) | Diffusive flux, z component | Domains 1–2 | |
| tds.grad_cOxr | cOxr | mol/m⁴ | Concentration gradient, r component | Domains 1–2 | |
| tds.grad_cOxphi | 0 | mol/m⁴ | Concentration gradient, phi component | Domains 1–2 | |
| tds.grad_cOxz | cOxz | mol/m⁴ | Concentration gradient, z component | Domains 1–2 | |
| cRedrt | cRedrTIME-cRedrr*d(r,TIME)-cRedrz*d(z,TIME) | mol/(m⁴·s) | Gradient of cRed, r component, first time derivative | Domains 1–2 | |
| cRedzt | cRedzTIME-cRedzr*d(r,TIME)-cRedzz*d(z,TIME) | mol/(m⁴·s) | Gradient of cRed, z component, first time derivative | Domains 1–2 | |
| cRedrtt | d(cRedrTIME-cRedrr*d(r,TIME)-cRedrz*d(z,TIME),TIME)-d(cRedrTIME-cRedrr*d(r,TIME)-cRedrz*d(z,TIME),r)*d(r,TIME)-d(cRedrTIME-cRedrr*d(r,TIME)-cRedrz*d(z,TIME),z)*d(z,TIME) | mol/(m⁴·s²) | Gradient of cRed, r component, second time derivative | Domains 1–2 | |
| cRedztt | d(cRedzTIME-cRedzr*d(r,TIME)- | mol/(m⁴·s²) | Gradient of cRed, z component, | Domains 1–2 | |

| Name | Expression | Unit | Description | Selection | Details |
|---|---|---|---|---|---|
| | cRedzz*d(z,TIME),TIME)-d(cRedzTIME-cRedzr*d(r,TIME)-cRedzz*d(z,TIME),r)*d(r,TIME)-d(cRedzTIME-cRedzr*d(r,TIME)-cRedzz*d(z,TIME),z)*d(z,TIME) | | second time derivative | | |
| cRedt | cRedTIME-cRedr*d(r,TIME)-cRedz*d(z,TIME) | mol/(m³·s) | Concentration, first time derivative | Domains 1–2 | |
| cRedtt | d(cRedTIME-cRedr*d(r,TIME)-cRedz*d(z,TIME),TIME)-d(cRedTIME-cRedr*d(r,TIME)-cRedz*d(z,TIME),r)*d(r,TIME)-d(cRedTIME-cRedr*d(r,TIME)-cRedz*d(z,TIME),z)*d(z,TIME) | mol/(m³·s²) | Concentration, second time derivative | Domains 1–2 | |
| tds.dflux_cRedr | -tds.D_cRedrr*cRedr-tds.D_cRedrz*cRedz | mol/(m²·s) | Diffusive flux, r component | Domains 1–2 | |
| tds.dflux_cRedphi | -tds.D_cRedphir*cRedr-tds.D_cRedphiz*cRedz | mol/(m²·s) | Diffusive flux, phi component | Domains 1–2 | |
| tds.dflux_cRedz | -tds.D_cRedzr*cRedr-tds.D_cRedzz*cRedz | mol/(m²·s) | Diffusive flux, z component | Domains 1–2 | |
| tds.grad_cRedr | cRedr | mol/m⁴ | Concentration gradient, r component | Domains 1–2 | |
| tds.grad_cRedphi | 0 | mol/m⁴ | Concentration gradient, phi component | Domains 1–2 | |
| tds.grad_cRedz | cRedz | mol/m⁴ | Concentration gradient, z component | Domains 1–2 | |
| tds.bndFlux_cOx | if(r>0.001/sqrt(sqrt( | mol/(m²·s) | Boundary flux | Boundaries 1– | |

| Name | Expression | Unit | Description | Selection | Details |
|---|---|---|---|---|---|
| | mean(emetric2))),-0.5*dflux_spatial(cOx)/(pi*r),NaN) | | | 4, 6–7 | |
| tds.bndFlux_cOx | if(r>0.001/sqrt(sqrt(mean(emetric2))),0.25*(uflux_spatial(cOx)-dflux_spatial(cOx))/(pi*r),NaN) | mol/(m²·s) | Boundary flux | Boundary 5 | |
| tds.bndFlux_cRed | if(r>0.001/sqrt(sqrt(mean(emetric2))),-0.5*dflux_spatial(cRed)/(pi*r),NaN) | mol/(m²·s) | Boundary flux | Boundaries 1–4, 6–7 | |
| tds.bndFlux_cRed | if(r>0.001/sqrt(sqrt(mean(emetric2))),0.25*(uflux_spatial(cRed)-dflux_spatial(cRed))/(pi*r),NaN) | mol/(m²·s) | Boundary flux | Boundary 5 | |
| tds.Res_cOx | d(cOx,TIME)-d(cOx,r)*d(r,TIME)-d(cOx,z)*d(z,TIME)-tds.R_cOx | mol/(m³·s) | Equation residual | Domains 1–2 | |
| tds.Res_cRed | d(cRed,TIME)-d(cRed,r)*d(r,TIME)-d(cRed,z)*d(z,TIME)-tds.R_cRed | mol/(m³·s) | Equation residual | Domains 1–2 | |

### Shape functions

| Name | Shape function | Unit | Description | Shape frame | Selection |
|---|---|---|---|---|---|
| cOx | Lagrange (Linear) | mol/m³ | Concentration | Spatial | Domains 1–2 |
| cRed | Lagrange (Linear) | mol/m³ | Concentration | Spatial | Domains 1–2 |

### Weak Expressions

| Weak expression | Integration order | Integration frame | Selection |
|---|---|---|---|
| 2*(-(cOxTIME-cOxr*d(r,TIME)-cOxz*d(z,TIME))*test(cOx)+tds.dflux_cOxr*test(cOxr)+tds.dflux_cOxz*test(cOxz))*pi*r | 2 | Spatial | Domains 1–2 |
| 2*(-(cRedTIME-cRedr*d(r,TIME)-cRedz*d(z,TIME))*test(cRed)+tds.dflux_cRedr*test(cRedr)+tds.dflux_cRedz*test(cRedz))*pi*r | 2 | Spatial | Domains 1–2 |

| Weak expression | Integration order | Integration frame | Selection |
|---|---|---|---|
| 2*tds.streamline*(isScalingSystemDomain==0)*pi*r | 2 | Spatial | Domains 1–2 |
| 2*tds.crosswind*(isScalingSystemDomain==0)*pi*r | 4 | Spatial | Domains 1–2 |

### 2.3.4 Axial Symmetry 1

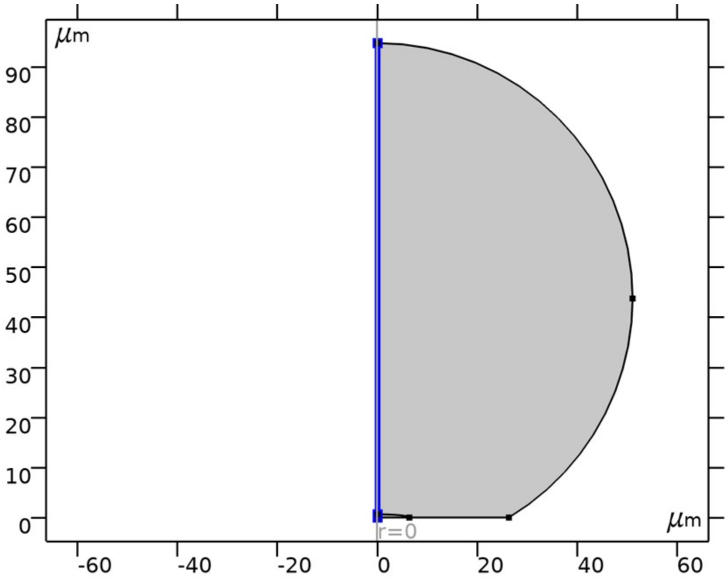


*Axial Symmetry 1*

SELECTION

| | |
|---|---|
| Geometric entity level | Boundary |
| Selection | Geometry geom1: Dimension 1: All boundaries |

### 2.3.5 No Flux 1

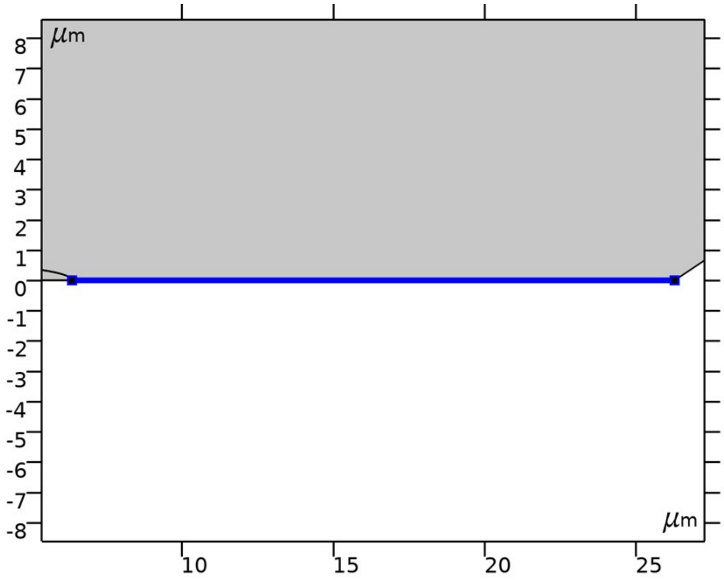


*No Flux 1*

SELECTION

| | |
|---|---|
| Geometric entity level | Boundary |
| Selection | Geometry geom1: Dimension 1: All boundaries |

EQUATIONS

$$-\mathbf{n}\cdot\mathbf{J}_i = 0$$

### 2.3.6 Initial Values in DCE

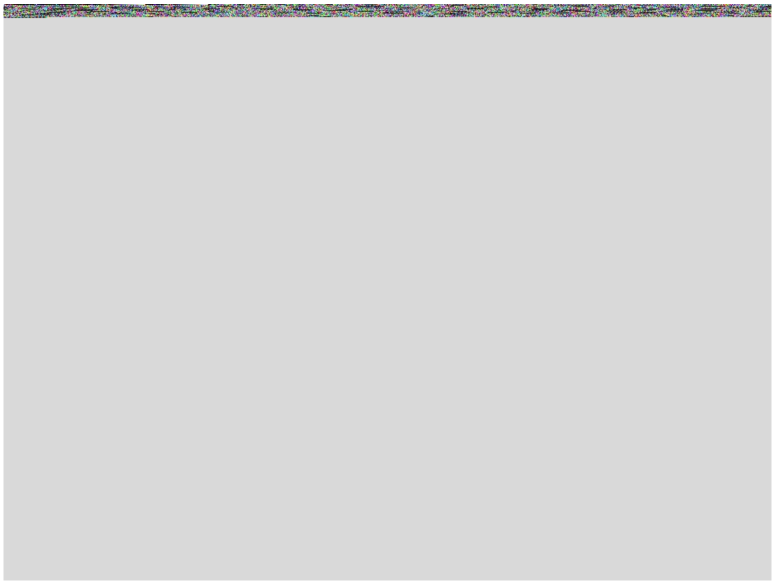

*Initial Values in DCE*

SELECTION

| | |
|---|---|
| Geometric entity level | Domain |
| Selection | Geometry geom1: Dimension 2: All domains |

### Initial Values

SETTINGS

| Description | Value |
|---|---|
| Concentration | {C0_ox_ini, C0_red_ini} |

## 2.3.7 Flux at electrode (E0 & ohm corrections)



*Flux at electrode (E0 & ohm corrections)*

SELECTION

| | |
|---|---|
| Geometric entity level | Boundary |
| Selection | Geometry geom1: Dimension 1: Boundary 2 |

EQUATIONS

$$-\mathbf{n}\cdot\mathbf{J}_i = J_{0,i}$$

### Inward Flux

SETTINGS

| Description | Value |
|---|---|
| Flux type | General inward flux |
| Species cOx | On |
| Species cRed | On |

| Description | Value |
| --- | --- |
| | {( (k0*exp((1 - alp)*f*(pot(t) - E0j - DE)))*cRed -(k0*exp(-alp*f*(pot(t) - E0j - DE)))*cOx), -( (k0*exp((1 - alp)*f*(pot(t) - E0j - DE)))*cRed -(k0*exp(-alp*f*(pot(t) - E0j - DE)))*cOx)} |

### Weak Expressions

| Weak expression | Integration order | Integration frame | Selection |
| --- | --- | --- | --- |
| 2*k0*(exp((1-alp)*f*(pot(t)-E0j-DE))*cRed-exp(-alp*f*(pot(t)-E0j-DE))*cOx)*test(cOx)*pi*r | 2 | Spatial | Boundary 2 |
| -2*k0*(exp((1-alp)*f*(pot(t)-E0j-DE))*cRed-exp(-alp*f*(pot(t)-E0j-DE))*cOx)*test(cRed)*pi*r | 2 | Spatial | Boundary 2 |

## 2.3.8 Flux at o/w boundary

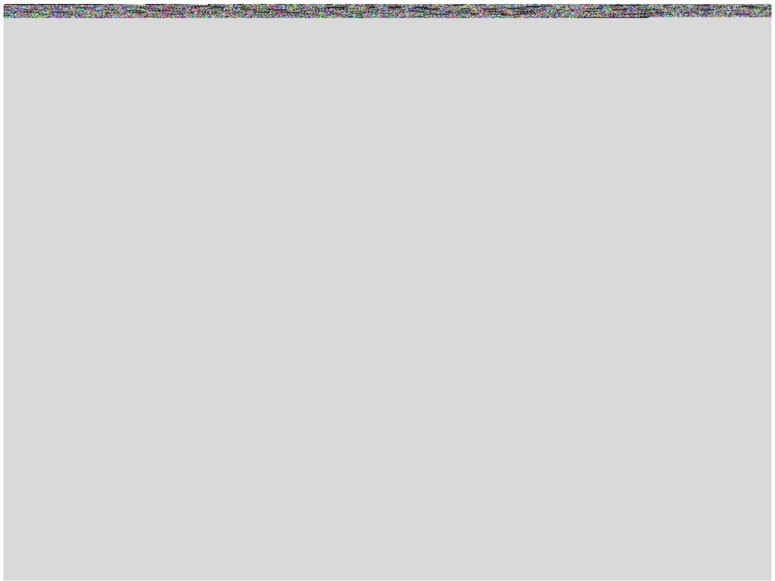

*Flux at o/w boundary*

SELECTION

| | |
| --- | --- |
| Geometric entity level | Boundary |
| Selection | Geometry geom1: Dimension 1: Boundaries 6–7 |

EQUATIONS

$$-\mathbf{n} \cdot \mathbf{J}_i = J_{0,i}$$

### Inward Flux

SETTINGS

| Description | Value |
| --- | --- |
| Flux type | General inward flux |

| Description | Value |
| --- | --- |
| Species cOx | On |
| Species cRed | On |
|  | {loss(t)* (if( r_c > 1.25*r_elec, -Vn(t), sqrt(Vr_CCR^2 + Vz_CCR^2) )*cOx) - kloss_ox*cOx, loss(t)* (if( r_c > 1.25*r_elec, -Vn(t), sqrt(Vr_CCR^2 + Vz_CCR^2) )*cRed) - kloss_red*cRed} |

### Weak Expressions

| Weak expression | Integration order | Integration frame | Selection |
| --- | --- | --- | --- |
| 2*cOx*(loss(t)*if(r_c>1.25*r_elec, - Vn(t),sqrt(Vr_CCR^2+Vz_CCR^2) )-kloss_ox)*test(cOx)*pi*r | 2 | Spatial | Boundaries 6–7 |
| 2*cRed*(loss(t)*if(r_c>1.25*r_ele c,- Vn(t),sqrt(Vr_CCR^2+Vz_CCR^2) )-kloss_red)*test(cRed)*pi*r | 2 | Spatial | Boundaries 6–7 |

## 2.4 MOVING MESH

USED PRODUCTS

| |
| --- |
| COMSOL Multiphysics |

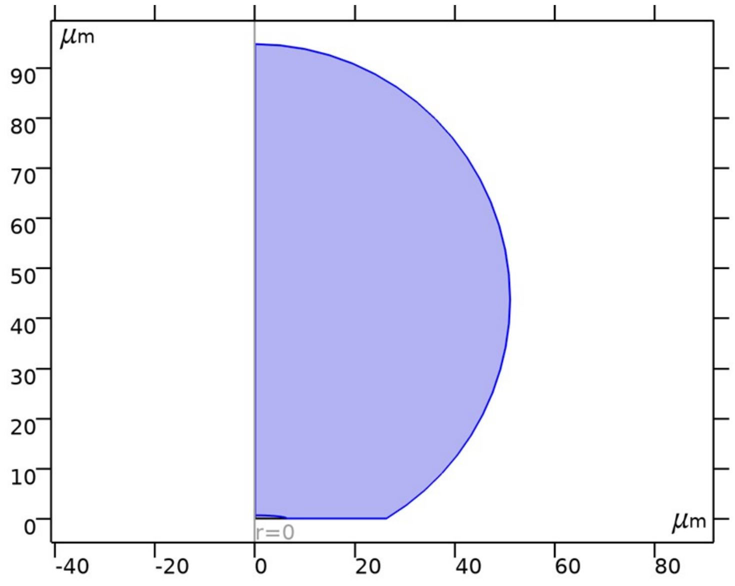


*Moving Mesh*

SELECTION

| | |
| --- | --- |
| Geometric entity level | Domain |
| Selection | Geometry geom1: Dimension 2: Domain 2 |

### 2.4.1 Interface Settings

#### Frame Settings

SETTINGS

| Description | Value |
| --- | --- |
| Geometry shape function | 1 |

#### Legacy Functionality (Development Only)

SETTINGS

| Description | Value |
| --- | --- |
| Use legacy moving frame functionality | Off |

### 2.4.2 Variables

| Name | Expression | Unit | Description | Selection | Details |
| --- | --- | --- | --- | --- | --- |
| ale.nR | nR | | Normal vector, R component | Boundaries 3–7 | Meta |
| ale.nPHI | root.nPHI | | Normal vector, PHI component | Boundaries 3–7 | Meta |
| ale.nZ | nZ | | Normal vector, Z component | Boundaries 3–7 | Meta |
| ale.nRmesh | nRmesh | | Normal vector (mesh), R component | Boundaries 3–7 | Meta |
| ale.nPHImesh | root.nPHImesh | | Normal vector (mesh), PHI component | Boundaries 3–7 | Meta |
| ale.nZmesh | nZmesh | | Normal vector (mesh), Z component | Boundaries 3–7 | Meta |
| ale.nr | nr | | Normal vector, r component | Boundaries 3–7 | Meta |
| ale.nphi | root.nphi | | Normal vector, phi component | Boundaries 3–7 | Meta |
| ale.nz | nz | | Normal vector, z component | Boundaries 3–7 | Meta |
| ale.nrmesh | nrmesh | | Normal vector (mesh), r component | Boundaries 3–7 | Meta |
| ale.nphimesh | root.nphimesh | | Normal vector (mesh), phi | Boundaries 3–7 | Meta |

| Name | Expression | Unit | Description | Selection | Details |
|---|---|---|---|---|---|
| | | | component | | |
| ale.nzmesh | nzmesh | | Normal vector (mesh), z component | Boundaries 3–7 | Meta |
| ale.nRg | nRg | | Normal vector, Rg component | Boundaries 3–7 | Meta |
| ale.nPHIg | root.nPHIg | | Normal vector, PHIg component | Boundaries 3–7 | Meta |
| ale.nZg | nZg | | Normal vector, Zg component | Boundaries 3–7 | Meta |
| ale.nRgmesh | nRgmesh | | Normal vector (mesh), Rg component | Boundaries 3–7 | Meta |
| ale.nPHIgmesh | root.nPHIgmesh | | Normal vector (mesh), PHIg component | Boundaries 3–7 | Meta |
| ale.nZgmesh | nZgmesh | | Normal vector (mesh), Zg component | Boundaries 3–7 | Meta |
| ale.nRm | nRm | | Normal vector, Rm component | Boundaries 3–7 | Meta |
| ale.nPHIm | root.nPHIm | | Normal vector, PHIm component | Boundaries 3–7 | Meta |
| ale.nZm | nZm | | Normal vector, Zm component | Boundaries 3–7 | Meta |
| ale.nRmmesh | nRmmesh | | Normal vector (mesh), Rm component | Boundaries 3–7 | Meta |
| ale.nPHImmesh | root.nPHImmesh | | Normal vector (mesh), PHIm component | Boundaries 3–7 | Meta |
| ale.nZmmesh | nZmmesh | | Normal vector (mesh), Zm component | Boundaries 3–7 | Meta |
| ale.relVol | spatial.relVol | 1 | Local relative element volume | Domain 2 | |
| ale.relVolMin | spatial.relVolMin | 1 | Minimum relative | Global | |

| Name | Expression | Unit | Description | Selection | Details |
|---|---|---|---|---|---|
| | | | element volume | | |
| ale.relVolMax | spatial.relVolMax | 1 | Maximum relative element volume | Global | |
| ale.minqual | spatial.minqual | | Minimum element quality | Global | |
| ale.I1iso | spatial.I1iso | 1 | First invariant of isochoric mesh strain tensor | Domain 2 | |
| ale.I1isoMax | spatial.I1isoMax | 1 | Maximum element distortion | Global | |
| ale.J_mesh | spatial.J_mesh | 1 | Mesh deformation volume ratio | Domain 2 | |
| rt | d(r,TIME) | m/s | Mesh velocity, r component | Global | |
| phit | 0 | m/s | Mesh velocity, phi component | Global | |
| zt | d(z,TIME) | m/s | Mesh velocity, z component | Global | |

### 2.4.3 Fixed Mesh 1

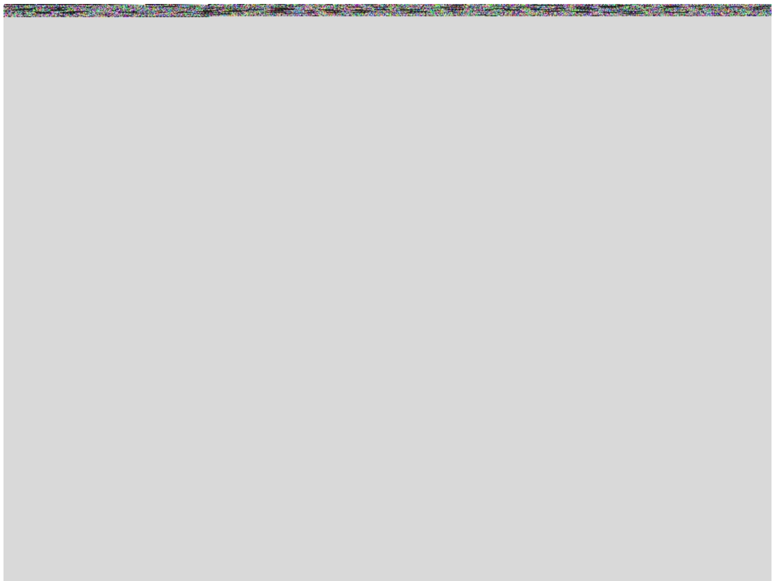

*Fixed Mesh 1*

SELECTION

| | |
|---|---|
| Geometric entity level | Domain |
| Selection | Geometry geom1: Dimension 2: All domains |

### 2.4.4 Prescribed Mesh Displacement 1

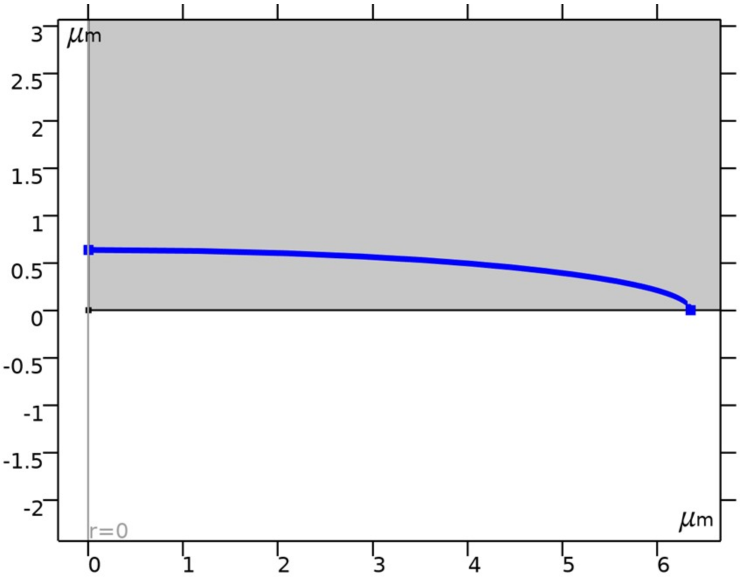


*Prescribed Mesh Displacement 1*

SELECTION

| | |
|---|---|
| Geometric entity level | Boundary |

| | |
|---|---|
| Selection | Geometry geom1: Dimension 1: All boundaries |

SETTINGS

| Description | Value |
|---|---|
| Prescribed mesh displacement | {0, 0} |
| Use weak constraints | Off |
| Prescribed # displacement | {On, On} |

#### Coordinate System Selection

SETTINGS

| Description | Value |
|---|---|
| Coordinate system | Global coordinate system |

### 2.4.5 Free Deformation 1

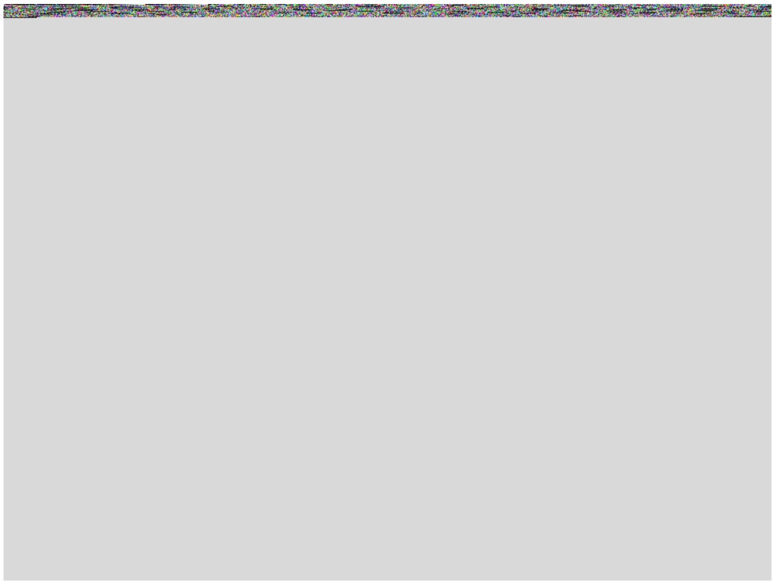

*Free Deformation 1*

SELECTION

| | |
|---|---|
| Geometric entity level | Domain |
| Selection | Geometry geom1: Dimension 2: Domain 2 |

SETTINGS

| Description | Value |
|---|---|
| Initial mesh displacement | {0, 0} |

### 2.4.6 Zero Normal Mesh Displacement 1

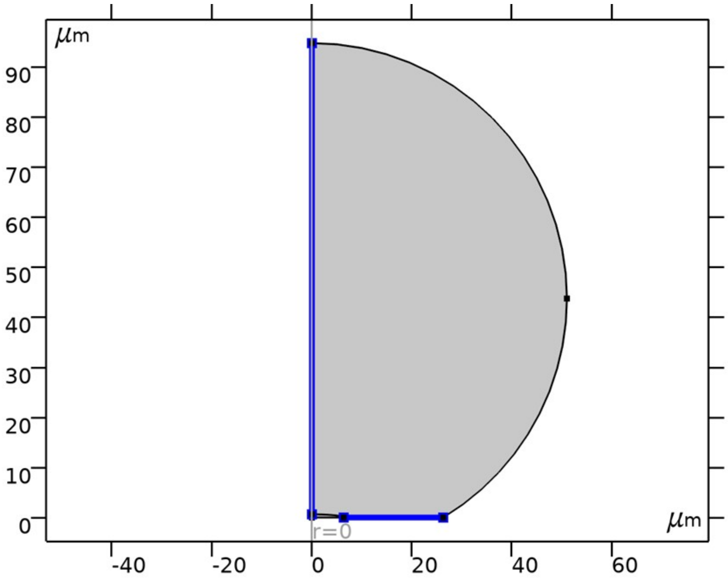


*Zero Normal Mesh Displacement 1*

SELECTION

| | |
|---|---|
| Geometric entity level | Boundary |
| Selection | Geometry geom1: Dimension 1: Boundaries 3–4 |

EQUATIONS

$$(x - X) \cdot N = 0$$

SETTINGS

| Description | Value |
|---|---|
| Use weak constraints | Off |

### 2.4.7 Prescribed Mesh Velocity 1

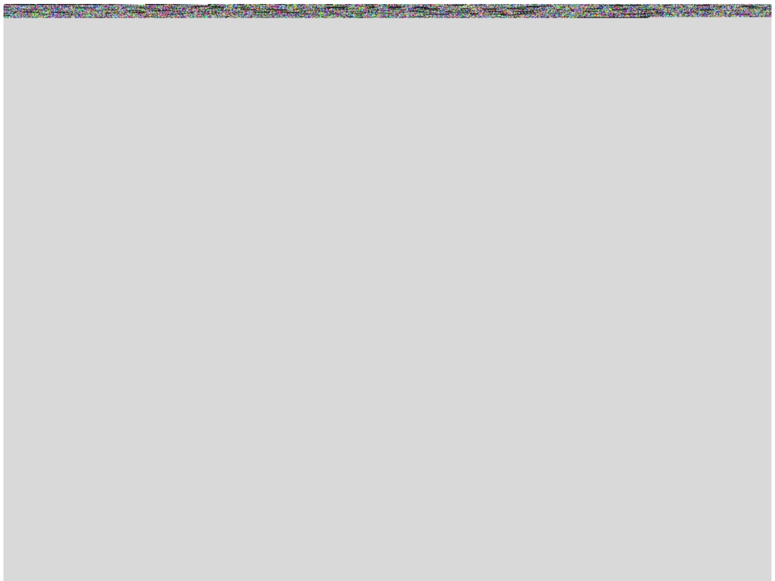

*Prescribed Mesh Velocity 1*

SELECTION

| | |
|---|---|
| Geometric entity level | Boundary |
| Selection | Geometry geom1: Dimension 1: Boundaries 6–7 |

SETTINGS

| **Description** | **Value** |
|---|---|
| Prescribed mesh velocity | {if( r_c > 1.25*r_elec,    Vr_CCA,    Vr_CCR  ), if( r_c > 1.25*r_elec,    Vz_CCA,    Vz_CCR  )} |
| Use weak constraints | Off |
| Prescribed # velocity | {On, On} |

#### Coordinate System Selection

SETTINGS

| **Description** | **Value** |
|---|---|
| Coordinate system | Global coordinate system |

## 2.5 MESHES

### 2.5.1 Mesh 1

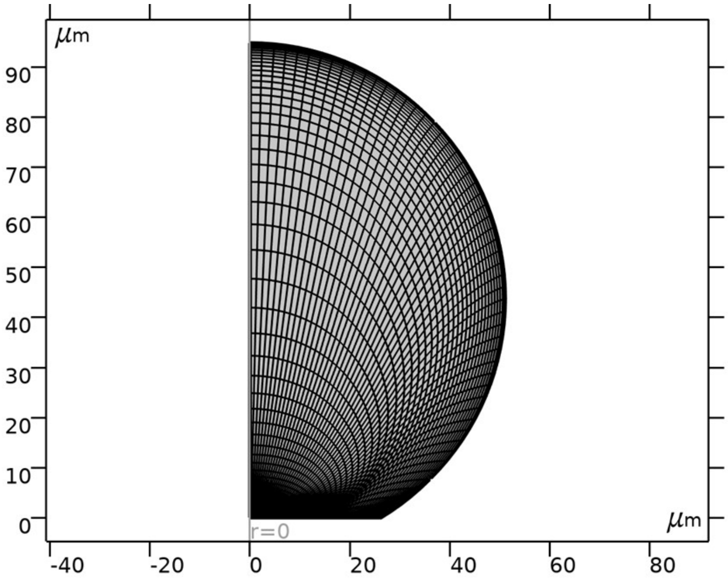


*Mesh 1*

#### MESH STATISTICS

| Description | Value |
|---|---|
| Minimum element quality | 0.01292 |
| Average element quality | 0.423 |
| Triangle | 534 |
| Quad | 2700 |
| Edge element | 266 |
| Vertex element | 6 |

**Size (size)**

#### SETTINGS

| Description | Value |
|---|---|
| Maximum element size | 6.35 |
| Minimum element size | 0.0284 |
| Curvature factor | 0.3 |
| Maximum element growth rate | 1.3 |

**electrode (edg1)**

#### SELECTION

| | |
|---|---|
| Geometric entity level | Boundary |

| | |
|---|---|
| Selection | Geometry geom1: Dimension 1: Boundaries 1–2, 5 |

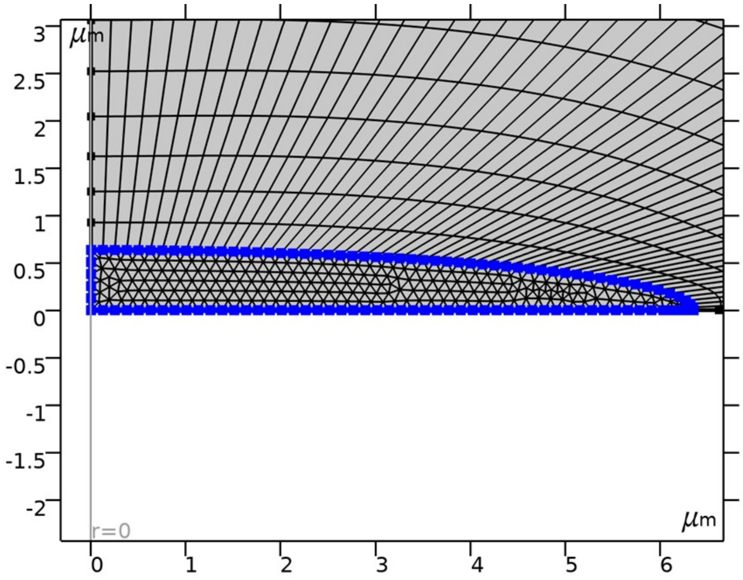


*electrode*

### SETTINGS

| Description | Value |
|---|---|
| Number of iterations | 4 |
| Maximum element depth to process | 4 |

**Size 1 (size1)**

### SELECTION

| | |
|---|---|
| Geometric entity level | Boundary |
| Selection | Geometry geom1: Dimension 1: Boundaries 1–2, 4–5 |

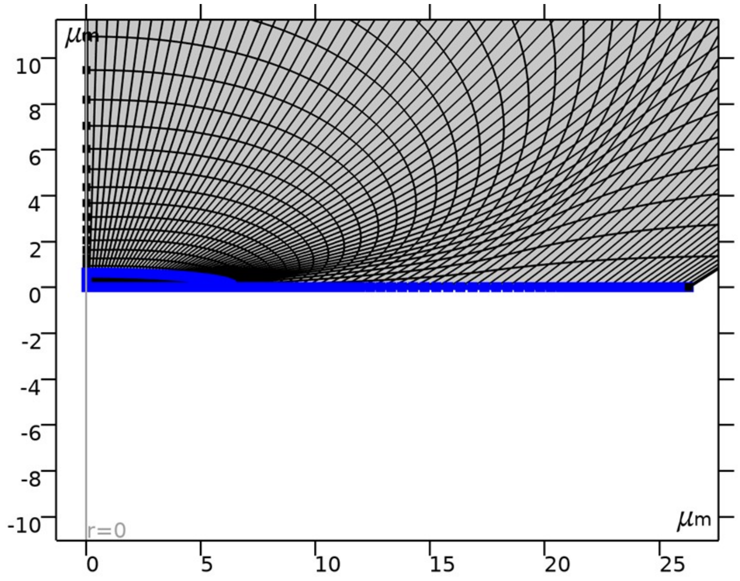


*Size 1*

SETTINGS

| Description | Value |
|---|---|
| Maximum element size | r_elec/50 |
| Minimum element size | 2.27E-8 |
| Minimum element size | Off |
| Curvature factor | 0.3 |
| Curvature factor | Off |
| Resolution of narrow regions | Off |
| Maximum element growth rate | 1.3 |
| Maximum element growth rate | Off |
| Custom element size | Custom |

### diffusion layer (ftri1)

SELECTION

| | |
|---|---|
| Geometric entity level | Domain |
| Selection | Geometry geom1: Dimension 2: Domain 1 |

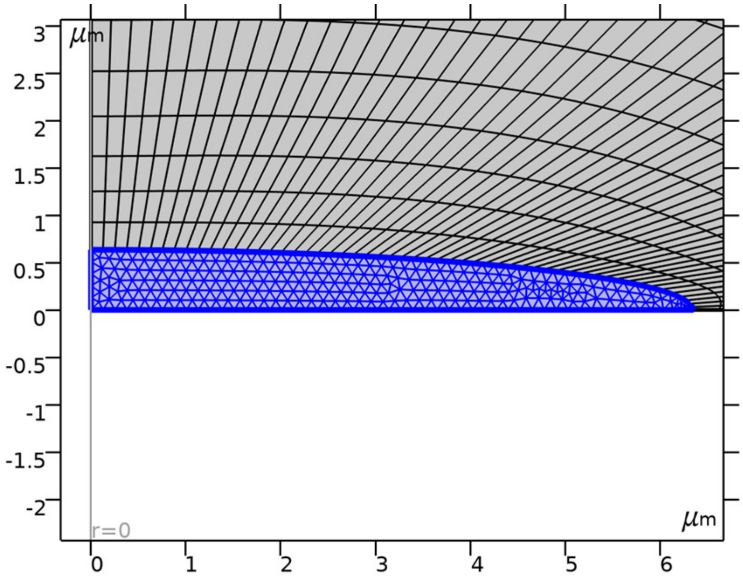


*diffusion layer*

SETTINGS

| Description | Value |
|---|---|
| Number of iterations | 4 |
| Maximum element depth to process | 4 |

**Size 1 (size1)**

SELECTION

| | |
|---|---|
| Geometric entity level | Domain |
| Selection | Geometry geom1: Dimension 2: Domain 1 |

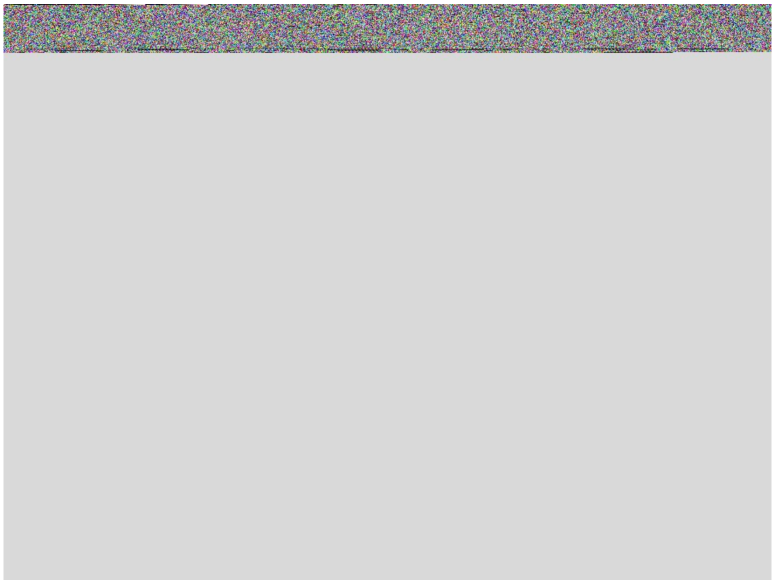

*Size 1*

SETTINGS

| Description | Value |
|---|---|
| Calibrate for | Semiconductor |
| Maximum element size | 0.189 |
| Minimum element size | 0.0379 |
| Curvature factor | 0.25 |
| Predefined size | Finer |

**revolution axis (edg2)**

SELECTION

| | |
|---|---|
| Geometric entity level | Boundary |
| Selection | Geometry geom1: Dimension 1: Boundary 3 |

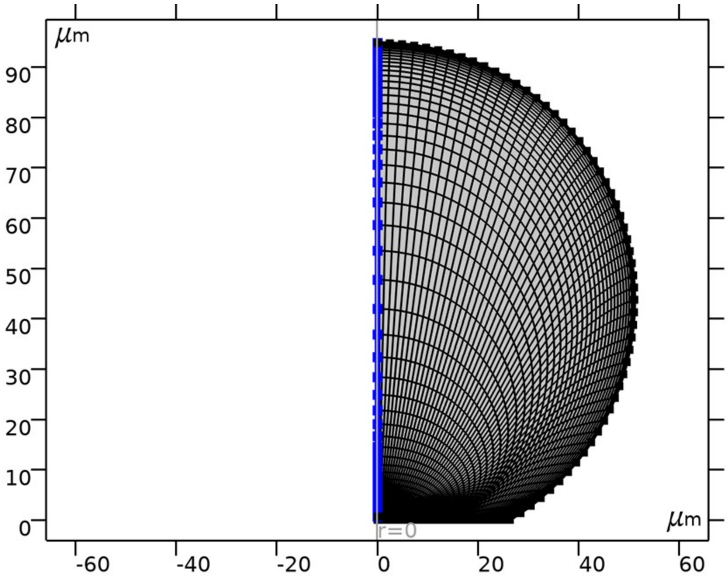


*revolution axis*

SETTINGS

| Description | Value |
|---|---|
| Number of iterations | 4 |
| Maximum element depth to process | 4 |

**Distribution 1 (dis1)**

SELECTION

| | |
|---|---|
| Geometric entity level | Boundary |
| Selection | Geometry geom1: Dimension 1: Boundary 3 |

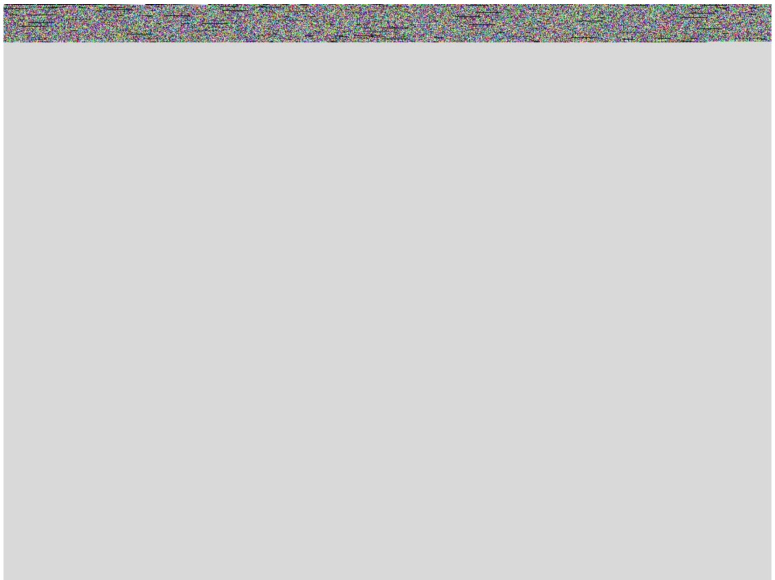

*Distribution 1*

### SETTINGS

| Description | Value |
| --- | --- |
| Distribution type | Predefined |
| Number of elements | 50 |
| Element ratio | 20 |
| Growth formula | Geometric sequence |
| Symmetric distribution | On |

## glass sheath (edg3)

### SELECTION

| | |
| --- | --- |
| Geometric entity level | Boundary |
| Selection | Geometry geom1: Dimension 1: Boundary 4 |

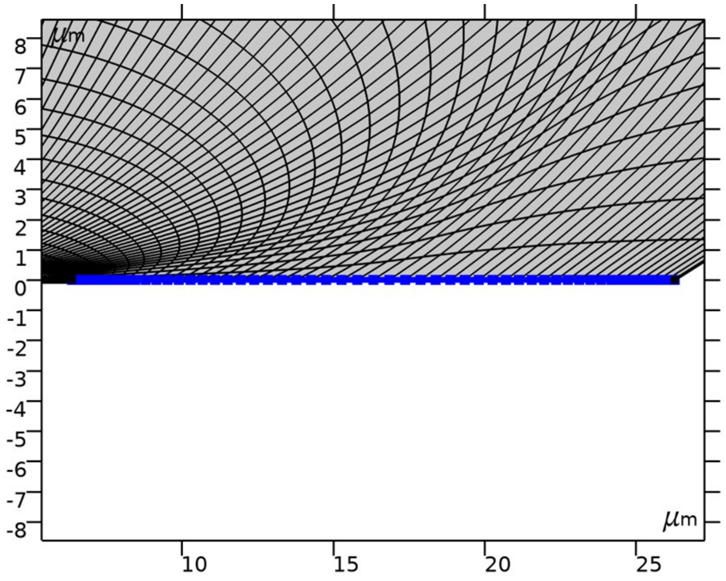


*glass sheath*

SETTINGS

| Description | Value |
|---|---|
| Number of iterations | 4 |
| Maximum element depth to process | 4 |

**Distribution 1 (dis1)**

SELECTION

| | |
|---|---|
| Geometric entity level | Boundary |
| Selection | Geometry geom1: Dimension 1: Boundary 4 |

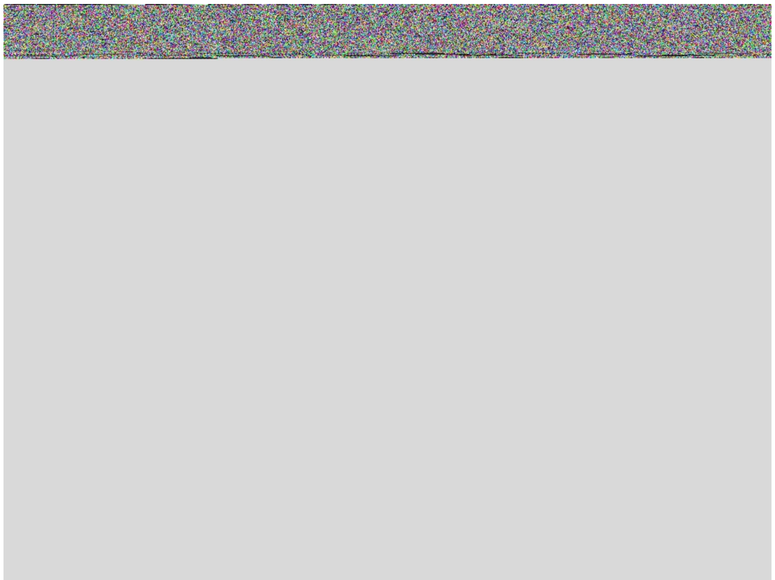

*Distribution 1*

SETTINGS

| Description | Value |
|---|---|
| Distribution type | Predefined |
| Number of elements | 50 |
| Element ratio | 2 |
| Symmetric distribution | On |

### Mapped 1 (map1)

SELECTION

| | |
|---|---|
| Geometric entity level | Domain |
| Selection | Geometry geom1: Dimension 2: Domain 2 |

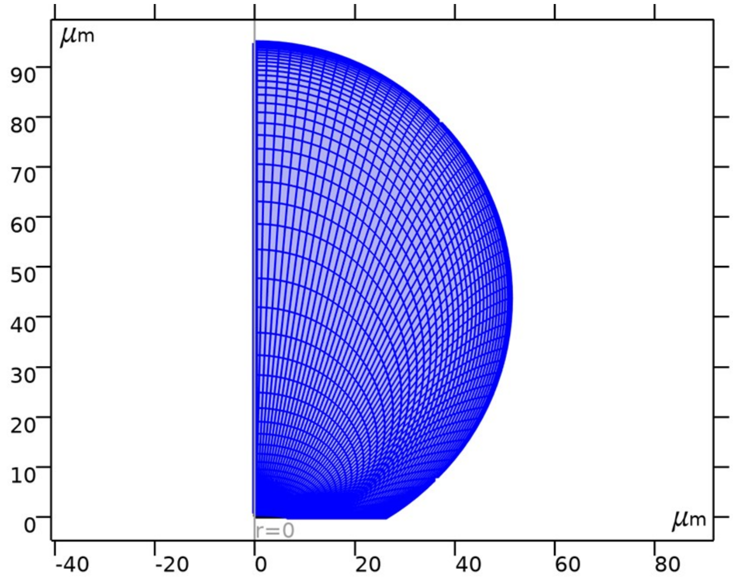


*Mapped 1*

SETTINGS

| Description | Value |
|---|---|
| Number of iterations | 4 |
| Maximum element depth to process | 4 |

### 2.5.2 Problematic Deformed Mesh 1

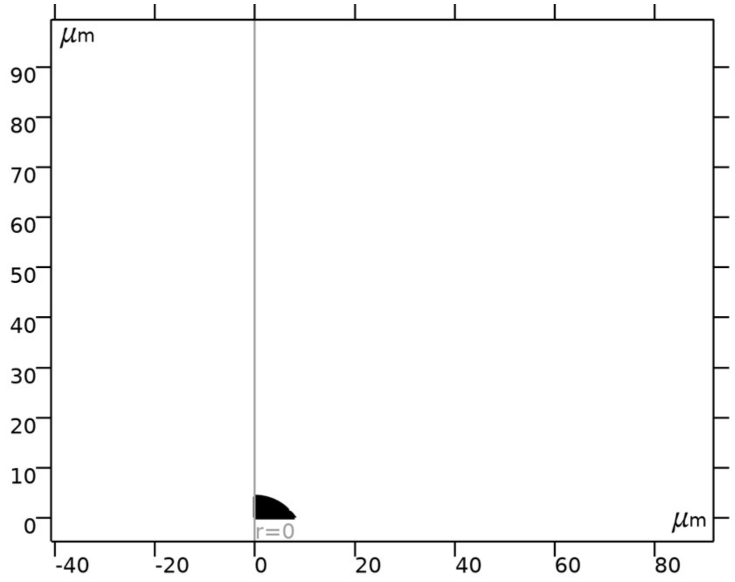


*Problematic Deformed Mesh 1*

MESH STATISTICS

| Description | Value |
|---|---|

| Description | Value |
| --- | --- |
| Minimum element quality | -0.08319 |
| Average element quality | 0.5246 |
| Triangle | 534 |
| Quad | 2700 |
| Edge element | 266 |
| Vertex element | 6 |

# 3 Study 1 (CV 1 to 39)

### COMPUTATION INFORMATION

| | |
|---|---|
| Computation time | 23 min 33 s |

## 3.1 TIME DEPENDENT

| Times | Unit |
|---|---|
| range(0*t_cv,t_tot/nb/200,39*t_cv) | s |

### STUDY SETTINGS

| Description | Value |
|---|---|
| Include geometric nonlinearity | Off |

### STUDY SETTINGS

### MESH

| Feature | Value |
|---|---|
| Geometry 1 | mesh1 |

### PHYSICS AND VARIABLES SELECTION

| Physics interface | Discretization |
|---|---|
| Transport of Diluted Species in droplet (tds) | physics |
| Moving Mesh (ale) | physics |

### MESH SELECTION

| Geometry | Mesh |
|---|---|
| Geometry 1 (geom1) | mesh1 |

## 3.2 SOLVER CONFIGURATIONS

### 3.2.1 Solution 1

**Compile Equations: Time Dependent (st1)**

### STUDY AND STEP

| Description | Value |
|---|---|
| Use study | Study 1 (CV 1 to 39) |
| Use study step | Time Dependent |

### LOG

```
<---- Compile Equations: Time Dependent in Study 1  (CV  1 to 39)/Solution 1
      (sol2) -----------------------------------------------------------------
Started at Jun 15, 2023 6:33:18 PM.
Geometry shape function: Linear Lagrange
Running on Intel64 Family 6 Model 158 Stepping 10, GenuineIntel.
Using 1 socket with 6 cores in total on LAPTOP-2492RD8I.
Available memory: 32.27 GB.
Time: 1 s.
Physical memory: 2.5 GB
Virtual memory: 2.61 GB
Ended at Jun 15, 2023 6:33:19 PM.
----- Compile Equations: Time Dependent in Study 1  (CV  1 to 39)/Solution 1
      (sol2) ---------------------------------------------------------------->
```

### Dependent Variables 1 (v1)

#### GENERAL

| Description | Value |
|---|---|
| Defined by study step | Time Dependent |

#### RESIDUAL SCALING

| Description | Value |
|---|---|
| Method | Manual |

#### INITIAL VALUE CALCULATION CONSTANTS

| Constant name | Initial value source |
|---|---|
| t | range(0*t_cv,t_tot/nb/200,39*t_cv) |
| timestep | 0.21450000000000002[s] |

#### LOG

```
<---- Dependent Variables 1 in Study 1  (CV  1 to 39)/Solution 1 (sol2) --------
Started at Jun 15, 2023 6:33:19 PM.
Solution time: 0 s.
Physical memory: 2.5 GB
Virtual memory: 2.61 GB
Ended at Jun 15, 2023 6:33:19 PM.
----- Dependent Variables 1 in Study 1  (CV  1 to 39)/Solution 1 (sol2) ------->
```

### Concentration (comp1.cOx) (comp1_cOx)

#### GENERAL

| Description | Value |
|---|---|
| Field components | comp1.cOx |
| Internal variables | {comp1.uflux.cOx, comp1.dflux.cOx, comp1.tds.dt2Inv_cOx} |

### Concentration (comp1.cRed) (comp1_cRed)

#### GENERAL

| Description | Value |
|---|---|

| Description | Value |
| --- | --- |
| Field components | comp1.cRed |
| Internal variables | {comp1.uflux.cRed, comp1.dflux.cRed, comp1.tds.dt2Inv_cRed} |

#### Spatial mesh displacement (comp1.spatial.disp) (comp1_spatial_disp)

##### GENERAL

| Description | Value |
| --- | --- |
| Field components | {comp1.spatial.u, comp1.spatial.w} |

##### SCALING

| Description | Value |
| --- | --- |
| Method | Manual |
| Scale | 2.4340912061794226E-7 |

#### comp1.RgZg (comp1_RgZg)

##### GENERAL

| Description | Value |
| --- | --- |
| Field components | {Rg, Zg} |
| Solve for this field | Off |

### Time-Dependent Solver 1 (t1)

##### GENERAL

| Description | Value |
| --- | --- |
| Defined by study step | Time Dependent |
| Output times | {0, 0.027500000000000004, 0.05500000000000001, 0.08250000000000002, 0.11000000000000001, 0.1375, 0.16500000000000004, 0.19250000000000003, 0.22000000000000003, 0.24750000000000003, 0.275, 0.30250000000000005, 0.33000000000000007, 0.35750000000000004, 0.38500000000000006, 0.41250000000000003, 0.44000000000000006, 0.4675000000000001, 0.49500000000000005, 0.5225000000000001, 0.55, 0.5775000000000001, 0.6050000000000001, 0.6325000000000001, 0.6600000000000001, 0.6875000000000001, 0.7150000000000001, 0.7425, 0.7700000000000001, 0.7975000000000001, 0.8250000000000001, 0.8525000000000001, 0.8800000000000001, 0.9075000000000001, 0.9350000000000002, 0.9625000000000001, 0.9900000000000001, 1.0175, 1.0450000000000002, 1.0725000000000002, 1.1, 1.1275000000000002, 1.1550000000000002, 1.1825, 1.2100000000000002, 1.2375000000000003, 1.2650000000000001, 1.2925000000000002, 1.3200000000000003, 1.3475000000000001, 1.3750000000000002, 1.4025, 1.4300000000000002, 1.4575000000000002, 1.485, 1.5125000000000002, 1.5400000000000003, 1.5675000000000001, 1.5950000000000002, 1.6225000000000003, 1.6500000000000001, 1.6775000000000002, 1.7050000000000003, 1.7325000000000002, |

| Description | Value |
| --- | --- |
| | 1.7600000000000002, 1.7875000000000003, 1.8150000000000002, 1.8425000000000002, 1.8700000000000003, 1.8975000000000002, 1.9250000000000003, 1.9525000000000003, 1.9800000000000002, 2.0075000000000003, 2.035, 2.0625000000000004, 2.0900000000000003, 2.1175, 2.1450000000000005, 2.1725000000000003, 2.2, 2.2275000000000005, 2.2550000000000003, 2.2825, 2.3100000000000005, 2.3375000000000004, 2.365, 2.3925000000000005, 2.4200000000000004, 2.4475000000000002, 2.4750000000000005, 2.5025000000000004, 2.5300000000000002, 2.5575000000000006, 2.5850000000000004, 2.6125000000000003, 2.6400000000000006, 2.6675000000000004, 2.6950000000000003, 2.7225, 2.7500000000000004, 2.7775000000000003, 2.805, 2.8325000000000005, 2.8600000000000003, 2.8875, 2.9150000000000005, 2.9425000000000003, 2.97, 2.9975000000000005, 3.0250000000000004, 3.0525, 3.0800000000000005, 3.1075000000000004, 3.1350000000000002, 3.1625000000000005, 3.1900000000000004, 3.2175000000000002, 3.2450000000000006, 3.2725000000000004, 3.3000000000000003, 3.3275000000000006, 3.3550000000000004, 3.3825000000000003, 3.4100000000000006, 3.4375000000000004, 3.4650000000000003, 3.4925000000000006, 3.5200000000000005, 3.5475000000000003, 3.5750000000000006, 3.6025000000000005, 3.6300000000000003, 3.6575000000000006, 3.6850000000000005, 3.7125000000000004, 3.7400000000000007, 3.7675000000000005, 3.7950000000000004, 3.8225000000000007, 3.8500000000000005, 3.8775000000000004, 3.9050000000000007, 3.9325000000000006, 3.9600000000000004, 3.9875000000000007, 4.015000000000001, 4.0425, 4.07, 4.0975, 4.125000000000001, 4.152500000000001, 4.180000000000001, 4.2075000000000005, 4.235, 4.2625, 4.290000000000001, 4.317500000000001, 4.345000000000001, 4.3725000000000005, 4.4, 4.4275, 4.455000000000001, 4.482500000000001, 4.510000000000001, 4.5375000000000005, 4.565, 4.5925, 4.620000000000001, 4.647500000000001, 4.675000000000001, 4.702500000000001, 4.73, 4.7575, 4.785000000000001, 4.812500000000001, 4.840000000000001, 4.867500000000001, 4.8950000000000005, 4.9225, 4.950000000000001, 4.977500000000001, 5.005000000000001, 5.032500000000001, 5.0600000000000005, 5.0875, 5.115000000000001, 5.142500000000001, 5.170000000000001, 5.197500000000001, 5.2250000000000005, 5.2525, 5.280000000000001, 5.307500000000001, 5.335000000000001, 5.362500000000001, 5.390000000000001, 5.4175, 5.445, 5.472500000000001, 5.500000000000001, 5.527500000000001, 5.555000000000001, 5.5825000000000005, 5.61, 5.637500000000001, 5.665000000000001, 5.692500000000001, 5.720000000000001, 5.7475000000000005, 5.775, 5.802500000000001, 5.830000000000001, 5.857500000000001, 5.885000000000001, 5.9125000000000005, 5.94, 5.967500000000001, 5.995000000000001, 6.022500000000001, 6.050000000000001, 6.077500000000001, 6.105, 6.132500000000001, 6.160000000000001, 6.187500000000001, 6.215000000000001, 6.242500000000001, 6.2700000000000005, 6.297500000000001, 6.325000000000001, |

| Description | Value |
| --- | --- |
| | 6.352500000000001, 6.380000000000001, 6.407500000000001, 6.4350000000000005, 6.462500000000001, 6.490000000000001, 6.517500000000001, 6.545000000000001, 6.572500000000001, 6.6000000000000005, 6.627500000000001, 6.655000000000001, 6.682500000000001, 6.710000000000001, 6.737500000000001, 6.765000000000001, 6.792500000000001, 6.820000000000001, 6.847500000000001, 6.875000000000001, 6.902500000000001, 6.930000000000001, 6.957500000000001, 6.985000000000001, 7.012500000000001, 7.040000000000001, 7.067500000000001, 7.095000000000001, 7.1225000000000005, 7.150000000000001, 7.177500000000001, 7.205000000000001, 7.232500000000001, 7.260000000000001, 7.2875000000000005, 7.315000000000001, 7.342500000000001, 7.370000000000001, 7.397500000000001, 7.425000000000001, 7.452500000000001, 7.480000000000001, 7.507500000000001, 7.535000000000001, 7.562500000000001, 7.590000000000001, 7.617500000000001, 7.645000000000001, 7.672500000000001, 7.700000000000001, 7.727500000000001, 7.755000000000001, 7.782500000000001, 7.810000000000001, 7.837500000000001, 7.865000000000001, 7.892500000000001, 7.920000000000001, 7.947500000000001, 7.975000000000001, 8.002500000000001, 8.030000000000001, 8.057500000000001, 8.085, 8.1125, 8.14, 8.1675, 8.195, 8.222500000000002, 8.250000000000002, 8.277500000000002, 8.305000000000001, 8.332500000000001, 8.360000000000001, 8.387500000000001, 8.415000000000001, 8.4425, 8.47, 8.4975, 8.525, 8.552500000000002, 8.580000000000002, 8.607500000000002, 8.635000000000002, 8.662500000000001, 8.690000000000001, 8.717500000000001, 8.745000000000001, 8.7725, 8.8, 8.8275, 8.855, 8.8825, 8.910000000000002, 8.937500000000002, 8.965000000000002, 8.992500000000001, 9.020000000000001, 9.047500000000001, 9.075000000000001, 9.102500000000001, 9.13, 9.1575, 9.185, 9.2125, 9.240000000000002, 9.267500000000002, 9.295000000000002, 9.322500000000002, 9.350000000000001, 9.377500000000001, 9.405000000000001, 9.432500000000001, 9.46, 9.4875, 9.515, 9.5425, 9.570000000000002, 9.597500000000002, 9.625000000000002, 9.652500000000002, 9.680000000000001, 9.707500000000001, 9.735000000000001, 9.762500000000001, 9.790000000000001, 9.8175, 9.845, 9.8725, 9.900000000000002, 9.927500000000002, 9.955000000000002, 9.982500000000002, 10.010000000000002, 10.037500000000001, 10.065000000000001, 10.092500000000001, 10.120000000000001, 10.1475, 10.175, 10.2025, 10.230000000000002, 10.257500000000002, 10.285000000000002, 10.312500000000002, 10.340000000000002, 10.367500000000001, 10.395000000000001, 10.422500000000001, 10.450000000000001, 10.477500000000001, 10.505, 10.5325, 10.560000000000002, 10.587500000000002, 10.615000000000002, 10.642500000000002, 10.670000000000002, 10.697500000000002, 10.725000000000001, 10.752500000000001, 10.780000000000001, 10.807500000000001, 10.835, 10.8625, 10.89, 10.917500000000002, 10.945000000000002, |

| Description | Value |
| --- | --- |
| | 10.972500000000002, 11.000000000000002, 11.027500000000002, 11.055000000000001, 11.082500000000001, 11.110000000000001, 11.137500000000001, 11.165000000000001, 11.1925, 11.22, 11.247500000000002, 11.275000000000002, 11.302500000000002, 11.330000000000002, 11.357500000000002, 11.385000000000002, 11.412500000000001, 11.440000000000001, 11.467500000000001, 11.495000000000001, 11.5225, 11.55, 11.577500000000002, 11.605000000000002, 11.632500000000002, 11.660000000000002, 11.687500000000002, 11.715000000000002, 11.742500000000001, 11.770000000000001, 11.797500000000001, 11.825000000000001, 11.852500000000001, 11.88, 11.907500000000002, 11.935000000000002, 11.962500000000002, 11.990000000000002, 12.017500000000002, 12.045000000000002, 12.072500000000002, 12.100000000000001, 12.127500000000001, 12.155000000000001, 12.182500000000001, 12.21, 12.237500000000002, 12.265000000000002, 12.292500000000002, 12.320000000000002, 12.347500000000002, 12.375000000000002, 12.402500000000002, 12.430000000000001, 12.457500000000001, 12.485000000000001, 12.512500000000001, 12.540000000000001, 12.5675, 12.595000000000002, 12.622500000000002, 12.650000000000002, 12.677500000000002, 12.705000000000002, 12.732500000000002, 12.760000000000002, 12.787500000000001, 12.815000000000001, 12.842500000000001, 12.870000000000001, 12.8975, 12.925000000000002, 12.952500000000002, 12.980000000000002, 13.007500000000002, 13.035000000000002, 13.062500000000002, 13.090000000000002, 13.117500000000001, 13.145000000000001, 13.172500000000001, 13.200000000000001, 13.227500000000001, 13.255000000000003, 13.282500000000002, 13.310000000000002, 13.337500000000002, 13.365000000000002, 13.392500000000002, 13.420000000000002, 13.447500000000002, 13.475000000000001, 13.502500000000001, 13.530000000000001, 13.557500000000001, 13.585000000000003, 13.612500000000002, 13.640000000000002, 13.667500000000002, 13.695000000000002, 13.722500000000002, 13.750000000000002, 13.777500000000002, 13.805000000000001, 13.832500000000001, 13.860000000000001, 13.887500000000001, 13.915000000000003, 13.942500000000003, 13.970000000000002, 13.997500000000002, 14.025000000000002, 14.052500000000002, 14.080000000000002, 14.107500000000002, 14.135000000000002, 14.162500000000001, 14.190000000000001, 14.217500000000001, 14.245000000000001, 14.272500000000003, 14.300000000000002, 14.327500000000002, 14.355000000000002, 14.382500000000002, 14.410000000000002, 14.437500000000002, 14.465000000000002, 14.492500000000001, 14.520000000000001, 14.547500000000001, 14.575000000000001, 14.602500000000003, 14.630000000000003, 14.657500000000002, 14.685000000000002, 14.712500000000002, 14.740000000000002, 14.767500000000002, 14.795000000000002, 14.822500000000002, 14.850000000000001, 14.877500000000001, 14.905000000000001, 14.932500000000003, 14.960000000000003, 14.987500000000002, 15.015000000000002, |

| Description | Value |
| --- | --- |
| | 15.042500000000002, 15.070000000000002, 15.097500000000002, 15.125000000000002, 15.152500000000002, 15.180000000000001, 15.207500000000001, 15.235000000000001, 15.262500000000003, 15.290000000000003, 15.317500000000003, 15.345000000000002, 15.372500000000002, 15.400000000000002, 15.427500000000002, 15.455000000000002, 15.482500000000002, 15.510000000000002, 15.537500000000001, 15.565000000000001, 15.592500000000003, 15.620000000000003, 15.647500000000003, 15.675000000000002, 15.702500000000002, 15.730000000000002, 15.757500000000002, 15.785000000000002, 15.812500000000002, 15.840000000000002, 15.867500000000001, 15.895000000000001, 15.922500000000001, 15.950000000000003, 15.977500000000003, 16.005000000000003, 16.032500000000002, 16.060000000000002, 16.087500000000002, 16.115000000000002, 16.142500000000002, 16.17, 16.1975, 16.225, 16.2525, 16.28, 16.3075, 16.335, 16.3625, 16.39, 16.4175, 16.445000000000004, 16.472500000000004, 16.500000000000004, 16.527500000000003, 16.555000000000003, 16.582500000000003, 16.610000000000003, 16.637500000000003, 16.665000000000003, 16.692500000000003, 16.720000000000002, 16.747500000000002, 16.775000000000002, 16.802500000000002, 16.830000000000002, 16.8575, 16.885, 16.9125, 16.94, 16.9675, 16.995, 17.0225, 17.05, 17.0775, 17.105000000000004, 17.132500000000004, 17.160000000000004, 17.187500000000004, 17.215000000000003, 17.242500000000003, 17.270000000000003, 17.297500000000003, 17.325000000000003, 17.352500000000003, 17.380000000000003, 17.407500000000002, 17.435000000000002, 17.462500000000002, 17.490000000000002, 17.517500000000002, 17.545, 17.5725, 17.6, 17.6275, 17.655, 17.6825, 17.71, 17.7375, 17.765, 17.792500000000004, 17.820000000000004, 17.847500000000004, 17.875000000000004, 17.902500000000003, 17.930000000000003, 17.957500000000003, 17.985000000000003, 18.012500000000003, 18.040000000000003, 18.067500000000003, 18.095000000000002, 18.122500000000002, 18.150000000000002, 18.177500000000002, 18.205000000000002, 18.2325, 18.26, 18.2875, 18.315, 18.3425, 18.37, 18.3975, 18.425, 18.452500000000004, 18.480000000000004, 18.507500000000004, 18.535000000000004, 18.562500000000004, 18.590000000000003, 18.617500000000003, 18.645000000000003, 18.672500000000003, 18.700000000000003, 18.727500000000003, 18.755000000000003, 18.782500000000002, 18.810000000000002, 18.837500000000002, 18.865000000000002, 18.892500000000002, 18.92, 18.9475, 18.975, 19.0025, 19.03, 19.0575, 19.085, 19.112500000000004, 19.140000000000004, 19.167500000000004, 19.195000000000004, 19.222500000000004, 19.250000000000004, 19.277500000000003, 19.305000000000003, 19.332500000000003, 19.360000000000003, 19.387500000000003, 19.415000000000003, 19.442500000000003, 19.470000000000002, 19.497500000000002, 19.525000000000002, 19.552500000000002, 19.580000000000002, 19.6075, 19.635, 19.6625, 19.69, 19.7175, 19.745, 19.7725, 19.800000000000004, 19.827500000000004, 19.855000000000004, |

| Description | Value |
| --- | --- |
| | 19.882500000000004, 19.910000000000004, 19.937500000000004, 19.965000000000003, 19.992500000000003, 20.020000000000003, 20.047500000000003, 20.075000000000003, 20.102500000000003, 20.130000000000003, 20.157500000000002, 20.185000000000002, 20.212500000000002, 20.240000000000002, 20.267500000000002, 20.295, 20.3225, 20.35, 20.3775, 20.405, 20.4325, 20.460000000000004, 20.487500000000004, 20.515000000000004, 20.542500000000004, 20.570000000000004, 20.597500000000004, 20.625000000000004, 20.652500000000003, 20.680000000000003, 20.707500000000003, 20.735000000000003, 20.762500000000003, 20.790000000000003, 20.817500000000003, 20.845000000000002, 20.872500000000002, 20.900000000000002, 20.927500000000002, 20.955000000000002, 20.9825, 21.01, 21.0375, 21.065, 21.0925, 21.120000000000005, 21.147500000000004, 21.175000000000004, 21.202500000000004, 21.230000000000004, 21.257500000000004, 21.285000000000004, 21.312500000000004, 21.340000000000003, 21.367500000000003, 21.395000000000003, 21.422500000000003, 21.450000000000003, 21.477500000000003, 21.505000000000003, 21.532500000000002, 21.560000000000002, 21.587500000000002, 21.615000000000002, 21.642500000000002, 21.67, 21.6975, 21.725, 21.7525, 21.78, 21.807500000000005, 21.835000000000004, 21.862500000000004, 21.890000000000004, 21.917500000000004, 21.945000000000004, 21.972500000000004, 22.000000000000004, 22.027500000000003, 22.055000000000003, 22.082500000000003, 22.110000000000003, 22.137500000000003, 22.165000000000003, 22.192500000000003, 22.220000000000002, 22.247500000000002, 22.275000000000002, 22.302500000000002, 22.330000000000002, 22.3575, 22.385, 22.4125, 22.44, 22.467500000000005, 22.495000000000005, 22.522500000000004, 22.550000000000004, 22.577500000000004, 22.605000000000004, 22.632500000000004, 22.660000000000004, 22.687500000000004, 22.715000000000003, 22.742500000000003, 22.770000000000003, 22.797500000000003, 22.825000000000003, 22.852500000000003, 22.880000000000003, 22.907500000000002, 22.935000000000002, 22.962500000000002, 22.990000000000002, 23.017500000000002, 23.045, 23.0725, 23.1, 23.1275, 23.155000000000005, 23.182500000000005, 23.210000000000004, 23.237500000000004, 23.265000000000004, 23.292500000000004, 23.320000000000004, 23.347500000000004, 23.375000000000004, 23.402500000000003, 23.430000000000003, 23.457500000000003, 23.485000000000003, 23.512500000000003, 23.540000000000003, 23.567500000000003, 23.595000000000002, 23.622500000000002, 23.650000000000002, 23.677500000000002, 23.705000000000002, 23.7325, 23.76, 23.7875, 23.815000000000005, 23.842500000000005, 23.870000000000005, 23.897500000000004, 23.925000000000004, 23.952500000000004, 23.980000000000004, 24.007500000000004, 24.035000000000004, 24.062500000000004, 24.090000000000003, 24.117500000000003, 24.145000000000003, 24.172500000000003, 24.200000000000003, 24.227500000000003, 24.255000000000003, 24.282500000000002, 24.310000000000002, |

| Description | Value |
|---|---|
| | 24.337500000000002, 24.365000000000002, 24.392500000000002, 24.42, 24.4475, 24.475000000000005, 24.502500000000005, 24.530000000000005, 24.557500000000005, 24.585000000000004, 24.612500000000004, 24.640000000000004, 24.667500000000004, 24.695000000000004, 24.722500000000004, 24.750000000000004, 24.777500000000003, 24.805000000000003, 24.832500000000003, 24.860000000000003, 24.887500000000003, 24.915000000000003, 24.942500000000003, 24.970000000000002, 24.997500000000002, 25.025000000000002, 25.052500000000002, 25.080000000000002, 25.1075, 25.135, 25.162500000000005, 25.190000000000005, 25.217500000000005, 25.245000000000005, 25.272500000000004, 25.300000000000004, 25.327500000000004, 25.355000000000004, 25.382500000000004, 25.410000000000004, 25.437500000000004, 25.465000000000003, 25.492500000000003, 25.520000000000003, 25.547500000000003, 25.575000000000003, 25.602500000000003, 25.630000000000003, 25.657500000000002, 25.685000000000002, 25.712500000000002, 25.740000000000002, 25.767500000000002, 25.795, 25.822500000000005, 25.850000000000005, 25.877500000000005, 25.905000000000005, 25.932500000000005, 25.960000000000004, 25.987500000000004, 26.015000000000004, 26.042500000000004, 26.070000000000004, 26.097500000000004, 26.125000000000004, 26.152500000000003, 26.180000000000003, 26.207500000000003, 26.235000000000003, 26.262500000000003, 26.290000000000003, 26.317500000000003, 26.345000000000002, 26.372500000000002, 26.400000000000002, 26.427500000000002, 26.455000000000002, 26.4825, 26.510000000000005, 26.537500000000005, 26.565000000000005, 26.592500000000005, 26.620000000000005, 26.647500000000004, 26.675000000000004, 26.702500000000004, 26.730000000000004, 26.757500000000004, 26.785000000000004, 26.812500000000004, 26.840000000000003, 26.867500000000003, 26.895000000000003, 26.922500000000003, 26.950000000000003, 26.977500000000003, 27.005000000000003, 27.032500000000002, 27.060000000000002, 27.087500000000002, 27.115000000000002, 27.142500000000002, 27.170000000000005, 27.197500000000005, 27.225000000000005, 27.252500000000005, 27.280000000000005, 27.307500000000005, 27.335000000000004, 27.362500000000004, 27.390000000000004, 27.417500000000004, 27.445000000000004, 27.472500000000004, 27.500000000000004, 27.527500000000003, 27.555000000000003, 27.582500000000003, 27.610000000000003, 27.637500000000003, 27.665000000000003, 27.692500000000003, 27.720000000000002, 27.747500000000002, 27.775000000000002, 27.802500000000002, 27.830000000000005, 27.857500000000005, 27.885000000000005, 27.912500000000005, 27.940000000000005, 27.967500000000005, 27.995000000000005, 28.022500000000004, 28.050000000000004, 28.077500000000004, 28.105000000000004, 28.132500000000004, 28.160000000000004, 28.187500000000004, 28.215000000000003, 28.242500000000003, 28.270000000000003, 28.297500000000003, 28.325000000000003, 28.352500000000003, |

| Description | Value |
| --- | --- |
| | 28.380000000000003, 28.407500000000002, 28.435000000000002, 28.462500000000002, 28.490000000000002, 28.517500000000005, 28.545000000000005, 28.572500000000005, 28.600000000000005, 28.627500000000005, 28.655000000000005, 28.682500000000005, 28.710000000000004, 28.737500000000004, 28.765000000000004, 28.792500000000004, 28.820000000000004, 28.847500000000004, 28.875000000000004, 28.902500000000003, 28.930000000000003, 28.957500000000003, 28.985000000000003, 29.012500000000003, 29.040000000000003, 29.067500000000003, 29.095000000000002, 29.122500000000002, 29.150000000000002, 29.177500000000006, 29.205000000000005, 29.232500000000005, 29.260000000000005, 29.287500000000005, 29.315000000000005, 29.342500000000005, 29.370000000000005, 29.397500000000004, 29.425000000000004, 29.452500000000004, 29.480000000000004, 29.507500000000004, 29.535000000000004, 29.562500000000004, 29.590000000000003, 29.617500000000003, 29.645000000000003, 29.672500000000003, 29.700000000000003, 29.727500000000003, 29.755000000000003, 29.782500000000002, 29.810000000000002, 29.837500000000006, 29.865000000000006, 29.892500000000005, 29.920000000000005, 29.947500000000005, 29.975000000000005, 30.002500000000005, 30.030000000000005, 30.057500000000005, 30.085000000000004, 30.112500000000004, 30.140000000000004, 30.167500000000004, 30.195000000000004, 30.222500000000004, 30.250000000000004, 30.277500000000003, 30.305000000000003, 30.332500000000003, 30.360000000000003, 30.387500000000003, 30.415000000000003, 30.442500000000003, 30.470000000000002, 30.497500000000002, 30.525000000000006, 30.552500000000006, 30.580000000000005, 30.607500000000005, 30.635000000000005, 30.662500000000005, 30.690000000000005, 30.717500000000005, 30.745000000000005, 30.772500000000004, 30.800000000000004, 30.827500000000004, 30.855000000000004, 30.882500000000004, 30.910000000000004, 30.937500000000004, 30.965000000000003, 30.992500000000003, 31.020000000000003, 31.047500000000003, 31.075000000000003, 31.102500000000003, 31.130000000000003, 31.157500000000002, 31.185000000000006, 31.212500000000006, 31.240000000000006, 31.267500000000005, 31.295000000000005, 31.322500000000005, 31.350000000000005, 31.377500000000005, 31.405000000000005, 31.432500000000005, 31.460000000000004, 31.487500000000004, 31.515000000000004, 31.542500000000004, 31.570000000000004, 31.597500000000004, 31.625000000000004, 31.652500000000003, 31.680000000000003, 31.707500000000003, 31.735000000000003, 31.762500000000003, 31.790000000000003, 31.817500000000003, 31.845000000000002, 31.872500000000006, 31.900000000000006, 31.927500000000006, 31.955000000000005, 31.982500000000005, 32.010000000000005, 32.0375, 32.065000000000005, 32.0925, 32.120000000000005, 32.1475, 32.175000000000004, 32.20250000000001, 32.230000000000004, 32.25750000000001, 32.285000000000004, 32.31250000000001, 32.34, 32.36750000000001, 32.395, |

| Description | Value |
| --- | --- |
| | 32.42250000000001, 32.45, 32.477500000000006, 32.505, 32.532500000000006, 32.56, 32.587500000000006, 32.615, 32.642500000000005, 32.67, 32.697500000000005, 32.725, 32.752500000000005, 32.78, 32.807500000000005, 32.835, 32.862500000000004, 32.89000000000001, 32.917500000000004, 32.94500000000001, 32.972500000000004, 33.00000000000001, 33.0275, 33.05500000000001, 33.0825, 33.11000000000001, 33.1375, 33.165000000000006, 33.1925, 33.220000000000006, 33.2475, 33.275000000000006, 33.3025, 33.330000000000005, 33.3575, 33.385000000000005, 33.4125, 33.440000000000005, 33.4675, 33.495000000000005, 33.5225, 33.550000000000004, 33.57750000000001, 33.605000000000004, 33.63250000000001, 33.660000000000004, 33.68750000000001, 33.715, 33.74250000000001, 33.77, 33.79750000000001, 33.825, 33.852500000000006, 33.88, 33.907500000000006, 33.935, 33.962500000000006, 33.99, 34.017500000000005, 34.045, 34.072500000000005, 34.1, 34.127500000000005, 34.155, 34.182500000000005, 34.21000000000001, 34.237500000000004, 34.26500000000001, 34.292500000000004, 34.32000000000001, 34.347500000000004, 34.37500000000001, 34.4025, 34.43000000000001, 34.4575, 34.48500000000001, 34.5125, 34.540000000000006, 34.5675, 34.595000000000006, 34.6225, 34.650000000000006, 34.6775, 34.705000000000005, 34.7325, 34.760000000000005, 34.7875, 34.815000000000005, 34.8425, 34.870000000000005, 34.89750000000001, 34.925000000000004, 34.95250000000001, 34.980000000000004, 35.00750000000001, 35.035000000000004, 35.06250000000001, 35.09, 35.11750000000001, 35.145, 35.17250000000001, 35.2, 35.227500000000006, 35.255, 35.282500000000006, 35.31, 35.337500000000006, 35.365, 35.392500000000005, 35.42, 35.447500000000005, 35.475, 35.502500000000005, 35.53, 35.557500000000005, 35.58500000000001, 35.612500000000004, 35.64000000000001, 35.667500000000004, 35.69500000000001, 35.722500000000004, 35.75000000000001, 35.7775, 35.80500000000001, 35.8325, 35.86000000000001, 35.8875, 35.915000000000006, 35.9425, 35.970000000000006, 35.9975, 36.025000000000006, 36.0525, 36.080000000000005, 36.1075, 36.135000000000005, 36.1625, 36.190000000000005, 36.21750000000001, 36.245000000000005, 36.27250000000001, 36.300000000000004, 36.32750000000001, 36.355000000000004, 36.38250000000001, 36.410000000000004, 36.43750000000001, 36.465, 36.49250000000001, 36.52, 36.54750000000001, 36.575, 36.602500000000006, 36.63, 36.657500000000006, 36.685, 36.712500000000006, 36.74, 36.767500000000005, 36.795, 36.822500000000005, 36.85, 36.877500000000005, 36.90500000000001, 36.932500000000005, 36.96000000000001, 36.987500000000004, 37.01500000000001, 37.042500000000004, 37.07000000000001, 37.097500000000004, 37.12500000000001, 37.1525, 37.18000000000001, 37.2075, 37.23500000000001, 37.2625, 37.290000000000006, 37.3175, 37.345000000000006, 37.3725, 37.400000000000006, 37.4275, |

| Description | Value |
| --- | --- |
| | 37.455000000000005, 37.4825, 37.510000000000005, 37.5375, 37.565000000000005, 37.59250000000001, 37.620000000000005, 37.64750000000001, 37.675000000000004, 37.70250000000001, 37.730000000000004, 37.75750000000001, 37.785000000000004, 37.81250000000001, 37.84, 37.86750000000001, 37.895, 37.92250000000001, 37.95, 37.977500000000006, 38.005, 38.032500000000006, 38.06, 38.087500000000006, 38.115, 38.142500000000005, 38.17, 38.197500000000005, 38.22500000000001, 38.252500000000005, 38.28000000000001, 38.307500000000005, 38.33500000000001, 38.362500000000004, 38.39000000000001, 38.417500000000004, 38.44500000000001, 38.472500000000004, 38.50000000000001, 38.5275, 38.55500000000001, 38.5825, 38.61000000000001, 38.6375, 38.665000000000006, 38.6925, 38.720000000000006, 38.7475, 38.775000000000006, 38.8025, 38.830000000000005, 38.8575, 38.885000000000005, 38.91250000000001, 38.940000000000005, 38.96750000000001, 38.995000000000005, 39.02250000000001, 39.050000000000004, 39.07750000000001, 39.105000000000004, 39.13250000000001, 39.160000000000004, 39.18750000000001, 39.215, 39.24250000000001, 39.27, 39.29750000000001, 39.325, 39.352500000000006, 39.38, 39.407500000000006, 39.435, 39.462500000000006, 39.49, 39.517500000000005, 39.545, 39.572500000000005, 39.60000000000001, 39.627500000000005, 39.65500000000001, 39.682500000000005, 39.71000000000001, 39.737500000000004, 39.76500000000001, 39.792500000000004, 39.82000000000001, 39.847500000000004, 39.87500000000001, 39.9025, 39.93000000000001, 39.9575, 39.98500000000001, 40.0125, 40.040000000000006, 40.0675, 40.095000000000006, 40.1225, 40.150000000000006, 40.1775, 40.205000000000005, 40.23250000000001, 40.260000000000005, 40.28750000000001, 40.315000000000005, 40.34250000000001, 40.370000000000005, 40.39750000000001, 40.425000000000004, 40.45250000000001, 40.480000000000004, 40.50750000000001, 40.535000000000004, 40.56250000000001, 40.59, 40.61750000000001, 40.645, 40.67250000000001, 40.7, 40.727500000000006, 40.755, 40.782500000000006, 40.81, 40.837500000000006, 40.865, 40.892500000000005, 40.92000000000001, 40.947500000000005, 40.97500000000001, 41.002500000000005, 41.03000000000001, 41.057500000000005, 41.08500000000001, 41.112500000000004, 41.14000000000001, 41.167500000000004, 41.19500000000001, 41.222500000000004, 41.25000000000001, 41.2775, 41.30500000000001, 41.3325, 41.36000000000001, 41.3875, 41.415000000000006, 41.4425, 41.470000000000006, 41.4975, 41.525000000000006, 41.5525, 41.580000000000005, 41.60750000000001, 41.635000000000005, 41.66250000000001, 41.690000000000005, 41.71750000000001, 41.745000000000005, 41.77250000000001, 41.800000000000004, 41.82750000000001, 41.855000000000004, 41.88250000000001, 41.910000000000004, 41.93750000000001, 41.965, 41.99250000000001, 42.02, 42.04750000000001, 42.075, 42.102500000000006, 42.13, |

| Description | Value |
| --- | --- |
| | 42.157500000000006, 42.185, 42.212500000000006, 42.24000000000001, 42.267500000000005, 42.29500000000001, 42.322500000000005, 42.35000000000001, 42.377500000000005, 42.40500000000001, 42.432500000000005, 42.46000000000001, 42.487500000000004, 42.51500000000001, 42.542500000000004, 42.57000000000001, 42.597500000000004, 42.62500000000001, 42.6525, 42.68000000000001, 42.7075, 42.73500000000001, 42.7625, 42.790000000000006, 42.8175, 42.845000000000006, 42.8725, 42.900000000000006, 42.92750000000001, 42.955000000000005, 42.98250000000001, 43.010000000000005, 43.03750000000001, 43.065000000000005, 43.09250000000001, 43.120000000000005, 43.14750000000001, 43.175000000000004, 43.20250000000001, 43.230000000000004, 43.25750000000001, 43.285000000000004, 43.31250000000001, 43.34, 43.36750000000001, 43.395, 43.42250000000001, 43.45, 43.477500000000006, 43.505, 43.532500000000006, 43.56, 43.587500000000006, 43.61500000000001, 43.642500000000005, 43.67000000000001, 43.697500000000005, 43.72500000000001, 43.752500000000005, 43.78000000000001, 43.807500000000005, 43.83500000000001, 43.862500000000004, 43.89000000000001, 43.917500000000004, 43.94500000000001, 43.972500000000004, 44.00000000000001, 44.0275, 44.05500000000001, 44.0825, 44.11000000000001, 44.1375, 44.165000000000006, 44.1925, 44.220000000000006, 44.2475, 44.275000000000006, 44.30250000000001, 44.330000000000005, 44.35750000000001, 44.385000000000005, 44.41250000000001, 44.440000000000005, 44.46750000000001, 44.495000000000005, 44.52250000000001, 44.550000000000004, 44.57750000000001, 44.605000000000004, 44.63250000000001, 44.660000000000004, 44.68750000000001, 44.715, 44.74250000000001, 44.77, 44.79750000000001, 44.825, 44.852500000000006, 44.88, 44.907500000000006, 44.93500000000001, 44.962500000000006, 44.99000000000001, 45.017500000000005, 45.04500000000001, 45.072500000000005, 45.10000000000001, 45.127500000000005, 45.15500000000001, 45.182500000000005, 45.21000000000001, 45.237500000000004, 45.26500000000001, 45.292500000000004, 45.32000000000001, 45.347500000000004, 45.37500000000001, 45.4025, 45.43000000000001, 45.4575, 45.48500000000001, 45.5125, 45.540000000000006, 45.5675, 45.595000000000006, 45.62250000000001, 45.650000000000006, 45.67750000000001, 45.705000000000005, 45.73250000000001, 45.760000000000005, 45.78750000000001, 45.815000000000005, 45.84250000000001, 45.870000000000005, 45.89750000000001, 45.925000000000004, 45.95250000000001, 45.980000000000004, 46.00750000000001, 46.035000000000004, 46.06250000000001, 46.09, 46.11750000000001, 46.145, 46.17250000000001, 46.2, 46.227500000000006, 46.255, 46.282500000000006, 46.31000000000001, 46.337500000000006, 46.36500000000001, 46.392500000000005, 46.42000000000001, 46.447500000000005, 46.47500000000001, 46.502500000000005, 46.53000000000001, 46.557500000000005, 46.58500000000001, 46.612500000000004, 46.64000000000001, 46.667500000000004, |

| Description | Value |
| --- | --- |
| | 46.69500000000001, 46.722500000000004, 46.75000000000001, 46.7775, 46.80500000000001, 46.8325, 46.86000000000001, 46.8875, 46.915000000000006, 46.94250000000001, 46.970000000000006, 46.99750000000001, 47.025000000000006, 47.05250000000001, 47.080000000000005, 47.10750000000001, 47.135000000000005, 47.16250000000001, 47.190000000000005, 47.21750000000001, 47.245000000000005, 47.27250000000001, 47.300000000000004, 47.32750000000001, 47.355000000000004, 47.38250000000001, 47.410000000000004, 47.43750000000001, 47.465, 47.49250000000001, 47.52, 47.54750000000001, 47.575, 47.602500000000006, 47.63000000000001, 47.657500000000006, 47.68500000000001, 47.712500000000006, 47.74000000000001, 47.767500000000005, 47.79500000000001, 47.822500000000005, 47.85000000000001, 47.877500000000005, 47.90500000000001, 47.932500000000005, 47.96000000000001, 47.987500000000004, 48.01500000000001, 48.042500000000004, 48.07000000000001, 48.097500000000004, 48.12500000000001, 48.1525, 48.18000000000001, 48.2075, 48.23500000000001, 48.2625, 48.290000000000006, 48.31750000000001, 48.345000000000006, 48.37250000000001, 48.400000000000006, 48.42750000000001, 48.455000000000005, 48.48250000000001, 48.510000000000005, 48.53750000000001, 48.565000000000005, 48.59250000000001, 48.620000000000005, 48.64750000000001, 48.675000000000004, 48.70250000000001, 48.730000000000004, 48.75750000000001, 48.785000000000004, 48.81250000000001, 48.84, 48.86750000000001, 48.895, 48.92250000000001, 48.95000000000001, 48.977500000000006, 49.00500000000001, 49.032500000000006, 49.06000000000001, 49.087500000000006, 49.11500000000001, 49.142500000000005, 49.17000000000001, 49.197500000000005, 49.22500000000001, 49.252500000000005, 49.28000000000001, 49.307500000000005, 49.33500000000001, 49.362500000000004, 49.39000000000001, 49.417500000000004, 49.44500000000001, 49.472500000000004, 49.50000000000001, 49.5275, 49.55500000000001, 49.5825, 49.61000000000001, 49.63750000000001, 49.665000000000006, 49.69250000000001, 49.720000000000006, 49.74750000000001, 49.775000000000006, 49.80250000000001, 49.830000000000005, 49.85750000000001, 49.885000000000005, 49.91250000000001, 49.940000000000005, 49.96750000000001, 49.995000000000005, 50.02250000000001, 50.050000000000004, 50.07750000000001, 50.105000000000004, 50.13250000000001, 50.160000000000004, 50.18750000000001, 50.215, 50.24250000000001, 50.27, 50.29750000000001, 50.32500000000001, 50.352500000000006, 50.38000000000001, 50.407500000000006, 50.43500000000001, 50.462500000000006, 50.49000000000001, 50.517500000000005, 50.54500000000001, 50.572500000000005, 50.60000000000001, 50.627500000000005, 50.65500000000001, 50.682500000000005, 50.71000000000001, 50.737500000000004, 50.76500000000001, 50.792500000000004, 50.82000000000001, 50.847500000000004, 50.87500000000001, 50.9025, 50.93000000000001, 50.95750000000001, |

| Description | Value |
|---|---|
| | 50.98500000000001, 51.01250000000001, 51.040000000000006, 51.06750000000001, 51.095000000000006, 51.12250000000001, 51.150000000000006, 51.17750000000001, 51.205000000000005, 51.23250000000001, 51.260000000000005, 51.28750000000001, 51.315000000000005, 51.34250000000001, 51.370000000000005, 51.39750000000001, 51.425000000000004, 51.45250000000001, 51.480000000000004, 51.50750000000001, 51.535000000000004, 51.56250000000001, 51.59, 51.61750000000001, 51.64500000000001, 51.67250000000001, 51.70000000000001, 51.727500000000006, 51.75500000000001, 51.782500000000006, 51.81000000000001, 51.837500000000006, 51.86500000000001, 51.892500000000005, 51.92000000000001, 51.947500000000005, 51.97500000000001, 52.002500000000005, 52.03000000000001, 52.057500000000005, 52.08500000000001, 52.112500000000004, 52.14000000000001, 52.167500000000004, 52.19500000000001, 52.222500000000004, 52.25000000000001, 52.2775, 52.30500000000001, 52.33250000000001, 52.36000000000001, 52.38750000000001, 52.415000000000006, 52.44250000000001, 52.470000000000006, 52.49750000000001, 52.525000000000006, 52.55250000000001, 52.580000000000005, 52.60750000000001, 52.635000000000005, 52.66250000000001, 52.690000000000005, 52.71750000000001, 52.745000000000005, 52.77250000000001, 52.800000000000004, 52.82750000000001, 52.855000000000004, 52.88250000000001, 52.910000000000004, 52.93750000000001, 52.965, 52.99250000000001, 53.02000000000001, 53.04750000000001, 53.07500000000001, 53.102500000000006, 53.13000000000001, 53.157500000000006, 53.18500000000001, 53.212500000000006, 53.24000000000001, 53.267500000000005, 53.29500000000001, 53.322500000000005, 53.35000000000001, 53.377500000000005, 53.40500000000001, 53.432500000000005, 53.46000000000001, 53.487500000000004, 53.51500000000001, 53.542500000000004, 53.57000000000001, 53.597500000000004, 53.62500000000001, 53.65250000000001, 53.68000000000001, 53.70750000000001, 53.73500000000001, 53.76250000000001, 53.790000000000006, 53.81750000000001, 53.845000000000006, 53.87250000000001, 53.900000000000006, 53.92750000000001, 53.955000000000005, 53.98250000000001, 54.010000000000005, 54.03750000000001, 54.065000000000005, 54.09250000000001, 54.120000000000005, 54.14750000000001, 54.175000000000004, 54.20250000000001, 54.230000000000004, 54.25750000000001, 54.285000000000004, 54.31250000000001, 54.34000000000001, 54.36750000000001, 54.39500000000001, 54.42250000000001, 54.45000000000001, 54.477500000000006, 54.50500000000001, 54.532500000000006, 54.56000000000001, 54.587500000000006, 54.61500000000001, 54.642500000000005, 54.67000000000001, 54.697500000000005, 54.72500000000001, 54.752500000000005, 54.78000000000001, 54.807500000000005, 54.83500000000001, 54.862500000000004, 54.89000000000001, 54.917500000000004, 54.94500000000001, 54.972500000000004, 55.00000000000001, |

| Description | Value |
| --- | --- |
| | 55.02750000000001, 55.05500000000001, 55.08250000000001, 55.11000000000001, 55.13750000000001, 55.165000000000006, 55.19250000000001, 55.220000000000006, 55.24750000000001, 55.275000000000006, 55.30250000000001, 55.330000000000005, 55.35750000000001, 55.385000000000005, 55.41250000000001, 55.440000000000005, 55.46750000000001, 55.495000000000005, 55.52250000000001, 55.550000000000004, 55.57750000000001, 55.605000000000004, 55.63250000000001, 55.66000000000001, 55.68750000000001, 55.71500000000001, 55.74250000000001, 55.77000000000001, 55.79750000000001, 55.82500000000001, 55.852500000000006, 55.88000000000001, 55.907500000000006, 55.93500000000001, 55.962500000000006, 55.99000000000001, 56.017500000000005, 56.04500000000001, 56.072500000000005, 56.10000000000001, 56.127500000000005, 56.15500000000001, 56.182500000000005, 56.21000000000001, 56.237500000000004, 56.26500000000001, 56.292500000000004, 56.32000000000001, 56.34750000000001, 56.37500000000001, 56.40250000000001, 56.43000000000001, 56.45750000000001, 56.48500000000001, 56.51250000000001, 56.540000000000006, 56.56750000000001, 56.595000000000006, 56.62250000000001, 56.650000000000006, 56.67750000000001, 56.705000000000005, 56.73250000000001, 56.760000000000005, 56.78750000000001, 56.815000000000005, 56.84250000000001, 56.870000000000005, 56.89750000000001, 56.925000000000004, 56.95250000000001, 56.980000000000004, 57.00750000000001, 57.03500000000001, 57.06250000000001, 57.09000000000001, 57.11750000000001, 57.14500000000001, 57.17250000000001, 57.20000000000001, 57.227500000000006, 57.25500000000001, 57.282500000000006, 57.31000000000001, 57.337500000000006, 57.36500000000001, 57.392500000000005, 57.42000000000001, 57.447500000000005, 57.47500000000001, 57.502500000000005, 57.53000000000001, 57.557500000000005, 57.58500000000001, 57.612500000000004, 57.64000000000001, 57.66750000000001, 57.69500000000001, 57.72250000000001, 57.75000000000001, 57.77750000000001, 57.80500000000001, 57.83250000000001, 57.86000000000001, 57.88750000000001, 57.915000000000006, 57.94250000000001, 57.970000000000006, 57.99750000000001, 58.025000000000006, 58.05250000000001, 58.080000000000005, 58.10750000000001, 58.135000000000005, 58.16250000000001, 58.190000000000005, 58.21750000000001, 58.245000000000005, 58.27250000000001, 58.300000000000004, 58.32750000000001, 58.35500000000001, 58.38250000000001, 58.41000000000001, 58.43750000000001, 58.46500000000001, 58.49250000000001, 58.52000000000001, 58.54750000000001, 58.57500000000001, 58.602500000000006, 58.63000000000001, 58.657500000000006, 58.68500000000001, 58.712500000000006, 58.74000000000001, 58.767500000000005, 58.79500000000001, 58.822500000000005, 58.85000000000001, 58.877500000000005, 58.90500000000001, 58.932500000000005, 58.96000000000001, |

| Description | Value |
| --- | --- |
| | 58.987500000000004, 59.01500000000001, 59.04250000000001, 59.07000000000001, 59.09750000000001, 59.12500000000001, 59.15250000000001, 59.18000000000001, 59.20750000000001, 59.23500000000001, 59.26250000000001, 59.290000000000006, 59.31750000000001, 59.345000000000006, 59.37250000000001, 59.400000000000006, 59.42750000000001, 59.455000000000005, 59.48250000000001, 59.510000000000005, 59.53750000000001, 59.565000000000005, 59.59250000000001, 59.620000000000005, 59.64750000000001, 59.67500000000001, 59.70250000000001, 59.73000000000001, 59.75750000000001, 59.78500000000001, 59.81250000000001, 59.84000000000001, 59.86750000000001, 59.89500000000001, 59.92250000000001, 59.95000000000001, 59.977500000000006, 60.00500000000001, 60.032500000000006, 60.06000000000001, 60.087500000000006, 60.11500000000001, 60.142500000000005, 60.17000000000001, 60.197500000000005, 60.22500000000001, 60.252500000000005, 60.28000000000001, 60.307500000000005, 60.33500000000001, 60.36250000000001, 60.39000000000001, 60.41750000000001, 60.44500000000001, 60.47250000000001, 60.50000000000001, 60.52750000000001, 60.55500000000001, 60.58250000000001, 60.61000000000001, 60.63750000000001, 60.665000000000006, 60.69250000000001, 60.720000000000006, 60.74750000000001, 60.775000000000006, 60.80250000000001, 60.830000000000005, 60.85750000000001, 60.885000000000005, 60.91250000000001, 60.940000000000005, 60.96750000000001, 60.995000000000005, 61.02250000000001, 61.05000000000001, 61.07750000000001, 61.10500000000001, 61.13250000000001, 61.16000000000001, 61.18750000000001, 61.21500000000001, 61.24250000000001, 61.27000000000001, 61.29750000000001, 61.32500000000001, 61.352500000000006, 61.38000000000001, 61.407500000000006, 61.43500000000001, 61.462500000000006, 61.49000000000001, 61.517500000000005, 61.54500000000001, 61.572500000000005, 61.60000000000001, 61.627500000000005, 61.65500000000001, 61.682500000000005, 61.71000000000001, 61.73750000000001, 61.76500000000001, 61.79250000000001, 61.82000000000001, 61.84750000000001, 61.87500000000001, 61.90250000000001, 61.93000000000001, 61.95750000000001, 61.98500000000001, 62.01250000000001, 62.040000000000006, 62.06750000000001, 62.095000000000006, 62.12250000000001, 62.150000000000006, 62.17750000000001, 62.205000000000005, 62.23250000000001, 62.260000000000005, 62.28750000000001, 62.315000000000005, 62.34250000000001, 62.37000000000001, 62.39750000000001, 62.42500000000001, 62.45250000000001, 62.48000000000001, 62.50750000000001, 62.53500000000001, 62.56250000000001, 62.59000000000001, 62.61750000000001, 62.64500000000001, 62.67250000000001, 62.70000000000001, 62.727500000000006, 62.75500000000001, 62.782500000000006, 62.81000000000001, 62.837500000000006, 62.86500000000001, 62.892500000000005, 62.92000000000001, |

| Description | Value |
| --- | --- |
| | 62.947500000000005, 62.97500000000001, 63.002500000000005, 63.03000000000001, 63.05750000000001, 63.08500000000001, 63.11250000000001, 63.14000000000001, 63.16750000000001, 63.19500000000001, 63.22250000000001, 63.25000000000001, 63.27750000000001, 63.30500000000001, 63.33250000000001, 63.36000000000001, 63.38750000000001, 63.415000000000006, 63.44250000000001, 63.470000000000006, 63.49750000000001, 63.525000000000006, 63.55250000000001, 63.580000000000005, 63.60750000000001, 63.635000000000005, 63.66250000000001, 63.690000000000005, 63.71750000000001, 63.74500000000001, 63.77250000000001, 63.80000000000001, 63.82750000000001, 63.85500000000001, 63.88250000000001, 63.91000000000001, 63.93750000000001, 63.96500000000001, 63.99250000000001, 64.02000000000001, 64.04750000000001, 64.075, 64.1025, 64.13000000000001, 64.15750000000001, 64.185, 64.2125, 64.24000000000001, 64.26750000000001, 64.295, 64.3225, 64.35000000000001, 64.37750000000001, 64.40500000000002, 64.4325, 64.46000000000001, 64.48750000000001, 64.51500000000001, 64.5425, 64.57000000000001, 64.59750000000001, 64.62500000000001, 64.6525, 64.68, 64.70750000000001, 64.73500000000001, 64.7625, 64.79, 64.81750000000001, 64.84500000000001, 64.8725, 64.9, 64.92750000000001, 64.95500000000001, 64.9825, 65.01, 65.03750000000001, 65.06500000000001, 65.09250000000002, 65.12, 65.14750000000001, 65.17500000000001, 65.20250000000001, 65.23, 65.25750000000001, 65.28500000000001, 65.31250000000001, 65.34, 65.3675, 65.39500000000001, 65.42250000000001, 65.45, 65.4775, 65.50500000000001, 65.53250000000001, 65.56, 65.5875, 65.61500000000001, 65.64250000000001, 65.67, 65.6975, 65.72500000000001, 65.75250000000001, 65.78000000000002, 65.8075, 65.83500000000001, 65.86250000000001, 65.89000000000001, 65.9175, 65.94500000000001, 65.97250000000001, 66.00000000000001, 66.0275, 66.055, 66.08250000000001, 66.11000000000001, 66.1375, 66.165, 66.19250000000001, 66.22000000000001, 66.2475, 66.275, 66.30250000000001, 66.33000000000001, 66.3575, 66.385, 66.41250000000001, 66.44000000000001, 66.46750000000002, 66.495, 66.52250000000001, 66.55000000000001, 66.57750000000001, 66.605, 66.63250000000001, 66.66000000000001, 66.68750000000001, 66.715, 66.7425, 66.77000000000001, 66.79750000000001, 66.825, 66.8525, 66.88000000000001, 66.90750000000001, 66.935, 66.9625, 66.99000000000001, 67.01750000000001, 67.045, 67.0725, 67.10000000000001, 67.12750000000001, 67.15500000000002, 67.1825, 67.21000000000001, 67.23750000000001, 67.26500000000001, 67.2925, 67.32000000000001, 67.34750000000001, 67.37500000000001, 67.4025, 67.43, 67.45750000000001, 67.48500000000001, 67.5125, 67.54, 67.56750000000001, 67.59500000000001, 67.6225, 67.65, 67.67750000000001, 67.70500000000001, 67.73250000000002, 67.76, 67.78750000000001, 67.81500000000001, 67.84250000000002, 67.87, 67.89750000000001, 67.92500000000001, 67.95250000000001, 67.98, |

| Description | Value |
| --- | --- |
| | 68.00750000000001, 68.03500000000001, 68.06250000000001, 68.09, 68.1175, 68.14500000000001, 68.17250000000001, 68.2, 68.2275, 68.25500000000001, 68.28250000000001, 68.31, 68.3375, 68.36500000000001, 68.39250000000001, 68.42000000000002, 68.4475, 68.47500000000001, 68.50250000000001, 68.53000000000002, 68.5575, 68.58500000000001, 68.61250000000001, 68.64000000000001, 68.6675, 68.69500000000001, 68.72250000000001, 68.75000000000001, 68.7775, 68.805, 68.83250000000001, 68.86000000000001, 68.8875, 68.915, 68.94250000000001, 68.97000000000001, 68.9975, 69.025, 69.05250000000001, 69.08000000000001, 69.10750000000002, 69.135, 69.16250000000001, 69.19000000000001, 69.21750000000002, 69.245, 69.27250000000001, 69.30000000000001, 69.32750000000001, 69.355, 69.38250000000001, 69.41000000000001, 69.43750000000001, 69.465, 69.4925, 69.52000000000001, 69.54750000000001, 69.575, 69.6025, 69.63000000000001, 69.65750000000001, 69.685, 69.7125, 69.74000000000001, 69.76750000000001, 69.79500000000002, 69.8225, 69.85000000000001, 69.87750000000001, 69.90500000000002, 69.9325, 69.96000000000001, 69.98750000000001, 70.01500000000001, 70.0425, 70.07000000000001, 70.09750000000001, 70.12500000000001, 70.1525, 70.18, 70.20750000000001, 70.23500000000001, 70.2625, 70.29, 70.31750000000001, 70.34500000000001, 70.3725, 70.4, 70.42750000000001, 70.45500000000001, 70.48250000000002, 70.51, 70.53750000000001, 70.56500000000001, 70.59250000000002, 70.62, 70.64750000000001, 70.67500000000001, 70.70250000000001, 70.73, 70.75750000000001, 70.78500000000001, 70.81250000000001, 70.84, 70.8675, 70.89500000000001, 70.92250000000001, 70.95, 70.9775, 71.00500000000001, 71.03250000000001, 71.06, 71.0875, 71.11500000000001, 71.14250000000001, 71.17000000000002, 71.1975, 71.22500000000001, 71.25250000000001, 71.28000000000002, 71.3075, 71.33500000000001, 71.36250000000001, 71.39000000000001, 71.4175, 71.44500000000001, 71.47250000000001, 71.50000000000001, 71.5275, 71.555, 71.58250000000001, 71.61000000000001, 71.6375, 71.665, 71.69250000000001, 71.72000000000001, 71.74750000000002, 71.775, 71.80250000000001, 71.83000000000001, 71.85750000000002, 71.885, 71.91250000000001, 71.94000000000001, 71.96750000000002, 71.995, 72.02250000000001, 72.05000000000001, 72.07750000000001, 72.105, 72.13250000000001, 72.16000000000001, 72.18750000000001, 72.215, 72.2425, 72.27000000000001, 72.29750000000001, 72.325, 72.3525, 72.38000000000001, 72.40750000000001, 72.43500000000002, 72.4625, 72.49000000000001, 72.51750000000001, 72.54500000000002, 72.5725, 72.60000000000001, 72.62750000000001, 72.65500000000002, 72.6825, 72.71000000000001, 72.73750000000001, 72.76500000000001, 72.7925, 72.82000000000001, 72.84750000000001, 72.87500000000001, 72.9025, 72.93, 72.95750000000001, 72.98500000000001, 73.0125, 73.04, 73.06750000000001, 73.09500000000001, 73.12250000000002, 73.15, 73.17750000000001, 73.20500000000001, 73.23250000000002, 73.26, 73.28750000000001, 73.31500000000001, 73.34250000000002, 73.37, 73.39750000000001, 73.42500000000001, 73.45250000000001, 73.48, |

| Description | Value |
| --- | --- |
| | 73.50750000000001, 73.53500000000001, 73.56250000000001, 73.59, 73.6175, 73.64500000000001, 73.67250000000001, 73.7, 73.7275, 73.75500000000001, 73.78250000000001, 73.81000000000002, 73.8375, 73.86500000000001, 73.89250000000001, 73.92000000000002, 73.9475, 73.97500000000001, 74.00250000000001, 74.03000000000002, 74.0575, 74.08500000000001, 74.11250000000001, 74.14000000000001, 74.1675, 74.19500000000001, 74.22250000000001, 74.25000000000001, 74.2775, 74.305, 74.33250000000001, 74.36000000000001, 74.3875, 74.415, 74.44250000000001, 74.47000000000001, 74.49750000000002, 74.525, 74.55250000000001, 74.58000000000001, 74.60750000000002, 74.635, 74.66250000000001, 74.69000000000001, 74.71750000000002, 74.745, 74.77250000000001, 74.80000000000001, 74.82750000000001, 74.855, 74.88250000000001, 74.91000000000001, 74.93750000000001, 74.965, 74.9925, 75.02000000000001, 75.04750000000001, 75.075, 75.1025, 75.13000000000001, 75.15750000000001, 75.18500000000002, 75.2125, 75.24000000000001, 75.26750000000001, 75.29500000000002, 75.3225, 75.35000000000001, 75.37750000000001, 75.40500000000002, 75.4325, 75.46000000000001, 75.48750000000001, 75.51500000000001, 75.5425, 75.57000000000001, 75.59750000000001, 75.62500000000001, 75.6525, 75.68, 75.70750000000001, 75.73500000000001, 75.7625, 75.79, 75.81750000000001, 75.84500000000001, 75.87250000000002, 75.9, 75.92750000000001, 75.95500000000001, 75.98250000000002, 76.01, 76.03750000000001, 76.06500000000001, 76.09250000000002, 76.12, 76.14750000000001, 76.17500000000001, 76.20250000000001, 76.23, 76.25750000000001, 76.28500000000001, 76.31250000000001, 76.34, 76.3675, 76.39500000000001, 76.42250000000001, 76.45000000000002, 76.4775, 76.50500000000001, 76.53250000000001, 76.56000000000002, 76.5875, 76.61500000000001, 76.64250000000001, 76.67000000000002, 76.6975, 76.72500000000001, 76.75250000000001, 76.78000000000002, 76.8075, 76.83500000000001, 76.86250000000001, 76.89000000000001, 76.9175, 76.94500000000001, 76.97250000000001, 77.00000000000001, 77.0275, 77.055, 77.08250000000001, 77.11000000000001, 77.13750000000002, 77.165, 77.19250000000001, 77.22000000000001, 77.24750000000002, 77.275, 77.30250000000001, 77.33000000000001, 77.35750000000002, 77.385, 77.41250000000001, 77.44000000000001, 77.46750000000002, 77.495, 77.52250000000001, 77.55000000000001, 77.57750000000001, 77.605, 77.63250000000001, 77.66000000000001, 77.68750000000001, 77.715, 77.7425, 77.77000000000001, 77.79750000000001, 77.82500000000002, 77.8525, 77.88000000000001, 77.90750000000001, 77.93500000000002, 77.9625, 77.99000000000001, 78.01750000000001, 78.04500000000002, 78.0725, 78.10000000000001, 78.12750000000001, 78.15500000000002, 78.1825, 78.21000000000001, 78.23750000000001, 78.26500000000001, 78.2925, 78.32000000000001, 78.34750000000001, 78.37500000000001, 78.4025, 78.43, 78.45750000000001, 78.48500000000001, 78.51250000000002, 78.54, 78.56750000000001, 78.59500000000001, 78.62250000000002, 78.65, 78.67750000000001, 78.70500000000001, 78.73250000000002, 78.76, 78.78750000000001, 78.81500000000001, 78.84250000000002, 78.87, |

| Description | Value |
| --- | --- |
| | 78.89750000000001, 78.92500000000001, 78.95250000000001, 78.98, 79.00750000000001, 79.03500000000001, 79.06250000000001, 79.09, 79.1175, 79.14500000000001, 79.17250000000001, 79.20000000000002, 79.2275, 79.25500000000001, 79.28250000000001, 79.31000000000002, 79.3375, 79.36500000000001, 79.39250000000001, 79.42000000000002, 79.4475, 79.47500000000001, 79.50250000000001, 79.53000000000002, 79.5575, 79.58500000000001, 79.61250000000001, 79.64000000000001, 79.6675, 79.69500000000001, 79.72250000000001, 79.75000000000001, 79.7775, 79.805, 79.83250000000001, 79.86000000000001, 79.88750000000002, 79.915, 79.94250000000001, 79.97000000000001, 79.99750000000002, 80.025, 80.05250000000001, 80.08000000000001, 80.10750000000002, 80.135, 80.16250000000001, 80.19000000000001, 80.21750000000002, 80.245, 80.27250000000001, 80.30000000000001, 80.32750000000001, 80.355, 80.38250000000001, 80.41000000000001, 80.43750000000001, 80.46500000000002, 80.4925, 80.52000000000001, 80.54750000000001, 80.57500000000002, 80.6025, 80.63000000000001, 80.65750000000001, 80.68500000000002, 80.7125, 80.74000000000001, 80.76750000000001, 80.79500000000002, 80.8225, 80.85000000000001, 80.87750000000001, 80.90500000000002, 80.9325, 80.96000000000001, 80.98750000000001, 81.01500000000001, 81.0425, 81.07000000000001, 81.09750000000001, 81.12500000000001, 81.15250000000002, 81.18, 81.20750000000001, 81.23500000000001, 81.26250000000002, 81.29, 81.31750000000001, 81.34500000000001, 81.37250000000002, 81.4, 81.42750000000001, 81.45500000000001, 81.48250000000002, 81.51, 81.53750000000001, 81.56500000000001, 81.59250000000002, 81.62, 81.64750000000001, 81.67500000000001, 81.70250000000001, 81.73, 81.75750000000001, 81.78500000000001, 81.81250000000001, 81.84000000000002, 81.8675, 81.89500000000001, 81.92250000000001, 81.95000000000002, 81.9775, 82.00500000000001, 82.03250000000001, 82.06000000000002, 82.0875, 82.11500000000001, 82.14250000000001, 82.17000000000002, 82.1975, 82.22500000000001, 82.25250000000001, 82.28000000000002, 82.3075, 82.33500000000001, 82.36250000000001, 82.39000000000001, 82.4175, 82.44500000000001, 82.47250000000001, 82.50000000000001, 82.52750000000002, 82.555, 82.58250000000001, 82.61000000000001, 82.63750000000002, 82.665, 82.69250000000001, 82.72000000000001, 82.74750000000002, 82.775, 82.80250000000001, 82.83000000000001, 82.85750000000002, 82.885, 82.91250000000001, 82.94000000000001, 82.96750000000002, 82.995, 83.02250000000001, 83.05000000000001, 83.07750000000001, 83.105, 83.13250000000001, 83.16000000000001, 83.18750000000001, 83.21500000000002, 83.2425, 83.27000000000001, 83.29750000000001, 83.32500000000002, 83.3525, 83.38000000000001, 83.40750000000001, 83.43500000000002, 83.4625, 83.49000000000001, 83.51750000000001, 83.54500000000002, 83.5725, 83.60000000000001, 83.62750000000001, 83.65500000000002, 83.6825, 83.71000000000001, 83.73750000000001, 83.76500000000001, 83.7925, 83.82000000000001, 83.84750000000001, 83.87500000000001, 83.90250000000002, 83.93, 83.95750000000001, 83.98500000000001, 84.01250000000002, 84.04, 84.06750000000001, 84.09500000000001, |

| Description | Value |
| --- | --- |
| | 84.12250000000002, 84.15, 84.17750000000001, 84.20500000000001, 84.23250000000002, 84.26, 84.28750000000001, 84.31500000000001, 84.34250000000002, 84.37, 84.39750000000001, 84.42500000000001, 84.45250000000001, 84.48000000000002, 84.50750000000001, 84.53500000000001, 84.56250000000001, 84.59000000000002, 84.6175, 84.64500000000001, 84.67250000000001, 84.70000000000002, 84.7275, 84.75500000000001, 84.78250000000001, 84.81000000000002, 84.8375, 84.86500000000001, 84.89250000000001, 84.92000000000002, 84.9475, 84.97500000000001, 85.00250000000001, 85.03000000000002, 85.0575, 85.08500000000001, 85.11250000000001, 85.14000000000001, 85.16750000000002, 85.19500000000001, 85.22250000000001, 85.25000000000001, 85.27750000000002, 85.305, 85.33250000000001, 85.36000000000001, 85.38750000000002, 85.415, 85.44250000000001, 85.47000000000001, 85.49750000000002, 85.525, 85.55250000000001, 85.58000000000001, 85.60750000000002, 85.635, 85.66250000000001, 85.69000000000001, 85.71750000000002, 85.745, 85.77250000000001, 85.80000000000001, 85.82750000000001, 85.85500000000002, 85.88250000000001, 85.91000000000001, 85.93750000000001, 85.96500000000002, 85.9925, 86.02000000000001, 86.04750000000001, 86.07500000000002, 86.1025, 86.13000000000001, 86.15750000000001, 86.18500000000002, 86.2125, 86.24000000000001, 86.26750000000001, 86.29500000000002, 86.3225, 86.35000000000001, 86.37750000000001, 86.40500000000002, 86.4325, 86.46000000000001, 86.48750000000001, 86.51500000000001, 86.54250000000002, 86.57000000000001, 86.59750000000001, 86.62500000000001, 86.65250000000002, 86.68, 86.70750000000001, 86.73500000000001, 86.76250000000002, 86.79, 86.81750000000001, 86.84500000000001, 86.87250000000002, 86.9, 86.92750000000001, 86.95500000000001, 86.98250000000002, 87.01, 87.03750000000001, 87.06500000000001, 87.09250000000002, 87.12, 87.14750000000001, 87.17500000000001, 87.20250000000001, 87.23000000000002, 87.25750000000001, 87.28500000000001, 87.31250000000001, 87.34000000000002, 87.3675, 87.39500000000001, 87.42250000000001, 87.45000000000002, 87.4775, 87.50500000000001, 87.53250000000001, 87.56000000000002, 87.5875, 87.61500000000001, 87.64250000000001, 87.67000000000002, 87.6975, 87.72500000000001, 87.75250000000001, 87.78000000000002, 87.8075, 87.83500000000001, 87.86250000000001, 87.89000000000001, 87.91750000000002, 87.94500000000001, 87.97250000000001, 88.00000000000001, 88.02750000000002, 88.055, 88.08250000000001, 88.11000000000001, 88.13750000000002, 88.165, 88.19250000000001, 88.22000000000001, 88.24750000000002, 88.275, 88.30250000000001, 88.33000000000001, 88.35750000000002, 88.385, 88.41250000000001, 88.44000000000001, 88.46750000000002, 88.495, 88.52250000000001, 88.55000000000001, 88.57750000000001, 88.60500000000002, 88.63250000000001, 88.66000000000001, 88.68750000000001, 88.71500000000002, 88.7425, 88.77000000000001, 88.79750000000001, 88.82500000000002, 88.8525, 88.88000000000001, 88.90750000000001, 88.93500000000002, 88.9625, 88.99000000000001, 89.01750000000001, 89.04500000000002, 89.0725, |

| Description | Value |
| --- | --- |
| | 89.10000000000001, 89.12750000000001, 89.15500000000002, 89.18250000000002, 89.21000000000001, 89.23750000000001, 89.26500000000001, 89.29250000000002, 89.32000000000001, 89.34750000000001, 89.37500000000001, 89.40250000000002, 89.43, 89.45750000000001, 89.48500000000001, 89.51250000000002, 89.54, 89.56750000000001, 89.59500000000001, 89.62250000000002, 89.65, 89.67750000000001, 89.70500000000001, 89.73250000000002, 89.76, 89.78750000000001, 89.81500000000001, 89.84250000000002, 89.87000000000002, 89.89750000000001, 89.92500000000001, 89.95250000000001, 89.98000000000002, 90.00750000000001, 90.03500000000001, 90.06250000000001, 90.09000000000002, 90.1175, 90.14500000000001, 90.17250000000001, 90.20000000000002, 90.2275, 90.25500000000001, 90.28250000000001, 90.31000000000002, 90.3375, 90.36500000000001, 90.39250000000001, 90.42000000000002, 90.4475, 90.47500000000001, 90.50250000000001, 90.53000000000002, 90.55750000000002, 90.58500000000001, 90.61250000000001, 90.64000000000001, 90.66750000000002, 90.69500000000001, 90.72250000000001, 90.75000000000001, 90.77750000000002, 90.805, 90.83250000000001, 90.86000000000001, 90.88750000000002, 90.915, 90.94250000000001, 90.97000000000001, 90.99750000000002, 91.025, 91.05250000000001, 91.08000000000001, 91.10750000000002, 91.135, 91.16250000000001, 91.19000000000001, 91.21750000000002, 91.24500000000002, 91.27250000000001, 91.30000000000001, 91.32750000000001, 91.35500000000002, 91.38250000000001, 91.41000000000001, 91.43750000000001, 91.46500000000002, 91.4925, 91.52000000000001, 91.54750000000001, 91.57500000000002, 91.6025, 91.63000000000001, 91.65750000000001, 91.68500000000002, 91.7125, 91.74000000000001, 91.76750000000001, 91.79500000000002, 91.8225, 91.85000000000001, 91.87750000000001, 91.90500000000002, 91.93250000000002, 91.96000000000001, 91.98750000000001, 92.01500000000001, 92.04250000000002, 92.07000000000001, 92.09750000000001, 92.12500000000001, 92.15250000000002, 92.18, 92.20750000000001, 92.23500000000001, 92.26250000000002, 92.29, 92.31750000000001, 92.34500000000001, 92.37250000000002, 92.4, 92.42750000000001, 92.45500000000001, 92.48250000000002, 92.51, 92.53750000000001, 92.56500000000001, 92.59250000000002, 92.62000000000002, 92.64750000000001, 92.67500000000001, 92.70250000000001, 92.73000000000002, 92.75750000000001, 92.78500000000001, 92.81250000000001, 92.84000000000002, 92.8675, 92.89500000000001, 92.92250000000001, 92.95000000000002, 92.9775, 93.00500000000001, 93.03250000000001, 93.06000000000002, 93.0875, 93.11500000000001, 93.14250000000001, 93.17000000000002, 93.19750000000002, 93.22500000000001, 93.25250000000001, 93.28000000000002, 93.30750000000002, 93.33500000000001, 93.36250000000001, 93.39000000000001, 93.41750000000002, 93.44500000000001, 93.47250000000001, 93.50000000000001, 93.52750000000002, 93.555, 93.58250000000001, 93.61000000000001, 93.63750000000002, 93.665, 93.69250000000001, 93.72000000000001, |

| Description | Value |
|---|---|
| | 93.74750000000002, 93.775, 93.80250000000001, 93.83000000000001, 93.85750000000002, 93.88500000000002, 93.91250000000001, 93.94000000000001, 93.96750000000002, 93.99500000000002, 94.02250000000001, 94.05000000000001, 94.07750000000001, 94.10500000000002, 94.13250000000001, 94.16000000000001, 94.18750000000001, 94.21500000000002, 94.2425, 94.27000000000001, 94.29750000000001, 94.32500000000002, 94.3525, 94.38000000000001, 94.40750000000001, 94.43500000000002, 94.4625, 94.49000000000001, 94.51750000000001, 94.54500000000002, 94.57250000000002, 94.60000000000001, 94.62750000000001, 94.65500000000002, 94.68250000000002, 94.71000000000001, 94.73750000000001, 94.76500000000001, 94.79250000000002, 94.82000000000001, 94.84750000000001, 94.87500000000001, 94.90250000000002, 94.93, 94.95750000000001, 94.98500000000001, 95.01250000000002, 95.04, 95.06750000000001, 95.09500000000001, 95.12250000000002, 95.15, 95.17750000000001, 95.20500000000001, 95.23250000000002, 95.26000000000002, 95.28750000000001, 95.31500000000001, 95.34250000000002, 95.37000000000002, 95.39750000000001, 95.42500000000001, 95.45250000000001, 95.48000000000002, 95.50750000000001, 95.53500000000001, 95.56250000000001, 95.59000000000002, 95.6175, 95.64500000000001, 95.67250000000001, 95.70000000000002, 95.7275, 95.75500000000001, 95.78250000000001, 95.81000000000002, 95.8375, 95.86500000000001, 95.89250000000001, 95.92000000000002, 95.94750000000002, 95.97500000000001, 96.00250000000001, 96.03000000000002, 96.05750000000002, 96.08500000000001, 96.11250000000001, 96.14000000000001, 96.16750000000002, 96.19500000000001, 96.22250000000001, 96.25000000000001, 96.27750000000002, 96.305, 96.33250000000001, 96.36000000000001, 96.38750000000002, 96.415, 96.44250000000001, 96.47000000000001, 96.49750000000002, 96.525, 96.55250000000001, 96.58000000000001, 96.60750000000002, 96.63500000000002, 96.66250000000001, 96.69000000000001, 96.71750000000002, 96.74500000000002, 96.77250000000001, 96.80000000000001, 96.82750000000001, 96.85500000000002, 96.88250000000001, 96.91000000000001, 96.93750000000001, 96.96500000000002, 96.9925, 97.02000000000001, 97.04750000000001, 97.07500000000002, 97.1025, 97.13000000000001, 97.15750000000001, 97.18500000000002, 97.2125, 97.24000000000001, 97.26750000000001, 97.29500000000002, 97.32250000000002, 97.35000000000001, 97.37750000000001, 97.40500000000002, 97.43250000000002, 97.46000000000001, 97.48750000000001, 97.51500000000001, 97.54250000000002, 97.57000000000001, 97.59750000000001, 97.62500000000001, 97.65250000000002, 97.68, 97.70750000000001, 97.73500000000001, 97.76250000000002, 97.79, 97.81750000000001, 97.84500000000001, 97.87250000000002, 97.90000000000002, 97.92750000000001, 97.95500000000001, 97.98250000000002, 98.01000000000002, 98.03750000000001, 98.06500000000001, 98.09250000000002, 98.12000000000002, 98.14750000000001, 98.17500000000001, |

| Description | Value |
| --- | --- |
| | 98.20250000000001, 98.23000000000002, 98.25750000000001, 98.28500000000001, 98.31250000000001, 98.34000000000002, 98.3675, 98.39500000000001, 98.42250000000001, 98.45000000000002, 98.4775, 98.50500000000001, 98.53250000000001, 98.56000000000002, 98.58750000000002, 98.61500000000001, 98.64250000000001, 98.67000000000002, 98.69750000000002, 98.72500000000001, 98.75250000000001, 98.78000000000002, 98.80750000000002, 98.83500000000001, 98.86250000000001, 98.89000000000001, 98.91750000000002, 98.94500000000001, 98.97250000000001, 99.00000000000001, 99.02750000000002, 99.055, 99.08250000000001, 99.11000000000001, 99.13750000000002, 99.165, 99.19250000000001, 99.22000000000001, 99.24750000000002, 99.27500000000002, 99.30250000000001, 99.33000000000001, 99.35750000000002, 99.38500000000002, 99.41250000000001, 99.44000000000001, 99.46750000000002, 99.49500000000002, 99.52250000000001, 99.55000000000001, 99.57750000000001, 99.60500000000002, 99.63250000000001, 99.66000000000001, 99.68750000000001, 99.71500000000002, 99.7425, 99.77000000000001, 99.79750000000001, 99.82500000000002, 99.8525, 99.88000000000001, 99.90750000000001, 99.93500000000002, 99.96250000000002, 99.99000000000001, 100.01750000000001, 100.04500000000002, 100.07250000000002, 100.10000000000001, 100.12750000000001, 100.15500000000002, 100.18250000000002, 100.21000000000001, 100.23750000000001, 100.26500000000001, 100.29250000000002, 100.32000000000001, 100.34750000000001, 100.37500000000001, 100.40250000000002, 100.43, 100.45750000000001, 100.48500000000001, 100.51250000000002, 100.54, 100.56750000000001, 100.59500000000001, 100.62250000000002, 100.65000000000002, 100.67750000000001, 100.70500000000001, 100.73250000000002, 100.76000000000002, 100.78750000000001, 100.81500000000001, 100.84250000000002, 100.87000000000002, 100.89750000000001, 100.92500000000001, 100.95250000000001, 100.98000000000002, 101.00750000000001, 101.03500000000001, 101.06250000000001, 101.09000000000002, 101.1175, 101.14500000000001, 101.17250000000001, 101.20000000000002, 101.2275, 101.25500000000001, 101.28250000000001, 101.31000000000002, 101.33750000000002, 101.36500000000001, 101.39250000000001, 101.42000000000002, 101.44750000000002, 101.47500000000001, 101.50250000000001, 101.53000000000002, 101.55750000000002, 101.58500000000001, 101.61250000000001, 101.64000000000001, 101.66750000000002, 101.69500000000001, 101.72250000000001, 101.75000000000001, 101.77750000000002, 101.805, 101.83250000000001, 101.86000000000001, 101.88750000000002, 101.91500000000002, 101.94250000000001, 101.97000000000001, 101.99750000000002, 102.02500000000002, 102.05250000000001, 102.08000000000001, 102.10750000000002, 102.13500000000002, 102.16250000000001, 102.19000000000001, 102.21750000000002, 102.24500000000002, 102.27250000000001, 102.30000000000001, 102.32750000000001, 102.35500000000002, |

| Description | Value |
| --- | --- |
| | 102.38250000000001, 102.41000000000001, 102.43750000000001, 102.46500000000002, 102.4925, 102.52000000000001, 102.54750000000001, 102.57500000000002, 102.60250000000002, 102.63000000000001, 102.65750000000001, 102.68500000000002, 102.71250000000002, 102.74000000000001, 102.76750000000001, 102.79500000000002, 102.82250000000002, 102.85000000000001, 102.87750000000001, 102.90500000000002, 102.93250000000002, 102.96000000000001, 102.98750000000001, 103.01500000000001, 103.04250000000002, 103.07000000000001, 103.09750000000001, 103.12500000000001, 103.15250000000002, 103.18, 103.20750000000001, 103.23500000000001, 103.26250000000002, 103.29000000000002, 103.31750000000001, 103.34500000000001, 103.37250000000002, 103.40000000000002, 103.42750000000001, 103.45500000000001, 103.48250000000002, 103.51000000000002, 103.53750000000001, 103.56500000000001, 103.59250000000002, 103.62000000000002, 103.64750000000001, 103.67500000000001, 103.70250000000001, 103.73000000000002, 103.75750000000001, 103.78500000000001, 103.81250000000001, 103.84000000000002, 103.8675, 103.89500000000001, 103.92250000000001, 103.95000000000002, 103.97750000000002, 104.00500000000001, 104.03250000000001, 104.06000000000002, 104.08750000000002, 104.11500000000001, 104.14250000000001, 104.17000000000002, 104.19750000000002, 104.22500000000001, 104.25250000000001, 104.28000000000002, 104.30750000000002, 104.33500000000001, 104.36250000000001, 104.39000000000001, 104.41750000000002, 104.44500000000001, 104.47250000000001, 104.50000000000001, 104.52750000000002, 104.555, 104.58250000000001, 104.61000000000001, 104.63750000000002, 104.66500000000002, 104.69250000000001, 104.72000000000001, 104.74750000000002, 104.77500000000002, 104.80250000000001, 104.83000000000001, 104.85750000000002, 104.88500000000002, 104.91250000000001, 104.94000000000001, 104.96750000000002, 104.99500000000002, 105.02250000000001, 105.05000000000001, 105.07750000000001, 105.10500000000002, 105.13250000000001, 105.16000000000001, 105.18750000000001, 105.21500000000002, 105.2425, 105.27000000000001, 105.29750000000001, 105.32500000000002, 105.35250000000002, 105.38000000000001, 105.40750000000001, 105.43500000000002, 105.46250000000002, 105.49000000000001, 105.51750000000001, 105.54500000000002, 105.57250000000002, 105.60000000000001, 105.62750000000001, 105.65500000000002, 105.68250000000002, 105.71000000000001, 105.73750000000001, 105.76500000000001, 105.79250000000002, 105.82000000000001, 105.84750000000001, 105.87500000000001, 105.90250000000002, 105.93, 105.95750000000001, 105.98500000000001, 106.01250000000002, 106.04000000000002, 106.06750000000001, 106.09500000000001, 106.12250000000002, 106.15000000000002, 106.17750000000001, 106.20500000000001, 106.23250000000002, 106.26000000000002, 106.28750000000001, 106.31500000000001, 106.34250000000002, 106.37000000000002, |

| Description | Value |
| --- | --- |
| | 106.39750000000001, 106.42500000000001, 106.45250000000001, 106.48000000000002, 106.50750000000001, 106.53500000000001, 106.56250000000001, 106.59000000000002, 106.61750000000002, 106.64500000000001, 106.67250000000001, 106.70000000000002, 106.72750000000002, 106.75500000000001, 106.78250000000001, 106.81000000000002, 106.83750000000002, 106.86500000000001, 106.89250000000001, 106.92000000000002, 106.94750000000002, 106.97500000000001, 107.00250000000001, 107.03000000000002, 107.05750000000002, 107.08500000000001, 107.11250000000001, 107.14000000000001, 107.16750000000002, 107.19500000000001, 107.22250000000001, 107.25000000000001, 107.27750000000002, 107.30500000000002, 107.33250000000001, 107.36000000000001, 107.38750000000002, 107.41500000000002, 107.44250000000001, 107.47000000000001, 107.49750000000002, 107.52500000000002, 107.55250000000001, 107.58000000000001, 107.60750000000002, 107.63500000000002, 107.66250000000001, 107.69000000000001, 107.71750000000002, 107.74500000000002, 107.77250000000001, 107.80000000000001, 107.82750000000001, 107.85500000000002, 107.88250000000001, 107.91000000000001, 107.93750000000001, 107.96500000000002, 107.99250000000002, 108.02000000000001, 108.04750000000001, 108.07500000000002, 108.10250000000002, 108.13000000000001, 108.15750000000001, 108.18500000000002, 108.21250000000002, 108.24000000000001, 108.26750000000001, 108.29500000000002, 108.32250000000002, 108.35000000000001, 108.37750000000001, 108.40500000000002, 108.43250000000002, 108.46000000000001, 108.48750000000001, 108.51500000000001, 108.54250000000002, 108.57000000000001, 108.59750000000001, 108.62500000000001, 108.65250000000002, 108.68000000000002, 108.70750000000001, 108.73500000000001, 108.76250000000002, 108.79000000000002, 108.81750000000001, 108.84500000000001, 108.87250000000002, 108.90000000000002, 108.92750000000001, 108.95500000000001, 108.98250000000002, 109.01000000000002, 109.03750000000001, 109.06500000000001, 109.09250000000002, 109.12000000000002, 109.14750000000001, 109.17500000000001, 109.20250000000001, 109.23000000000002, 109.25750000000001, 109.28500000000001, 109.31250000000001, 109.34000000000002, 109.36750000000002, 109.39500000000001, 109.42250000000001, 109.45000000000002, 109.47750000000002, 109.50500000000001, 109.53250000000001, 109.56000000000002, 109.58750000000002, 109.61500000000001, 109.64250000000001, 109.67000000000002, 109.69750000000002, 109.72500000000001, 109.75250000000001, 109.78000000000002, 109.80750000000002, 109.83500000000001, 109.86250000000001, 109.89000000000001, 109.91750000000002, 109.94500000000001, 109.97250000000001, 110.00000000000001, 110.02750000000002, 110.05500000000002, 110.08250000000001, 110.11000000000001, 110.13750000000002, 110.16500000000002, 110.19250000000001, 110.22000000000001, 110.24750000000002, 110.27500000000002, 110.30250000000001, 110.33000000000001, |

| Description | Value |
| --- | --- |
| | 110.35750000000002, 110.38500000000002, 110.41250000000001, |
| | 110.44000000000001, 110.46750000000002, 110.49500000000002, |
| | 110.52250000000001, 110.55000000000001, 110.57750000000001, |
| | 110.60500000000002, 110.63250000000002, 110.66000000000001, |
| | 110.68750000000001, 110.71500000000002, 110.74250000000002, |
| | 110.77000000000001, 110.79750000000001, 110.82500000000002, |
| | 110.85250000000002, 110.88000000000001, 110.90750000000001, |
| | 110.93500000000002, 110.96250000000002, 110.99000000000001, |
| | 111.01750000000001, 111.04500000000002, 111.07250000000002, |
| | 111.10000000000001, 111.12750000000001, 111.15500000000002, |
| | 111.18250000000002, 111.21000000000001, 111.23750000000001, |
| | 111.26500000000001, 111.29250000000002, 111.32000000000002, |
| | 111.34750000000001, 111.37500000000001, 111.40250000000002, |
| | 111.43000000000002, 111.45750000000001, 111.48500000000001, |
| | 111.51250000000002, 111.54000000000002, 111.56750000000001, |
| | 111.59500000000001, 111.62250000000002, 111.65000000000002, |
| | 111.67750000000001, 111.70500000000001, 111.73250000000002, |
| | 111.76000000000002, 111.78750000000001, 111.81500000000001, |
| | 111.84250000000002, 111.87000000000002, 111.89750000000001, |
| | 111.92500000000001, 111.95250000000001, 111.98000000000002, |
| | 112.00750000000002, 112.03500000000001, 112.06250000000001, |
| | 112.09000000000002, 112.11750000000002, 112.14500000000001, |
| | 112.17250000000001, 112.20000000000002, 112.22750000000002, |
| | 112.25500000000001, 112.28250000000001, 112.31000000000002, |
| | 112.33750000000002, 112.36500000000001, 112.39250000000001, |
| | 112.42000000000002, 112.44750000000002, 112.47500000000001, |
| | 112.50250000000001, 112.53000000000002, 112.55750000000002, |
| | 112.58500000000001, 112.61250000000001, 112.64000000000001, |
| | 112.66750000000002, 112.69500000000002, 112.72250000000001, |
| | 112.75000000000001, 112.77750000000002, 112.80500000000002, |
| | 112.83250000000001, 112.86000000000001, 112.88750000000002, |
| | 112.91500000000002, 112.94250000000001, 112.97000000000001, |
| | 112.99750000000002, 113.02500000000002, 113.05250000000001, |
| | 113.08000000000001, 113.10750000000002, 113.13500000000002, |
| | 113.16250000000001, 113.19000000000001, 113.21750000000002, |
| | 113.24500000000002, 113.27250000000001, 113.30000000000001, |
| | 113.32750000000001, 113.35500000000002, 113.38250000000002, |
| | 113.41000000000001, 113.43750000000001, 113.46500000000002, |
| | 113.49250000000002, 113.52000000000001, 113.54750000000001, |
| | 113.57500000000002, 113.60250000000002, 113.63000000000001, |
| | 113.65750000000001, 113.68500000000002, 113.71250000000002, |
| | 113.74000000000001, 113.76750000000001, 113.79500000000002, |
| | 113.82250000000002, 113.85000000000001, 113.87750000000001, |
| | 113.90500000000002, 113.93250000000002, 113.96000000000001, |
| | 113.98750000000001, 114.01500000000001, 114.04250000000002, |
| | 114.07000000000002, 114.09750000000001, 114.12500000000001, |
| | 114.15250000000002, 114.18000000000002, 114.20750000000001, |
| | 114.23500000000001, 114.26250000000002, 114.29000000000002, |

| Description | Value |
|---|---|
| | 114.31750000000001, 114.34500000000001, 114.37250000000002, 114.40000000000002, 114.42750000000001, 114.45500000000001, 114.48250000000002, 114.51000000000002, 114.53750000000001, 114.56500000000001, 114.59250000000002, 114.62000000000002, 114.64750000000001, 114.67500000000001, 114.70250000000001, 114.73000000000002, 114.75750000000002, 114.78500000000001, 114.81250000000001, 114.84000000000002, 114.86750000000002, 114.89500000000001, 114.92250000000001, 114.95000000000002, 114.97750000000002, 115.00500000000001, 115.03250000000001, 115.06000000000002, 115.08750000000002, 115.11500000000001, 115.14250000000001, 115.17000000000002, 115.19750000000002, 115.22500000000001, 115.25250000000001, 115.28000000000002, 115.30750000000002, 115.33500000000002, 115.36250000000001, 115.39000000000001, 115.41750000000002, 115.44500000000002, 115.47250000000001, 115.50000000000001, 115.52750000000002, 115.55500000000002, 115.58250000000001, 115.61000000000001, 115.63750000000002, 115.66500000000002, 115.69250000000001, 115.72000000000001, 115.74750000000002, 115.77500000000002, 115.80250000000001, 115.83000000000001, 115.85750000000002, 115.88500000000002, 115.91250000000001, 115.94000000000001, 115.96750000000002, 115.99500000000002, 116.02250000000002, 116.05000000000001, 116.07750000000001, 116.10500000000002, 116.13250000000002, 116.16000000000001, 116.18750000000001, 116.21500000000002, 116.24250000000002, 116.27000000000001, 116.29750000000001, 116.32500000000002, 116.35250000000002, 116.38000000000001, 116.40750000000001, 116.43500000000002, 116.46250000000002, 116.49000000000001, 116.51750000000001, 116.54500000000002, 116.57250000000002, 116.60000000000001, 116.62750000000001, 116.65500000000002, 116.68250000000002, 116.71000000000002, 116.73750000000001, 116.76500000000001, 116.79250000000002, 116.82000000000002, 116.84750000000001, 116.87500000000001, 116.90250000000002, 116.93000000000002, 116.95750000000001, 116.98500000000001, 117.01250000000002, 117.04000000000002, 117.06750000000001, 117.09500000000001, 117.12250000000002, 117.15000000000002, 117.17750000000001, 117.20500000000001, 117.23250000000002, 117.26000000000002, 117.28750000000001, 117.31500000000001, 117.34250000000002, 117.37000000000002, 117.39750000000002, 117.42500000000001, 117.45250000000001, 117.48000000000002, 117.50750000000002, 117.53500000000001, 117.56250000000001, 117.59000000000002, 117.61750000000002, 117.64500000000001, 117.67250000000001, 117.70000000000002, 117.72750000000002, 117.75500000000001, 117.78250000000001, 117.81000000000002, 117.83750000000002, 117.86500000000001, 117.89250000000001, 117.92000000000002, 117.94750000000002, 117.97500000000001, 118.00250000000001, 118.03000000000002, 118.05750000000002, 118.08500000000002, 118.11250000000001, 118.14000000000001, 118.16750000000002, 118.19500000000002, 118.22250000000001, 118.25000000000001, |

| Description | Value |
| --- | --- |
| | 118.27750000000002, 118.30500000000002, 118.33250000000001, 118.36000000000001, 118.38750000000002, 118.41500000000002, 118.44250000000001, 118.47000000000001, 118.49750000000002, 118.52500000000002, 118.55250000000001, 118.58000000000001, 118.60750000000002, 118.63500000000002, 118.66250000000001, 118.69000000000001, 118.71750000000002, 118.74500000000002, 118.77250000000002, 118.80000000000001, 118.82750000000001, 118.85500000000002, 118.88250000000002, 118.91000000000001, 118.93750000000001, 118.96500000000002, 118.99250000000002, 119.02000000000001, 119.04750000000001, 119.07500000000002, 119.10250000000002, 119.13000000000001, 119.15750000000001, 119.18500000000002, 119.21250000000002, 119.24000000000001, 119.26750000000001, 119.29500000000002, 119.32250000000002, 119.35000000000002, 119.37750000000001, 119.40500000000002, 119.43250000000002, 119.46000000000002, 119.48750000000001, 119.51500000000001, 119.54250000000002, 119.57000000000002, 119.59750000000001, 119.62500000000001, 119.65250000000002, 119.68000000000002, 119.70750000000001, 119.73500000000001, 119.76250000000002, 119.79000000000002, 119.81750000000001, 119.84500000000001, 119.87250000000002, 119.90000000000002, 119.92750000000001, 119.95500000000001, 119.98250000000002, 120.01000000000002, 120.03750000000002, 120.06500000000001, 120.09250000000002, 120.12000000000002, 120.14750000000002, 120.17500000000001, 120.20250000000001, 120.23000000000002, 120.25750000000002, 120.28500000000001, 120.31250000000001, 120.34000000000002, 120.36750000000002, 120.39500000000001, 120.42250000000001, 120.45000000000002, 120.47750000000002, 120.50500000000001, 120.53250000000001, 120.56000000000002, 120.58750000000002, 120.61500000000001, 120.64250000000001, 120.67000000000002, 120.69750000000002, 120.72500000000002, 120.75250000000001, 120.78000000000002, 120.80750000000002, 120.83500000000002, 120.86250000000001, 120.89000000000001, 120.91750000000002, 120.94500000000002, 120.97250000000001, 121.00000000000001, 121.02750000000002, 121.05500000000002, 121.08250000000001, 121.11000000000001, 121.13750000000002, 121.16500000000002, 121.19250000000001, 121.22000000000001, 121.24750000000002, 121.27500000000002, 121.30250000000001, 121.33000000000001, 121.35750000000002, 121.38500000000002, 121.41250000000002, 121.44000000000001, 121.46750000000002, 121.49500000000002, 121.52250000000002, 121.55000000000001, 121.57750000000001, 121.60500000000002, 121.63250000000002, 121.66000000000001, 121.68750000000001, 121.71500000000002, 121.74250000000002, 121.77000000000001, 121.79750000000001, 121.82500000000002, 121.85250000000002, 121.88000000000001, 121.90750000000001, 121.93500000000002, 121.96250000000002, 121.99000000000001, 122.01750000000001, 122.04500000000002, 122.07250000000002, 122.10000000000002, 122.12750000000001, 122.15500000000002, 122.18250000000002, 122.21000000000002, |

| Description | Value |
| --- | --- |
| | 122.23750000000001, 122.26500000000001, 122.29250000000002, 122.32000000000002, 122.34750000000001, 122.37500000000001, 122.40250000000002, 122.43000000000002, 122.45750000000001, 122.48500000000001, 122.51250000000002, 122.54000000000002, 122.56750000000001, 122.59500000000001, 122.62250000000002, 122.65000000000002, 122.67750000000001, 122.70500000000001, 122.73250000000002, 122.76000000000002, 122.78750000000002, 122.81500000000001, 122.84250000000002, 122.87000000000002, 122.89750000000002, 122.92500000000001, 122.95250000000001, 122.98000000000002, 123.00750000000002, 123.03500000000001, 123.06250000000001, 123.09000000000002, 123.11750000000002, 123.14500000000001, 123.17250000000001, 123.20000000000002, 123.22750000000002, 123.25500000000001, 123.28250000000001, 123.31000000000002, 123.33750000000002, 123.36500000000001, 123.39250000000001, 123.42000000000002, 123.44750000000002, 123.47500000000002, 123.50250000000001, 123.53000000000002, 123.55750000000002, 123.58500000000002, 123.61250000000001, 123.64000000000001, 123.66750000000002, 123.69500000000002, 123.72250000000001, 123.75000000000001, 123.77750000000002, 123.80500000000002, 123.83250000000001, 123.86000000000001, 123.88750000000002, 123.91500000000002, 123.94250000000001, 123.97000000000001, 123.99750000000002, 124.02500000000002, 124.05250000000002, 124.08000000000001, 124.10750000000002, 124.13500000000002, 124.16250000000002, 124.19000000000001, 124.21750000000002, 124.24500000000002, 124.27250000000002, 124.30000000000001, 124.32750000000001, 124.35500000000002, 124.38250000000002, 124.41000000000001, 124.43750000000001, 124.46500000000002, 124.49250000000002, 124.52000000000001, 124.54750000000001, 124.57500000000002, 124.60250000000002, 124.63000000000001, 124.65750000000001, 124.68500000000002, 124.71250000000002, 124.74000000000002, 124.76750000000001, 124.79500000000002, 124.82250000000002, 124.85000000000002, 124.87750000000001, 124.90500000000002, 124.93250000000002, 124.96000000000002, 124.98750000000001, 125.01500000000001, 125.04250000000002, 125.07000000000002, 125.09750000000001, 125.12500000000001, 125.15250000000002, 125.18000000000002, 125.20750000000001, 125.23500000000001, 125.26250000000002, 125.29000000000002, 125.31750000000001, 125.34500000000001, 125.37250000000002, 125.40000000000002, 125.42750000000002, 125.45500000000001, 125.48250000000002, 125.51000000000002, 125.53750000000002, 125.56500000000001, 125.59250000000002, 125.62000000000002, 125.64750000000002, 125.67500000000001, 125.70250000000001, 125.73000000000002, 125.75750000000002, 125.78500000000001, 125.81250000000001, 125.84000000000002, 125.86750000000002, 125.89500000000001, 125.92250000000001, 125.95000000000002, 125.97750000000002, 126.00500000000001, 126.03250000000001, 126.06000000000002, 126.08750000000002, 126.11500000000002, 126.14250000000001, 126.17000000000002, |

| Description | Value |
| --- | --- |
| | 126.19750000000002, 126.22500000000002, 126.25250000000001, 126.28000000000002, 126.30750000000002, 126.33500000000002, 126.36250000000001, 126.39000000000001, 126.41750000000002, 126.44500000000002, 126.47250000000001, 126.50000000000001, 126.52750000000002, 126.55500000000002, 126.58250000000001, 126.61000000000001, 126.63750000000002, 126.66500000000002, 126.69250000000001, 126.72000000000001, 126.74750000000002, 126.77500000000002, 126.80250000000002, 126.83000000000001, 126.85750000000002, 126.88500000000002, 126.91250000000002, 126.94000000000001, 126.96750000000002, 126.99500000000002, 127.02250000000002, 127.05000000000001, 127.07750000000001, 127.10500000000002, 127.13250000000002, 127.16000000000001, 127.18750000000001, 127.21500000000002, 127.24250000000002, 127.27000000000001, 127.29750000000001, 127.32500000000002, 127.35250000000002, 127.38000000000001, 127.40750000000001, 127.43500000000002, 127.46250000000002, 127.49000000000002, 127.51750000000001, 127.54500000000002, 127.57250000000002, 127.60000000000002, 127.62750000000001, 127.65500000000002, 127.68250000000002, 127.71000000000002, 127.73750000000001, 127.76500000000001, 127.79250000000002, 127.82000000000002, 127.84750000000001, 127.87500000000001, 127.90250000000002, 127.93000000000002, 127.95750000000001, 127.98500000000001, 128.01250000000002, 128.04000000000002, 128.06750000000002, 128.09500000000003, 128.12250000000003, 128.15, 128.1775, 128.205, 128.23250000000002, 128.26000000000002, 128.28750000000002, 128.31500000000003, 128.34250000000003, 128.37, 128.3975, 128.425, 128.45250000000001, 128.48000000000002, 128.50750000000002, 128.53500000000003, 128.56250000000003, 128.59, 128.6175, 128.645, 128.6725, 128.70000000000002, 128.72750000000002, 128.75500000000002, 128.78250000000003, 128.81000000000003, 128.8375, 128.865, 128.8925, 128.92000000000002, 128.94750000000002, 128.97500000000002, 129.00250000000003, 129.03000000000003, 129.0575, 129.085, 129.1125, 129.14000000000001, 129.16750000000002, 129.19500000000002, 129.22250000000003, 129.25000000000003, 129.2775, 129.305, 129.3325, 129.36, 129.38750000000002, 129.41500000000002, 129.44250000000002, 129.47000000000003, 129.49750000000003, 129.525, 129.5525, 129.58, 129.60750000000002, 129.63500000000002, 129.66250000000002, 129.69000000000003, 129.71750000000003, 129.745, 129.7725, 129.8, 129.82750000000001, 129.85500000000002, 129.88250000000002, 129.91000000000003, 129.93750000000003, 129.965, 129.9925, 130.02, 130.0475, 130.07500000000002, 130.10250000000002, 130.13000000000002, 130.15750000000003, 130.18500000000003, 130.2125, 130.24, 130.2675, 130.29500000000002, 130.32250000000002, 130.35000000000002, 130.37750000000003, 130.40500000000003, 130.4325, 130.46, 130.4875, 130.51500000000001, 130.54250000000002, 130.57000000000002, 130.59750000000003, 130.62500000000003, 130.6525, 130.68, 130.7075, 130.735, 130.76250000000002, 130.79000000000002, |

| Description | Value |
| --- | --- |
| | 130.81750000000002, 130.84500000000003, 130.87250000000003, 130.9, 130.9275, 130.955, 130.98250000000002, 131.01000000000002, 131.03750000000002, 131.06500000000003, 131.09250000000003, 131.12, 131.1475, 131.175, 131.20250000000001, 131.23000000000002, 131.25750000000002, 131.28500000000003, 131.31250000000003, 131.34, 131.3675, 131.395, 131.4225, 131.45000000000002, 131.47750000000002, 131.50500000000002, 131.53250000000003, 131.56000000000003, 131.5875, 131.615, 131.6425, 131.67000000000002, 131.69750000000002, 131.72500000000002, 131.75250000000003, 131.78000000000003, 131.8075, 131.835, 131.8625, 131.89000000000001, 131.91750000000002, 131.94500000000002, 131.97250000000003, 132.00000000000003, 132.0275, 132.055, 132.0825, 132.11, 132.13750000000002, 132.16500000000002, 132.19250000000002, 132.22000000000003, 132.24750000000003, 132.275, 132.3025, 132.33, 132.35750000000002, 132.38500000000002, 132.41250000000002, 132.44000000000003, 132.46750000000003, 132.495, 132.5225, 132.55, 132.57750000000001, 132.60500000000002, 132.63250000000002, 132.66000000000003, 132.68750000000003, 132.715, 132.7425, 132.77, 132.7975, 132.82500000000002, 132.85250000000002, 132.88000000000002, 132.90750000000003, 132.93500000000003, 132.9625, 132.99, 133.0175, 133.04500000000002, 133.07250000000002, 133.10000000000002, 133.12750000000003, 133.15500000000003, 133.1825, 133.21, 133.2375, 133.26500000000001, 133.29250000000002, 133.32000000000002, 133.34750000000003, 133.37500000000003, 133.4025, 133.43, 133.4575, 133.485, 133.51250000000002, 133.54000000000002, 133.56750000000002, 133.59500000000003, 133.62250000000003, 133.65, 133.6775, 133.705, 133.73250000000002, 133.76000000000002, 133.78750000000002, 133.81500000000003, 133.84250000000003, 133.87, 133.8975, 133.925, 133.95250000000001, 133.98000000000002, 134.00750000000002, 134.03500000000003, 134.06250000000003, 134.09, 134.1175, 134.145, 134.1725, 134.20000000000002, 134.22750000000002, 134.25500000000002, 134.28250000000003, 134.31000000000003, 134.3375, 134.365, 134.3925, 134.42000000000002, 134.44750000000002, 134.47500000000002, 134.50250000000003, 134.53000000000003, 134.5575, 134.585, 134.6125, 134.64000000000001, 134.66750000000002, 134.69500000000002, 134.72250000000003, 134.75000000000003, 134.77750000000003, 134.805, 134.8325, 134.86, 134.88750000000002, 134.91500000000002, 134.94250000000002, 134.97000000000003, 134.99750000000003, 135.025, 135.0525, 135.08, 135.10750000000002, 135.13500000000002, 135.16250000000002, 135.19000000000003, 135.21750000000003, 135.245, 135.2725, 135.3, 135.32750000000001, 135.35500000000002, 135.38250000000002, 135.41000000000003, 135.43750000000003, 135.46500000000003, 135.4925, 135.52, 135.5475, 135.57500000000002, 135.60250000000002, 135.63000000000002, 135.65750000000003, 135.68500000000003, 135.7125, 135.74, 135.7675, 135.79500000000002, 135.82250000000002, 135.85000000000002, 135.87750000000003, 135.90500000000003, 135.9325, 135.96, 135.9875, 136.01500000000001, 136.04250000000002, 136.07000000000002, |

| Description | Value |
| --- | --- |
| | 136.09750000000003, 136.12500000000003, 136.15250000000003, 136.18, 136.2075, 136.235, 136.26250000000002, 136.29000000000002, 136.31750000000002, 136.34500000000003, 136.37250000000003, 136.4, 136.4275, 136.455, 136.48250000000002, 136.51000000000002, 136.53750000000002, 136.56500000000003, 136.59250000000003, 136.62, 136.6475, 136.675, 136.70250000000001, 136.73000000000002, 136.75750000000002, 136.78500000000003, 136.81250000000003, 136.84000000000003, 136.8675, 136.895, 136.9225, 136.95000000000002, 136.97750000000002, 137.00500000000002, 137.03250000000003, 137.06000000000003, 137.0875, 137.115, 137.1425, 137.17000000000002, 137.19750000000002, 137.22500000000002, 137.25250000000003, 137.28000000000003, 137.3075, 137.335, 137.3625, 137.39000000000001, 137.41750000000002, 137.44500000000002, 137.47250000000003, 137.50000000000003, 137.52750000000003, 137.555, 137.5825, 137.61, 137.63750000000002, 137.66500000000002, 137.69250000000002, 137.72000000000003, 137.74750000000003, 137.775, 137.8025, 137.83, 137.85750000000002, 137.88500000000002, 137.91250000000002, 137.94000000000003, 137.96750000000003, 137.995, 138.0225, 138.05, 138.07750000000001, 138.10500000000002, 138.13250000000002, 138.16000000000003, 138.18750000000003, 138.21500000000003, 138.2425, 138.27, 138.2975, 138.32500000000002, 138.35250000000002, 138.38000000000002, 138.40750000000003, 138.43500000000003, 138.4625, 138.49, 138.5175, 138.54500000000002, 138.57250000000002, 138.60000000000002, 138.62750000000003, 138.65500000000003, 138.6825, 138.71, 138.7375, 138.76500000000001, 138.79250000000002, 138.82000000000002, 138.84750000000003, 138.87500000000003, 138.90250000000003, 138.93, 138.9575, 138.985, 139.01250000000002, 139.04000000000002, 139.06750000000002, 139.09500000000003, 139.12250000000003, 139.15, 139.1775, 139.205, 139.23250000000002, 139.26000000000002, 139.28750000000002, 139.31500000000003, 139.34250000000003, 139.37, 139.3975, 139.425, 139.45250000000001, 139.48000000000002, 139.50750000000002, 139.53500000000003, 139.56250000000003, 139.59000000000003, 139.6175, 139.645, 139.6725, 139.70000000000002, 139.72750000000002, 139.75500000000002, 139.78250000000003, 139.81000000000003, 139.8375, 139.865, 139.8925, 139.92000000000002, 139.94750000000002, 139.97500000000002, 140.00250000000003, 140.03000000000003, 140.0575, 140.085, 140.1125, 140.14000000000001, 140.16750000000002, 140.19500000000002, 140.22250000000003, 140.25000000000003, 140.27750000000003, 140.305, 140.3325, 140.36, 140.38750000000002, 140.41500000000002, 140.44250000000002, 140.47000000000003, 140.49750000000003, 140.525, 140.5525, 140.58, 140.60750000000002, 140.63500000000002, 140.66250000000002, 140.69000000000003, 140.71750000000003, 140.745, 140.7725, 140.8, 140.82750000000001, 140.85500000000002, 140.88250000000002, 140.91000000000003, 140.93750000000003, 140.96500000000003, 140.9925, 141.02, 141.0475, 141.07500000000002, 141.10250000000002, 141.13000000000002, 141.15750000000003, 141.18500000000003, 141.2125, 141.24, 141.2675, 141.29500000000002, |

| Description | Value |
|---|---|
| | 141.32250000000002, 141.35000000000002, 141.37750000000003, 141.40500000000003, 141.4325, 141.46, 141.4875, 141.51500000000001, 141.54250000000002, 141.57000000000002, 141.59750000000003, 141.62500000000003, 141.65250000000003, 141.68, 141.7075, 141.735, 141.76250000000002, 141.79000000000002, 141.81750000000002, 141.84500000000003, 141.87250000000003, 141.9, 141.9275, 141.955, 141.98250000000002, 142.01000000000002, 142.03750000000002, 142.06500000000003, 142.09250000000003, 142.12, 142.1475, 142.175, 142.20250000000001, 142.23000000000002, 142.25750000000002, 142.28500000000003, 142.31250000000003, 142.34000000000003, 142.3675, 142.395, 142.4225, 142.45000000000002, 142.47750000000002, 142.50500000000002, 142.53250000000003, 142.56000000000003, 142.5875, 142.615, 142.6425, 142.67000000000002, 142.69750000000002, 142.72500000000002, 142.75250000000003, 142.78000000000003, 142.8075, 142.835, 142.8625, 142.89000000000001, 142.91750000000002, 142.94500000000002, 142.97250000000003, 143.00000000000003, 143.02750000000003, 143.055, 143.0825, 143.11, 143.13750000000002, 143.16500000000002, 143.19250000000002, 143.22000000000003, 143.24750000000003, 143.275, 143.3025, 143.33, 143.35750000000002, 143.38500000000002, 143.41250000000002, 143.44000000000003, 143.46750000000003, 143.49500000000003, 143.5225, 143.55, 143.57750000000001, 143.60500000000002, 143.63250000000002, 143.66000000000003, 143.68750000000003, 143.71500000000003, 143.7425, 143.77, 143.7975, 143.82500000000002, 143.85250000000002, 143.88000000000002, 143.90750000000003, 143.93500000000003, 143.9625, 143.99, 144.0175, 144.04500000000002, 144.07250000000002, 144.10000000000002, 144.12750000000003, 144.15500000000003, 144.18250000000003, 144.21, 144.2375, 144.26500000000001, 144.29250000000002, 144.32000000000002, 144.34750000000003, 144.37500000000003, 144.40250000000003, 144.43, 144.4575, 144.485, 144.51250000000002, 144.54000000000002, 144.56750000000002, 144.59500000000003, 144.62250000000003, 144.65, 144.6775, 144.705, 144.73250000000002, 144.76000000000002, 144.78750000000002, 144.81500000000003, 144.84250000000003, 144.87000000000003, 144.8975, 144.925, 144.95250000000001, 144.98000000000002, 145.00750000000002, 145.03500000000003, 145.06250000000003, 145.09000000000003, 145.1175, 145.145, 145.1725, 145.20000000000002, 145.22750000000002, 145.25500000000002, 145.28250000000003, 145.31000000000003, 145.3375, 145.365, 145.3925, 145.42000000000002, 145.44750000000002, 145.47500000000002, 145.50250000000003, 145.53000000000003, 145.55750000000003, 145.585, 145.6125, 145.64000000000001, 145.66750000000002, 145.69500000000002, 145.72250000000003, 145.75000000000003, 145.77750000000003, 145.805, 145.8325, 145.86, 145.88750000000002, 145.91500000000002, 145.94250000000002, 145.97000000000003, 145.99750000000003, 146.025, 146.0525, 146.08, 146.10750000000002, 146.13500000000002, 146.16250000000002, 146.19000000000003, 146.21750000000003, 146.24500000000003, 146.2725, 146.3, 146.32750000000001, |

| Description | Value |
| --- | --- |
| | 146.35500000000002, 146.38250000000002, 146.41000000000003, 146.43750000000003, 146.46500000000003, 146.4925, 146.52, 146.5475, 146.57500000000002, 146.60250000000002, 146.63000000000002, 146.65750000000003, 146.68500000000003, 146.7125, 146.74, 146.7675, 146.79500000000002, 146.82250000000002, 146.85000000000002, 146.87750000000003, 146.90500000000003, 146.93250000000003, 146.96, 146.9875, 147.01500000000001, 147.04250000000002, 147.07000000000002, 147.09750000000003, 147.12500000000003, 147.15250000000003, 147.18, 147.2075, 147.235, 147.26250000000002, 147.29000000000002, 147.31750000000002, 147.34500000000003, 147.37250000000003, 147.4, 147.4275, 147.455, 147.48250000000002, 147.51000000000002, 147.53750000000002, 147.56500000000003, 147.59250000000003, 147.62000000000003, 147.6475, 147.675, 147.70250000000001, 147.73000000000002, 147.75750000000002, 147.78500000000003, 147.81250000000003, 147.84000000000003, 147.8675, 147.895, 147.9225, 147.95000000000002, 147.97750000000002, 148.00500000000002, 148.03250000000003, 148.06000000000003, 148.0875, 148.115, 148.1425, 148.17000000000002, 148.19750000000002, 148.22500000000002, 148.25250000000003, 148.28000000000003, 148.30750000000003, 148.335, 148.3625, 148.39000000000001, 148.41750000000002, 148.44500000000002, 148.47250000000003, 148.50000000000003, 148.52750000000003, 148.555, 148.5825, 148.61, 148.63750000000002, 148.66500000000002, 148.69250000000002, 148.72000000000003, 148.74750000000003, 148.775, 148.8025, 148.83, 148.85750000000002, 148.88500000000002, 148.91250000000002, 148.94000000000003, 148.96750000000003, 148.99500000000003, 149.0225, 149.05, 149.07750000000001, 149.10500000000002, 149.13250000000002, 149.16000000000003, 149.18750000000003, 149.21500000000003, 149.2425, 149.27, 149.2975, 149.32500000000002, 149.35250000000002, 149.38000000000002, 149.40750000000003, 149.43500000000003, 149.4625, 149.49, 149.5175, 149.54500000000002, 149.57250000000002, 149.60000000000002, 149.62750000000003, 149.65500000000003, 149.68250000000003, 149.71, 149.7375, 149.76500000000001, 149.79250000000002, 149.82000000000002, 149.84750000000003, 149.87500000000003, 149.90250000000003, 149.93, 149.9575, 149.985, 150.01250000000002, 150.04000000000002, 150.06750000000002, 150.09500000000003, 150.12250000000003, 150.15, 150.1775, 150.205, 150.23250000000002, 150.26000000000002, 150.28750000000002, 150.31500000000003, 150.34250000000003, 150.37000000000003, 150.3975, 150.425, 150.45250000000001, 150.48000000000002, 150.50750000000002, 150.53500000000003, 150.56250000000003, 150.59000000000003, 150.6175, 150.645, 150.6725, 150.70000000000002, 150.72750000000002, 150.75500000000002, 150.78250000000003, 150.81000000000003, 150.8375, 150.865, 150.8925, 150.92000000000002, 150.94750000000002, 150.97500000000002, 151.00250000000003, 151.03000000000003, 151.05750000000003, 151.085, 151.1125, 151.14000000000001, 151.16750000000002, 151.19500000000002, 151.22250000000003, 151.25000000000003, |

| Description | Value |
| --- | --- |
| | 151.27750000000003, 151.305, 151.3325, 151.36, 151.38750000000002, 151.41500000000002, 151.44250000000002, 151.47000000000003, 151.49750000000003, 151.525, 151.5525, 151.58, 151.60750000000002, 151.63500000000002, 151.66250000000002, 151.69000000000003, 151.71750000000003, 151.74500000000003, 151.7725, 151.8, 151.82750000000001, 151.85500000000002, 151.88250000000002, 151.91000000000003, 151.93750000000003, 151.96500000000003, 151.9925, 152.02, 152.0475, 152.07500000000002, 152.10250000000002, 152.13000000000002, 152.15750000000003, 152.18500000000003, 152.21250000000003, 152.24, 152.2675, 152.29500000000002, 152.32250000000002, 152.35000000000002, 152.37750000000003, 152.40500000000003, 152.43250000000003, 152.46, 152.4875, 152.51500000000001, 152.54250000000002, 152.57000000000002, 152.59750000000003, 152.62500000000003, 152.65250000000003, 152.68, 152.7075, 152.735, 152.76250000000002, 152.79000000000002, 152.81750000000002, 152.84500000000003, 152.87250000000003, 152.90000000000003, 152.9275, 152.955, 152.98250000000002, 153.01000000000002, 153.03750000000002, 153.06500000000003, 153.09250000000003, 153.12000000000003, 153.1475, 153.175, 153.20250000000001, 153.23000000000002, 153.25750000000002, 153.28500000000003, 153.31250000000003, 153.34000000000003, 153.3675, 153.395, 153.4225, 153.45000000000002, 153.47750000000002, 153.50500000000002, 153.53250000000003, 153.56000000000003, 153.58750000000003, 153.615, 153.6425, 153.67000000000002, 153.69750000000002, 153.72500000000002, 153.75250000000003, 153.78000000000003, 153.80750000000003, 153.835, 153.8625, 153.89000000000001, 153.91750000000002, 153.94500000000002, 153.97250000000003, 154.00000000000003, 154.02750000000003, 154.055, 154.0825, 154.11, 154.13750000000002, 154.16500000000002, 154.19250000000002, 154.22000000000003, 154.24750000000003, 154.27500000000003, 154.3025, 154.33, 154.35750000000002, 154.38500000000002, 154.41250000000002, 154.44000000000003, 154.46750000000003, 154.49500000000003, 154.5225, 154.55, 154.57750000000001, 154.60500000000002, 154.63250000000002, 154.66000000000003, 154.68750000000003, 154.71500000000003, 154.7425, 154.77, 154.7975, 154.82500000000002, 154.85250000000002, 154.88000000000002, 154.90750000000003, 154.93500000000003, 154.96250000000003, 154.99, 155.0175, 155.04500000000002, 155.07250000000002, 155.10000000000002, 155.12750000000003, 155.15500000000003, 155.18250000000003, 155.21, 155.2375, 155.26500000000001, 155.29250000000002, 155.32000000000002, 155.34750000000003, 155.37500000000003, 155.40250000000003, 155.43, 155.4575, 155.485, 155.51250000000002, 155.54000000000002, 155.56750000000002, 155.59500000000003, 155.62250000000003, 155.65000000000003, 155.6775, 155.705, 155.73250000000002, 155.76000000000002, 155.78750000000002, 155.81500000000003, 155.84250000000003, 155.87000000000003, 155.8975, 155.925, 155.95250000000001, 155.98000000000002, 156.00750000000002, |

| Description | Value |
| --- | --- |
| | 156.03500000000003, 156.06250000000003, 156.09000000000003, 156.1175, 156.145, 156.1725, 156.20000000000002, 156.22750000000002, 156.25500000000002, 156.28250000000003, 156.31000000000003, 156.33750000000003, 156.365, 156.3925, 156.42000000000002, 156.44750000000002, 156.47500000000002, 156.50250000000003, 156.53000000000003, 156.55750000000003, 156.585, 156.6125, 156.64000000000001, 156.66750000000002, 156.69500000000002, 156.72250000000003, 156.75000000000003, 156.77750000000003, 156.805, 156.8325, 156.86, 156.88750000000002, 156.91500000000002, 156.94250000000002, 156.97000000000003, 156.99750000000003, 157.02500000000003, 157.0525, 157.08, 157.10750000000002, 157.13500000000002, 157.16250000000002, 157.19000000000003, 157.21750000000003, 157.24500000000003, 157.2725, 157.3, 157.32750000000001, 157.35500000000002, 157.38250000000002, 157.41000000000003, 157.43750000000003, 157.46500000000003, 157.4925, 157.52, 157.5475, 157.57500000000002, 157.60250000000002, 157.63000000000002, 157.65750000000003, 157.68500000000003, 157.71250000000003, 157.74, 157.7675, 157.79500000000002, 157.82250000000002, 157.85000000000002, 157.87750000000003, 157.90500000000003, 157.93250000000003, 157.96, 157.9875, 158.01500000000001, 158.04250000000002, 158.07000000000002, 158.09750000000003, 158.12500000000003, 158.15250000000003, 158.18, 158.2075, 158.235, 158.26250000000002, 158.29000000000002, 158.31750000000002, 158.34500000000003, 158.37250000000003, 158.40000000000003, 158.4275, 158.455, 158.48250000000002, 158.51000000000002, 158.53750000000002, 158.56500000000003, 158.59250000000003, 158.62000000000003, 158.6475, 158.675, 158.70250000000001, 158.73000000000002, 158.75750000000002, 158.78500000000003, 158.81250000000003, 158.84000000000003, 158.8675, 158.895, 158.9225, 158.95000000000002, 158.97750000000002, 159.00500000000002, 159.03250000000003, 159.06000000000003, 159.08750000000003, 159.115, 159.1425, 159.17000000000002, 159.19750000000002, 159.22500000000002, 159.25250000000003, 159.28000000000003, 159.30750000000003, 159.335, 159.3625, 159.39000000000001, 159.41750000000002, 159.44500000000002, 159.47250000000003, 159.50000000000003, 159.52750000000003, 159.555, 159.5825, 159.61, 159.63750000000002, 159.66500000000002, 159.69250000000002, 159.72000000000003, 159.74750000000003, 159.77500000000003, 159.8025, 159.83, 159.85750000000002, 159.88500000000002, 159.91250000000002, 159.94000000000003, 159.96750000000003, 159.99500000000003, 160.0225, 160.05, 160.07750000000001, 160.10500000000002, 160.13250000000002, 160.16000000000003, 160.18750000000003, 160.21500000000003, 160.2425, 160.27, 160.2975, 160.32500000000002, 160.35250000000002, 160.38000000000002, 160.40750000000003, 160.43500000000003, 160.46250000000003, 160.49, 160.5175, 160.54500000000002, 160.57250000000002, 160.60000000000002, 160.62750000000003, 160.65500000000003, 160.68250000000003, 160.71, 160.7375, |

| Description | Value |
| --- | --- |
| | 160.76500000000001, 160.79250000000002, 160.82000000000002, 160.84750000000003, 160.87500000000003, 160.90250000000003, 160.93000000000004, 160.9575, 160.985, 161.01250000000002, 161.04000000000002, 161.06750000000002, 161.09500000000003, 161.12250000000003, 161.15000000000003, 161.1775, 161.205, 161.23250000000002, 161.26000000000002, 161.28750000000002, 161.31500000000003, 161.34250000000003, 161.37000000000003, 161.3975, 161.425, 161.45250000000001, 161.48000000000002, 161.50750000000002, 161.53500000000003, 161.56250000000003, 161.59000000000003, 161.61750000000004, 161.645, 161.6725, 161.70000000000002, 161.72750000000002, 161.75500000000002, 161.78250000000003, 161.81000000000003, 161.83750000000003, 161.865, 161.8925, 161.92000000000002, 161.94750000000002, 161.97500000000002, 162.00250000000003, 162.03000000000003, 162.05750000000003, 162.085, 162.1125, 162.14000000000001, 162.16750000000002, 162.19500000000002, 162.22250000000003, 162.25000000000003, 162.27750000000003, 162.30500000000004, 162.3325, 162.36, 162.38750000000002, 162.41500000000002, 162.44250000000002, 162.47000000000003, 162.49750000000003, 162.52500000000003, 162.5525, 162.58, 162.60750000000002, 162.63500000000002, 162.66250000000002, 162.69000000000003, 162.71750000000003, 162.74500000000003, 162.7725, 162.8, 162.82750000000001, 162.85500000000002, 162.88250000000002, 162.91000000000003, 162.93750000000003, 162.96500000000003, 162.99250000000004, 163.02, 163.0475, 163.07500000000002, 163.10250000000002, 163.13000000000002, 163.15750000000003, 163.18500000000003, 163.21250000000003, 163.24, 163.2675, 163.29500000000002, 163.32250000000002, 163.35000000000002, 163.37750000000003, 163.40500000000003, 163.43250000000003, 163.46, 163.4875, 163.51500000000001, 163.54250000000002, 163.57000000000002, 163.59750000000003, 163.62500000000003, 163.65250000000003, 163.68000000000004, 163.7075, 163.735, 163.76250000000002, 163.79000000000002, 163.81750000000002, 163.84500000000003, 163.87250000000003, 163.90000000000003, 163.9275, 163.955, 163.98250000000002, 164.01000000000002, 164.03750000000002, 164.06500000000003, 164.09250000000003, 164.12000000000003, 164.1475, 164.175, 164.20250000000001, 164.23000000000002, 164.25750000000002, 164.28500000000003, 164.31250000000003, 164.34000000000003, 164.36750000000004, 164.395, 164.4225, 164.45000000000002, 164.47750000000002, 164.50500000000002, 164.53250000000003, 164.56000000000003, 164.58750000000003, 164.615, 164.6425, 164.67000000000002, 164.69750000000002, 164.72500000000002, 164.75250000000003, 164.78000000000003, 164.80750000000003, 164.835, 164.8625, 164.89000000000001, 164.91750000000002, 164.94500000000002, 164.97250000000003, 165.00000000000003, 165.02750000000003, 165.05500000000004, 165.0825, 165.11, 165.13750000000002, 165.16500000000002, 165.19250000000002, 165.22000000000003, |

| Description | Value |
|---|---|
| | 165.24750000000003, 165.27500000000003, 165.3025, 165.33, 165.35750000000002, 165.38500000000002, 165.41250000000002, 165.44000000000003, 165.46750000000003, 165.49500000000003, 165.5225, 165.55, 165.57750000000001, 165.60500000000002, 165.63250000000002, 165.66000000000003, 165.68750000000003, 165.71500000000003, 165.74250000000004, 165.77, 165.7975, 165.82500000000002, 165.85250000000002, 165.88000000000002, 165.90750000000003, 165.93500000000003, 165.96250000000003, 165.99, 166.0175, 166.04500000000002, 166.07250000000002, 166.10000000000002, 166.12750000000003, 166.15500000000003, 166.18250000000003, 166.21, 166.2375, 166.26500000000001, 166.29250000000002, 166.32000000000002, 166.34750000000003, 166.37500000000003, 166.40250000000003, 166.43000000000004, 166.4575, 166.485, 166.51250000000002, 166.54000000000002, 166.56750000000002, 166.59500000000003, 166.62250000000003, 166.65000000000003, 166.6775, 166.705, 166.73250000000002, 166.76000000000002, 166.78750000000002, 166.81500000000003, 166.84250000000003, 166.87000000000003, 166.8975, 166.925, 166.95250000000001, 166.98000000000002, 167.00750000000002, 167.03500000000003, 167.06250000000003, 167.09000000000003, 167.11750000000004, 167.145, 167.1725, 167.20000000000002, 167.22750000000002, 167.25500000000002, 167.28250000000003, 167.31000000000003, 167.33750000000003, 167.365, 167.3925, 167.42000000000002, 167.44750000000002, 167.47500000000002, 167.50250000000003, 167.53000000000003, 167.55750000000003, 167.585, 167.6125, 167.64000000000001, 167.66750000000002, 167.69500000000002, 167.72250000000003, 167.75000000000003, 167.77750000000003, 167.80500000000004, 167.8325, 167.86, 167.88750000000002, 167.91500000000002, 167.94250000000002, 167.97000000000003, 167.99750000000003, 168.02500000000003, 168.0525, 168.08, 168.10750000000002, 168.13500000000002, 168.16250000000002, 168.19000000000003, 168.21750000000003, 168.24500000000003, 168.2725, 168.3, 168.32750000000001, 168.35500000000002, 168.38250000000002, 168.41000000000003, 168.43750000000003, 168.46500000000003, 168.49250000000004, 168.52, 168.5475, 168.57500000000002, 168.60250000000002, 168.63000000000002, 168.65750000000003, 168.68500000000003, 168.71250000000003, 168.74, 168.7675, 168.79500000000002, 168.82250000000002, 168.85000000000002, 168.87750000000003, 168.90500000000003, 168.93250000000003, 168.96000000000004, 168.9875, 169.01500000000001, 169.04250000000002, 169.07000000000002, 169.09750000000003, 169.12500000000003, 169.15250000000003, 169.18000000000004, 169.2075, 169.235, 169.26250000000002, 169.29000000000002, 169.31750000000002, 169.34500000000003, 169.37250000000003, 169.40000000000003, 169.4275, 169.455, 169.48250000000002, 169.51000000000002, 169.53750000000002, 169.56500000000003, 169.59250000000003, 169.62000000000003, 169.64750000000004, 169.675, |

| Description | Value |
| --- | --- |
| | 169.70250000000001, 169.73000000000002, 169.75750000000002, 169.78500000000003, 169.81250000000003, 169.84000000000003, 169.86750000000004, 169.895, 169.9225, 169.95000000000002, 169.97750000000002, 170.00500000000002, 170.03250000000003, 170.06000000000003, 170.08750000000003, 170.115, 170.1425, 170.17000000000002, 170.19750000000002, 170.22500000000002, 170.25250000000003, 170.28000000000003, 170.30750000000003, 170.33500000000004, 170.3625, 170.39000000000001, 170.41750000000002, 170.44500000000002, 170.47250000000003, 170.50000000000003, 170.52750000000003, 170.55500000000004, 170.5825, 170.61, 170.63750000000002, 170.66500000000002, 170.69250000000002, 170.72000000000003, 170.74750000000003, 170.77500000000003, 170.8025, 170.83, 170.85750000000002, 170.88500000000002, 170.91250000000002, 170.94000000000003, 170.96750000000003, 170.99500000000003, 171.02250000000004, 171.05, 171.07750000000001, 171.10500000000002, 171.13250000000002, 171.16000000000003, 171.18750000000003, 171.21500000000003, 171.24250000000004, 171.27, 171.2975, 171.32500000000002, 171.35250000000002, 171.38000000000002, 171.40750000000003, 171.43500000000003, 171.46250000000003, 171.49, 171.5175, 171.54500000000002, 171.57250000000002, 171.60000000000002, 171.62750000000003, 171.65500000000003, 171.68250000000003, 171.71000000000004, 171.7375, 171.76500000000001, 171.79250000000002, 171.82000000000002, 171.84750000000003, 171.87500000000003, 171.90250000000003, 171.93000000000004, 171.9575, 171.985, 172.01250000000002, 172.04000000000002, 172.06750000000002, 172.09500000000003, 172.12250000000003, 172.15000000000003, 172.1775, 172.205, 172.23250000000002, 172.26000000000002, 172.28750000000002, 172.31500000000003, 172.34250000000003, 172.37000000000003, 172.39750000000004, 172.425, 172.45250000000001, 172.48000000000002, 172.50750000000002, 172.53500000000003, 172.56250000000003, 172.59000000000003, 172.61750000000004, 172.645, 172.6725, 172.70000000000002, 172.72750000000002, 172.75500000000002, 172.78250000000003, 172.81000000000003, 172.83750000000003, 172.865, 172.8925, 172.92000000000002, 172.94750000000002, 172.97500000000002, 173.00250000000003, 173.03000000000003, 173.05750000000003, 173.08500000000004, 173.1125, 173.14000000000001, 173.16750000000002, 173.19500000000002, 173.22250000000003, 173.25000000000003, 173.27750000000003, 173.30500000000004, 173.3325, 173.36, 173.38750000000002, 173.41500000000002, 173.44250000000002, 173.47000000000003, 173.49750000000003, 173.52500000000003, 173.5525, 173.58, 173.60750000000002, 173.63500000000002, 173.66250000000002, 173.69000000000003, 173.71750000000003, 173.74500000000003, 173.77250000000004, 173.8, 173.82750000000001, 173.85500000000002, 173.88250000000002, 173.91000000000003, 173.93750000000003, 173.96500000000003, 173.99250000000004, 174.02, 174.0475, |

| Description | Value |
| --- | --- |
| | 174.07500000000002, 174.10250000000002, 174.13000000000002, 174.15750000000003, 174.18500000000003, 174.21250000000003, 174.24, 174.2675, 174.29500000000002, 174.32250000000002, 174.35000000000002, 174.37750000000003, 174.40500000000003, 174.43250000000003, 174.46000000000004, 174.4875, 174.51500000000001, 174.54250000000002, 174.57000000000002, 174.59750000000003, 174.62500000000003, 174.65250000000003, 174.68000000000004, 174.7075, 174.735, 174.76250000000002, 174.79000000000002, 174.81750000000002, 174.84500000000003, 174.87250000000003, 174.90000000000003, 174.9275, 174.955, 174.98250000000002, 175.01000000000002, 175.03750000000002, 175.06500000000003, 175.09250000000003, 175.12000000000003, 175.14750000000004, 175.175, 175.20250000000001, 175.23000000000002, 175.25750000000002, 175.28500000000003, 175.31250000000003, 175.34000000000003, 175.36750000000004, 175.395, 175.4225, 175.45000000000002, 175.47750000000002, 175.50500000000002, 175.53250000000003, 175.56000000000003, 175.58750000000003, 175.615, 175.6425, 175.67000000000002, 175.69750000000002, 175.72500000000002, 175.75250000000003, 175.78000000000003, 175.80750000000003, 175.83500000000004, 175.8625, 175.89000000000001, 175.91750000000002, 175.94500000000002, 175.97250000000003, 176.00000000000003, 176.02750000000003, 176.05500000000004, 176.0825, 176.11, 176.13750000000002, 176.16500000000002, 176.19250000000002, 176.22000000000003, 176.24750000000003, 176.27500000000003, 176.3025, 176.33, 176.35750000000002, 176.38500000000002, 176.41250000000002, 176.44000000000003, 176.46750000000003, 176.49500000000003, 176.52250000000004, 176.55, 176.57750000000001, 176.60500000000002, 176.63250000000002, 176.66000000000003, 176.68750000000003, 176.71500000000003, 176.74250000000004, 176.77, 176.7975, 176.82500000000002, 176.85250000000002, 176.88000000000002, 176.90750000000003, 176.93500000000003, 176.96250000000003, 176.99, 177.0175, 177.04500000000002, 177.07250000000002, 177.10000000000002, 177.12750000000003, 177.15500000000003, 177.18250000000003, 177.21000000000004, 177.2375, 177.26500000000001, 177.29250000000002, 177.32000000000002, 177.34750000000003, 177.37500000000003, 177.40250000000003, 177.43000000000004, 177.4575, 177.485, 177.51250000000002, 177.54000000000002, 177.56750000000002, 177.59500000000003, 177.62250000000003, 177.65000000000003, 177.67750000000004, 177.705, 177.73250000000002, 177.76000000000002, 177.78750000000002, 177.81500000000003, 177.84250000000003, 177.87000000000003, 177.89750000000004, 177.925, 177.95250000000001, 177.98000000000002, 178.00750000000002, 178.03500000000003, 178.06250000000003, 178.09000000000003, 178.11750000000004, 178.145, 178.1725, 178.20000000000002, 178.22750000000002, 178.25500000000002, 178.28250000000003, 178.31000000000003, 178.33750000000003, |

| Description | Value |
| --- | --- |
| | 178.36500000000004, 178.3925, 178.42000000000002,<br>178.44750000000002, 178.47500000000002, 178.50250000000003,<br>178.53000000000003, 178.55750000000003, 178.58500000000004,<br>178.6125, 178.64000000000001, 178.66750000000002,<br>178.69500000000002, 178.72250000000003, 178.75000000000003,<br>178.77750000000003, 178.80500000000004, 178.8325, 178.86,<br>178.88750000000002, 178.91500000000002, 178.94250000000002,<br>178.97000000000003, 178.99750000000003, 179.02500000000003,<br>179.05250000000004, 179.08, 179.10750000000002, 179.13500000000002,<br>179.16250000000002, 179.19000000000003, 179.21750000000003,<br>179.24500000000003, 179.27250000000004, 179.3, 179.32750000000001,<br>179.35500000000002, 179.38250000000002, 179.41000000000003,<br>179.43750000000003, 179.46500000000003, 179.49250000000004, 179.52,<br>179.5475, 179.57500000000002, 179.60250000000002,<br>179.63000000000002, 179.65750000000003, 179.68500000000003,<br>179.71250000000003, 179.74000000000004, 179.7675,<br>179.79500000000002, 179.82250000000002, 179.85000000000002,<br>179.87750000000003, 179.90500000000003, 179.93250000000003,<br>179.96000000000004, 179.9875, 180.01500000000001,<br>180.04250000000002, 180.07000000000002, 180.09750000000003,<br>180.12500000000003, 180.15250000000003, 180.18000000000004,<br>180.2075, 180.235, 180.26250000000002, 180.29000000000002,<br>180.31750000000002, 180.34500000000003, 180.37250000000003,<br>180.40000000000003, 180.42750000000004, 180.455,<br>180.48250000000002, 180.51000000000002, 180.53750000000002,<br>180.56500000000003, 180.59250000000003, 180.62000000000003,<br>180.64750000000004, 180.675, 180.70250000000001,<br>180.73000000000002, 180.75750000000002, 180.78500000000003,<br>180.81250000000003, 180.84000000000003, 180.86750000000004,<br>180.895, 180.9225, 180.95000000000002, 180.97750000000002,<br>181.00500000000002, 181.03250000000003, 181.06000000000003,<br>181.08750000000003, 181.11500000000004, 181.1425,<br>181.17000000000002, 181.19750000000002, 181.22500000000002,<br>181.25250000000003, 181.28000000000003, 181.30750000000003,<br>181.33500000000004, 181.3625, 181.39000000000001,<br>181.41750000000002, 181.44500000000002, 181.47250000000003,<br>181.50000000000003, 181.52750000000003, 181.55500000000004,<br>181.5825, 181.61, 181.63750000000002, 181.66500000000002,<br>181.69250000000002, 181.72000000000003, 181.74750000000003,<br>181.77500000000003, 181.80250000000004, 181.83, 181.85750000000002,<br>181.88500000000002, 181.91250000000002, 181.94000000000003,<br>181.96750000000003, 181.99500000000003, 182.02250000000004, 182.05,<br>182.07750000000001, 182.10500000000002, 182.13250000000002,<br>182.16000000000003, 182.18750000000003, 182.21500000000003,<br>182.24250000000004, 182.27, 182.2975, 182.32500000000002,<br>182.35250000000002, 182.38000000000002, 182.40750000000003,<br>182.43500000000003, 182.46250000000003, 182.49000000000004,<br>182.5175, 182.54500000000002, 182.57250000000002, |

| Description | Value |
| --- | --- |
| | 182.60000000000002, 182.62750000000003, 182.65500000000003, 182.68250000000003, 182.71000000000004, 182.7375, 182.76500000000001, 182.79250000000002, 182.82000000000002, 182.84750000000003, 182.87500000000003, 182.90250000000003, 182.93000000000004, 182.9575, 182.985, 183.01250000000002, 183.04000000000002, 183.06750000000002, 183.09500000000003, 183.12250000000003, 183.15000000000003, 183.17750000000004, 183.205, 183.23250000000002, 183.26000000000002, 183.28750000000002, 183.31500000000003, 183.34250000000003, 183.37000000000003, 183.39750000000004, 183.425, 183.45250000000001, 183.48000000000002, 183.50750000000002, 183.53500000000003, 183.56250000000003, 183.59000000000003, 183.61750000000004, 183.645, 183.6725, 183.70000000000002, 183.72750000000002, 183.75500000000002, 183.78250000000003, 183.81000000000003, 183.83750000000003, 183.86500000000004, 183.8925, 183.92000000000002, 183.94750000000002, 183.97500000000002, 184.00250000000003, 184.03000000000003, 184.05750000000003, 184.08500000000004, 184.1125, 184.14000000000001, 184.16750000000002, 184.19500000000002, 184.22250000000003, 184.25000000000003, 184.27750000000003, 184.30500000000004, 184.3325, 184.36, 184.38750000000002, 184.41500000000002, 184.44250000000002, 184.47000000000003, 184.49750000000003, 184.52500000000003, 184.55250000000004, 184.58, 184.60750000000002, 184.63500000000002, 184.66250000000002, 184.69000000000003, 184.71750000000003, 184.74500000000003, 184.77250000000004, 184.8, 184.82750000000001, 184.85500000000002, 184.88250000000002, 184.91000000000003, 184.93750000000003, 184.96500000000003, 184.99250000000004, 185.02, 185.0475, 185.07500000000002, 185.10250000000002, 185.13000000000002, 185.15750000000003, 185.18500000000003, 185.21250000000003, 185.24000000000004, 185.2675, 185.29500000000002, 185.32250000000002, 185.35000000000002, 185.37750000000003, 185.40500000000003, 185.43250000000003, 185.46000000000004, 185.4875, 185.51500000000001, 185.54250000000002, 185.57000000000002, 185.59750000000003, 185.62500000000003, 185.65250000000003, 185.68000000000004, 185.7075, 185.735, 185.76250000000002, 185.79000000000002, 185.81750000000002, 185.84500000000003, 185.87250000000003, 185.90000000000003, 185.92750000000004, 185.955, 185.98250000000002, 186.01000000000002, 186.03750000000002, 186.06500000000003, 186.09250000000003, 186.12000000000003, 186.14750000000004, 186.175, 186.20250000000001, 186.23000000000002, 186.25750000000002, 186.28500000000003, 186.31250000000003, 186.34000000000003, 186.36750000000004, 186.39500000000004, 186.4225, 186.45000000000002, 186.47750000000002, 186.50500000000002, 186.53250000000003, 186.56000000000003, 186.58750000000003, 186.61500000000004, 186.6425, 186.67000000000002, 186.69750000000002, 186.72500000000002, |

| Description | Value |
| --- | --- |
| | 186.75250000000003, 186.78000000000003, 186.80750000000003, 186.83500000000004, 186.8625, 186.89000000000001, 186.91750000000002, 186.94500000000002, 186.97250000000003, 187.00000000000003, 187.02750000000003, 187.05500000000004, 187.08250000000004, 187.11, 187.13750000000002, 187.16500000000002, 187.19250000000002, 187.22000000000003, 187.24750000000003, 187.27500000000003, 187.30250000000004, 187.33, 187.35750000000002, 187.38500000000002, 187.41250000000002, 187.44000000000003, 187.46750000000003, 187.49500000000003, 187.52250000000004, 187.55, 187.57750000000001, 187.60500000000002, 187.63250000000002, 187.66000000000003, 187.68750000000003, 187.71500000000003, 187.74250000000004, 187.77000000000004, 187.7975, 187.82500000000002, 187.85250000000002, 187.88000000000002, 187.90750000000003, 187.93500000000003, 187.96250000000003, 187.99000000000004, 188.0175, 188.04500000000002, 188.07250000000002, 188.10000000000002, 188.12750000000003, 188.15500000000003, 188.18250000000003, 188.21000000000004, 188.2375, 188.26500000000001, 188.29250000000002, 188.32000000000002, 188.34750000000003, 188.37500000000003, 188.40250000000003, 188.43000000000004, 188.45750000000004, 188.485, 188.51250000000002, 188.54000000000002, 188.56750000000002, 188.59500000000003, 188.62250000000003, 188.65000000000003, 188.67750000000004, 188.705, 188.73250000000002, 188.76000000000002, 188.78750000000002, 188.81500000000003, 188.84250000000003, 188.87000000000003, 188.89750000000004, 188.925, 188.95250000000001, 188.98000000000002, 189.00750000000002, 189.03500000000003, 189.06250000000003, 189.09000000000003, 189.11750000000004, 189.14500000000004, 189.1725, 189.20000000000002, 189.22750000000002, 189.25500000000002, 189.28250000000003, 189.31000000000003, 189.33750000000003, 189.36500000000004, 189.3925, 189.42000000000002, 189.44750000000002, 189.47500000000002, 189.50250000000003, 189.53000000000003, 189.55750000000003, 189.58500000000004, 189.6125, 189.64000000000001, 189.66750000000002, 189.69500000000002, 189.72250000000003, 189.75000000000003, 189.77750000000003, 189.80500000000004, 189.83250000000004, 189.86, 189.88750000000002, 189.91500000000002, 189.94250000000002, 189.97000000000003, 189.99750000000003, 190.02500000000003, 190.05250000000004, 190.08, 190.10750000000002, 190.13500000000002, 190.16250000000002, 190.19000000000003, 190.21750000000003, 190.24500000000003, 190.27250000000004, 190.3, 190.32750000000001, 190.35500000000002, 190.38250000000002, 190.41000000000003, 190.43750000000003, 190.46500000000003, 190.49250000000004, 190.52000000000004, 190.5475, 190.57500000000002, 190.60250000000002, 190.63000000000002, 190.65750000000003, 190.68500000000003, 190.71250000000003, 190.74000000000004, 190.7675, 190.79500000000002, 190.82250000000002, 190.85000000000002, |

| Description | Value |
| --- | --- |
| | 190.87750000000003, 190.90500000000003, 190.93250000000003, 190.96000000000004, 190.9875, 191.01500000000001, 191.04250000000002, 191.07000000000002, 191.09750000000003, 191.12500000000003, 191.15250000000003, 191.18000000000004, 191.20750000000004, 191.235, 191.26250000000002, 191.29000000000002, 191.31750000000002, 191.34500000000003, 191.37250000000003, 191.40000000000003, 191.42750000000004, 191.455, 191.48250000000002, 191.51000000000002, 191.53750000000002, 191.56500000000003, 191.59250000000003, 191.62000000000003, 191.64750000000004, 191.675, 191.70250000000001, 191.73000000000002, 191.75750000000002, 191.78500000000003, 191.81250000000003, 191.84000000000003, 191.86750000000004, 191.89500000000004, 191.9225, 191.95000000000002, 191.97750000000002, 192.00500000000002, 192.03250000000003, 192.06000000000003, 192.08750000000003, 192.11500000000004, 192.1425, 192.17000000000002, 192.19750000000002, 192.22500000000002, 192.25250000000003, 192.28000000000003, 192.30750000000003, 192.33500000000004, 192.3625, 192.39000000000001, 192.41750000000002, 192.44500000000002, 192.47250000000003, 192.50000000000003, 192.52750000000003, 192.55500000000004, 192.58250000000004, 192.61, 192.63750000000002, 192.66500000000002, 192.69250000000002, 192.72000000000003, 192.74750000000003, 192.77500000000003, 192.80250000000004, 192.83, 192.85750000000002, 192.88500000000002, 192.91250000000002, 192.94000000000003, 192.96750000000003, 192.99500000000003, 193.02250000000004, 193.05, 193.07750000000001, 193.10500000000002, 193.13250000000002, 193.16000000000003, 193.18750000000003, 193.21500000000003, 193.24250000000004, 193.27000000000004, 193.2975, 193.32500000000002, 193.35250000000002, 193.38000000000002, 193.40750000000003, 193.43500000000003, 193.46250000000003, 193.49000000000004, 193.5175, 193.54500000000002, 193.57250000000002, 193.60000000000002, 193.62750000000003, 193.65500000000003, 193.68250000000003, 193.71000000000004, 193.7375, 193.76500000000001, 193.79250000000002, 193.82000000000002, 193.84750000000003, 193.87500000000003, 193.90250000000003, 193.93000000000004, 193.95750000000004, 193.985, 194.01250000000002, 194.04000000000002, 194.06750000000002, 194.09500000000003, 194.12250000000003, 194.15000000000003, 194.17750000000004, 194.205, 194.23250000000002, 194.26000000000002, 194.28750000000002, 194.31500000000003, 194.34250000000003, 194.37000000000003, 194.39750000000004, 194.425, 194.45250000000001, 194.48000000000002, 194.50750000000002, 194.53500000000003, 194.56250000000003, 194.59000000000003, 194.61750000000004, 194.64500000000004, 194.6725, 194.70000000000002, 194.72750000000002, 194.75500000000002, 194.78250000000003, 194.81000000000003, 194.83750000000003, 194.86500000000004, 194.8925, |

| Description | Value |
| --- | --- |
| | 194.92000000000002, 194.94750000000002, 194.97500000000002,<br>195.00250000000003, 195.03000000000003, 195.05750000000003,<br>195.08500000000004, 195.11250000000004, 195.14000000000001,<br>195.16750000000002, 195.19500000000002, 195.22250000000003,<br>195.25000000000003, 195.27750000000003, 195.30500000000004,<br>195.33250000000004, 195.36, 195.38750000000002, 195.41500000000002,<br>195.44250000000002, 195.47000000000003, 195.49750000000003,<br>195.52500000000003, 195.55250000000004, 195.58, 195.60750000000002,<br>195.63500000000002, 195.66250000000002, 195.69000000000003,<br>195.71750000000003, 195.74500000000003, 195.77250000000004,<br>195.80000000000004, 195.82750000000001, 195.85500000000002,<br>195.88250000000002, 195.91000000000003, 195.93750000000003,<br>195.96500000000003, 195.99250000000004, 196.02000000000004,<br>196.0475, 196.07500000000002, 196.10250000000002,<br>196.13000000000002, 196.15750000000003, 196.18500000000003,<br>196.21250000000003, 196.24000000000004, 196.2675,<br>196.29500000000002, 196.32250000000002, 196.35000000000002,<br>196.37750000000003, 196.40500000000003, 196.43250000000003,<br>196.46000000000004, 196.48750000000004, 196.51500000000001,<br>196.54250000000002, 196.57000000000002, 196.59750000000003,<br>196.62500000000003, 196.65250000000003, 196.68000000000004,<br>196.70750000000004, 196.735, 196.76250000000002,<br>196.79000000000002, 196.81750000000002, 196.84500000000003,<br>196.87250000000003, 196.90000000000003, 196.92750000000004,<br>196.955, 196.98250000000002, 197.01000000000002,<br>197.03750000000002, 197.06500000000003, 197.09250000000003,<br>197.12000000000003, 197.14750000000004, 197.17500000000004,<br>197.20250000000001, 197.23000000000002, 197.25750000000002,<br>197.28500000000003, 197.31250000000003, 197.34000000000003,<br>197.36750000000004, 197.39500000000004, 197.4225,<br>197.45000000000002, 197.47750000000002, 197.50500000000002,<br>197.53250000000003, 197.56000000000003, 197.58750000000003,<br>197.61500000000004, 197.6425, 197.67000000000002,<br>197.69750000000002, 197.72500000000002, 197.75250000000003,<br>197.78000000000003, 197.80750000000003, 197.83500000000004,<br>197.86250000000004, 197.89000000000001, 197.91750000000002,<br>197.94500000000002, 197.97250000000003, 198.00000000000003,<br>198.02750000000003, 198.05500000000004, 198.08250000000004, 198.11,<br>198.13750000000002, 198.16500000000002, 198.19250000000002,<br>198.22000000000003, 198.24750000000003, 198.27500000000003,<br>198.30250000000004, 198.33, 198.35750000000002, 198.38500000000002,<br>198.41250000000002, 198.44000000000003, 198.46750000000003,<br>198.49500000000003, 198.52250000000004, 198.55000000000004,<br>198.57750000000001, 198.60500000000002, 198.63250000000002,<br>198.66000000000003, 198.68750000000003, 198.71500000000003,<br>198.74250000000004, 198.77000000000004, 198.7975,<br>198.82500000000002, 198.85250000000002, 198.88000000000002,<br>198.90750000000003, 198.93500000000003, 198.96250000000003, |

| Description | Value |
| --- | --- |
| | 198.99000000000004, 199.0175, 199.04500000000002, 199.07250000000002, 199.10000000000002, 199.12750000000003, 199.15500000000003, 199.18250000000003, 199.21000000000004, 199.23750000000004, 199.26500000000001, 199.29250000000002, 199.32000000000002, 199.34750000000003, 199.37500000000003, 199.40250000000003, 199.43000000000004, 199.45750000000004, 199.485, 199.51250000000002, 199.54000000000002, 199.56750000000002, 199.59500000000003, 199.62250000000003, 199.65000000000003, 199.67750000000004, 199.705, 199.73250000000002, 199.76000000000002, 199.78750000000002, 199.81500000000003, 199.84250000000003, 199.87000000000003, 199.89750000000004, 199.92500000000004, 199.95250000000001, 199.98000000000002, 200.00750000000002, 200.03500000000003, 200.06250000000003, 200.09000000000003, 200.11750000000004, 200.14500000000004, 200.1725, 200.20000000000002, 200.22750000000002, 200.25500000000002, 200.28250000000003, 200.31000000000003, 200.33750000000003, 200.36500000000004, 200.3925, 200.42000000000002, 200.44750000000002, 200.47500000000002, 200.50250000000003, 200.53000000000003, 200.55750000000003, 200.58500000000004, 200.61250000000004, 200.64000000000001, 200.66750000000002, 200.69500000000002, 200.72250000000003, 200.75000000000003, 200.77750000000003, 200.80500000000004, 200.83250000000004, 200.86, 200.88750000000002, 200.91500000000002, 200.94250000000002, 200.97000000000003, 200.99750000000003, 201.02500000000003, 201.05250000000004, 201.08, 201.10750000000002, 201.13500000000002, 201.16250000000002, 201.19000000000003, 201.21750000000003, 201.24500000000003, 201.27250000000004, 201.30000000000004, 201.32750000000001, 201.35500000000002, 201.38250000000002, 201.41000000000003, 201.43750000000003, 201.46500000000003, 201.49250000000004, 201.52000000000004, 201.5475, 201.57500000000002, 201.60250000000002, 201.63000000000002, 201.65750000000003, 201.68500000000003, 201.71250000000003, 201.74000000000004, 201.7675, 201.79500000000002, 201.82250000000002, 201.85000000000002, 201.87750000000003, 201.90500000000003, 201.93250000000003, 201.96000000000004, 201.98750000000004, 202.01500000000001, 202.04250000000002, 202.07000000000002, 202.09750000000003, 202.12500000000003, 202.15250000000003, 202.18000000000004, 202.20750000000004, 202.235, 202.26250000000002, 202.29000000000002, 202.31750000000002, 202.34500000000003, 202.37250000000003, 202.40000000000003, 202.42750000000004, 202.455, 202.48250000000002, 202.51000000000002, 202.53750000000002, 202.56500000000003, 202.59250000000003, 202.62000000000003, 202.64750000000004, 202.67500000000004, 202.70250000000001, 202.73000000000002, 202.75750000000002, 202.78500000000003, 202.81250000000003, 202.84000000000003, 202.86750000000004, 202.89500000000004, 202.9225, 202.95000000000002, 202.97750000000002, |

| Description | Value |
| --- | --- |
| | 203.00500000000002, 203.03250000000003, 203.06000000000003, 203.08750000000003, 203.11500000000004, 203.1425, 203.17000000000002, 203.19750000000002, 203.22500000000002, 203.25250000000003, 203.28000000000003, 203.30750000000003, 203.33500000000004, 203.36250000000004, 203.39000000000001, 203.41750000000002, 203.44500000000002, 203.47250000000003, 203.50000000000003, 203.52750000000003, 203.55500000000004, 203.58250000000004, 203.61, 203.63750000000002, 203.66500000000002, 203.69250000000002, 203.72000000000003, 203.74750000000003, 203.77500000000003, 203.80250000000004, 203.83000000000004, 203.85750000000002, 203.88500000000002, 203.91250000000002, 203.94000000000003, 203.96750000000003, 203.99500000000003, 204.02250000000004, 204.05000000000004, 204.07750000000001, 204.10500000000002, 204.13250000000002, 204.16000000000003, 204.18750000000003, 204.21500000000003, 204.24250000000004, 204.27000000000004, 204.2975, 204.32500000000002, 204.35250000000002, 204.38000000000002, 204.40750000000003, 204.43500000000003, 204.46250000000003, 204.49000000000004, 204.51750000000004, 204.54500000000002, 204.57250000000002, 204.60000000000002, 204.62750000000003, 204.65500000000003, 204.68250000000003, 204.71000000000004, 204.73750000000004, 204.76500000000001, 204.79250000000002, 204.82000000000002, 204.84750000000003, 204.87500000000003, 204.90250000000003, 204.93000000000004, 204.95750000000004, 204.985, 205.01250000000002, 205.04000000000002, 205.06750000000002, 205.09500000000003, 205.12250000000003, 205.15000000000003, 205.17750000000004, 205.20500000000004, 205.23250000000002, 205.26000000000002, 205.28750000000002, 205.31500000000003, 205.34250000000003, 205.37000000000003, 205.39750000000004, 205.42500000000004, 205.45250000000001, 205.48000000000002, 205.50750000000002, 205.53500000000003, 205.56250000000003, 205.59000000000003, 205.61750000000004, 205.64500000000004, 205.6725, 205.70000000000002, 205.72750000000002, 205.75500000000002, 205.78250000000003, 205.81000000000003, 205.83750000000003, 205.86500000000004, 205.89250000000004, 205.92000000000002, 205.94750000000002, 205.97500000000002, 206.00250000000003, 206.03000000000003, 206.05750000000003, 206.08500000000004, 206.11250000000004, 206.14000000000001, 206.16750000000002, 206.19500000000002, 206.22250000000003, 206.25000000000003, 206.27750000000003, 206.30500000000004, 206.33250000000004, 206.36, 206.38750000000002, 206.41500000000002, 206.44250000000002, 206.47000000000003, 206.49750000000003, 206.52500000000003, 206.55250000000004, 206.58000000000004, 206.60750000000002, 206.63500000000002, 206.66250000000002, 206.69000000000003, 206.71750000000003, 206.74500000000003, 206.77250000000004, 206.80000000000004, 206.82750000000001, 206.85500000000002, 206.88250000000002, 206.91000000000003, 206.93750000000003, 206.96500000000003, 206.99250000000004, |

| Description | Value |
|---|---|
| | 207.02000000000004, 207.0475, 207.07500000000002, 207.10250000000002, 207.13000000000002, 207.15750000000003, 207.18500000000003, 207.21250000000003, 207.24000000000004, 207.26750000000004, 207.29500000000002, 207.32250000000002, 207.35000000000002, 207.37750000000003, 207.40500000000003, 207.43250000000003, 207.46000000000004, 207.48750000000004, 207.51500000000001, 207.54250000000002, 207.57000000000002, 207.59750000000003, 207.62500000000003, 207.65250000000003, 207.68000000000004, 207.70750000000004, 207.735, 207.76250000000002, 207.79000000000002, 207.81750000000002, 207.84500000000003, 207.87250000000003, 207.90000000000003, 207.92750000000004, 207.95500000000004, 207.98250000000002, 208.01000000000002, 208.03750000000002, 208.06500000000003, 208.09250000000003, 208.12000000000003, 208.14750000000004, 208.17500000000004, 208.20250000000001, 208.23000000000002, 208.25750000000002, 208.28500000000003, 208.31250000000003, 208.34000000000003, 208.36750000000004, 208.39500000000004, 208.4225, 208.45000000000002, 208.47750000000002, 208.50500000000002, 208.53250000000003, 208.56000000000003, 208.58750000000003, 208.61500000000004, 208.64250000000004, 208.67000000000002, 208.69750000000002, 208.72500000000002, 208.75250000000003, 208.78000000000003, 208.80750000000003, 208.83500000000004, 208.86250000000004, 208.89000000000001, 208.91750000000002, 208.94500000000002, 208.97250000000003, 209.00000000000003, 209.02750000000003, 209.05500000000004, 209.08250000000004, 209.11, 209.13750000000002, 209.16500000000002, 209.19250000000002, 209.22000000000003, 209.24750000000003, 209.27500000000003, 209.30250000000004, 209.33000000000004, 209.35750000000002, 209.38500000000002, 209.41250000000002, 209.44000000000003, 209.46750000000003, 209.49500000000003, 209.52250000000004, 209.55000000000004, 209.57750000000001, 209.60500000000002, 209.63250000000002, 209.66000000000003, 209.68750000000003, 209.71500000000003, 209.74250000000004, 209.77000000000004, 209.7975, 209.82500000000002, 209.85250000000002, 209.88000000000002, 209.90750000000003, 209.93500000000003, 209.96250000000003, 209.99000000000004, 210.01750000000004, 210.04500000000002, 210.07250000000002, 210.10000000000002, 210.12750000000003, 210.15500000000003, 210.18250000000003, 210.21000000000004, 210.23750000000004, 210.26500000000001, 210.29250000000002, 210.32000000000002, 210.34750000000003, 210.37500000000003, 210.40250000000003, 210.43000000000004, 210.45750000000004, 210.485, 210.51250000000002, 210.54000000000002, 210.56750000000002, 210.59500000000003, 210.62250000000003, 210.65000000000003, 210.67750000000004, 210.70500000000004, 210.73250000000002, 210.76000000000002, 210.78750000000002, 210.81500000000003, 210.84250000000003, 210.87000000000003, 210.89750000000004, 210.92500000000004, 210.95250000000001, 210.98000000000002, |

| Description | Value |
| --- | --- |
| | 211.00750000000002, 211.03500000000003, 211.06250000000003, 211.09000000000003, 211.11750000000004, 211.14500000000004, 211.1725, 211.20000000000002, 211.22750000000002, 211.25500000000002, 211.28250000000003, 211.31000000000003, 211.33750000000003, 211.36500000000004, 211.39250000000004, 211.42000000000002, 211.44750000000002, 211.47500000000002, 211.50250000000003, 211.53000000000003, 211.55750000000003, 211.58500000000004, 211.61250000000004, 211.64000000000001, 211.66750000000002, 211.69500000000002, 211.72250000000003, 211.75000000000003, 211.77750000000003, 211.80500000000004, 211.83250000000004, 211.86, 211.88750000000002, 211.91500000000002, 211.94250000000002, 211.97000000000003, 211.99750000000003, 212.02500000000003, 212.05250000000004, 212.08000000000004, 212.10750000000002, 212.13500000000002, 212.16250000000002, 212.19000000000003, 212.21750000000003, 212.24500000000003, 212.27250000000004, 212.30000000000004, 212.32750000000001, 212.35500000000002, 212.38250000000002, 212.41000000000003, 212.43750000000003, 212.46500000000003, 212.49250000000004, 212.52000000000004, 212.54750000000004, 212.57500000000002, 212.60250000000002, 212.63000000000002, 212.65750000000003, 212.68500000000003, 212.71250000000003, 212.74000000000004, 212.76750000000004, 212.79500000000002, 212.82250000000002, 212.85000000000002, 212.87750000000003, 212.90500000000003, 212.93250000000003, 212.96000000000004, 212.98750000000004, 213.01500000000001, 213.04250000000002, 213.07000000000002, 213.09750000000003, 213.12500000000003, 213.15250000000003, 213.18000000000004, 213.20750000000004, 213.23500000000004, 213.26250000000002, 213.29000000000002, 213.31750000000002, 213.34500000000003, 213.37250000000003, 213.40000000000003, 213.42750000000004, 213.45500000000004, 213.48250000000002, 213.51000000000002, 213.53750000000002, 213.56500000000003, 213.59250000000003, 213.62000000000003, 213.64750000000004, 213.67500000000004, 213.70250000000001, 213.73000000000002, 213.75750000000002, 213.78500000000003, 213.81250000000003, 213.84000000000003, 213.86750000000004, 213.89500000000004, 213.92250000000004, 213.95000000000002, 213.97750000000002, 214.00500000000002, 214.03250000000003, 214.06000000000003, 214.08750000000003, 214.11500000000004, 214.14250000000004, 214.17000000000002, 214.19750000000002, 214.22500000000002, 214.25250000000003, 214.28000000000003, 214.30750000000003, 214.33500000000004, 214.36250000000004, 214.39000000000001, 214.41750000000002, 214.44500000000002, 214.47250000000003, 214.50000000000003} |
| Relative tolerance | 0.005 |

### TIME STEPPING

| Description | Value |
| --- | --- |

| Description | Value |
| --- | --- |
| Maximum BDF order | 2 |
| Nonlinear controller | On |

LOG

| | | | | | | | | | | |
|---|---|---|---|---|---|---|---|---|---|---|
| - | 202.26 | - out | | | | | | | | |
| - | 202.29 | - out | | | | | | | | |
| 2243 | 202.3 | 0.055119 | 34 | 18 | 34 | 2 | 0 | 0 | 2.1e-14 | 5.3e-15 |
| - | 202.32 | - out | | | | | | | | |
| - | 202.35 | - out | | | | | | | | |
| 2244 | 202.36 | 0.055119 | 36 | 19 | 36 | 2 | 0 | 0 | 8.1e-14 | 4.3e-15 |
| - | 202.37 | - out | | | | | | | | |
| - | 202.4 | - out | | | | | | | | |
| 2245 | 202.41 | 0.055119 | 38 | 20 | 38 | 2 | 0 | 0 | 4.1e-14 | 2.9e-15 |
| - | 202.43 | - out | | | | | | | | |
| - | 202.46 | - out | | | | | | | | |
| 2246 | 202.47 | 0.055119 | 40 | 21 | 40 | 2 | 0 | 0 | 2.8e-13 | 4.2e-15 |
| - | 202.48 | - out | | | | | | | | |
| - | 202.51 | - out | | | | | | | | |
| 2247 | 202.52 | 0.055119 | 42 | 22 | 42 | 2 | 0 | 0 | 3.8e-14 | 4e-15 |
| - | 202.54 | - out | | | | | | | | |
| - | 202.57 | - out | | | | | | | | |
| 2248 | 202.58 | 0.055119 | 44 | 23 | 44 | 2 | 0 | 0 | 2.4e-13 | 4.3e-15 |
| - | 202.59 | - out | | | | | | | | |
| - | 202.62 | - out | | | | | | | | |
| 2249 | 202.63 | 0.055119 | 46 | 24 | 46 | 2 | 0 | 0 | 4.6e-13 | 4.6e-15 |
| - | 202.65 | - out | | | | | | | | |
| - | 202.68 | - out | | | | | | | | |
| 2250 | 202.69 | 0.055119 | 48 | 25 | 48 | 2 | 0 | 0 | 7.4e-13 | 5.5e-15 |
| - | 202.7 | - out | | | | | | | | |
| - | 202.73 | - out | | | | | | | | |
| 2251 | 202.74 | 0.055119 | 50 | 26 | 50 | 2 | 0 | 0 | 4.8e-13 | 3.1e-15 |
| - | 202.76 | - out | | | | | | | | |
| - | 202.79 | - out | | | | | | | | |
| 2252 | 202.8 | 0.055119 | 52 | 27 | 52 | 2 | 0 | 0 | 2.1e-13 | 4.1e-15 |
| - | 202.81 | - out | | | | | | | | |
| - | 202.84 | - out | | | | | | | | |
| 2253 | 202.85 | 0.055119 | 54 | 28 | 54 | 2 | 0 | 0 | 6.7e-13 | 5.2e-15 |
| - | 202.87 | - out | | | | | | | | |
| - | 202.9 | - out | | | | | | | | |
| - | 202.92 | - out | | | | | | | | |
| - | 202.95 | - out | | | | | | | | |
| 2254 | 202.96 | 0.11024 | 56 | 29 | 56 | 2 | 0 | 0 | 9.4e-13 | 3.3e-15 |
| - | 202.98 | - out | | | | | | | | |
| - | 203.01 | - out | | | | | | | | |
| - | 203.03 | - out | | | | | | | | |
| - | 203.06 | - out | | | | | | | | |
| 2255 | 203.07 | 0.11024 | 58 | 30 | 58 | 2 | 0 | 0 | 2.2e-13 | 3.4e-15 |
| - | 203.09 | - out | | | | | | | | |
| - | 203.12 | - out | | | | | | | | |
| - | 203.14 | - out | | | | | | | | |
| - | 203.17 | - out | | | | | | | | |
| 2256 | 203.18 | 0.11024 | 60 | 31 | 60 | 2 | 0 | 0 | 2.5e-12 | 6.2e-15 |
| - | 203.2 | - out | | | | | | | | |
| - | 203.23 | - out | | | | | | | | |
| - | 203.25 | - out | | | | | | | | |
| - | 203.28 | - out | | | | | | | | |
| 2257 | 203.29 | 0.11024 | 62 | 32 | 62 | 2 | 0 | 0 | 3.9e-12 | 5.2e-15 |
| - | 203.31 | - out | | | | | | | | |
| - | 203.34 | - out | | | | | | | | |
| - | 203.36 | - out | | | | | | | | |

| | | | | | | | | | | |
|---|---|---|---|---|---|---|---|---|---|---|
| - | 203.39 | - | out | | | | | | | |
| 2258 | 203.4 | 0.11024 | | 64 | 33 | 64 | 2 | 0 | 0 | 7.3e-13 | 4.7e-15 |
| - | 203.42 | - | out | | | | | | | |
| - | 203.45 | - | out | | | | | | | |
| - | 203.47 | - | out | | | | | | | |
| - | 203.5 | - | out | | | | | | | |
| - | 203.53 | - | out | | | | | | | |
| - | 203.56 | - | out | | | | | | | |
| - | 203.58 | - | out | | | | | | | |
| - | 203.61 | - | out | | | | | | | |
| 2259 | 203.63 | 0.22048 | | 66 | 34 | 66 | 2 | 0 | 0 | 4.4e-14 | 8.4e-15 |
| - | 203.64 | - | out | | | | | | | |
| - | 203.67 | - | out | | | | | | | |
| - | 203.69 | - | out | | | | | | | |
| - | 203.72 | - | out | | | | | | | |
| - | 203.75 | - | out | | | | | | | |
| - | 203.78 | - | out | | | | | | | |
| - | 203.8 | - | out | | | | | | | |
| - | 203.83 | - | out | | | | | | | |
| 2260 | 203.85 | 0.22048 | | 68 | 35 | 68 | 2 | 0 | 0 | 1e-12 | 3.8e-15 |
| - | 203.86 | - | out | | | | | | | |
| - | 203.89 | - | out | | | | | | | |
| - | 203.91 | - | out | | | | | | | |
| - | 203.94 | - | out | | | | | | | |
| - | 203.97 | - | out | | | | | | | |
| 2261 | 203.99 | 0.14225 | | 72 | 37 | 72 | 2 | 1 | 0 | 6.8e-13 | 5.6e-15 |
| - | 204 | - | out | | | | | | | |
| - | 204.02 | - | out | | | | | | | |
| - | 204.05 | - | out | | | | | | | |
| - | 204.08 | - | out | | | | | | | |
| 2262 | 204.08 | 0.093143 | | 76 | 39 | 76 | 2 | 2 | 0 | 2.1e-13 | 3.3e-15 |
| - | 204.11 | - | out | | | | | | | |
| - | 204.13 | - | out | | | | | | | |
| - | 204.16 | - | out | | | | | | | |
| 2263 | 204.17 | 0.093143 | | 78 | 40 | 78 | 2 | 2 | 0 | 1.6e-13 | 3.6e-15 |
| - | 204.19 | - | out | | | | | | | |
| - | 204.22 | - | out | | | | | | | |
| - | 204.24 | - | out | | | | | | | |
| 2264 | 204.25 | 0.077448 | | 80 | 41 | 80 | 2 | 2 | 0 | 6.6e-14 | 5.2e-15 |
| - | 204.27 | - | out | | | | | | | |
| - | 204.3 | - | out | | | | | | | |
| 2265 | 204.32 | 0.064858 | | 82 | 42 | 82 | 2 | 2 | 0 | 1.4e-13 | 4.7e-15 |
| - | 204.33 | - | out | | | | | | | |
| - | 204.35 | - | out | | | | | | | |
| 2266 | 204.37 | 0.058372 | | 84 | 43 | 84 | 2 | 2 | 0 | 4e-14 | 5e-15 |
| - | 204.38 | - | out | | | | | | | |
| - | 204.41 | - | out | | | | | | | |
| 2267 | 204.43 | 0.058372 | | 86 | 44 | 86 | 2 | 2 | 0 | 4.3e-14 | 6.4e-15 |
| - | 204.44 | - | out | | | | | | | |
| - | 204.46 | - | out | | | | | | | |
| - | 204.49 | - | out | | | | | | | |
| 2268 | 204.49 | 0.058372 | | 88 | 45 | 88 | 2 | 2 | 0 | 2.9e-14 | 8.4e-15 |
| - | 204.52 | - | out | | | | | | | |
| - | 204.55 | - | out | | | | | | | |
| 2269 | 204.55 | 0.058372 | | 90 | 46 | 90 | 2 | 2 | 0 | 1.9e-14 | 1.4e-14 |
| - | 204.57 | - | out | | | | | | | |

| | | | | | | | | | |
|---|---|---|---|---|---|---|---|---|---|
| - | 204.6 | - out | | | | | | | |
| 2270 | 204.61 | 0.058372 | 92 | 47 | 92 | 2 | 2 | 0 | 4.2e-14 | 2.1e-14 |
| - | 204.63 | - out | | | | | | | |
| - | 204.66 | - out | | | | | | | |
| 2271 | 204.67 | 0.058372 | 94 | 48 | 94 | 2 | 2 | 0 | 1.6e-14 | 3.4e-14 |
| - | 204.68 | - out | | | | | | | |
| - | 204.71 | - out | | | | | | | |
| - | 204.74 | - out | | | | | | | |
| - | 204.77 | - out | | | | | | | |
| 2272 | 204.78 | 0.11674 | 96 | 49 | 96 | 2 | 2 | 0 | 3.6e-14 | 2.2e-14 |
| - | 204.79 | - out | | | | | | | |
| - | 204.82 | - out | | | | | | | |
| - | 204.85 | - out | | | | | | | |
| - | 204.88 | - out | | | | | | | |
| 2273 | 204.89 | 0.10507 | 98 | 50 | 98 | 2 | 2 | 0 | 4.3e-14 | 7.5e-15 |
| - | 204.9 | - out | | | | | | | |
| - | 204.93 | - out | | | | | | | |
| - | 204.96 | - out | | | | | | | |
| - | 204.99 | - out | | | | | | | |
| 2274 | 204.99 | 0.10507 | 100 | 51 | 100 | 2 | 2 | 0 | 1.1e-13 | 6.6e-15 |
| - | 205.01 | - out | | | | | | | |
| - | 205.04 | - out | | | | | | | |
| - | 205.07 | - out | | | | | | | |
| 2275 | 205.08 | 0.089807 | 102 | 52 | 102 | 2 | 2 | 0 | 6.3e-14 | 5.7e-15 |
| - | 205.1 | - out | | | | | | | |
| - | 205.12 | - out | | | | | | | |
| - | 205.15 | - out | | | | | | | |
| 2276 | 205.16 | 0.078493 | 104 | 53 | 104 | 2 | 2 | 0 | 8.3e-14 | 4.8e-15 |
| - | 205.18 | - out | | | | | | | |
| - | 205.21 | - out | | | | | | | |
| - | 205.23 | - out | | | | | | | |
| 2277 | 205.24 | 0.078493 | 106 | 54 | 106 | 2 | 2 | 0 | 1.9e-14 | 6.2e-15 |
| - | 205.26 | - out | | | | | | | |
| - | 205.29 | - out | | | | | | | |
| - | 205.32 | - out | | | | | | | |
| 2278 | 205.32 | 0.078493 | 108 | 55 | 108 | 2 | 2 | 0 | 2.7e-13 | 4.7e-15 |
| - | 205.34 | - out | | | | | | | |
| - | 205.37 | - out | | | | | | | |
| 2279 | 205.4 | 0.078493 | 110 | 56 | 110 | 2 | 2 | 0 | 2.3e-13 | 5e-15 |
| - | 205.4 | - out | | | | | | | |
| - | 205.43 | - out | | | | | | | |
| - | 205.45 | - out | | | | | | | |
| 2280 | 205.48 | 0.078493 | 112 | 57 | 112 | 2 | 2 | 0 | 1.5e-13 | 4.5e-15 |
| - | 205.48 | - out | | | | | | | |
| - | 205.51 | - out | | | | | | | |
| - | 205.54 | - out | | | | | | | |
| 2281 | 205.55 | 0.070643 | 114 | 58 | 114 | 2 | 2 | 0 | 2e-13 | 5.4e-15 |
| - | 205.56 | - out | | | | | | | |
| - | 205.59 | - out | | | | | | | |
| 2282 | 205.62 | 0.070643 | 116 | 59 | 116 | 2 | 2 | 0 | 1.6e-13 | 3.4e-15 |
| - | 205.62 | - out | | | | | | | |
| - | 205.65 | - out | | | | | | | |
| - | 205.67 | - out | | | | | | | |
| 2283 | 205.69 | 0.070643 | 118 | 60 | 118 | 2 | 2 | 0 | 4.6e-13 | 5.3e-15 |
| - | 205.7 | - out | | | | | | | |
| - | 205.73 | - out | | | | | | | |

```
   -      205.76          - out
2284      205.76    0.070643      120   61  120     2     2      0  1.9e-13  3.1e-15
   -      205.78          - out
   -      205.81          - out
2285      205.83    0.070643      122   62  122     2     2      0    9e-14  2.9e-15
   -      205.84          - out
   -      205.87          - out
   -      205.89          - out
2286       205.9    0.070643      124   63  124     2     2      0  2.8e-13    6e-15
   -      205.92          - out
   -      205.95          - out
2287      205.97    0.070643      126   64  126     2     2      0  2.4e-13  4.1e-15
   -      205.98          - out
   -         206          - out
   -      206.03          - out
2288      206.04    0.070643      128   65  128     2     2      0  6.9e-13  3.6e-15
   -      206.06          - out
   -      206.09          - out
   -      206.11          - out
   -      206.14          - out
   -      206.17          - out
2289      206.18     0.14129      130   66  130     2     2      0  2.9e-13  4.1e-15
   -       206.2          - out
   -      206.22          - out
   -      206.25          - out
   -      206.28          - out
   -      206.31          - out
2290      206.32     0.14129      132   67  132     2     2      0  7.5e-14  6.1e-15
   -      206.33          - out
   -      206.36          - out
   -      206.39          - out
   -      206.42          - out
   -      206.44          - out
2291      206.46     0.14129      134   68  134     2     2      0  1.4e-14  3.7e-14
   -      206.46          - out
2292      206.61     0.14129      136   69  136     2     2      0  8.5e-14  4.7e-15
Number of vertex elements: 3
Number of boundary elements: 112
Number of vertex elements: 3
Number of boundary elements: 112
Number of elements: 534
Minimum element quality: 0.5661
Number of vertex elements: 4
Number of boundary elements: 162
Number of vertex elements: 5
Number of boundary elements: 212
Number of vertex elements: 6
Number of boundary elements: 266
Minimum element quality: 0.07055
Geometry shape function: Linear Lagrange
Time interval 10
Time-dependent solver (BDF)
Number of degrees of freedom solved for: 11758 (plus 7124 internal DOFs).
Nonsymmetric matrix found.
Scales for dependent variables:
Concentration (comp1.cOx): 8.3
```

```
Concentration (comp1.cRed): 5.5
Spatial mesh displacement (comp1.spatial.disp): 2.4e-07
Step         Time    Stepsize      Res  Jac  Sol Order Tfail NLfail   LinErr   LinRes
   -       206.46           - out
   -       206.47           - out
2293       206.49      0.0275        2    2    2     1     0      0  2.4e-14  2.7e-15
   -        206.5           - out
2294       206.52      0.0275        4    3    4     1     0      0  8.6e-14    5e-15
   -       206.53           - out
   -       206.55           - out
2295       206.57       0.055        6    4    6     2     0      0  2.2e-13  2.8e-15
   -       206.58           - out
   -       206.61           - out
   -       206.64           - out
   -       206.66           - out
2296       206.68        0.11        8    5    8     2     0      0  3.9e-13  3.9e-15
   -       206.69           - out
   -       206.72           - out
   -       206.75           - out
   -       206.77           - out
2297       206.79        0.11       10    6   10     2     0      0  5.9e-13  5.4e-15
   -        206.8           - out
   -       206.83           - out
   -       206.86           - out
   -       206.88           - out
2298        206.9        0.11       12    7   12     2     0      0  2.8e-13  4.5e-15
   -       206.91           - out
   -       206.94           - out
   -       206.97           - out
2299       206.98    0.075276       16    9   16     2     1      0  2.1e-13  4.4e-15
   -       206.99           - out
   -       207.02           - out
   -       207.05           - out
2300       207.06    0.075276       18   10   18     2     1      0  8.2e-14  4.8e-15
   -       207.08           - out
   -        207.1           - out
2301       207.12    0.067408       20   11   20     2     1      0  3.5e-13  8.3e-15
   -       207.13           - out
   -       207.16           - out
2302       207.18    0.057645       22   12   22     2     1      0  4.1e-14  4.9e-15
Reassembling sparsity pattern
   -       207.19           - out
   -       207.21           - out
2303       207.23     0.05188       24   13   24     2     1      0  4.3e-15  3.2e-16
   -       207.24           - out
   -       207.27           - out
2304       207.28     0.05188       26   14   26     2     1      0    2e-14  4.5e-16
   -        207.3           - out
   -       207.32           - out
2305       207.33    0.046692       28   15   28     2     1      0  3.2e-15  3.3e-16
   -       207.35           - out
   -       207.38           - out
2306       207.38    0.046692       30   16   30     2     1      0  4.7e-15  3.5e-16
   -       207.41           - out
2307       207.42    0.046692       32   17   32     2     1      0  7.4e-15  4.8e-16
   -       207.43           - out
```

| | | | | | | | | | | |
|---|---|---|---|---|---|---|---|---|---|---|
| - | 207.46 | - | out | | | | | | | |
| 2308 | 207.47 | 0.046692 | 34 | 18 | 34 | 2 | 1 | 0 | 5.4e-14 | 2.9e-15 |
| - | 207.49 | - | out | | | | | | | |
| - | 207.52 | - | out | | | | | | | |
| 2309 | 207.52 | 0.046692 | 36 | 19 | 36 | 2 | 1 | 0 | 2.4e-14 | 3.8e-15 |
| - | 207.54 | - | out | | | | | | | |
| 2310 | 207.56 | 0.046692 | 38 | 20 | 38 | 2 | 1 | 0 | 5.4e-14 | 4.9e-15 |
| - | 207.57 | - | out | | | | | | | |
| - | 207.6 | - | out | | | | | | | |
| 2311 | 207.61 | 0.046692 | 40 | 21 | 40 | 2 | 1 | 0 | 1e-13 | 4.8e-15 |
| - | 207.63 | - | out | | | | | | | |
| - | 207.65 | - | out | | | | | | | |
| 2312 | 207.66 | 0.046692 | 42 | 22 | 42 | 2 | 1 | 0 | 1.3e-14 | 3.7e-15 |
| - | 207.68 | - | out | | | | | | | |
| 2313 | 207.7 | 0.046692 | 44 | 23 | 44 | 2 | 1 | 0 | 1.4e-13 | 5.6e-15 |
| - | 207.71 | - | out | | | | | | | |
| - | 207.74 | - | out | | | | | | | |
| 2314 | 207.75 | 0.046692 | 46 | 24 | 46 | 2 | 1 | 0 | 2.6e-14 | 5.8e-15 |
| - | 207.76 | - | out | | | | | | | |
| - | 207.79 | - | out | | | | | | | |
| 2315 | 207.8 | 0.046692 | 48 | 25 | 48 | 2 | 1 | 0 | 1.3e-14 | 1.2e-14 |
| - | 207.82 | - | out | | | | | | | |
| - | 207.85 | - | out | | | | | | | |
| - | 207.87 | - | out | | | | | | | |
| 2316 | 207.89 | 0.093384 | 50 | 26 | 50 | 2 | 1 | 0 | 3.9e-14 | 3.5e-15 |
| - | 207.9 | - | out | | | | | | | |
| - | 207.93 | - | out | | | | | | | |
| - | 207.96 | - | out | | | | | | | |
| 2317 | 207.98 | 0.084046 | 52 | 27 | 52 | 2 | 1 | 0 | 9.7e-14 | 5e-15 |
| - | 207.98 | - | out | | | | | | | |
| - | 208.01 | - | out | | | | | | | |
| - | 208.04 | - | out | | | | | | | |
| 2318 | 208.06 | 0.084046 | 54 | 28 | 54 | 2 | 1 | 0 | 1.4e-13 | 4e-15 |
| - | 208.07 | - | out | | | | | | | |
| - | 208.09 | - | out | | | | | | | |
| - | 208.12 | - | out | | | | | | | |
| 2319 | 208.13 | 0.075641 | 56 | 29 | 56 | 2 | 1 | 0 | 5.4e-14 | 3.1e-15 |
| - | 208.15 | - | out | | | | | | | |
| - | 208.18 | - | out | | | | | | | |
| - | 208.2 | - | out | | | | | | | |
| 2320 | 208.21 | 0.075641 | 58 | 30 | 58 | 2 | 1 | 0 | 4.5e-13 | 4.2e-15 |
| - | 208.23 | - | out | | | | | | | |
| - | 208.26 | - | out | | | | | | | |
| - | 208.29 | - | out | | | | | | | |
| 2321 | 208.29 | 0.075641 | 60 | 31 | 60 | 2 | 1 | 0 | 3.3e-13 | 4.3e-15 |
| - | 208.31 | - | out | | | | | | | |
| - | 208.34 | - | out | | | | | | | |
| 2322 | 208.36 | 0.075641 | 62 | 32 | 62 | 2 | 1 | 0 | 3.8e-13 | 3.9e-15 |
| - | 208.37 | - | out | | | | | | | |
| - | 208.4 | - | out | | | | | | | |
| - | 208.42 | - | out | | | | | | | |
| 2323 | 208.44 | 0.075641 | 64 | 33 | 64 | 2 | 1 | 0 | 5.6e-13 | 2.8e-15 |
| - | 208.45 | - | out | | | | | | | |
| - | 208.48 | - | out | | | | | | | |
| - | 208.51 | - | out | | | | | | | |
| 2324 | 208.51 | 0.075641 | 66 | 34 | 66 | 2 | 1 | 0 | 4.7e-13 | 3.9e-15 |

| | | | | | | | | | | |
|---|---|---|---|---|---|---|---|---|---|---|
| - | 208.53 | - out | | | | | | | | |
| - | 208.56 | - out | | | | | | | | |
| - | 208.59 | - out | | | | | | | | |
| 2325 | 208.59 | 0.075641 | 68 | 35 | 68 | 2 | 1 | 0 | 7.3e-14 | 3.4e-15 |
| - | 208.62 | - out | | | | | | | | |
| - | 208.64 | - out | | | | | | | | |
| - | 208.67 | - out | | | | | | | | |
| - | 208.7 | - out | | | | | | | | |
| - | 208.73 | - out | | | | | | | | |
| 2326 | 208.74 | 0.15128 | 70 | 36 | 70 | 2 | 1 | 0 | 8.5e-13 | 3.1e-15 |
| - | 208.75 | - out | | | | | | | | |
| - | 208.78 | - out | | | | | | | | |
| - | 208.81 | - out | | | | | | | | |
| - | 208.84 | - out | | | | | | | | |
| - | 208.86 | - out | | | | | | | | |
| - | 208.89 | - out | | | | | | | | |
| 2327 | 208.89 | 0.15128 | 72 | 37 | 72 | 2 | 1 | 0 | 6.7e-13 | 3.6e-15 |
| - | 208.92 | - out | | | | | | | | |
| - | 208.95 | - out | | | | | | | | |
| - | 208.97 | - out | | | | | | | | |
| - | 209 | - out | | | | | | | | |
| - | 209.03 | - out | | | | | | | | |
| 2328 | 209.04 | 0.15128 | 74 | 38 | 74 | 2 | 1 | 0 | 5.8e-13 | 4.7e-15 |
| - | 209.06 | - out | | | | | | | | |
| - | 209.08 | - out | | | | | | | | |
| - | 209.11 | - out | | | | | | | | |
| - | 209.14 | - out | | | | | | | | |
| - | 209.17 | - out | | | | | | | | |
| - | 209.19 | - out | | | | | | | | |
| 2329 | 209.19 | 0.15128 | 76 | 39 | 76 | 2 | 1 | 0 | 7.4e-14 | 5e-15 |
| - | 209.22 | - out | | | | | | | | |
| - | 209.25 | - out | | | | | | | | |
| - | 209.28 | - out | | | | | | | | |
| - | 209.3 | - out | | | | | | | | |
| - | 209.33 | - out | | | | | | | | |
| 2330 | 209.35 | 0.15128 | 78 | 40 | 78 | 2 | 1 | 0 | 4.3e-13 | 3.6e-15 |
| - | 209.36 | - out | | | | | | | | |
| - | 209.39 | - out | | | | | | | | |
| - | 209.41 | - out | | | | | | | | |
| - | 209.44 | - out | | | | | | | | |
| - | 209.47 | - out | | | | | | | | |
| - | 209.5 | - out | | | | | | | | |
| 2331 | 209.5 | 0.15128 | 80 | 41 | 80 | 2 | 1 | 0 | 7.1e-13 | 5.4e-15 |
| - | 209.52 | - out | | | | | | | | |
| - | 209.55 | - out | | | | | | | | |
| 2332 | 209.57 | 0.073805 | 84 | 43 | 84 | 2 | 2 | 0 | 3e-13 | 4.4e-15 |
| - | 209.58 | - out | | | | | | | | |
| - | 209.61 | - out | | | | | | | | |
| - | 209.63 | - out | | | | | | | | |
| 2333 | 209.64 | 0.073805 | 86 | 44 | 86 | 2 | 2 | 0 | 1e-13 | 3.3e-15 |
| - | 209.66 | - out | | | | | | | | |
| - | 209.69 | - out | | | | | | | | |
| 2334 | 209.71 | 0.066425 | 88 | 45 | 88 | 2 | 2 | 0 | 1.2e-13 | 4.8e-15 |
| - | 209.72 | - out | | | | | | | | |
| - | 209.74 | - out | | | | | | | | |
| 2335 | 209.77 | 0.059416 | 90 | 46 | 90 | 2 | 2 | 0 | 1.3e-13 | 5.4e-15 |

| | | | | | | | | | | |
|---|---|---|---|---|---|---|---|---|---|---|
| - | 209.77 | - out | | | | | | | | |
| - | 209.8 | - out | | | | | | | | |
| 2336 | 209.82 | 0.053475 | 92 | 47 | 92 | 2 | 2 | 0 | 8.1e-14 | 6.1e-15 |
| - | 209.83 | - out | | | | | | | | |
| - | 209.85 | - out | | | | | | | | |
| 2337 | 209.88 | 0.053475 | 94 | 48 | 94 | 2 | 2 | 0 | 1.1e-13 | 7.9e-15 |
| - | 209.88 | - out | | | | | | | | |
| - | 209.91 | - out | | | | | | | | |
| 2338 | 209.93 | 0.053475 | 96 | 49 | 96 | 2 | 2 | 0 | 5.4e-14 | 9.6e-15 |
| - | 209.94 | - out | | | | | | | | |
| - | 209.96 | - out | | | | | | | | |
| 2339 | 209.98 | 0.053475 | 98 | 50 | 98 | 2 | 2 | 0 | 7.8e-14 | 1.2e-14 |
| - | 209.99 | - out | | | | | | | | |
| - | 210.02 | - out | | | | | | | | |
| 2340 | 210.04 | 0.053475 | 100 | 51 | 100 | 2 | 2 | 0 | 8.1e-14 | 1.1e-14 |
| - | 210.05 | - out | | | | | | | | |
| - | 210.07 | - out | | | | | | | | |
| 2341 | 210.09 | 0.053475 | 102 | 52 | 102 | 2 | 2 | 0 | 9.7e-14 | 1.1e-14 |
| - | 210.1 | - out | | | | | | | | |
| - | 210.13 | - out | | | | | | | | |
| 2342 | 210.14 | 0.053475 | 104 | 53 | 104 | 2 | 2 | 0 | 2.1e-14 | 1.2e-14 |
| - | 210.16 | - out | | | | | | | | |
| - | 210.18 | - out | | | | | | | | |
| 2343 | 210.2 | 0.053475 | 106 | 54 | 106 | 2 | 2 | 0 | 3.2e-14 | 1.4e-14 |
| - | 210.21 | - out | | | | | | | | |
| - | 210.24 | - out | | | | | | | | |
| - | 210.27 | - out | | | | | | | | |
| - | 210.29 | - out | | | | | | | | |
| 2344 | 210.3 | 0.10695 | 108 | 55 | 108 | 2 | 2 | 0 | 2.7e-14 | 7.5e-14 |
| - | 210.32 | - out | | | | | | | | |
| - | 210.35 | - out | | | | | | | | |
| - | 210.38 | - out | | | | | | | | |
| - | 210.4 | - out | | | | | | | | |
| 2345 | 210.41 | 0.10695 | 110 | 56 | 110 | 2 | 2 | 0 | 7.3e-14 | 7.5e-15 |
| - | 210.43 | - out | | | | | | | | |
| - | 210.46 | - out | | | | | | | | |
| - | 210.49 | - out | | | | | | | | |
| - | 210.51 | - out | | | | | | | | |
| 2346 | 210.52 | 0.10695 | 112 | 57 | 112 | 2 | 2 | 0 | 3.7e-14 | 6.3e-15 |
| - | 210.54 | - out | | | | | | | | |
| - | 210.57 | - out | | | | | | | | |
| - | 210.6 | - out | | | | | | | | |
| 2347 | 210.61 | 0.089197 | 114 | 58 | 114 | 2 | 2 | 0 | 6e-14 | 5.8e-15 |
| - | 210.62 | - out | | | | | | | | |
| - | 210.65 | - out | | | | | | | | |
| - | 210.68 | - out | | | | | | | | |
| 2348 | 210.69 | 0.077948 | 116 | 59 | 116 | 2 | 2 | 0 | 7.3e-14 | 4.6e-15 |
| - | 210.71 | - out | | | | | | | | |
| - | 210.73 | - out | | | | | | | | |
| 2349 | 210.76 | 0.070153 | 118 | 60 | 118 | 2 | 2 | 0 | 3.1e-14 | 6.3e-15 |
| - | 210.76 | - out | | | | | | | | |
| - | 210.79 | - out | | | | | | | | |
| - | 210.82 | - out | | | | | | | | |
| 2350 | 210.83 | 0.070153 | 120 | 61 | 120 | 2 | 2 | 0 | 5.3e-14 | 3.6e-15 |
| - | 210.84 | - out | | | | | | | | |
| - | 210.87 | - out | | | | | | | | |

| | | | | | | | | | | |
|---|---|---|---|---|---|---|---|---|---|---|
| 2351 | 210.9 | 0.070153 | 122 | 62 | 122 | 2 | 2 | 0 | 1.5e-13 | 3.1e-15 |
| - | 210.9 | - out | | | | | | | | |
| - | 210.93 | - out | | | | | | | | |
| - | 210.95 | - out | | | | | | | | |
| 2352 | 210.97 | 0.070153 | 124 | 63 | 124 | 2 | 2 | 0 | 2.2e-13 | 4.6e-15 |
| - | 210.98 | - out | | | | | | | | |
| - | 211.01 | - out | | | | | | | | |
| - | 211.04 | - out | | | | | | | | |
| 2353 | 211.04 | 0.070153 | 126 | 64 | 126 | 2 | 2 | 0 | 1.5e-13 | 5.2e-15 |
| - | 211.06 | - out | | | | | | | | |
| - | 211.09 | - out | | | | | | | | |
| 2354 | 211.11 | 0.070153 | 128 | 65 | 128 | 2 | 2 | 0 | 1.4e-13 | 2.7e-15 |
| - | 211.12 | - out | | | | | | | | |
| - | 211.15 | - out | | | | | | | | |
| - | 211.17 | - out | | | | | | | | |
| 2355 | 211.18 | 0.070153 | 130 | 66 | 130 | 2 | 2 | 0 | 2.1e-13 | 3.7e-15 |
| - | 211.2 | - out | | | | | | | | |
| - | 211.23 | - out | | | | | | | | |
| 2356 | 211.25 | 0.070153 | 132 | 67 | 132 | 2 | 2 | 0 | 2.1e-13 | 2.7e-15 |
| - | 211.26 | - out | | | | | | | | |
| - | 211.28 | - out | | | | | | | | |
| - | 211.31 | - out | | | | | | | | |
| 2357 | 211.32 | 0.070153 | 134 | 68 | 134 | 2 | 2 | 0 | 2.8e-13 | 4.2e-15 |
| - | 211.34 | - out | | | | | | | | |
| - | 211.37 | - out | | | | | | | | |
| 2358 | 211.39 | 0.070153 | 136 | 69 | 136 | 2 | 2 | 0 | 8.7e-14 | 3.7e-15 |
| - | 211.39 | - out | | | | | | | | |
| - | 211.42 | - out | | | | | | | | |
| - | 211.45 | - out | | | | | | | | |
| 2359 | 211.46 | 0.070153 | 138 | 70 | 138 | 2 | 2 | 0 | 5.2e-13 | 3e-15 |
| - | 211.48 | - out | | | | | | | | |
| - | 211.5 | - out | | | | | | | | |
| 2360 | 211.53 | 0.070153 | 140 | 71 | 140 | 2 | 2 | 0 | 2.9e-13 | 5.2e-15 |
| - | 211.53 | - out | | | | | | | | |
| - | 211.56 | - out | | | | | | | | |
| - | 211.59 | - out | | | | | | | | |
| 2361 | 211.6 | 0.070153 | 142 | 72 | 142 | 2 | 2 | 0 | 3.1e-13 | 4.1e-15 |
| - | 211.61 | - out | | | | | | | | |
| - | 211.64 | - out | | | | | | | | |
| - | 211.67 | - out | | | | | | | | |
| 2362 | 211.67 | 0.070153 | 144 | 73 | 144 | 2 | 2 | 0 | 2.1e-13 | 4.5e-15 |
| - | 211.7 | - out | | | | | | | | |
| - | 211.72 | - out | | | | | | | | |
| 2363 | 211.74 | 0.070153 | 146 | 74 | 146 | 2 | 2 | 0 | 8.7e-14 | 5.7e-15 |
| - | 211.75 | - out | | | | | | | | |
| - | 211.78 | - out | | | | | | | | |
| - | 211.81 | - out | | | | | | | | |
| 2364 | 211.81 | 0.070153 | 148 | 75 | 148 | 2 | 2 | 0 | 8.6e-14 | 4.8e-15 |
| - | 211.83 | - out | | | | | | | | |
| - | 211.86 | - out | | | | | | | | |
| 2365 | 211.88 | 0.070153 | 150 | 76 | 150 | 2 | 2 | 0 | 2.4e-13 | 4e-15 |
| - | 211.89 | - out | | | | | | | | |
| - | 211.92 | - out | | | | | | | | |
| - | 211.94 | - out | | | | | | | | |
| 2366 | 211.95 | 0.070153 | 152 | 77 | 152 | 2 | 2 | 0 | 8.4e-14 | 9.1e-15 |
| - | 211.97 | - out | | | | | | | | |

| | | | | | | | | | | |
|---|---|---|---|---|---|---|---|---|---|---|
| - | 212 | - | out | | | | | | | |
| 2367 | 212.02 | 0.070153 | 154 | 78 | 154 | 2 | 2 | 0 | 5.7e-14 | 4.5e-15 |
| - | 212.03 | - | out | | | | | | | |
| - | 212.05 | - | out | | | | | | | |
| - | 212.08 | - | out | | | | | | | |
| 2368 | 212.09 | 0.070153 | 156 | 79 | 156 | 2 | 2 | 0 | 2.5e-13 | 4.5e-15 |
| - | 212.11 | - | out | | | | | | | |
| - | 212.14 | - | out | | | | | | | |
| 2369 | 212.16 | 0.070153 | 158 | 80 | 158 | 2 | 2 | 0 | 7.5e-13 | 7.6e-15 |
| - | 212.16 | - | out | | | | | | | |
| - | 212.19 | - | out | | | | | | | |
| - | 212.22 | - | out | | | | | | | |
| 2370 | 212.23 | 0.070153 | 160 | 81 | 160 | 2 | 2 | 0 | 2.4e-13 | 3.6e-15 |
| - | 212.25 | - | out | | | | | | | |
| - | 212.27 | - | out | | | | | | | |
| 2371 | 212.3 | 0.070153 | 162 | 82 | 162 | 2 | 2 | 0 | 1.2e-12 | 5.7e-15 |
| - | 212.3 | - | out | | | | | | | |
| - | 212.33 | - | out | | | | | | | |
| - | 212.36 | - | out | | | | | | | |
| 2372 | 212.37 | 0.070153 | 164 | 83 | 164 | 2 | 2 | 0 | 1.5e-12 | 6.6e-15 |
| - | 212.38 | - | out | | | | | | | |
| - | 212.41 | - | out | | | | | | | |
| 2373 | 212.43 | 0.06025 | 166 | 84 | 166 | 2 | 2 | 0 | 4.9e-13 | 5.7e-15 |
| - | 212.44 | - | out | | | | | | | |
| - | 212.47 | - | out | | | | | | | |
| 2374 | 212.48 | 0.05275 | 168 | 85 | 168 | 2 | 2 | 0 | 2.6e-13 | 7e-15 |
| - | 212.49 | - | out | | | | | | | |
| - | 212.52 | - | out | | | | | | | |
| 2375 | 212.53 | 0.047475 | 170 | 86 | 170 | 2 | 2 | 0 | 2e-13 | 1.1e-14 |
| - | 212.55 | - | out | | | | | | | |
| 2376 | 212.57 | 0.042727 | 172 | 87 | 172 | 2 | 2 | 0 | 2.4e-13 | 3.3e-15 |
| - | 212.58 | - | out | | | | | | | |
| - | 212.6 | - | out | | | | | | | |
| 2377 | 212.62 | 0.042727 | 174 | 88 | 174 | 2 | 2 | 0 | 1.7e-13 | 3.2e-15 |
| - | 212.63 | - | out | | | | | | | |
| 2378 | 212.65 | 0.038455 | 176 | 89 | 176 | 2 | 2 | 0 | 9.5e-14 | 4e-15 |
| - | 212.66 | - | out | | | | | | | |
| - | 212.69 | - | out | | | | | | | |
| 2379 | 212.69 | 0.038455 | 178 | 90 | 178 | 2 | 2 | 0 | 1.2e-13 | 4.3e-15 |
| - | 212.71 | - | out | | | | | | | |
| 2380 | 212.73 | 0.038455 | 180 | 91 | 180 | 2 | 2 | 0 | 9.6e-14 | 4.7e-15 |
| - | 212.74 | - | out | | | | | | | |
| - | 212.77 | - | out | | | | | | | |
| 2381 | 212.77 | 0.038455 | 182 | 92 | 182 | 2 | 2 | 0 | 3.2e-14 | 3.1e-15 |
| - | 212.8 | - | out | | | | | | | |
| 2382 | 212.81 | 0.038455 | 184 | 93 | 184 | 2 | 2 | 0 | 4.8e-14 | 5.2e-15 |
| - | 212.82 | - | out | | | | | | | |
| 2383 | 212.85 | 0.038455 | 186 | 94 | 186 | 2 | 2 | 0 | 4.4e-14 | 5.9e-15 |
| - | 212.85 | - | out | | | | | | | |
| - | 212.88 | - | out | | | | | | | |
| 2384 | 212.88 | 0.038455 | 188 | 95 | 188 | 2 | 2 | 0 | 2.7e-14 | 4.5e-15 |
| - | 212.91 | - | out | | | | | | | |
| 2385 | 212.92 | 0.038455 | 190 | 96 | 190 | 2 | 2 | 0 | 1.3e-14 | 4.7e-15 |
| - | 212.93 | - | out | | | | | | | |
| - | 212.96 | - | out | | | | | | | |
| 2386 | 212.96 | 0.038455 | 192 | 97 | 192 | 2 | 2 | 0 | 2e-14 | 3.7e-15 |

| | | | | | | | | | | |
|---|---|---|---|---|---|---|---|---|---|---|
| - | 212.99 | - out | | | | | | | | |
| 2387 | 213 | 0.038455 | 194 | 98 | 194 | 2 | 2 | 0 | 3.1e-14 | 5e-15 |
| - | 213.02 | - out | | | | | | | | |
| 2388 | 213.04 | 0.038455 | 196 | 99 | 196 | 2 | 2 | 0 | 3.8e-14 | 4.7e-15 |
| - | 213.04 | - out | | | | | | | | |
| - | 213.07 | - out | | | | | | | | |
| 2389 | 213.08 | 0.038455 | 198 | 100 | 198 | 2 | 2 | 0 | 5.9e-14 | 5.1e-15 |
| - | 213.1 | - out | | | | | | | | |
| 2390 | 213.12 | 0.038455 | 200 | 101 | 200 | 2 | 2 | 0 | 5.2e-14 | 5.9e-15 |
| - | 213.13 | - out | | | | | | | | |
| - | 213.15 | - out | | | | | | | | |
| 2391 | 213.15 | 0.038455 | 202 | 102 | 202 | 2 | 2 | 0 | 3.6e-14 | 3.9e-15 |
| - | 213.18 | - out | | | | | | | | |
| 2392 | 213.19 | 0.038455 | 204 | 103 | 204 | 2 | 2 | 0 | 2.6e-14 | 5.3e-15 |
| - | 213.21 | - out | | | | | | | | |
| - | 213.24 | - out | | | | | | | | |
| - | 213.26 | - out | | | | | | | | |
| 2393 | 213.27 | 0.076909 | 206 | 104 | 206 | 2 | 2 | 0 | 1.7e-14 | 8e-15 |
| - | 213.29 | - out | | | | | | | | |
| - | 213.32 | - out | | | | | | | | |
| - | 213.35 | - out | | | | | | | | |
| 2394 | 213.35 | 0.076909 | 208 | 105 | 208 | 2 | 2 | 0 | 8.1e-14 | 5.3e-15 |
| - | 213.37 | - out | | | | | | | | |
| - | 213.4 | - out | | | | | | | | |
| 2395 | 213.42 | 0.076909 | 210 | 106 | 210 | 2 | 2 | 0 | 2.5e-13 | 4.1e-15 |
| - | 213.43 | - out | | | | | | | | |
| - | 213.46 | - out | | | | | | | | |
| - | 213.48 | - out | | | | | | | | |
| 2396 | 213.5 | 0.076909 | 212 | 107 | 212 | 2 | 2 | 0 | 4.7e-14 | 3.1e-15 |
| - | 213.51 | - out | | | | | | | | |
| - | 213.54 | - out | | | | | | | | |
| - | 213.57 | - out | | | | | | | | |
| 2397 | 213.58 | 0.076909 | 214 | 108 | 214 | 2 | 2 | 0 | 1.6e-13 | 3.2e-15 |
| - | 213.59 | - out | | | | | | | | |
| - | 213.62 | - out | | | | | | | | |
| - | 213.65 | - out | | | | | | | | |
| 2398 | 213.65 | 0.076909 | 216 | 109 | 216 | 2 | 2 | 0 | 1.7e-13 | 3.6e-15 |
| - | 213.68 | - out | | | | | | | | |
| - | 213.7 | - out | | | | | | | | |
| - | 213.73 | - out | | | | | | | | |
| 2399 | 213.73 | 0.076909 | 218 | 110 | 218 | 2 | 2 | 0 | 7.7e-14 | 4e-15 |
| - | 213.76 | - out | | | | | | | | |
| - | 213.79 | - out | | | | | | | | |
| 2400 | 213.81 | 0.076909 | 220 | 111 | 220 | 2 | 2 | 0 | 6.2e-13 | 4.2e-15 |
| - | 213.81 | - out | | | | | | | | |
| - | 213.84 | - out | | | | | | | | |
| - | 213.87 | - out | | | | | | | | |
| 2401 | 213.88 | 0.076909 | 222 | 112 | 222 | 2 | 2 | 0 | 2.3e-13 | 3.7e-15 |
| - | 213.9 | - out | | | | | | | | |
| - | 213.92 | - out | | | | | | | | |
| - | 213.95 | - out | | | | | | | | |
| 2402 | 213.96 | 0.076909 | 224 | 113 | 224 | 2 | 2 | 0 | 4.1e-13 | 3.2e-15 |
| - | 213.98 | - out | | | | | | | | |
| - | 214.01 | - out | | | | | | | | |
| - | 214.03 | - out | | | | | | | | |
| 2403 | 214.04 | 0.076909 | 226 | 114 | 226 | 2 | 2 | 0 | 2.6e-13 | 4.2e-15 |

```
   -      214.06          - out
   -      214.09          - out
   -      214.12          - out
2404      214.12    0.076909      228   115   228      2      2       0     9e-14     3e-15
   -      214.14          - out
   -      214.17          - out
   -       214.2          - out
   -      214.23          - out
   -      214.25          - out
2405      214.27     0.15382      230   116   230      2      2       0   2.4e-12   5.1e-15
   -      214.28          - out
   -      214.31          - out
   -      214.34          - out
   -      214.36          - out
   -      214.39          - out
   -      214.42          - out
2406      214.42     0.15382      232   117   232      2      2       0   6.3e-13   3.8e-15
   -      214.45          - out
   -      214.47          - out
   -       214.5          - out
2407      214.58     0.15382      234   118   234      2      2       0   1.8e-13   7.7e-15
Time-stepping completed.
Geometry shape function: Linear Lagrange
Solution time: 1411 s. (23 minutes, 31 seconds)
Physical memory: 2.85 GB
Virtual memory: 2.97 GB
Ended at Jun 15, 2023 6:56:50 PM.
----- Time-Dependent Solver 1 in Study 1  (CV  1 to 39)/Solution 1 (sol2) ----->
```

**Advanced (aDef)**

### ASSEMBLY SETTINGS

| Description | Value |
|---|---|
| Reuse sparsity pattern | On |

**Fully Coupled 1 (fc1)**

### GENERAL

| Description | Value |
|---|---|
| Linear solver | Direct 1 |

### METHOD AND TERMINATION

| Description | Value |
|---|---|
| Damping factor | 0.9 |
| Jacobian update | Once per time step |
| Maximum number of iterations | 8 |
| Stabilization and acceleration | Anderson acceleration |
| Dimension of iteration space | 5 |

**Automatic Remeshing 1 (ar1)**

### GENERAL

| Description | Value |
|---|---|
| Remesh in geometry | Geometry 1 |

### CONDITION FOR REMESHING

| Description | Value |
|---|---|
| Condition type | Distortion |

### OUTPUT

| Description | Value |
|---|---|
| Solution | Remeshed Solution 1 |
| Meshes | {mesh2, mesh3, mesh4, mesh5, mesh6, mesh7, mesh8, mesh9, mesh10} |

# 4 Study 2 (CV 40 to 44)

COMPUTATION INFORMATION

| | |
|---|---|
| Computation time | 9 min 5 s |

## 4.1 TIME DEPENDENT

| Times | Unit |
|---|---|
| range(39*t_cv,t_tot/nb/200,44*t_cv) | s |

STUDY SETTINGS

| Description | Value |
|---|---|
| Include geometric nonlinearity | Off |

STUDY SETTINGS

| Description | Value |
|---|---|
| Output times | {214.50000000000003, 214.52750000000003, 214.55500000000004, 214.58250000000004, 214.61000000000004, 214.63750000000002, 214.66500000000002, 214.69250000000002, 214.72000000000003, 214.74750000000003, 214.77500000000003, 214.80250000000004, 214.83000000000004, 214.85750000000002, 214.88500000000002, 214.91250000000002, 214.94000000000003, 214.96750000000003, 214.99500000000003, 215.02250000000004, 215.05000000000004, 215.07750000000001, 215.10500000000002, 215.13250000000002, 215.16000000000003, 215.18750000000003, 215.21500000000003, 215.24250000000004, 215.27000000000004, 215.29750000000004, 215.32500000000002, 215.35250000000002, 215.38000000000002, 215.40750000000003, 215.43500000000003, 215.46250000000003, 215.49000000000004, 215.51750000000004, 215.54500000000002, 215.57250000000002, 215.60000000000002, 215.62750000000003, 215.65500000000003, 215.68250000000003, 215.71000000000004, 215.73750000000004, 215.76500000000001, 215.79250000000002, 215.82000000000002, 215.84750000000003, 215.87500000000003, 215.90250000000003, 215.93000000000004, 215.95750000000004, 215.98500000000004, 216.01250000000002, 216.04000000000002, 216.06750000000002, 216.09500000000003, 216.12250000000003, 216.15000000000003, 216.17750000000004, 216.20500000000004, 216.23250000000002, 216.26000000000002, 216.28750000000002, 216.31500000000003, 216.34250000000003, 216.37000000000003, 216.39750000000004, 216.42500000000004, 216.45250000000001, 216.48000000000002, 216.50750000000002, 216.53500000000003, 216.56250000000003, 216.59000000000003, 216.61750000000004, 216.64500000000004, 216.67250000000004, 216.70000000000002, 216.72750000000002, 216.75500000000002, 216.78250000000003, 216.81000000000003, 216.83750000000003, 216.86500000000004, 216.89250000000004, 216.92000000000002, 216.94750000000002, 216.97500000000002, 217.00250000000003, 217.03000000000003, |

| Description | Value |
| --- | --- |
| | 217.05750000000003, 217.08500000000004, 217.11250000000004, 217.14000000000004, 217.16750000000002, 217.19500000000002, 217.22250000000003, 217.25000000000003, 217.27750000000003, 217.30500000000004, 217.33250000000004, 217.36000000000004, 217.38750000000002, 217.41500000000002, 217.44250000000002, 217.47000000000003, 217.49750000000003, 217.52500000000003, 217.55250000000004, 217.58000000000004, 217.60750000000002, 217.63500000000002, 217.66250000000002, 217.69000000000003, 217.71750000000003, 217.74500000000003, 217.77250000000004, 217.80000000000004, 217.82750000000004, 217.85500000000002, 217.88250000000002, 217.91000000000003, 217.93750000000003, 217.96500000000003, 217.99250000000004, 218.02000000000004, 218.04750000000004, 218.07500000000002, 218.10250000000002, 218.13000000000002, 218.15750000000003, 218.18500000000003, 218.21250000000003, 218.24000000000004, 218.26750000000004, 218.29500000000002, 218.32250000000002, 218.35000000000002, 218.37750000000003, 218.40500000000003, 218.43250000000003, 218.46000000000004, 218.48750000000004, 218.51500000000004, 218.54250000000002, 218.57000000000002, 218.59750000000003, 218.62500000000003, 218.65250000000003, 218.68000000000004, 218.70750000000004, 218.73500000000004, 218.76250000000002, 218.79000000000002, 218.81750000000002, 218.84500000000003, 218.87250000000003, 218.90000000000003, 218.92750000000004, 218.95500000000004, 218.98250000000002, 219.01000000000002, 219.03750000000002, 219.06500000000003, 219.09250000000003, 219.12000000000003, 219.14750000000004, 219.17500000000004, 219.20250000000004, 219.23000000000002, 219.25750000000002, 219.28500000000003, 219.31250000000003, 219.34000000000003, 219.36750000000004, 219.39500000000004, 219.42250000000004, 219.45000000000002, 219.47750000000002, 219.50500000000002, 219.53250000000003, 219.56000000000003, 219.58750000000003, 219.61500000000004, 219.64250000000004, 219.67000000000002, 219.69750000000002, 219.72500000000002, 219.75250000000003, 219.78000000000003, 219.80750000000003, 219.83500000000004, 219.86250000000004, 219.89000000000004, 219.91750000000002, 219.94500000000002, 219.97250000000003, 220.00000000000003, 220.02750000000003, 220.05500000000004, 220.08250000000004, 220.11000000000004, 220.13750000000002, 220.16500000000002, 220.19250000000002, 220.22000000000003, 220.24750000000003, 220.27500000000003, 220.30250000000004, 220.33000000000004, 220.35750000000002, 220.38500000000002, 220.41250000000002, 220.44000000000003, 220.46750000000003, 220.49500000000003, 220.52250000000004, 220.55000000000004, 220.57750000000004, 220.60500000000002, 220.63250000000002, 220.66000000000003, 220.68750000000003, 220.71500000000003, 220.74250000000004, 220.77000000000004, 220.79750000000004, 220.82500000000002, 220.85250000000002, 220.88000000000002, 220.90750000000003, 220.93500000000003, 220.96250000000003, 220.99000000000004, |

| Description | Value |
|---|---|
| | 221.01750000000004, 221.04500000000002, 221.07250000000002, 221.10000000000002, 221.12750000000003, 221.15500000000003, 221.18250000000003, 221.21000000000004, 221.23750000000004, 221.26500000000004, 221.29250000000002, 221.32000000000002, 221.34750000000003, 221.37500000000003, 221.40250000000003, 221.43000000000004, 221.45750000000004, 221.48500000000004, 221.51250000000002, 221.54000000000002, 221.56750000000002, 221.59500000000003, 221.62250000000003, 221.65000000000003, 221.67750000000004, 221.70500000000004, 221.73250000000002, 221.76000000000002, 221.78750000000002, 221.81500000000003, 221.84250000000003, 221.87000000000003, 221.89750000000004, 221.92500000000004, 221.95250000000004, 221.98000000000002, 222.00750000000002, 222.03500000000003, 222.06250000000003, 222.09000000000003, 222.11750000000004, 222.14500000000004, 222.17250000000004, 222.20000000000002, 222.22750000000002, 222.25500000000002, 222.28250000000003, 222.31000000000003, 222.33750000000003, 222.36500000000004, 222.39250000000004, 222.42000000000002, 222.44750000000002, 222.47500000000002, 222.50250000000003, 222.53000000000003, 222.55750000000003, 222.58500000000004, 222.61250000000004, 222.64000000000004, 222.66750000000002, 222.69500000000002, 222.72250000000003, 222.75000000000003, 222.77750000000003, 222.80500000000004, 222.83250000000004, 222.86000000000004, 222.88750000000002, 222.91500000000002, 222.94250000000002, 222.97000000000003, 222.99750000000003, 223.02500000000003, 223.05250000000004, 223.08000000000004, 223.10750000000002, 223.13500000000002, 223.16250000000002, 223.19000000000003, 223.21750000000003, 223.24500000000003, 223.27250000000004, 223.30000000000004, 223.32750000000004, 223.35500000000002, 223.38250000000002, 223.41000000000003, 223.43750000000003, 223.46500000000003, 223.49250000000004, 223.52000000000004, 223.54750000000004, 223.57500000000002, 223.60250000000002, 223.63000000000002, 223.65750000000003, 223.68500000000003, 223.71250000000003, 223.74000000000004, 223.76750000000004, 223.79500000000002, 223.82250000000002, 223.85000000000002, 223.87750000000003, 223.90500000000003, 223.93250000000003, 223.96000000000004, 223.98750000000004, 224.01500000000004, 224.04250000000002, 224.07000000000002, 224.09750000000003, 224.12500000000003, 224.15250000000003, 224.18000000000004, 224.20750000000004, 224.23500000000004, 224.26250000000002, 224.29000000000002, 224.31750000000002, 224.34500000000003, 224.37250000000003, 224.40000000000003, 224.42750000000004, 224.45500000000004, 224.48250000000002, 224.51000000000002, 224.53750000000002, 224.56500000000003, 224.59250000000003, 224.62000000000003, 224.64750000000004, 224.67500000000004, 224.70250000000004, 224.73000000000002, 224.75750000000002, 224.78500000000003, 224.81250000000003, 224.84000000000003, 224.86750000000004, 224.89500000000004, 224.92250000000004, 224.95000000000002, |

| Description | Value |
|---|---|
| | 224.97750000000002, 225.00500000000002, 225.03250000000003, 225.06000000000003, 225.08750000000003, 225.11500000000004, 225.14250000000004, 225.17000000000002, 225.19750000000002, 225.22500000000002, 225.25250000000003, 225.28000000000003, 225.30750000000003, 225.33500000000004, 225.36250000000004, 225.39000000000004, 225.41750000000002, 225.44500000000002, 225.47250000000003, 225.50000000000003, 225.52750000000003, 225.55500000000004, 225.58250000000004, 225.61000000000004, 225.63750000000002, 225.66500000000002, 225.69250000000002, 225.72000000000003, 225.74750000000003, 225.77500000000003, 225.80250000000004, 225.83000000000004, 225.85750000000002, 225.88500000000002, 225.91250000000002, 225.94000000000003, 225.96750000000003, 225.99500000000003, 226.02250000000004, 226.05000000000004, 226.07750000000004, 226.10500000000002, 226.13250000000002, 226.16000000000003, 226.18750000000003, 226.21500000000003, 226.24250000000004, 226.27000000000004, 226.29750000000004, 226.32500000000002, 226.35250000000002, 226.38000000000002, 226.40750000000003, 226.43500000000003, 226.46250000000003, 226.49000000000004, 226.51750000000004, 226.54500000000002, 226.57250000000002, 226.60000000000002, 226.62750000000003, 226.65500000000003, 226.68250000000003, 226.71000000000004, 226.73750000000004, 226.76500000000004, 226.79250000000002, 226.82000000000002, 226.84750000000003, 226.87500000000003, 226.90250000000003, 226.93000000000004, 226.95750000000004, 226.98500000000004, 227.01250000000002, 227.04000000000002, 227.06750000000002, 227.09500000000003, 227.12250000000003, 227.15000000000003, 227.17750000000004, 227.20500000000004, 227.23250000000002, 227.26000000000002, 227.28750000000002, 227.31500000000003, 227.34250000000003, 227.37000000000003, 227.39750000000004, 227.42500000000004, 227.45250000000004, 227.48000000000002, 227.50750000000002, 227.53500000000003, 227.56250000000003, 227.59000000000003, 227.61750000000004, 227.64500000000004, 227.67250000000004, 227.70000000000002, 227.72750000000002, 227.75500000000002, 227.78250000000003, 227.81000000000003, 227.83750000000003, 227.86500000000004, 227.89250000000004, 227.92000000000002, 227.94750000000002, 227.97500000000002, 228.00250000000003, 228.03000000000003, 228.05750000000003, 228.08500000000004, 228.11250000000004, 228.14000000000004, 228.16750000000002, 228.19500000000002, 228.22250000000003, 228.25000000000003, 228.27750000000003, 228.30500000000004, 228.33250000000004, 228.36000000000004, 228.38750000000002, 228.41500000000002, 228.44250000000002, 228.47000000000003, 228.49750000000003, 228.52500000000003, 228.55250000000004, 228.58000000000004, 228.60750000000002, 228.63500000000002, 228.66250000000002, 228.69000000000003, 228.71750000000003, 228.74500000000003, 228.77250000000004, 228.80000000000004, 228.82750000000004, 228.85500000000002, 228.88250000000002, 228.91000000000003, |

| Description | Value |
| --- | --- |
| | 228.93750000000003, 228.96500000000003, 228.99250000000004, 229.02000000000004, 229.04750000000004, 229.07500000000002, 229.10250000000002, 229.13000000000002, 229.15750000000003, 229.18500000000003, 229.21250000000003, 229.24000000000004, 229.26750000000004, 229.29500000000002, 229.32250000000002, 229.35000000000002, 229.37750000000003, 229.40500000000003, 229.43250000000003, 229.46000000000004, 229.48750000000004, 229.51500000000004, 229.54250000000002, 229.57000000000002, 229.59750000000003, 229.62500000000003, 229.65250000000003, 229.68000000000004, 229.70750000000004, 229.73500000000004, 229.76250000000005, 229.79000000000002, 229.81750000000002, 229.84500000000003, 229.87250000000003, 229.90000000000003, 229.92750000000004, 229.95500000000004, 229.98250000000002, 230.01000000000002, 230.03750000000002, 230.06500000000003, 230.09250000000003, 230.12000000000003, 230.14750000000004, 230.17500000000004, 230.20250000000004, 230.23000000000002, 230.25750000000002, 230.28500000000003, 230.31250000000003, 230.34000000000003, 230.36750000000004, 230.39500000000004, 230.42250000000004, 230.45000000000005, 230.47750000000002, 230.50500000000002, 230.53250000000003, 230.56000000000003, 230.58750000000003, 230.61500000000004, 230.64250000000004, 230.67000000000002, 230.69750000000002, 230.72500000000002, 230.75250000000003, 230.78000000000003, 230.80750000000003, 230.83500000000004, 230.86250000000004, 230.89000000000004, 230.91750000000002, 230.94500000000002, 230.97250000000003, 231.00000000000003, 231.02750000000003, 231.05500000000004, 231.08250000000004, 231.11000000000004, 231.13750000000005, 231.16500000000002, 231.19250000000002, 231.22000000000003, 231.24750000000003, 231.27500000000003, 231.30250000000004, 231.33000000000004, 231.35750000000002, 231.38500000000002, 231.41250000000002, 231.44000000000003, 231.46750000000003, 231.49500000000003, 231.52250000000004, 231.55000000000004, 231.57750000000004, 231.60500000000002, 231.63250000000002, 231.66000000000003, 231.68750000000003, 231.71500000000003, 231.74250000000004, 231.77000000000004, 231.79750000000004, 231.82500000000005, 231.85250000000002, 231.88000000000002, 231.90750000000003, 231.93500000000003, 231.96250000000003, 231.99000000000004, 232.01750000000004, 232.04500000000002, 232.07250000000002, 232.10000000000002, 232.12750000000003, 232.15500000000003, 232.18250000000003, 232.21000000000004, 232.23750000000004, 232.26500000000004, 232.29250000000002, 232.32000000000002, 232.34750000000003, 232.37500000000003, 232.40250000000003, 232.43000000000004, 232.45750000000004, 232.48500000000004, 232.51250000000005, 232.54000000000002, 232.56750000000002, 232.59500000000003, 232.62250000000003, 232.65000000000003, 232.67750000000004, 232.70500000000004, 232.73250000000002, 232.76000000000002, 232.78750000000002, 232.81500000000003, 232.84250000000003, 232.87000000000003, |

| Description | Value |
| --- | --- |
| | 232.89750000000004, 232.92500000000004, 232.95250000000004, 232.98000000000002, 233.00750000000002, 233.03500000000003, 233.06250000000003, 233.09000000000003, 233.11750000000004, 233.14500000000004, 233.17250000000004, 233.20000000000005, 233.22750000000002, 233.25500000000002, 233.28250000000003, 233.31000000000003, 233.33750000000003, 233.36500000000004, 233.39250000000004, 233.42000000000002, 233.44750000000002, 233.47500000000002, 233.50250000000003, 233.53000000000003, 233.55750000000003, 233.58500000000004, 233.61250000000004, 233.64000000000004, 233.66750000000002, 233.69500000000002, 233.72250000000003, 233.75000000000003, 233.77750000000003, 233.80500000000004, 233.83250000000004, 233.86000000000004, 233.88750000000005, 233.91500000000002, 233.94250000000002, 233.97000000000003, 233.99750000000003, 234.02500000000003, 234.05250000000004, 234.08000000000004, 234.10750000000002, 234.13500000000002, 234.16250000000002, 234.19000000000003, 234.21750000000003, 234.24500000000003, 234.27250000000004, 234.30000000000004, 234.32750000000004, 234.35500000000002, 234.38250000000002, 234.41000000000003, 234.43750000000003, 234.46500000000003, 234.49250000000004, 234.52000000000004, 234.54750000000004, 234.57500000000005, 234.60250000000002, 234.63000000000002, 234.65750000000003, 234.68500000000003, 234.71250000000003, 234.74000000000004, 234.76750000000004, 234.79500000000002, 234.82250000000002, 234.85000000000002, 234.87750000000003, 234.90500000000003, 234.93250000000003, 234.96000000000004, 234.98750000000004, 235.01500000000004, 235.04250000000002, 235.07000000000002, 235.09750000000003, 235.12500000000003, 235.15250000000003, 235.18000000000004, 235.20750000000004, 235.23500000000004, 235.26250000000005, 235.29000000000002, 235.31750000000002, 235.34500000000003, 235.37250000000003, 235.40000000000003, 235.42750000000004, 235.45500000000004, 235.48250000000002, 235.51000000000002, 235.53750000000002, 235.56500000000003, 235.59250000000003, 235.62000000000003, 235.64750000000004, 235.67500000000004, 235.70250000000004, 235.73000000000002, 235.75750000000002, 235.78500000000003, 235.81250000000003, 235.84000000000003, 235.86750000000004, 235.89500000000004, 235.92250000000004, 235.95000000000005, 235.97750000000002, 236.00500000000002, 236.03250000000003, 236.06000000000003, 236.08750000000003, 236.11500000000004, 236.14250000000004, 236.17000000000002, 236.19750000000002, 236.22500000000002, 236.25250000000003, 236.28000000000003, 236.30750000000003, 236.33500000000004, 236.36250000000004, 236.39000000000004, 236.41750000000002, 236.44500000000002, 236.47250000000003, 236.50000000000003, 236.52750000000003, 236.55500000000004, 236.58250000000004, 236.61000000000004, 236.63750000000005, 236.66500000000002, 236.69250000000002, 236.72000000000003, 236.74750000000003, 236.77500000000003, 236.80250000000004, 236.83000000000004, |

| Description | Value |
|---|---|
| | 236.85750000000002, 236.88500000000002, 236.91250000000002, 236.94000000000003, 236.96750000000003, 236.99500000000003, 237.02250000000004, 237.05000000000004, 237.07750000000004, 237.10500000000002, 237.13250000000002, 237.16000000000003, 237.18750000000003, 237.21500000000003, 237.24250000000004, 237.27000000000004, 237.29750000000004, 237.32500000000005, 237.35250000000002, 237.38000000000002, 237.40750000000003, 237.43500000000003, 237.46250000000003, 237.49000000000004, 237.51750000000004, 237.54500000000002, 237.57250000000002, 237.60000000000002, 237.62750000000003, 237.65500000000003, 237.68250000000003, 237.71000000000004, 237.73750000000004, 237.76500000000004, 237.79250000000002, 237.82000000000002, 237.84750000000003, 237.87500000000003, 237.90250000000003, 237.93000000000004, 237.95750000000004, 237.98500000000004, 238.01250000000005, 238.04000000000002, 238.06750000000002, 238.09500000000003, 238.12250000000003, 238.15000000000003, 238.17750000000004, 238.20500000000004, 238.23250000000002, 238.26000000000002, 238.28750000000002, 238.31500000000003, 238.34250000000003, 238.37000000000003, 238.39750000000004, 238.42500000000004, 238.45250000000004, 238.48000000000002, 238.50750000000002, 238.53500000000003, 238.56250000000003, 238.59000000000003, 238.61750000000004, 238.64500000000004, 238.67250000000004, 238.70000000000005, 238.72750000000002, 238.75500000000002, 238.78250000000003, 238.81000000000003, 238.83750000000003, 238.86500000000004, 238.89250000000004, 238.92000000000002, 238.94750000000002, 238.97500000000002, 239.00250000000003, 239.03000000000003, 239.05750000000003, 239.08500000000004, 239.11250000000004, 239.14000000000004, 239.16750000000002, 239.19500000000002, 239.22250000000003, 239.25000000000003, 239.27750000000003, 239.30500000000004, 239.33250000000004, 239.36000000000004, 239.38750000000005, 239.41500000000002, 239.44250000000002, 239.47000000000003, 239.49750000000003, 239.52500000000003, 239.55250000000004, 239.58000000000004, 239.60750000000002, 239.63500000000002, 239.66250000000002, 239.69000000000003, 239.71750000000003, 239.74500000000003, 239.77250000000004, 239.80000000000004, 239.82750000000004, 239.85500000000002, 239.88250000000002, 239.91000000000003, 239.93750000000003, 239.96500000000003, 239.99250000000004, 240.02000000000004, 240.04750000000004, 240.07500000000005, 240.10250000000002, 240.13000000000002, 240.15750000000003, 240.18500000000003, 240.21250000000003, 240.24000000000004, 240.26750000000004, 240.29500000000002, 240.32250000000005, 240.35000000000002, 240.37750000000003, 240.40500000000003, 240.43250000000003, 240.46000000000004, 240.48750000000004, 240.51500000000004, 240.54250000000002, 240.57000000000002, 240.59750000000003, 240.62500000000003, 240.65250000000003, 240.68000000000004, 240.70750000000004, 240.73500000000004, 240.76250000000005, 240.79000000000002, |

| Description | Value |
|---|---|
| | 240.81750000000002, 240.84500000000003, 240.87250000000003, 240.90000000000003, 240.92750000000004, 240.95500000000004, 240.98250000000002, 241.01000000000005, 241.03750000000002, 241.06500000000003, 241.09250000000003, 241.12000000000003, 241.14750000000004, 241.17500000000004, 241.20250000000004, 241.23000000000002, 241.25750000000002, 241.28500000000003, 241.31250000000003, 241.34000000000003, 241.36750000000004, 241.39500000000004, 241.42250000000004, 241.45000000000005, 241.47750000000002, 241.50500000000002, 241.53250000000003, 241.56000000000003, 241.58750000000003, 241.61500000000004, 241.64250000000004, 241.67000000000004, 241.69750000000005, 241.72500000000002, 241.75250000000003, 241.78000000000003, 241.80750000000003, 241.83500000000004, 241.86250000000004, 241.89000000000004, 241.91750000000002, 241.94500000000002, 241.97250000000003, 242.00000000000003} |

MESH

| Feature | Value |
|---|---|
| Geometry 1 | mesh10 |

PHYSICS AND VARIABLES SELECTION

| Physics interface | Discretization |
|---|---|
| Transport of Diluted Species in droplet (tds) | physics |
| Moving Mesh (ale) | physics |

MESH SELECTION

| Geometry | Mesh |
|---|---|
| Geometry 1 (geom1) | mesh10 |

## 4.2 SOLVER CONFIGURATIONS

### 4.2.1 Solution 4

#### Compile Equations: Time Dependent (st1)

STUDY AND STEP

| Description | Value |
|---|---|
| Use study | Study 2 (CV 40 to 44) |
| Use study step | Time Dependent |

LOG

```
<---- Compile Equations: Time Dependent in Study 2 (CV 40 to 44)/Solution 4
      (sol4) ------------------------------------------------------------------
Started at Jun 21, 2023 12:18:29 PM.
Geometry shape function: Linear Lagrange
Running on Intel64 Family 6 Model 158 Stepping 10, GenuineIntel.
Using 1 socket with 6 cores in total on LAPTOP-2492RD8I.
Available memory: 32.27 GB.
Time: 1 s.
Physical memory: 2.37 GB
Virtual memory: 2.6 GB
Ended at Jun 21, 2023 12:18:30 PM.
----- Compile Equations: Time Dependent in Study 2 (CV 40 to 44)/Solution 4
      (sol4) ----------------------------------------------------------------->
```

### Dependent Variables 1 (v1)

#### INITIAL VALUES OF VARIABLES SOLVED FOR

| Description | Value |
| --- | --- |
| Method | Solution |
| Solution | Remeshed Solution 1 |

#### RESIDUAL SCALING

| Description | Value |
| --- | --- |
| Method | Manual |

#### VALUES OF VARIABLES NOT SOLVED FOR

| Description | Value |
| --- | --- |
| Method | Solution |
| Solution | Remeshed Solution 1 |

#### INITIAL VALUE CALCULATION CONSTANTS

| Constant name | Initial value source |
| --- | --- |
| t | range(39*t_cv,t_tot/nb/200,44*t_cv) |
| timestep | 0.0275[s] |

#### LOG

```
<---- Dependent Variables 1 in Study 2 (CV 40 to 44)/Solution 4 (sol4) ---------
Started at Jun 21, 2023 12:18:30 PM.
Initial values of variables solved for: Remeshed Solution 1 (sol3), t=214.5 s [Last]
.
Values of variables not solved for: Remeshed Solution 1 (sol3), t=214.5 s [Last].
Solution time: 0 s.
Physical memory: 2.37 GB
Virtual memory: 2.6 GB
Ended at Jun 21, 2023 12:18:30 PM.
----- Dependent Variables 1 in Study 2 (CV 40 to 44)/Solution 4 (sol4) -------->
```

### Concentration (comp1.cOx) (comp1_cOx)

#### GENERAL

| Description | Value |
|---|---|
| Field components | comp1.cOx |
| Internal variables | {comp1.uflux.cOx, comp1.dflux.cOx, comp1.tds.dt2Inv_cOx} |

### Concentration (comp1.cRed) (comp1_cRed)

#### GENERAL

| Description | Value |
|---|---|
| Field components | comp1.cRed |
| Internal variables | {comp1.uflux.cRed, comp1.dflux.cRed, comp1.tds.dt2Inv_cRed} |

### Spatial mesh displacement (comp1.spatial.disp) (comp1_spatial_disp)

#### GENERAL

| Description | Value |
|---|---|
| Field components | {comp1.spatial.u, comp1.spatial.w} |

#### SCALING

| Description | Value |
|---|---|
| Method | Manual |
| Scale | 2.353429412580713E-7 |

### comp1.RgZg (comp1_RgZg)

#### GENERAL

| Description | Value |
|---|---|
| Field components | {Rg, Zg} |
| Solve for this field | Off |

### Time-Dependent Solver 1 (t1)

#### GENERAL

| Description | Value |
|---|---|

| Description | Value |
| --- | --- |
| Output times | {214.50000000000003, 214.52750000000003, 214.55500000000004, 214.58250000000004, 214.61000000000004, 214.63750000000002, 214.66500000000002, 214.69250000000002, 214.72000000000003, 214.74750000000003, 214.77500000000003, 214.80250000000004, 214.83000000000004, 214.85750000000002, 214.88500000000002, 214.91250000000002, 214.94000000000003, 214.96750000000003, 214.99500000000003, 215.02250000000004, 215.05000000000004, 215.07750000000001, 215.10500000000002, 215.13250000000002, 215.16000000000003, 215.18750000000003, 215.21500000000003, 215.24250000000004, 215.27000000000004, 215.29750000000004, 215.32500000000002, 215.35250000000002, 215.38000000000002, 215.40750000000003, 215.43500000000003, 215.46250000000003, 215.49000000000004, 215.51750000000004, 215.54500000000002, 215.57250000000002, 215.60000000000002, 215.62750000000003, 215.65500000000003, 215.68250000000003, 215.71000000000004, 215.73750000000004, 215.76500000000001, 215.79250000000002, 215.82000000000002, 215.84750000000003, 215.87500000000003, 215.90250000000003, 215.93000000000004, 215.95750000000004, 215.98500000000004, 216.01250000000002, 216.04000000000002, 216.06750000000002, 216.09500000000003, 216.12250000000003, 216.15000000000003, 216.17750000000004, 216.20500000000004, 216.23250000000002, 216.26000000000002, 216.28750000000002, 216.31500000000003, 216.34250000000003, 216.37000000000003, 216.39750000000004, 216.42500000000004, 216.45250000000001, 216.48000000000002, 216.50750000000002, 216.53500000000003, 216.56250000000003, 216.59000000000003, 216.61750000000004, 216.64500000000004, 216.67250000000004, 216.70000000000002, 216.72750000000002, 216.75500000000002, 216.78250000000003, 216.81000000000003, 216.83750000000003, 216.86500000000004, 216.89250000000004, 216.92000000000002, 216.94750000000002, 216.97500000000002, 217.00250000000003, 217.03000000000003, 217.05750000000003, 217.08500000000004, 217.11250000000004, 217.14000000000004, 217.16750000000002, 217.19500000000002, 217.22250000000003, 217.25000000000003, 217.27750000000003, 217.30500000000004, 217.33250000000004, 217.36000000000004, 217.38750000000002, 217.41500000000002, 217.44250000000002, 217.47000000000003, 217.49750000000003, 217.52500000000003, 217.55250000000004, 217.58000000000004, 217.60750000000002, 217.63500000000002, 217.66250000000002, 217.69000000000003, 217.71750000000003, 217.74500000000003, 217.77250000000004, 217.80000000000004, 217.82750000000004, 217.85500000000002, 217.88250000000002, 217.91000000000003, 217.93750000000003, 217.96500000000003, 217.99250000000004, 218.02000000000004, 218.04750000000004, 218.07500000000002, 218.10250000000002, 218.13000000000002, 218.15750000000003, 218.18500000000003, 218.21250000000003, 218.24000000000004, 218.26750000000004, 218.29500000000002, 218.32250000000002, 218.35000000000002, |

| Description | Value |
| --- | --- |
| | 218.37750000000003, 218.40500000000003, 218.43250000000003, 218.46000000000004, 218.48750000000004, 218.51500000000004, 218.54250000000002, 218.57000000000002, 218.59750000000003, 218.62500000000003, 218.65250000000003, 218.68000000000004, 218.70750000000004, 218.73500000000004, 218.76250000000002, 218.79000000000002, 218.81750000000002, 218.84500000000003, 218.87250000000003, 218.90000000000003, 218.92750000000004, 218.95500000000004, 218.98250000000002, 219.01000000000002, 219.03750000000002, 219.06500000000003, 219.09250000000003, 219.12000000000003, 219.14750000000004, 219.17500000000004, 219.20250000000004, 219.23000000000002, 219.25750000000002, 219.28500000000003, 219.31250000000003, 219.34000000000003, 219.36750000000004, 219.39500000000004, 219.42250000000004, 219.45000000000002, 219.47750000000002, 219.50500000000002, 219.53250000000003, 219.56000000000003, 219.58750000000003, 219.61500000000004, 219.64250000000004, 219.67000000000002, 219.69750000000002, 219.72500000000002, 219.75250000000003, 219.78000000000003, 219.80750000000003, 219.83500000000004, 219.86250000000004, 219.89000000000004, 219.91750000000002, 219.94500000000002, 219.97250000000003, 220.00000000000003, 220.02750000000003, 220.05500000000004, 220.08250000000004, 220.11000000000004, 220.13750000000002, 220.16500000000002, 220.19250000000002, 220.22000000000003, 220.24750000000003, 220.27500000000003, 220.30250000000004, 220.33000000000004, 220.35750000000002, 220.38500000000002, 220.41250000000002, 220.44000000000003, 220.46750000000003, 220.49500000000003, 220.52250000000004, 220.55000000000004, 220.57750000000004, 220.60500000000002, 220.63250000000002, 220.66000000000003, 220.68750000000003, 220.71500000000003, 220.74250000000004, 220.77000000000004, 220.79750000000004, 220.82500000000002, 220.85250000000002, 220.88000000000002, 220.90750000000003, 220.93500000000003, 220.96250000000003, 220.99000000000004, 221.01750000000004, 221.04500000000002, 221.07250000000002, 221.10000000000002, 221.12750000000003, 221.15500000000003, 221.18250000000003, 221.21000000000004, 221.23750000000004, 221.26500000000004, 221.29250000000002, 221.32000000000002, 221.34750000000003, 221.37500000000003, 221.40250000000003, 221.43000000000004, 221.45750000000004, 221.48500000000004, 221.51250000000002, 221.54000000000002, 221.56750000000002, 221.59500000000003, 221.62250000000003, 221.65000000000003, 221.67750000000004, 221.70500000000004, 221.73250000000002, 221.76000000000002, 221.78750000000002, 221.81500000000003, 221.84250000000003, 221.87000000000003, 221.89750000000004, 221.92500000000004, 221.95250000000004, 221.98000000000002, 222.00750000000002, 222.03500000000003, 222.06250000000003, 222.09000000000003, 222.11750000000004, 222.14500000000004, 222.17250000000004, 222.20000000000002, 222.22750000000002, |

| Description | Value |
| --- | --- |
| | 222.25500000000002, 222.28250000000003, 222.31000000000003, 222.33750000000003, 222.36500000000004, 222.39250000000004, 222.42000000000002, 222.44750000000002, 222.47500000000002, 222.50250000000003, 222.53000000000003, 222.55750000000003, 222.58500000000004, 222.61250000000004, 222.64000000000004, 222.66750000000002, 222.69500000000002, 222.72250000000003, 222.75000000000003, 222.77750000000003, 222.80500000000004, 222.83250000000004, 222.86000000000004, 222.88750000000002, 222.91500000000002, 222.94250000000002, 222.97000000000003, 222.99750000000003, 223.02500000000003, 223.05250000000004, 223.08000000000004, 223.10750000000002, 223.13500000000002, 223.16250000000002, 223.19000000000003, 223.21750000000003, 223.24500000000003, 223.27250000000004, 223.30000000000004, 223.32750000000004, 223.35500000000002, 223.38250000000002, 223.41000000000003, 223.43750000000003, 223.46500000000003, 223.49250000000004, 223.52000000000004, 223.54750000000004, 223.57500000000002, 223.60250000000002, 223.63000000000002, 223.65750000000003, 223.68500000000003, 223.71250000000003, 223.74000000000004, 223.76750000000004, 223.79500000000002, 223.82250000000002, 223.85000000000002, 223.87750000000003, 223.90500000000003, 223.93250000000003, 223.96000000000004, 223.98750000000004, 224.01500000000004, 224.04250000000002, 224.07000000000002, 224.09750000000003, 224.12500000000003, 224.15250000000003, 224.18000000000004, 224.20750000000004, 224.23500000000004, 224.26250000000002, 224.29000000000002, 224.31750000000002, 224.34500000000003, 224.37250000000003, 224.40000000000003, 224.42750000000004, 224.45500000000004, 224.48250000000002, 224.51000000000002, 224.53750000000002, 224.56500000000003, 224.59250000000003, 224.62000000000003, 224.64750000000004, 224.67500000000004, 224.70250000000004, 224.73000000000002, 224.75750000000002, 224.78500000000003, 224.81250000000003, 224.84000000000003, 224.86750000000004, 224.89500000000004, 224.92250000000004, 224.95000000000002, 224.97750000000002, 225.00500000000002, 225.03250000000003, 225.06000000000003, 225.08750000000003, 225.11500000000004, 225.14250000000004, 225.17000000000002, 225.19750000000002, 225.22500000000002, 225.25250000000003, 225.28000000000003, 225.30750000000003, 225.33500000000004, 225.36250000000004, 225.39000000000004, 225.41750000000002, 225.44500000000002, 225.47250000000003, 225.50000000000003, 225.52750000000003, 225.55500000000004, 225.58250000000004, 225.61000000000004, 225.63750000000002, 225.66500000000002, 225.69250000000002, 225.72000000000003, 225.74750000000003, 225.77500000000003, 225.80250000000004, 225.83000000000004, 225.85750000000002, 225.88500000000002, 225.91250000000002, 225.94000000000003, 225.96750000000003, 225.99500000000003, 226.02250000000004, 226.05000000000004, 226.07750000000004, 226.10500000000002, |

| Description | Value |
| --- | --- |
| | 226.13250000000002, 226.16000000000003, 226.18750000000003, 226.21500000000003, 226.24250000000004, 226.27000000000004, 226.29750000000004, 226.32500000000002, 226.35250000000002, 226.38000000000002, 226.40750000000003, 226.43500000000003, 226.46250000000003, 226.49000000000004, 226.51750000000004, 226.54500000000002, 226.57250000000002, 226.60000000000002, 226.62750000000003, 226.65500000000003, 226.68250000000003, 226.71000000000004, 226.73750000000004, 226.76500000000004, 226.79250000000002, 226.82000000000002, 226.84750000000003, 226.87500000000003, 226.90250000000003, 226.93000000000004, 226.95750000000004, 226.98500000000004, 227.01250000000002, 227.04000000000002, 227.06750000000002, 227.09500000000003, 227.12250000000003, 227.15000000000003, 227.17750000000004, 227.20500000000004, 227.23250000000002, 227.26000000000002, 227.28750000000002, 227.31500000000003, 227.34250000000003, 227.37000000000003, 227.39750000000004, 227.42500000000004, 227.45250000000004, 227.48000000000002, 227.50750000000002, 227.53500000000003, 227.56250000000003, 227.59000000000003, 227.61750000000004, 227.64500000000004, 227.67250000000004, 227.70000000000002, 227.72750000000002, 227.75500000000002, 227.78250000000003, 227.81000000000003, 227.83750000000003, 227.86500000000004, 227.89250000000004, 227.92000000000002, 227.94750000000002, 227.97500000000002, 228.00250000000003, 228.03000000000003, 228.05750000000003, 228.08500000000004, 228.11250000000004, 228.14000000000004, 228.16750000000002, 228.19500000000002, 228.22250000000003, 228.25000000000003, 228.27750000000003, 228.30500000000004, 228.33250000000004, 228.36000000000004, 228.38750000000002, 228.41500000000002, 228.44250000000002, 228.47000000000003, 228.49750000000003, 228.52500000000003, 228.55250000000004, 228.58000000000004, 228.60750000000002, 228.63500000000002, 228.66250000000002, 228.69000000000003, 228.71750000000003, 228.74500000000003, 228.77250000000004, 228.80000000000004, 228.82750000000004, 228.85500000000002, 228.88250000000002, 228.91000000000003, 228.93750000000003, 228.96500000000003, 228.99250000000004, 229.02000000000004, 229.04750000000004, 229.07500000000002, 229.10250000000002, 229.13000000000002, 229.15750000000003, 229.18500000000003, 229.21250000000003, 229.24000000000004, 229.26750000000004, 229.29500000000002, 229.32250000000002, 229.35000000000002, 229.37750000000003, 229.40500000000003, 229.43250000000003, 229.46000000000004, 229.48750000000004, 229.51500000000004, 229.54250000000002, 229.57000000000002, 229.59750000000003, 229.62500000000003, 229.65250000000003, 229.68000000000004, 229.70750000000004, 229.73500000000004, 229.76250000000005, 229.79000000000002, 229.81750000000002, 229.84500000000003, 229.87250000000003, 229.90000000000003, 229.92750000000004, 229.95500000000004, 229.98250000000002, |

| Description | Value |
| --- | --- |
| | 230.01000000000002, 230.03750000000002, 230.06500000000003, 230.09250000000003, 230.12000000000003, 230.14750000000004, 230.17500000000004, 230.20250000000004, 230.23000000000002, 230.25750000000002, 230.28500000000003, 230.31250000000003, 230.34000000000003, 230.36750000000004, 230.39500000000004, 230.42250000000004, 230.45000000000005, 230.47750000000002, 230.50500000000002, 230.53250000000003, 230.56000000000003, 230.58750000000003, 230.61500000000004, 230.64250000000004, 230.67000000000002, 230.69750000000002, 230.72500000000002, 230.75250000000003, 230.78000000000003, 230.80750000000003, 230.83500000000004, 230.86250000000004, 230.89000000000004, 230.91750000000002, 230.94500000000002, 230.97250000000003, 231.00000000000003, 231.02750000000003, 231.05500000000004, 231.08250000000004, 231.11000000000004, 231.13750000000005, 231.16500000000002, 231.19250000000002, 231.22000000000003, 231.24750000000003, 231.27500000000003, 231.30250000000004, 231.33000000000004, 231.35750000000002, 231.38500000000002, 231.41250000000002, 231.44000000000003, 231.46750000000003, 231.49500000000003, 231.52250000000004, 231.55000000000004, 231.57750000000004, 231.60500000000002, 231.63250000000002, 231.66000000000003, 231.68750000000003, 231.71500000000003, 231.74250000000004, 231.77000000000004, 231.79750000000004, 231.82500000000005, 231.85250000000002, 231.88000000000002, 231.90750000000003, 231.93500000000003, 231.96250000000003, 231.99000000000004, 232.01750000000004, 232.04500000000002, 232.07250000000002, 232.10000000000002, 232.12750000000003, 232.15500000000003, 232.18250000000003, 232.21000000000004, 232.23750000000004, 232.26500000000004, 232.29250000000002, 232.32000000000002, 232.34750000000003, 232.37500000000003, 232.40250000000003, 232.43000000000004, 232.45750000000004, 232.48500000000004, 232.51250000000005, 232.54000000000002, 232.56750000000002, 232.59500000000003, 232.62250000000003, 232.65000000000003, 232.67750000000004, 232.70500000000004, 232.73250000000002, 232.76000000000002, 232.78750000000002, 232.81500000000003, 232.84250000000003, 232.87000000000003, 232.89750000000004, 232.92500000000004, 232.95250000000004, 232.98000000000002, 233.00750000000002, 233.03500000000003, 233.06250000000003, 233.09000000000003, 233.11750000000004, 233.14500000000004, 233.17250000000004, 233.20000000000005, 233.22750000000002, 233.25500000000002, 233.28250000000003, 233.31000000000003, 233.33750000000003, 233.36500000000004, 233.39250000000004, 233.42000000000002, 233.44750000000002, 233.47500000000002, 233.50250000000003, 233.53000000000003, 233.55750000000003, 233.58500000000004, 233.61250000000004, 233.64000000000004, 233.66750000000002, 233.69500000000002, 233.72250000000003, 233.75000000000003, 233.77750000000003, 233.80500000000004, 233.83250000000004, 233.86000000000004, |

| Description | Value |
| --- | --- |
| | 233.88750000000005, 233.91500000000002, 233.94250000000002, 233.97000000000003, 233.99750000000003, 234.02500000000003, 234.05250000000004, 234.08000000000004, 234.10750000000002, 234.13500000000002, 234.16250000000002, 234.19000000000003, 234.21750000000003, 234.24500000000003, 234.27250000000004, 234.30000000000004, 234.32750000000004, 234.35500000000002, 234.38250000000002, 234.41000000000003, 234.43750000000003, 234.46500000000003, 234.49250000000004, 234.52000000000004, 234.54750000000004, 234.57500000000005, 234.60250000000002, 234.63000000000002, 234.65750000000003, 234.68500000000003, 234.71250000000003, 234.74000000000004, 234.76750000000004, 234.79500000000002, 234.82250000000002, 234.85000000000002, 234.87750000000003, 234.90500000000003, 234.93250000000003, 234.96000000000004, 234.98750000000004, 235.01500000000004, 235.04250000000002, 235.07000000000002, 235.09750000000003, 235.12500000000003, 235.15250000000003, 235.18000000000004, 235.20750000000004, 235.23500000000004, 235.26250000000005, 235.29000000000002, 235.31750000000002, 235.34500000000003, 235.37250000000003, 235.40000000000003, 235.42750000000004, 235.45500000000004, 235.48250000000002, 235.51000000000002, 235.53750000000002, 235.56500000000003, 235.59250000000003, 235.62000000000003, 235.64750000000004, 235.67500000000004, 235.70250000000004, 235.73000000000002, 235.75750000000002, 235.78500000000003, 235.81250000000003, 235.84000000000003, 235.86750000000004, 235.89500000000004, 235.92250000000004, 235.95000000000005, 235.97750000000002, 236.00500000000002, 236.03250000000003, 236.06000000000003, 236.08750000000003, 236.11500000000004, 236.14250000000004, 236.17000000000002, 236.19750000000002, 236.22500000000002, 236.25250000000003, 236.28000000000003, 236.30750000000003, 236.33500000000004, 236.36250000000004, 236.39000000000004, 236.41750000000002, 236.44500000000002, 236.47250000000003, 236.50000000000003, 236.52750000000003, 236.55500000000004, 236.58250000000004, 236.61000000000004, 236.63750000000005, 236.66500000000002, 236.69250000000002, 236.72000000000003, 236.74750000000003, 236.77500000000003, 236.80250000000004, 236.83000000000004, 236.85750000000002, 236.88500000000002, 236.91250000000002, 236.94000000000003, 236.96750000000003, 236.99500000000003, 237.02250000000004, 237.05000000000004, 237.07750000000004, 237.10500000000002, 237.13250000000002, 237.16000000000003, 237.18750000000003, 237.21500000000003, 237.24250000000004, 237.27000000000004, 237.29750000000004, 237.32500000000005, 237.35250000000002, 237.38000000000002, 237.40750000000003, 237.43500000000003, 237.46250000000003, 237.49000000000004, 237.51750000000004, 237.54500000000002, 237.57250000000002, 237.60000000000002, 237.62750000000003, 237.65500000000003, 237.68250000000003, 237.71000000000004, 237.73750000000004, |

| Description | Value |
| --- | --- |
| | 237.76500000000004, 237.79250000000002, 237.82000000000002, 237.84750000000003, 237.87500000000003, 237.90250000000003, 237.93000000000004, 237.95750000000004, 237.98500000000004, 238.01250000000005, 238.04000000000002, 238.06750000000002, 238.09500000000003, 238.12250000000003, 238.15000000000003, 238.17750000000004, 238.20500000000004, 238.23250000000002, 238.26000000000002, 238.28750000000002, 238.31500000000003, 238.34250000000003, 238.37000000000003, 238.39750000000004, 238.42500000000004, 238.45250000000004, 238.48000000000002, 238.50750000000002, 238.53500000000003, 238.56250000000003, 238.59000000000003, 238.61750000000004, 238.64500000000004, 238.67250000000004, 238.70000000000005, 238.72750000000002, 238.75500000000002, 238.78250000000003, 238.81000000000003, 238.83750000000003, 238.86500000000004, 238.89250000000004, 238.92000000000002, 238.94750000000002, 238.97500000000002, 239.00250000000003, 239.03000000000003, 239.05750000000003, 239.08500000000004, 239.11250000000004, 239.14000000000004, 239.16750000000002, 239.19500000000002, 239.22250000000003, 239.25000000000003, 239.27750000000003, 239.30500000000004, 239.33250000000004, 239.36000000000004, 239.38750000000005, 239.41500000000002, 239.44250000000002, 239.47000000000003, 239.49750000000003, 239.52500000000003, 239.55250000000004, 239.58000000000004, 239.60750000000002, 239.63500000000002, 239.66250000000002, 239.69000000000003, 239.71750000000003, 239.74500000000003, 239.77250000000004, 239.80000000000004, 239.82750000000004, 239.85500000000002, 239.88250000000002, 239.91000000000003, 239.93750000000003, 239.96500000000003, 239.99250000000004, 240.02000000000004, 240.04750000000004, 240.07500000000005, 240.10250000000002, 240.13000000000002, 240.15750000000003, 240.18500000000003, 240.21250000000003, 240.24000000000004, 240.26750000000004, 240.29500000000002, 240.32250000000005, 240.35000000000002, 240.37750000000003, 240.40500000000003, 240.43250000000003, 240.46000000000004, 240.48750000000004, 240.51500000000004, 240.54250000000002, 240.57000000000002, 240.59750000000003, 240.62500000000003, 240.65250000000003, 240.68000000000004, 240.70750000000004, 240.73500000000004, 240.76250000000005, 240.79000000000002, 240.81750000000002, 240.84500000000003, 240.87250000000003, 240.90000000000003, 240.92750000000004, 240.95500000000004, 240.98250000000002, 241.01000000000005, 241.03750000000002, 241.06500000000003, 241.09250000000003, 241.12000000000003, 241.14750000000004, 241.17500000000004, 241.20250000000004, 241.23000000000002, 241.25750000000002, 241.28500000000003, 241.31250000000003, 241.34000000000003, 241.36750000000004, 241.39500000000004, 241.42250000000004, 241.45000000000005, 241.47750000000002, 241.50500000000002, 241.53250000000003, 241.56000000000003, 241.58750000000003, 241.61500000000004, |

| Description | Value |
| --- | --- |
| | 241.64250000000004, 241.67000000000004, 241.69750000000005, 241.72500000000002, 241.75250000000003, 241.78000000000003, 241.80750000000003, 241.83500000000004, 241.86250000000004, 241.89000000000004, 241.91750000000002, 241.94500000000002, 241.97250000000003, 242.00000000000003} |
| Relative tolerance | 0.005 |

### TIME STEPPING

| Description | Value |
| --- | --- |
| Maximum BDF order | 2 |
| Nonlinear controller | On |

### LOG

| | | | | | | | | | | |
|---|---|---|---|---|---|---|---|---|---|---|
| - | 234.52 | - | out | | | | | | | |
| - | 234.55 | - | out | | | | | | | |
| 381 | 234.57 | 0.055 | | 6 | 4 | 6 | 2 | 0 | 0 | 3.7e-13 | 2.6e-15 |
| - | 234.58 | - | out | | | | | | | |
| - | 234.6 | - | out | | | | | | | |
| - | 234.63 | - | out | | | | | | | |
| 382 | 234.64 | 0.070034 | | 10 | 6 | 10 | 2 | 1 | 0 | 1e-13 | 4.8e-15 |
| - | 234.66 | - | out | | | | | | | |
| - | 234.69 | - | out | | | | | | | |
| 383 | 234.71 | 0.070034 | | 12 | 7 | 12 | 2 | 1 | 0 | 4.1e-14 | 6.1e-15 |
| - | 234.71 | - | out | | | | | | | |
| - | 234.74 | - | out | | | | | | | |
| - | 234.77 | - | out | | | | | | | |
| 384 | 234.77 | 0.063031 | | 14 | 8 | 14 | 2 | 1 | 0 | 1.1e-13 | 6.6e-15 |
| - | 234.8 | - | out | | | | | | | |
| - | 234.82 | - | out | | | | | | | |
| 385 | 234.83 | 0.056727 | | 16 | 9 | 16 | 2 | 1 | 0 | 9.1e-14 | 7.6e-15 |
| - | 234.85 | - | out | | | | | | | |
| - | 234.88 | - | out | | | | | | | |
| 386 | 234.88 | 0.056727 | | 18 | 10 | 18 | 1 | 1 | 0 | 2.4e-13 | 3.9e-15 |
| - | 234.91 | - | out | | | | | | | |
| - | 234.93 | - | out | | | | | | | |
| 387 | 234.94 | 0.050733 | | 20 | 11 | 20 | 1 | 1 | 0 | 1.2e-13 | 5.5e-15 |
| - | 234.96 | - | out | | | | | | | |
| 388 | 234.98 | 0.043626 | | 22 | 12 | 22 | 1 | 1 | 0 | 1.6e-13 | 6.2e-15 |
| - | 234.99 | - | out | | | | | | | |
| - | 235.02 | - | out | | | | | | | |
| 389 | 235.02 | 0.038946 | | 24 | 13 | 24 | 1 | 1 | 0 | 1.1e-13 | 6.3e-15 |
| - | 235.04 | - | out | | | | | | | |
| 390 | 235.05 | 0.035052 | | 26 | 14 | 26 | 1 | 1 | 0 | 2e-13 | 6.7e-15 |
| - | 235.07 | - | out | | | | | | | |
| 391 | 235.08 | 0.031547 | | 28 | 15 | 28 | 1 | 1 | 0 | 1.4e-13 | 6.9e-15 |
| - | 235.1 | - | out | | | | | | | |
| 392 | 235.12 | 0.031547 | | 30 | 16 | 30 | 1 | 1 | 0 | 2.7e-13 | 6.4e-15 |
| - | 235.13 | - | out | | | | | | | |
| 393 | 235.15 | 0.031547 | | 32 | 17 | 32 | 1 | 1 | 0 | 7.3e-14 | 6.2e-15 |
| - | 235.15 | - | out | | | | | | | |
| - | 235.18 | - | out | | | | | | | |
| - | 235.21 | - | out | | | | | | | |
| 394 | 235.21 | 0.063093 | | 34 | 18 | 34 | 2 | 1 | 0 | 1.5e-13 | 4.6e-15 |
| - | 235.24 | - | out | | | | | | | |
| - | 235.26 | - | out | | | | | | | |
| 395 | 235.27 | 0.063093 | | 36 | 19 | 36 | 2 | 1 | 0 | 5.7e-13 | 3.2e-15 |
| - | 235.29 | - | out | | | | | | | |
| - | 235.32 | - | out | | | | | | | |
| 396 | 235.34 | 0.063093 | | 38 | 20 | 38 | 2 | 1 | 0 | 3.4e-14 | 9.2e-15 |
| - | 235.35 | - | out | | | | | | | |
| - | 235.37 | - | out | | | | | | | |
| - | 235.4 | - | out | | | | | | | |
| 397 | 235.4 | 0.063093 | | 40 | 21 | 40 | 2 | 1 | 0 | 6.1e-13 | 9e-15 |
| - | 235.43 | - | out | | | | | | | |
| - | 235.46 | - | out | | | | | | | |
| 398 | 235.46 | 0.063093 | | 42 | 22 | 42 | 2 | 1 | 0 | 6.3e-14 | 9.1e-15 |
| - | 235.48 | - | out | | | | | | | |
| - | 235.51 | - | out | | | | | | | |
| 399 | 235.53 | 0.063093 | | 44 | 23 | 44 | 2 | 1 | 0 | 3.2e-13 | 8.7e-15 |

```
   -      235.54           - out
   -      235.57           - out
 400      235.59    0.063093       46   24   46     2     1      0    2e-13  8.6e-15
   -      235.59           - out
   -      235.62           - out
   -      235.65           - out
 401      235.65    0.063093       48   25   48     2     1      0  2.2e-13  8.3e-15
   -      235.65           - out
 402      235.72    0.063093       50   26   50     2     1      0  5.1e-13  6.7e-15
Number of vertex elements: 3
Number of boundary elements: 112
Number of vertex elements: 3
Number of boundary elements: 112
Number of elements: 534
Minimum element quality: 0.5661
Number of vertex elements: 4
Number of boundary elements: 162
Number of vertex elements: 5
Number of boundary elements: 212
Number of vertex elements: 6
Number of boundary elements: 266
Minimum element quality: 0.3716
Geometry shape function: Linear Lagrange
Time interval 7
Time-dependent solver (BDF)
Number of degrees of freedom solved for: 11758 (plus 7124 internal DOFs).
Nonsymmetric matrix found.
Scales for dependent variables:
Concentration (comp1.cOx): 13
Concentration (comp1.cRed): 7.1
Spatial mesh displacement (comp1.spatial.disp): 2.4e-07
Step        Time    Stepsize      Res  Jac  Sol Order Tfail NLfail   LinErr   LinRes
   -      235.65           - out
   -      235.68           - out
 403      235.68      0.0275        2    2    2     1     0      0  1.9e-13  2.6e-15
   -       235.7           - out
 404      235.71      0.0275        4    3    4     1     0      0  6.8e-13  3.6e-15
   -      235.73           - out
   -      235.76           - out
 405      235.76       0.055        6    4    6     2     0      0  1.2e-12  1.4e-15
   -      235.79           - out
   -      235.81           - out
 406      235.82       0.055        8    5    8     2     0      0  8.5e-13    3e-15
   -      235.84           - out
   -      235.87           - out
 407      235.87       0.055       10    6   10     2     0      0  1.5e-12    9e-15
   -       235.9           - out
   -      235.92           - out
 408      235.93       0.055       12    7   12     2     0      0  3.8e-13  9.3e-15
   -      235.95           - out
   -      235.98           - out
 409      235.98       0.055       14    8   14     2     0      0  6.3e-13  8.4e-15
   -      236.01           - out
   -      236.03           - out
 410      236.04       0.055       16    9   16     2     0      0  4.1e-12  8.5e-15
   -      236.06           - out
```

| | | | | | | | | | | |
|---|---|---|---|---|---|---|---|---|---|---|
| - | 236.09 | - out | | | | | | | | |
| 411 | 236.09 | 0.055 | 18 | 10 | 18 | 2 | 0 | 0 | 1.1e-12 | 8.3e-15 |
| - | 236.12 | - out | | | | | | | | |
| - | 236.14 | - out | | | | | | | | |
| 412 | 236.15 | 0.055 | 20 | 11 | 20 | 2 | 0 | 0 | 1.5e-12 | 8.2e-15 |
| - | 236.17 | - out | | | | | | | | |
| - | 236.2 | - out | | | | | | | | |
| 413 | 236.2 | 0.055 | 22 | 12 | 22 | 2 | 0 | 0 | 1.2e-12 | 7.8e-15 |
| - | 236.23 | - out | | | | | | | | |
| - | 236.25 | - out | | | | | | | | |
| 414 | 236.26 | 0.055 | 24 | 13 | 24 | 2 | 0 | 0 | 4.4e-13 | 7.9e-15 |
| - | 236.28 | - out | | | | | | | | |
| - | 236.31 | - out | | | | | | | | |
| 415 | 236.31 | 0.055 | 26 | 14 | 26 | 2 | 0 | 0 | 2.9e-13 | 7.3e-15 |
| - | 236.34 | - out | | | | | | | | |
| - | 236.36 | - out | | | | | | | | |
| 416 | 236.37 | 0.055 | 28 | 15 | 28 | 2 | 0 | 0 | 2.6e-12 | 7.1e-15 |
| - | 236.39 | - out | | | | | | | | |
| - | 236.42 | - out | | | | | | | | |
| 417 | 236.42 | 0.055 | 30 | 16 | 30 | 2 | 0 | 0 | 2e-12 | 6.4e-15 |
| - | 236.45 | - out | | | | | | | | |
| - | 236.47 | - out | | | | | | | | |
| 418 | 236.48 | 0.055 | 32 | 17 | 32 | 2 | 0 | 0 | 5e-13 | 6.2e-15 |
| - | 236.5 | - out | | | | | | | | |
| 419 | 236.5 | 0.023835 | 36 | 19 | 36 | 2 | 1 | 0 | 1.9e-12 | 5.1e-15 |
| 420 | 236.52 | 0.015531 | 40 | 21 | 40 | 2 | 2 | 0 | 1.7e-12 | 6.3e-15 |
| - | 236.53 | - out | | | | | | | | |
| 421 | 236.53 | 0.013761 | 42 | 22 | 42 | 2 | 2 | 0 | 3.1e-12 | 5.3e-15 |
| 422 | 236.54 | 0.012385 | 44 | 23 | 44 | 2 | 2 | 0 | 1.5e-13 | 1.1e-13 |
| - | 236.56 | - out | | | | | | | | |
| 423 | 236.56 | 0.012385 | 46 | 24 | 46 | 2 | 2 | 0 | 2e-13 | 9.9e-14 |
| 424 | 236.57 | 0.012385 | 48 | 25 | 48 | 2 | 2 | 0 | 1.7e-13 | 5.3e-14 |
| 425 | 236.58 | 0.012385 | 50 | 26 | 50 | 2 | 2 | 0 | 4.4e-13 | 1.1e-14 |
| - | 236.58 | - out | | | | | | | | |
| 426 | 236.6 | 0.02477 | 52 | 27 | 52 | 2 | 2 | 0 | 3.2e-13 | 6e-15 |
| - | 236.61 | - out | | | | | | | | |
| - | 236.64 | - out | | | | | | | | |
| 427 | 236.65 | 0.049541 | 54 | 28 | 54 | 2 | 2 | 0 | 3.5e-12 | 4.6e-15 |
| - | 236.67 | - out | | | | | | | | |
| - | 236.69 | - out | | | | | | | | |
| 428 | 236.7 | 0.049541 | 56 | 29 | 56 | 2 | 2 | 0 | 5.2e-12 | 5.4e-15 |
| - | 236.72 | - out | | | | | | | | |
| - | 236.75 | - out | | | | | | | | |
| 429 | 236.75 | 0.049541 | 58 | 30 | 58 | 2 | 2 | 0 | 3.8e-12 | 5.5e-15 |
| - | 236.78 | - out | | | | | | | | |
| - | 236.8 | - out | | | | | | | | |
| 430 | 236.8 | 0.049541 | 60 | 31 | 60 | 2 | 2 | 0 | 6.1e-12 | 5.8e-15 |
| - | 236.83 | - out | | | | | | | | |
| 431 | 236.85 | 0.049541 | 62 | 32 | 62 | 2 | 2 | 0 | 2.2e-13 | 6.3e-15 |
| - | 236.86 | - out | | | | | | | | |
| - | 236.89 | - out | | | | | | | | |
| 432 | 236.9 | 0.049541 | 64 | 33 | 64 | 2 | 2 | 0 | 4e-12 | 6.9e-15 |
| - | 236.91 | - out | | | | | | | | |
| - | 236.94 | - out | | | | | | | | |
| 433 | 236.95 | 0.049541 | 66 | 34 | 66 | 2 | 2 | 0 | 9.9e-13 | 7.1e-15 |
| - | 236.97 | - out | | | | | | | | |

```
   -         237           - out
 434         237    0.049541       68   35   68     2     2       0  2.3e-12  7.3e-15
   -      237.02           - out
   -      237.05           - out
 435      237.05    0.049541       70   36   70     2     2       0  5.1e-13  7.2e-15
   -      237.08           - out
 436       237.1    0.049541       72   37   72     2     2       0  2.7e-13  7.4e-15
   -       237.1           - out
 437      237.15    0.049541       74   38   74     2     2       0  8.2e-13  7.1e-15
Number of vertex elements: 3
Number of boundary elements: 112
Number of vertex elements: 3
Number of boundary elements: 112
Number of elements: 534
Minimum element quality: 0.5661
Number of vertex elements: 4
Number of boundary elements: 162
Number of vertex elements: 5
Number of boundary elements: 212
Number of vertex elements: 6
Number of boundary elements: 266
Minimum element quality: 0.349
Geometry shape function: Linear Lagrange
Time interval 8
Time-dependent solver (BDF)
Number of degrees of freedom solved for: 11758 (plus 7124 internal DOFs).
Nonsymmetric matrix found.
Scales for dependent variables:
Concentration (comp1.cOx): 13
Concentration (comp1.cRed): 5.3
Spatial mesh displacement (comp1.spatial.disp): 2.4e-07
Step        Time    Stepsize      Res  Jac  Sol Order Tfail NLfail   LinErr   LinRes
   -       237.1           - out
   -      237.11           - out
 438      237.13      0.0275        2    2    2     1     0       0  4.8e-13  8.8e-16
   -      237.13           - out
 439      237.16      0.0275        4    3    4     1     0       0  1.3e-12  3.4e-15
   -      237.16           - out
   -      237.19           - out
 440      237.21       0.055        6    4    6     2     0       0  7.8e-13  7.7e-15
   -      237.22           - out
   -      237.24           - out
 441      237.27       0.055        8    5    8     2     0       0  2.2e-13  5.5e-15
   -      237.27           - out
   -       237.3           - out
 442      237.32       0.055       10    6   10     2     0       0    1e-12  7.5e-15
   -      237.33           - out
   -      237.35           - out
 443      237.38       0.055       12    7   12     2     0       0  4.4e-13  7.8e-15
   -      237.38           - out
   -      237.41           - out
 444      237.43       0.055       14    8   14     2     0       0  1.5e-13  7.5e-15
   -      237.44           - out
   -      237.46           - out
 445      237.49       0.055       16    9   16     2     0       0  7.5e-13  7.9e-15
   -      237.49           - out
```

```
   -      237.52           - out
 446      237.54       0.055       18   10   18     2     0      0  1.5e-12    8e-15
   -      237.55           - out
   -      237.57           - out
 447       237.6       0.055       20   11   20     2     0      0  3.9e-13  7.6e-15
   -       237.6           - out
   -      237.63           - out
 448      237.65       0.055       22   12   22     2     0      0  3.7e-13  7.8e-15
   -      237.66           - out
   -      237.68           - out
 449      237.71       0.055       24   13   24     2     0      0  8.4e-13  8.1e-15
   -      237.71           - out
   -      237.74           - out
 450      237.76       0.055       26   14   26     2     0      0  7.4e-13    8e-15
   -      237.77           - out
   -      237.79           - out
 451      237.82       0.055       28   15   28     2     0      0  2.7e-13  7.7e-15
   -      237.82           - out
   -      237.85           - out
 452      237.87       0.055       30   16   30     2     0      0  3.9e-13  7.9e-15
   -      237.88           - out
   -       237.9           - out
 453      237.93       0.055       32   17   32     2     0      0  3.3e-13  8.2e-15
   -      237.93           - out
   -      237.96           - out
 454      237.98       0.055       34   18   34     2     0      0  2.8e-13  7.7e-15
   -      237.99           - out
   -      238.01           - out
 455      238.04       0.055       36   19   36     2     0      0  2.7e-13  7.6e-15
   -      238.04           - out
 456      238.09       0.055       38   20   38     2     0      0  6.2e-13  7.7e-15
Number of vertex elements: 3
Number of boundary elements: 112
Number of vertex elements: 3
Number of boundary elements: 112
Number of elements: 534
Minimum element quality: 0.5661
Number of vertex elements: 4
Number of boundary elements: 162
Number of vertex elements: 5
Number of boundary elements: 212
Number of vertex elements: 6
Number of boundary elements: 266
Minimum element quality: 0.3041
Geometry shape function: Linear Lagrange
Time interval 9
Time-dependent solver (BDF)
Number of degrees of freedom solved for: 11758 (plus 7124 internal DOFs).
Nonsymmetric matrix found.
Scales for dependent variables:
Concentration (comp1.cOx): 26
Concentration (comp1.cRed): 16
Spatial mesh displacement (comp1.spatial.disp): 2.4e-07
Step        Time    Stepsize      Res  Jac  Sol Order Tfail NLfail   LinErr   LinRes
   -      238.04           - out
   -      238.04           - out
```

| | | | | | | | | | | |
|---|---|---|---|---|---|---|---|---|---|---|
| 457 | 238.06 | 0.0275 | | 2 | 2 | 2 | 1 | 0 | 0 | 4.9e-13 | 7.3e-15 |
| - | 238.07 | - | out | | | | | | | | |
| 458 | 238.09 | 0.0275 | | 4 | 3 | 4 | 1 | 0 | 0 | 1.2e-13 | 6.9e-15 |
| - | 238.1 | - | out | | | | | | | | |
| - | 238.12 | - | out | | | | | | | | |
| 459 | 238.15 | 0.055 | | 6 | 4 | 6 | 2 | 0 | 0 | 2.2e-13 | 9e-15 |
| - | 238.15 | - | out | | | | | | | | |
| - | 238.18 | - | out | | | | | | | | |
| 460 | 238.2 | 0.055 | | 8 | 5 | 8 | 2 | 0 | 0 | 2.2e-13 | 1e-14 |
| - | 238.21 | - | out | | | | | | | | |
| - | 238.23 | - | out | | | | | | | | |
| 461 | 238.26 | 0.055 | | 10 | 6 | 10 | 2 | 0 | 0 | 1.7e-13 | 6e-15 |
| - | 238.26 | - | out | | | | | | | | |
| - | 238.29 | - | out | | | | | | | | |
| - | 238.32 | - | out | | | | | | | | |
| - | 238.34 | - | out | | | | | | | | |
| 462 | 238.37 | 0.11 | | 12 | 7 | 12 | 2 | 0 | 0 | 1.5e-13 | 6.2e-15 |
| - | 238.37 | - | out | | | | | | | | |
| - | 238.4 | - | out | | | | | | | | |
| - | 238.43 | - | out | | | | | | | | |
| 463 | 238.44 | 0.073032 | | 16 | 9 | 16 | 2 | 1 | 0 | 3.2e-13 | 6e-15 |
| - | 238.45 | - | out | | | | | | | | |
| - | 238.48 | - | out | | | | | | | | |
| - | 238.51 | - | out | | | | | | | | |
| 464 | 238.51 | 0.073032 | | 18 | 10 | 18 | 2 | 1 | 0 | 4.7e-13 | 4.8e-15 |
| - | 238.54 | - | out | | | | | | | | |
| 465 | 238.55 | 0.043154 | | 22 | 12 | 22 | 1 | 2 | 0 | 1.7e-13 | 4.5e-15 |
| - | 238.56 | - | out | | | | | | | | |
| - | 238.59 | - | out | | | | | | | | |
| 466 | 238.6 | 0.043154 | | 24 | 13 | 24 | 1 | 2 | 0 | 3.3e-13 | 4.6e-15 |
| - | 238.62 | - | out | | | | | | | | |
| 467 | 238.63 | 0.034829 | | 26 | 14 | 26 | 1 | 2 | 0 | 5.4e-13 | 4.4e-15 |
| - | 238.65 | - | out | | | | | | | | |
| 468 | 238.66 | 0.028325 | | 28 | 15 | 28 | 1 | 2 | 0 | 1.6e-13 | 5.2e-15 |
| - | 238.67 | - | out | | | | | | | | |
| 469 | 238.69 | 0.024585 | | 30 | 16 | 30 | 1 | 2 | 0 | 9.3e-14 | 4.1e-15 |
| - | 238.7 | - | out | | | | | | | | |
| 470 | 238.71 | 0.022127 | | 32 | 17 | 32 | 1 | 2 | 0 | 6.8e-14 | 3.5e-15 |
| - | 238.73 | - | out | | | | | | | | |
| 471 | 238.73 | 0.019914 | | 34 | 18 | 34 | 1 | 2 | 0 | 1e-13 | 4e-15 |
| 472 | 238.75 | 0.017923 | | 36 | 19 | 36 | 1 | 2 | 0 | 7.3e-14 | 4.4e-15 |
| - | 238.76 | - | out | | | | | | | | |
| 473 | 238.76 | 0.017923 | | 38 | 20 | 38 | 1 | 2 | 0 | 1.8e-13 | 4.4e-15 |
| 474 | 238.78 | 0.017923 | | 40 | 21 | 40 | 1 | 2 | 0 | 1.1e-13 | 4.9e-15 |
| - | 238.78 | - | out | | | | | | | | |
| - | 238.81 | - | out | | | | | | | | |
| 475 | 238.82 | 0.035845 | | 42 | 22 | 42 | 2 | 2 | 0 | 9.1e-14 | 4.5e-15 |
| - | 238.84 | - | out | | | | | | | | |
| 476 | 238.85 | 0.035845 | | 44 | 23 | 44 | 2 | 2 | 0 | 1.2e-12 | 4.5e-15 |
| - | 238.87 | - | out | | | | | | | | |
| 477 | 238.89 | 0.035845 | | 46 | 24 | 46 | 2 | 2 | 0 | 3.9e-14 | 3.1e-15 |
| - | 238.89 | - | out | | | | | | | | |
| - | 238.92 | - | out | | | | | | | | |
| 478 | 238.92 | 0.035845 | | 48 | 25 | 48 | 2 | 2 | 0 | 4.5e-13 | 3.9e-15 |
| - | 238.95 | - | out | | | | | | | | |
| 479 | 238.96 | 0.035845 | | 50 | 26 | 50 | 2 | 2 | 0 | 1e-13 | 3.9e-15 |

| | | | | | | | | | | |
|---|---|---|---|---|---|---|---|---|---|---|
| - | 238.98 | - | out | | | | | | | |
| 480 | 239 | 0.035845 | 52 | 27 | 52 | 2 | 2 | 0 | 5e-13 | 2.9e-15 |
| - | 239 | - | out | | | | | | | |
| - | 239.03 | - | out | | | | | | | |
| 481 | 239.03 | 0.035845 | 54 | 28 | 54 | 2 | 2 | 0 | 1.7e-13 | 2.9e-15 |
| - | 239.06 | - | out | | | | | | | |
| 482 | 239.07 | 0.035845 | 56 | 29 | 56 | 2 | 2 | 0 | 5.5e-13 | 4e-15 |
| - | 239.09 | - | out | | | | | | | |
| 483 | 239.1 | 0.032261 | 58 | 30 | 58 | 2 | 2 | 0 | 1.2e-12 | 4.9e-15 |
| - | 239.11 | - | out | | | | | | | |
| 484 | 239.13 | 0.032261 | 60 | 31 | 60 | 2 | 2 | 0 | 9.5e-13 | 3.3e-15 |
| - | 239.14 | - | out | | | | | | | |
| 485 | 239.16 | 0.032261 | 62 | 32 | 62 | 2 | 2 | 0 | 4.3e-13 | 4.4e-15 |
| - | 239.17 | - | out | | | | | | | |
| - | 239.2 | - | out | | | | | | | |
| 486 | 239.2 | 0.032261 | 64 | 33 | 64 | 2 | 2 | 0 | 3.1e-13 | 4.6e-15 |
| - | 239.22 | - | out | | | | | | | |
| 487 | 239.23 | 0.032261 | 66 | 34 | 66 | 2 | 2 | 0 | 1.4e-12 | 3e-15 |
| 488 | 239.24 | 0.014301 | 70 | 36 | 70 | 2 | 3 | 0 | 2.9e-13 | 3e-15 |
| - | 239.25 | - | out | | | | | | | |
| 489 | 239.25 | 0.0083336 | 75 | 38 | 75 | 2 | 4 | 0 | 2.1e-12 | 6.3e-15 |
| 490 | 239.26 | 0.0049804 | 79 | 40 | 79 | 2 | 5 | 0 | 5.5e-13 | 2e-14 |
| 491 | 239.26 | 0.0049804 | 81 | 41 | 81 | 2 | 5 | 0 | 2.8e-13 | 1.7e-14 |
| 492 | 239.27 | 0.0044824 | 83 | 42 | 83 | 2 | 5 | 0 | 2.8e-14 | 4.7e-14 |
| 493 | 239.27 | 0.0044824 | 85 | 43 | 85 | 1 | 5 | 0 | 3e-13 | 4e-15 |
| - | 239.28 | - | out | | | | | | | |
| 494 | 239.28 | 0.0089648 | 87 | 44 | 87 | 1 | 5 | 0 | 4.2e-13 | 2.7e-15 |
| 495 | 239.29 | 0.0089648 | 89 | 45 | 89 | 1 | 5 | 0 | 2.9e-13 | 4.2e-15 |
| 496 | 239.3 | 0.0089648 | 91 | 46 | 91 | 1 | 5 | 0 | 2.7e-13 | 3.7e-15 |
| - | 239.31 | - | out | | | | | | | |
| 497 | 239.32 | 0.01793 | 93 | 47 | 93 | 2 | 5 | 0 | 1.3e-13 | 2.9e-15 |
| - | 239.33 | - | out | | | | | | | |
| 498 | 239.33 | 0.01793 | 95 | 48 | 95 | 2 | 5 | 0 | 5.9e-13 | 3.2e-15 |
| 499 | 239.35 | 0.01793 | 97 | 49 | 97 | 2 | 5 | 0 | 1.2e-12 | 4.8e-15 |
| - | 239.36 | - | out | | | | | | | |
| 500 | 239.37 | 0.01793 | 99 | 50 | 99 | 2 | 5 | 0 | 5e-13 | 4.5e-15 |
| 501 | 239.39 | 0.01793 | 101 | 51 | 101 | 2 | 5 | 0 | 2.1e-13 | 4.4e-15 |
| - | 239.39 | - | out | | | | | | | |
| 502 | 239.41 | 0.01793 | 103 | 52 | 103 | 2 | 5 | 0 | 4.3e-13 | 3.1e-15 |
| - | 239.42 | - | out | | | | | | | |
| 503 | 239.42 | 0.01793 | 105 | 53 | 105 | 2 | 5 | 0 | 9.2e-14 | 3.1e-15 |
| 504 | 239.44 | 0.01793 | 107 | 54 | 107 | 2 | 5 | 0 | 2.8e-13 | 2.9e-15 |
| - | 239.44 | - | out | | | | | | | |
| 505 | 239.46 | 0.01793 | 109 | 55 | 109 | 2 | 5 | 0 | 3.5e-13 | 5.7e-15 |
| - | 239.47 | - | out | | | | | | | |
| 506 | 239.48 | 0.01793 | 111 | 56 | 111 | 2 | 5 | 0 | 3.3e-13 | 4.7e-15 |
| 507 | 239.5 | 0.01793 | 113 | 57 | 113 | 2 | 5 | 0 | 3.1e-13 | 4.6e-15 |
| - | 239.5 | - | out | | | | | | | |
| 508 | 239.51 | 0.01793 | 115 | 58 | 115 | 2 | 5 | 0 | 3e-13 | 3.7e-15 |
| - | 239.53 | - | out | | | | | | | |
| 509 | 239.53 | 0.01793 | 117 | 59 | 117 | 2 | 5 | 0 | 1.7e-13 | 4.1e-15 |
| 510 | 239.55 | 0.01793 | 119 | 60 | 119 | 2 | 5 | 0 | 1.4e-13 | 4.3e-15 |
| - | 239.55 | - | out | | | | | | | |
| 511 | 239.57 | 0.01793 | 121 | 61 | 121 | 2 | 5 | 0 | 3.4e-13 | 3.8e-15 |
| - | 239.58 | - | out | | | | | | | |
| 512 | 239.58 | 0.01793 | 123 | 62 | 123 | 2 | 5 | 0 | 3.4e-13 | 3.8e-15 |

| | | | | | | | | | | |
|---|---|---|---|---|---|---|---|---|---|---|
| 513 | 239.6 | 0.01793 | 125 | 63 | 125 | 2 | 5 | 0 | 9.3e-14 | 3.6e-15 |
| - | 239.61 | - out | | | | | | | | |
| 514 | 239.62 | 0.01793 | 127 | 64 | 127 | 2 | 5 | 0 | 2.8e-13 | 3.7e-15 |
| - | 239.64 | - out | | | | | | | | |
| 515 | 239.64 | 0.01793 | 129 | 65 | 129 | 2 | 5 | 0 | 3.3e-13 | 3.3e-15 |
| - | 239.66 | - out | | | | | | | | |
| 516 | 239.67 | 0.035859 | 131 | 66 | 131 | 2 | 5 | 0 | 5.8e-13 | 3.9e-15 |
| - | 239.69 | - out | | | | | | | | |
| 517 | 239.71 | 0.035859 | 133 | 67 | 133 | 2 | 5 | 0 | 3.8e-13 | 4.9e-15 |
| - | 239.72 | - out | | | | | | | | |
| - | 239.75 | - out | | | | | | | | |
| 518 | 239.75 | 0.035859 | 135 | 68 | 135 | 2 | 5 | 0 | 8.3e-14 | 4.9e-15 |
| - | 239.77 | - out | | | | | | | | |
| 519 | 239.78 | 0.035859 | 137 | 69 | 137 | 2 | 5 | 0 | 3.4e-13 | 4.1e-15 |
| - | 239.8 | - out | | | | | | | | |
| 520 | 239.82 | 0.035859 | 139 | 70 | 139 | 2 | 5 | 0 | 3.4e-13 | 5e-15 |
| - | 239.83 | - out | | | | | | | | |
| 521 | 239.85 | 0.035859 | 141 | 71 | 141 | 2 | 5 | 0 | 1.4e-13 | 4.5e-15 |
| - | 239.86 | - out | | | | | | | | |
| - | 239.88 | - out | | | | | | | | |
| - | 239.91 | - out | | | | | | | | |
| 522 | 239.93 | 0.071718 | 143 | 72 | 143 | 2 | 5 | 0 | 1.7e-12 | 4.6e-15 |
| - | 239.94 | - out | | | | | | | | |
| - | 239.97 | - out | | | | | | | | |
| - | 239.99 | - out | | | | | | | | |
| 523 | 240 | 0.071718 | 145 | 73 | 145 | 2 | 5 | 0 | 3.3e-13 | 5.5e-15 |
| - | 240.02 | - out | | | | | | | | |
| - | 240.05 | - out | | | | | | | | |
| 524 | 240.06 | 0.064546 | 147 | 74 | 147 | 2 | 5 | 0 | 1.1e-12 | 6e-15 |
| - | 240.08 | - out | | | | | | | | |
| - | 240.1 | - out | | | | | | | | |
| 525 | 240.12 | 0.058092 | 149 | 75 | 149 | 2 | 5 | 0 | 2.2e-12 | 6.3e-15 |
| - | 240.13 | - out | | | | | | | | |
| - | 240.16 | - out | | | | | | | | |
| 526 | 240.18 | 0.058092 | 151 | 76 | 151 | 1 | 5 | 0 | 4.4e-13 | 5.1e-15 |
| - | 240.19 | - out | | | | | | | | |
| - | 240.21 | - out | | | | | | | | |
| 527 | 240.24 | 0.058092 | 153 | 77 | 153 | 1 | 5 | 0 | 6.5e-14 | 6.4e-15 |
| - | 240.24 | - out | | | | | | | | |
| - | 240.27 | - out | | | | | | | | |
| 528 | 240.28 | 0.048992 | 155 | 78 | 155 | 1 | 5 | 0 | 1.6e-12 | 6.5e-15 |
| - | 240.3 | - out | | | | | | | | |
| - | 240.32 | - out | | | | | | | | |
| 529 | 240.33 | 0.04124 | 157 | 79 | 157 | 1 | 5 | 0 | 1.7e-13 | 6.6e-15 |
| - | 240.35 | - out | | | | | | | | |
| 530 | 240.36 | 0.037116 | 159 | 80 | 159 | 1 | 5 | 0 | 2.3e-13 | 6.5e-15 |
| - | 240.36 | - out | | | | | | | | |
| 531 | 240.4 | 0.033404 | 161 | 81 | 161 | 1 | 5 | 0 | 2.1e-12 | 6.8e-15 |

Number of vertex elements: 3
Number of boundary elements: 112
Number of vertex elements: 3
Number of boundary elements: 112
Number of elements: 534
Minimum element quality: 0.5661
Number of vertex elements: 4
Number of boundary elements: 162

```
Number of vertex elements: 5
Number of boundary elements: 212
Number of vertex elements: 6
Number of boundary elements: 266
Minimum element quality: 0.1707
Geometry shape function: Linear Lagrange
Time interval 10
Time-dependent solver (BDF)
Number of degrees of freedom solved for: 11758 (plus 7124 internal DOFs).
Nonsymmetric matrix found.
Scales for dependent variables:
Concentration (comp1.cOx): 45
Concentration (comp1.cRed): 31
Spatial mesh displacement (comp1.spatial.disp): 2.4e-07
Step        Time    Stepsize      Res  Jac  Sol Order Tfail NLfail   LinErr   LinRes
   -      240.36           - out
   -      240.38           - out
 532      240.39      0.0275        2    2    2     1     0      0  3.6e-13  5.4e-15
   -      240.41           - out
 533      240.42      0.0275        4    3    4     1     0      0  2.2e-13  6.6e-15
   -      240.43           - out
   -      240.46           - out
 534      240.47       0.055        6    4    6     2     0      0  1.2e-12  6.9e-15
   -      240.49           - out
   -      240.52           - out
 535      240.53       0.055        8    5    8     2     0      0  3.6e-12  9.4e-15
   -      240.54           - out
   -      240.57           - out
 536      240.58       0.055       10    6   10     2     0      0  5.2e-13  9.3e-15
   -       240.6           - out
   -      240.63           - out
 537      240.64       0.055       12    7   12     2     0      0  2.7e-12  9.3e-15
   -      240.65           - out
   -      240.68           - out
 538      240.69       0.055       14    8   14     2     0      0  4.2e-12  9.5e-15
   -      240.71           - out
   -      240.74           - out
 539      240.75       0.055       16    9   16     2     0      0  5.2e-12    1e-14
   -      240.76           - out
   -      240.79           - out
 540       240.8       0.055       18   10   18     2     0      0  3.5e-12  9.6e-15
   -      240.82           - out
   -      240.85           - out
 541      240.86       0.055       20   11   20     2     0      0  6.4e-12    1e-14
   -      240.87           - out
   -       240.9           - out
 542      240.91       0.055       22   12   22     2     0      0  5.5e-12  1.2e-14
   -      240.93           - out
   -      240.96           - out
 543      240.97       0.055       24   13   24     2     0      0  2.2e-12  1.3e-14
   -      240.98           - out
   -      241.01           - out
 544      241.02       0.055       26   14   26     2     0      0  1.3e-11  1.5e-14
   -      241.04           - out
   -      241.07           - out
 545      241.08       0.055       28   15   28     2     0      0  9.2e-12  1.8e-14
```

```
   -      241.09          - out
   -      241.12          - out
 546      241.13       0.055       30   16   30     2     0      0  1.1e-11  2.2e-14
   -      241.15          - out
   -      241.18          - out
 547      241.19       0.055       32   17   32     2     0      0  1.9e-11  2.7e-14
   -       241.2          - out
   -      241.23          - out
 548      241.24       0.055       34   18   34     2     0      0  1.1e-11  3.8e-14
   -      241.26          - out
   -      241.29          - out
 549       241.3       0.055       36   19   36     2     0      0  2.5e-11    5e-14
   -      241.31          - out
   -      241.34          - out
 550      241.35       0.055       38   20   38     2     0      0  1.3e-11  6.7e-14
   -      241.37          - out
   -       241.4          - out
 551      241.41       0.055       40   21   40     2     0      0  2.8e-11    1e-13
   -      241.41          - out
 552      241.46       0.055       42   22   42     2     0      0  4.2e-11  1.3e-13
Number of vertex elements: 3
Number of boundary elements: 112
Number of vertex elements: 3
Number of boundary elements: 112
Number of elements: 534
Minimum element quality: 0.5661
Number of vertex elements: 4
Number of boundary elements: 162
Number of vertex elements: 5
Number of boundary elements: 212
Number of vertex elements: 6
Number of boundary elements: 266
Minimum element quality: 0.04508
Geometry shape function: Linear Lagrange
Time interval 11
Time-dependent solver (BDF)
Number of degrees of freedom solved for: 11758 (plus 7124 internal DOFs).
Nonsymmetric matrix found.
Scales for dependent variables:
Concentration (comp1.cOx): 69
Concentration (comp1.cRed): 19
Spatial mesh displacement (comp1.spatial.disp): 2.4e-07
Step        Time    Stepsize      Res  Jac  Sol Order Tfail NLfail   LinErr   LinRes
   -      241.41          - out
   -      241.42          - out
 553      241.44      0.0275        2    2    2     1     0      0  2.6e-11  2.3e-13
   -      241.45          - out
 554      241.46      0.0275        4    3    4     1     0      0  2.7e-11  2.5e-13
   -      241.48          - out
   -      241.51          - out
 555      241.52       0.055        6    4    6     2     0      0  5.6e-11  1.6e-13
   -      241.53          - out
   -      241.56          - out
 556      241.57       0.055        8    5    8     2     0      0  1.2e-10  4.7e-13
   -      241.57          - out
 557      241.59     0.01375       11    6   10     2     0      1  1.4e-11  2.2e-13
```

```
Number of vertex elements: 3
Number of boundary elements: 112
Number of vertex elements: 3
Number of boundary elements: 112
Number of elements: 534
Minimum element quality: 0.5661
Number of vertex elements: 4
Number of boundary elements: 162
Number of vertex elements: 5
Number of boundary elements: 212
Number of vertex elements: 6
Number of boundary elements: 266
Minimum element quality: 0.0002336
Geometry shape function: Linear Lagrange
Time interval 12
Time-dependent solver (BDF)
Number of degrees of freedom solved for: 11758 (plus 7124 internal DOFs).
Nonsymmetric matrix found.
Scales for dependent variables:
Concentration (comp1.cOx): 97
Concentration (comp1.cRed): 23
Spatial mesh displacement (comp1.spatial.disp): 2.4e-07
Step        Time    Stepsize      Res  Jac  Sol Order Tfail NLfail   LinErr   LinRes
   -      241.57           - out
   -      241.59           - out
 558       241.6      0.0275        2    2    2     1     0      0  9.7e-11  3.9e-13
 559      241.61    0.006875        5    3    4     1     0      1  6.9e-11  2.7e-13
   -      241.61           - out
 560      241.61   0.0059813        8    4    5     1     0      3  7.3e-11  3.7e-13
Number of vertex elements: 3
Number of boundary elements: 112
Number of vertex elements: 3
Number of boundary elements: 112
Number of elements: 534
Minimum element quality: 0.5661
Number of vertex elements: 4
Number of boundary elements: 162
Number of vertex elements: 5
Number of boundary elements: 212
Number of vertex elements: 6
Number of boundary elements: 266
Minimum element quality: 0.5661
Solution time: 494 s. (8 minutes, 14 seconds)
Physical memory: 2.6 GB
Virtual memory: 2.8 GB
Ended at Jun 21, 2023 12:26:44 PM.
----- Time-Dependent Solver 1 in Study 2 (CV 40 to 44)/Solution 4 (sol4) ------>
```

#### Advanced (aDef)

##### ASSEMBLY SETTINGS

| Description | Value |
| --- | --- |
| Reuse sparsity pattern | On |

### Fully Coupled 1 (fc1)

#### GENERAL

| Description | Value |
|---|---|
| Linear solver | Direct 1 |

#### METHOD AND TERMINATION

| Description | Value |
|---|---|
| Damping factor | 0.9 |
| Jacobian update | Once per time step |
| Maximum number of iterations | 8 |
| Stabilization and acceleration | Anderson acceleration |
| Dimension of iteration space | 5 |

### Automatic Remeshing 1 (ar1)

#### GENERAL

| Description | Value |
|---|---|
| Remesh in geometry | Geometry 1 |

#### CONDITION FOR REMESHING

| Description | Value |
|---|---|
| Condition type | Distortion |
| Stop when distortion exceeds | 1.5 |

#### OUTPUT

| Description | Value |
|---|---|
| Solution | Remeshed Solution 2 |
| Meshes | {mesh11, mesh12, mesh13, mesh14, mesh15, mesh16, mesh17, mesh18, mesh19, mesh20, mesh21, mesh22} |

# 5 Study 3 (CV 21 to 30)

COMPUTATION INFORMATION

| | |
|---|---|
| Computation time | 11 min 46 s |

## 5.1 TIME DEPENDENT

| Times | Unit |
|---|---|
| range(20*t_cv,t_tot/nb/200,30*t_cv) | s |

STUDY SETTINGS

| Description | Value |
|---|---|
| Include geometric nonlinearity | Off |

STUDY SETTINGS

| Description | Value |
|---|---|
| Output times | {110.00000000000001, 110.02750000000002, 110.05500000000002, 110.08250000000001, 110.11000000000001, 110.13750000000002, 110.16500000000002, 110.19250000000001, 110.22000000000001, 110.24750000000002, 110.27500000000002, 110.30250000000001, 110.33000000000001, 110.35750000000002, 110.38500000000002, 110.41250000000001, 110.44000000000001, 110.46750000000002, 110.49500000000002, 110.52250000000001, 110.55000000000001, 110.57750000000001, 110.60500000000002, 110.63250000000001, 110.66000000000001, 110.68750000000001, 110.71500000000002, 110.74250000000002, 110.77000000000001, 110.79750000000001, 110.82500000000002, 110.85250000000002, 110.88000000000001, 110.90750000000001, 110.93500000000002, 110.96250000000002, 110.99000000000001, 111.01750000000001, 111.04500000000002, 111.07250000000002, 111.10000000000001, 111.12750000000001, 111.15500000000002, 111.18250000000002, 111.21000000000001, 111.23750000000001, 111.26500000000001, 111.29250000000002, 111.32000000000002, 111.34750000000001, 111.37500000000001, 111.40250000000002, 111.43000000000002, 111.45750000000001, 111.48500000000001, 111.51250000000002, 111.54000000000002, 111.56750000000001, 111.59500000000001, 111.62250000000002, 111.65000000000002, 111.67750000000001, 111.70500000000001, 111.73250000000002, 111.76000000000002, 111.78750000000001, 111.81500000000001, 111.84250000000002, 111.87000000000002, 111.89750000000001, 111.92500000000001, 111.95250000000001, 111.98000000000002, 112.00750000000002, 112.03500000000001, 112.06250000000001, 112.09000000000002, 112.11750000000002, 112.14500000000001, 112.17250000000001, 112.20000000000002, 112.22750000000002, 112.25500000000001, 112.28250000000001, 112.31000000000002, 112.33750000000002, 112.36500000000001, 112.39250000000001, 112.42000000000002, 112.44750000000002, 112.47500000000001, 112.50250000000001, 112.53000000000002, |

| Description | Value |
|---|---|
| | 112.55750000000002, 112.58500000000001, 112.61250000000001, 112.64000000000001, 112.66750000000002, 112.69500000000002, 112.72250000000001, 112.75000000000001, 112.77750000000002, 112.80500000000002, 112.83250000000001, 112.86000000000001, 112.88750000000002, 112.91500000000002, 112.94250000000001, 112.97000000000001, 112.99750000000002, 113.02500000000002, 113.05250000000001, 113.08000000000001, 113.10750000000002, 113.13500000000002, 113.16250000000001, 113.19000000000001, 113.21750000000002, 113.24500000000002, 113.27250000000001, 113.30000000000001, 113.32750000000001, 113.35500000000002, 113.38250000000002, 113.41000000000001, 113.43750000000001, 113.46500000000002, 113.49250000000002, 113.52000000000001, 113.54750000000001, 113.57500000000002, 113.60250000000002, 113.63000000000001, 113.65750000000001, 113.68500000000002, 113.71250000000002, 113.74000000000001, 113.76750000000001, 113.79500000000002, 113.82250000000002, 113.85000000000001, 113.87750000000001, 113.90500000000002, 113.93250000000002, 113.96000000000001, 113.98750000000001, 114.01500000000001, 114.04250000000002, 114.07000000000002, 114.09750000000001, 114.12500000000001, 114.15250000000002, 114.18000000000002, 114.20750000000001, 114.23500000000001, 114.26250000000002, 114.29000000000002, 114.31750000000001, 114.34500000000001, 114.37250000000002, 114.40000000000002, 114.42750000000001, 114.45500000000001, 114.48250000000002, 114.51000000000002, 114.53750000000001, 114.56500000000001, 114.59250000000002, 114.62000000000002, 114.64750000000001, 114.67500000000001, 114.70250000000001, 114.73000000000002, 114.75750000000002, 114.78500000000001, 114.81250000000001, 114.84000000000002, 114.86750000000002, 114.89500000000001, 114.92250000000001, 114.95000000000002, 114.97750000000002, 115.00500000000001, 115.03250000000001, 115.06000000000002, 115.08750000000002, 115.11500000000001, 115.14250000000001, 115.17000000000002, 115.19750000000002, 115.22500000000001, 115.25250000000001, 115.28000000000002, 115.30750000000002, 115.33500000000001, 115.36250000000001, 115.39000000000001, 115.41750000000002, 115.44500000000002, 115.47250000000001, 115.50000000000001, 115.52750000000002, 115.55500000000002, 115.58250000000001, 115.61000000000001, 115.63750000000002, 115.66500000000002, 115.69250000000001, 115.72000000000001, 115.74750000000002, 115.77500000000002, 115.80250000000001, 115.83000000000001, 115.85750000000002, 115.88500000000002, 115.91250000000001, 115.94000000000001, 115.96750000000002, 115.99500000000002, 116.02250000000001, 116.05000000000001, 116.07750000000001, 116.10500000000002, 116.13250000000002, 116.16000000000001, 116.18750000000001, 116.21500000000002, 116.24250000000002, 116.27000000000001, 116.29750000000001, 116.32500000000002, 116.35250000000002, 116.38000000000001, 116.40750000000001, 116.43500000000002, 116.46250000000002, 116.49000000000001, |

| Description | Value |
| --- | --- |
| | 116.51750000000001, 116.54500000000002, 116.57250000000002, 116.60000000000001, 116.62750000000001, 116.65500000000002, 116.68250000000002, 116.71000000000001, 116.73750000000001, 116.76500000000001, 116.79250000000002, 116.82000000000002, 116.84750000000001, 116.87500000000001, 116.90250000000002, 116.93000000000002, 116.95750000000001, 116.98500000000001, 117.01250000000002, 117.04000000000002, 117.06750000000001, 117.09500000000001, 117.12250000000002, 117.15000000000002, 117.17750000000001, 117.20500000000001, 117.23250000000002, 117.26000000000002, 117.28750000000001, 117.31500000000001, 117.34250000000002, 117.37000000000002, 117.39750000000001, 117.42500000000001, 117.45250000000001, 117.48000000000002, 117.50750000000002, 117.53500000000001, 117.56250000000001, 117.59000000000002, 117.61750000000002, 117.64500000000001, 117.67250000000001, 117.70000000000002, 117.72750000000002, 117.75500000000001, 117.78250000000001, 117.81000000000002, 117.83750000000002, 117.86500000000001, 117.89250000000001, 117.92000000000002, 117.94750000000002, 117.97500000000002, 118.00250000000001, 118.03000000000002, 118.05750000000002, 118.08500000000001, 118.11250000000001, 118.14000000000001, 118.16750000000002, 118.19500000000002, 118.22250000000001, 118.25000000000001, 118.27750000000002, 118.30500000000002, 118.33250000000001, 118.36000000000001, 118.38750000000002, 118.41500000000002, 118.44250000000001, 118.47000000000001, 118.49750000000002, 118.52500000000002, 118.55250000000001, 118.58000000000001, 118.60750000000002, 118.63500000000002, 118.66250000000002, 118.69000000000001, 118.71750000000002, 118.74500000000002, 118.77250000000001, 118.80000000000001, 118.82750000000001, 118.85500000000002, 118.88250000000002, 118.91000000000001, 118.93750000000001, 118.96500000000002, 118.99250000000002, 119.02000000000001, 119.04750000000001, 119.07500000000002, 119.10250000000002, 119.13000000000001, 119.15750000000001, 119.18500000000002, 119.21250000000002, 119.24000000000001, 119.26750000000001, 119.29500000000002, 119.32250000000002, 119.35000000000002, 119.37750000000001, 119.40500000000002, 119.43250000000002, 119.46000000000001, 119.48750000000001, 119.51500000000001, 119.54250000000002, 119.57000000000002, 119.59750000000001, 119.62500000000001, 119.65250000000002, 119.68000000000002, 119.70750000000001, 119.73500000000001, 119.76250000000002, 119.79000000000002, 119.81750000000001, 119.84500000000001, 119.87250000000002, 119.90000000000002, 119.92750000000001, 119.95500000000001, 119.98250000000002, 120.01000000000002, 120.03750000000002, 120.06500000000001, 120.09250000000002, 120.12000000000002, 120.14750000000001, 120.17500000000001, 120.20250000000001, 120.23000000000002, 120.25750000000002, 120.28500000000001, 120.31250000000001, 120.34000000000002, 120.36750000000002, 120.39500000000001, 120.42250000000001, 120.45000000000002, |

| Description | Value |
| --- | --- |
| | 120.47750000000002, 120.50500000000001, 120.53250000000001, 120.56000000000002, 120.58750000000002, 120.61500000000001, 120.64250000000001, 120.67000000000002, 120.69750000000002, 120.72500000000002, 120.75250000000001, 120.78000000000002, 120.80750000000002, 120.83500000000001, 120.86250000000001, 120.89000000000001, 120.91750000000002, 120.94500000000002, 120.97250000000001, 121.00000000000001, 121.02750000000002, 121.05500000000002, 121.08250000000001, 121.11000000000001, 121.13750000000002, 121.16500000000002, 121.19250000000001, 121.22000000000001, 121.24750000000002, 121.27500000000002, 121.30250000000001, 121.33000000000001, 121.35750000000002, 121.38500000000002, 121.41250000000002, 121.44000000000001, 121.46750000000002, 121.49500000000002, 121.52250000000001, 121.55000000000001, 121.57750000000001, 121.60500000000002, 121.63250000000002, 121.66000000000001, 121.68750000000001, 121.71500000000002, 121.74250000000002, 121.77000000000001, 121.79750000000001, 121.82500000000002, 121.85250000000002, 121.88000000000001, 121.90750000000001, 121.93500000000002, 121.96250000000002, 121.99000000000001, 122.01750000000001, 122.04500000000002, 122.07250000000002, 122.10000000000002, 122.12750000000001, 122.15500000000002, 122.18250000000002, 122.21000000000001, 122.23750000000001, 122.26500000000001, 122.29250000000002, 122.32000000000002, 122.34750000000001, 122.37500000000001, 122.40250000000002, 122.43000000000002, 122.45750000000001, 122.48500000000001, 122.51250000000002, 122.54000000000002, 122.56750000000001, 122.59500000000001, 122.62250000000002, 122.65000000000002, 122.67750000000001, 122.70500000000001, 122.73250000000002, 122.76000000000002, 122.78750000000002, 122.81500000000001, 122.84250000000002, 122.87000000000002, 122.89750000000001, 122.92500000000001, 122.95250000000001, 122.98000000000002, 123.00750000000002, 123.03500000000001, 123.06250000000001, 123.09000000000002, 123.11750000000002, 123.14500000000001, 123.17250000000001, 123.20000000000002, 123.22750000000002, 123.25500000000002, 123.28250000000001, 123.31000000000002, 123.33750000000002, 123.36500000000001, 123.39250000000001, 123.42000000000002, 123.44750000000002, 123.47500000000002, 123.50250000000001, 123.53000000000002, 123.55750000000002, 123.58500000000002, 123.61250000000001, 123.64000000000001, 123.66750000000002, 123.69500000000002, 123.72250000000001, 123.75000000000001, 123.77750000000002, 123.80500000000002, 123.83250000000001, 123.86000000000001, 123.88750000000002, 123.91500000000002, 123.94250000000002, 123.97000000000001, 123.99750000000002, 124.02500000000002, 124.05250000000001, 124.08000000000001, 124.10750000000002, 124.13500000000002, 124.16250000000002, 124.19000000000001, 124.21750000000002, 124.24500000000002, 124.27250000000002, 124.30000000000001, 124.32750000000001, 124.35500000000002, 124.38250000000002, 124.41000000000001, |

| Description | Value |
| --- | --- |
| | 124.43750000000001, 124.46500000000002, 124.49250000000002, 124.52000000000001, 124.54750000000001, 124.57500000000002, 124.60250000000002, 124.63000000000002, 124.65750000000001, 124.68500000000002, 124.71250000000002, 124.74000000000001, 124.76750000000001, 124.79500000000002, 124.82250000000002, 124.85000000000002, 124.87750000000001, 124.90500000000002, 124.93250000000002, 124.96000000000002, 124.98750000000001, 125.01500000000001, 125.04250000000002, 125.07000000000002, 125.09750000000001, 125.12500000000001, 125.15250000000002, 125.18000000000002, 125.20750000000001, 125.23500000000001, 125.26250000000002, 125.29000000000002, 125.31750000000002, 125.34500000000001, 125.37250000000002, 125.40000000000002, 125.42750000000001, 125.45500000000001, 125.48250000000002, 125.51000000000002, 125.53750000000002, 125.56500000000001, 125.59250000000002, 125.62000000000002, 125.64750000000002, 125.67500000000001, 125.70250000000001, 125.73000000000002, 125.75750000000002, 125.78500000000001, 125.81250000000001, 125.84000000000002, 125.86750000000002, 125.89500000000001, 125.92250000000001, 125.95000000000002, 125.97750000000002, 126.00500000000002, 126.03250000000001, 126.06000000000002, 126.08750000000002, 126.11500000000001, 126.14250000000001, 126.17000000000002, 126.19750000000002, 126.22500000000002, 126.25250000000001, 126.28000000000002, 126.30750000000002, 126.33500000000001, 126.36250000000001, 126.39000000000001, 126.41750000000002, 126.44500000000002, 126.47250000000003, 126.50000000000001, 126.52750000000002, 126.55500000000002, 126.58250000000001, 126.61000000000001, 126.63750000000002, 126.66500000000002, 126.69250000000002, 126.72000000000001, 126.74750000000002, 126.77500000000002, 126.80250000000001, 126.83000000000001, 126.85750000000002, 126.88500000000002, 126.91250000000002, 126.94000000000001, 126.96750000000002, 126.99500000000002, 127.02250000000001, 127.05000000000001, 127.07750000000001, 127.10500000000002, 127.13250000000002, 127.16000000000003, 127.18750000000001, 127.21500000000002, 127.24250000000002, 127.27000000000001, 127.29750000000001, 127.32500000000002, 127.35250000000002, 127.38000000000002, 127.40750000000001, 127.43500000000002, 127.46250000000002, 127.49000000000001, 127.51750000000001, 127.54500000000002, 127.57250000000002, 127.60000000000002, 127.62750000000001, 127.65500000000002, 127.68250000000002, 127.71000000000001, 127.73750000000001, 127.76500000000001, 127.79250000000002, 127.82000000000002, 127.84750000000003, 127.87500000000001, 127.90250000000002, 127.93000000000002, 127.95750000000001, 127.98500000000001, 128.01250000000002, 128.04000000000002, 128.06750000000002, 128.09500000000003, 128.1225, 128.15, 128.1775, 128.205, 128.23250000000002, 128.26000000000002, 128.28750000000002, 128.31500000000003, 128.34250000000003, 128.37, 128.3975, 128.425, 128.45250000000001, 128.48000000000002, 128.50750000000002, |

| Description | Value |
|---|---|
| | 128.53500000000003, 128.56250000000003, 128.59000000000003, 128.6175, 128.645, 128.6725, 128.70000000000002, 128.72750000000002, 128.75500000000002, 128.78250000000003, 128.81, 128.8375, 128.865, 128.8925, 128.92000000000002, 128.94750000000002, 128.97500000000002, 129.00250000000003, 129.03000000000003, 129.0575, 129.085, 129.1125, 129.14000000000001, 129.16750000000002, 129.19500000000002, 129.22250000000003, 129.25000000000003, 129.27750000000003, 129.305, 129.3325, 129.36, 129.38750000000002, 129.41500000000002, 129.44250000000002, 129.47000000000003, 129.4975, 129.525, 129.5525, 129.58, 129.60750000000002, 129.63500000000002, 129.66250000000002, 129.69000000000003, 129.71750000000003, 129.745, 129.7725, 129.8, 129.82750000000001, 129.85500000000002, 129.88250000000002, 129.91000000000003, 129.93750000000003, 129.96500000000003, 129.9925, 130.02, 130.0475, 130.07500000000002, 130.10250000000002, 130.13000000000002, 130.15750000000003, 130.185, 130.2125, 130.24, 130.2675, 130.29500000000002, 130.32250000000002, 130.35000000000002, 130.37750000000003, 130.40500000000003, 130.4325, 130.46, 130.4875, 130.51500000000001, 130.54250000000002, 130.57000000000002, 130.59750000000003, 130.62500000000003, 130.65250000000003, 130.68, 130.7075, 130.735, 130.76250000000002, 130.79000000000002, 130.81750000000002, 130.84500000000003, 130.8725, 130.9, 130.9275, 130.955, 130.98250000000002, 131.01000000000002, 131.03750000000002, 131.06500000000003, 131.09250000000003, 131.12, 131.1475, 131.175, 131.20250000000001, 131.23000000000002, 131.25750000000002, 131.28500000000003, 131.31250000000003, 131.34000000000003, 131.3675, 131.395, 131.4225, 131.45000000000002, 131.47750000000002, 131.50500000000002, 131.53250000000003, 131.56, 131.5875, 131.615, 131.6425, 131.67000000000002, 131.69750000000002, 131.72500000000002, 131.75250000000003, 131.78000000000003, 131.8075, 131.835, 131.8625, 131.89000000000001, 131.91750000000002, 131.94500000000002, 131.97250000000003, 132.00000000000003, 132.02750000000003, 132.055, 132.0825, 132.11, 132.13750000000002, 132.16500000000002, 132.19250000000002, 132.22000000000003, 132.2475, 132.275, 132.3025, 132.33, 132.35750000000002, 132.38500000000002, 132.41250000000002, 132.44000000000003, 132.46750000000003, 132.495, 132.5225, 132.55, 132.57750000000001, 132.60500000000002, 132.63250000000002, 132.66000000000003, 132.68750000000003, 132.71500000000003, 132.7425, 132.77, 132.7975, 132.82500000000002, 132.85250000000002, 132.88000000000002, 132.90750000000003, 132.935, 132.9625, 132.99, 133.0175, 133.04500000000002, 133.07250000000002, 133.10000000000002, 133.12750000000003, 133.15500000000003, 133.1825, 133.21, 133.2375, 133.26500000000001, 133.29250000000002, 133.32000000000002, 133.34750000000003, 133.37500000000003, 133.40250000000003, 133.43, 133.4575, 133.485, 133.51250000000002, 133.54000000000002, 133.56750000000002, 133.59500000000003, 133.6225, 133.65, 133.6775, 133.705, 133.73250000000002, 133.76000000000002, 133.78750000000002, 133.81500000000003, 133.84250000000003, 133.87, 133.8975, 133.925, 133.95250000000001, 133.98000000000002, 134.00750000000002, 134.03500000000003, |

| Description | Value |
| --- | --- |
| | 134.06250000000003, 134.09000000000003, 134.1175, 134.145, 134.1725, 134.20000000000002, 134.22750000000002, 134.25500000000002, 134.28250000000003, 134.31, 134.3375, 134.365, 134.3925, 134.42000000000002, 134.44750000000002, 134.47500000000002, 134.50250000000003, 134.53000000000003, 134.5575, 134.585, 134.6125, 134.64000000000001, 134.66750000000002, 134.69500000000002, 134.72250000000003, 134.75000000000003, 134.77750000000003, 134.805, 134.8325, 134.86, 134.88750000000002, 134.91500000000002, 134.94250000000002, 134.97000000000003, 134.9975, 135.025, 135.0525, 135.08, 135.10750000000002, 135.13500000000002, 135.16250000000002, 135.19000000000003, 135.21750000000003, 135.245, 135.2725, 135.3, 135.32750000000001, 135.35500000000002, 135.38250000000002, 135.41000000000003, 135.43750000000003, 135.46500000000003, 135.4925, 135.52, 135.5475, 135.57500000000002, 135.60250000000002, 135.63000000000002, 135.65750000000003, 135.685, 135.7125, 135.74, 135.7675, 135.79500000000002, 135.82250000000002, 135.85000000000002, 135.87750000000003, 135.90500000000003, 135.9325, 135.96, 135.9875, 136.01500000000001, 136.04250000000002, 136.07000000000002, 136.09750000000003, 136.12500000000003, 136.15250000000003, 136.18, 136.2075, 136.235, 136.26250000000002, 136.29000000000002, 136.31750000000002, 136.34500000000003, 136.3725, 136.4, 136.4275, 136.455, 136.48250000000002, 136.51000000000002, 136.53750000000002, 136.56500000000003, 136.59250000000003, 136.62, 136.6475, 136.675, 136.70250000000001, 136.73000000000002, 136.75750000000002, 136.78500000000003, 136.81250000000003, 136.84000000000003, 136.8675, 136.895, 136.9225, 136.95000000000002, 136.97750000000002, 137.00500000000002, 137.03250000000003, 137.06, 137.0875, 137.115, 137.1425, 137.17000000000002, 137.19750000000002, 137.22500000000002, 137.25250000000003, 137.28000000000003, 137.3075, 137.335, 137.3625, 137.39000000000001, 137.41750000000002, 137.44500000000002, 137.47250000000003, 137.50000000000003, 137.52750000000003, 137.555, 137.5825, 137.61, 137.63750000000002, 137.66500000000002, 137.69250000000002, 137.72000000000003, 137.7475, 137.775, 137.8025, 137.83, 137.85750000000002, 137.88500000000002, 137.91250000000002, 137.94000000000003, 137.96750000000003, 137.995, 138.0225, 138.05, 138.07750000000001, 138.10500000000002, 138.13250000000002, 138.16000000000003, 138.18750000000003, 138.21500000000003, 138.2425, 138.27, 138.2975, 138.32500000000002, 138.35250000000002, 138.38000000000002, 138.40750000000003, 138.435, 138.4625, 138.49, 138.5175, 138.54500000000002, 138.57250000000002, 138.60000000000002, 138.62750000000003, 138.65500000000003, 138.6825, 138.71, 138.7375, 138.76500000000001, 138.79250000000002, 138.82000000000002, 138.84750000000003, 138.87500000000003, 138.90250000000003, 138.93, 138.9575, 138.985, 139.01250000000002, 139.04000000000002, 139.06750000000002, 139.09500000000003, 139.1225, 139.15, 139.1775, 139.205, 139.23250000000002, 139.26000000000002, 139.28750000000002, 139.31500000000003, 139.34250000000003, 139.37, 139.3975, 139.425, 139.45250000000001, 139.48000000000002, 139.50750000000002, 139.53500000000003, |

| Description | Value |
| --- | --- |
| | 139.56250000000003, 139.59000000000003, 139.6175, 139.645, 139.6725, 139.70000000000002, 139.72750000000002, 139.75500000000002, 139.78250000000003, 139.81, 139.83750000000003, 139.865, 139.8925, 139.92000000000002, 139.94750000000002, 139.97500000000002, 140.00250000000003, 140.03000000000003, 140.0575, 140.085, 140.1125, 140.14000000000001, 140.16750000000002, 140.19500000000002, 140.22250000000003, 140.25000000000003, 140.27750000000003, 140.305, 140.3325, 140.36, 140.38750000000002, 140.41500000000002, 140.44250000000002, 140.47000000000003, 140.4975, 140.52500000000003, 140.5525, 140.58, 140.60750000000002, 140.63500000000002, 140.66250000000002, 140.69000000000003, 140.71750000000003, 140.745, 140.7725, 140.8, 140.82750000000001, 140.85500000000002, 140.88250000000002, 140.91000000000003, 140.93750000000003, 140.96500000000003, 140.9925, 141.02, 141.0475, 141.07500000000002, 141.10250000000002, 141.13000000000002, 141.15750000000003, 141.18500000000003, 141.21250000000003, 141.24, 141.2675, 141.29500000000002, 141.32250000000002, 141.35000000000002, 141.37750000000003, 141.40500000000003, 141.4325, 141.46, 141.4875, 141.51500000000001, 141.54250000000002, 141.57000000000002, 141.59750000000003, 141.62500000000003, 141.65250000000003, 141.68, 141.7075, 141.735, 141.76250000000002, 141.79000000000002, 141.81750000000002, 141.84500000000003, 141.87250000000003, 141.90000000000003, 141.9275, 141.955, 141.98250000000002, 142.01000000000002, 142.03750000000002, 142.06500000000003, 142.09250000000003, 142.12, 142.1475, 142.175, 142.20250000000001, 142.23000000000002, 142.25750000000002, 142.28500000000003, 142.31250000000003, 142.34000000000003, 142.3675, 142.395, 142.4225, 142.45000000000002, 142.47750000000002, 142.50500000000002, 142.53250000000003, 142.56, 142.58750000000003, 142.615, 142.6425, 142.67000000000002, 142.69750000000002, 142.72500000000002, 142.75250000000003, 142.78000000000003, 142.8075, 142.835, 142.8625, 142.89000000000001, 142.91750000000002, 142.94500000000002, 142.97250000000003, 143.00000000000003, 143.02750000000003, 143.055, 143.0825, 143.11, 143.13750000000002, 143.16500000000002, 143.19250000000002, 143.22000000000003, 143.2475, 143.27500000000003, 143.3025, 143.33, 143.35750000000002, 143.38500000000002, 143.41250000000002, 143.44000000000003, 143.46750000000003, 143.495, 143.5225, 143.55, 143.57750000000001, 143.60500000000002, 143.63250000000002, 143.66000000000003, 143.68750000000003, 143.71500000000003, 143.7425, 143.77, 143.7975, 143.82500000000002, 143.85250000000002, 143.88000000000002, 143.90750000000003, 143.935, 143.96250000000003, 143.99, 144.0175, 144.04500000000002, 144.07250000000002, 144.10000000000002, 144.12750000000003, 144.15500000000003, 144.1825, 144.21000000000004, 144.2375, 144.26500000000001, 144.29250000000002, 144.32000000000002, 144.34750000000003, 144.37500000000003, 144.40250000000003, 144.43, 144.4575, 144.485, 144.51250000000002, 144.54000000000002, 144.56750000000002, 144.59500000000003, 144.6225, 144.65000000000003, 144.6775, 144.705, 144.73250000000002, 144.76000000000002, 144.78750000000002, 144.81500000000003, 144.84250000000003, 144.87, 144.89750000000004, 144.925, 144.95250000000001, 144.98000000000002, 145.00750000000002, |

| Description | Value |
| --- | --- |
| | 145.03500000000003, 145.06250000000003, 145.09000000000003, 145.1175, 145.145, 145.1725, 145.20000000000002, 145.22750000000002, 145.25500000000002, 145.28250000000003, 145.31, 145.33750000000003, 145.365, 145.3925, 145.42000000000002, 145.44750000000002, 145.47500000000002, 145.50250000000003, 145.53000000000003, 145.5575, 145.58500000000004, 145.6125, 145.64000000000001, 145.66750000000002, 145.69500000000002, 145.72250000000003, 145.75000000000003, 145.77750000000003, 145.805, 145.8325, 145.86, 145.88750000000002, 145.91500000000002, 145.94250000000002, 145.97000000000003, 145.9975, 146.02500000000003, 146.0525, 146.08, 146.10750000000002, 146.13500000000002, 146.16250000000002, 146.19000000000003, 146.21750000000003, 146.245, 146.27250000000004, 146.3, 146.32750000000001, 146.35500000000002, 146.38250000000002, 146.41000000000003, 146.43750000000003, 146.46500000000003, 146.4925, 146.52, 146.5475, 146.57500000000002, 146.60250000000002, 146.63000000000002, 146.65750000000003, 146.685, 146.71250000000003, 146.74, 146.7675, 146.79500000000002, 146.82250000000002, 146.85000000000002, 146.87750000000003, 146.90500000000003, 146.9325, 146.96000000000004, 146.9875, 147.01500000000001, 147.04250000000002, 147.07000000000002, 147.09750000000003, 147.12500000000003, 147.15250000000003, 147.18, 147.2075, 147.235, 147.26250000000002, 147.29000000000002, 147.31750000000002, 147.34500000000003, 147.3725, 147.40000000000003, 147.4275, 147.455, 147.48250000000002, 147.51000000000002, 147.53750000000002, 147.56500000000003, 147.59250000000003, 147.62, 147.64750000000004, 147.675, 147.70250000000001, 147.73000000000002, 147.75750000000002, 147.78500000000003, 147.81250000000003, 147.84000000000003, 147.8675, 147.895, 147.9225, 147.95000000000002, 147.97750000000002, 148.00500000000002, 148.03250000000003, 148.06, 148.08750000000003, 148.115, 148.1425, 148.17000000000002, 148.19750000000002, 148.22500000000002, 148.25250000000003, 148.28000000000003, 148.3075, 148.33500000000004, 148.3625, 148.39000000000001, 148.41750000000002, 148.44500000000002, 148.47250000000003, 148.50000000000003, 148.52750000000003, 148.555, 148.5825, 148.61, 148.63750000000002, 148.66500000000002, 148.69250000000002, 148.72000000000003, 148.7475, 148.77500000000003, 148.8025, 148.83, 148.85750000000002, 148.88500000000002, 148.91250000000002, 148.94000000000003, 148.96750000000003, 148.995, 149.02250000000004, 149.05, 149.07750000000001, 149.10500000000002, 149.13250000000002, 149.16000000000003, 149.18750000000003, 149.21500000000003, 149.2425, 149.27, 149.2975, 149.32500000000002, 149.35250000000002, 149.38000000000002, 149.40750000000003, 149.435, 149.46250000000003, 149.49, 149.5175, 149.54500000000002, 149.57250000000002, 149.60000000000002, 149.62750000000003, 149.65500000000003, 149.6825, 149.71000000000004, 149.7375, 149.76500000000001, 149.79250000000002, 149.82000000000002, 149.84750000000003, 149.87500000000003, 149.90250000000003, 149.93, 149.9575, 149.985, 150.01250000000002, 150.04000000000002, 150.06750000000002, 150.09500000000003, 150.1225, 150.15000000000003, 150.1775, 150.205, 150.23250000000002, 150.26000000000002, 150.28750000000002, 150.31500000000003, 150.34250000000003, 150.37, 150.39750000000004, 150.425, 150.45250000000001, 150.48000000000002, 150.50750000000002, |

| Description | Value |
| --- | --- |
| | 150.53500000000003, 150.56250000000003, 150.59000000000003, 150.6175, 150.645, 150.6725, 150.70000000000002, 150.72750000000002, 150.75500000000002, 150.78250000000003, 150.81, 150.83750000000003, 150.865, 150.8925, 150.92000000000002, 150.94750000000002, 150.97500000000002, 151.00250000000003, 151.03000000000003, 151.0575, 151.08500000000004, 151.1125, 151.14000000000001, 151.16750000000002, 151.19500000000002, 151.22250000000003, 151.25000000000003, 151.27750000000003, 151.305, 151.3325, 151.36, 151.38750000000002, 151.41500000000002, 151.44250000000002, 151.47000000000003, 151.4975, 151.52500000000003, 151.5525, 151.58, 151.60750000000002, 151.63500000000002, 151.66250000000002, 151.69000000000003, 151.71750000000003, 151.745, 151.77250000000004, 151.8, 151.82750000000001, 151.85500000000002, 151.88250000000002, 151.91000000000003, 151.93750000000003, 151.96500000000003, 151.9925, 152.02, 152.0475, 152.07500000000002, 152.10250000000002, 152.13000000000002, 152.15750000000003, 152.185, 152.21250000000003, 152.24, 152.2675, 152.29500000000002, 152.32250000000002, 152.35000000000002, 152.37750000000003, 152.40500000000003, 152.4325, 152.46000000000004, 152.4875, 152.51500000000001, 152.54250000000002, 152.57000000000002, 152.59750000000003, 152.62500000000003, 152.65250000000003, 152.68, 152.7075, 152.735, 152.76250000000002, 152.79000000000002, 152.81750000000002, 152.84500000000003, 152.8725, 152.90000000000003, 152.9275, 152.955, 152.98250000000002, 153.01000000000002, 153.03750000000002, 153.06500000000003, 153.09250000000003, 153.12, 153.14750000000004, 153.175, 153.20250000000001, 153.23000000000002, 153.25750000000002, 153.28500000000003, 153.31250000000003, 153.34000000000003, 153.3675, 153.395, 153.4225, 153.45000000000002, 153.47750000000002, 153.50500000000002, 153.53250000000003, 153.56, 153.58750000000003, 153.615, 153.6425, 153.67000000000002, 153.69750000000002, 153.72500000000002, 153.75250000000003, 153.78000000000003, 153.8075, 153.83500000000004, 153.8625, 153.89000000000001, 153.91750000000002, 153.94500000000002, 153.97250000000003, 154.00000000000003, 154.02750000000003, 154.055, 154.0825, 154.11, 154.13750000000002, 154.16500000000002, 154.19250000000002, 154.22000000000003, 154.2475, 154.27500000000003, 154.3025, 154.33, 154.35750000000002, 154.38500000000002, 154.41250000000002, 154.44000000000003, 154.46750000000003, 154.495, 154.52250000000004, 154.55, 154.57750000000001, 154.60500000000002, 154.63250000000002, 154.66000000000003, 154.68750000000003, 154.71500000000003, 154.7425, 154.77, 154.7975, 154.82500000000002, 154.85250000000002, 154.88000000000002, 154.90750000000003, 154.93500000000003, 154.96250000000003, 154.99, 155.0175, 155.04500000000002, 155.07250000000002, 155.10000000000002, 155.12750000000003, 155.15500000000003, 155.1825, 155.21000000000004, 155.2375, 155.26500000000001, 155.29250000000002, 155.32000000000002, 155.34750000000003, 155.37500000000003, 155.40250000000003, 155.43, 155.4575, 155.485, 155.51250000000002, 155.54000000000002, 155.56750000000002, 155.59500000000003, 155.62250000000003, 155.65000000000003, 155.6775, 155.705, 155.73250000000002, 155.76000000000002, 155.78750000000002, 155.81500000000003, 155.84250000000003, 155.87, 155.89750000000004, 155.925, 155.95250000000001, 155.98000000000002, 156.00750000000002, |

| Description | Value |
|---|---|
| | 156.03500000000003, 156.06250000000003, 156.09000000000003, 156.1175, 156.145, 156.1725, 156.20000000000002, 156.22750000000002, 156.25500000000002, 156.28250000000003, 156.31000000000003, 156.33750000000003, 156.365, 156.3925, 156.42000000000002, 156.44750000000002, 156.47500000000002, 156.50250000000003, 156.53000000000003, 156.5575, 156.58500000000004, 156.6125, 156.64000000000001, 156.66750000000002, 156.69500000000002, 156.72250000000003, 156.75000000000003, 156.77750000000003, 156.805, 156.8325, 156.86, 156.88750000000002, 156.91500000000002, 156.94250000000002, 156.97000000000003, 156.99750000000003, 157.02500000000003, 157.0525, 157.08, 157.10750000000002, 157.13500000000002, 157.16250000000002, 157.19000000000003, 157.21750000000003, 157.245, 157.27250000000004, 157.3, 157.32750000000001, 157.35500000000002, 157.38250000000002, 157.41000000000003, 157.43750000000003, 157.46500000000003, 157.4925, 157.52, 157.5475, 157.57500000000002, 157.60250000000002, 157.63000000000002, 157.65750000000003, 157.68500000000003, 157.71250000000003, 157.74, 157.7675, 157.79500000000002, 157.82250000000002, 157.85000000000002, 157.87750000000003, 157.90500000000003, 157.9325, 157.96000000000004, 157.9875, 158.01500000000001, 158.04250000000002, 158.07000000000002, 158.09750000000003, 158.12500000000003, 158.15250000000003, 158.18, 158.2075, 158.235, 158.26250000000002, 158.29000000000002, 158.31750000000002, 158.34500000000003, 158.37250000000003, 158.40000000000003, 158.4275, 158.455, 158.48250000000002, 158.51000000000002, 158.53750000000002, 158.56500000000003, 158.59250000000003, 158.62, 158.64750000000004, 158.675, 158.70250000000001, 158.73000000000002, 158.75750000000002, 158.78500000000003, 158.81250000000003, 158.84000000000003, 158.8675, 158.895, 158.9225, 158.95000000000002, 158.97750000000002, 159.00500000000002, 159.03250000000003, 159.06000000000003, 159.08750000000003, 159.115, 159.1425, 159.17000000000002, 159.19750000000002, 159.22500000000002, 159.25250000000003, 159.28000000000003, 159.3075, 159.33500000000004, 159.3625, 159.39000000000001, 159.41750000000002, 159.44500000000002, 159.47250000000003, 159.50000000000003, 159.52750000000003, 159.555, 159.5825, 159.61, 159.63750000000002, 159.66500000000002, 159.69250000000002, 159.72000000000003, 159.74750000000003, 159.77500000000003, 159.8025, 159.83, 159.85750000000002, 159.88500000000002, 159.91250000000002, 159.94000000000003, 159.96750000000003, 159.995, 160.02250000000004, 160.05, 160.07750000000001, 160.10500000000002, 160.13250000000002, 160.16000000000003, 160.18750000000003, 160.21500000000003, 160.2425, 160.27, 160.2975, 160.32500000000002, 160.35250000000002, 160.38000000000002, 160.40750000000003, 160.43500000000003, 160.46250000000003, 160.49, 160.5175, 160.54500000000002, 160.57250000000002, 160.60000000000002, 160.62750000000003, 160.65500000000003, 160.6825, 160.71000000000004, 160.7375, 160.76500000000001, 160.79250000000002, 160.82000000000002, 160.84750000000003, 160.87500000000003, 160.90250000000003, 160.93, 160.95750000000004, 160.985, 161.01250000000002, 161.04000000000002, 161.06750000000002, 161.09500000000003, 161.12250000000003, 161.15000000000003, 161.1775, 161.205, 161.23250000000002, 161.26000000000002, 161.28750000000002, 161.31500000000003, 161.34250000000003, 161.37, 161.39750000000004, 161.425, 161.45250000000001, 161.48000000000002, |

| Description | Value |
| --- | --- |
| | 161.50750000000002, 161.53500000000003, 161.56250000000003, 161.59000000000003, 161.6175, 161.64500000000004, 161.6725, 161.70000000000002, 161.72750000000002, 161.75500000000002, 161.78250000000003, 161.81000000000003, 161.83750000000003, 161.865, 161.8925, 161.92000000000002, 161.94750000000002, 161.97500000000002, 162.00250000000003, 162.03000000000003, 162.0575, 162.08500000000004, 162.1125, 162.14000000000001, 162.16750000000002, 162.19500000000002, 162.22250000000003, 162.25000000000003, 162.27750000000003, 162.305, 162.33250000000004, 162.36, 162.38750000000002, 162.41500000000002, 162.44250000000002, 162.47000000000003, 162.49750000000003, 162.52500000000003, 162.5525, 162.58, 162.60750000000002, 162.63500000000002, 162.66250000000002, 162.69000000000003, 162.71750000000003, 162.745, 162.77250000000004, 162.8, 162.82750000000001, 162.85500000000002, 162.88250000000002, 162.91000000000003, 162.93750000000003, 162.96500000000003, 162.9925, 163.02000000000004, 163.0475, 163.07500000000002, 163.10250000000002, 163.13000000000002, 163.15750000000003, 163.18500000000003, 163.21250000000003, 163.24, 163.2675, 163.29500000000002, 163.32250000000002, 163.35000000000002, 163.37750000000003, 163.40500000000003, 163.4325, 163.46000000000004, 163.4875, 163.51500000000001, 163.54250000000002, 163.57000000000002, 163.59750000000003, 163.62500000000003, 163.65250000000003, 163.68, 163.70750000000004, 163.735, 163.76250000000002, 163.79000000000002, 163.81750000000002, 163.84500000000003, 163.87250000000003, 163.90000000000003, 163.9275, 163.955, 163.98250000000002, 164.01000000000002, 164.03750000000002, 164.06500000000003, 164.09250000000003, 164.12, 164.14750000000004, 164.175, 164.20250000000001, 164.23000000000002, 164.25750000000002, 164.28500000000003, 164.31250000000003, 164.34000000000003, 164.3675, 164.39500000000004, 164.4225, 164.45000000000002, 164.47750000000002, 164.50500000000002, 164.53250000000003, 164.56000000000003, 164.58750000000003, 164.615, 164.6425, 164.67000000000002, 164.69750000000002, 164.72500000000002, 164.75250000000003, 164.78000000000003, 164.8075, 164.83500000000004, 164.8625, 164.89000000000001, 164.91750000000002, 164.94500000000002, 164.97250000000003, 165.00000000000003} |

### VALUES OF DEPENDENT VARIABLES

| Description | Value |
| --- | --- |
| Settings | User controlled |
| Method | Solution |
| Study | Study 2 (CV 40 to 44) |
| Settings | User controlled |
| Method | Solution |
| Study | Study 2 (CV 40 to 44) |

### MESH

| Feature | Value |
| --- | --- |

| Feature | Value |
| --- | --- |
| Geometry 1 | mesh1 |

#### PHYSICS AND VARIABLES SELECTION

| Physics interface | Discretization |
| --- | --- |
| Transport of Diluted Species in droplet (tds) | physics |
| Moving Mesh (ale) | physics |

#### MESH SELECTION

| Geometry | Mesh |
| --- | --- |
| Geometry 1 (geom1) | mesh1 |

## 5.2 SOLVER CONFIGURATIONS

### 5.2.1 Solution 6

**Compile Equations: Time Dependent (st1)**

#### STUDY AND STEP

| Description | Value |
| --- | --- |
| Use study | Study 3 (CV 21 to 30) |
| Use study step | Time Dependent |

#### LOG

```
<---- Compile Equations: Time Dependent in Study 3 (CV 21 to 30)/Solution 6
      (sol6) -----------------------------------------------------------------
Started at May 31, 2023 10:51:37 AM.
Geometry shape function: Linear Lagrange
Running on Intel64 Family 6 Model 158 Stepping 10, GenuineIntel.
Using 1 socket with 6 cores in total on LAPTOP-2492RD8I.
Available memory: 32.27 GB.
Time: 2 s.
Physical memory: 2.42 GB
Virtual memory: 2.5 GB
Ended at May 31, 2023 10:51:38 AM.
----- Compile Equations: Time Dependent in Study 3 (CV 21 to 30)/Solution 6
      (sol6) ---------------------------------------------------------------->
```

**Dependent Variables 1 (v1)**

#### GENERAL

| Description | Value |
| --- | --- |
| Defined by study step | Time Dependent |

#### INITIAL VALUES OF VARIABLES SOLVED FOR

| Description | Value |
| --- | --- |

| Description | Value |
| --- | --- |
| Method | Solution |
| Solution | Remeshed Solution 2 |

### RESIDUAL SCALING

| Description | Value |
| --- | --- |
| Method | Manual |

### VALUES OF VARIABLES NOT SOLVED FOR

| Description | Value |
| --- | --- |
| Method | Solution |
| Solution | Remeshed Solution 2 |

### INITIAL VALUE CALCULATION CONSTANTS

| Constant name | Initial value source |
| --- | --- |
| t | range(20*t_cv,t_tot/nb/200,30*t_cv) |
| timestep | 0.055000000000000014[s] |

### LOG

```
<---- Dependent Variables 1 in Study 3 (CV 21 to 30)/Solution 6 (sol6) ---------
Started at May 31, 2023 10:51:38 AM.
Initial values of variables solved for: Remeshed Solution 2 (sol5), t=110 s [Last].
Values of variables not solved for: Remeshed Solution 2 (sol5), t=110 s [Last].
Solution time: 0 s.
Physical memory: 2.42 GB
Virtual memory: 2.5 GB
Ended at May 31, 2023 10:51:39 AM.
----- Dependent Variables 1 in Study 3 (CV 21 to 30)/Solution 6 (sol6) -------->
```

**Concentration (comp1.cOx) (comp1_cOx)**

### GENERAL

| Description | Value |
| --- | --- |
| Field components | comp1.cOx |
| Internal variables | {comp1.uflux.cOx, comp1.dflux.cOx} |

**Concentration (comp1.cRed) (comp1_cRed)**

### GENERAL

| Description | Value |
| --- | --- |
| Field components | comp1.cRed |
| Internal variables | {comp1.uflux.cRed, comp1.dflux.cRed} |

**comp1.comp1.RgZg (comp1_comp1_RgZg)**

### GENERAL

| Description | Value |
| --- | --- |
| Field components | {Rg, Zg} |
| Solve for this field | Off |

#### Spatial mesh displacement (comp1.spatial.disp) (comp1_spatial_disp)

GENERAL

| Description | Value |
| --- | --- |
| Field components | {comp1.spatial.u, comp1.spatial.w} |

SCALING

| Description | Value |
| --- | --- |
| Method | Manual |
| Scale | 1.7715973018719574E-7 |

### Time-Dependent Solver 1 (t1)

GENERAL

| Description | Value |
| --- | --- |
| Defined by study step | Time Dependent |
| Output times | {110.00000000000001, 110.02750000000002, 110.05500000000002, 110.08250000000001, 110.11000000000001, 110.13750000000002, 110.16500000000002, 110.19250000000001, 110.22000000000001, 110.24750000000002, 110.27500000000002, 110.30250000000001, 110.33000000000001, 110.35750000000002, 110.38500000000002, 110.41250000000001, 110.44000000000001, 110.46750000000002, 110.49500000000002, 110.52250000000001, 110.55000000000001, 110.57750000000001, 110.60500000000002, 110.63250000000001, 110.66000000000001, 110.68750000000001, 110.71500000000002, 110.74250000000002, 110.77000000000001, 110.79750000000001, 110.82500000000002, 110.85250000000002, 110.88000000000001, 110.90750000000001, 110.93500000000002, 110.96250000000002, 110.99000000000001, 111.01750000000001, 111.04500000000002, 111.07250000000002, 111.10000000000001, 111.12750000000001, 111.15500000000002, 111.18250000000002, 111.21000000000001, 111.23750000000001, 111.26500000000001, 111.29250000000002, 111.32000000000002, 111.34750000000001, 111.37500000000001, 111.40250000000002, 111.43000000000002, 111.45750000000001, 111.48500000000001, 111.51250000000002, 111.54000000000002, 111.56750000000001, 111.59500000000001, 111.62250000000002, 111.65000000000002, 111.67750000000001, 111.70500000000001, 111.73250000000002, 111.76000000000002, 111.78750000000001, 111.81500000000001, 111.84250000000002, 111.87000000000002, 111.89750000000001, 111.92500000000001, 111.95250000000001, 111.98000000000002, 112.00750000000002, 112.03500000000001, 112.06250000000001, 112.09000000000002, 112.11750000000002, |

| Description | Value |
|---|---|
| | 112.14500000000001, 112.17250000000001, 112.20000000000002, 112.22750000000002, 112.25500000000001, 112.28250000000001, 112.31000000000002, 112.33750000000002, 112.36500000000001, 112.39250000000001, 112.42000000000002, 112.44750000000002, 112.47500000000001, 112.50250000000001, 112.53000000000002, 112.55750000000002, 112.58500000000001, 112.61250000000001, 112.64000000000001, 112.66750000000002, 112.69500000000002, 112.72250000000001, 112.75000000000001, 112.77750000000002, 112.80500000000002, 112.83250000000001, 112.86000000000001, 112.88750000000002, 112.91500000000002, 112.94250000000001, 112.97000000000001, 112.99750000000002, 113.02500000000002, 113.05250000000001, 113.08000000000001, 113.10750000000002, 113.13500000000002, 113.16250000000001, 113.19000000000001, 113.21750000000002, 113.24500000000002, 113.27250000000001, 113.30000000000001, 113.32750000000001, 113.35500000000002, 113.38250000000002, 113.41000000000001, 113.43750000000001, 113.46500000000002, 113.49250000000002, 113.52000000000001, 113.54750000000001, 113.57500000000002, 113.60250000000002, 113.63000000000001, 113.65750000000001, 113.68500000000002, 113.71250000000002, 113.74000000000001, 113.76750000000001, 113.79500000000002, 113.82250000000002, 113.85000000000001, 113.87750000000001, 113.90500000000002, 113.93250000000002, 113.96000000000001, 113.98750000000001, 114.01500000000001, 114.04250000000002, 114.07000000000002, 114.09750000000001, 114.12500000000001, 114.15250000000002, 114.18000000000002, 114.20750000000001, 114.23500000000001, 114.26250000000002, 114.29000000000002, 114.31750000000001, 114.34500000000001, 114.37250000000002, 114.40000000000002, 114.42750000000001, 114.45500000000001, 114.48250000000002, 114.51000000000002, 114.53750000000001, 114.56500000000001, 114.59250000000002, 114.62000000000002, 114.64750000000001, 114.67500000000001, 114.70250000000001, 114.73000000000002, 114.75750000000002, 114.78500000000001, 114.81250000000001, 114.84000000000002, 114.86750000000002, 114.89500000000001, 114.92250000000001, 114.95000000000002, 114.97750000000002, 115.00500000000001, 115.03250000000001, 115.06000000000002, 115.08750000000002, 115.11500000000001, 115.14250000000001, 115.17000000000002, 115.19750000000002, 115.22500000000001, 115.25250000000001, 115.28000000000002, 115.30750000000002, 115.33500000000001, 115.36250000000001, 115.39000000000001, 115.41750000000002, 115.44500000000002, 115.47250000000001, 115.50000000000001, 115.52750000000002, 115.55500000000002, 115.58250000000001, 115.61000000000001, 115.63750000000002, 115.66500000000002, 115.69250000000001, 115.72000000000001, 115.74750000000002, 115.77500000000002, 115.80250000000001, 115.83000000000001, 115.85750000000002, 115.88500000000002, 115.91250000000001, 115.94000000000001, 115.96750000000002, 115.99500000000002, 116.02250000000001, 116.05000000000001, 116.07750000000001, |

| Description | Value |
| --- | --- |
| | 116.10500000000002, 116.13250000000002, 116.16000000000001, 116.18750000000001, 116.21500000000002, 116.24250000000002, 116.27000000000001, 116.29750000000001, 116.32500000000002, 116.35250000000002, 116.38000000000001, 116.40750000000001, 116.43500000000002, 116.46250000000002, 116.49000000000001, 116.51750000000001, 116.54500000000002, 116.57250000000002, 116.60000000000001, 116.62750000000001, 116.65500000000002, 116.68250000000002, 116.71000000000001, 116.73750000000001, 116.76500000000001, 116.79250000000002, 116.82000000000002, 116.84750000000001, 116.87500000000001, 116.90250000000002, 116.93000000000002, 116.95750000000001, 116.98500000000001, 117.01250000000002, 117.04000000000002, 117.06750000000001, 117.09500000000001, 117.12250000000002, 117.15000000000002, 117.17750000000001, 117.20500000000001, 117.23250000000002, 117.26000000000002, 117.28750000000001, 117.31500000000001, 117.34250000000002, 117.37000000000002, 117.39750000000001, 117.42500000000001, 117.45250000000001, 117.48000000000002, 117.50750000000002, 117.53500000000001, 117.56250000000001, 117.59000000000002, 117.61750000000002, 117.64500000000001, 117.67250000000001, 117.70000000000002, 117.72750000000002, 117.75500000000001, 117.78250000000001, 117.81000000000002, 117.83750000000002, 117.86500000000001, 117.89250000000001, 117.92000000000002, 117.94750000000002, 117.97500000000002, 118.00250000000001, 118.03000000000002, 118.05750000000002, 118.08500000000001, 118.11250000000001, 118.14000000000001, 118.16750000000002, 118.19500000000002, 118.22250000000001, 118.25000000000001, 118.27750000000002, 118.30500000000002, 118.33250000000001, 118.36000000000001, 118.38750000000002, 118.41500000000002, 118.44250000000001, 118.47000000000001, 118.49750000000002, 118.52500000000002, 118.55250000000001, 118.58000000000001, 118.60750000000002, 118.63500000000002, 118.66250000000002, 118.69000000000001, 118.71750000000002, 118.74500000000002, 118.77250000000001, 118.80000000000001, 118.82750000000001, 118.85500000000002, 118.88250000000002, 118.91000000000001, 118.93750000000001, 118.96500000000002, 118.99250000000002, 119.02000000000001, 119.04750000000001, 119.07500000000002, 119.10250000000002, 119.13000000000001, 119.15750000000001, 119.18500000000002, 119.21250000000002, 119.24000000000001, 119.26750000000001, 119.29500000000002, 119.32250000000002, 119.35000000000002, 119.37750000000001, 119.40500000000002, 119.43250000000002, 119.46000000000001, 119.48750000000001, 119.51500000000001, 119.54250000000002, 119.57000000000002, 119.59750000000001, 119.62500000000001, 119.65250000000002, 119.68000000000002, 119.70750000000001, 119.73500000000001, 119.76250000000002, 119.79000000000002, 119.81750000000001, 119.84500000000001, 119.87250000000002, 119.90000000000002, 119.92750000000001, 119.95500000000001, 119.98250000000002, 120.01000000000002, 120.03750000000002, |

| Description | Value |
| --- | --- |
| | 120.06500000000001, 120.09250000000002, 120.12000000000002, 120.14750000000001, 120.17500000000001, 120.20250000000001, 120.23000000000002, 120.25750000000002, 120.28500000000001, 120.31250000000001, 120.34000000000002, 120.36750000000002, 120.39500000000001, 120.42250000000001, 120.45000000000002, 120.47750000000002, 120.50500000000001, 120.53250000000001, 120.56000000000002, 120.58750000000002, 120.61500000000001, 120.64250000000001, 120.67000000000002, 120.69750000000002, 120.72500000000002, 120.75250000000001, 120.78000000000002, 120.80750000000002, 120.83500000000001, 120.86250000000001, 120.89000000000001, 120.91750000000002, 120.94500000000002, 120.97250000000001, 121.00000000000001, 121.02750000000002, 121.05500000000002, 121.08250000000001, 121.11000000000001, 121.13750000000002, 121.16500000000002, 121.19250000000001, 121.22000000000001, 121.24750000000002, 121.27500000000002, 121.30250000000001, 121.33000000000001, 121.35750000000002, 121.38500000000002, 121.41250000000002, 121.44000000000001, 121.46750000000002, 121.49500000000002, 121.52250000000001, 121.55000000000001, 121.57750000000001, 121.60500000000002, 121.63250000000002, 121.66000000000001, 121.68750000000001, 121.71500000000002, 121.74250000000002, 121.77000000000001, 121.79750000000001, 121.82500000000002, 121.85250000000002, 121.88000000000001, 121.90750000000001, 121.93500000000002, 121.96250000000002, 121.99000000000001, 122.01750000000001, 122.04500000000002, 122.07250000000002, 122.10000000000002, 122.12750000000001, 122.15500000000002, 122.18250000000002, 122.21000000000001, 122.23750000000001, 122.26500000000001, 122.29250000000002, 122.32000000000002, 122.34750000000001, 122.37500000000001, 122.40250000000002, 122.43000000000002, 122.45750000000001, 122.48500000000001, 122.51250000000002, 122.54000000000002, 122.56750000000001, 122.59500000000001, 122.62250000000002, 122.65000000000002, 122.67750000000001, 122.70500000000001, 122.73250000000002, 122.76000000000002, 122.78750000000002, 122.81500000000001, 122.84250000000002, 122.87000000000002, 122.89750000000001, 122.92500000000001, 122.95250000000001, 122.98000000000002, 123.00750000000002, 123.03500000000001, 123.06250000000001, 123.09000000000002, 123.11750000000002, 123.14500000000001, 123.17250000000001, 123.20000000000002, 123.22750000000002, 123.25500000000002, 123.28250000000001, 123.31000000000002, 123.33750000000002, 123.36500000000001, 123.39250000000001, 123.42000000000002, 123.44750000000002, 123.47500000000002, 123.50250000000001, 123.53000000000002, 123.55750000000002, 123.58500000000002, 123.61250000000001, 123.64000000000001, 123.66750000000002, 123.69500000000002, 123.72250000000001, 123.75000000000001, 123.77750000000002, 123.80500000000002, 123.83250000000001, 123.86000000000001, 123.88750000000002, 123.91500000000002, 123.94250000000002, 123.97000000000001, 123.99750000000002, |

| Description | Value |
| --- | --- |
| | 124.02500000000002, 124.05250000000001, 124.08000000000001, 124.10750000000002, 124.13500000000002, 124.16250000000002, 124.19000000000001, 124.21750000000002, 124.24500000000002, 124.27250000000002, 124.30000000000001, 124.32750000000001, 124.35500000000002, 124.38250000000002, 124.41000000000001, 124.43750000000001, 124.46500000000002, 124.49250000000002, 124.52000000000001, 124.54750000000001, 124.57500000000002, 124.60250000000002, 124.63000000000002, 124.65750000000001, 124.68500000000002, 124.71250000000002, 124.74000000000001, 124.76750000000001, 124.79500000000002, 124.82250000000002, 124.85000000000002, 124.87750000000001, 124.90500000000002, 124.93250000000002, 124.96000000000002, 124.98750000000001, 125.01500000000001, 125.04250000000002, 125.07000000000002, 125.09750000000001, 125.12500000000001, 125.15250000000002, 125.18000000000002, 125.20750000000001, 125.23500000000001, 125.26250000000002, 125.29000000000002, 125.31750000000002, 125.34500000000001, 125.37250000000002, 125.40000000000002, 125.42750000000001, 125.45500000000001, 125.48250000000002, 125.51000000000002, 125.53750000000002, 125.56500000000001, 125.59250000000002, 125.62000000000002, 125.64750000000002, 125.67500000000001, 125.70250000000001, 125.73000000000002, 125.75750000000002, 125.78500000000001, 125.81250000000001, 125.84000000000002, 125.86750000000002, 125.89500000000001, 125.92250000000001, 125.95000000000002, 125.97750000000002, 126.00500000000002, 126.03250000000001, 126.06000000000002, 126.08750000000002, 126.11500000000001, 126.14250000000001, 126.17000000000002, 126.19750000000002, 126.22500000000002, 126.25250000000001, 126.28000000000002, 126.30750000000002, 126.33500000000001, 126.36250000000001, 126.39000000000001, 126.41750000000002, 126.44500000000002, 126.47250000000003, 126.50000000000001, 126.52750000000002, 126.55500000000002, 126.58250000000001, 126.61000000000001, 126.63750000000002, 126.66500000000002, 126.69250000000002, 126.72000000000001, 126.74750000000002, 126.77500000000002, 126.80250000000001, 126.83000000000001, 126.85750000000002, 126.88500000000002, 126.91250000000002, 126.94000000000001, 126.96750000000002, 126.99500000000002, 127.02250000000001, 127.05000000000001, 127.07750000000001, 127.10500000000002, 127.13250000000002, 127.16000000000003, 127.18750000000001, 127.21500000000002, 127.24250000000002, 127.27000000000001, 127.29750000000001, 127.32500000000002, 127.35250000000002, 127.38000000000002, 127.40750000000001, 127.43500000000002, 127.46250000000002, 127.49000000000001, 127.51750000000001, 127.54500000000002, 127.57250000000002, 127.60000000000002, 127.62750000000001, 127.65500000000002, 127.68250000000002, 127.71000000000001, 127.73750000000001, 127.76500000000001, 127.79250000000002, 127.82000000000002, 127.84750000000003, 127.87500000000001, 127.90250000000002, 127.93000000000002, 127.95750000000001, |

| Description | Value |
| --- | --- |
| | 127.98500000000001, 128.01250000000002, 128.04000000000002, 128.06750000000002, 128.09500000000003, 128.1225, 128.15, 128.1775, 128.205, 128.23250000000002, 128.26000000000002, 128.28750000000002, 128.31500000000003, 128.34250000000003, 128.37, 128.3975, 128.425, 128.45250000000001, 128.48000000000002, 128.50750000000002, 128.53500000000003, 128.56250000000003, 128.59000000000003, 128.6175, 128.645, 128.6725, 128.70000000000002, 128.72750000000002, 128.75500000000002, 128.78250000000003, 128.81, 128.8375, 128.865, 128.8925, 128.92000000000002, 128.94750000000002, 128.97500000000002, 129.00250000000003, 129.03000000000003, 129.0575, 129.085, 129.1125, 129.14000000000001, 129.16750000000002, 129.19500000000002, 129.22250000000003, 129.25000000000003, 129.27750000000003, 129.305, 129.3325, 129.36, 129.38750000000002, 129.41500000000002, 129.44250000000002, 129.47000000000003, 129.4975, 129.525, 129.5525, 129.58, 129.60750000000002, 129.63500000000002, 129.66250000000002, 129.69000000000003, 129.71750000000003, 129.745, 129.7725, 129.8, 129.82750000000001, 129.85500000000002, 129.88250000000002, 129.91000000000003, 129.93750000000003, 129.96500000000003, 129.9925, 130.02, 130.0475, 130.07500000000002, 130.10250000000002, 130.13000000000002, 130.15750000000003, 130.185, 130.2125, 130.24, 130.2675, 130.29500000000002, 130.32250000000002, 130.35000000000002, 130.37750000000003, 130.40500000000003, 130.4325, 130.46, 130.4875, 130.51500000000001, 130.54250000000002, 130.57000000000002, 130.59750000000003, 130.62500000000003, 130.65250000000003, 130.68, 130.7075, 130.735, 130.76250000000002, 130.79000000000002, 130.81750000000002, 130.84500000000003, 130.8725, 130.9, 130.9275, 130.955, 130.98250000000002, 131.01000000000002, 131.03750000000002, 131.06500000000003, 131.09250000000003, 131.12, 131.1475, 131.175, 131.20250000000001, 131.23000000000002, 131.25750000000002, 131.28500000000003, 131.31250000000003, 131.34000000000003, 131.3675, 131.395, 131.4225, 131.45000000000002, 131.47750000000002, 131.50500000000002, 131.53250000000003, 131.56, 131.5875, 131.615, 131.6425, 131.67000000000002, 131.69750000000002, 131.72500000000002, 131.75250000000003, 131.78000000000003, 131.8075, 131.835, 131.8625, 131.89000000000001, 131.91750000000002, 131.94500000000002, 131.97250000000003, 132.00000000000003, 132.02750000000003, 132.055, 132.0825, 132.11, 132.13750000000002, 132.16500000000002, 132.19250000000002, 132.22000000000003, 132.2475, 132.275, 132.3025, 132.33, 132.35750000000002, 132.38500000000002, 132.41250000000002, 132.44000000000003, 132.46750000000003, 132.495, 132.5225, 132.55, 132.57750000000001, 132.60500000000002, 132.63250000000002, 132.66000000000003, 132.68750000000003, 132.71500000000003, 132.7425, 132.77, 132.7975, 132.82500000000002, 132.85250000000002, 132.88000000000002, 132.90750000000003, 132.935, 132.9625, 132.99, 133.0175, 133.04500000000002, 133.07250000000002, 133.10000000000002, 133.12750000000003, 133.15500000000003, 133.1825, 133.21, 133.2375, |

| Description | Value |
| --- | --- |
| | 133.26500000000001, 133.29250000000002, 133.32000000000002, 133.34750000000003, 133.37500000000003, 133.40250000000003, 133.43, 133.4575, 133.485, 133.51250000000002, 133.54000000000002, 133.56750000000002, 133.59500000000003, 133.6225, 133.65, 133.6775, 133.705, 133.73250000000002, 133.76000000000002, 133.78750000000002, 133.81500000000003, 133.84250000000003, 133.87, 133.8975, 133.925, 133.95250000000001, 133.98000000000002, 134.00750000000002, 134.03500000000003, 134.06250000000003, 134.09000000000003, 134.1175, 134.145, 134.1725, 134.20000000000002, 134.22750000000002, 134.25500000000002, 134.28250000000003, 134.31, 134.3375, 134.365, 134.3925, 134.42000000000002, 134.44750000000002, 134.47500000000002, 134.50250000000003, 134.53000000000003, 134.5575, 134.585, 134.6125, 134.64000000000001, 134.66750000000002, 134.69500000000002, 134.72250000000003, 134.75000000000003, 134.77750000000003, 134.805, 134.8325, 134.86, 134.88750000000002, 134.91500000000002, 134.94250000000002, 134.97000000000003, 134.9975, 135.025, 135.0525, 135.08, 135.10750000000002, 135.13500000000002, 135.16250000000002, 135.19000000000003, 135.21750000000003, 135.245, 135.2725, 135.3, 135.32750000000001, 135.35500000000002, 135.38250000000002, 135.41000000000003, 135.43750000000003, 135.46500000000003, 135.4925, 135.52, 135.5475, 135.57500000000002, 135.60250000000002, 135.63000000000002, 135.65750000000003, 135.685, 135.7125, 135.74, 135.7675, 135.79500000000002, 135.82250000000002, 135.85000000000002, 135.87750000000003, 135.90500000000003, 135.9325, 135.96, 135.9875, 136.01500000000001, 136.04250000000002, 136.07000000000002, 136.09750000000003, 136.12500000000003, 136.15250000000003, 136.18, 136.2075, 136.235, 136.26250000000002, 136.29000000000002, 136.31750000000002, 136.34500000000003, 136.3725, 136.4, 136.4275, 136.455, 136.48250000000002, 136.51000000000002, 136.53750000000002, 136.56500000000003, 136.59250000000003, 136.62, 136.6475, 136.675, 136.70250000000001, 136.73000000000002, 136.75750000000002, 136.78500000000003, 136.81250000000003, 136.84000000000003, 136.8675, 136.895, 136.9225, 136.95000000000002, 136.97750000000002, 137.00500000000002, 137.03250000000003, 137.06, 137.0875, 137.115, 137.1425, 137.17000000000002, 137.19750000000002, 137.22500000000002, 137.25250000000003, 137.28000000000003, 137.3075, 137.335, 137.3625, 137.39000000000001, 137.41750000000002, 137.44500000000002, 137.47250000000003, 137.50000000000003, 137.52750000000003, 137.555, 137.5825, 137.61, 137.63750000000002, 137.66500000000002, 137.69250000000002, 137.72000000000003, 137.7475, 137.775, 137.8025, 137.83, 137.85750000000002, 137.88500000000002, 137.91250000000002, 137.94000000000003, 137.96750000000003, 137.995, 138.0225, 138.05, 138.07750000000001, 138.10500000000002, 138.13250000000002, 138.16000000000003, 138.18750000000003, 138.21500000000003, 138.2425, 138.27, 138.2975, 138.32500000000002, 138.35250000000002, 138.38000000000002, 138.40750000000003, 138.435, 138.4625, 138.49, 138.5175, |

| Description | Value |
| --- | --- |
| | 138.54500000000002, 138.57250000000002, 138.60000000000002, 138.62750000000003, 138.65500000000003, 138.6825, 138.71, 138.7375, 138.76500000000001, 138.79250000000002, 138.82000000000002, 138.84750000000003, 138.87500000000003, 138.90250000000003, 138.93, 138.9575, 138.985, 139.01250000000002, 139.04000000000002, 139.06750000000002, 139.09500000000003, 139.1225, 139.15, 139.1775, 139.205, 139.23250000000002, 139.26000000000002, 139.28750000000002, 139.31500000000003, 139.34250000000003, 139.37, 139.3975, 139.425, 139.45250000000001, 139.48000000000002, 139.50750000000002, 139.53500000000003, 139.56250000000003, 139.59000000000003, 139.6175, 139.645, 139.6725, 139.70000000000002, 139.72750000000002, 139.75500000000002, 139.78250000000003, 139.81, 139.83750000000003, 139.865, 139.8925, 139.92000000000002, 139.94750000000002, 139.97500000000002, 140.00250000000003, 140.03000000000003, 140.0575, 140.085, 140.1125, 140.14000000000001, 140.16750000000002, 140.19500000000002, 140.22250000000003, 140.25000000000003, 140.27750000000003, 140.305, 140.3325, 140.36, 140.38750000000002, 140.41500000000002, 140.44250000000002, 140.47000000000003, 140.4975, 140.52500000000003, 140.5525, 140.58, 140.60750000000002, 140.63500000000002, 140.66250000000002, 140.69000000000003, 140.71750000000003, 140.745, 140.7725, 140.8, 140.82750000000001, 140.85500000000002, 140.88250000000002, 140.91000000000003, 140.93750000000003, 140.96500000000003, 140.9925, 141.02, 141.0475, 141.07500000000002, 141.10250000000002, 141.13000000000002, 141.15750000000003, 141.18500000000003, 141.21250000000003, 141.24, 141.2675, 141.29500000000002, 141.32250000000002, 141.35000000000002, 141.37750000000003, 141.40500000000003, 141.4325, 141.46, 141.4875, 141.51500000000001, 141.54250000000002, 141.57000000000002, 141.59750000000003, 141.62500000000003, 141.65250000000003, 141.68, 141.7075, 141.735, 141.76250000000002, 141.79000000000002, 141.81750000000002, 141.84500000000003, 141.87250000000003, 141.90000000000003, 141.9275, 141.955, 141.98250000000002, 142.01000000000002, 142.03750000000002, 142.06500000000003, 142.09250000000003, 142.12, 142.1475, 142.175, 142.20250000000001, 142.23000000000002, 142.25750000000002, 142.28500000000003, 142.31250000000003, 142.34000000000003, 142.3675, 142.395, 142.4225, 142.45000000000002, 142.47750000000002, 142.50500000000002, 142.53250000000003, 142.56, 142.58750000000003, 142.615, 142.6425, 142.67000000000002, 142.69750000000002, 142.72500000000002, 142.75250000000003, 142.78000000000003, 142.8075, 142.835, 142.8625, 142.89000000000001, 142.91750000000002, 142.94500000000002, 142.97250000000003, 143.00000000000003, 143.02750000000003, 143.055, 143.0825, 143.11, 143.13750000000002, 143.16500000000002, 143.19250000000002, 143.22000000000003, 143.2475, 143.27500000000003, 143.3025, 143.33, 143.35750000000002, 143.38500000000002, 143.41250000000002, 143.44000000000003, 143.46750000000003, 143.495, 143.5225, 143.55, 143.57750000000001, 143.60500000000002, 143.63250000000002, |

| Description | Value |
|---|---|
| | 143.66000000000003, 143.68750000000003, 143.71500000000003, 143.7425, 143.77, 143.7975, 143.82500000000002, 143.85250000000002, 143.88000000000002, 143.90750000000003, 143.935, 143.96250000000003, 143.99, 144.0175, 144.04500000000002, 144.07250000000002, 144.10000000000002, 144.12750000000003, 144.15500000000003, 144.1825, 144.21000000000004, 144.2375, 144.26500000000001, 144.29250000000002, 144.32000000000002, 144.34750000000003, 144.37500000000003, 144.40250000000003, 144.43, 144.4575, 144.485, 144.51250000000002, 144.54000000000002, 144.56750000000002, 144.59500000000003, 144.6225, 144.65000000000003, 144.6775, 144.705, 144.73250000000002, 144.76000000000002, 144.78750000000002, 144.81500000000003, 144.84250000000003, 144.87, 144.89750000000004, 144.925, 144.95250000000001, 144.98000000000002, 145.00750000000002, 145.03500000000003, 145.06250000000003, 145.09000000000003, 145.1175, 145.145, 145.1725, 145.20000000000002, 145.22750000000002, 145.25500000000002, 145.28250000000003, 145.31, 145.33750000000003, 145.365, 145.3925, 145.42000000000002, 145.44750000000002, 145.47500000000002, 145.50250000000003, 145.53000000000003, 145.5575, 145.58500000000004, 145.6125, 145.64000000000001, 145.66750000000002, 145.69500000000002, 145.72250000000003, 145.75000000000003, 145.77750000000003, 145.805, 145.8325, 145.86, 145.88750000000002, 145.91500000000002, 145.94250000000002, 145.97000000000003, 145.9975, 146.02500000000003, 146.0525, 146.08, 146.10750000000002, 146.13500000000002, 146.16250000000002, 146.19000000000003, 146.21750000000003, 146.245, 146.27250000000004, 146.3, 146.32750000000001, 146.35500000000002, 146.38250000000002, 146.41000000000003, 146.43750000000003, 146.46500000000003, 146.4925, 146.52, 146.5475, 146.57500000000002, 146.60250000000002, 146.63000000000002, 146.65750000000003, 146.685, 146.71250000000003, 146.74, 146.7675, 146.79500000000002, 146.82250000000002, 146.85000000000002, 146.87750000000003, 146.90500000000003, 146.9325, 146.96000000000004, 146.9875, 147.01500000000001, 147.04250000000002, 147.07000000000002, 147.09750000000003, 147.12500000000003, 147.15250000000003, 147.18, 147.2075, 147.235, 147.26250000000002, 147.29000000000002, 147.31750000000002, 147.34500000000003, 147.3725, 147.40000000000003, 147.4275, 147.455, 147.48250000000002, 147.51000000000002, 147.53750000000002, 147.56500000000003, 147.59250000000003, 147.62, 147.64750000000004, 147.675, 147.70250000000001, 147.73000000000002, 147.75750000000002, 147.78500000000003, 147.81250000000003, 147.84000000000003, 147.8675, 147.895, 147.9225, 147.95000000000002, 147.97750000000002, 148.00500000000002, 148.03250000000003, 148.06, 148.08750000000003, 148.115, 148.1425, 148.17000000000002, 148.19750000000002, 148.22500000000002, 148.25250000000003, 148.28000000000003, 148.3075, 148.33500000000004, 148.3625, 148.39000000000001, 148.41750000000002, 148.44500000000002, 148.47250000000003, |

| Description | Value |
|---|---|
| | 148.50000000000003, 148.52750000000003, 148.555, 148.5825, 148.61, 148.63750000000002, 148.66500000000002, 148.69250000000002, 148.72000000000003, 148.7475, 148.77500000000003, 148.8025, 148.83, 148.85750000000002, 148.88500000000002, 148.91250000000002, 148.94000000000003, 148.96750000000003, 148.995, 149.02250000000004, 149.05, 149.07750000000001, 149.10500000000002, 149.13250000000002, 149.16000000000003, 149.18750000000003, 149.21500000000003, 149.2425, 149.27, 149.2975, 149.32500000000002, 149.35250000000002, 149.38000000000002, 149.40750000000003, 149.435, 149.46250000000003, 149.49, 149.5175, 149.54500000000002, 149.57250000000002, 149.60000000000002, 149.62750000000003, 149.65500000000003, 149.6825, 149.71000000000004, 149.7375, 149.76500000000001, 149.79250000000002, 149.82000000000002, 149.84750000000003, 149.87500000000003, 149.90250000000003, 149.93, 149.9575, 149.985, 150.01250000000002, 150.04000000000002, 150.06750000000002, 150.09500000000003, 150.1225, 150.15000000000003, 150.1775, 150.205, 150.23250000000002, 150.26000000000002, 150.28750000000002, 150.31500000000003, 150.34250000000003, 150.37, 150.39750000000004, 150.425, 150.45250000000001, 150.48000000000002, 150.50750000000002, 150.53500000000003, 150.56250000000003, 150.59000000000003, 150.6175, 150.645, 150.6725, 150.70000000000002, 150.72750000000002, 150.75500000000002, 150.78250000000003, 150.81, 150.83750000000003, 150.865, 150.8925, 150.92000000000002, 150.94750000000002, 150.97500000000002, 151.00250000000003, 151.03000000000003, 151.0575, 151.08500000000004, 151.1125, 151.14000000000001, 151.16750000000002, 151.19500000000002, 151.22250000000003, 151.25000000000003, 151.27750000000003, 151.305, 151.3325, 151.36, 151.38750000000002, 151.41500000000002, 151.44250000000002, 151.47000000000003, 151.4975, 151.52500000000003, 151.5525, 151.58, 151.60750000000002, 151.63500000000002, 151.66250000000002, 151.69000000000003, 151.71750000000003, 151.745, 151.77250000000004, 151.8, 151.82750000000001, 151.85500000000002, 151.88250000000002, 151.91000000000003, 151.93750000000003, 151.96500000000003, 151.9925, 152.02, 152.0475, 152.07500000000002, 152.10250000000002, 152.13000000000002, 152.15750000000003, 152.185, 152.21250000000003, 152.24, 152.2675, 152.29500000000002, 152.32250000000002, 152.35000000000002, 152.37750000000003, 152.40500000000003, 152.4325, 152.46000000000004, 152.4875, 152.51500000000001, 152.54250000000002, 152.57000000000002, 152.59750000000003, 152.62500000000003, 152.65250000000003, 152.68, 152.7075, 152.735, 152.76250000000002, 152.79000000000002, 152.81750000000002, 152.84500000000003, 152.8725, 152.90000000000003, 152.9275, 152.955, 152.98250000000002, 153.01000000000002, 153.03750000000002, 153.06500000000003, 153.09250000000003, 153.12, 153.14750000000004, 153.175, 153.20250000000001, 153.23000000000002, 153.25750000000002, 153.28500000000003, 153.31250000000003, 153.34000000000003, |

| Description | Value |
| --- | --- |
| | 153.3675, 153.395, 153.4225, 153.45000000000002, 153.47750000000002, 153.50500000000002, 153.53250000000003, 153.56, 153.58750000000003, 153.615, 153.6425, 153.67000000000002, 153.69750000000002, 153.72500000000002, 153.75250000000003, 153.78000000000003, 153.8075, 153.83500000000004, 153.8625, 153.89000000000001, 153.91750000000002, 153.94500000000002, 153.97250000000003, 154.00000000000003, 154.02750000000003, 154.055, 154.0825, 154.11, 154.13750000000002, 154.16500000000002, 154.19250000000002, 154.22000000000003, 154.2475, 154.27500000000003, 154.3025, 154.33, 154.35750000000002, 154.38500000000002, 154.41250000000002, 154.44000000000003, 154.46750000000003, 154.495, 154.52250000000004, 154.55, 154.57750000000001, 154.60500000000002, 154.63250000000002, 154.66000000000003, 154.68750000000003, 154.71500000000003, 154.7425, 154.77, 154.7975, 154.82500000000002, 154.85250000000002, 154.88000000000002, 154.90750000000003, 154.93500000000003, 154.96250000000003, 154.99, 155.0175, 155.04500000000002, 155.07250000000002, 155.10000000000002, 155.12750000000003, 155.15500000000003, 155.1825, 155.21000000000004, 155.2375, 155.26500000000001, 155.29250000000002, 155.32000000000002, 155.34750000000003, 155.37500000000003, 155.40250000000003, 155.43, 155.4575, 155.485, 155.51250000000002, 155.54000000000002, 155.56750000000002, 155.59500000000003, 155.62250000000003, 155.65000000000003, 155.6775, 155.705, 155.73250000000002, 155.76000000000002, 155.78750000000002, 155.81500000000003, 155.84250000000003, 155.87, 155.89750000000004, 155.925, 155.95250000000001, 155.98000000000002, 156.00750000000002, 156.03500000000003, 156.06250000000003, 156.09000000000003, 156.1175, 156.145, 156.1725, 156.20000000000002, 156.22750000000002, 156.25500000000002, 156.28250000000003, 156.31000000000003, 156.33750000000003, 156.365, 156.3925, 156.42000000000002, 156.44750000000002, 156.47500000000002, 156.50250000000003, 156.53000000000003, 156.5575, 156.58500000000004, 156.6125, 156.64000000000001, 156.66750000000002, 156.69500000000002, 156.72250000000003, 156.75000000000003, 156.77750000000003, 156.805, 156.8325, 156.86, 156.88750000000002, 156.91500000000002, 156.94250000000002, 156.97000000000003, 156.99750000000003, 157.02500000000003, 157.0525, 157.08, 157.10750000000002, 157.13500000000002, 157.16250000000002, 157.19000000000003, 157.21750000000003, 157.245, 157.27250000000004, 157.3, 157.32750000000001, 157.35500000000002, 157.38250000000002, 157.41000000000003, 157.43750000000003, 157.46500000000003, 157.4925, 157.52, 157.5475, 157.57500000000002, 157.60250000000002, 157.63000000000002, 157.65750000000003, 157.68500000000003, 157.71250000000003, 157.74, 157.7675, 157.79500000000002, 157.82250000000002, 157.85000000000002, 157.87750000000003, 157.90500000000003, 157.9325, 157.96000000000004, 157.9875, 158.01500000000001, 158.04250000000002, 158.07000000000002, 158.09750000000003, |

| Description | Value |
| --- | --- |
| | 158.12500000000003, 158.15250000000003, 158.18, 158.2075, 158.235, 158.26250000000002, 158.29000000000002, 158.31750000000002, 158.34500000000003, 158.37250000000003, 158.40000000000003, 158.4275, 158.455, 158.48250000000002, 158.51000000000002, 158.53750000000002, 158.56500000000003, 158.59250000000003, 158.62, 158.64750000000004, 158.675, 158.70250000000001, 158.73000000000002, 158.75750000000002, 158.78500000000003, 158.81250000000003, 158.84000000000003, 158.8675, 158.895, 158.9225, 158.95000000000002, 158.97750000000002, 159.00500000000002, 159.03250000000003, 159.06000000000003, 159.08750000000003, 159.115, 159.1425, 159.17000000000002, 159.19750000000002, 159.22500000000002, 159.25250000000003, 159.28000000000003, 159.3075, 159.33500000000004, 159.3625, 159.39000000000001, 159.41750000000002, 159.44500000000002, 159.47250000000003, 159.50000000000003, 159.52750000000003, 159.555, 159.5825, 159.61, 159.63750000000002, 159.66500000000002, 159.69250000000002, 159.72000000000003, 159.74750000000003, 159.77500000000003, 159.8025, 159.83, 159.85750000000002, 159.88500000000002, 159.91250000000002, 159.94000000000003, 159.96750000000003, 159.995, 160.02250000000004, 160.05, 160.07750000000001, 160.10500000000002, 160.13250000000002, 160.16000000000003, 160.18750000000003, 160.21500000000003, 160.2425, 160.27, 160.2975, 160.32500000000002, 160.35250000000002, 160.38000000000002, 160.40750000000003, 160.43500000000003, 160.46250000000003, 160.49, 160.5175, 160.54500000000002, 160.57250000000002, 160.60000000000002, 160.62750000000003, 160.65500000000003, 160.6825, 160.71000000000004, 160.7375, 160.76500000000001, 160.79250000000002, 160.82000000000002, 160.84750000000003, 160.87500000000003, 160.90250000000003, 160.93, 160.95750000000004, 160.985, 161.01250000000002, 161.04000000000002, 161.06750000000002, 161.09500000000003, 161.12250000000003, 161.15000000000003, 161.1775, 161.205, 161.23250000000002, 161.26000000000002, 161.28750000000002, 161.31500000000003, 161.34250000000003, 161.37, 161.39750000000004, 161.425, 161.45250000000001, 161.48000000000002, 161.50750000000002, 161.53500000000003, 161.56250000000003, 161.59000000000003, 161.6175, 161.64500000000004, 161.6725, 161.70000000000002, 161.72750000000002, 161.75500000000002, 161.78250000000003, 161.81000000000003, 161.83750000000003, 161.865, 161.8925, 161.92000000000002, 161.94750000000002, 161.97500000000002, 162.00250000000003, 162.03000000000003, 162.0575, 162.08500000000004, 162.1125, 162.14000000000001, 162.16750000000002, 162.19500000000002, 162.22250000000003, 162.25000000000003, 162.27750000000003, 162.305, 162.33250000000004, 162.36, 162.38750000000002, 162.41500000000002, 162.44250000000002, 162.47000000000003, 162.49750000000003, 162.52500000000003, 162.5525, 162.58, 162.60750000000002, 162.63500000000002, 162.66250000000002, 162.69000000000003, |

| Description | Value |
|---|---|
| | 162.71750000000003, 162.745, 162.77250000000004, 162.8, 162.82750000000001, 162.85500000000002, 162.88250000000002, 162.91000000000003, 162.93750000000003, 162.96500000000003, 162.9925, 163.02000000000004, 163.0475, 163.07500000000002, 163.10250000000002, 163.13000000000002, 163.15750000000003, 163.18500000000003, 163.21250000000003, 163.24, 163.2675, 163.29500000000002, 163.32250000000002, 163.35000000000002, 163.37750000000003, 163.40500000000003, 163.4325, 163.46000000000004, 163.4875, 163.51500000000001, 163.54250000000002, 163.57000000000002, 163.59750000000003, 163.62500000000003, 163.65250000000003, 163.68, 163.70750000000004, 163.735, 163.76250000000002, 163.79000000000002, 163.81750000000002, 163.84500000000003, 163.87250000000003, 163.90000000000003, 163.9275, 163.955, 163.98250000000002, 164.01000000000002, 164.03750000000002, 164.06500000000003, 164.09250000000003, 164.12, 164.14750000000004, 164.175, 164.20250000000001, 164.23000000000002, 164.25750000000002, 164.28500000000003, 164.31250000000003, 164.34000000000003, 164.3675, 164.39500000000004, 164.4225, 164.45000000000002, 164.47750000000002, 164.50500000000002, 164.53250000000003, 164.56000000000003, 164.58750000000003, 164.615, 164.6425, 164.67000000000002, 164.69750000000002, 164.72500000000002, 164.75250000000003, 164.78000000000003, 164.8075, 164.83500000000004, 164.8625, 164.89000000000001, 164.91750000000002, 164.94500000000002, 164.97250000000003, 165.00000000000003} |
| Relative tolerance | 0.005 |

TIME STEPPING

| Description | Value |
|---|---|
| Maximum BDF order | 2 |
| Nonlinear controller | On |

LOG

| | | | | | | | | | | |
|---|---|---|---|---|---|---|---|---|---|---|
| - | 151.22 | - out | | | | | | | | |
| - | 151.25 | - out | | | | | | | | |
| - | 151.28 | - out | | | | | | | | |
| - | 151.31 | - out | | | | | | | | |
| - | 151.33 | - out | | | | | | | | |
| 438 | 151.35 | 0.27192 | 336 | 169 | 336 | 2 | 15 | 0 | 8.2e-13 | 4.1e-15 |
| - | 151.36 | - out | | | | | | | | |
| - | 151.39 | - out | | | | | | | | |
| - | 151.42 | - out | | | | | | | | |
| - | 151.44 | - out | | | | | | | | |
| - | 151.47 | - out | | | | | | | | |
| - | 151.5 | - out | | | | | | | | |
| - | 151.53 | - out | | | | | | | | |
| - | 151.55 | - out | | | | | | | | |
| - | 151.58 | - out | | | | | | | | |
| - | 151.61 | - out | | | | | | | | |
| 439 | 151.62 | 0.27192 | 338 | 170 | 338 | 2 | 15 | 0 | 1.4e-14 | 1.3e-14 |
| - | 151.64 | - out | | | | | | | | |
| - | 151.66 | - out | | | | | | | | |
| - | 151.69 | - out | | | | | | | | |
| - | 151.72 | - out | | | | | | | | |
| - | 151.75 | - out | | | | | | | | |
| - | 151.77 | - out | | | | | | | | |
| - | 151.8 | - out | | | | | | | | |
| - | 151.83 | - out | | | | | | | | |
| - | 151.86 | - out | | | | | | | | |
| 440 | 151.86 | 0.23188 | 342 | 172 | 342 | 2 | 16 | 0 | 1.7e-12 | 4.9e-15 |
| - | 151.88 | - out | | | | | | | | |
| - | 151.91 | - out | | | | | | | | |
| - | 151.94 | - out | | | | | | | | |
| - | 151.97 | - out | | | | | | | | |
| - | 151.99 | - out | | | | | | | | |
| - | 152.02 | - out | | | | | | | | |
| - | 152.05 | - out | | | | | | | | |
| - | 152.08 | - out | | | | | | | | |
| 441 | 152.09 | 0.23188 | 344 | 173 | 344 | 2 | 16 | 0 | 1.7e-13 | 3.9e-15 |
| - | 152.1 | - out | | | | | | | | |
| - | 152.13 | - out | | | | | | | | |
| - | 152.16 | - out | | | | | | | | |
| - | 152.19 | - out | | | | | | | | |
| 442 | 152.19 | 0.10221 | 348 | 175 | 348 | 2 | 17 | 0 | 1.4e-12 | 6e-15 |
| - | 152.21 | - out | | | | | | | | |
| - | 152.24 | - out | | | | | | | | |
| - | 152.27 | - out | | | | | | | | |
| 443 | 152.29 | 0.10221 | 350 | 176 | 350 | 2 | 17 | 0 | 1.2e-13 | 5.1e-15 |
| - | 152.3 | - out | | | | | | | | |
| - | 152.32 | - out | | | | | | | | |
| - | 152.35 | - out | | | | | | | | |
| - | 152.38 | - out | | | | | | | | |
| 444 | 152.38 | 0.087549 | 352 | 177 | 352 | 2 | 17 | 0 | 3e-13 | 4.3e-15 |
| - | 152.41 | - out | | | | | | | | |
| - | 152.43 | - out | | | | | | | | |
| 445 | 152.45 | 0.071948 | 354 | 178 | 354 | 2 | 17 | 0 | 9.1e-14 | 3.6e-15 |
| - | 152.46 | - out | | | | | | | | |
| - | 152.49 | - out | | | | | | | | |
| - | 152.52 | - out | | | | | | | | |

| | | | | | | | | | | |
|---|---|---|---|---|---|---|---|---|---|---|
| 446 | 152.52 | 0.064753 | 356 | 179 | 356 | 2 | 17 | 0 | 1.9e-14 | 3.8e-15 |
| - | 152.54 | - out | | | | | | | | |
| - | 152.57 | - out | | | | | | | | |
| 447 | 152.58 | 0.064753 | 358 | 180 | 358 | 2 | 17 | 0 | 1.5e-14 | 4.1e-15 |
| - | 152.6 | - out | | | | | | | | |
| - | 152.63 | - out | | | | | | | | |
| 448 | 152.65 | 0.064753 | 360 | 181 | 360 | 2 | 17 | 0 | 1e-13 | 4.5e-15 |
| - | 152.65 | - out | | | | | | | | |
| - | 152.68 | - out | | | | | | | | |
| - | 152.71 | - out | | | | | | | | |
| 449 | 152.71 | 0.064753 | 362 | 182 | 362 | 2 | 17 | 0 | 3.7e-14 | 5.4e-15 |
| - | 152.74 | - out | | | | | | | | |
| - | 152.76 | - out | | | | | | | | |
| 450 | 152.78 | 0.064753 | 364 | 183 | 364 | 2 | 17 | 0 | 4.8e-14 | 5.5e-15 |
| - | 152.79 | - out | | | | | | | | |
| - | 152.82 | - out | | | | | | | | |
| 451 | 152.84 | 0.064753 | 366 | 184 | 366 | 2 | 17 | 0 | 1.7e-14 | 5e-15 |
| - | 152.85 | - out | | | | | | | | |
| - | 152.87 | - out | | | | | | | | |
| - | 152.9 | - out | | | | | | | | |
| 452 | 152.91 | 0.064753 | 368 | 185 | 368 | 2 | 17 | 0 | 1.1e-14 | 5.2e-15 |
| - | 152.93 | - out | | | | | | | | |
| - | 152.96 | - out | | | | | | | | |
| 453 | 152.97 | 0.064753 | 370 | 186 | 370 | 2 | 17 | 0 | 5.8e-14 | 5e-15 |
| - | 152.98 | - out | | | | | | | | |
| - | 153.01 | - out | | | | | | | | |
| 454 | 153.03 | 0.064753 | 372 | 187 | 372 | 2 | 17 | 0 | 1.1e-13 | 4.9e-15 |
| - | 153.04 | - out | | | | | | | | |
| - | 153.07 | - out | | | | | | | | |
| - | 153.09 | - out | | | | | | | | |
| 455 | 153.1 | 0.064753 | 374 | 188 | 374 | 2 | 17 | 0 | 2.4e-13 | 6e-15 |
| - | 153.12 | - out | | | | | | | | |
| - | 153.15 | - out | | | | | | | | |
| 456 | 153.16 | 0.064753 | 376 | 189 | 376 | 2 | 17 | 0 | 1.3e-13 | 3.8e-15 |
| - | 153.18 | - out | | | | | | | | |
| - | 153.2 | - out | | | | | | | | |
| 457 | 153.23 | 0.064753 | 378 | 190 | 378 | 2 | 17 | 0 | 2.9e-13 | 4.3e-15 |
| - | 153.23 | - out | | | | | | | | |
| - | 153.26 | - out | | | | | | | | |
| - | 153.29 | - out | | | | | | | | |
| 458 | 153.29 | 0.064753 | 380 | 191 | 380 | 2 | 17 | 0 | 2.5e-13 | 4.1e-15 |
| - | 153.31 | - out | | | | | | | | |
| - | 153.34 | - out | | | | | | | | |
| 459 | 153.36 | 0.064753 | 382 | 192 | 382 | 2 | 17 | 0 | 1.1e-12 | 5.3e-15 |
| - | 153.37 | - out | | | | | | | | |
| - | 153.4 | - out | | | | | | | | |
| - | 153.42 | - out | | | | | | | | |
| 460 | 153.42 | 0.064753 | 384 | 193 | 384 | 2 | 17 | 0 | 2.8e-13 | 2.8e-15 |
| - | 153.45 | - out | | | | | | | | |
| - | 153.48 | - out | | | | | | | | |
| 461 | 153.49 | 0.064753 | 386 | 194 | 386 | 2 | 17 | 0 | 7.7e-13 | 5.6e-15 |
| - | 153.51 | - out | | | | | | | | |
| - | 153.53 | - out | | | | | | | | |
| 462 | 153.55 | 0.064753 | 388 | 195 | 388 | 2 | 17 | 0 | 1e-12 | 4e-15 |
| - | 153.56 | - out | | | | | | | | |
| - | 153.59 | - out | | | | | | | | |

| | | | | | | | | | |
|---|---|---|---|---|---|---|---|---|---|
| - | 153.62 | - out | | | | | | | |
| - | 153.64 | - out | | | | | | | |
| - | 153.67 | - out | | | | | | | |
| 463 | 153.68 | 0.12951 | 390 | 196 | 390 | 2 | 17 | 0 | 1.6e-12 | 3.5e-15 |
| - | 153.7 | - out | | | | | | | |
| - | 153.73 | - out | | | | | | | |
| - | 153.75 | - out | | | | | | | |
| - | 153.78 | - out | | | | | | | |
| - | 153.81 | - out | | | | | | | |
| 464 | 153.81 | 0.12951 | 392 | 197 | 392 | 2 | 17 | 0 | 1e-12 | 4.1e-15 |
| - | 153.84 | - out | | | | | | | |
| - | 153.86 | - out | | | | | | | |
| - | 153.89 | - out | | | | | | | |
| - | 153.92 | - out | | | | | | | |
| 465 | 153.94 | 0.12951 | 394 | 198 | 394 | 2 | 17 | 0 | 1.8e-12 | 3.3e-15 |
| - | 153.95 | - out | | | | | | | |
| - | 153.97 | - out | | | | | | | |
| - | 154 | - out | | | | | | | |
| - | 154.03 | - out | | | | | | | |
| - | 154.06 | - out | | | | | | | |
| 466 | 154.07 | 0.12951 | 396 | 199 | 396 | 2 | 17 | 0 | 7.2e-13 | 5.5e-15 |
| - | 154.08 | - out | | | | | | | |
| - | 154.11 | - out | | | | | | | |
| - | 154.14 | - out | | | | | | | |
| - | 154.17 | - out | | | | | | | |
| - | 154.19 | - out | | | | | | | |
| - | 154.22 | - out | | | | | | | |
| - | 154.25 | - out | | | | | | | |
| - | 154.28 | - out | | | | | | | |
| - | 154.3 | - out | | | | | | | |
| 467 | 154.33 | 0.25901 | 398 | 200 | 398 | 2 | 17 | 0 | 9.1e-14 | 5e-15 |
| - | 154.33 | - out | | | | | | | |
| - | 154.36 | - out | | | | | | | |
| - | 154.39 | - out | | | | | | | |
| - | 154.41 | - out | | | | | | | |
| - | 154.44 | - out | | | | | | | |
| - | 154.47 | - out | | | | | | | |
| - | 154.5 | - out | | | | | | | |
| - | 154.52 | - out | | | | | | | |
| - | 154.55 | - out | | | | | | | |
| - | 154.58 | - out | | | | | | | |
| 468 | 154.59 | 0.25901 | 400 | 201 | 400 | 2 | 17 | 0 | 3.4e-13 | 3.4e-15 |
| - | 154.61 | - out | | | | | | | |
| - | 154.63 | - out | | | | | | | |
| - | 154.66 | - out | | | | | | | |
| - | 154.69 | - out | | | | | | | |
| 469 | 154.71 | 0.11674 | 404 | 203 | 404 | 2 | 18 | 0 | 3.3e-13 | 3.9e-15 |
| - | 154.72 | - out | | | | | | | |
| - | 154.74 | - out | | | | | | | |
| - | 154.77 | - out | | | | | | | |
| - | 154.8 | - out | | | | | | | |
| 470 | 154.82 | 0.11674 | 406 | 204 | 406 | 2 | 18 | 0 | 1.1e-13 | 3.1e-15 |
| - | 154.83 | - out | | | | | | | |
| - | 154.85 | - out | | | | | | | |
| - | 154.88 | - out | | | | | | | |
| 471 | 154.89 | 0.070483 | 410 | 206 | 410 | 2 | 19 | 0 | 2.5e-14 | 4.1e-15 |

| | | | | | | | | | | |
|---|---|---|---|---|---|---|---|---|---|---|
| - | 154.91 | - out | | | | | | | | |
| - | 154.94 | - out | | | | | | | | |
| - | 154.96 | - out | | | | | | | | |
| 472 | 154.96 | 0.070483 | 412 | 207 | 412 | 2 | 19 | 0 | 1.1e-13 | 3.6e-15 |
| - | 154.99 | - out | | | | | | | | |
| - | 155.02 | - out | | | | | | | | |
| 473 | 155.03 | 0.070483 | 414 | 208 | 414 | 2 | 19 | 0 | 7.6e-14 | 5.4e-15 |
| - | 155.05 | - out | | | | | | | | |
| - | 155.07 | - out | | | | | | | | |
| - | 155.1 | - out | | | | | | | | |
| 474 | 155.1 | 0.070483 | 416 | 209 | 416 | 2 | 19 | 0 | 3.3e-14 | 4.7e-15 |
| - | 155.13 | - out | | | | | | | | |
| - | 155.16 | - out | | | | | | | | |
| 475 | 155.17 | 0.070483 | 418 | 210 | 418 | 2 | 19 | 0 | 1.1e-13 | 4.6e-15 |
| - | 155.18 | - out | | | | | | | | |
| - | 155.21 | - out | | | | | | | | |
| - | 155.24 | - out | | | | | | | | |
| 476 | 155.24 | 0.070483 | 420 | 211 | 420 | 2 | 19 | 0 | 4.2e-14 | 5.6e-15 |
| - | 155.27 | - out | | | | | | | | |
| - | 155.29 | - out | | | | | | | | |
| 477 | 155.32 | 0.070483 | 422 | 212 | 422 | 2 | 19 | 0 | 1.8e-14 | 4.4e-15 |
| - | 155.32 | - out | | | | | | | | |
| - | 155.35 | - out | | | | | | | | |
| - | 155.38 | - out | | | | | | | | |
| 478 | 155.39 | 0.070483 | 424 | 213 | 424 | 2 | 19 | 0 | 7e-14 | 4.6e-15 |
| - | 155.4 | - out | | | | | | | | |
| - | 155.43 | - out | | | | | | | | |
| 479 | 155.45 | 0.063435 | 426 | 214 | 426 | 2 | 19 | 0 | 1e-13 | 4.2e-15 |
| - | 155.46 | - out | | | | | | | | |
| - | 155.49 | - out | | | | | | | | |
| - | 155.51 | - out | | | | | | | | |
| 480 | 155.51 | 0.063435 | 428 | 215 | 428 | 2 | 19 | 0 | 1.2e-13 | 4.6e-15 |
| - | 155.54 | - out | | | | | | | | |
| - | 155.57 | - out | | | | | | | | |
| 481 | 155.58 | 0.063435 | 430 | 216 | 430 | 2 | 19 | 0 | 1e-13 | 4.9e-15 |
| - | 155.6 | - out | | | | | | | | |
| - | 155.62 | - out | | | | | | | | |
| 482 | 155.64 | 0.063435 | 432 | 217 | 432 | 2 | 19 | 0 | 7.6e-14 | 2.9e-15 |
| - | 155.65 | - out | | | | | | | | |
| - | 155.68 | - out | | | | | | | | |
| 483 | 155.7 | 0.063435 | 434 | 218 | 434 | 2 | 19 | 0 | 3.8e-13 | 3.9e-15 |
| - | 155.71 | - out | | | | | | | | |
| - | 155.73 | - out | | | | | | | | |
| - | 155.76 | - out | | | | | | | | |
| 484 | 155.77 | 0.063435 | 436 | 219 | 436 | 2 | 19 | 0 | 6.1e-13 | 4e-15 |
| - | 155.79 | - out | | | | | | | | |
| - | 155.82 | - out | | | | | | | | |
| 485 | 155.83 | 0.063435 | 438 | 220 | 438 | 2 | 19 | 0 | 2e-13 | 5e-15 |
| - | 155.84 | - out | | | | | | | | |
| - | 155.87 | - out | | | | | | | | |
| 486 | 155.89 | 0.063435 | 440 | 221 | 440 | 2 | 19 | 0 | 2.9e-13 | 5.2e-15 |
| - | 155.9 | - out | | | | | | | | |
| - | 155.93 | - out | | | | | | | | |
| - | 155.95 | - out | | | | | | | | |
| 487 | 155.96 | 0.063435 | 442 | 222 | 442 | 2 | 19 | 0 | 5.6e-14 | 3.7e-15 |
| - | 155.98 | - out | | | | | | | | |

| | | | | | | | | | | | |
|---|---|---|---|---|---|---|---|---|---|---|---|
| - | 156.01 | - | out | | | | | | | | |
| 488 | 156.02 | 0.063435 | | 444 | 223 | 444 | 2 | 19 | 0 | 7.4e-13 | 4e-15 |
| - | 156.04 | - | out | | | | | | | | |
| - | 156.06 | - | out | | | | | | | | |
| 489 | 156.08 | 0.063435 | | 446 | 224 | 446 | 2 | 19 | 0 | 3.2e-13 | 4.1e-15 |
| - | 156.09 | - | out | | | | | | | | |
| - | 156.12 | - | out | | | | | | | | |
| - | 156.15 | - | out | | | | | | | | |
| 490 | 156.15 | 0.063435 | | 448 | 225 | 448 | 2 | 19 | 0 | 3.8e-13 | 7.2e-15 |
| - | 156.17 | - | out | | | | | | | | |
| - | 156.2 | - | out | | | | | | | | |
| - | 156.23 | - | out | | | | | | | | |
| - | 156.26 | - | out | | | | | | | | |
| 491 | 156.27 | 0.12687 | | 450 | 226 | 450 | 2 | 19 | 0 | 6.8e-13 | 7.6e-15 |
| - | 156.28 | - | out | | | | | | | | |
| - | 156.31 | - | out | | | | | | | | |
| - | 156.34 | - | out | | | | | | | | |
| - | 156.37 | - | out | | | | | | | | |
| - | 156.39 | - | out | | | | | | | | |
| 492 | 156.4 | 0.12687 | | 452 | 227 | 452 | 2 | 19 | 0 | 6.7e-13 | 6.4e-15 |
| - | 156.42 | - | out | | | | | | | | |
| - | 156.45 | - | out | | | | | | | | |
| - | 156.48 | - | out | | | | | | | | |
| - | 156.5 | - | out | | | | | | | | |
| 493 | 156.53 | 0.12687 | | 454 | 228 | 454 | 2 | 19 | 0 | 5.2e-13 | 5.2e-15 |
| - | 156.53 | - | out | | | | | | | | |
| - | 156.56 | - | out | | | | | | | | |
| - | 156.59 | - | out | | | | | | | | |
| - | 156.61 | - | out | | | | | | | | |
| - | 156.64 | - | out | | | | | | | | |
| - | 156.67 | - | out | | | | | | | | |
| - | 156.7 | - | out | | | | | | | | |
| - | 156.72 | - | out | | | | | | | | |
| - | 156.75 | - | out | | | | | | | | |
| - | 156.78 | - | out | | | | | | | | |
| 494 | 156.78 | 0.25374 | | 456 | 229 | 456 | 2 | 19 | 0 | 1.2e-12 | 3.9e-15 |
| - | 156.81 | - | out | | | | | | | | |
| - | 156.83 | - | out | | | | | | | | |
| - | 156.86 | - | out | | | | | | | | |
| - | 156.89 | - | out | | | | | | | | |
| - | 156.92 | - | out | | | | | | | | |
| - | 156.94 | - | out | | | | | | | | |
| - | 156.97 | - | out | | | | | | | | |
| - | 157 | - | out | | | | | | | | |
| - | 157.03 | - | out | | | | | | | | |
| 495 | 157.04 | 0.25374 | | 458 | 230 | 458 | 2 | 19 | 0 | 1e-13 | 6.1e-15 |
| - | 157.05 | - | out | | | | | | | | |
| - | 157.08 | - | out | | | | | | | | |
| - | 157.11 | - | out | | | | | | | | |
| - | 157.14 | - | out | | | | | | | | |
| - | 157.16 | - | out | | | | | | | | |
| - | 157.19 | - | out | | | | | | | | |
| - | 157.22 | - | out | | | | | | | | |
| - | 157.25 | - | out | | | | | | | | |
| - | 157.27 | - | out | | | | | | | | |
| - | 157.3 | - | out | | | | | | | | |

| | | | | | | | | | |
|---|---|---|---|---|---|---|---|---|---|
| - | 157.33 | - out | | | | | | | |
| 496 | 157.33 | 0.29504 | 462 | 232 | 462 | 2 | 20 | 0 | 7e-13 | 3.8e-15 |
| - | 157.36 | - out | | | | | | | |
| - | 157.38 | - out | | | | | | | |
| - | 157.41 | - out | | | | | | | |
| - | 157.44 | - out | | | | | | | |
| - | 157.47 | - out | | | | | | | |
| - | 157.49 | - out | | | | | | | |
| 497 | 157.51 | 0.17678 | 466 | 234 | 466 | 2 | 21 | 0 | 4.8e-13 | 4.3e-15 |
| - | 157.52 | - out | | | | | | | |
| - | 157.55 | - out | | | | | | | |
| - | 157.58 | - out | | | | | | | |
| - | 157.6 | - out | | | | | | | |
| 498 | 157.62 | 0.11719 | 470 | 236 | 470 | 2 | 22 | 0 | 9.3e-13 | 5.8e-15 |
| - | 157.63 | - out | | | | | | | |
| - | 157.66 | - out | | | | | | | |
| - | 157.69 | - out | | | | | | | |
| - | 157.71 | - out | | | | | | | |
| - | 157.74 | - out | | | | | | | |
| 499 | 157.74 | 0.11719 | 472 | 237 | 472 | 2 | 22 | 0 | 3e-13 | 3.6e-15 |
| - | 157.77 | - out | | | | | | | |
| - | 157.8 | - out | | | | | | | |
| 500 | 157.81 | 0.067981 | 476 | 239 | 476 | 2 | 23 | 0 | 1e-13 | 3.5e-15 |
| - | 157.82 | - out | | | | | | | |
| - | 157.85 | - out | | | | | | | |
| 501 | 157.88 | 0.067981 | 478 | 240 | 478 | 2 | 23 | 0 | 1.8e-13 | 5.3e-15 |
| - | 157.88 | - out | | | | | | | |
| - | 157.91 | - out | | | | | | | |
| - | 157.93 | - out | | | | | | | |
| 502 | 157.94 | 0.061183 | 480 | 241 | 480 | 2 | 23 | 0 | 1.3e-13 | 4.4e-15 |
| - | 157.96 | - out | | | | | | | |
| - | 157.99 | - out | | | | | | | |
| 503 | 158 | 0.061183 | 482 | 242 | 482 | 2 | 23 | 0 | 1.6e-13 | 5.3e-15 |
| - | 158.02 | - out | | | | | | | |
| - | 158.04 | - out | | | | | | | |
| 504 | 158.06 | 0.061183 | 484 | 243 | 484 | 2 | 23 | 0 | 3.6e-14 | 4.2e-15 |
| - | 158.07 | - out | | | | | | | |
| - | 158.1 | - out | | | | | | | |
| 505 | 158.12 | 0.061183 | 486 | 244 | 486 | 2 | 23 | 0 | 5.2e-14 | 3.8e-15 |
| - | 158.13 | - out | | | | | | | |
| - | 158.15 | - out | | | | | | | |
| - | 158.18 | - out | | | | | | | |
| 506 | 158.19 | 0.066869 | 490 | 246 | 490 | 2 | 24 | 0 | 4.1e-14 | 7.2e-15 |
| - | 158.21 | - out | | | | | | | |
| - | 158.24 | - out | | | | | | | |
| 507 | 158.26 | 0.066869 | 492 | 247 | 492 | 2 | 24 | 0 | 2.7e-14 | 9.6e-15 |
| - | 158.26 | - out | | | | | | | |
| - | 158.29 | - out | | | | | | | |
| - | 158.32 | - out | | | | | | | |
| 508 | 158.32 | 0.066869 | 494 | 248 | 494 | 2 | 24 | 0 | 7.1e-14 | 8.2e-15 |
| - | 158.35 | - out | | | | | | | |
| - | 158.37 | - out | | | | | | | |
| 509 | 158.38 | 0.060182 | 496 | 249 | 496 | 2 | 24 | 0 | 3.7e-14 | 6.8e-15 |
| - | 158.4 | - out | | | | | | | |
| - | 158.43 | - out | | | | | | | |
| 510 | 158.44 | 0.060182 | 498 | 250 | 498 | 2 | 24 | 0 | 2.4e-14 | 4.8e-15 |

```
   -      158.46           - out
   -      158.48           - out
 511       158.5    0.060182      500  251  500     2    24       0  1.2e-14  6.2e-15
   -      158.51           - out
   -      158.54           - out
   -      158.57           - out
 512      158.58    0.077343      504  253  504     2    25       0  4.1e-14  6.7e-15
   -      158.59           - out
   -      158.62           - out
   -      158.65           - out
 513      158.66    0.077343      506  254  506     2    25       0    1e-13  1.1e-14
   -      158.68           - out
   -       158.7           - out
 514      158.73    0.069609      508  255  508     2    25       0  3.8e-13    1e-14
   -      158.73           - out
 515       158.8    0.069609      510  256  510     2    25       0  4.7e-13  5.3e-15
Number of vertex elements: 3
Number of boundary elements: 112
Number of vertex elements: 3
Number of boundary elements: 112
Number of elements: 534
Minimum element quality: 0.5661
Number of vertex elements: 4
Number of boundary elements: 162
Number of vertex elements: 5
Number of boundary elements: 212
Number of vertex elements: 6
Number of boundary elements: 266
Minimum element quality: 0.1631
Geometry shape function: Linear Lagrange
Time interval 3
Time-dependent solver (BDF)
Number of degrees of freedom solved for: 11758 (plus 7124 internal DOFs).
Nonsymmetric matrix found.
Scales for dependent variables:
Concentration (comp1.cOx): 2.9
Concentration (comp1.cRed): 1.9
Spatial mesh displacement (comp1.spatial.disp): 1.8e-07
Step        Time    Stepsize      Res  Jac  Sol Order Tfail NLfail   LinErr   LinRes
   -      158.73           - out
   -      158.73           - out
 516      158.76      0.0275        2    2    2     1     0       0  9.8e-14  2.7e-15
   -      158.76           - out
 517      158.78      0.0275        4    3    4     1     0       0  1.9e-13  3.6e-15
   -      158.79           - out
   -      158.81           - out
 518      158.84       0.055        6    4    6     2     0       0  1.9e-13  2.4e-15
   -      158.84           - out
   -      158.87           - out
 519      158.89       0.055        8    5    8     2     0       0  6.4e-13  4.1e-15
   -       158.9           - out
   -      158.92           - out
   -      158.95           - out
   -      158.98           - out
 520         159        0.11       10    6   10     2     0       0  4.9e-13  4.4e-15
   -      159.01           - out
```

| | | | | | | | | | | |
|---|---|---|---|---|---|---|---|---|---|---|
| - | 159.03 | - out | | | | | | | | |
| - | 159.06 | - out | | | | | | | | |
| - | 159.09 | - out | | | | | | | | |
| 521 | 159.11 | 0.11 | 12 | 7 | 12 | 2 | 0 | 0 | 9e-13 | 6.5e-15 |
| - | 159.12 | - out | | | | | | | | |
| - | 159.14 | - out | | | | | | | | |
| - | 159.17 | - out | | | | | | | | |
| - | 159.2 | - out | | | | | | | | |
| 522 | 159.22 | 0.11 | 14 | 8 | 14 | 2 | 0 | 0 | 1.1e-12 | 3.9e-15 |
| - | 159.23 | - out | | | | | | | | |
| - | 159.25 | - out | | | | | | | | |
| - | 159.28 | - out | | | | | | | | |
| - | 159.31 | - out | | | | | | | | |
| 523 | 159.33 | 0.11 | 16 | 9 | 16 | 2 | 0 | 0 | 8.9e-13 | 4.5e-15 |
| - | 159.34 | - out | | | | | | | | |
| - | 159.36 | - out | | | | | | | | |
| - | 159.39 | - out | | | | | | | | |
| - | 159.42 | - out | | | | | | | | |
| - | 159.45 | - out | | | | | | | | |
| - | 159.47 | - out | | | | | | | | |
| - | 159.5 | - out | | | | | | | | |
| - | 159.53 | - out | | | | | | | | |
| 524 | 159.55 | 0.22 | 18 | 10 | 18 | 2 | 0 | 0 | 1.4e-12 | 3.8e-15 |
| - | 159.56 | - out | | | | | | | | |
| - | 159.58 | - out | | | | | | | | |
| - | 159.61 | - out | | | | | | | | |
| - | 159.64 | - out | | | | | | | | |
| - | 159.67 | - out | | | | | | | | |
| - | 159.69 | - out | | | | | | | | |
| - | 159.72 | - out | | | | | | | | |
| - | 159.75 | - out | | | | | | | | |
| 525 | 159.77 | 0.22 | 20 | 11 | 20 | 2 | 0 | 0 | 6.4e-14 | 5.9e-15 |
| - | 159.78 | - out | | | | | | | | |
| - | 159.8 | - out | | | | | | | | |
| - | 159.83 | - out | | | | | | | | |
| - | 159.86 | - out | | | | | | | | |
| - | 159.89 | - out | | | | | | | | |
| - | 159.91 | - out | | | | | | | | |
| - | 159.94 | - out | | | | | | | | |
| - | 159.97 | - out | | | | | | | | |
| 526 | 159.99 | 0.21337 | 24 | 13 | 24 | 2 | 1 | 0 | 3.9e-13 | 2.7e-15 |
| - | 160 | - out | | | | | | | | |
| - | 160.02 | - out | | | | | | | | |
| - | 160.05 | - out | | | | | | | | |
| - | 160.08 | - out | | | | | | | | |
| - | 160.11 | - out | | | | | | | | |
| - | 160.13 | - out | | | | | | | | |
| - | 160.16 | - out | | | | | | | | |
| - | 160.19 | - out | | | | | | | | |
| 527 | 160.2 | 0.21337 | 26 | 14 | 26 | 2 | 1 | 0 | 1e-12 | 6.3e-15 |
| - | 160.22 | - out | | | | | | | | |
| - | 160.24 | - out | | | | | | | | |
| - | 160.27 | - out | | | | | | | | |
| 528 | 160.29 | 0.095507 | 30 | 16 | 30 | 2 | 2 | 0 | 1.2e-13 | 4.8e-15 |
| - | 160.3 | - out | | | | | | | | |
| - | 160.33 | - out | | | | | | | | |

| | | | | | | | | | | |
|---|---|---|---|---|---|---|---|---|---|---|
| - | 160.35 | - out | | | | | | | | |
| - | 160.38 | - out | | | | | | | | |
| 529 | 160.39 | 0.095507 | 32 | 17 | 32 | 2 | 2 | 0 | 2.6e-13 | 4.6e-15 |
| - | 160.41 | - out | | | | | | | | |
| - | 160.44 | - out | | | | | | | | |
| - | 160.46 | - out | | | | | | | | |
| 530 | 160.47 | 0.083902 | 34 | 18 | 34 | 2 | 2 | 0 | 1.7e-13 | 4.5e-15 |
| - | 160.49 | - out | | | | | | | | |
| - | 160.52 | - out | | | | | | | | |
| - | 160.55 | - out | | | | | | | | |
| 531 | 160.55 | 0.074598 | 36 | 19 | 36 | 2 | 2 | 0 | 4.7e-14 | 4.7e-15 |
| - | 160.57 | - out | | | | | | | | |
| - | 160.6 | - out | | | | | | | | |
| 532 | 160.62 | 0.074598 | 38 | 20 | 38 | 2 | 2 | 0 | 5.6e-14 | 5e-15 |
| - | 160.63 | - out | | | | | | | | |
| - | 160.66 | - out | | | | | | | | |
| - | 160.68 | - out | | | | | | | | |
| 533 | 160.7 | 0.074598 | 40 | 21 | 40 | 2 | 2 | 0 | 1.3e-13 | 4.8e-15 |
| - | 160.71 | - out | | | | | | | | |
| - | 160.74 | - out | | | | | | | | |
| - | 160.77 | - out | | | | | | | | |
| 534 | 160.77 | 0.074598 | 42 | 22 | 42 | 2 | 2 | 0 | 7.1e-14 | 4.8e-15 |
| - | 160.79 | - out | | | | | | | | |
| - | 160.82 | - out | | | | | | | | |
| 535 | 160.85 | 0.074598 | 44 | 23 | 44 | 2 | 2 | 0 | 1.7e-13 | 6.3e-15 |
| - | 160.85 | - out | | | | | | | | |
| - | 160.88 | - out | | | | | | | | |
| - | 160.9 | - out | | | | | | | | |
| 536 | 160.91 | 0.067138 | 46 | 24 | 46 | 2 | 2 | 0 | 7.8e-14 | 5.2e-15 |
| - | 160.93 | - out | | | | | | | | |
| - | 160.96 | - out | | | | | | | | |
| 537 | 160.98 | 0.067138 | 48 | 25 | 48 | 2 | 2 | 0 | 1.3e-13 | 5.1e-15 |
| - | 160.99 | - out | | | | | | | | |
| - | 161.01 | - out | | | | | | | | |
| - | 161.04 | - out | | | | | | | | |
| 538 | 161.05 | 0.067138 | 50 | 26 | 50 | 2 | 2 | 0 | 6.2e-14 | 5.6e-15 |
| - | 161.07 | - out | | | | | | | | |
| - | 161.1 | - out | | | | | | | | |
| 539 | 161.12 | 0.067138 | 52 | 27 | 52 | 2 | 2 | 0 | 2.1e-13 | 3.7e-15 |
| - | 161.12 | - out | | | | | | | | |
| - | 161.15 | - out | | | | | | | | |
| - | 161.18 | - out | | | | | | | | |
| 540 | 161.18 | 0.067138 | 54 | 28 | 54 | 2 | 2 | 0 | 1.5e-13 | 4.7e-15 |
| - | 161.21 | - out | | | | | | | | |
| - | 161.23 | - out | | | | | | | | |
| 541 | 161.25 | 0.067138 | 56 | 29 | 56 | 2 | 2 | 0 | 2.6e-13 | 3.5e-15 |
| - | 161.26 | - out | | | | | | | | |
| - | 161.29 | - out | | | | | | | | |
| - | 161.32 | - out | | | | | | | | |
| 542 | 161.32 | 0.067138 | 58 | 30 | 58 | 2 | 2 | 0 | 2.8e-13 | 3.9e-15 |
| - | 161.34 | - out | | | | | | | | |
| - | 161.37 | - out | | | | | | | | |
| 543 | 161.38 | 0.067138 | 60 | 31 | 60 | 2 | 2 | 0 | 4.8e-13 | 5.8e-15 |
| - | 161.4 | - out | | | | | | | | |
| - | 161.43 | - out | | | | | | | | |
| 544 | 161.45 | 0.067138 | 62 | 32 | 62 | 2 | 2 | 0 | 1.6e-13 | 2.8e-15 |

| | | | | | | | | | | |
|---|---|---|---|---|---|---|---|---|---|---|
| - | 161.45 | - out | | | | | | | | |
| - | 161.48 | - out | | | | | | | | |
| - | 161.51 | - out | | | | | | | | |
| 545 | 161.52 | 0.067138 | 64 | 33 | 64 | 2 | 2 | 0 | 4.3e-13 | 5.4e-15 |
| - | 161.54 | - out | | | | | | | | |
| - | 161.56 | - out | | | | | | | | |
| 546 | 161.59 | 0.067138 | 66 | 34 | 66 | 2 | 2 | 0 | 2.6e-13 | 4.8e-15 |
| - | 161.59 | - out | | | | | | | | |
| - | 161.62 | - out | | | | | | | | |
| - | 161.65 | - out | | | | | | | | |
| 547 | 161.65 | 0.067138 | 68 | 35 | 68 | 2 | 2 | 0 | 1e-12 | 5.1e-15 |
| - | 161.67 | - out | | | | | | | | |
| - | 161.7 | - out | | | | | | | | |
| - | 161.73 | - out | | | | | | | | |
| - | 161.76 | - out | | | | | | | | |
| - | 161.78 | - out | | | | | | | | |
| 548 | 161.79 | 0.13428 | 70 | 36 | 70 | 2 | 2 | 0 | 1.6e-12 | 5.7e-15 |
| - | 161.81 | - out | | | | | | | | |
| - | 161.84 | - out | | | | | | | | |
| - | 161.87 | - out | | | | | | | | |
| - | 161.89 | - out | | | | | | | | |
| - | 161.92 | - out | | | | | | | | |
| 549 | 161.92 | 0.13428 | 72 | 37 | 72 | 2 | 2 | 0 | 2.8e-12 | 9.1e-15 |
| - | 161.95 | - out | | | | | | | | |
| - | 161.98 | - out | | | | | | | | |
| - | 162 | - out | | | | | | | | |
| - | 162.03 | - out | | | | | | | | |
| 550 | 162.06 | 0.13428 | 74 | 38 | 74 | 2 | 2 | 0 | 6.7e-13 | 5.8e-15 |
| - | 162.06 | - out | | | | | | | | |
| - | 162.09 | - out | | | | | | | | |
| - | 162.11 | - out | | | | | | | | |
| - | 162.14 | - out | | | | | | | | |
| - | 162.17 | - out | | | | | | | | |
| 551 | 162.19 | 0.13428 | 76 | 39 | 76 | 2 | 2 | 0 | 6.5e-13 | 4.9e-15 |
| - | 162.2 | - out | | | | | | | | |
| - | 162.22 | - out | | | | | | | | |
| - | 162.25 | - out | | | | | | | | |
| - | 162.28 | - out | | | | | | | | |
| - | 162.31 | - out | | | | | | | | |
| - | 162.33 | - out | | | | | | | | |
| - | 162.36 | - out | | | | | | | | |
| - | 162.39 | - out | | | | | | | | |
| - | 162.42 | - out | | | | | | | | |
| - | 162.44 | - out | | | | | | | | |
| 552 | 162.46 | 0.26855 | 78 | 40 | 78 | 2 | 2 | 0 | 1.2e-13 | 6.5e-15 |
| - | 162.47 | - out | | | | | | | | |
| - | 162.5 | - out | | | | | | | | |
| - | 162.53 | - out | | | | | | | | |
| - | 162.55 | - out | | | | | | | | |
| - | 162.58 | - out | | | | | | | | |
| - | 162.61 | - out | | | | | | | | |
| - | 162.64 | - out | | | | | | | | |
| - | 162.66 | - out | | | | | | | | |
| - | 162.69 | - out | | | | | | | | |
| - | 162.72 | - out | | | | | | | | |
| 553 | 162.73 | 0.26855 | 80 | 41 | 80 | 2 | 2 | 0 | 3.2e-13 | 3.2e-15 |

| | | | | | | | | | | |
|---|---|---|---|---|---|---|---|---|---|---|
| - | 162.75 | - out | | | | | | | | |
| - | 162.77 | - out | | | | | | | | |
| - | 162.8 | - out | | | | | | | | |
| - | 162.83 | - out | | | | | | | | |
| - | 162.86 | - out | | | | | | | | |
| - | 162.88 | - out | | | | | | | | |
| 554 | 162.89 | 0.1614 | 84 | 43 | 84 | 2 | 3 | 0 | 1.7e-12 | 4.2e-15 |
| - | 162.91 | - out | | | | | | | | |
| - | 162.94 | - out | | | | | | | | |
| - | 162.97 | - out | | | | | | | | |
| - | 162.99 | - out | | | | | | | | |
| - | 163.02 | - out | | | | | | | | |
| - | 163.05 | - out | | | | | | | | |
| 555 | 163.05 | 0.1614 | 86 | 44 | 86 | 2 | 3 | 0 | 2.9e-13 | 3.4e-15 |
| - | 163.08 | - out | | | | | | | | |
| - | 163.1 | - out | | | | | | | | |
| - | 163.13 | - out | | | | | | | | |
| 556 | 163.15 | 0.095381 | 90 | 46 | 90 | 2 | 4 | 0 | 2.5e-13 | 4.3e-15 |
| - | 163.16 | - out | | | | | | | | |
| - | 163.19 | - out | | | | | | | | |
| - | 163.21 | - out | | | | | | | | |
| - | 163.24 | - out | | | | | | | | |
| 557 | 163.24 | 0.095381 | 92 | 47 | 92 | 2 | 4 | 0 | 1.5e-13 | 3.2e-15 |
| - | 163.27 | - out | | | | | | | | |
| - | 163.3 | - out | | | | | | | | |
| - | 163.32 | - out | | | | | | | | |
| 558 | 163.33 | 0.085069 | 94 | 48 | 94 | 2 | 4 | 0 | 1.9e-14 | 2.8e-15 |
| - | 163.35 | - out | | | | | | | | |
| - | 163.38 | - out | | | | | | | | |
| 559 | 163.4 | 0.071344 | 96 | 49 | 96 | 2 | 4 | 0 | 1e-13 | 6.1e-15 |
| - | 163.41 | - out | | | | | | | | |
| - | 163.43 | - out | | | | | | | | |
| - | 163.46 | - out | | | | | | | | |
| 560 | 163.46 | 0.06421 | 98 | 50 | 98 | 2 | 4 | 0 | 1e-13 | 5.5e-15 |
| - | 163.49 | - out | | | | | | | | |
| - | 163.52 | - out | | | | | | | | |
| 561 | 163.53 | 0.06421 | 100 | 51 | 100 | 2 | 4 | 0 | 9.4e-14 | 4.1e-15 |
| - | 163.54 | - out | | | | | | | | |
| - | 163.57 | - out | | | | | | | | |
| 562 | 163.59 | 0.06421 | 102 | 52 | 102 | 2 | 4 | 0 | 7.5e-15 | 9.4e-15 |
| - | 163.6 | - out | | | | | | | | |
| - | 163.63 | - out | | | | | | | | |
| - | 163.65 | - out | | | | | | | | |
| 563 | 163.65 | 0.06421 | 104 | 53 | 104 | 2 | 4 | 0 | 2.5e-14 | 3.9e-15 |
| - | 163.68 | - out | | | | | | | | |
| - | 163.71 | - out | | | | | | | | |
| 564 | 163.72 | 0.06421 | 106 | 54 | 106 | 2 | 4 | 0 | 2.9e-14 | 3.7e-15 |
| - | 163.74 | - out | | | | | | | | |
| - | 163.76 | - out | | | | | | | | |
| 565 | 163.78 | 0.06421 | 108 | 55 | 108 | 2 | 4 | 0 | 1.2e-14 | 4.8e-15 |
| - | 163.79 | - out | | | | | | | | |
| - | 163.82 | - out | | | | | | | | |
| - | 163.85 | - out | | | | | | | | |
| 566 | 163.85 | 0.06421 | 110 | 56 | 110 | 2 | 4 | 0 | 3.9e-14 | 7.1e-15 |
| - | 163.87 | - out | | | | | | | | |
| - | 163.9 | - out | | | | | | | | |

```
 567      163.91     0.06421      112   57  112      2     4       0  8.3e-14  4.2e-15
   -      163.93           - out
   -      163.96           - out
 568      163.97     0.06421      114   58  114      2     4       0    1e-14  6.3e-15
   -      163.98           - out
   -      164.01           - out
   -      164.04           - out
 569      164.04     0.06421      116   59  116      2     4       0  1.1e-13  4.5e-15
   -      164.07           - out
   -      164.09           - out
 570       164.1     0.06421      118   60  118      2     4       0  9.3e-14  4.9e-15
   -      164.12           - out
   -      164.15           - out
 571      164.17     0.06421      120   61  120      2     4       0  2.5e-13  3.3e-15
   -      164.18           - out
   -       164.2           - out
   -      164.23           - out
 572      164.23     0.06421      122   62  122      2     4       0  2.3e-13  4.8e-15
   -      164.26           - out
   -      164.29           - out
 573       164.3     0.06421      124   63  124      2     4       0  5.2e-13  8.9e-15
   -      164.31           - out
   -      164.34           - out
 574      164.36     0.06421      126   64  126      2     4       0  2.9e-13    3e-15
   -      164.37           - out
   -       164.4           - out
   -      164.42           - out
 575      164.42     0.06421      128   65  128      2     4       0  6.5e-13  5.7e-15
   -      164.45           - out
   -      164.48           - out
 576      164.49     0.06421      130   66  130      2     4       0  1.6e-12  3.9e-15
   -      164.51           - out
   -      164.53           - out
 577      164.55     0.06421      132   67  132      2     4       0  1.2e-12  5.3e-15
   -      164.56           - out
   -      164.59           - out
   -      164.62           - out
   -      164.64           - out
   -      164.67           - out
 578      164.68     0.12842      134   68  134      2     4       0  3.6e-12  4.7e-15
   -       164.7           - out
   -      164.73           - out
   -      164.75           - out
   -      164.78           - out
   -      164.81           - out
 579      164.81     0.12842      136   69  136      2     4       0    2e-12  7.4e-15
   -      164.84           - out
   -      164.86           - out
   -      164.89           - out
   -      164.92           - out
 580      164.94     0.12842      138   70  138      2     4       0  1.3e-12  4.7e-15
   -      164.95           - out
   -      164.97           - out
   -         165           - out
 581      165.19     0.25684      140   71  140      2     4       0  4.2e-13  4.3e-15
Time-stepping completed.
```

```
Geometry shape function: Linear Lagrange
Solution time: 703 s. (11 minutes, 43 seconds)
Physical memory: 2.78 GB
Virtual memory: 2.87 GB
Ended at May 31, 2023 11:03:22 AM.
----- Time-Dependent Solver 1 in Study 3 (CV 21 to 30)/Solution 6 (sol6) ------>
```

**Advanced (aDef)**

### ASSEMBLY SETTINGS

| Description | Value |
|---|---|
| Reuse sparsity pattern | On |

**Fully Coupled 1 (fc1)**

### GENERAL

| Description | Value |
|---|---|
| Linear solver | Direct 1 |

### METHOD AND TERMINATION

| Description | Value |
|---|---|
| Damping factor | 0.9 |
| Jacobian update | Once per time step |
| Maximum number of iterations | 8 |
| Stabilization and acceleration | Anderson acceleration |
| Dimension of iteration space | 5 |

**Automatic Remeshing 1 (ar1)**

### GENERAL

| Description | Value |
|---|---|
| Remesh in geometry | Geometry 1 |

### CONDITION FOR REMESHING

| Description | Value |
|---|---|
| Condition type | Distortion |

### OUTPUT

| Description | Value |
|---|---|
| Solution | Remeshed Solution 3 |
| Meshes | {mesh4, mesh5} |

# 6 Study 4 (CV 31 to 38)

COMPUTATION INFORMATION

| | |
|---|---|
| Computation time | 10 min 42 s |

## 6.1 TIME DEPENDENT

| Times | Unit |
|---|---|
| range(30*t_cv,t_tot/nb/200,38*t_cv) | s |

STUDY SETTINGS

| Description | Value |
|---|---|
| Include geometric nonlinearity | Off |

STUDY SETTINGS

| Description | Value |
|---|---|
| Output times | {165.00000000000003, 165.02750000000003, 165.05500000000004, 165.08250000000004, 165.11000000000004, 165.13750000000002, 165.16500000000002, 165.19250000000002, 165.22000000000003, 165.24750000000003, 165.27500000000003, 165.30250000000004, 165.33000000000004, 165.35750000000002, 165.38500000000002, 165.41250000000002, 165.44000000000003, 165.46750000000003, 165.49500000000003, 165.52250000000004, 165.55000000000004, 165.57750000000001, 165.60500000000002, 165.63250000000002, 165.66000000000003, 165.68750000000003, 165.71500000000003, 165.74250000000004, 165.77000000000004, 165.79750000000004, 165.82500000000002, 165.85250000000002, 165.88000000000002, 165.90750000000003, 165.93500000000003, 165.96250000000003, 165.99000000000004, 166.01750000000004, 166.04500000000002, 166.07250000000002, 166.10000000000002, 166.12750000000003, 166.15500000000003, 166.18250000000003, 166.21000000000004, 166.23750000000004, 166.26500000000001, 166.29250000000002, 166.32000000000002, 166.34750000000003, 166.37500000000003, 166.40250000000003, 166.43000000000004, 166.45750000000004, 166.48500000000004, 166.51250000000002, 166.54000000000002, 166.56750000000002, 166.59500000000003, 166.62250000000003, 166.65000000000003, 166.67750000000004, 166.70500000000004, 166.73250000000002, 166.76000000000002, 166.78750000000002, 166.81500000000003, 166.84250000000003, 166.87000000000003, 166.89750000000004, 166.92500000000004, 166.95250000000001, 166.98000000000002, 167.00750000000002, 167.03500000000003, 167.06250000000003, 167.09000000000003, 167.11750000000004, 167.14500000000004, 167.17250000000004, 167.20000000000002, 167.22750000000002, 167.25500000000002, 167.28250000000003, 167.31000000000003, 167.33750000000003, 167.36500000000004, 167.39250000000004, 167.42000000000002, 167.44750000000002, 167.47500000000002, 167.50250000000003, 167.53000000000003, |

| Description | Value |
|---|---|
| | 167.55750000000003, 167.58500000000004, 167.61250000000004, 167.64000000000004, 167.66750000000002, 167.69500000000002, 167.72250000000003, 167.75000000000003, 167.77750000000003, 167.80500000000004, 167.83250000000004, 167.86000000000004, 167.88750000000002, 167.91500000000002, 167.94250000000002, 167.97000000000003, 167.99750000000003, 168.02500000000003, 168.05250000000004, 168.08000000000004, 168.10750000000002, 168.13500000000002, 168.16250000000002, 168.19000000000003, 168.21750000000003, 168.24500000000003, 168.27250000000004, 168.30000000000004, 168.32750000000004, 168.35500000000002, 168.38250000000002, 168.41000000000003, 168.43750000000003, 168.46500000000003, 168.49250000000004, 168.52000000000004, 168.54750000000004, 168.57500000000002, 168.60250000000002, 168.63000000000002, 168.65750000000003, 168.68500000000003, 168.71250000000003, 168.74000000000004, 168.76750000000004, 168.79500000000002, 168.82250000000002, 168.85000000000002, 168.87750000000003, 168.90500000000003, 168.93250000000003, 168.96000000000004, 168.98750000000004, 169.01500000000004, 169.04250000000002, 169.07000000000002, 169.09750000000003, 169.12500000000003, 169.15250000000003, 169.18000000000004, 169.20750000000004, 169.23500000000004, 169.26250000000002, 169.29000000000002, 169.31750000000002, 169.34500000000003, 169.37250000000003, 169.40000000000003, 169.42750000000004, 169.45500000000004, 169.48250000000002, 169.51000000000002, 169.53750000000002, 169.56500000000003, 169.59250000000003, 169.62000000000003, 169.64750000000004, 169.67500000000004, 169.70250000000004, 169.73000000000002, 169.75750000000002, 169.78500000000003, 169.81250000000003, 169.84000000000003, 169.86750000000004, 169.89500000000004, 169.92250000000004, 169.95000000000002, 169.97750000000002, 170.00500000000002, 170.03250000000003, 170.06000000000003, 170.08750000000003, 170.11500000000004, 170.14250000000004, 170.17000000000002, 170.19750000000002, 170.22500000000002, 170.25250000000003, 170.28000000000003, 170.30750000000003, 170.33500000000004, 170.36250000000004, 170.39000000000004, 170.41750000000002, 170.44500000000002, 170.47250000000003, 170.50000000000003, 170.52750000000003, 170.55500000000004, 170.58250000000004, 170.61000000000004, 170.63750000000002, 170.66500000000002, 170.69250000000002, 170.72000000000003, 170.74750000000003, 170.77500000000003, 170.80250000000004, 170.83000000000004, 170.85750000000002, 170.88500000000002, 170.91250000000002, 170.94000000000003, 170.96750000000003, 170.99500000000003, 171.02250000000004, 171.05000000000004, 171.07750000000004, 171.10500000000002, 171.13250000000002, 171.16000000000003, 171.18750000000003, 171.21500000000003, 171.24250000000004, 171.27000000000004, 171.29750000000004, 171.32500000000002, 171.35250000000002, 171.38000000000002, 171.40750000000003, 171.43500000000003, 171.46250000000003, 171.49000000000004, |

| Description | Value |
| --- | --- |
| | 171.51750000000004, 171.54500000000002, 171.57250000000002, 171.60000000000002, 171.62750000000003, 171.65500000000003, 171.68250000000003, 171.71000000000004, 171.73750000000004, 171.76500000000004, 171.79250000000002, 171.82000000000002, 171.84750000000003, 171.87500000000003, 171.90250000000003, 171.93000000000004, 171.95750000000004, 171.98500000000004, 172.01250000000002, 172.04000000000002, 172.06750000000002, 172.09500000000003, 172.12250000000003, 172.15000000000003, 172.17750000000004, 172.20500000000004, 172.23250000000002, 172.26000000000002, 172.28750000000002, 172.31500000000003, 172.34250000000003, 172.37000000000003, 172.39750000000004, 172.42500000000004, 172.45250000000004, 172.48000000000002, 172.50750000000002, 172.53500000000003, 172.56250000000003, 172.59000000000003, 172.61750000000004, 172.64500000000004, 172.67250000000004, 172.70000000000002, 172.72750000000002, 172.75500000000002, 172.78250000000003, 172.81000000000003, 172.83750000000003, 172.86500000000004, 172.89250000000004, 172.92000000000002, 172.94750000000002, 172.97500000000002, 173.00250000000003, 173.03000000000003, 173.05750000000003, 173.08500000000004, 173.11250000000004, 173.14000000000004, 173.16750000000002, 173.19500000000002, 173.22250000000003, 173.25000000000003, 173.27750000000003, 173.30500000000004, 173.33250000000004, 173.36000000000004, 173.38750000000002, 173.41500000000002, 173.44250000000002, 173.47000000000003, 173.49750000000003, 173.52500000000003, 173.55250000000004, 173.58000000000004, 173.60750000000002, 173.63500000000002, 173.66250000000002, 173.69000000000003, 173.71750000000003, 173.74500000000003, 173.77250000000004, 173.80000000000004, 173.82750000000004, 173.85500000000002, 173.88250000000002, 173.91000000000003, 173.93750000000003, 173.96500000000003, 173.99250000000004, 174.02000000000004, 174.04750000000004, 174.07500000000002, 174.10250000000002, 174.13000000000002, 174.15750000000003, 174.18500000000003, 174.21250000000003, 174.24000000000004, 174.26750000000004, 174.29500000000002, 174.32250000000002, 174.35000000000002, 174.37750000000003, 174.40500000000003, 174.43250000000003, 174.46000000000004, 174.48750000000004, 174.51500000000004, 174.54250000000002, 174.57000000000002, 174.59750000000003, 174.62500000000003, 174.65250000000003, 174.68000000000004, 174.70750000000004, 174.73500000000004, 174.76250000000002, 174.79000000000002, 174.81750000000002, 174.84500000000003, 174.87250000000003, 174.90000000000003, 174.92750000000004, 174.95500000000004, 174.98250000000002, 175.01000000000002, 175.03750000000002, 175.06500000000003, 175.09250000000003, 175.12000000000003, 175.14750000000004, 175.17500000000004, 175.20250000000004, 175.23000000000002, 175.25750000000002, 175.28500000000003, 175.31250000000003, 175.34000000000003, 175.36750000000004, 175.39500000000004, 175.42250000000004, 175.45000000000002, |

| Description | Value |
|---|---|
| | 175.47750000000002, 175.50500000000002, 175.53250000000003, 175.56000000000003, 175.58750000000003, 175.61500000000004, 175.64250000000004, 175.67000000000002, 175.69750000000002, 175.72500000000002, 175.75250000000003, 175.78000000000003, 175.80750000000003, 175.83500000000004, 175.86250000000004, 175.89000000000004, 175.91750000000002, 175.94500000000002, 175.97250000000003, 176.00000000000003, 176.02750000000003, 176.05500000000004, 176.08250000000004, 176.11000000000004, 176.13750000000002, 176.16500000000002, 176.19250000000002, 176.22000000000003, 176.24750000000003, 176.27500000000003, 176.30250000000004, 176.33000000000004, 176.35750000000002, 176.38500000000002, 176.41250000000002, 176.44000000000003, 176.46750000000003, 176.49500000000003, 176.52250000000004, 176.55000000000004, 176.57750000000004, 176.60500000000002, 176.63250000000002, 176.66000000000003, 176.68750000000003, 176.71500000000003, 176.74250000000004, 176.77000000000004, 176.79750000000004, 176.82500000000002, 176.85250000000002, 176.88000000000002, 176.90750000000003, 176.93500000000003, 176.96250000000003, 176.99000000000004, 177.01750000000004, 177.04500000000002, 177.07250000000002, 177.10000000000002, 177.12750000000003, 177.15500000000003, 177.18250000000003, 177.21000000000004, 177.23750000000004, 177.26500000000004, 177.29250000000002, 177.32000000000002, 177.34750000000003, 177.37500000000003, 177.40250000000003, 177.43000000000004, 177.45750000000004, 177.48500000000004, 177.51250000000002, 177.54000000000002, 177.56750000000002, 177.59500000000003, 177.62250000000003, 177.65000000000003, 177.67750000000004, 177.70500000000004, 177.73250000000002, 177.76000000000002, 177.78750000000002, 177.81500000000003, 177.84250000000003, 177.87000000000003, 177.89750000000004, 177.92500000000004, 177.95250000000004, 177.98000000000002, 178.00750000000002, 178.03500000000003, 178.06250000000003, 178.09000000000003, 178.11750000000004, 178.14500000000004, 178.17250000000004, 178.20000000000002, 178.22750000000002, 178.25500000000002, 178.28250000000003, 178.31000000000003, 178.33750000000003, 178.36500000000004, 178.39250000000004, 178.42000000000002, 178.44750000000002, 178.47500000000002, 178.50250000000003, 178.53000000000003, 178.55750000000003, 178.58500000000004, 178.61250000000004, 178.64000000000004, 178.66750000000002, 178.69500000000002, 178.72250000000003, 178.75000000000003, 178.77750000000003, 178.80500000000004, 178.83250000000004, 178.86000000000004, 178.88750000000002, 178.91500000000002, 178.94250000000002, 178.97000000000003, 178.99750000000003, 179.02500000000003, 179.05250000000004, 179.08000000000004, 179.10750000000002, 179.13500000000002, 179.16250000000002, 179.19000000000003, 179.21750000000003, 179.24500000000003, 179.27250000000004, 179.30000000000004, 179.32750000000004, 179.35500000000002, 179.38250000000002, 179.41000000000003, |

| Description | Value |
|---|---|
| | 179.43750000000003, 179.46500000000003, 179.49250000000004, 179.52000000000004, 179.54750000000004, 179.57500000000002, 179.60250000000002, 179.63000000000002, 179.65750000000003, 179.68500000000003, 179.71250000000003, 179.74000000000004, 179.76750000000004, 179.79500000000002, 179.82250000000002, 179.85000000000002, 179.87750000000003, 179.90500000000003, 179.93250000000003, 179.96000000000004, 179.98750000000004, 180.01500000000004, 180.04250000000002, 180.07000000000002, 180.09750000000003, 180.12500000000003, 180.15250000000003, 180.18000000000004, 180.20750000000004, 180.23500000000004, 180.26250000000005, 180.29000000000002, 180.31750000000002, 180.34500000000003, 180.37250000000003, 180.40000000000003, 180.42750000000004, 180.45500000000004, 180.48250000000002, 180.51000000000002, 180.53750000000002, 180.56500000000003, 180.59250000000003, 180.62000000000003, 180.64750000000004, 180.67500000000004, 180.70250000000004, 180.73000000000002, 180.75750000000002, 180.78500000000003, 180.81250000000003, 180.84000000000003, 180.86750000000004, 180.89500000000004, 180.92250000000004, 180.95000000000005, 180.97750000000002, 181.00500000000002, 181.03250000000003, 181.06000000000003, 181.08750000000003, 181.11500000000004, 181.14250000000004, 181.17000000000002, 181.19750000000002, 181.22500000000002, 181.25250000000003, 181.28000000000003, 181.30750000000003, 181.33500000000004, 181.36250000000004, 181.39000000000004, 181.41750000000002, 181.44500000000002, 181.47250000000003, 181.50000000000003, 181.52750000000003, 181.55500000000004, 181.58250000000004, 181.61000000000004, 181.63750000000005, 181.66500000000002, 181.69250000000002, 181.72000000000003, 181.74750000000003, 181.77500000000003, 181.80250000000004, 181.83000000000004, 181.85750000000002, 181.88500000000002, 181.91250000000002, 181.94000000000003, 181.96750000000003, 181.99500000000003, 182.02250000000004, 182.05000000000004, 182.07750000000004, 182.10500000000002, 182.13250000000002, 182.16000000000003, 182.18750000000003, 182.21500000000003, 182.24250000000004, 182.27000000000004, 182.29750000000004, 182.32500000000005, 182.35250000000002, 182.38000000000002, 182.40750000000003, 182.43500000000003, 182.46250000000003, 182.49000000000004, 182.51750000000004, 182.54500000000002, 182.57250000000002, 182.60000000000002, 182.62750000000003, 182.65500000000003, 182.68250000000003, 182.71000000000004, 182.73750000000004, 182.76500000000004, 182.79250000000002, 182.82000000000002, 182.84750000000003, 182.87500000000003, 182.90250000000003, 182.93000000000004, 182.95750000000004, 182.98500000000004, 183.01250000000005, 183.04000000000002, 183.06750000000002, 183.09500000000003, 183.12250000000003, 183.15000000000003, 183.17750000000004, 183.20500000000004, 183.23250000000002, 183.26000000000002, 183.28750000000002, 183.31500000000003, 183.34250000000003, 183.37000000000003, |

| Description | Value |
|---|---|
| | 183.39750000000004, 183.42500000000004, 183.45250000000004, 183.48000000000002, 183.50750000000002, 183.53500000000003, 183.56250000000003, 183.59000000000003, 183.61750000000004, 183.64500000000004, 183.67250000000004, 183.70000000000005, 183.72750000000002, 183.75500000000002, 183.78250000000003, 183.81000000000003, 183.83750000000003, 183.86500000000004, 183.89250000000004, 183.92000000000002, 183.94750000000002, 183.97500000000002, 184.00250000000003, 184.03000000000003, 184.05750000000003, 184.08500000000004, 184.11250000000004, 184.14000000000004, 184.16750000000002, 184.19500000000002, 184.22250000000003, 184.25000000000003, 184.27750000000003, 184.30500000000004, 184.33250000000004, 184.36000000000004, 184.38750000000005, 184.41500000000002, 184.44250000000002, 184.47000000000003, 184.49750000000003, 184.52500000000003, 184.55250000000004, 184.58000000000004, 184.60750000000002, 184.63500000000002, 184.66250000000002, 184.69000000000003, 184.71750000000003, 184.74500000000003, 184.77250000000004, 184.80000000000004, 184.82750000000004, 184.85500000000002, 184.88250000000002, 184.91000000000003, 184.93750000000003, 184.96500000000003, 184.99250000000004, 185.02000000000004, 185.04750000000004, 185.07500000000005, 185.10250000000002, 185.13000000000002, 185.15750000000003, 185.18500000000003, 185.21250000000003, 185.24000000000004, 185.26750000000004, 185.29500000000002, 185.32250000000002, 185.35000000000002, 185.37750000000003, 185.40500000000003, 185.43250000000003, 185.46000000000004, 185.48750000000004, 185.51500000000004, 185.54250000000002, 185.57000000000002, 185.59750000000003, 185.62500000000003, 185.65250000000003, 185.68000000000004, 185.70750000000004, 185.73500000000004, 185.76250000000005, 185.79000000000002, 185.81750000000002, 185.84500000000003, 185.87250000000003, 185.90000000000003, 185.92750000000004, 185.95500000000004, 185.98250000000002, 186.01000000000002, 186.03750000000002, 186.06500000000003, 186.09250000000003, 186.12000000000003, 186.14750000000004, 186.17500000000004, 186.20250000000004, 186.23000000000002, 186.25750000000002, 186.28500000000003, 186.31250000000003, 186.34000000000003, 186.36750000000004, 186.39500000000004, 186.42250000000004, 186.45000000000005, 186.47750000000002, 186.50500000000002, 186.53250000000003, 186.56000000000003, 186.58750000000003, 186.61500000000004, 186.64250000000004, 186.67000000000002, 186.69750000000002, 186.72500000000002, 186.75250000000003, 186.78000000000003, 186.80750000000003, 186.83500000000004, 186.86250000000004, 186.89000000000004, 186.91750000000002, 186.94500000000002, 186.97250000000003, 187.00000000000003, 187.02750000000003, 187.05500000000004, 187.08250000000004, 187.11000000000004, 187.13750000000005, 187.16500000000002, 187.19250000000002, 187.22000000000003, 187.24750000000003, 187.27500000000003, 187.30250000000004, 187.33000000000004, |

| Description | Value |
| --- | --- |
| | 187.35750000000002, 187.38500000000002, 187.41250000000002, 187.44000000000003, 187.46750000000003, 187.49500000000003, 187.52250000000004, 187.55000000000004, 187.57750000000004, 187.60500000000002, 187.63250000000002, 187.66000000000003, 187.68750000000003, 187.71500000000003, 187.74250000000004, 187.77000000000004, 187.79750000000004, 187.82500000000005, 187.85250000000002, 187.88000000000002, 187.90750000000003, 187.93500000000003, 187.96250000000003, 187.99000000000004, 188.01750000000004, 188.04500000000002, 188.07250000000002, 188.10000000000002, 188.12750000000003, 188.15500000000003, 188.18250000000003, 188.21000000000004, 188.23750000000004, 188.26500000000004, 188.29250000000002, 188.32000000000002, 188.34750000000003, 188.37500000000003, 188.40250000000003, 188.43000000000004, 188.45750000000004, 188.48500000000004, 188.51250000000005, 188.54000000000002, 188.56750000000002, 188.59500000000003, 188.62250000000003, 188.65000000000003, 188.67750000000004, 188.70500000000004, 188.73250000000002, 188.76000000000002, 188.78750000000002, 188.81500000000003, 188.84250000000003, 188.87000000000003, 188.89750000000004, 188.92500000000004, 188.95250000000004, 188.98000000000002, 189.00750000000002, 189.03500000000003, 189.06250000000003, 189.09000000000003, 189.11750000000004, 189.14500000000004, 189.17250000000004, 189.20000000000005, 189.22750000000002, 189.25500000000002, 189.28250000000003, 189.31000000000003, 189.33750000000003, 189.36500000000004, 189.39250000000004, 189.42000000000002, 189.44750000000002, 189.47500000000002, 189.50250000000003, 189.53000000000003, 189.55750000000003, 189.58500000000004, 189.61250000000004, 189.64000000000004, 189.66750000000002, 189.69500000000002, 189.72250000000003, 189.75000000000003, 189.77750000000003, 189.80500000000004, 189.83250000000004, 189.86000000000004, 189.88750000000005, 189.91500000000002, 189.94250000000002, 189.97000000000003, 189.99750000000003, 190.02500000000003, 190.05250000000004, 190.08000000000004, 190.10750000000002, 190.13500000000002, 190.16250000000002, 190.19000000000003, 190.21750000000003, 190.24500000000003, 190.27250000000004, 190.30000000000004, 190.32750000000004, 190.35500000000002, 190.38250000000002, 190.41000000000003, 190.43750000000003, 190.46500000000003, 190.49250000000004, 190.52000000000004, 190.54750000000004, 190.57500000000005, 190.60250000000002, 190.63000000000002, 190.65750000000003, 190.68500000000003, 190.71250000000003, 190.74000000000004, 190.76750000000004, 190.79500000000002, 190.82250000000005, 190.85000000000002, 190.87750000000003, 190.90500000000003, 190.93250000000003, 190.96000000000004, 190.98750000000004, 191.01500000000004, 191.04250000000002, 191.07000000000002, 191.09750000000003, 191.12500000000003, 191.15250000000003, 191.18000000000004, 191.20750000000004, 191.23500000000004, 191.26250000000005, 191.29000000000002, |

| Description | Value |
| --- | --- |
| | 191.31750000000002, 191.34500000000003, 191.37250000000003, 191.40000000000003, 191.42750000000004, 191.45500000000004, 191.48250000000002, 191.51000000000005, 191.53750000000002, 191.56500000000003, 191.59250000000003, 191.62000000000003, 191.64750000000004, 191.67500000000004, 191.70250000000004, 191.73000000000002, 191.75750000000002, 191.78500000000003, 191.81250000000003, 191.84000000000003, 191.86750000000004, 191.89500000000004, 191.92250000000004, 191.95000000000005, 191.97750000000002, 192.00500000000002, 192.03250000000003, 192.06000000000003, 192.08750000000003, 192.11500000000004, 192.14250000000004, 192.17000000000004, 192.19750000000005, 192.22500000000002, 192.25250000000003, 192.28000000000003, 192.30750000000003, 192.33500000000004, 192.36250000000004, 192.39000000000004, 192.41750000000002, 192.44500000000002, 192.47250000000003, 192.50000000000003, 192.52750000000003, 192.55500000000004, 192.58250000000004, 192.61000000000004, 192.63750000000005, 192.66500000000002, 192.69250000000002, 192.72000000000003, 192.74750000000003, 192.77500000000003, 192.80250000000004, 192.83000000000004, 192.85750000000004, 192.88500000000005, 192.91250000000002, 192.94000000000003, 192.96750000000003, 192.99500000000003, 193.02250000000004, 193.05000000000004, 193.07750000000004, 193.10500000000002, 193.13250000000002, 193.16000000000003, 193.18750000000003, 193.21500000000003, 193.24250000000004, 193.27000000000004, 193.29750000000004, 193.32500000000005, 193.35250000000002, 193.38000000000002, 193.40750000000003, 193.43500000000003, 193.46250000000003, 193.49000000000004, 193.51750000000004, 193.54500000000004, 193.57250000000005, 193.60000000000002, 193.62750000000003, 193.65500000000003, 193.68250000000003, 193.71000000000004, 193.73750000000004, 193.76500000000004, 193.79250000000002, 193.82000000000002, 193.84750000000003, 193.87500000000003, 193.90250000000003, 193.93000000000004, 193.95750000000004, 193.98500000000004, 194.01250000000005, 194.04000000000002, 194.06750000000002, 194.09500000000003, 194.12250000000003, 194.15000000000003, 194.17750000000004, 194.20500000000004, 194.23250000000004, 194.26000000000005, 194.28750000000002, 194.31500000000003, 194.34250000000003, 194.37000000000003, 194.39750000000004, 194.42500000000004, 194.45250000000004, 194.48000000000002, 194.50750000000002, 194.53500000000003, 194.56250000000003, 194.59000000000003, 194.61750000000004, 194.64500000000004, 194.67250000000004, 194.70000000000005, 194.72750000000002, 194.75500000000002, 194.78250000000003, 194.81000000000003, 194.83750000000003, 194.86500000000004, 194.89250000000004, 194.92000000000004, 194.94750000000005, 194.97500000000002, 195.00250000000003, 195.03000000000003, 195.05750000000003, 195.08500000000004, 195.11250000000004, 195.14000000000004, 195.16750000000002, 195.19500000000002, 195.22250000000003, 195.25000000000003, |

| Description | Value |
| --- | --- |
| | 195.27750000000003, 195.30500000000004, 195.33250000000004, 195.36000000000004, 195.38750000000005, 195.41500000000002, 195.44250000000002, 195.47000000000003, 195.49750000000003, 195.52500000000003, 195.55250000000004, 195.58000000000004, 195.60750000000004, 195.63500000000005, 195.66250000000002, 195.69000000000003, 195.71750000000003, 195.74500000000003, 195.77250000000004, 195.80000000000004, 195.82750000000004, 195.85500000000002, 195.88250000000002, 195.91000000000003, 195.93750000000003, 195.96500000000003, 195.99250000000004, 196.02000000000004, 196.04750000000004, 196.07500000000005, 196.10250000000002, 196.13000000000002, 196.15750000000003, 196.18500000000003, 196.21250000000003, 196.24000000000004, 196.26750000000004, 196.29500000000004, 196.32250000000005, 196.35000000000002, 196.37750000000003, 196.40500000000003, 196.43250000000003, 196.46000000000004, 196.48750000000004, 196.51500000000004, 196.54250000000002, 196.57000000000002, 196.59750000000003, 196.62500000000003, 196.65250000000003, 196.68000000000004, 196.70750000000004, 196.73500000000004, 196.76250000000005, 196.79000000000002, 196.81750000000002, 196.84500000000003, 196.87250000000003, 196.90000000000003, 196.92750000000004, 196.95500000000004, 196.98250000000004, 197.01000000000005, 197.03750000000002, 197.06500000000003, 197.09250000000003, 197.12000000000003, 197.14750000000004, 197.17500000000004, 197.20250000000004, 197.23000000000002, 197.25750000000005, 197.28500000000003, 197.31250000000003, 197.34000000000003, 197.36750000000004, 197.39500000000004, 197.42250000000004, 197.45000000000005, 197.47750000000002, 197.50500000000002, 197.53250000000003, 197.56000000000003, 197.58750000000003, 197.61500000000004, 197.64250000000004, 197.67000000000002, 197.69750000000005, 197.72500000000002, 197.75250000000003, 197.78000000000003, 197.80750000000003, 197.83500000000004, 197.86250000000004, 197.89000000000004, 197.91750000000002, 197.94500000000005, 197.97250000000003, 198.00000000000003, 198.02750000000003, 198.05500000000004, 198.08250000000004, 198.11000000000004, 198.13750000000005, 198.16500000000002, 198.19250000000002, 198.22000000000003, 198.24750000000003, 198.27500000000003, 198.30250000000004, 198.33000000000004, 198.35750000000002, 198.38500000000005, 198.41250000000002, 198.44000000000003, 198.46750000000003, 198.49500000000003, 198.52250000000004, 198.55000000000004, 198.57750000000004, 198.60500000000002, 198.63250000000005, 198.66000000000003, 198.68750000000003, 198.71500000000003, 198.74250000000004, 198.77000000000004, 198.79750000000004, 198.82500000000005, 198.85250000000002, 198.88000000000002, 198.90750000000003, 198.93500000000003, 198.96250000000003, 198.99000000000004, 199.01750000000004, 199.04500000000002, 199.07250000000005, 199.10000000000002, 199.12750000000003, 199.15500000000003, 199.18250000000003, 199.21000000000004, |

| Description | Value |
| --- | --- |
| | 199.23750000000004, 199.26500000000004, 199.29250000000002, 199.32000000000005, 199.34750000000003, 199.37500000000003, 199.40250000000003, 199.43000000000004, 199.45750000000004, 199.48500000000004, 199.51250000000005, 199.54000000000002, 199.56750000000002, 199.59500000000003, 199.62250000000003, 199.65000000000003, 199.67750000000004, 199.70500000000004, 199.73250000000002, 199.76000000000005, 199.78750000000002, 199.81500000000003, 199.84250000000003, 199.87000000000003, 199.89750000000004, 199.92500000000004, 199.95250000000004, 199.98000000000002, 200.00750000000005, 200.03500000000003, 200.06250000000003, 200.09000000000003, 200.11750000000004, 200.14500000000004, 200.17250000000004, 200.20000000000005, 200.22750000000002, 200.25500000000002, 200.28250000000003, 200.31000000000003, 200.33750000000003, 200.36500000000004, 200.39250000000004, 200.42000000000002, 200.44750000000005, 200.47500000000002, 200.50250000000003, 200.53000000000003, 200.55750000000003, 200.58500000000004, 200.61250000000004, 200.64000000000004, 200.66750000000002, 200.69500000000005, 200.72250000000003, 200.75000000000003, 200.77750000000003, 200.80500000000004, 200.83250000000004, 200.86000000000004, 200.88750000000005, 200.91500000000002, 200.94250000000002, 200.97000000000003, 200.99750000000003, 201.02500000000003, 201.05250000000004, 201.08000000000004, 201.10750000000002, 201.13500000000005, 201.16250000000002, 201.19000000000003, 201.21750000000003, 201.24500000000003, 201.27250000000004, 201.30000000000004, 201.32750000000004, 201.35500000000002, 201.38250000000005, 201.41000000000003, 201.43750000000003, 201.46500000000003, 201.49250000000004, 201.52000000000004, 201.54750000000004, 201.57500000000005, 201.60250000000002, 201.63000000000002, 201.65750000000003, 201.68500000000003, 201.71250000000003, 201.74000000000004, 201.76750000000004, 201.79500000000002, 201.82250000000005, 201.85000000000002, 201.87750000000003, 201.90500000000003, 201.93250000000003, 201.96000000000004, 201.98750000000004, 202.01500000000004, 202.04250000000002, 202.07000000000005, 202.09750000000003, 202.12500000000003, 202.15250000000003, 202.18000000000004, 202.20750000000004, 202.23500000000004, 202.26250000000005, 202.29000000000002, 202.31750000000002, 202.34500000000003, 202.37250000000003, 202.40000000000003, 202.42750000000004, 202.45500000000004, 202.48250000000002, 202.51000000000005, 202.53750000000002, 202.56500000000003, 202.59250000000003, 202.62000000000003, 202.64750000000004, 202.67500000000004, 202.70250000000004, 202.73000000000002, 202.75750000000005, 202.78500000000003, 202.81250000000003, 202.84000000000003, 202.86750000000004, 202.89500000000004, 202.92250000000004, 202.95000000000005, 202.97750000000002, 203.00500000000002, 203.03250000000003, 203.06000000000003, 203.08750000000003, 203.11500000000004, 203.14250000000004, 203.17000000000002, |

| Description | Value |
| --- | --- |
| | 203.19750000000005, 203.22500000000002, 203.25250000000003, 203.28000000000003, 203.30750000000003, 203.33500000000004, 203.36250000000004, 203.39000000000004, 203.41750000000002, 203.44500000000005, 203.47250000000003, 203.50000000000003, 203.52750000000003, 203.55500000000004, 203.58250000000004, 203.61000000000004, 203.63750000000005, 203.66500000000002, 203.69250000000002, 203.72000000000003, 203.74750000000003, 203.77500000000003, 203.80250000000004, 203.83000000000004, 203.85750000000002, 203.88500000000005, 203.91250000000002, 203.94000000000003, 203.96750000000003, 203.99500000000003, 204.02250000000004, 204.05000000000004, 204.07750000000004, 204.10500000000002, 204.13250000000005, 204.16000000000003, 204.18750000000003, 204.21500000000003, 204.24250000000004, 204.27000000000004, 204.29750000000004, 204.32500000000005, 204.35250000000002, 204.38000000000002, 204.40750000000003, 204.43500000000003, 204.46250000000003, 204.49000000000004, 204.51750000000004, 204.54500000000002, 204.57250000000005, 204.60000000000002, 204.62750000000003, 204.65500000000003, 204.68250000000003, 204.71000000000004, 204.73750000000004, 204.76500000000004, 204.79250000000002, 204.82000000000005, 204.84750000000003, 204.87500000000003, 204.90250000000003, 204.93000000000004, 204.95750000000004, 204.98500000000004, 205.01250000000005, 205.04000000000002, 205.06750000000002, 205.09500000000003, 205.12250000000003, 205.15000000000003, 205.17750000000004, 205.20500000000004, 205.23250000000004, 205.26000000000005, 205.28750000000002, 205.31500000000003, 205.34250000000003, 205.37000000000003, 205.39750000000004, 205.42500000000004, 205.45250000000004, 205.48000000000002, 205.50750000000005, 205.53500000000003, 205.56250000000003, 205.59000000000003, 205.61750000000004, 205.64500000000004, 205.67250000000004, 205.70000000000005, 205.72750000000002, 205.75500000000002, 205.78250000000003, 205.81000000000003, 205.83750000000003, 205.86500000000004, 205.89250000000004, 205.92000000000004, 205.94750000000005, 205.97500000000002, 206.00250000000003, 206.03000000000003, 206.05750000000003, 206.08500000000004, 206.11250000000004, 206.14000000000004, 206.16750000000002, 206.19500000000005, 206.22250000000003, 206.25000000000003, 206.27750000000003, 206.30500000000004, 206.33250000000004, 206.36000000000004, 206.38750000000005, 206.41500000000002, 206.44250000000002, 206.47000000000003, 206.49750000000003, 206.52500000000003, 206.55250000000004, 206.58000000000004, 206.60750000000004, 206.63500000000005, 206.66250000000002, 206.69000000000003, 206.71750000000003, 206.74500000000003, 206.77250000000004, 206.80000000000004, 206.82750000000004, 206.85500000000002, 206.88250000000005, 206.91000000000003, 206.93750000000003, 206.96500000000003, 206.99250000000004, 207.02000000000004, 207.04750000000004, 207.07500000000005, 207.10250000000002, 207.13000000000002, |

| Description | Value |
| --- | --- |
| | 207.15750000000003, 207.18500000000003, 207.21250000000003, 207.24000000000004, 207.26750000000004, 207.29500000000004, 207.32250000000005, 207.35000000000002, 207.37750000000003, 207.40500000000003, 207.43250000000003, 207.46000000000004, 207.48750000000004, 207.51500000000004, 207.54250000000002, 207.57000000000005, 207.59750000000003, 207.62500000000003, 207.65250000000003, 207.68000000000004, 207.70750000000004, 207.73500000000004, 207.76250000000005, 207.79000000000002, 207.81750000000002, 207.84500000000003, 207.87250000000003, 207.90000000000003, 207.92750000000004, 207.95500000000004, 207.98250000000004, 208.01000000000005, 208.03750000000002, 208.06500000000003, 208.09250000000003, 208.12000000000003, 208.14750000000004, 208.17500000000004, 208.20250000000004, 208.23000000000002, 208.25750000000005, 208.28500000000003, 208.31250000000003, 208.34000000000003, 208.36750000000004, 208.39500000000004, 208.42250000000004, 208.45000000000005, 208.47750000000002, 208.50500000000002, 208.53250000000003, 208.56000000000003, 208.58750000000003, 208.61500000000004, 208.64250000000004, 208.67000000000004, 208.69750000000005, 208.72500000000002, 208.75250000000003, 208.78000000000003, 208.80750000000003, 208.83500000000004, 208.86250000000004, 208.89000000000004, 208.91750000000002, 208.94500000000005, 208.97250000000003, 209.00000000000003} |

### VALUES OF DEPENDENT VARIABLES

| Description | Value |
| --- | --- |
| Settings | User controlled |
| Method | Solution |
| Study | Study 3 (CV 21 to 30) |
| Settings | User controlled |
| Method | Solution |
| Study | Study 3 (CV 21 to 30) |

### MESH

| Feature | Value |
| --- | --- |
| Geometry 1 | mesh1 |

### PHYSICS AND VARIABLES SELECTION

| Physics interface | Discretization |
| --- | --- |
| Transport of Diluted Species in droplet (tds) | physics |
| Moving Mesh (ale) | physics |

### MESH SELECTION

| Geometry | Mesh |
| --- | --- |
| Geometry 1 (geom1) | mesh1 |

## 6.2 SOLVER CONFIGURATIONS

### 6.2.1 Solution 8

#### Compile Equations: Time Dependent (st1)

##### STUDY AND STEP

| Description | Value |
| --- | --- |
| Use study | Study 4 (CV 31 to 38) |
| Use study step | Time Dependent |

##### LOG

```
<---- Compile Equations: Time Dependent in Study 4 (CV 31 to 38)/Solution 8
      (sol8) ------------------------------------------------------------------
Started at May 31, 2023 11:06:43 AM.
Geometry shape function: Linear Lagrange
Running on Intel64 Family 6 Model 158 Stepping 10, GenuineIntel.
Using 1 socket with 6 cores in total on LAPTOP-2492RD8I.
Available memory: 32.27 GB.
Time: 2 s.
Physical memory: 2.43 GB
Virtual memory: 2.51 GB
Ended at May 31, 2023 11:06:45 AM.
----- Compile Equations: Time Dependent in Study 4 (CV 31 to 38)/Solution 8
      (sol8) ----------------------------------------------------------------->
```

#### Dependent Variables 1 (v1)

##### GENERAL

| Description | Value |
| --- | --- |
| Defined by study step | Time Dependent |

##### INITIAL VALUES OF VARIABLES SOLVED FOR

| Description | Value |
| --- | --- |
| Method | Solution |
| Solution | Remeshed Solution 3 |

##### RESIDUAL SCALING

| Description | Value |
| --- | --- |
| Method | Manual |

##### VALUES OF VARIABLES NOT SOLVED FOR

| Description | Value |
| --- | --- |

| Description | Value |
|---|---|
| Method | Solution |
| Solution | Remeshed Solution 3 |

### INITIAL VALUE CALCULATION CONSTANTS

| Constant name | Initial value source |
|---|---|
| t | range(30*t_cv,t_tot/nb/200,38*t_cv) |
| timestep | 0.044[s] |

### LOG

```
<---- Dependent Variables 1 in Study 4 (CV 31 to 38)/Solution 8 (sol8) ---------
Started at May 31, 2023 11:06:45 AM.
Initial values of variables solved for: Remeshed Solution 3 (sol7), t=165 s [Last].
Values of variables not solved for: Remeshed Solution 3 (sol7), t=165 s [Last].
Solution time: 0 s.
Physical memory: 2.44 GB
Virtual memory: 2.52 GB
Ended at May 31, 2023 11:06:45 AM.
----- Dependent Variables 1 in Study 4 (CV 31 to 38)/Solution 8 (sol8) -------->
```

**Concentration (comp1.cOx) (comp1_cOx)**

### GENERAL

| Description | Value |
|---|---|
| Field components | comp1.cOx |
| Internal variables | {comp1.uflux.cOx, comp1.dflux.cOx} |

**Concentration (comp1.cRed) (comp1_cRed)**

### GENERAL

| Description | Value |
|---|---|
| Field components | comp1.cRed |
| Internal variables | {comp1.uflux.cRed, comp1.dflux.cRed} |

**comp1.comp1.RgZg (comp1_comp1_RgZg)**

### GENERAL

| Description | Value |
|---|---|
| Field components | {Rg, Zg} |
| Solve for this field | Off |

**Spatial mesh displacement (comp1.spatial.disp) (comp1_spatial_disp)**

### GENERAL

| Description | Value |
|---|---|
| Field components | {comp1.spatial.u, comp1.spatial.w} |

SCALING

| Description | Value |
|---|---|
| Method | Manual |
| Scale | 1.3669045321455333E-7 |

### Time-Dependent Solver 1 (t1)

GENERAL

| Description | Value |
|---|---|
| Defined by study step | Time Dependent |
| Output times | {165.00000000000003, 165.02750000000003, 165.05500000000004, 165.08250000000004, 165.11000000000004, 165.13750000000002, 165.16500000000002, 165.19250000000002, 165.22000000000003, 165.24750000000003, 165.27500000000003, 165.30250000000004, 165.33000000000004, 165.35750000000002, 165.38500000000002, 165.41250000000002, 165.44000000000003, 165.46750000000003, 165.49500000000003, 165.52250000000004, 165.55000000000004, 165.57750000000001, 165.60500000000002, 165.63250000000002, 165.66000000000003, 165.68750000000003, 165.71500000000003, 165.74250000000004, 165.77000000000004, 165.79750000000004, 165.82500000000002, 165.85250000000002, 165.88000000000002, 165.90750000000003, 165.93500000000003, 165.96250000000003, 165.99000000000004, 166.01750000000004, 166.04500000000002, 166.07250000000002, 166.10000000000002, 166.12750000000003, 166.15500000000003, 166.18250000000003, 166.21000000000004, 166.23750000000004, 166.26500000000001, 166.29250000000002, 166.32000000000002, 166.34750000000003, 166.37500000000003, 166.40250000000003, 166.43000000000004, 166.45750000000004, 166.48500000000004, 166.51250000000002, 166.54000000000002, 166.56750000000002, 166.59500000000003, 166.62250000000003, 166.65000000000003, 166.67750000000004, 166.70500000000004, 166.73250000000002, 166.76000000000002, 166.78750000000002, 166.81500000000003, 166.84250000000003, 166.87000000000003, 166.89750000000004, 166.92500000000004, 166.95250000000001, 166.98000000000002, 167.00750000000002, 167.03500000000003, 167.06250000000003, 167.09000000000003, 167.11750000000004, 167.14500000000004, 167.17250000000004, 167.20000000000002, 167.22750000000002, 167.25500000000002, 167.28250000000003, 167.31000000000003, 167.33750000000003, 167.36500000000004, 167.39250000000004, 167.42000000000002, 167.44750000000002, 167.47500000000002, 167.50250000000003, 167.53000000000003, 167.55750000000003, 167.58500000000004, 167.61250000000004, 167.64000000000004, 167.66750000000002, 167.69500000000002, 167.72250000000003, 167.75000000000003, 167.77750000000003, 167.80500000000004, 167.83250000000004, 167.86000000000004, 167.88750000000002, 167.91500000000002, 167.94250000000002, 167.97000000000003, 167.99750000000003, 168.02500000000003, |

| Description | Value |
| --- | --- |
| | 168.05250000000004, 168.08000000000004, 168.10750000000002, 168.13500000000002, 168.16250000000002, 168.19000000000003, 168.21750000000003, 168.24500000000003, 168.27250000000004, 168.30000000000004, 168.32750000000004, 168.35500000000002, 168.38250000000002, 168.41000000000003, 168.43750000000003, 168.46500000000003, 168.49250000000004, 168.52000000000004, 168.54750000000004, 168.57500000000002, 168.60250000000002, 168.63000000000002, 168.65750000000003, 168.68500000000003, 168.71250000000003, 168.74000000000004, 168.76750000000004, 168.79500000000002, 168.82250000000002, 168.85000000000002, 168.87750000000003, 168.90500000000003, 168.93250000000003, 168.96000000000004, 168.98750000000004, 169.01500000000004, 169.04250000000002, 169.07000000000002, 169.09750000000003, 169.12500000000003, 169.15250000000003, 169.18000000000004, 169.20750000000004, 169.23500000000004, 169.26250000000002, 169.29000000000002, 169.31750000000002, 169.34500000000003, 169.37250000000003, 169.40000000000003, 169.42750000000004, 169.45500000000004, 169.48250000000002, 169.51000000000002, 169.53750000000002, 169.56500000000003, 169.59250000000003, 169.62000000000003, 169.64750000000004, 169.67500000000004, 169.70250000000004, 169.73000000000002, 169.75750000000002, 169.78500000000003, 169.81250000000003, 169.84000000000003, 169.86750000000004, 169.89500000000004, 169.92250000000004, 169.95000000000002, 169.97750000000002, 170.00500000000002, 170.03250000000003, 170.06000000000003, 170.08750000000003, 170.11500000000004, 170.14250000000004, 170.17000000000002, 170.19750000000002, 170.22500000000002, 170.25250000000003, 170.28000000000003, 170.30750000000003, 170.33500000000004, 170.36250000000004, 170.39000000000004, 170.41750000000002, 170.44500000000002, 170.47250000000003, 170.50000000000003, 170.52750000000003, 170.55500000000004, 170.58250000000004, 170.61000000000004, 170.63750000000002, 170.66500000000002, 170.69250000000002, 170.72000000000003, 170.74750000000003, 170.77500000000003, 170.80250000000004, 170.83000000000004, 170.85750000000002, 170.88500000000002, 170.91250000000002, 170.94000000000003, 170.96750000000003, 170.99500000000003, 171.02250000000004, 171.05000000000004, 171.07750000000004, 171.10500000000002, 171.13250000000002, 171.16000000000003, 171.18750000000003, 171.21500000000003, 171.24250000000004, 171.27000000000004, 171.29750000000004, 171.32500000000002, 171.35250000000002, 171.38000000000002, 171.40750000000003, 171.43500000000003, 171.46250000000003, 171.49000000000004, 171.51750000000004, 171.54500000000002, 171.57250000000002, 171.60000000000002, 171.62750000000003, 171.65500000000003, 171.68250000000003, 171.71000000000004, 171.73750000000004, 171.76500000000004, 171.79250000000002, 171.82000000000002, 171.84750000000003, 171.87500000000003, 171.90250000000003, 171.93000000000004, 171.95750000000004, 171.98500000000004, |

| Description | Value |
| --- | --- |
| | 172.01250000000002, 172.04000000000002, 172.06750000000002, 172.09500000000003, 172.12250000000003, 172.15000000000003, 172.17750000000004, 172.20500000000004, 172.23250000000002, 172.26000000000002, 172.28750000000002, 172.31500000000003, 172.34250000000003, 172.37000000000003, 172.39750000000004, 172.42500000000004, 172.45250000000004, 172.48000000000002, 172.50750000000002, 172.53500000000003, 172.56250000000003, 172.59000000000003, 172.61750000000004, 172.64500000000004, 172.67250000000004, 172.70000000000002, 172.72750000000002, 172.75500000000002, 172.78250000000003, 172.81000000000003, 172.83750000000003, 172.86500000000004, 172.89250000000004, 172.92000000000002, 172.94750000000002, 172.97500000000002, 173.00250000000003, 173.03000000000003, 173.05750000000003, 173.08500000000004, 173.11250000000004, 173.14000000000004, 173.16750000000002, 173.19500000000002, 173.22250000000003, 173.25000000000003, 173.27750000000003, 173.30500000000004, 173.33250000000004, 173.36000000000004, 173.38750000000002, 173.41500000000002, 173.44250000000002, 173.47000000000003, 173.49750000000003, 173.52500000000003, 173.55250000000004, 173.58000000000004, 173.60750000000002, 173.63500000000002, 173.66250000000002, 173.69000000000003, 173.71750000000003, 173.74500000000003, 173.77250000000004, 173.80000000000004, 173.82750000000004, 173.85500000000002, 173.88250000000002, 173.91000000000003, 173.93750000000003, 173.96500000000003, 173.99250000000004, 174.02000000000004, 174.04750000000004, 174.07500000000002, 174.10250000000002, 174.13000000000002, 174.15750000000003, 174.18500000000003, 174.21250000000003, 174.24000000000004, 174.26750000000004, 174.29500000000002, 174.32250000000002, 174.35000000000002, 174.37750000000003, 174.40500000000003, 174.43250000000003, 174.46000000000004, 174.48750000000004, 174.51500000000004, 174.54250000000002, 174.57000000000002, 174.59750000000003, 174.62500000000003, 174.65250000000003, 174.68000000000004, 174.70750000000004, 174.73500000000004, 174.76250000000002, 174.79000000000002, 174.81750000000002, 174.84500000000003, 174.87250000000003, 174.90000000000003, 174.92750000000004, 174.95500000000004, 174.98250000000002, 175.01000000000002, 175.03750000000002, 175.06500000000003, 175.09250000000003, 175.12000000000003, 175.14750000000004, 175.17500000000004, 175.20250000000004, 175.23000000000002, 175.25750000000002, 175.28500000000003, 175.31250000000003, 175.34000000000003, 175.36750000000004, 175.39500000000004, 175.42250000000004, 175.45000000000002, 175.47750000000002, 175.50500000000002, 175.53250000000003, 175.56000000000003, 175.58750000000003, 175.61500000000004, 175.64250000000004, 175.67000000000002, 175.69750000000002, 175.72500000000002, 175.75250000000003, 175.78000000000003, 175.80750000000003, 175.83500000000004, 175.86250000000004, 175.89000000000004, 175.91750000000002, 175.94500000000002, |

| Description | Value |
|---|---|
| | 175.97250000000003, 176.00000000000003, 176.02750000000003, 176.05500000000004, 176.08250000000004, 176.11000000000004, 176.13750000000002, 176.16500000000002, 176.19250000000002, 176.22000000000003, 176.24750000000003, 176.27500000000003, 176.30250000000004, 176.33000000000004, 176.35750000000002, 176.38500000000002, 176.41250000000002, 176.44000000000003, 176.46750000000003, 176.49500000000003, 176.52250000000004, 176.55000000000004, 176.57750000000004, 176.60500000000002, 176.63250000000002, 176.66000000000003, 176.68750000000003, 176.71500000000003, 176.74250000000004, 176.77000000000004, 176.79750000000004, 176.82500000000002, 176.85250000000002, 176.88000000000002, 176.90750000000003, 176.93500000000003, 176.96250000000003, 176.99000000000004, 177.01750000000004, 177.04500000000002, 177.07250000000002, 177.10000000000002, 177.12750000000003, 177.15500000000003, 177.18250000000003, 177.21000000000004, 177.23750000000004, 177.26500000000004, 177.29250000000002, 177.32000000000002, 177.34750000000003, 177.37500000000003, 177.40250000000003, 177.43000000000004, 177.45750000000004, 177.48500000000004, 177.51250000000002, 177.54000000000002, 177.56750000000002, 177.59500000000003, 177.62250000000003, 177.65000000000003, 177.67750000000004, 177.70500000000004, 177.73250000000002, 177.76000000000002, 177.78750000000002, 177.81500000000003, 177.84250000000003, 177.87000000000003, 177.89750000000004, 177.92500000000004, 177.95250000000004, 177.98000000000002, 178.00750000000002, 178.03500000000003, 178.06250000000003, 178.09000000000003, 178.11750000000004, 178.14500000000004, 178.17250000000004, 178.20000000000002, 178.22750000000002, 178.25500000000002, 178.28250000000003, 178.31000000000003, 178.33750000000003, 178.36500000000004, 178.39250000000004, 178.42000000000002, 178.44750000000002, 178.47500000000002, 178.50250000000003, 178.53000000000003, 178.55750000000003, 178.58500000000004, 178.61250000000004, 178.64000000000004, 178.66750000000002, 178.69500000000002, 178.72250000000003, 178.75000000000003, 178.77750000000003, 178.80500000000004, 178.83250000000004, 178.86000000000004, 178.88750000000002, 178.91500000000002, 178.94250000000002, 178.97000000000003, 178.99750000000003, 179.02500000000003, 179.05250000000004, 179.08000000000004, 179.10750000000002, 179.13500000000002, 179.16250000000002, 179.19000000000003, 179.21750000000003, 179.24500000000003, 179.27250000000004, 179.30000000000004, 179.32750000000004, 179.35500000000002, 179.38250000000002, 179.41000000000003, 179.43750000000003, 179.46500000000003, 179.49250000000004, 179.52000000000004, 179.54750000000004, 179.57500000000002, 179.60250000000002, 179.63000000000002, 179.65750000000003, 179.68500000000003, 179.71250000000003, 179.74000000000004, 179.76750000000004, 179.79500000000002, 179.82250000000002, 179.85000000000002, 179.87750000000003, 179.90500000000003, |

| Description | Value |
| --- | --- |
| | 179.93250000000003, 179.96000000000004, 179.98750000000004, 180.01500000000004, 180.04250000000002, 180.07000000000002, 180.09750000000003, 180.12500000000003, 180.15250000000003, 180.18000000000004, 180.20750000000004, 180.23500000000004, 180.26250000000005, 180.29000000000002, 180.31750000000002, 180.34500000000003, 180.37250000000003, 180.40000000000003, 180.42750000000004, 180.45500000000004, 180.48250000000002, 180.51000000000002, 180.53750000000002, 180.56500000000003, 180.59250000000003, 180.62000000000003, 180.64750000000004, 180.67500000000004, 180.70250000000004, 180.73000000000002, 180.75750000000002, 180.78500000000003, 180.81250000000003, 180.84000000000003, 180.86750000000004, 180.89500000000004, 180.92250000000004, 180.95000000000005, 180.97750000000002, 181.00500000000002, 181.03250000000003, 181.06000000000003, 181.08750000000003, 181.11500000000004, 181.14250000000004, 181.17000000000002, 181.19750000000002, 181.22500000000002, 181.25250000000003, 181.28000000000003, 181.30750000000003, 181.33500000000004, 181.36250000000004, 181.39000000000004, 181.41750000000002, 181.44500000000002, 181.47250000000003, 181.50000000000003, 181.52750000000003, 181.55500000000004, 181.58250000000004, 181.61000000000004, 181.63750000000005, 181.66500000000002, 181.69250000000002, 181.72000000000003, 181.74750000000003, 181.77500000000003, 181.80250000000004, 181.83000000000004, 181.85750000000002, 181.88500000000002, 181.91250000000002, 181.94000000000003, 181.96750000000003, 181.99500000000003, 182.02250000000004, 182.05000000000004, 182.07750000000004, 182.10500000000002, 182.13250000000002, 182.16000000000003, 182.18750000000003, 182.21500000000003, 182.24250000000004, 182.27000000000004, 182.29750000000004, 182.32500000000005, 182.35250000000002, 182.38000000000002, 182.40750000000003, 182.43500000000003, 182.46250000000003, 182.49000000000004, 182.51750000000004, 182.54500000000002, 182.57250000000002, 182.60000000000002, 182.62750000000003, 182.65500000000003, 182.68250000000003, 182.71000000000004, 182.73750000000004, 182.76500000000004, 182.79250000000002, 182.82000000000002, 182.84750000000003, 182.87500000000003, 182.90250000000003, 182.93000000000004, 182.95750000000004, 182.98500000000004, 183.01250000000005, 183.04000000000002, 183.06750000000002, 183.09500000000003, 183.12250000000003, 183.15000000000003, 183.17750000000004, 183.20500000000004, 183.23250000000002, 183.26000000000002, 183.28750000000002, 183.31500000000003, 183.34250000000003, 183.37000000000003, 183.39750000000004, 183.42500000000004, 183.45250000000004, 183.48000000000002, 183.50750000000002, 183.53500000000003, 183.56250000000003, 183.59000000000003, 183.61750000000004, 183.64500000000004, 183.67250000000004, 183.70000000000005, 183.72750000000002, 183.75500000000002, 183.78250000000003, 183.81000000000003, 183.83750000000003, 183.86500000000004, |

| Description | Value |
| --- | --- |
| | 183.89250000000004, 183.92000000000002, 183.94750000000002, 183.97500000000002, 184.00250000000003, 184.03000000000003, 184.05750000000003, 184.08500000000004, 184.11250000000004, 184.14000000000004, 184.16750000000002, 184.19500000000002, 184.22250000000003, 184.25000000000003, 184.27750000000003, 184.30500000000004, 184.33250000000004, 184.36000000000004, 184.38750000000005, 184.41500000000002, 184.44250000000002, 184.47000000000003, 184.49750000000003, 184.52500000000003, 184.55250000000004, 184.58000000000004, 184.60750000000002, 184.63500000000002, 184.66250000000002, 184.69000000000003, 184.71750000000003, 184.74500000000003, 184.77250000000004, 184.80000000000004, 184.82750000000004, 184.85500000000002, 184.88250000000002, 184.91000000000003, 184.93750000000003, 184.96500000000003, 184.99250000000004, 185.02000000000004, 185.04750000000004, 185.07500000000005, 185.10250000000002, 185.13000000000002, 185.15750000000003, 185.18500000000003, 185.21250000000003, 185.24000000000004, 185.26750000000004, 185.29500000000002, 185.32250000000002, 185.35000000000002, 185.37750000000003, 185.40500000000003, 185.43250000000003, 185.46000000000004, 185.48750000000004, 185.51500000000004, 185.54250000000002, 185.57000000000002, 185.59750000000003, 185.62500000000003, 185.65250000000003, 185.68000000000004, 185.70750000000004, 185.73500000000004, 185.76250000000005, 185.79000000000002, 185.81750000000002, 185.84500000000003, 185.87250000000003, 185.90000000000003, 185.92750000000004, 185.95500000000004, 185.98250000000002, 186.01000000000002, 186.03750000000002, 186.06500000000003, 186.09250000000003, 186.12000000000003, 186.14750000000004, 186.17500000000004, 186.20250000000004, 186.23000000000002, 186.25750000000002, 186.28500000000003, 186.31250000000003, 186.34000000000003, 186.36750000000004, 186.39500000000004, 186.42250000000004, 186.45000000000005, 186.47750000000002, 186.50500000000002, 186.53250000000003, 186.56000000000003, 186.58750000000003, 186.61500000000004, 186.64250000000004, 186.67000000000002, 186.69750000000002, 186.72500000000002, 186.75250000000003, 186.78000000000003, 186.80750000000003, 186.83500000000004, 186.86250000000004, 186.89000000000004, 186.91750000000002, 186.94500000000002, 186.97250000000003, 187.00000000000003, 187.02750000000003, 187.05500000000004, 187.08250000000004, 187.11000000000004, 187.13750000000005, 187.16500000000002, 187.19250000000002, 187.22000000000003, 187.24750000000003, 187.27500000000003, 187.30250000000004, 187.33000000000004, 187.35750000000002, 187.38500000000002, 187.41250000000002, 187.44000000000003, 187.46750000000003, 187.49500000000003, 187.52250000000004, 187.55000000000004, 187.57750000000004, 187.60500000000002, 187.63250000000002, 187.66000000000003, 187.68750000000003, 187.71500000000003, 187.74250000000004, 187.77000000000004, 187.79750000000004, 187.82500000000005, |

| Description | Value |
|---|---|
| | 187.85250000000002, 187.88000000000002, 187.90750000000003, 187.93500000000003, 187.96250000000003, 187.99000000000004, 188.01750000000004, 188.04500000000002, 188.07250000000002, 188.10000000000002, 188.12750000000003, 188.15500000000003, 188.18250000000003, 188.21000000000004, 188.23750000000004, 188.26500000000004, 188.29250000000002, 188.32000000000002, 188.34750000000003, 188.37500000000003, 188.40250000000003, 188.43000000000004, 188.45750000000004, 188.48500000000004, 188.51250000000005, 188.54000000000002, 188.56750000000002, 188.59500000000003, 188.62250000000003, 188.65000000000003, 188.67750000000004, 188.70500000000004, 188.73250000000002, 188.76000000000002, 188.78750000000002, 188.81500000000003, 188.84250000000003, 188.87000000000003, 188.89750000000004, 188.92500000000004, 188.95250000000004, 188.98000000000002, 189.00750000000002, 189.03500000000003, 189.06250000000003, 189.09000000000003, 189.11750000000004, 189.14500000000004, 189.17250000000004, 189.20000000000005, 189.22750000000002, 189.25500000000002, 189.28250000000003, 189.31000000000003, 189.33750000000003, 189.36500000000004, 189.39250000000004, 189.42000000000002, 189.44750000000002, 189.47500000000002, 189.50250000000003, 189.53000000000003, 189.55750000000003, 189.58500000000004, 189.61250000000004, 189.64000000000004, 189.66750000000002, 189.69500000000002, 189.72250000000003, 189.75000000000003, 189.77750000000003, 189.80500000000004, 189.83250000000004, 189.86000000000004, 189.88750000000005, 189.91500000000002, 189.94250000000002, 189.97000000000003, 189.99750000000003, 190.02500000000003, 190.05250000000004, 190.08000000000004, 190.10750000000002, 190.13500000000002, 190.16250000000002, 190.19000000000003, 190.21750000000003, 190.24500000000003, 190.27250000000004, 190.30000000000004, 190.32750000000004, 190.35500000000002, 190.38250000000002, 190.41000000000003, 190.43750000000003, 190.46500000000003, 190.49250000000004, 190.52000000000004, 190.54750000000004, 190.57500000000005, 190.60250000000002, 190.63000000000002, 190.65750000000003, 190.68500000000003, 190.71250000000003, 190.74000000000004, 190.76750000000004, 190.79500000000002, 190.82250000000005, 190.85000000000002, 190.87750000000003, 190.90500000000003, 190.93250000000003, 190.96000000000004, 190.98750000000004, 191.01500000000004, 191.04250000000002, 191.07000000000002, 191.09750000000003, 191.12500000000003, 191.15250000000003, 191.18000000000004, 191.20750000000004, 191.23500000000004, 191.26250000000005, 191.29000000000002, 191.31750000000002, 191.34500000000003, 191.37250000000003, 191.40000000000003, 191.42750000000004, 191.45500000000004, 191.48250000000002, 191.51000000000005, 191.53750000000002, 191.56500000000003, 191.59250000000003, 191.62000000000003, 191.64750000000004, 191.67500000000004, 191.70250000000004, 191.73000000000002, 191.75750000000002, 191.78500000000003, |

| Description | Value |
| --- | --- |
| | 191.81250000000003, 191.84000000000003, 191.86750000000004, 191.89500000000004, 191.92250000000004, 191.95000000000005, 191.97750000000002, 192.00500000000002, 192.03250000000003, 192.06000000000003, 192.08750000000003, 192.11500000000004, 192.14250000000004, 192.17000000000004, 192.19750000000005, 192.22500000000002, 192.25250000000003, 192.28000000000003, 192.30750000000003, 192.33500000000004, 192.36250000000004, 192.39000000000004, 192.41750000000002, 192.44500000000002, 192.47250000000003, 192.50000000000003, 192.52750000000003, 192.55500000000004, 192.58250000000004, 192.61000000000004, 192.63750000000005, 192.66500000000002, 192.69250000000002, 192.72000000000003, 192.74750000000003, 192.77500000000003, 192.80250000000004, 192.83000000000004, 192.85750000000004, 192.88500000000005, 192.91250000000002, 192.94000000000003, 192.96750000000003, 192.99500000000003, 193.02250000000004, 193.05000000000004, 193.07750000000004, 193.10500000000002, 193.13250000000002, 193.16000000000003, 193.18750000000003, 193.21500000000003, 193.24250000000004, 193.27000000000004, 193.29750000000004, 193.32500000000005, 193.35250000000002, 193.38000000000002, 193.40750000000003, 193.43500000000003, 193.46250000000003, 193.49000000000004, 193.51750000000004, 193.54500000000004, 193.57250000000005, 193.60000000000002, 193.62750000000003, 193.65500000000003, 193.68250000000003, 193.71000000000004, 193.73750000000004, 193.76500000000004, 193.79250000000002, 193.82000000000002, 193.84750000000003, 193.87500000000003, 193.90250000000003, 193.93000000000004, 193.95750000000004, 193.98500000000004, 194.01250000000005, 194.04000000000002, 194.06750000000002, 194.09500000000003, 194.12250000000003, 194.15000000000003, 194.17750000000004, 194.20500000000004, 194.23250000000004, 194.26000000000005, 194.28750000000002, 194.31500000000003, 194.34250000000003, 194.37000000000003, 194.39750000000004, 194.42500000000004, 194.45250000000004, 194.48000000000002, 194.50750000000002, 194.53500000000003, 194.56250000000003, 194.59000000000003, 194.61750000000004, 194.64500000000004, 194.67250000000004, 194.70000000000005, 194.72750000000002, 194.75500000000002, 194.78250000000003, 194.81000000000003, 194.83750000000003, 194.86500000000004, 194.89250000000004, 194.92000000000004, 194.94750000000005, 194.97500000000002, 195.00250000000003, 195.03000000000003, 195.05750000000003, 195.08500000000004, 195.11250000000004, 195.14000000000004, 195.16750000000002, 195.19500000000002, 195.22250000000003, 195.25000000000003, 195.27750000000003, 195.30500000000004, 195.33250000000004, 195.36000000000004, 195.38750000000005, 195.41500000000002, 195.44250000000002, 195.47000000000003, 195.49750000000003, 195.52500000000003, 195.55250000000004, 195.58000000000004, 195.60750000000004, 195.63500000000005, 195.66250000000002, 195.69000000000003, 195.71750000000003, 195.74500000000003, |

| Description | Value |
| --- | --- |
| | 195.77250000000004, 195.80000000000004, 195.82750000000004, 195.85500000000002, 195.88250000000002, 195.91000000000003, 195.93750000000003, 195.96500000000003, 195.99250000000004, 196.02000000000004, 196.04750000000004, 196.07500000000005, 196.10250000000002, 196.13000000000002, 196.15750000000003, 196.18500000000003, 196.21250000000003, 196.24000000000004, 196.26750000000004, 196.29500000000004, 196.32250000000005, 196.35000000000002, 196.37750000000003, 196.40500000000003, 196.43250000000003, 196.46000000000004, 196.48750000000004, 196.51500000000004, 196.54250000000002, 196.57000000000002, 196.59750000000003, 196.62500000000003, 196.65250000000003, 196.68000000000004, 196.70750000000004, 196.73500000000004, 196.76250000000005, 196.79000000000002, 196.81750000000002, 196.84500000000003, 196.87250000000003, 196.90000000000003, 196.92750000000004, 196.95500000000004, 196.98250000000004, 197.01000000000005, 197.03750000000002, 197.06500000000003, 197.09250000000003, 197.12000000000003, 197.14750000000004, 197.17500000000004, 197.20250000000004, 197.23000000000002, 197.25750000000005, 197.28500000000003, 197.31250000000003, 197.34000000000003, 197.36750000000004, 197.39500000000004, 197.42250000000004, 197.45000000000005, 197.47750000000002, 197.50500000000002, 197.53250000000003, 197.56000000000003, 197.58750000000003, 197.61500000000004, 197.64250000000004, 197.67000000000002, 197.69750000000005, 197.72500000000002, 197.75250000000003, 197.78000000000003, 197.80750000000003, 197.83500000000004, 197.86250000000004, 197.89000000000004, 197.91750000000002, 197.94500000000005, 197.97250000000003, 198.00000000000003, 198.02750000000003, 198.05500000000004, 198.08250000000004, 198.11000000000004, 198.13750000000005, 198.16500000000002, 198.19250000000002, 198.22000000000003, 198.24750000000003, 198.27500000000003, 198.30250000000004, 198.33000000000004, 198.35750000000002, 198.38500000000005, 198.41250000000002, 198.44000000000003, 198.46750000000003, 198.49500000000003, 198.52250000000004, 198.55000000000004, 198.57750000000004, 198.60500000000002, 198.63250000000005, 198.66000000000003, 198.68750000000003, 198.71500000000003, 198.74250000000004, 198.77000000000004, 198.79750000000004, 198.82500000000005, 198.85250000000002, 198.88000000000002, 198.90750000000003, 198.93500000000003, 198.96250000000003, 198.99000000000004, 199.01750000000004, 199.04500000000002, 199.07250000000005, 199.10000000000002, 199.12750000000003, 199.15500000000003, 199.18250000000003, 199.21000000000004, 199.23750000000004, 199.26500000000004, 199.29250000000002, 199.32000000000005, 199.34750000000003, 199.37500000000003, 199.40250000000003, 199.43000000000004, 199.45750000000004, 199.48500000000004, 199.51250000000005, 199.54000000000002, 199.56750000000002, 199.59500000000003, 199.62250000000003, 199.65000000000003, 199.67750000000004, 199.70500000000004, |

| Description | Value |
| --- | --- |
| | 199.73250000000002, 199.76000000000005, 199.78750000000002, 199.81500000000003, 199.84250000000003, 199.87000000000003, 199.89750000000004, 199.92500000000004, 199.95250000000004, 199.98000000000002, 200.00750000000005, 200.03500000000003, 200.06250000000003, 200.09000000000003, 200.11750000000004, 200.14500000000004, 200.17250000000004, 200.20000000000005, 200.22750000000002, 200.25500000000002, 200.28250000000003, 200.31000000000003, 200.33750000000003, 200.36500000000004, 200.39250000000004, 200.42000000000002, 200.44750000000005, 200.47500000000002, 200.50250000000003, 200.53000000000003, 200.55750000000003, 200.58500000000004, 200.61250000000004, 200.64000000000004, 200.66750000000002, 200.69500000000005, 200.72250000000003, 200.75000000000003, 200.77750000000003, 200.80500000000004, 200.83250000000004, 200.86000000000004, 200.88750000000005, 200.91500000000002, 200.94250000000002, 200.97000000000003, 200.99750000000003, 201.02500000000003, 201.05250000000004, 201.08000000000004, 201.10750000000002, 201.13500000000005, 201.16250000000002, 201.19000000000003, 201.21750000000003, 201.24500000000003, 201.27250000000004, 201.30000000000004, 201.32750000000004, 201.35500000000002, 201.38250000000005, 201.41000000000003, 201.43750000000003, 201.46500000000003, 201.49250000000004, 201.52000000000004, 201.54750000000004, 201.57500000000005, 201.60250000000002, 201.63000000000002, 201.65750000000003, 201.68500000000003, 201.71250000000003, 201.74000000000004, 201.76750000000004, 201.79500000000002, 201.82250000000005, 201.85000000000002, 201.87750000000003, 201.90500000000003, 201.93250000000003, 201.96000000000004, 201.98750000000004, 202.01500000000004, 202.04250000000002, 202.07000000000005, 202.09750000000003, 202.12500000000003, 202.15250000000003, 202.18000000000004, 202.20750000000004, 202.23500000000004, 202.26250000000005, 202.29000000000002, 202.31750000000002, 202.34500000000003, 202.37250000000003, 202.40000000000003, 202.42750000000004, 202.45500000000004, 202.48250000000002, 202.51000000000005, 202.53750000000002, 202.56500000000003, 202.59250000000003, 202.62000000000003, 202.64750000000004, 202.67500000000004, 202.70250000000004, 202.73000000000002, 202.75750000000005, 202.78500000000003, 202.81250000000003, 202.84000000000003, 202.86750000000004, 202.89500000000004, 202.92250000000004, 202.95000000000005, 202.97750000000002, 203.00500000000002, 203.03250000000003, 203.06000000000003, 203.08750000000003, 203.11500000000004, 203.14250000000004, 203.17000000000002, 203.19750000000005, 203.22500000000002, 203.25250000000003, 203.28000000000003, 203.30750000000003, 203.33500000000004, 203.36250000000004, 203.39000000000004, 203.41750000000002, 203.44500000000005, 203.47250000000003, 203.50000000000003, 203.52750000000003, 203.55500000000004, 203.58250000000004, 203.61000000000004, 203.63750000000005, 203.66500000000002, |

| Description | Value |
| --- | --- |
| | 203.69250000000002, 203.72000000000003, 203.74750000000003, 203.77500000000003, 203.80250000000004, 203.83000000000004, 203.85750000000002, 203.88500000000005, 203.91250000000002, 203.94000000000003, 203.96750000000003, 203.99500000000003, 204.02250000000004, 204.05000000000004, 204.07750000000004, 204.10500000000002, 204.13250000000005, 204.16000000000003, 204.18750000000003, 204.21500000000003, 204.24250000000004, 204.27000000000004, 204.29750000000004, 204.32500000000005, 204.35250000000002, 204.38000000000002, 204.40750000000003, 204.43500000000003, 204.46250000000003, 204.49000000000004, 204.51750000000004, 204.54500000000002, 204.57250000000005, 204.60000000000002, 204.62750000000003, 204.65500000000003, 204.68250000000003, 204.71000000000004, 204.73750000000004, 204.76500000000004, 204.79250000000002, 204.82000000000005, 204.84750000000003, 204.87500000000003, 204.90250000000003, 204.93000000000004, 204.95750000000004, 204.98500000000004, 205.01250000000005, 205.04000000000002, 205.06750000000002, 205.09500000000003, 205.12250000000003, 205.15000000000003, 205.17750000000004, 205.20500000000004, 205.23250000000004, 205.26000000000005, 205.28750000000002, 205.31500000000003, 205.34250000000003, 205.37000000000003, 205.39750000000004, 205.42500000000004, 205.45250000000004, 205.48000000000002, 205.50750000000005, 205.53500000000003, 205.56250000000003, 205.59000000000003, 205.61750000000004, 205.64500000000004, 205.67250000000004, 205.70000000000005, 205.72750000000002, 205.75500000000002, 205.78250000000003, 205.81000000000003, 205.83750000000003, 205.86500000000004, 205.89250000000004, 205.92000000000004, 205.94750000000005, 205.97500000000002, 206.00250000000003, 206.03000000000003, 206.05750000000003, 206.08500000000004, 206.11250000000004, 206.14000000000004, 206.16750000000002, 206.19500000000005, 206.22250000000003, 206.25000000000003, 206.27750000000003, 206.30500000000004, 206.33250000000004, 206.36000000000004, 206.38750000000005, 206.41500000000002, 206.44250000000002, 206.47000000000003, 206.49750000000003, 206.52500000000003, 206.55250000000004, 206.58000000000004, 206.60750000000004, 206.63500000000005, 206.66250000000002, 206.69000000000003, 206.71750000000003, 206.74500000000003, 206.77250000000004, 206.80000000000004, 206.82750000000004, 206.85500000000002, 206.88250000000005, 206.91000000000003, 206.93750000000003, 206.96500000000003, 206.99250000000004, 207.02000000000004, 207.04750000000004, 207.07500000000005, 207.10250000000002, 207.13000000000002, 207.15750000000003, 207.18500000000003, 207.21250000000003, 207.24000000000004, 207.26750000000004, 207.29500000000004, 207.32250000000005, 207.35000000000002, 207.37750000000003, 207.40500000000003, 207.43250000000003, 207.46000000000004, 207.48750000000004, 207.51500000000004, 207.54250000000002, 207.57000000000005, 207.59750000000003, 207.62500000000003, |

| Description | Value |
| --- | --- |
| | 207.65250000000003, 207.68000000000004, 207.70750000000004, 207.73500000000004, 207.76250000000005, 207.79000000000002, 207.81750000000002, 207.84500000000003, 207.87250000000003, 207.90000000000003, 207.92750000000004, 207.95500000000004, 207.98250000000004, 208.01000000000005, 208.03750000000002, 208.06500000000003, 208.09250000000003, 208.12000000000003, 208.14750000000004, 208.17500000000004, 208.20250000000004, 208.23000000000002, 208.25750000000005, 208.28500000000003, 208.31250000000003, 208.34000000000003, 208.36750000000004, 208.39500000000004, 208.42250000000004, 208.45000000000005, 208.47750000000002, 208.50500000000002, 208.53250000000003, 208.56000000000003, 208.58750000000003, 208.61500000000004, 208.64250000000004, 208.67000000000004, 208.69750000000005, 208.72500000000002, 208.75250000000003, 208.78000000000003, 208.80750000000003, 208.83500000000004, 208.86250000000004, 208.89000000000004, 208.91750000000002, 208.94500000000005, 208.97250000000003, 209.00000000000003} |
| Relative tolerance | 0.005 |

### TIME STEPPING

| Description | Value |
| --- | --- |
| Maximum BDF order | 2 |
| Nonlinear controller | On |

### LOG

| | | | | | | | | | | |
|---|---|---|---|---|---|---|---|---|---|---|
| 368 | 196.76 | 0.051309 | 58 | 30 | 58 | 2 | 2 | 0 | 6.8e-14 | 5.1e-15 |
| - | 196.79 | - out | | | | | | | | |
| 369 | 196.81 | 0.051309 | 60 | 31 | 60 | 2 | 2 | 0 | 1.5e-14 | 5.7e-15 |
| - | 196.82 | - out | | | | | | | | |
| - | 196.85 | - out | | | | | | | | |
| 370 | 196.87 | 0.051309 | 62 | 32 | 62 | 2 | 2 | 0 | 8.9e-14 | 5.3e-15 |
| - | 196.87 | - out | | | | | | | | |
| - | 196.9 | - out | | | | | | | | |
| - | 196.93 | - out | | | | | | | | |
| - | 196.96 | - out | | | | | | | | |
| 371 | 196.97 | 0.10262 | 64 | 33 | 64 | 2 | 2 | 0 | 7.5e-14 | 3.9e-15 |
| - | 196.98 | - out | | | | | | | | |
| - | 197.01 | - out | | | | | | | | |
| - | 197.04 | - out | | | | | | | | |
| 372 | 197.05 | 0.08619 | 66 | 34 | 66 | 2 | 2 | 0 | 1.5e-13 | 4.6e-15 |
| - | 197.07 | - out | | | | | | | | |
| - | 197.09 | - out | | | | | | | | |
| - | 197.12 | - out | | | | | | | | |
| 373 | 197.13 | 0.077571 | 68 | 35 | 68 | 2 | 2 | 0 | 2.7e-13 | 3.6e-15 |
| - | 197.15 | - out | | | | | | | | |
| - | 197.18 | - out | | | | | | | | |
| - | 197.2 | - out | | | | | | | | |
| 374 | 197.21 | 0.077571 | 70 | 36 | 70 | 2 | 2 | 0 | 8.8e-14 | 4.4e-15 |
| - | 197.23 | - out | | | | | | | | |
| - | 197.26 | - out | | | | | | | | |
| - | 197.29 | - out | | | | | | | | |
| 375 | 197.29 | 0.077571 | 72 | 37 | 72 | 2 | 2 | 0 | 4.1e-13 | 2.9e-15 |
| - | 197.31 | - out | | | | | | | | |
| - | 197.34 | - out | | | | | | | | |
| 376 | 197.36 | 0.077571 | 74 | 38 | 74 | 2 | 2 | 0 | 5.1e-13 | 4.9e-15 |
| - | 197.37 | - out | | | | | | | | |
| - | 197.4 | - out | | | | | | | | |
| - | 197.42 | - out | | | | | | | | |
| 377 | 197.44 | 0.077571 | 76 | 39 | 76 | 2 | 2 | 0 | 2.1e-13 | 3.3e-15 |
| - | 197.45 | - out | | | | | | | | |
| - | 197.48 | - out | | | | | | | | |
| - | 197.51 | - out | | | | | | | | |
| 378 | 197.52 | 0.077571 | 78 | 40 | 78 | 2 | 2 | 0 | 6.3e-13 | 5.4e-15 |
| - | 197.53 | - out | | | | | | | | |
| - | 197.56 | - out | | | | | | | | |
| - | 197.59 | - out | | | | | | | | |
| - | 197.62 | - out | | | | | | | | |
| - | 197.64 | - out | | | | | | | | |
| - | 197.67 | - out | | | | | | | | |
| 379 | 197.68 | 0.15514 | 80 | 41 | 80 | 2 | 2 | 0 | 1.7e-12 | 4.5e-15 |
| - | 197.7 | - out | | | | | | | | |
| - | 197.73 | - out | | | | | | | | |
| - | 197.75 | - out | | | | | | | | |
| - | 197.78 | - out | | | | | | | | |
| - | 197.81 | - out | | | | | | | | |
| 380 | 197.83 | 0.15514 | 82 | 42 | 82 | 2 | 2 | 0 | 1.3e-12 | 3e-15 |
| - | 197.84 | - out | | | | | | | | |
| - | 197.86 | - out | | | | | | | | |
| - | 197.89 | - out | | | | | | | | |
| - | 197.92 | - out | | | | | | | | |
| - | 197.95 | - out | | | | | | | | |

| | | | | | | | | | | |
|---|---|---|---|---|---|---|---|---|---|---|
| - | 197.97 | - | out | | | | | | | |
| 381 | 197.99 | 0.15514 | 84 | 43 | 84 | 2 | 2 | 0 | 1.5e-12 | 3e-15 |
| - | 198 | - | out | | | | | | | |
| - | 198.03 | - | out | | | | | | | |
| - | 198.06 | - | out | | | | | | | |
| - | 198.08 | - | out | | | | | | | |
| - | 198.11 | - | out | | | | | | | |
| - | 198.14 | - | out | | | | | | | |
| 382 | 198.14 | 0.15514 | 86 | 44 | 86 | 2 | 2 | 0 | 9.6e-14 | 4.2e-15 |
| - | 198.17 | - | out | | | | | | | |
| - | 198.19 | - | out | | | | | | | |
| - | 198.22 | - | out | | | | | | | |
| - | 198.25 | - | out | | | | | | | |
| - | 198.28 | - | out | | | | | | | |
| - | 198.3 | - | out | | | | | | | |
| - | 198.33 | - | out | | | | | | | |
| - | 198.36 | - | out | | | | | | | |
| - | 198.39 | - | out | | | | | | | |
| - | 198.41 | - | out | | | | | | | |
| - | 198.44 | - | out | | | | | | | |
| 383 | 198.45 | 0.31029 | 88 | 45 | 88 | 2 | 2 | 0 | 3.1e-13 | 4.4e-15 |
| - | 198.47 | - | out | | | | | | | |
| - | 198.5 | - | out | | | | | | | |
| - | 198.52 | - | out | | | | | | | |
| - | 198.55 | - | out | | | | | | | |
| 384 | 198.57 | 0.12036 | 92 | 47 | 92 | 2 | 3 | 0 | 6.4e-13 | 4.6e-15 |
| - | 198.58 | - | out | | | | | | | |
| - | 198.61 | - | out | | | | | | | |
| - | 198.63 | - | out | | | | | | | |
| - | 198.66 | - | out | | | | | | | |
| - | 198.69 | - | out | | | | | | | |
| 385 | 198.69 | 0.12036 | 94 | 48 | 94 | 2 | 3 | 0 | 9.5e-14 | 4.4e-15 |
| - | 198.72 | - | out | | | | | | | |
| - | 198.74 | - | out | | | | | | | |
| 386 | 198.76 | 0.066976 | 98 | 50 | 98 | 2 | 4 | 0 | 2.4e-13 | 5.2e-15 |
| - | 198.77 | - | out | | | | | | | |
| - | 198.8 | - | out | | | | | | | |
| - | 198.83 | - | out | | | | | | | |
| 387 | 198.83 | 0.066976 | 100 | 51 | 100 | 2 | 4 | 0 | 2e-13 | 3.8e-15 |
| - | 198.85 | - | out | | | | | | | |
| - | 198.88 | - | out | | | | | | | |
| 388 | 198.89 | 0.060279 | 102 | 52 | 102 | 2 | 4 | 0 | 1.2e-13 | 5e-15 |
| - | 198.91 | - | out | | | | | | | |
| - | 198.94 | - | out | | | | | | | |
| 389 | 198.95 | 0.060279 | 104 | 53 | 104 | 2 | 4 | 0 | 1.9e-13 | 5.5e-15 |
| - | 198.96 | - | out | | | | | | | |
| - | 198.99 | - | out | | | | | | | |
| 390 | 199.01 | 0.060279 | 106 | 54 | 106 | 2 | 4 | 0 | 6.8e-14 | 6e-15 |
| - | 199.02 | - | out | | | | | | | |
| - | 199.05 | - | out | | | | | | | |
| 391 | 199.07 | 0.060279 | 108 | 55 | 108 | 2 | 4 | 0 | 2.4e-14 | 8.2e-15 |
| - | 199.07 | - | out | | | | | | | |
| - | 199.1 | - | out | | | | | | | |
| 392 | 199.13 | 0.060279 | 110 | 56 | 110 | 2 | 4 | 0 | 9.4e-15 | 2.4e-14 |
| - | 199.13 | - | out | | | | | | | |
| - | 199.16 | - | out | | | | | | | |

| | | | | | | | | | | |
|---|---|---|---|---|---|---|---|---|---|---|
| - | 199.18 | - out | | | | | | | | |
| 393 | 199.19 | 0.060279 | 112 | 57 | 112 | 2 | 4 | 0 | 3.6e-14 | 1.2e-14 |
| - | 199.21 | - out | | | | | | | | |
| - | 199.24 | - out | | | | | | | | |
| 394 | 199.25 | 0.060279 | 114 | 58 | 114 | 2 | 4 | 0 | 3.5e-14 | 8.6e-15 |
| - | 199.27 | - out | | | | | | | | |
| - | 199.29 | - out | | | | | | | | |
| 395 | 199.31 | 0.060279 | 116 | 59 | 116 | 2 | 4 | 0 | 1.8e-14 | 8.8e-15 |
| - | 199.32 | - out | | | | | | | | |
| - | 199.35 | - out | | | | | | | | |
| 396 | 199.37 | 0.060279 | 118 | 60 | 118 | 2 | 4 | 0 | 1.2e-14 | 7.8e-15 |
| - | 199.38 | - out | | | | | | | | |
| - | 199.4 | - out | | | | | | | | |
| 397 | 199.43 | 0.060279 | 120 | 61 | 120 | 2 | 4 | 0 | 2.3e-14 | 7.4e-15 |
| - | 199.43 | - out | | | | | | | | |
| - | 199.46 | - out | | | | | | | | |
| - | 199.49 | - out | | | | | | | | |
| 398 | 199.49 | 0.060279 | 122 | 62 | 122 | 2 | 4 | 0 | 2.3e-13 | 7.2e-15 |
| - | 199.51 | - out | | | | | | | | |
| - | 199.54 | - out | | | | | | | | |
| 399 | 199.55 | 0.060279 | 124 | 63 | 124 | 2 | 4 | 0 | 7.2e-14 | 6.1e-15 |
| - | 199.57 | - out | | | | | | | | |
| - | 199.6 | - out | | | | | | | | |
| 400 | 199.61 | 0.060279 | 126 | 64 | 126 | 2 | 4 | 0 | 5.1e-14 | 5.5e-15 |
| - | 199.62 | - out | | | | | | | | |
| - | 199.65 | - out | | | | | | | | |
| 401 | 199.67 | 0.060279 | 128 | 65 | 128 | 2 | 4 | 0 | 6.9e-14 | 5.2e-15 |
| - | 199.68 | - out | | | | | | | | |
| - | 199.71 | - out | | | | | | | | |
| 402 | 199.73 | 0.060279 | 130 | 66 | 130 | 2 | 4 | 0 | 1.6e-14 | 9.1e-15 |
| - | 199.73 | - out | | | | | | | | |
| - | 199.76 | - out | | | | | | | | |
| - | 199.79 | - out | | | | | | | | |
| 403 | 199.79 | 0.060279 | 132 | 67 | 132 | 2 | 4 | 0 | 3.4e-14 | 3.6e-15 |
| - | 199.82 | - out | | | | | | | | |
| - | 199.84 | - out | | | | | | | | |
| 404 | 199.85 | 0.060279 | 134 | 68 | 134 | 2 | 4 | 0 | 1.2e-13 | 3.6e-15 |
| - | 199.87 | - out | | | | | | | | |
| - | 199.9 | - out | | | | | | | | |
| 405 | 199.91 | 0.060279 | 136 | 69 | 136 | 2 | 4 | 0 | 3.1e-13 | 3.3e-15 |
| - | 199.93 | - out | | | | | | | | |
| - | 199.95 | - out | | | | | | | | |
| 406 | 199.97 | 0.060279 | 138 | 70 | 138 | 2 | 4 | 0 | 2.9e-13 | 3.3e-15 |
| - | 199.98 | - out | | | | | | | | |
| - | 200.01 | - out | | | | | | | | |
| 407 | 200.03 | 0.060279 | 140 | 71 | 140 | 2 | 4 | 0 | 3.7e-13 | 3.4e-15 |
| - | 200.04 | - out | | | | | | | | |
| - | 200.06 | - out | | | | | | | | |
| - | 200.09 | - out | | | | | | | | |
| 408 | 200.09 | 0.060279 | 142 | 72 | 142 | 2 | 4 | 0 | 2.8e-13 | 4.4e-15 |
| - | 200.12 | - out | | | | | | | | |
| - | 200.15 | - out | | | | | | | | |
| 409 | 200.15 | 0.060279 | 144 | 73 | 144 | 2 | 4 | 0 | 1.4e-12 | 5.4e-15 |
| - | 200.17 | - out | | | | | | | | |
| - | 200.2 | - out | | | | | | | | |
| 410 | 200.21 | 0.060279 | 146 | 74 | 146 | 2 | 4 | 0 | 3.5e-13 | 4e-15 |

```
  -      200.23          - out
  -      200.26          - out
 411     200.27   0.060279      148   75  148     2     4      0  7.2e-13  5.3e-15
  -      200.28          - out
  -      200.31          - out
  -      200.34          - out
  -      200.37          - out
  -      200.39          - out
 412     200.39    0.12056      150   76  150     2     4      0  5.1e-13  4.5e-15
  -      200.42          - out
  -      200.45          - out
  -      200.48          - out
  -       200.5          - out
 413     200.51    0.12056      152   77  152     2     4      0  1.8e-12  4.6e-15
  -      200.53          - out
  -      200.56          - out
  -      200.59          - out
  -      200.61          - out
 414     200.63    0.12056      154   78  154     2     4      0  5.2e-13  3.9e-15
  -      200.64          - out
  -      200.67          - out
  -       200.7          - out
  -      200.72          - out
  -      200.75          - out
 415     200.75    0.12056      156   79  156     2     4      0    2e-12  6.5e-15
  -      200.78          - out
  -      200.81          - out
  -      200.83          - out
  -      200.86          - out
 416     200.88    0.12056      158   80  158     2     4      0  2.2e-13  7.1e-15
  -      200.89          - out
  -      200.92          - out
  -      200.94          - out
  -      200.97          - out
 417        201    0.12056      160   81  160     2     4      0  4.5e-14  4.7e-15
  -         201          - out
  -      201.03          - out
  -      201.05          - out
  -      201.08          - out
  -      201.11          - out
 418     201.12    0.12056      162   82  162     2     4      0  2.4e-13  4.3e-15
  -      201.14          - out
  -      201.16          - out
  -      201.19          - out
  -      201.22          - out
 419     201.24    0.12056      164   83  164     2     4      0  2.9e-13  3.8e-15
  -      201.24          - out
 420     201.36    0.12056      166   84  166     2     4      0  3.3e-13  2.9e-15
Number of vertex elements: 3
Number of boundary elements: 112
Number of vertex elements: 3
Number of boundary elements: 112
Number of elements: 534
Minimum element quality: 0.5661
Number of vertex elements: 4
Number of boundary elements: 162
```

```
Number of vertex elements: 5
Number of boundary elements: 212
Number of vertex elements: 6
Number of boundary elements: 266
Minimum element quality: 0.09645
Geometry shape function: Linear Lagrange
Time interval 5
Time-dependent solver (BDF)
Number of degrees of freedom solved for: 11758 (plus 7124 internal DOFs).
Nonsymmetric matrix found.
Scales for dependent variables:
Concentration (comp1.cOx): 4.2
Concentration (comp1.cRed): 2.8
Spatial mesh displacement (comp1.spatial.disp): 1.4e-07
Step        Time    Stepsize      Res  Jac  Sol Order Tfail NLfail   LinErr   LinRes
   -      201.24           - out
   -      201.25           - out
 421      201.26      0.0275        2    2    2     1     0      0  1.3e-14  2.7e-15
   -      201.27           - out
 422      201.29      0.0275        4    3    4     1     0      0  1.3e-13  3.2e-15
   -       201.3           - out
   -      201.33           - out
 423      201.35       0.055        6    4    6     2     0      0  1.7e-13  3.1e-15
   -      201.36           - out
   -      201.38           - out
   -      201.41           - out
   -      201.44           - out
 424      201.46        0.11        8    5    8     2     0      0  9.7e-14  3.2e-15
   -      201.47           - out
   -      201.49           - out
   -      201.52           - out
 425      201.55    0.090001       10    6   10     2     0      0  1.7e-13  3.6e-15
   -      201.55           - out
   -      201.58           - out
   -       201.6           - out
 426      201.62    0.077339       12    7   12     2     0      0  3.8e-14  3.5e-15
   -      201.63           - out
   -      201.66           - out
   -      201.69           - out
 427      201.69    0.064268       14    8   14     2     0      0  8.9e-14    4e-15
   -      201.71           - out
   -      201.74           - out
 428      201.75     0.05694       16    9   16     2     0      0  1.3e-13  3.4e-15
   -      201.77           - out
   -       201.8           - out
 429       201.8    0.051246       18   10   18     2     0      0  1.4e-13    6e-15
   -      201.82           - out
 430      201.85    0.051246       20   11   20     2     0      0  4.2e-14  5.6e-15
   -      201.85           - out
   -      201.88           - out
 431       201.9    0.051246       22   12   22     2     0      0  1.9e-14  6.1e-15
   -      201.91           - out
   -      201.93           - out
 432      201.95    0.051246       24   13   24     2     0      0  6.8e-14  4.3e-15
   -      201.96           - out
   -      201.99           - out
```

| | | | | | | | | | | |
|---|---|---|---|---|---|---|---|---|---|---|
| 433 | 202 | 0.051246 | 26 | 14 | 26 | 2 | 0 | 0 | 1.8e-14 | 3.7e-15 |
| - | 202.02 | - out | | | | | | | | |
| - | 202.04 | - out | | | | | | | | |
| 434 | 202.05 | 0.051246 | 28 | 15 | 28 | 2 | 0 | 0 | 4.1e-14 | 5e-15 |
| - | 202.07 | - out | | | | | | | | |
| - | 202.1 | - out | | | | | | | | |
| 435 | 202.1 | 0.051246 | 30 | 16 | 30 | 2 | 0 | 0 | 1.4e-14 | 4.2e-15 |
| - | 202.13 | - out | | | | | | | | |
| - | 202.15 | - out | | | | | | | | |
| 436 | 202.16 | 0.051246 | 32 | 17 | 32 | 2 | 0 | 0 | 4.5e-14 | 4.2e-15 |
| - | 202.18 | - out | | | | | | | | |
| 437 | 202.21 | 0.051246 | 34 | 18 | 34 | 2 | 0 | 0 | 9.5e-15 | 3.7e-15 |
| - | 202.21 | - out | | | | | | | | |
| - | 202.24 | - out | | | | | | | | |
| 438 | 202.26 | 0.051246 | 36 | 19 | 36 | 2 | 0 | 0 | 9.9e-15 | 4.3e-15 |
| - | 202.26 | - out | | | | | | | | |
| - | 202.29 | - out | | | | | | | | |
| 439 | 202.31 | 0.051246 | 38 | 20 | 38 | 2 | 0 | 0 | 2.1e-14 | 9.8e-15 |
| - | 202.32 | - out | | | | | | | | |
| - | 202.35 | - out | | | | | | | | |
| - | 202.37 | - out | | | | | | | | |
| - | 202.4 | - out | | | | | | | | |
| 440 | 202.41 | 0.10249 | 40 | 21 | 40 | 2 | 0 | 0 | 1.3e-13 | 4.2e-15 |
| - | 202.43 | - out | | | | | | | | |
| - | 202.46 | - out | | | | | | | | |
| - | 202.48 | - out | | | | | | | | |
| 441 | 202.5 | 0.091526 | 42 | 22 | 42 | 2 | 0 | 0 | 1e-13 | 4e-15 |
| - | 202.51 | - out | | | | | | | | |
| - | 202.54 | - out | | | | | | | | |
| - | 202.57 | - out | | | | | | | | |
| 442 | 202.59 | 0.082373 | 44 | 23 | 44 | 2 | 0 | 0 | 1e-13 | 2.8e-15 |
| - | 202.59 | - out | | | | | | | | |
| - | 202.62 | - out | | | | | | | | |
| - | 202.65 | - out | | | | | | | | |
| 443 | 202.66 | 0.074136 | 46 | 24 | 46 | 2 | 0 | 0 | 5.5e-14 | 3e-15 |
| - | 202.68 | - out | | | | | | | | |
| - | 202.7 | - out | | | | | | | | |
| - | 202.73 | - out | | | | | | | | |
| 444 | 202.73 | 0.074136 | 48 | 25 | 48 | 2 | 0 | 0 | 2.1e-13 | 4.5e-15 |
| - | 202.76 | - out | | | | | | | | |
| - | 202.79 | - out | | | | | | | | |
| 445 | 202.81 | 0.074136 | 50 | 26 | 50 | 2 | 0 | 0 | 3.7e-13 | 3.7e-15 |
| - | 202.81 | - out | | | | | | | | |
| - | 202.84 | - out | | | | | | | | |
| - | 202.87 | - out | | | | | | | | |
| 446 | 202.88 | 0.074136 | 52 | 27 | 52 | 2 | 0 | 0 | 7.5e-13 | 5.4e-15 |
| - | 202.9 | - out | | | | | | | | |
| - | 202.92 | - out | | | | | | | | |
| - | 202.95 | - out | | | | | | | | |
| 447 | 202.96 | 0.074136 | 54 | 28 | 54 | 2 | 0 | 0 | 6.1e-13 | 3.5e-15 |
| - | 202.98 | - out | | | | | | | | |
| - | 203.01 | - out | | | | | | | | |
| 448 | 203.03 | 0.074136 | 56 | 29 | 56 | 2 | 0 | 0 | 5.4e-13 | 3.4e-15 |
| - | 203.03 | - out | | | | | | | | |
| - | 203.06 | - out | | | | | | | | |
| - | 203.09 | - out | | | | | | | | |

| | | | | | | | | | |
|---|---|---|---|---|---|---|---|---|---|
| - | 203.12 | - out | | | | | | | |
| - | 203.14 | - out | | | | | | | |
| - | 203.17 | - out | | | | | | | |
| 449 | 203.18 | 0.14827 | 58 | 30 | 58 | 2 | 0 | 0 1.3e-12 | 4.4e-15 |
| - | 203.2 | - out | | | | | | | |
| - | 203.23 | - out | | | | | | | |
| - | 203.25 | - out | | | | | | | |
| - | 203.28 | - out | | | | | | | |
| - | 203.31 | - out | | | | | | | |
| 450 | 203.33 | 0.14827 | 60 | 31 | 60 | 2 | 0 | 0 2.7e-13 | 3.1e-15 |
| - | 203.34 | - out | | | | | | | |
| - | 203.36 | - out | | | | | | | |
| - | 203.39 | - out | | | | | | | |
| - | 203.42 | - out | | | | | | | |
| - | 203.45 | - out | | | | | | | |
| - | 203.47 | - out | | | | | | | |
| 451 | 203.48 | 0.14827 | 62 | 32 | 62 | 2 | 0 | 0 4.4e-13 | 4.5e-15 |
| - | 203.5 | - out | | | | | | | |
| - | 203.53 | - out | | | | | | | |
| - | 203.56 | - out | | | | | | | |
| - | 203.58 | - out | | | | | | | |
| - | 203.61 | - out | | | | | | | |
| 452 | 203.62 | 0.14827 | 64 | 33 | 64 | 2 | 0 | 0 2.3e-13 | 8.4e-15 |
| - | 203.64 | - out | | | | | | | |
| - | 203.67 | - out | | | | | | | |
| - | 203.69 | - out | | | | | | | |
| - | 203.72 | - out | | | | | | | |
| - | 203.75 | - out | | | | | | | |
| - | 203.78 | - out | | | | | | | |
| - | 203.8 | - out | | | | | | | |
| - | 203.83 | - out | | | | | | | |
| - | 203.86 | - out | | | | | | | |
| - | 203.89 | - out | | | | | | | |
| - | 203.91 | - out | | | | | | | |
| 453 | 203.92 | 0.29654 | 66 | 34 | 66 | 2 | 0 | 0 2.2e-13 | 4.1e-15 |
| - | 203.94 | - out | | | | | | | |
| - | 203.97 | - out | | | | | | | |
| - | 204 | - out | | | | | | | |
| - | 204.02 | - out | | | | | | | |
| 454 | 204.04 | 0.11543 | 70 | 36 | 70 | 2 | 1 | 0 4.3e-13 | 4.2e-15 |
| - | 204.05 | - out | | | | | | | |
| - | 204.08 | - out | | | | | | | |
| - | 204.11 | - out | | | | | | | |
| - | 204.13 | - out | | | | | | | |
| 455 | 204.15 | 0.11543 | 72 | 37 | 72 | 2 | 1 | 0 4.1e-13 | 4.2e-15 |
| - | 204.16 | - out | | | | | | | |
| - | 204.19 | - out | | | | | | | |
| 456 | 204.21 | 0.063119 | 76 | 39 | 76 | 2 | 2 | 0 6.4e-13 | 5e-15 |
| - | 204.22 | - out | | | | | | | |
| - | 204.24 | - out | | | | | | | |
| - | 204.27 | - out | | | | | | | |
| 457 | 204.28 | 0.063119 | 78 | 40 | 78 | 2 | 2 | 0 2.6e-13 | 3.9e-15 |
| - | 204.3 | - out | | | | | | | |
| - | 204.33 | - out | | | | | | | |
| 458 | 204.33 | 0.056808 | 80 | 41 | 80 | 2 | 2 | 0 1.6e-13 | 4.3e-15 |
| - | 204.35 | - out | | | | | | | |

| | | | | | | | | | | |
|---|---|---|---|---|---|---|---|---|---|---|
| - | 204.38 | - out | | | | | | | | |
| 459 | 204.39 | 0.056808 | 82 | 42 | 82 | 2 | 2 | 0 | 8.1e-14 | 6.5e-15 |
| - | 204.41 | - out | | | | | | | | |
| - | 204.44 | - out | | | | | | | | |
| 460 | 204.45 | 0.056808 | 84 | 43 | 84 | 2 | 2 | 0 | 1e-13 | 6.7e-15 |
| - | 204.46 | - out | | | | | | | | |
| - | 204.49 | - out | | | | | | | | |
| 461 | 204.5 | 0.056808 | 86 | 44 | 86 | 2 | 2 | 0 | 8.8e-14 | 8.5e-15 |
| - | 204.52 | - out | | | | | | | | |
| - | 204.55 | - out | | | | | | | | |
| 462 | 204.56 | 0.056808 | 88 | 45 | 88 | 2 | 2 | 0 | 4.1e-14 | 1.3e-14 |
| - | 204.57 | - out | | | | | | | | |
| - | 204.6 | - out | | | | | | | | |
| 463 | 204.62 | 0.056808 | 90 | 46 | 90 | 2 | 2 | 0 | 3.1e-14 | 1.8e-14 |
| - | 204.63 | - out | | | | | | | | |
| - | 204.66 | - out | | | | | | | | |
| 464 | 204.67 | 0.056808 | 92 | 47 | 92 | 2 | 2 | 0 | 1.9e-14 | 2.4e-14 |
| - | 204.68 | - out | | | | | | | | |
| - | 204.71 | - out | | | | | | | | |
| - | 204.74 | - out | | | | | | | | |
| - | 204.77 | - out | | | | | | | | |
| 465 | 204.79 | 0.11362 | 94 | 48 | 94 | 2 | 2 | 0 | 1.4e-14 | 5.2e-14 |
| - | 204.79 | - out | | | | | | | | |
| - | 204.82 | - out | | | | | | | | |
| - | 204.85 | - out | | | | | | | | |
| - | 204.88 | - out | | | | | | | | |
| 466 | 204.89 | 0.10225 | 96 | 49 | 96 | 2 | 2 | 0 | 1.2e-13 | 8e-15 |
| - | 204.9 | - out | | | | | | | | |
| - | 204.93 | - out | | | | | | | | |
| - | 204.96 | - out | | | | | | | | |
| - | 204.99 | - out | | | | | | | | |
| 467 | 204.99 | 0.10225 | 98 | 50 | 98 | 2 | 2 | 0 | 8.2e-14 | 7.2e-15 |
| - | 205.01 | - out | | | | | | | | |
| - | 205.04 | - out | | | | | | | | |
| - | 205.07 | - out | | | | | | | | |
| 468 | 205.08 | 0.092028 | 100 | 51 | 100 | 2 | 2 | 0 | 5.8e-14 | 5.4e-15 |
| - | 205.1 | - out | | | | | | | | |
| - | 205.12 | - out | | | | | | | | |
| - | 205.15 | - out | | | | | | | | |
| 469 | 205.16 | 0.079069 | 102 | 52 | 102 | 2 | 2 | 0 | 7.5e-14 | 4.4e-15 |
| - | 205.18 | - out | | | | | | | | |
| - | 205.21 | - out | | | | | | | | |
| - | 205.23 | - out | | | | | | | | |
| 470 | 205.24 | 0.071162 | 104 | 53 | 104 | 2 | 2 | 0 | 1.4e-13 | 6.1e-15 |
| - | 205.26 | - out | | | | | | | | |
| - | 205.29 | - out | | | | | | | | |
| 471 | 205.31 | 0.071162 | 106 | 54 | 106 | 2 | 2 | 0 | 1.8e-13 | 5.3e-15 |
| - | 205.32 | - out | | | | | | | | |
| - | 205.34 | - out | | | | | | | | |
| - | 205.37 | - out | | | | | | | | |
| 472 | 205.38 | 0.071162 | 108 | 55 | 108 | 2 | 2 | 0 | 8.3e-14 | 3.8e-15 |
| - | 205.4 | - out | | | | | | | | |
| - | 205.43 | - out | | | | | | | | |
| 473 | 205.45 | 0.071162 | 110 | 56 | 110 | 2 | 2 | 0 | 1.3e-13 | 4.8e-15 |
| - | 205.45 | - out | | | | | | | | |
| - | 205.48 | - out | | | | | | | | |

| | | | | | | | | | | | |
|---|---|---|---|---|---|---|---|---|---|---|---|
| - | 205.51 | - | out | | | | | | | | |
| 474 | 205.52 | 0.071162 | | 112 | 57 | 112 | 2 | 2 | 0 | 3.2e-13 | 4.4e-15 |
| - | 205.54 | - | out | | | | | | | | |
| - | 205.56 | - | out | | | | | | | | |
| - | 205.59 | - | out | | | | | | | | |
| 475 | 205.59 | 0.071162 | | 114 | 58 | 114 | 2 | 2 | 0 | 9.8e-14 | 2.8e-15 |
| - | 205.62 | - | out | | | | | | | | |
| - | 205.65 | - | out | | | | | | | | |
| 476 | 205.66 | 0.071162 | | 116 | 59 | 116 | 2 | 2 | 0 | 5.6e-13 | 4.3e-15 |
| - | 205.67 | - | out | | | | | | | | |
| - | 205.7 | - | out | | | | | | | | |
| - | 205.73 | - | out | | | | | | | | |
| 477 | 205.73 | 0.071162 | | 118 | 60 | 118 | 2 | 2 | 0 | 1.3e-12 | 5.5e-15 |
| - | 205.76 | - | out | | | | | | | | |
| - | 205.78 | - | out | | | | | | | | |
| 478 | 205.8 | 0.071162 | | 120 | 61 | 120 | 2 | 2 | 0 | 1.1e-13 | 4.8e-15 |
| - | 205.81 | - | out | | | | | | | | |
| - | 205.84 | - | out | | | | | | | | |
| - | 205.87 | - | out | | | | | | | | |
| 479 | 205.88 | 0.071162 | | 122 | 62 | 122 | 2 | 2 | 0 | 7.6e-13 | 3.8e-15 |
| - | 205.89 | - | out | | | | | | | | |
| - | 205.92 | - | out | | | | | | | | |
| 480 | 205.95 | 0.071162 | | 124 | 63 | 124 | 2 | 2 | 0 | 1.3e-12 | 5.7e-15 |
| - | 205.95 | - | out | | | | | | | | |
| - | 205.98 | - | out | | | | | | | | |
| - | 206 | - | out | | | | | | | | |
| 481 | 206.02 | 0.071162 | | 126 | 64 | 126 | 2 | 2 | 0 | 4.4e-13 | 4.1e-15 |
| - | 206.03 | - | out | | | | | | | | |
| - | 206.06 | - | out | | | | | | | | |
| - | 206.09 | - | out | | | | | | | | |
| 482 | 206.09 | 0.071162 | | 128 | 65 | 128 | 2 | 2 | 0 | 4.8e-13 | 3.4e-15 |
| - | 206.11 | - | out | | | | | | | | |
| - | 206.14 | - | out | | | | | | | | |
| - | 206.17 | - | out | | | | | | | | |
| - | 206.2 | - | out | | | | | | | | |
| - | 206.22 | - | out | | | | | | | | |
| 483 | 206.23 | 0.14232 | | 130 | 66 | 130 | 2 | 2 | 0 | 2.9e-13 | 4.4e-15 |
| - | 206.25 | - | out | | | | | | | | |
| - | 206.28 | - | out | | | | | | | | |
| - | 206.31 | - | out | | | | | | | | |
| - | 206.33 | - | out | | | | | | | | |
| - | 206.36 | - | out | | | | | | | | |
| 484 | 206.37 | 0.14232 | | 132 | 67 | 132 | 2 | 2 | 0 | 3.3e-14 | 2.4e-14 |
| - | 206.39 | - | out | | | | | | | | |
| - | 206.42 | - | out | | | | | | | | |
| - | 206.44 | - | out | | | | | | | | |
| - | 206.47 | - | out | | | | | | | | |
| - | 206.5 | - | out | | | | | | | | |
| 485 | 206.52 | 0.14232 | | 134 | 68 | 134 | 2 | 2 | 0 | 2.5e-14 | 2.2e-14 |
| - | 206.52 | - | out | | | | | | | | |
| 486 | 206.66 | 0.14232 | | 136 | 69 | 136 | 2 | 2 | 0 | 4.6e-13 | 3.4e-15 |

Number of vertex elements: 3
Number of boundary elements: 112
Number of vertex elements: 3
Number of boundary elements: 112
Number of elements: 534

```
Minimum element quality: 0.5661
Number of vertex elements: 4
Number of boundary elements: 162
Number of vertex elements: 5
Number of boundary elements: 212
Number of vertex elements: 6
Number of boundary elements: 266
Minimum element quality: 0.07019
Geometry shape function: Linear Lagrange
Time interval 6
Time-dependent solver (BDF)
Number of degrees of freedom solved for: 11758 (plus 7124 internal DOFs).
Nonsymmetric matrix found.
Scales for dependent variables:
Concentration (comp1.cOx): 4.8
Concentration (comp1.cRed): 3.2
Spatial mesh displacement (comp1.spatial.disp): 1.4e-07
Step        Time    Stepsize      Res  Jac  Sol Order Tfail NLfail   LinErr   LinRes
   -      206.52           - out
   -      206.53           - out
 487      206.54      0.0275        2    2    2     1     0      0  1.6e-14  2.7e-15
   -      206.55           - out
 488      206.57      0.0275        4    3    4     1     0      0  1.5e-13  3.6e-15
   -      206.58           - out
   -      206.61           - out
 489      206.63       0.055        6    4    6     2     0      0  2.9e-14  2.2e-15
   -      206.64           - out
   -      206.66           - out
 490      206.68       0.055        8    5    8     2     0      0  4.9e-13  5.2e-15
   -      206.69           - out
   -      206.72           - out
   -      206.75           - out
   -      206.77           - out
 491      206.79        0.11       10    6   10     2     0      0  1.5e-13  3.1e-15
   -       206.8           - out
   -      206.83           - out
   -      206.86           - out
   -      206.88           - out
 492       206.9        0.11       12    7   12     2     0      0  6.2e-13  6.1e-15
   -      206.91           - out
   -      206.94           - out
 493      206.96    0.063869       16    9   16     2     1      0  6.2e-14  4.8e-15
   -      206.97           - out
   -      206.99           - out
   -      207.02           - out
 494      207.03    0.063869       18   10   18     2     1      0  2.3e-13    4e-15
   -      207.05           - out
   -      207.08           - out
 495      207.09    0.057482       20   11   20     2     1      0  2.8e-14  5.2e-15
   -       207.1           - out
   -      207.13           - out
 496      207.14    0.051734       22   12   22     2     1      0    5e-14  5.9e-15
   -      207.16           - out
 497      207.18    0.046561       24   13   24     2     1      0  7.1e-14  4.8e-15
   -      207.19           - out
   -      207.21           - out
```

| | | | | | | | | | | |
|---|---|---|---|---|---|---|---|---|---|---|
| 498 | 207.23 | 0.046561 | 26 | 14 | 26 | 2 | 1 | 0 | 4.9e-14 | 4e-15 |
| - | 207.24 | - out | | | | | | | | |
| - | 207.27 | - out | | | | | | | | |
| 499 | 207.28 | 0.046561 | 28 | 15 | 28 | 2 | 1 | 0 | 1.1e-13 | 5.5e-15 |
| - | 207.3 | - out | | | | | | | | |
| - | 207.32 | - out | | | | | | | | |
| 500 | 207.32 | 0.046561 | 30 | 16 | 30 | 2 | 1 | 0 | 5.2e-14 | 4.1e-15 |
| - | 207.35 | - out | | | | | | | | |
| 501 | 207.37 | 0.046561 | 32 | 17 | 32 | 2 | 1 | 0 | 2.6e-14 | 2.6e-14 |
| - | 207.38 | - out | | | | | | | | |
| - | 207.41 | - out | | | | | | | | |
| 502 | 207.42 | 0.046561 | 34 | 18 | 34 | 2 | 1 | 0 | 2.7e-14 | 4e-15 |
| - | 207.43 | - out | | | | | | | | |
| - | 207.46 | - out | | | | | | | | |
| 503 | 207.46 | 0.046561 | 36 | 19 | 36 | 2 | 1 | 0 | 3.8e-14 | 4.4e-15 |
| - | 207.49 | - out | | | | | | | | |
| 504 | 207.51 | 0.046561 | 38 | 20 | 38 | 2 | 1 | 0 | 7.6e-14 | 4.3e-15 |
| - | 207.52 | - out | | | | | | | | |
| - | 207.54 | - out | | | | | | | | |
| 505 | 207.56 | 0.046561 | 40 | 21 | 40 | 2 | 1 | 0 | 4.7e-14 | 5.7e-15 |
| - | 207.57 | - out | | | | | | | | |
| - | 207.6 | - out | | | | | | | | |
| 506 | 207.6 | 0.046561 | 42 | 22 | 42 | 2 | 1 | 0 | 2.5e-14 | 5.2e-15 |
| - | 207.63 | - out | | | | | | | | |
| 507 | 207.65 | 0.046561 | 44 | 23 | 44 | 2 | 1 | 0 | 2.8e-14 | 4.2e-15 |
| - | 207.65 | - out | | | | | | | | |
| - | 207.68 | - out | | | | | | | | |
| 508 | 207.7 | 0.046561 | 46 | 24 | 46 | 2 | 1 | 0 | 5.8e-14 | 5.1e-15 |
| - | 207.71 | - out | | | | | | | | |
| - | 207.74 | - out | | | | | | | | |
| 509 | 207.74 | 0.046561 | 48 | 25 | 48 | 2 | 1 | 0 | 3.1e-14 | 6.4e-15 |
| - | 207.76 | - out | | | | | | | | |
| 510 | 207.79 | 0.046561 | 50 | 26 | 50 | 2 | 1 | 0 | 4.2e-14 | 5.5e-15 |
| - | 207.79 | - out | | | | | | | | |
| - | 207.82 | - out | | | | | | | | |
| - | 207.85 | - out | | | | | | | | |
| - | 207.87 | - out | | | | | | | | |
| 511 | 207.88 | 0.093121 | 52 | 27 | 52 | 2 | 1 | 0 | 6.2e-14 | 4.7e-15 |
| - | 207.9 | - out | | | | | | | | |
| - | 207.93 | - out | | | | | | | | |
| - | 207.96 | - out | | | | | | | | |
| 512 | 207.97 | 0.083809 | 54 | 28 | 54 | 2 | 1 | 0 | 2e-13 | 4.7e-15 |
| - | 207.98 | - out | | | | | | | | |
| - | 208.01 | - out | | | | | | | | |
| - | 208.04 | - out | | | | | | | | |
| 513 | 208.05 | 0.083809 | 56 | 29 | 56 | 2 | 1 | 0 | 1e-13 | 4.2e-15 |
| - | 208.07 | - out | | | | | | | | |
| - | 208.09 | - out | | | | | | | | |
| - | 208.12 | - out | | | | | | | | |
| 514 | 208.13 | 0.075428 | 58 | 30 | 58 | 2 | 1 | 0 | 4.6e-14 | 2.8e-15 |
| - | 208.15 | - out | | | | | | | | |
| - | 208.18 | - out | | | | | | | | |
| 515 | 208.2 | 0.075428 | 60 | 31 | 60 | 2 | 1 | 0 | 1.9e-13 | 4.2e-15 |
| - | 208.2 | - out | | | | | | | | |
| - | 208.23 | - out | | | | | | | | |
| - | 208.26 | - out | | | | | | | | |

```
 516      208.28    0.075428      62   32   62     2     1      0    2e-13    3e-15
   -      208.29           - out
   -      208.31           - out
   -      208.34           - out
 517      208.35    0.075428      64   33   64     2     1      0  3.9e-13  4.3e-15
   -      208.37           - out
   -       208.4           - out
   -      208.42           - out
 518      208.43    0.075428      66   34   66     2     1      0    1e-12    3e-15
   -      208.45           - out
   -      208.48           - out
 519       208.5    0.075428      68   35   68     2     1      0  4.4e-13  4.4e-15
   -      208.51           - out
   -      208.53           - out
   -      208.56           - out
 520      208.58    0.075428      70   36   70     2     1      0  3.8e-13  3.4e-15
   -      208.59           - out
   -      208.62           - out
   -      208.64           - out
   -      208.67           - out
   -       208.7           - out
   -      208.73           - out
 521      208.73     0.15086      72   37   72     2     1      0  7.3e-13    3e-15
   -      208.75           - out
   -      208.78           - out
   -      208.81           - out
   -      208.84           - out
   -      208.86           - out
 522      208.88     0.15086      74   38   74     2     1      0  1.4e-12  3.3e-15
   -      208.89           - out
   -      208.92           - out
   -      208.95           - out
   -      208.97           - out
   -         209           - out
 523      209.03     0.15086      76   39   76     2     1      0  2.1e-13  4.7e-15
Time-stepping completed.
Geometry shape function: Linear Lagrange
Solution time: 639 s. (10 minutes, 39 seconds)
Physical memory: 2.82 GB
Virtual memory: 2.92 GB
Ended at May 31, 2023 11:17:24 AM.
----- Time-Dependent Solver 1 in Study 4 (CV 31 to 38)/Solution 8 (sol8) ------>
```

#### Advanced (aDef)

##### ASSEMBLY SETTINGS

| Description | Value |
| --- | --- |
| Reuse sparsity pattern | On |

#### Fully Coupled 1 (fc1)

##### GENERAL

| Description | Value |
| --- | --- |

| Description | Value |
| --- | --- |
| Linear solver | Direct 1 |

### METHOD AND TERMINATION

| Description | Value |
| --- | --- |
| Damping factor | 0.9 |
| Jacobian update | Once per time step |
| Maximum number of iterations | 8 |
| Stabilization and acceleration | Anderson acceleration |
| Dimension of iteration space | 5 |

**Automatic Remeshing 1 (ar1)**

### GENERAL

| Description | Value |
| --- | --- |
| Remesh in geometry | Geometry 1 |

### CONDITION FOR REMESHING

| Description | Value |
| --- | --- |
| Condition type | Distortion |

### OUTPUT

| Description | Value |
| --- | --- |
| Solution | Remeshed Solution 4 |
| Meshes | {mesh6, mesh7, mesh8, mesh9, mesh10} |

# 7 Study 5 (CV 39 to 44)

COMPUTATION INFORMATION

| | |
|---|---|
| Computation time | 17 min 17 s |

## 7.1 TIME DEPENDENT

| Times | Unit |
|---|---|
| range(38*t_cv,t_tot/nb/200,44*t_cv) | s |

STUDY SETTINGS

| Description | Value |
|---|---|
| Include geometric nonlinearity | Off |

STUDY SETTINGS

| Description | Value |
|---|---|
| Output times | {209.00000000000003, 209.02750000000003, 209.05500000000004, 209.08250000000004, 209.11000000000004, 209.13750000000002, 209.16500000000002, 209.19250000000002, 209.22000000000003, 209.24750000000003, 209.27500000000003, 209.30250000000004, 209.33000000000004, 209.35750000000002, 209.38500000000002, 209.41250000000002, 209.44000000000003, 209.46750000000003, 209.49500000000003, 209.52250000000004, 209.55000000000004, 209.57750000000001, 209.60500000000002, 209.63250000000002, 209.66000000000003, 209.68750000000003, 209.71500000000003, 209.74250000000004, 209.77000000000004, 209.79750000000004, 209.82500000000002, 209.85250000000002, 209.88000000000002, 209.90750000000003, 209.93500000000003, 209.96250000000003, 209.99000000000004, 210.01750000000004, 210.04500000000002, 210.07250000000002, 210.10000000000002, 210.12750000000003, 210.15500000000003, 210.18250000000003, 210.21000000000004, 210.23750000000004, 210.26500000000001, 210.29250000000002, 210.32000000000002, 210.34750000000003, 210.37500000000003, 210.40250000000003, 210.43000000000004, 210.45750000000004, 210.48500000000004, 210.51250000000002, 210.54000000000002, 210.56750000000002, 210.59500000000003, 210.62250000000003, 210.65000000000003, 210.67750000000004, 210.70500000000004, 210.73250000000002, 210.76000000000002, 210.78750000000002, 210.81500000000003, 210.84250000000003, 210.87000000000003, 210.89750000000004, 210.92500000000004, 210.95250000000001, 210.98000000000002, 211.00750000000002, 211.03500000000003, 211.06250000000003, 211.09000000000003, 211.11750000000004, 211.14500000000004, 211.17250000000004, 211.20000000000002, 211.22750000000002, 211.25500000000002, 211.28250000000003, 211.31000000000003, 211.33750000000003, 211.36500000000004, 211.39250000000004, 211.42000000000002, 211.44750000000002, 211.47500000000002, 211.50250000000003, 211.53000000000003, |

| Description | Value |
| --- | --- |
| | 211.55750000000003, 211.58500000000004, 211.61250000000004, 211.64000000000004, 211.66750000000002, 211.69500000000002, 211.72250000000003, 211.75000000000003, 211.77750000000003, 211.80500000000004, 211.83250000000004, 211.86000000000004, 211.88750000000002, 211.91500000000002, 211.94250000000002, 211.97000000000003, 211.99750000000003, 212.02500000000003, 212.05250000000004, 212.08000000000004, 212.10750000000002, 212.13500000000002, 212.16250000000002, 212.19000000000003, 212.21750000000003, 212.24500000000003, 212.27250000000004, 212.30000000000004, 212.32750000000004, 212.35500000000002, 212.38250000000002, 212.41000000000003, 212.43750000000003, 212.46500000000003, 212.49250000000004, 212.52000000000004, 212.54750000000004, 212.57500000000002, 212.60250000000002, 212.63000000000002, 212.65750000000003, 212.68500000000003, 212.71250000000003, 212.74000000000004, 212.76750000000004, 212.79500000000002, 212.82250000000002, 212.85000000000002, 212.87750000000003, 212.90500000000003, 212.93250000000003, 212.96000000000004, 212.98750000000004, 213.01500000000004, 213.04250000000002, 213.07000000000002, 213.09750000000003, 213.12500000000003, 213.15250000000003, 213.18000000000004, 213.20750000000004, 213.23500000000004, 213.26250000000002, 213.29000000000002, 213.31750000000002, 213.34500000000003, 213.37250000000003, 213.40000000000003, 213.42750000000004, 213.45500000000004, 213.48250000000002, 213.51000000000002, 213.53750000000002, 213.56500000000003, 213.59250000000003, 213.62000000000003, 213.64750000000004, 213.67500000000004, 213.70250000000004, 213.73000000000002, 213.75750000000002, 213.78500000000003, 213.81250000000003, 213.84000000000003, 213.86750000000004, 213.89500000000004, 213.92250000000004, 213.95000000000002, 213.97750000000002, 214.00500000000002, 214.03250000000003, 214.06000000000003, 214.08750000000003, 214.11500000000004, 214.14250000000004, 214.17000000000002, 214.19750000000002, 214.22500000000002, 214.25250000000003, 214.28000000000003, 214.30750000000003, 214.33500000000004, 214.36250000000004, 214.39000000000004, 214.41750000000002, 214.44500000000002, 214.47250000000003, 214.50000000000003, 214.52750000000003, 214.55500000000004, 214.58250000000004, 214.61000000000004, 214.63750000000002, 214.66500000000002, 214.69250000000002, 214.72000000000003, 214.74750000000003, 214.77500000000003, 214.80250000000004, 214.83000000000004, 214.85750000000002, 214.88500000000002, 214.91250000000002, 214.94000000000003, 214.96750000000003, 214.99500000000003, 215.02250000000004, 215.05000000000004, 215.07750000000004, 215.10500000000002, 215.13250000000002, 215.16000000000003, 215.18750000000003, 215.21500000000003, 215.24250000000004, 215.27000000000004, 215.29750000000004, 215.32500000000002, 215.35250000000002, 215.38000000000002, 215.40750000000003, 215.43500000000003, 215.46250000000003, 215.49000000000004, |

| Description | Value |
|---|---|
| | 215.51750000000004, 215.54500000000002, 215.57250000000002, 215.60000000000002, 215.62750000000003, 215.65500000000003, 215.68250000000003, 215.71000000000004, 215.73750000000004, 215.76500000000004, 215.79250000000002, 215.82000000000002, 215.84750000000003, 215.87500000000003, 215.90250000000003, 215.93000000000004, 215.95750000000004, 215.98500000000004, 216.01250000000002, 216.04000000000002, 216.06750000000002, 216.09500000000003, 216.12250000000003, 216.15000000000003, 216.17750000000004, 216.20500000000004, 216.23250000000002, 216.26000000000002, 216.28750000000002, 216.31500000000003, 216.34250000000003, 216.37000000000003, 216.39750000000004, 216.42500000000004, 216.45250000000004, 216.48000000000002, 216.50750000000002, 216.53500000000003, 216.56250000000003, 216.59000000000003, 216.61750000000004, 216.64500000000004, 216.67250000000004, 216.70000000000002, 216.72750000000002, 216.75500000000002, 216.78250000000003, 216.81000000000003, 216.83750000000003, 216.86500000000004, 216.89250000000004, 216.92000000000002, 216.94750000000002, 216.97500000000002, 217.00250000000003, 217.03000000000003, 217.05750000000003, 217.08500000000004, 217.11250000000004, 217.14000000000004, 217.16750000000002, 217.19500000000002, 217.22250000000003, 217.25000000000003, 217.27750000000003, 217.30500000000004, 217.33250000000004, 217.36000000000004, 217.38750000000002, 217.41500000000002, 217.44250000000002, 217.47000000000003, 217.49750000000003, 217.52500000000003, 217.55250000000004, 217.58000000000004, 217.60750000000002, 217.63500000000002, 217.66250000000002, 217.69000000000003, 217.71750000000003, 217.74500000000003, 217.77250000000004, 217.80000000000004, 217.82750000000004, 217.85500000000002, 217.88250000000002, 217.91000000000003, 217.93750000000003, 217.96500000000003, 217.99250000000004, 218.02000000000004, 218.04750000000004, 218.07500000000002, 218.10250000000002, 218.13000000000002, 218.15750000000003, 218.18500000000003, 218.21250000000003, 218.24000000000004, 218.26750000000004, 218.29500000000002, 218.32250000000002, 218.35000000000002, 218.37750000000003, 218.40500000000003, 218.43250000000003, 218.46000000000004, 218.48750000000004, 218.51500000000004, 218.54250000000002, 218.57000000000002, 218.59750000000003, 218.62500000000003, 218.65250000000003, 218.68000000000004, 218.70750000000004, 218.73500000000004, 218.76250000000002, 218.79000000000002, 218.81750000000002, 218.84500000000003, 218.87250000000003, 218.90000000000003, 218.92750000000004, 218.95500000000004, 218.98250000000002, 219.01000000000002, 219.03750000000002, 219.06500000000003, 219.09250000000003, 219.12000000000003, 219.14750000000004, 219.17500000000004, 219.20250000000004, 219.23000000000002, 219.25750000000002, 219.28500000000003, 219.31250000000003, 219.34000000000003, 219.36750000000004, 219.39500000000004, 219.42250000000004, 219.45000000000002, |

| Description | Value |
| --- | --- |
| | 219.47750000000002, 219.50500000000002, 219.53250000000003, 219.56000000000003, 219.58750000000003, 219.61500000000004, 219.64250000000004, 219.67000000000002, 219.69750000000002, 219.72500000000002, 219.75250000000003, 219.78000000000003, 219.80750000000003, 219.83500000000004, 219.86250000000004, 219.89000000000004, 219.91750000000002, 219.94500000000002, 219.97250000000003, 220.00000000000003, 220.02750000000003, 220.05500000000004, 220.08250000000004, 220.11000000000004, 220.13750000000002, 220.16500000000002, 220.19250000000002, 220.22000000000003, 220.24750000000003, 220.27500000000003, 220.30250000000004, 220.33000000000004, 220.35750000000002, 220.38500000000002, 220.41250000000002, 220.44000000000003, 220.46750000000003, 220.49500000000003, 220.52250000000004, 220.55000000000004, 220.57750000000004, 220.60500000000002, 220.63250000000002, 220.66000000000003, 220.68750000000003, 220.71500000000003, 220.74250000000004, 220.77000000000004, 220.79750000000004, 220.82500000000002, 220.85250000000002, 220.88000000000002, 220.90750000000003, 220.93500000000003, 220.96250000000003, 220.99000000000004, 221.01750000000004, 221.04500000000002, 221.07250000000002, 221.10000000000002, 221.12750000000003, 221.15500000000003, 221.18250000000003, 221.21000000000004, 221.23750000000004, 221.26500000000004, 221.29250000000002, 221.32000000000002, 221.34750000000003, 221.37500000000003, 221.40250000000003, 221.43000000000004, 221.45750000000004, 221.48500000000004, 221.51250000000002, 221.54000000000002, 221.56750000000002, 221.59500000000003, 221.62250000000003, 221.65000000000003, 221.67750000000004, 221.70500000000004, 221.73250000000002, 221.76000000000002, 221.78750000000002, 221.81500000000003, 221.84250000000003, 221.87000000000003, 221.89750000000004, 221.92500000000004, 221.95250000000004, 221.98000000000002, 222.00750000000002, 222.03500000000003, 222.06250000000003, 222.09000000000003, 222.11750000000004, 222.14500000000004, 222.17250000000004, 222.20000000000002, 222.22750000000002, 222.25500000000002, 222.28250000000003, 222.31000000000003, 222.33750000000003, 222.36500000000004, 222.39250000000004, 222.42000000000002, 222.44750000000002, 222.47500000000002, 222.50250000000003, 222.53000000000003, 222.55750000000003, 222.58500000000004, 222.61250000000004, 222.64000000000004, 222.66750000000002, 222.69500000000002, 222.72250000000003, 222.75000000000003, 222.77750000000003, 222.80500000000004, 222.83250000000004, 222.86000000000004, 222.88750000000002, 222.91500000000002, 222.94250000000002, 222.97000000000003, 222.99750000000003, 223.02500000000003, 223.05250000000004, 223.08000000000004, 223.10750000000002, 223.13500000000002, 223.16250000000002, 223.19000000000003, 223.21750000000003, 223.24500000000003, 223.27250000000004, 223.30000000000004, 223.32750000000004, 223.35500000000002, 223.38250000000002, 223.41000000000003, |

| Description | Value |
| --- | --- |
| | 223.43750000000003, 223.46500000000003, 223.49250000000004, 223.52000000000004, 223.54750000000004, 223.57500000000002, 223.60250000000002, 223.63000000000002, 223.65750000000003, 223.68500000000003, 223.71250000000003, 223.74000000000004, 223.76750000000004, 223.79500000000002, 223.82250000000002, 223.85000000000002, 223.87750000000003, 223.90500000000003, 223.93250000000003, 223.96000000000004, 223.98750000000004, 224.01500000000004, 224.04250000000002, 224.07000000000002, 224.09750000000003, 224.12500000000003, 224.15250000000003, 224.18000000000004, 224.20750000000004, 224.23500000000004, 224.26250000000005, 224.29000000000002, 224.31750000000002, 224.34500000000003, 224.37250000000003, 224.40000000000003, 224.42750000000004, 224.45500000000004, 224.48250000000002, 224.51000000000002, 224.53750000000002, 224.56500000000003, 224.59250000000003, 224.62000000000003, 224.64750000000004, 224.67500000000004, 224.70250000000004, 224.73000000000002, 224.75750000000002, 224.78500000000003, 224.81250000000003, 224.84000000000003, 224.86750000000004, 224.89500000000004, 224.92250000000004, 224.95000000000005, 224.97750000000002, 225.00500000000002, 225.03250000000003, 225.06000000000003, 225.08750000000003, 225.11500000000004, 225.14250000000004, 225.17000000000002, 225.19750000000002, 225.22500000000002, 225.25250000000003, 225.28000000000003, 225.30750000000003, 225.33500000000004, 225.36250000000004, 225.39000000000004, 225.41750000000002, 225.44500000000002, 225.47250000000003, 225.50000000000003, 225.52750000000003, 225.55500000000004, 225.58250000000004, 225.61000000000004, 225.63750000000005, 225.66500000000002, 225.69250000000002, 225.72000000000003, 225.74750000000003, 225.77500000000003, 225.80250000000004, 225.83000000000004, 225.85750000000002, 225.88500000000002, 225.91250000000002, 225.94000000000003, 225.96750000000003, 225.99500000000003, 226.02250000000004, 226.05000000000004, 226.07750000000004, 226.10500000000002, 226.13250000000002, 226.16000000000003, 226.18750000000003, 226.21500000000003, 226.24250000000004, 226.27000000000004, 226.29750000000004, 226.32500000000005, 226.35250000000002, 226.38000000000002, 226.40750000000003, 226.43500000000003, 226.46250000000003, 226.49000000000004, 226.51750000000004, 226.54500000000002, 226.57250000000002, 226.60000000000002, 226.62750000000003, 226.65500000000003, 226.68250000000003, 226.71000000000004, 226.73750000000004, 226.76500000000004, 226.79250000000002, 226.82000000000002, 226.84750000000003, 226.87500000000003, 226.90250000000003, 226.93000000000004, 226.95750000000004, 226.98500000000004, 227.01250000000005, 227.04000000000002, 227.06750000000002, 227.09500000000003, 227.12250000000003, 227.15000000000003, 227.17750000000004, 227.20500000000004, 227.23250000000002, 227.26000000000002, 227.28750000000002, 227.31500000000003, 227.34250000000003, 227.37000000000003, |

| Description | Value |
| --- | --- |
| | 227.39750000000004, 227.42500000000004, 227.45250000000004, 227.48000000000002, 227.50750000000002, 227.53500000000003, 227.56250000000003, 227.59000000000003, 227.61750000000004, 227.64500000000004, 227.67250000000004, 227.70000000000005, 227.72750000000002, 227.75500000000002, 227.78250000000003, 227.81000000000003, 227.83750000000003, 227.86500000000004, 227.89250000000004, 227.92000000000002, 227.94750000000002, 227.97500000000002, 228.00250000000003, 228.03000000000003, 228.05750000000003, 228.08500000000004, 228.11250000000004, 228.14000000000004, 228.16750000000002, 228.19500000000002, 228.22250000000003, 228.25000000000003, 228.27750000000003, 228.30500000000004, 228.33250000000004, 228.36000000000004, 228.38750000000005, 228.41500000000002, 228.44250000000002, 228.47000000000003, 228.49750000000003, 228.52500000000003, 228.55250000000004, 228.58000000000004, 228.60750000000002, 228.63500000000002, 228.66250000000002, 228.69000000000003, 228.71750000000003, 228.74500000000003, 228.77250000000004, 228.80000000000004, 228.82750000000004, 228.85500000000002, 228.88250000000002, 228.91000000000003, 228.93750000000003, 228.96500000000003, 228.99250000000004, 229.02000000000004, 229.04750000000004, 229.07500000000005, 229.10250000000002, 229.13000000000002, 229.15750000000003, 229.18500000000003, 229.21250000000003, 229.24000000000004, 229.26750000000004, 229.29500000000002, 229.32250000000002, 229.35000000000002, 229.37750000000003, 229.40500000000003, 229.43250000000003, 229.46000000000004, 229.48750000000004, 229.51500000000004, 229.54250000000002, 229.57000000000002, 229.59750000000003, 229.62500000000003, 229.65250000000003, 229.68000000000004, 229.70750000000004, 229.73500000000004, 229.76250000000005, 229.79000000000002, 229.81750000000002, 229.84500000000003, 229.87250000000003, 229.90000000000003, 229.92750000000004, 229.95500000000004, 229.98250000000002, 230.01000000000002, 230.03750000000002, 230.06500000000003, 230.09250000000003, 230.12000000000003, 230.14750000000004, 230.17500000000004, 230.20250000000004, 230.23000000000002, 230.25750000000002, 230.28500000000003, 230.31250000000003, 230.34000000000003, 230.36750000000004, 230.39500000000004, 230.42250000000004, 230.45000000000005, 230.47750000000002, 230.50500000000002, 230.53250000000003, 230.56000000000003, 230.58750000000003, 230.61500000000004, 230.64250000000004, 230.67000000000002, 230.69750000000002, 230.72500000000002, 230.75250000000003, 230.78000000000003, 230.80750000000003, 230.83500000000004, 230.86250000000004, 230.89000000000004, 230.91750000000002, 230.94500000000002, 230.97250000000003, 231.00000000000003, 231.02750000000003, 231.05500000000004, 231.08250000000004, 231.11000000000004, 231.13750000000005, 231.16500000000002, 231.19250000000002, 231.22000000000003, 231.24750000000003, 231.27500000000003, 231.30250000000004, 231.33000000000004, |

| Description | Value |
| --- | --- |
| | 231.35750000000002, 231.38500000000002, 231.41250000000002, 231.44000000000003, 231.46750000000003, 231.49500000000003, 231.52250000000004, 231.55000000000004, 231.57750000000004, 231.60500000000002, 231.63250000000002, 231.66000000000003, 231.68750000000003, 231.71500000000003, 231.74250000000004, 231.77000000000004, 231.79750000000004, 231.82500000000005, 231.85250000000002, 231.88000000000002, 231.90750000000003, 231.93500000000003, 231.96250000000003, 231.99000000000004, 232.01750000000004, 232.04500000000002, 232.07250000000002, 232.10000000000002, 232.12750000000003, 232.15500000000003, 232.18250000000003, 232.21000000000004, 232.23750000000004, 232.26500000000004, 232.29250000000002, 232.32000000000002, 232.34750000000003, 232.37500000000003, 232.40250000000003, 232.43000000000004, 232.45750000000004, 232.48500000000004, 232.51250000000005, 232.54000000000002, 232.56750000000002, 232.59500000000003, 232.62250000000003, 232.65000000000003, 232.67750000000004, 232.70500000000004, 232.73250000000002, 232.76000000000002, 232.78750000000002, 232.81500000000003, 232.84250000000003, 232.87000000000003, 232.89750000000004, 232.92500000000004, 232.95250000000004, 232.98000000000002, 233.00750000000002, 233.03500000000003, 233.06250000000003, 233.09000000000003, 233.11750000000004, 233.14500000000004, 233.17250000000004, 233.20000000000005, 233.22750000000002, 233.25500000000002, 233.28250000000003, 233.31000000000003, 233.33750000000003, 233.36500000000004, 233.39250000000004, 233.42000000000002, 233.44750000000002, 233.47500000000002, 233.50250000000003, 233.53000000000003, 233.55750000000003, 233.58500000000004, 233.61250000000004, 233.64000000000004, 233.66750000000002, 233.69500000000002, 233.72250000000003, 233.75000000000003, 233.77750000000003, 233.80500000000004, 233.83250000000004, 233.86000000000004, 233.88750000000005, 233.91500000000002, 233.94250000000002, 233.97000000000003, 233.99750000000003, 234.02500000000003, 234.05250000000004, 234.08000000000004, 234.10750000000002, 234.13500000000002, 234.16250000000002, 234.19000000000003, 234.21750000000003, 234.24500000000003, 234.27250000000004, 234.30000000000004, 234.32750000000004, 234.35500000000002, 234.38250000000002, 234.41000000000003, 234.43750000000003, 234.46500000000003, 234.49250000000004, 234.52000000000004, 234.54750000000004, 234.57500000000005, 234.60250000000002, 234.63000000000002, 234.65750000000003, 234.68500000000003, 234.71250000000003, 234.74000000000004, 234.76750000000004, 234.79500000000002, 234.82250000000005, 234.85000000000002, 234.87750000000003, 234.90500000000003, 234.93250000000003, 234.96000000000004, 234.98750000000004, 235.01500000000004, 235.04250000000002, 235.07000000000002, 235.09750000000003, 235.12500000000003, 235.15250000000003, 235.18000000000004, 235.20750000000004, 235.23500000000004, 235.26250000000005, 235.29000000000002, |

| Description | Value |
| --- | --- |
| | 235.31750000000002, 235.34500000000003, 235.37250000000003, 235.40000000000003, 235.42750000000004, 235.45500000000004, 235.48250000000002, 235.51000000000005, 235.53750000000002, 235.56500000000003, 235.59250000000003, 235.62000000000003, 235.64750000000004, 235.67500000000004, 235.70250000000004, 235.73000000000002, 235.75750000000002, 235.78500000000003, 235.81250000000003, 235.84000000000003, 235.86750000000004, 235.89500000000004, 235.92250000000004, 235.95000000000005, 235.97750000000002, 236.00500000000002, 236.03250000000003, 236.06000000000003, 236.08750000000003, 236.11500000000004, 236.14250000000004, 236.17000000000004, 236.19750000000005, 236.22500000000002, 236.25250000000003, 236.28000000000003, 236.30750000000003, 236.33500000000004, 236.36250000000004, 236.39000000000004, 236.41750000000002, 236.44500000000002, 236.47250000000003, 236.50000000000003, 236.52750000000003, 236.55500000000004, 236.58250000000004, 236.61000000000004, 236.63750000000005, 236.66500000000002, 236.69250000000002, 236.72000000000003, 236.74750000000003, 236.77500000000003, 236.80250000000004, 236.83000000000004, 236.85750000000004, 236.88500000000005, 236.91250000000002, 236.94000000000003, 236.96750000000003, 236.99500000000003, 237.02250000000004, 237.05000000000004, 237.07750000000004, 237.10500000000002, 237.13250000000002, 237.16000000000003, 237.18750000000003, 237.21500000000003, 237.24250000000004, 237.27000000000004, 237.29750000000004, 237.32500000000005, 237.35250000000002, 237.38000000000002, 237.40750000000003, 237.43500000000003, 237.46250000000003, 237.49000000000004, 237.51750000000004, 237.54500000000004, 237.57250000000005, 237.60000000000002, 237.62750000000003, 237.65500000000003, 237.68250000000003, 237.71000000000004, 237.73750000000004, 237.76500000000004, 237.79250000000002, 237.82000000000002, 237.84750000000003, 237.87500000000003, 237.90250000000003, 237.93000000000004, 237.95750000000004, 237.98500000000004, 238.01250000000005, 238.04000000000002, 238.06750000000002, 238.09500000000003, 238.12250000000003, 238.15000000000003, 238.17750000000004, 238.20500000000004, 238.23250000000004, 238.26000000000005, 238.28750000000002, 238.31500000000003, 238.34250000000003, 238.37000000000003, 238.39750000000004, 238.42500000000004, 238.45250000000004, 238.48000000000002, 238.50750000000002, 238.53500000000003, 238.56250000000003, 238.59000000000003, 238.61750000000004, 238.64500000000004, 238.67250000000004, 238.70000000000005, 238.72750000000002, 238.75500000000002, 238.78250000000003, 238.81000000000003, 238.83750000000003, 238.86500000000004, 238.89250000000004, 238.92000000000004, 238.94750000000005, 238.97500000000002, 239.00250000000003, 239.03000000000003, 239.05750000000003, 239.08500000000004, 239.11250000000004, 239.14000000000004, 239.16750000000002, 239.19500000000002, 239.22250000000003, 239.25000000000003, |

| Description | Value |
| --- | --- |
| | 239.27750000000003, 239.30500000000004, 239.33250000000004, 239.36000000000004, 239.38750000000005, 239.41500000000002, 239.44250000000002, 239.47000000000003, 239.49750000000003, 239.52500000000003, 239.55250000000004, 239.58000000000004, 239.60750000000004, 239.63500000000005, 239.66250000000002, 239.69000000000003, 239.71750000000003, 239.74500000000003, 239.77250000000004, 239.80000000000004, 239.82750000000004, 239.85500000000002, 239.88250000000002, 239.91000000000003, 239.93750000000003, 239.96500000000003, 239.99250000000004, 240.02000000000004, 240.04750000000004, 240.07500000000005, 240.10250000000002, 240.13000000000002, 240.15750000000003, 240.18500000000003, 240.21250000000003, 240.24000000000004, 240.26750000000004, 240.29500000000004, 240.32250000000005, 240.35000000000002, 240.37750000000003, 240.40500000000003, 240.43250000000003, 240.46000000000004, 240.48750000000004, 240.51500000000004, 240.54250000000002, 240.57000000000002, 240.59750000000003, 240.62500000000003, 240.65250000000003, 240.68000000000004, 240.70750000000004, 240.73500000000004, 240.76250000000005, 240.79000000000002, 240.81750000000002, 240.84500000000003, 240.87250000000003, 240.90000000000003, 240.92750000000004, 240.95500000000004, 240.98250000000004, 241.01000000000005, 241.03750000000002, 241.06500000000003, 241.09250000000003, 241.12000000000003, 241.14750000000004, 241.17500000000004, 241.20250000000004, 241.23000000000002, 241.25750000000005, 241.28500000000003, 241.31250000000003, 241.34000000000003, 241.36750000000004, 241.39500000000004, 241.42250000000004, 241.45000000000005, 241.47750000000002, 241.50500000000002, 241.53250000000003, 241.56000000000003, 241.58750000000003, 241.61500000000004, 241.64250000000004, 241.67000000000002, 241.69750000000005, 241.72500000000002, 241.75250000000003, 241.78000000000003, 241.80750000000003, 241.83500000000004, 241.86250000000004, 241.89000000000004, 241.91750000000002, 241.94500000000005, 241.97250000000003, 242.00000000000003} |

### VALUES OF DEPENDENT VARIABLES

| Description | Value |
| --- | --- |
| Settings | User controlled |
| Method | Solution |
| Study | Study 4 (CV 31 to 38) |
| Settings | User controlled |
| Method | Solution |
| Study | Study 4 (CV 31 to 38) |

### MESH

| Feature | Value |
|---|---|
| Geometry 1 | mesh1 |

#### PHYSICS AND VARIABLES SELECTION

| Physics interface | Discretization |
|---|---|
| Transport of Diluted Species in droplet (tds) | physics |
| Moving Mesh (ale) | physics |

#### MESH SELECTION

| Geometry | Mesh |
|---|---|
| Geometry 1 (geom1) | mesh1 |

## 7.2 SOLVER CONFIGURATIONS

### 7.2.1 Solution 10

**Compile Equations: Time Dependent (st1)**

#### STUDY AND STEP

| Description | Value |
|---|---|
| Use study | Study 5 (CV 39 to 44) |
| Use study step | Time Dependent |

#### LOG

```
<---- Compile Equations: Time Dependent in Study 5 (CV 39 to 44)/Solution 10
      (sol10) ------------------------------------------------------------------
Started at Jun 2, 2023 10:22:24 AM.
Time: 0 s.
Physical memory: 3.36 GB
Virtual memory: 3.21 GB
Ended at Jun 2, 2023 10:22:25 AM.
----- Compile Equations: Time Dependent in Study 5 (CV 39 to 44)/Solution 10
      (sol10) ------------------------------------------------------------------>
```

**Dependent Variables 1 (v1)**

#### GENERAL

| Description | Value |
|---|---|
| Defined by study step | Time Dependent |

#### INITIAL VALUES OF VARIABLES SOLVED FOR

| Description | Value |
|---|---|
| Method | Solution |
| Solution | Remeshed Solution 4 |

### RESIDUAL SCALING

| Description | Value |
| --- | --- |
| Method | Manual |

### VALUES OF VARIABLES NOT SOLVED FOR

| Description | Value |
| --- | --- |
| Method | Solution |
| Solution | Remeshed Solution 4 |

### INITIAL VALUE CALCULATION CONSTANTS

| Constant name | Initial value source |
| --- | --- |
| t | range(38*t_cv,t_tot/nb/200,44*t_cv) |
| timestep | 0.033[s] |

### LOG

```
<---- Dependent Variables 1 in Study 5 (CV 39 to 43)/Solution 10 (sol10) -------
Started at May 31, 2023 9:33:23 PM.
Initial values of variables solved for: Remeshed Solution 4 (sol9), t=209 s [Last].
Values of variables not solved for: Remeshed Solution 4 (sol9), t=209 s [Last].
Solution time: 1 s.
Physical memory: 2.06 GB
Virtual memory: 2.21 GB
Ended at May 31, 2023 9:33:24 PM.
----- Dependent Variables 1 in Study 5 (CV 39 to 43)/Solution 10 (sol10) ------>
```

#### Concentration (comp1.cOx) (comp1_cOx)

### GENERAL

| Description | Value |
| --- | --- |
| Field components | comp1.cOx |
| Internal variables | {comp1.uflux.cOx, comp1.dflux.cOx} |

#### Concentration (comp1.cRed) (comp1_cRed)

### GENERAL

| Description | Value |
| --- | --- |
| Field components | comp1.cRed |
| Internal variables | {comp1.uflux.cRed, comp1.dflux.cRed} |

#### comp1.comp1.RgZg (comp1_comp1_RgZg)

### GENERAL

| Description | Value |
| --- | --- |
| Field components | {Rg, Zg} |
| Solve for this field | Off |

**Spatial mesh displacement (comp1.spatial.disp) (comp1_spatial_disp)**

GENERAL

| Description | Value |
| --- | --- |
| Field components | {comp1.spatial.u, comp1.spatial.w} |

SCALING

| Description | Value |
| --- | --- |
| Method | Manual |
| Scale | 6.86772451398569E-8 |

**Time-Dependent Solver 1 (t1)**

GENERAL

| Description | Value |
| --- | --- |
| Defined by study step | Time Dependent |
| Output times | {209.00000000000003, 209.02750000000003, 209.05500000000004, 209.08250000000004, 209.11000000000004, 209.13750000000002, 209.16500000000002, 209.19250000000002, 209.22000000000003, 209.24750000000003, 209.27500000000003, 209.30250000000004, 209.33000000000004, 209.35750000000002, 209.38500000000002, 209.41250000000002, 209.44000000000003, 209.46750000000003, 209.49500000000003, 209.52250000000004, 209.55000000000004, 209.57750000000001, 209.60500000000002, 209.63250000000002, 209.66000000000003, 209.68750000000003, 209.71500000000003, 209.74250000000004, 209.77000000000004, 209.79750000000004, 209.82500000000002, 209.85250000000002, 209.88000000000002, 209.90750000000003, 209.93500000000003, 209.96250000000003, 209.99000000000004, 210.01750000000004, 210.04500000000002, 210.07250000000002, 210.10000000000002, 210.12750000000003, 210.15500000000003, 210.18250000000003, 210.21000000000004, 210.23750000000004, 210.26500000000001, 210.29250000000002, 210.32000000000002, 210.34750000000003, 210.37500000000003, 210.40250000000003, 210.43000000000004, 210.45750000000004, 210.48500000000004, 210.51250000000002, 210.54000000000002, 210.56750000000002, 210.59500000000003, 210.62250000000003, 210.65000000000003, 210.67750000000004, 210.70500000000004, 210.73250000000002, 210.76000000000002, 210.78750000000002, 210.81500000000003, 210.84250000000003, 210.87000000000003, 210.89750000000004, 210.92500000000004, 210.95250000000001, 210.98000000000002, 211.00750000000002, 211.03500000000003, 211.06250000000003, 211.09000000000003, 211.11750000000004, 211.14500000000004, 211.17250000000004, 211.20000000000002, 211.22750000000002, 211.25500000000002, 211.28250000000003, 211.31000000000003, 211.33750000000003, 211.36500000000004, 211.39250000000004, 211.42000000000002, 211.44750000000002, 211.47500000000002, 211.50250000000003, 211.53000000000003, |

| Description | Value |
| --- | --- |
| | 211.55750000000003, 211.58500000000004, 211.61250000000004, 211.64000000000004, 211.66750000000002, 211.69500000000002, 211.72250000000003, 211.75000000000003, 211.77750000000003, 211.80500000000004, 211.83250000000004, 211.86000000000004, 211.88750000000002, 211.91500000000002, 211.94250000000002, 211.97000000000003, 211.99750000000003, 212.02500000000003, 212.05250000000004, 212.08000000000004, 212.10750000000002, 212.13500000000002, 212.16250000000002, 212.19000000000003, 212.21750000000003, 212.24500000000003, 212.27250000000004, 212.30000000000004, 212.32750000000004, 212.35500000000002, 212.38250000000002, 212.41000000000003, 212.43750000000003, 212.46500000000003, 212.49250000000004, 212.52000000000004, 212.54750000000004, 212.57500000000002, 212.60250000000002, 212.63000000000002, 212.65750000000003, 212.68500000000003, 212.71250000000003, 212.74000000000004, 212.76750000000004, 212.79500000000002, 212.82250000000002, 212.85000000000002, 212.87750000000003, 212.90500000000003, 212.93250000000003, 212.96000000000004, 212.98750000000004, 213.01500000000004, 213.04250000000002, 213.07000000000002, 213.09750000000003, 213.12500000000003, 213.15250000000003, 213.18000000000004, 213.20750000000004, 213.23500000000004, 213.26250000000002, 213.29000000000002, 213.31750000000002, 213.34500000000003, 213.37250000000003, 213.40000000000003, 213.42750000000004, 213.45500000000004, 213.48250000000002, 213.51000000000002, 213.53750000000002, 213.56500000000003, 213.59250000000003, 213.62000000000003, 213.64750000000004, 213.67500000000004, 213.70250000000004, 213.73000000000002, 213.75750000000002, 213.78500000000003, 213.81250000000003, 213.84000000000003, 213.86750000000004, 213.89500000000004, 213.92250000000004, 213.95000000000002, 213.97750000000002, 214.00500000000002, 214.03250000000003, 214.06000000000003, 214.08750000000003, 214.11500000000004, 214.14250000000004, 214.17000000000002, 214.19750000000002, 214.22500000000002, 214.25250000000003, 214.28000000000003, 214.30750000000003, 214.33500000000004, 214.36250000000004, 214.39000000000004, 214.41750000000002, 214.44500000000002, 214.47250000000003, 214.50000000000003, 214.52750000000003, 214.55500000000004, 214.58250000000004, 214.61000000000004, 214.63750000000002, 214.66500000000002, 214.69250000000002, 214.72000000000003, 214.74750000000003, 214.77500000000003, 214.80250000000004, 214.83000000000004, 214.85750000000002, 214.88500000000002, 214.91250000000002, 214.94000000000003, 214.96750000000003, 214.99500000000003, 215.02250000000004, 215.05000000000004, 215.07750000000004, 215.10500000000002, 215.13250000000002, 215.16000000000003, 215.18750000000003, 215.21500000000003, 215.24250000000004, 215.27000000000004, 215.29750000000004, 215.32500000000002, 215.35250000000002, 215.38000000000002, 215.40750000000003, 215.43500000000003, 215.46250000000003, 215.49000000000004, |

| Description | Value |
| --- | --- |
| | 215.51750000000004, 215.54500000000002, 215.57250000000002, 215.60000000000002, 215.62750000000003, 215.65500000000003, 215.68250000000003, 215.71000000000004, 215.73750000000004, 215.76500000000004, 215.79250000000002, 215.82000000000002, 215.84750000000003, 215.87500000000003, 215.90250000000003, 215.93000000000004, 215.95750000000004, 215.98500000000004, 216.01250000000002, 216.04000000000002, 216.06750000000002, 216.09500000000003, 216.12250000000003, 216.15000000000003, 216.17750000000004, 216.20500000000004, 216.23250000000002, 216.26000000000002, 216.28750000000002, 216.31500000000003, 216.34250000000003, 216.37000000000003, 216.39750000000004, 216.42500000000004, 216.45250000000004, 216.48000000000002, 216.50750000000002, 216.53500000000003, 216.56250000000003, 216.59000000000003, 216.61750000000004, 216.64500000000004, 216.67250000000004, 216.70000000000002, 216.72750000000002, 216.75500000000002, 216.78250000000003, 216.81000000000003, 216.83750000000003, 216.86500000000004, 216.89250000000004, 216.92000000000002, 216.94750000000002, 216.97500000000002, 217.00250000000003, 217.03000000000003, 217.05750000000003, 217.08500000000004, 217.11250000000004, 217.14000000000004, 217.16750000000002, 217.19500000000002, 217.22250000000003, 217.25000000000003, 217.27750000000003, 217.30500000000004, 217.33250000000004, 217.36000000000004, 217.38750000000002, 217.41500000000002, 217.44250000000002, 217.47000000000003, 217.49750000000003, 217.52500000000003, 217.55250000000004, 217.58000000000004, 217.60750000000002, 217.63500000000002, 217.66250000000002, 217.69000000000003, 217.71750000000003, 217.74500000000003, 217.77250000000004, 217.80000000000004, 217.82750000000004, 217.85500000000002, 217.88250000000002, 217.91000000000003, 217.93750000000003, 217.96500000000003, 217.99250000000004, 218.02000000000004, 218.04750000000004, 218.07500000000002, 218.10250000000002, 218.13000000000002, 218.15750000000003, 218.18500000000003, 218.21250000000003, 218.24000000000004, 218.26750000000004, 218.29500000000002, 218.32250000000002, 218.35000000000002, 218.37750000000003, 218.40500000000003, 218.43250000000003, 218.46000000000004, 218.48750000000004, 218.51500000000004, 218.54250000000002, 218.57000000000002, 218.59750000000003, 218.62500000000003, 218.65250000000003, 218.68000000000004, 218.70750000000004, 218.73500000000004, 218.76250000000002, 218.79000000000002, 218.81750000000002, 218.84500000000003, 218.87250000000003, 218.90000000000003, 218.92750000000004, 218.95500000000004, 218.98250000000002, 219.01000000000002, 219.03750000000002, 219.06500000000003, 219.09250000000003, 219.12000000000003, 219.14750000000004, 219.17500000000004, 219.20250000000004, 219.23000000000002, 219.25750000000002, 219.28500000000003, 219.31250000000003, 219.34000000000003, 219.36750000000004, 219.39500000000004, 219.42250000000004, 219.45000000000002, |

| Description | Value |
|---|---|
| | 219.47750000000002, 219.50500000000002, 219.53250000000003, 219.56000000000003, 219.58750000000003, 219.61500000000004, 219.64250000000004, 219.67000000000002, 219.69750000000002, 219.72500000000002, 219.75250000000003, 219.78000000000003, 219.80750000000003, 219.83500000000004, 219.86250000000004, 219.89000000000004, 219.91750000000002, 219.94500000000002, 219.97250000000003, 220.00000000000003, 220.02750000000003, 220.05500000000004, 220.08250000000004, 220.11000000000004, 220.13750000000002, 220.16500000000002, 220.19250000000002, 220.22000000000003, 220.24750000000003, 220.27500000000003, 220.30250000000004, 220.33000000000004, 220.35750000000002, 220.38500000000002, 220.41250000000002, 220.44000000000003, 220.46750000000003, 220.49500000000003, 220.52250000000004, 220.55000000000004, 220.57750000000004, 220.60500000000002, 220.63250000000002, 220.66000000000003, 220.68750000000003, 220.71500000000003, 220.74250000000004, 220.77000000000004, 220.79750000000004, 220.82500000000002, 220.85250000000002, 220.88000000000002, 220.90750000000003, 220.93500000000003, 220.96250000000003, 220.99000000000004, 221.01750000000004, 221.04500000000002, 221.07250000000002, 221.10000000000002, 221.12750000000003, 221.15500000000003, 221.18250000000003, 221.21000000000004, 221.23750000000004, 221.26500000000004, 221.29250000000002, 221.32000000000002, 221.34750000000003, 221.37500000000003, 221.40250000000003, 221.43000000000004, 221.45750000000004, 221.48500000000004, 221.51250000000002, 221.54000000000002, 221.56750000000002, 221.59500000000003, 221.62250000000003, 221.65000000000003, 221.67750000000004, 221.70500000000004, 221.73250000000002, 221.76000000000002, 221.78750000000002, 221.81500000000003, 221.84250000000003, 221.87000000000003, 221.89750000000004, 221.92500000000004, 221.95250000000004, 221.98000000000002, 222.00750000000002, 222.03500000000003, 222.06250000000003, 222.09000000000003, 222.11750000000004, 222.14500000000004, 222.17250000000004, 222.20000000000002, 222.22750000000002, 222.25500000000002, 222.28250000000003, 222.31000000000003, 222.33750000000003, 222.36500000000004, 222.39250000000004, 222.42000000000002, 222.44750000000002, 222.47500000000002, 222.50250000000003, 222.53000000000003, 222.55750000000003, 222.58500000000004, 222.61250000000004, 222.64000000000004, 222.66750000000002, 222.69500000000002, 222.72250000000003, 222.75000000000003, 222.77750000000003, 222.80500000000004, 222.83250000000004, 222.86000000000004, 222.88750000000002, 222.91500000000002, 222.94250000000002, 222.97000000000003, 222.99750000000003, 223.02500000000003, 223.05250000000004, 223.08000000000004, 223.10750000000002, 223.13500000000002, 223.16250000000002, 223.19000000000003, 223.21750000000003, 223.24500000000003, 223.27250000000004, 223.30000000000004, 223.32750000000004, 223.35500000000002, 223.38250000000002, 223.41000000000003, |

| Description | Value |
| --- | --- |
| | 223.43750000000003, 223.46500000000003, 223.49250000000004, 223.52000000000004, 223.54750000000004, 223.57500000000002, 223.60250000000002, 223.63000000000002, 223.65750000000003, 223.68500000000003, 223.71250000000003, 223.74000000000004, 223.76750000000004, 223.79500000000002, 223.82250000000002, 223.85000000000002, 223.87750000000003, 223.90500000000003, 223.93250000000003, 223.96000000000004, 223.98750000000004, 224.01500000000004, 224.04250000000002, 224.07000000000002, 224.09750000000003, 224.12500000000003, 224.15250000000003, 224.18000000000004, 224.20750000000004, 224.23500000000004, 224.26250000000005, 224.29000000000002, 224.31750000000002, 224.34500000000003, 224.37250000000003, 224.40000000000003, 224.42750000000004, 224.45500000000004, 224.48250000000002, 224.51000000000002, 224.53750000000002, 224.56500000000003, 224.59250000000003, 224.62000000000003, 224.64750000000004, 224.67500000000004, 224.70250000000004, 224.73000000000002, 224.75750000000002, 224.78500000000003, 224.81250000000003, 224.84000000000003, 224.86750000000004, 224.89500000000004, 224.92250000000004, 224.95000000000005, 224.97750000000002, 225.00500000000002, 225.03250000000003, 225.06000000000003, 225.08750000000003, 225.11500000000004, 225.14250000000004, 225.17000000000002, 225.19750000000002, 225.22500000000002, 225.25250000000003, 225.28000000000003, 225.30750000000003, 225.33500000000004, 225.36250000000004, 225.39000000000004, 225.41750000000002, 225.44500000000002, 225.47250000000003, 225.50000000000003, 225.52750000000003, 225.55500000000004, 225.58250000000004, 225.61000000000004, 225.63750000000005, 225.66500000000002, 225.69250000000002, 225.72000000000003, 225.74750000000003, 225.77500000000003, 225.80250000000004, 225.83000000000004, 225.85750000000002, 225.88500000000002, 225.91250000000002, 225.94000000000003, 225.96750000000003, 225.99500000000003, 226.02250000000004, 226.05000000000004, 226.07750000000004, 226.10500000000002, 226.13250000000002, 226.16000000000003, 226.18750000000003, 226.21500000000003, 226.24250000000004, 226.27000000000004, 226.29750000000004, 226.32500000000005, 226.35250000000002, 226.38000000000002, 226.40750000000003, 226.43500000000003, 226.46250000000003, 226.49000000000004, 226.51750000000004, 226.54500000000002, 226.57250000000002, 226.60000000000002, 226.62750000000003, 226.65500000000003, 226.68250000000003, 226.71000000000004, 226.73750000000004, 226.76500000000004, 226.79250000000002, 226.82000000000002, 226.84750000000003, 226.87500000000003, 226.90250000000003, 226.93000000000004, 226.95750000000004, 226.98500000000004, 227.01250000000005, 227.04000000000002, 227.06750000000002, 227.09500000000003, 227.12250000000003, 227.15000000000003, 227.17750000000004, 227.20500000000004, 227.23250000000002, 227.26000000000002, 227.28750000000002, 227.31500000000003, 227.34250000000003, 227.37000000000003, |

| Description | Value |
|---|---|
| | 227.39750000000004, 227.42500000000004, 227.45250000000004, 227.48000000000002, 227.50750000000002, 227.53500000000003, 227.56250000000003, 227.59000000000003, 227.61750000000004, 227.64500000000004, 227.67250000000004, 227.70000000000005, 227.72750000000002, 227.75500000000002, 227.78250000000003, 227.81000000000003, 227.83750000000003, 227.86500000000004, 227.89250000000004, 227.92000000000002, 227.94750000000002, 227.97500000000002, 228.00250000000003, 228.03000000000003, 228.05750000000003, 228.08500000000004, 228.11250000000004, 228.14000000000004, 228.16750000000002, 228.19500000000002, 228.22250000000003, 228.25000000000003, 228.27750000000003, 228.30500000000004, 228.33250000000004, 228.36000000000004, 228.38750000000005, 228.41500000000002, 228.44250000000002, 228.47000000000003, 228.49750000000003, 228.52500000000003, 228.55250000000004, 228.58000000000004, 228.60750000000002, 228.63500000000002, 228.66250000000002, 228.69000000000003, 228.71750000000003, 228.74500000000003, 228.77250000000004, 228.80000000000004, 228.82750000000004, 228.85500000000002, 228.88250000000002, 228.91000000000003, 228.93750000000003, 228.96500000000003, 228.99250000000004, 229.02000000000004, 229.04750000000004, 229.07500000000005, 229.10250000000002, 229.13000000000002, 229.15750000000003, 229.18500000000003, 229.21250000000003, 229.24000000000004, 229.26750000000004, 229.29500000000002, 229.32250000000002, 229.35000000000002, 229.37750000000003, 229.40500000000003, 229.43250000000003, 229.46000000000004, 229.48750000000004, 229.51500000000004, 229.54250000000002, 229.57000000000002, 229.59750000000003, 229.62500000000003, 229.65250000000003, 229.68000000000004, 229.70750000000004, 229.73500000000004, 229.76250000000005, 229.79000000000002, 229.81750000000002, 229.84500000000003, 229.87250000000003, 229.90000000000003, 229.92750000000004, 229.95500000000004, 229.98250000000002, 230.01000000000002, 230.03750000000002, 230.06500000000003, 230.09250000000003, 230.12000000000003, 230.14750000000004, 230.17500000000004, 230.20250000000004, 230.23000000000002, 230.25750000000002, 230.28500000000003, 230.31250000000003, 230.34000000000003, 230.36750000000004, 230.39500000000004, 230.42250000000004, 230.45000000000005, 230.47750000000002, 230.50500000000002, 230.53250000000003, 230.56000000000003, 230.58750000000003, 230.61500000000004, 230.64250000000004, 230.67000000000002, 230.69750000000002, 230.72500000000002, 230.75250000000003, 230.78000000000003, 230.80750000000003, 230.83500000000004, 230.86250000000004, 230.89000000000004, 230.91750000000002, 230.94500000000002, 230.97250000000003, 231.00000000000003, 231.02750000000003, 231.05500000000004, 231.08250000000004, 231.11000000000004, 231.13750000000005, 231.16500000000002, 231.19250000000002, 231.22000000000003, 231.24750000000003, 231.27500000000003, 231.30250000000004, 231.33000000000004, |

| Description | Value |
| --- | --- |
| | 231.35750000000002, 231.38500000000002, 231.41250000000002, 231.44000000000003, 231.46750000000003, 231.49500000000003, 231.52250000000004, 231.55000000000004, 231.57750000000004, 231.60500000000002, 231.63250000000002, 231.66000000000003, 231.68750000000003, 231.71500000000003, 231.74250000000004, 231.77000000000004, 231.79750000000004, 231.82500000000005, 231.85250000000002, 231.88000000000002, 231.90750000000003, 231.93500000000003, 231.96250000000003, 231.99000000000004, 232.01750000000004, 232.04500000000002, 232.07250000000002, 232.10000000000002, 232.12750000000003, 232.15500000000003, 232.18250000000003, 232.21000000000004, 232.23750000000004, 232.26500000000004, 232.29250000000002, 232.32000000000002, 232.34750000000003, 232.37500000000003, 232.40250000000003, 232.43000000000004, 232.45750000000004, 232.48500000000004, 232.51250000000005, 232.54000000000002, 232.56750000000002, 232.59500000000003, 232.62250000000003, 232.65000000000003, 232.67750000000004, 232.70500000000004, 232.73250000000002, 232.76000000000002, 232.78750000000002, 232.81500000000003, 232.84250000000003, 232.87000000000003, 232.89750000000004, 232.92500000000004, 232.95250000000004, 232.98000000000002, 233.00750000000002, 233.03500000000003, 233.06250000000003, 233.09000000000003, 233.11750000000004, 233.14500000000004, 233.17250000000004, 233.20000000000005, 233.22750000000002, 233.25500000000002, 233.28250000000003, 233.31000000000003, 233.33750000000003, 233.36500000000004, 233.39250000000004, 233.42000000000002, 233.44750000000002, 233.47500000000002, 233.50250000000003, 233.53000000000003, 233.55750000000003, 233.58500000000004, 233.61250000000004, 233.64000000000004, 233.66750000000002, 233.69500000000002, 233.72250000000003, 233.75000000000003, 233.77750000000003, 233.80500000000004, 233.83250000000004, 233.86000000000004, 233.88750000000005, 233.91500000000002, 233.94250000000002, 233.97000000000003, 233.99750000000003, 234.02500000000003, 234.05250000000004, 234.08000000000004, 234.10750000000002, 234.13500000000002, 234.16250000000002, 234.19000000000003, 234.21750000000003, 234.24500000000003, 234.27250000000004, 234.30000000000004, 234.32750000000004, 234.35500000000002, 234.38250000000002, 234.41000000000003, 234.43750000000003, 234.46500000000003, 234.49250000000004, 234.52000000000004, 234.54750000000004, 234.57500000000005, 234.60250000000002, 234.63000000000002, 234.65750000000003, 234.68500000000003, 234.71250000000003, 234.74000000000004, 234.76750000000004, 234.79500000000002, 234.82250000000005, 234.85000000000002, 234.87750000000003, 234.90500000000003, 234.93250000000003, 234.96000000000004, 234.98750000000004, 235.01500000000004, 235.04250000000002, 235.07000000000002, 235.09750000000003, 235.12500000000003, 235.15250000000003, 235.18000000000004, 235.20750000000004, 235.23500000000004, 235.26250000000005, 235.29000000000002, |

| Description | Value |
|---|---|
| | 235.31750000000002, 235.34500000000003, 235.37250000000003, 235.40000000000003, 235.42750000000004, 235.45500000000004, 235.48250000000002, 235.51000000000005, 235.53750000000002, 235.56500000000003, 235.59250000000003, 235.62000000000003, 235.64750000000004, 235.67500000000004, 235.70250000000004, 235.73000000000002, 235.75750000000002, 235.78500000000003, 235.81250000000003, 235.84000000000003, 235.86750000000004, 235.89500000000004, 235.92250000000004, 235.95000000000005, 235.97750000000002, 236.00500000000002, 236.03250000000003, 236.06000000000003, 236.08750000000003, 236.11500000000004, 236.14250000000004, 236.17000000000004, 236.19750000000005, 236.22500000000002, 236.25250000000003, 236.28000000000003, 236.30750000000003, 236.33500000000004, 236.36250000000004, 236.39000000000004, 236.41750000000002, 236.44500000000002, 236.47250000000003, 236.50000000000003, 236.52750000000003, 236.55500000000004, 236.58250000000004, 236.61000000000004, 236.63750000000005, 236.66500000000002, 236.69250000000002, 236.72000000000003, 236.74750000000003, 236.77500000000003, 236.80250000000004, 236.83000000000004, 236.85750000000004, 236.88500000000005, 236.91250000000002, 236.94000000000003, 236.96750000000003, 236.99500000000003, 237.02250000000004, 237.05000000000004, 237.07750000000004, 237.10500000000002, 237.13250000000002, 237.16000000000003, 237.18750000000003, 237.21500000000003, 237.24250000000004, 237.27000000000004, 237.29750000000004, 237.32500000000005, 237.35250000000002, 237.38000000000002, 237.40750000000003, 237.43500000000003, 237.46250000000003, 237.49000000000004, 237.51750000000004, 237.54500000000004, 237.57250000000005, 237.60000000000002, 237.62750000000003, 237.65500000000003, 237.68250000000003, 237.71000000000004, 237.73750000000004, 237.76500000000004, 237.79250000000002, 237.82000000000002, 237.84750000000003, 237.87500000000003, 237.90250000000003, 237.93000000000004, 237.95750000000004, 237.98500000000004, 238.01250000000005, 238.04000000000002, 238.06750000000002, 238.09500000000003, 238.12250000000003, 238.15000000000003, 238.17750000000004, 238.20500000000004, 238.23250000000004, 238.26000000000005, 238.28750000000002, 238.31500000000003, 238.34250000000003, 238.37000000000003, 238.39750000000004, 238.42500000000004, 238.45250000000004, 238.48000000000002, 238.50750000000002, 238.53500000000003, 238.56250000000003, 238.59000000000003, 238.61750000000004, 238.64500000000004, 238.67250000000004, 238.70000000000005, 238.72750000000002, 238.75500000000002, 238.78250000000003, 238.81000000000003, 238.83750000000003, 238.86500000000004, 238.89250000000004, 238.92000000000004, 238.94750000000005, 238.97500000000002, 239.00250000000003, 239.03000000000003, 239.05750000000003, 239.08500000000004, 239.11250000000004, 239.14000000000004, 239.16750000000002, 239.19500000000002, 239.22250000000003, 239.25000000000003, |

| Description | Value |
|---|---|
| | 239.27750000000003, 239.30500000000004, 239.33250000000004, 239.36000000000004, 239.38750000000005, 239.41500000000002, 239.44250000000002, 239.47000000000003, 239.49750000000003, 239.52500000000003, 239.55250000000004, 239.58000000000004, 239.60750000000004, 239.63500000000005, 239.66250000000002, 239.69000000000003, 239.71750000000003, 239.74500000000003, 239.77250000000004, 239.80000000000004, 239.82750000000004, 239.85500000000002, 239.88250000000002, 239.91000000000003, 239.93750000000003, 239.96500000000003, 239.99250000000004, 240.02000000000004, 240.04750000000004, 240.07500000000005, 240.10250000000002, 240.13000000000002, 240.15750000000003, 240.18500000000003, 240.21250000000003, 240.24000000000004, 240.26750000000004, 240.29500000000004, 240.32250000000005, 240.35000000000002, 240.37750000000003, 240.40500000000003, 240.43250000000003, 240.46000000000004, 240.48750000000004, 240.51500000000004, 240.54250000000002, 240.57000000000002, 240.59750000000003, 240.62500000000003, 240.65250000000003, 240.68000000000004, 240.70750000000004, 240.73500000000004, 240.76250000000005, 240.79000000000002, 240.81750000000002, 240.84500000000003, 240.87250000000003, 240.90000000000003, 240.92750000000004, 240.95500000000004, 240.98250000000004, 241.01000000000005, 241.03750000000002, 241.06500000000003, 241.09250000000003, 241.12000000000003, 241.14750000000004, 241.17500000000004, 241.20250000000004, 241.23000000000002, 241.25750000000005, 241.28500000000003, 241.31250000000003, 241.34000000000003, 241.36750000000004, 241.39500000000004, 241.42250000000004, 241.45000000000005, 241.47750000000002, 241.50500000000002, 241.53250000000003, 241.56000000000003, 241.58750000000003, 241.61500000000004, 241.64250000000004, 241.67000000000002, 241.69750000000005, 241.72500000000002, 241.75250000000003, 241.78000000000003, 241.80750000000003, 241.83500000000004, 241.86250000000004, 241.89000000000004, 241.91750000000002, 241.94500000000005, 241.97250000000003, 242.00000000000003} |
| Relative tolerance | 0.005 |

### TIME STEPPING

| Description | Value |
|---|---|
| Maximum BDF order | 2 |
| Nonlinear controller | On |

### LOG

| | | | | | | | | | | | |
|---|---|---|---|---|---|---|---|---|---|---|---|
| 495 | 232.22 | 0.055 | | 8 | 5 | 8 | 2 | 0 | 0 | 5e-13 | 1e-14 |
| - | 232.24 | - | out | | | | | | | | |
| - | 232.27 | - | out | | | | | | | | |
| 496 | 232.27 | 0.055 | | 10 | 6 | 10 | 2 | 0 | 0 | 1.4e-13 | 8.3e-15 |
| - | 232.29 | - | out | | | | | | | | |
| - | 232.32 | - | out | | | | | | | | |
| 497 | 232.33 | 0.055 | | 12 | 7 | 12 | 2 | 0 | 0 | 2.5e-13 | 7.8e-15 |
| - | 232.35 | - | out | | | | | | | | |
| - | 232.38 | - | out | | | | | | | | |
| 498 | 232.38 | 0.055 | | 14 | 8 | 14 | 2 | 0 | 0 | 1.3e-13 | 7.7e-15 |
| - | 232.4 | - | out | | | | | | | | |
| - | 232.43 | - | out | | | | | | | | |
| 499 | 232.44 | 0.055 | | 16 | 9 | 16 | 2 | 0 | 0 | 4.9e-13 | 8.5e-15 |
| - | 232.46 | - | out | | | | | | | | |
| - | 232.49 | - | out | | | | | | | | |
| 500 | 232.49 | 0.055 | | 18 | 10 | 18 | 2 | 0 | 0 | 4e-14 | 8.4e-15 |
| - | 232.51 | - | out | | | | | | | | |
| - | 232.54 | - | out | | | | | | | | |
| 501 | 232.55 | 0.055 | | 20 | 11 | 20 | 2 | 0 | 0 | 3.9e-13 | 8.3e-15 |
| - | 232.57 | - | out | | | | | | | | |
| - | 232.6 | - | out | | | | | | | | |
| 502 | 232.6 | 0.055 | | 22 | 12 | 22 | 2 | 0 | 0 | 6e-14 | 8.4e-15 |
| - | 232.62 | - | out | | | | | | | | |
| - | 232.65 | - | out | | | | | | | | |
| 503 | 232.66 | 0.055 | | 24 | 13 | 24 | 2 | 0 | 0 | 2.4e-13 | 8e-15 |
| - | 232.68 | - | out | | | | | | | | |
| - | 232.71 | - | out | | | | | | | | |
| 504 | 232.71 | 0.055 | | 26 | 14 | 26 | 2 | 0 | 0 | 1.9e-13 | 1e-14 |
| - | 232.73 | - | out | | | | | | | | |
| - | 232.76 | - | out | | | | | | | | |
| - | 232.79 | - | out | | | | | | | | |
| - | 232.82 | - | out | | | | | | | | |
| 505 | 232.82 | 0.11 | | 28 | 15 | 28 | 2 | 0 | 0 | 1.8e-13 | 6.3e-15 |
| - | 232.84 | - | out | | | | | | | | |
| 506 | 232.85 | 0.0275 | | 33 | 17 | 33 | 2 | 1 | 0 | 8.5e-14 | 6e-15 |
| - | 232.87 | - | out | | | | | | | | |
| - | 232.9 | - | out | | | | | | | | |
| 507 | 232.9 | 0.055 | | 35 | 18 | 35 | 2 | 1 | 0 | 2e-13 | 7e-15 |
| 508 | 232.92 | 0.01375 | | 40 | 20 | 40 | 2 | 2 | 0 | 3.1e-14 | 4.2e-15 |
| 509 | 232.92 | 0.006875 | | 44 | 22 | 44 | 2 | 3 | 0 | 1.5e-14 | 7.1e-15 |
| - | 232.93 | - | out | | | | | | | | |
| 510 | 232.93 | 0.0034375 | | 48 | 24 | 48 | 2 | 4 | 0 | 1.3e-14 | 6.2e-15 |
| 511 | 232.93 | 0.0017188 | | 53 | 26 | 53 | 2 | 5 | 0 | 1.2e-14 | 5.9e-15 |
| 512 | 232.93 | 0.00085938 | | 58 | 28 | 58 | 2 | 6 | 0 | 9.5e-15 | 3.5e-15 |
| 513 | 232.93 | 0.0017188 | | 59 | 29 | 59 | 2 | 6 | 0 | 3.1e-14 | 3.5e-15 |
| 514 | 232.93 | 0.00021484 | | 69 | 32 | 69 | 2 | 8 | 0 | 5.4e-15 | 5.3e-16 |
| 515 | 232.93 | 0.00016701 | | 74 | 34 | 74 | 2 | 9 | 0 | 2.2e-15 | 3.6e-16 |
| 516 | 232.93 | 6.116e-06 | | 88 | 38 | 88 | 1 | 12 | 0 | 3.6e-16 | 3e-16 |
| 517 | 232.93 | 1.2232e-05 | | 89 | 39 | 89 | 1 | 12 | 0 | 1.6e-16 | 1.1e-16 |
| 518 | 232.93 | 1.5775e-06 | | 101 | 42 | 101 | 1 | 14 | 0 | 5.2e-16 | 5.7e-16 |
| 519 | 232.93 | 3.1549e-06 | | 102 | 43 | 102 | 1 | 14 | 0 | 1.6e-16 | 1.7e-16 |
| 520 | 232.93 | 5.1659e-07 | | 112 | 46 | 112 | 1 | 16 | 0 | 3e-16 | 2.1e-16 |
| 521 | 232.93 | 4.3053e-07 | | 116 | 48 | 116 | 1 | 17 | 0 | 3.3e-16 | 3.6e-16 |
| 522 | 232.93 | 9.1749e-08 | | 123 | 51 | 123 | 1 | 19 | 0 | 3e-16 | 2.8e-16 |
| 523 | 232.93 | 1.0374e-07 | | 126 | 53 | 126 | 1 | 20 | 0 | 2.5e-16 | 2.4e-16 |
| 524 | 232.93 | 1.1457e-07 | | 130 | 55 | 130 | 1 | 21 | 0 | 2.3e-15 | 8e-16 |

| | | | | | | | | | | |
|---|---|---|---|---|---|---|---|---|---|---|
| 525 | 232.93 | 8.9759e-08 | 132 | 56 | 132 | 1 | 21 | 0 | 1e-14 | 1.1e-14 |
| 526 | 232.93 | 8.9759e-08 | 134 | 57 | 134 | 1 | 21 | 0 | 1.9e-14 | 1.9e-14 |
| 527 | 232.93 | 8.9759e-08 | 136 | 58 | 136 | 1 | 21 | 0 | 3.3e-14 | 3.4e-14 |
| 528 | 232.93 | 1.7952e-07 | 138 | 59 | 138 | 1 | 21 | 0 | 2.3e-14 | 2e-14 |
| 529 | 232.93 | 1.7952e-07 | 140 | 60 | 140 | 1 | 21 | 0 | 1.3e-14 | 9.5e-15 |
| 530 | 232.93 | 3.5904e-07 | 142 | 61 | 142 | 1 | 21 | 0 | 3.5e-15 | 1.7e-15 |
| 531 | 232.93 | 3.5904e-07 | 144 | 62 | 144 | 1 | 21 | 0 | 4.6e-15 | 2.2e-15 |
| 532 | 232.93 | 7.1807e-07 | 146 | 63 | 146 | 1 | 21 | 0 | 3.9e-15 | 1.6e-15 |
| 533 | 232.93 | 1.4361e-06 | 148 | 64 | 148 | 1 | 21 | 0 | 3.4e-15 | 1.3e-15 |
| 534 | 232.93 | 1.4361e-06 | 150 | 65 | 150 | 1 | 21 | 0 | 3.1e-15 | 1.6e-15 |
| 535 | 232.93 | 2.8723e-06 | 152 | 66 | 152 | 1 | 21 | 0 | 4.9e-15 | 3.1e-15 |
| 536 | 232.93 | 2.8723e-06 | 154 | 67 | 154 | 1 | 21 | 0 | 7.3e-15 | 4.2e-15 |
| 537 | 232.93 | 5.7446e-06 | 156 | 68 | 156 | 1 | 21 | 0 | 4.2e-15 | 1.3e-15 |
| 538 | 232.93 | 5.7446e-06 | 158 | 69 | 158 | 1 | 21 | 0 | 6.7e-15 | 1.7e-15 |
| 539 | 232.93 | 5.7446e-06 | 160 | 70 | 160 | 1 | 21 | 0 | 4.5e-15 | 3.1e-15 |
| 540 | 232.93 | 1.1489e-05 | 162 | 71 | 162 | 2 | 21 | 0 | 4.6e-15 | 2e-15 |
| 541 | 232.93 | 1.1489e-05 | 164 | 72 | 164 | 2 | 21 | 0 | 4e-15 | 2.5e-15 |
| 542 | 232.93 | 2.2978e-05 | 166 | 73 | 166 | 2 | 21 | 0 | 5.2e-15 | 6.1e-15 |
| 543 | 232.93 | 2.068e-05 | 168 | 74 | 168 | 2 | 21 | 0 | 2.4e-15 | 1e-15 |
| 544 | 232.93 | 2.068e-05 | 170 | 75 | 170 | 2 | 21 | 0 | 2.8e-15 | 3.7e-15 |
| 545 | 232.93 | 2.068e-05 | 172 | 76 | 172 | 2 | 21 | 0 | 2.5e-15 | 3.7e-15 |
| 546 | 232.93 | 2.068e-05 | 174 | 77 | 174 | 2 | 21 | 0 | 1.9e-15 | 2.4e-15 |
| 547 | 232.93 | 4.1361e-05 | 176 | 78 | 176 | 2 | 21 | 0 | 1.6e-15 | 1.6e-15 |
| 548 | 232.93 | 4.1361e-05 | 178 | 79 | 178 | 2 | 21 | 0 | 2.1e-15 | 1.6e-15 |
| 549 | 232.93 | 4.1361e-05 | 180 | 80 | 180 | 2 | 21 | 0 | 1.5e-15 | 1.2e-15 |
| 550 | 232.93 | 4.1361e-05 | 182 | 81 | 182 | 2 | 21 | 0 | 1.8e-15 | 1.4e-15 |
| 551 | 232.93 | 8.2722e-05 | 184 | 82 | 184 | 2 | 21 | 0 | 2e-15 | 1.8e-15 |
| 552 | 232.93 | 8.2722e-05 | 186 | 83 | 186 | 2 | 21 | 0 | 1.7e-15 | 2.5e-15 |
| 553 | 232.93 | 8.2722e-05 | 188 | 84 | 188 | 2 | 21 | 0 | 1.7e-15 | 2.4e-15 |
| 554 | 232.93 | 8.2722e-05 | 190 | 85 | 190 | 2 | 21 | 0 | 1.7e-15 | 2.6e-15 |
| 555 | 232.93 | 8.2722e-05 | 192 | 86 | 192 | 2 | 21 | 0 | 1.5e-15 | 2.3e-15 |
| 556 | 232.93 | 0.00016544 | 194 | 87 | 194 | 2 | 21 | 0 | 2.7e-15 | 4.9e-15 |
| 557 | 232.93 | 0.00016544 | 196 | 88 | 196 | 2 | 21 | 0 | 3.4e-15 | 1.2e-14 |
| 558 | 232.93 | 0.00016544 | 198 | 89 | 198 | 2 | 21 | 0 | 2e-15 | 1.2e-14 |
| 559 | 232.93 | 0.00016544 | 200 | 90 | 200 | 2 | 21 | 0 | 3.4e-15 | 1.1e-14 |
| 560 | 232.93 | 0.00016544 | 202 | 91 | 202 | 2 | 21 | 0 | 5.8e-15 | 6e-15 |
| 561 | 232.93 | 0.00033089 | 204 | 92 | 204 | 2 | 21 | 0 | 6.8e-15 | 1.2e-14 |
| 562 | 232.94 | 0.00033089 | 206 | 93 | 206 | 2 | 21 | 0 | 7.2e-15 | 1.2e-14 |
| 563 | 232.94 | 0.00033089 | 208 | 94 | 208 | 2 | 21 | 0 | 3.6e-15 | 9.8e-15 |
| 564 | 232.94 | 0.00033089 | 210 | 95 | 210 | 2 | 21 | 0 | 6.7e-15 | 9.7e-15 |
| 565 | 232.94 | 0.00033089 | 212 | 96 | 212 | 2 | 21 | 0 | 3.9e-15 | 7.7e-15 |
| 566 | 232.94 | 0.00033089 | 214 | 97 | 214 | 2 | 21 | 0 | 2.8e-15 | 7.2e-15 |
| 567 | 232.94 | 0.00066177 | 216 | 98 | 216 | 2 | 21 | 0 | 7.6e-15 | 1.1e-14 |
| 568 | 232.94 | 0.00066177 | 218 | 99 | 218 | 2 | 21 | 0 | 5.1e-15 | 9.7e-15 |
| 569 | 232.94 | 0.00066177 | 220 | 100 | 220 | 2 | 21 | 0 | 3.7e-15 | 1.1e-14 |
| 570 | 232.94 | 0.00066177 | 222 | 101 | 222 | 2 | 21 | 0 | 7.4e-15 | 1.1e-14 |
| 571 | 232.94 | 0.00066177 | 224 | 102 | 224 | 2 | 21 | 0 | 7.9e-15 | 1e-14 |
| 572 | 232.94 | 0.0013235 | 226 | 103 | 226 | 2 | 21 | 0 | 6.3e-15 | 1.8e-14 |
| 573 | 232.94 | 0.0013235 | 228 | 104 | 228 | 2 | 21 | 0 | 6.4e-15 | 1.5e-14 |
| 574 | 232.94 | 0.0013235 | 230 | 105 | 230 | 2 | 21 | 0 | 6.6e-15 | 1.6e-14 |
| 575 | 232.95 | 0.0013235 | 232 | 106 | 232 | 2 | 21 | 0 | 7e-15 | 1.7e-14 |
| 576 | 232.95 | 0.0013235 | 234 | 107 | 234 | 2 | 21 | 0 | 7.9e-15 | 1.6e-14 |
| 577 | 232.95 | 0.0013235 | 236 | 108 | 236 | 2 | 21 | 0 | 6e-15 | 1.6e-14 |
| 578 | 232.95 | 0.0026471 | 238 | 109 | 238 | 2 | 21 | 0 | 8.1e-15 | 2.8e-14 |
| - | 232.95 | - out | | | | | | | | |
| 579 | 232.95 | 0.0026471 | 240 | 110 | 240 | 2 | 21 | 0 | 8.2e-15 | 2.4e-14 |

| | | | | | | | | | | |
|---|---|---|---|---|---|---|---|---|---|---|
| 580 | 232.96 | 0.0026471 | 242 | 111 | 242 | 2 | 21 | 0 | 8.5e-15 | 2.9e-14 |
| 581 | 232.96 | 0.0026471 | 244 | 112 | 244 | 2 | 21 | 0 | 4e-15 | 2.9e-14 |
| 582 | 232.96 | 0.0026471 | 246 | 113 | 246 | 2 | 21 | 0 | 1.1e-14 | 3.1e-14 |
| 583 | 232.96 | 0.0026471 | 248 | 114 | 248 | 2 | 21 | 0 | 8.4e-15 | 2.9e-14 |
| 584 | 232.97 | 0.0026471 | 250 | 115 | 250 | 2 | 21 | 0 | 7.3e-15 | 2.7e-14 |
| 585 | 232.97 | 0.0026471 | 252 | 116 | 252 | 2 | 21 | 0 | 5.3e-15 | 2.6e-14 |
| 586 | 232.97 | 0.0026471 | 254 | 117 | 254 | 2 | 21 | 0 | 6.1e-15 | 2.3e-14 |
| 587 | 232.97 | 0.0026471 | 256 | 118 | 256 | 2 | 21 | 0 | 6.7e-15 | 2.4e-14 |
| 588 | 232.98 | 0.0026471 | 258 | 119 | 258 | 2 | 21 | 0 | 5.3e-15 | 2.5e-14 |
| 589 | 232.98 | 0.0026471 | 260 | 120 | 260 | 2 | 21 | 0 | 6.7e-15 | 2.6e-14 |
| - | 232.98 | - out | | | | | | | | |
| 590 | 232.98 | 0.0026471 | 262 | 121 | 262 | 2 | 21 | 0 | 7.1e-15 | 2.7e-14 |
| 591 | 232.98 | 0.0026471 | 264 | 122 | 264 | 2 | 21 | 0 | 5.5e-15 | 2.7e-14 |
| 592 | 232.99 | 0.0052942 | 266 | 123 | 266 | 2 | 21 | 0 | 6.2e-15 | 4.3e-14 |
| 593 | 233 | 0.0052942 | 268 | 124 | 268 | 2 | 21 | 0 | 8.5e-15 | 3.2e-14 |
| 594 | 233 | 0.0052942 | 270 | 125 | 270 | 2 | 21 | 0 | 9.7e-15 | 3.4e-14 |
| 595 | 233.01 | 0.0052942 | 272 | 126 | 272 | 2 | 21 | 0 | 9.7e-15 | 2.5e-14 |
| - | 233.01 | - out | | | | | | | | |
| 596 | 233.01 | 0.0052942 | 274 | 127 | 274 | 2 | 21 | 0 | 8.1e-15 | 1.7e-14 |
| 597 | 233.02 | 0.0052942 | 276 | 128 | 276 | 2 | 21 | 0 | 5.4e-15 | 1.9e-14 |
| 598 | 233.03 | 0.010588 | 278 | 129 | 278 | 2 | 21 | 0 | 2.3e-14 | 1.5e-14 |
| - | 233.04 | - out | | | | | | | | |
| 599 | 233.04 | 0.010588 | 280 | 130 | 280 | 2 | 21 | 0 | 2.3e-14 | 5.2e-15 |
| 600 | 233.06 | 0.021177 | 282 | 131 | 282 | 2 | 21 | 0 | 2e-14 | 4.4e-15 |
| - | 233.06 | - out | | | | | | | | |
| 601 | 233.08 | 0.021177 | 284 | 132 | 284 | 2 | 21 | 0 | 4.2e-13 | 3.7e-15 |
| - | 233.09 | - out | | | | | | | | |
| 602 | 233.1 | 0.021177 | 286 | 133 | 286 | 2 | 21 | 0 | 1e-13 | 5.2e-15 |
| - | 233.12 | - out | | | | | | | | |
| 603 | 233.12 | 0.021177 | 288 | 134 | 288 | 2 | 21 | 0 | 2e-13 | 7.5e-15 |
| 604 | 233.14 | 0.021177 | 290 | 135 | 290 | 2 | 21 | 0 | 5.7e-13 | 1.9e-14 |
| - | 233.15 | - out | | | | | | | | |
| 605 | 233.16 | 0.021177 | 292 | 136 | 292 | 2 | 21 | 0 | 3.6e-14 | 1.4e-14 |
| - | 233.17 | - out | | | | | | | | |
| 606 | 233.19 | 0.021177 | 294 | 137 | 294 | 2 | 21 | 0 | 4.6e-12 | 2.7e-14 |
| - | 233.2 | - out | | | | | | | | |
| 607 | 233.21 | 0.021177 | 296 | 138 | 296 | 2 | 21 | 0 | 6.5e-13 | 1.3e-14 |
| - | 233.23 | - out | | | | | | | | |
| 608 | 233.23 | 0.021177 | 298 | 139 | 298 | 2 | 21 | 0 | 1e-12 | 2.5e-14 |
| 609 | 233.25 | 0.021177 | 300 | 140 | 300 | 2 | 21 | 0 | 6.8e-13 | 4.7e-14 |
| - | 233.26 | - out | | | | | | | | |
| 610 | 233.27 | 0.021177 | 302 | 141 | 302 | 2 | 21 | 0 | 1.6e-13 | 5e-14 |
| - | 233.28 | - out | | | | | | | | |
| 611 | 233.29 | 0.021177 | 304 | 142 | 304 | 2 | 21 | 0 | 3.9e-13 | 4.2e-14 |
| - | 233.31 | - out | | | | | | | | |
| 612 | 233.31 | 0.021177 | 306 | 143 | 306 | 2 | 21 | 0 | 4.7e-13 | 3.3e-14 |
| 613 | 233.33 | 0.021177 | 308 | 144 | 308 | 2 | 21 | 0 | 8.1e-14 | 2.8e-14 |
| - | 233.34 | - out | | | | | | | | |
| 614 | 233.36 | 0.021177 | 310 | 145 | 310 | 2 | 21 | 0 | 1.4e-12 | 3.4e-14 |
| - | 233.37 | - out | | | | | | | | |
| 615 | 233.38 | 0.021177 | 312 | 146 | 312 | 2 | 21 | 0 | 4.6e-13 | 3.6e-14 |
| - | 233.39 | - out | | | | | | | | |
| 616 | 233.4 | 0.021177 | 314 | 147 | 314 | 2 | 21 | 0 | 2.3e-13 | 2.1e-14 |
| 617 | 233.42 | 0.021177 | 316 | 148 | 316 | 2 | 21 | 0 | 1.7e-13 | 2.6e-14 |
| - | 233.42 | - out | | | | | | | | |
| 618 | 233.44 | 0.021177 | 318 | 149 | 318 | 2 | 21 | 0 | 8.6e-13 | 2.9e-14 |

```
   -      233.45          - out
 619      233.46    0.021177      320  150  320     2    21      0  9.6e-13  4.1e-14
   -      233.48          - out
 620      233.48    0.021177      322  151  322     2    21      0  4.1e-13  5.4e-14
   -       233.5          - out
 621       233.5    0.021177      324  152  324     2    21      0  1.7e-12  2.6e-14
 622      233.52    0.021177      326  153  326     2    21      0  1.6e-13  3.8e-14
   -      233.53          - out
 623      233.55    0.021177      328  154  328     2    21      0  3.1e-13  3.2e-14
   -      233.56          - out
 624      233.57    0.021177      330  155  330     2    21      0  1.1e-12  2.7e-14
   -      233.59          - out
 625      233.59    0.021177      332  156  332     2    21      0  1.3e-12  2.2e-14
 626      233.61    0.021177      334  157  334     2    21      0  1.1e-12    2e-14
   -      233.61          - out
 627      233.63    0.021177      336  158  336     2    21      0  6.4e-13  1.6e-14
   -      233.64          - out
 628      233.65    0.021177      338  159  338     2    21      0  1.3e-12  1.5e-14
   -      233.67          - out
 629      233.67    0.021177      340  160  340     2    21      0  4.7e-12  1.2e-14
 630      233.69    0.021177      342  161  342     2    21      0  1.8e-12  9.8e-15
   -       233.7          - out
 631      233.72    0.021177      344  162  344     2    21      0  8.6e-13  6.8e-15
   -      233.72          - out
 632      233.74    0.021177      346  163  346     2    21      0  8.2e-12  9.3e-15
 633      233.75    0.011194      350  165  350     2    22      0  6.1e-13  2.8e-15
   -      233.75          - out
 634      233.75   0.0069539      354  167  354     2    23      0  1.4e-12    3e-15
 635      233.76   0.0062585      356  168  356     2    23      0  1.2e-12  2.3e-15
 636      233.77   0.0062585      358  169  358     2    23      0  8.6e-13  1.9e-15
 637      233.77   0.0056327      360  170  360     2    23      0  2.8e-14  2.5e-14
   -      233.78          - out
 638      233.78   0.0056327      362  171  362     2    23      0  1.5e-14  1.9e-14
 639      233.78   0.0056327      364  172  364     2    23      0  2.5e-14  1.3e-14
 640       233.8    0.011265      366  173  366     2    23      0  1.8e-13  1.4e-14
   -      233.81          - out
 641      233.81    0.011265      368  174  368     2    23      0  7.5e-13  9.5e-15
 642      233.83    0.022531      370  175  370     2    23      0  3.2e-12  1.7e-14
   -      233.83          - out
 643      233.85    0.022531      372  176  372     2    23      0  9.3e-13  1.4e-14
Number of vertex elements: 3
Number of boundary elements: 112
Number of vertex elements: 3
Number of boundary elements: 112
Number of elements: 534
Minimum element quality: 0.5661
Number of vertex elements: 4
Number of boundary elements: 162
Number of vertex elements: 5
Number of boundary elements: 212
Number of vertex elements: 6
Number of boundary elements: 266
Minimum element quality: 0.5535
Geometry shape function: Linear Lagrange
Time interval 9
Time-dependent solver (BDF)
```

```
Number of degrees of freedom solved for: 11758 (plus 7124 internal DOFs).
Nonsymmetric matrix found.
Scales for dependent variables:
Concentration (comp1.cOx): 2.8
Concentration (comp1.cRed): 5.1
Spatial mesh displacement (comp1.spatial.disp): 6.9e-08
Step         Time    Stepsize      Res  Jac  Sol Order Tfail NLfail   LinErr   LinRes
   -       233.83           - out
   -       233.83           - out
 644       233.85    0.022531        2    2    2     1     0      0  7.1e-13  8.3e-16
   -       233.86           - out
 645       233.87    0.022531        4    3    4     1     0      0  2.1e-12    1e-14
   -       233.89           - out
   -       233.92           - out
 646       233.92    0.045061        6    4    6     2     0      0  4.6e-13  1.5e-14
   -       233.94           - out
 647       233.96    0.045061        8    5    8     2     0      0  7.6e-12  1.6e-14
   -       233.97           - out
   -          234           - out
 648       234.01    0.045061       10    6   10     2     0      0  1.6e-11  4.3e-14
   -       234.03           - out
   -       234.05           - out
 649       234.05    0.045061       12    7   12     2     0      0  7.3e-13    3e-14
   -       234.08           - out
 650        234.1    0.045061       14    8   14     2     0      0  5.7e-12  3.5e-14
   -       234.11           - out
   -       234.14           - out
 651       234.14    0.040555       16    9   16     2     0      0    7e-13  1.9e-14
   -       234.16           - out
 652       234.18      0.0365       18   10   18     2     0      0  3.3e-13  2.5e-14
   -       234.19           - out
 653       234.21      0.0365       20   11   20     2     0      0    3e-13  3.1e-14
   -       234.22           - out
   -       234.25           - out
 654       234.25     0.03285       22   12   22     2     0      0  6.8e-12  3.7e-14
   -       234.27           - out
 655       234.28     0.03285       24   13   24     2     0      0  9.5e-13  4.9e-14
   -        234.3           - out
 656       234.31     0.03285       26   14   26     2     0      0  2.7e-12    5e-14
   -       234.33           - out
 657       234.34     0.03285       28   15   28     2     0      0  1.9e-12  3.2e-14
   -       234.36           - out
 658       234.38     0.03285       30   16   30     2     0      0  1.6e-12    6e-14
   -       234.38           - out
   -       234.41           - out
 659       234.41     0.03285       32   17   32     2     0      0  2.3e-12  4.8e-14
   -       234.44           - out
 660       234.44     0.03285       34   18   34     2     0      0  1.2e-12    4e-14
   -       234.47           - out
 661       234.48     0.03285       36   19   36     2     0      0  2.1e-13  2.3e-14
   -       234.49           - out
 662       234.51     0.03285       38   20   38     2     0      0  4.3e-12  9.6e-15
   -       234.52           - out
 663       234.54     0.03285       40   21   40     2     0      0  1.5e-12  9.8e-15
   -       234.55           - out
 664       234.57     0.03285       42   22   42     2     0      0  3.8e-13  6.4e-15
```

| | | | | | | | | | | |
|---|---|---|---|---|---|---|---|---|---|---|
| - | 234.58 | - | out | | | | | | | |
| - | 234.6 | - | out | | | | | | | |
| 665 | 234.61 | 0.03285 | 44 | 23 | 44 | 2 | 0 | 0 | 9.9e-13 | 4.7e-15 |
| - | 234.63 | - | out | | | | | | | |
| 666 | 234.64 | 0.03285 | 46 | 24 | 46 | 2 | 0 | 0 | 4.1e-13 | 4.7e-15 |
| - | 234.66 | - | out | | | | | | | |
| 667 | 234.67 | 0.03285 | 48 | 25 | 48 | 2 | 0 | 0 | 3.9e-13 | 4.2e-15 |
| - | 234.69 | - | out | | | | | | | |
| 668 | 234.71 | 0.03285 | 50 | 26 | 50 | 2 | 0 | 0 | 1.1e-13 | 5.7e-15 |
| - | 234.71 | - | out | | | | | | | |
| 669 | 234.74 | 0.03285 | 52 | 27 | 52 | 2 | 0 | 0 | 5.8e-14 | 6.3e-15 |
| - | 234.74 | - | out | | | | | | | |
| - | 234.77 | - | out | | | | | | | |
| 670 | 234.77 | 0.03285 | 54 | 28 | 54 | 2 | 0 | 0 | 7.5e-13 | 9.6e-15 |
| - | 234.8 | - | out | | | | | | | |
| 671 | 234.8 | 0.03285 | 56 | 29 | 56 | 2 | 0 | 0 | 1.3e-13 | 6.9e-15 |
| - | 234.82 | - | out | | | | | | | |
| 672 | 234.84 | 0.03285 | 58 | 30 | 58 | 2 | 0 | 0 | 1e-13 | 8.4e-15 |
| - | 234.85 | - | out | | | | | | | |
| 673 | 234.87 | 0.03285 | 60 | 31 | 60 | 2 | 0 | 0 | 4.8e-14 | 1.1e-14 |
| - | 234.88 | - | out | | | | | | | |
| - | 234.91 | - | out | | | | | | | |
| - | 234.93 | - | out | | | | | | | |
| 674 | 234.94 | 0.0657 | 62 | 32 | 62 | 2 | 0 | 0 | 7.3e-14 | 9.4e-15 |
| - | 234.96 | - | out | | | | | | | |
| - | 234.99 | - | out | | | | | | | |
| 675 | 235 | 0.0657 | 64 | 33 | 64 | 2 | 0 | 0 | 3.5e-13 | 1.2e-14 |
| - | 235.02 | - | out | | | | | | | |
| 676 | 235.02 | 0.014872 | 71 | 35 | 71 | 2 | 1 | 0 | 6e-14 | 9.6e-15 |
| 677 | 235.02 | 0.0018589 | 82 | 38 | 82 | 2 | 3 | 0 | 1.5e-14 | 1.7e-14 |
| 678 | 235.02 | 0.0037179 | 83 | 39 | 83 | 2 | 3 | 0 | 2.6e-14 | 3.1e-14 |
| 679 | 235.02 | 0.00011618 | 98 | 43 | 98 | 1 | 6 | 0 | 4.1e-15 | 2.6e-15 |
| 680 | 235.02 | 0.00023237 | 99 | 44 | 99 | 1 | 6 | 0 | 2.2e-14 | 1.7e-15 |
| 681 | 235.02 | 2.9046e-05 | 115 | 47 | 115 | 1 | 8 | 0 | 2.8e-14 | 1.4e-14 |
| 682 | 235.02 | 9.878e-06 | 120 | 49 | 120 | 1 | 9 | 0 | 3.2e-14 | 2.1e-14 |
| 683 | 235.02 | 9.878e-06 | 122 | 50 | 122 | 1 | 9 | 0 | 4.7e-15 | 8.1e-16 |
| 684 | 235.02 | 9.878e-06 | 124 | 51 | 124 | 1 | 9 | 0 | 3.2e-15 | 6.1e-16 |
| 685 | 235.02 | 9.878e-06 | 126 | 52 | 126 | 1 | 9 | 0 | 2.2e-15 | 6.6e-16 |
| 686 | 235.02 | 1.9756e-05 | 128 | 53 | 128 | 2 | 9 | 0 | 4e-15 | 6.4e-16 |
| 687 | 235.02 | 1.9756e-05 | 130 | 54 | 130 | 2 | 9 | 0 | 3.3e-15 | 1e-15 |
| 688 | 235.02 | 3.9512e-05 | 132 | 55 | 132 | 2 | 9 | 0 | 2.9e-15 | 7.1e-16 |
| 689 | 235.02 | 3.9512e-05 | 134 | 56 | 134 | 2 | 9 | 0 | 2.4e-15 | 7e-16 |
| 690 | 235.02 | 3.9512e-05 | 136 | 57 | 136 | 2 | 9 | 0 | 3e-15 | 1.3e-15 |
| 691 | 235.02 | 3.9512e-05 | 138 | 58 | 138 | 2 | 9 | 0 | 2e-15 | 8.9e-16 |
| 692 | 235.02 | 3.9512e-05 | 140 | 59 | 140 | 2 | 9 | 0 | 2e-15 | 9.2e-16 |
| 693 | 235.02 | 7.9024e-05 | 142 | 60 | 142 | 2 | 9 | 0 | 2.9e-15 | 1.5e-15 |
| 694 | 235.02 | 7.9024e-05 | 144 | 61 | 144 | 2 | 9 | 0 | 1.8e-15 | 1.4e-15 |
| 695 | 235.02 | 7.9024e-05 | 146 | 62 | 146 | 2 | 9 | 0 | 2.3e-15 | 1.3e-15 |
| 696 | 235.02 | 7.9024e-05 | 148 | 63 | 148 | 2 | 9 | 0 | 2e-15 | 1.3e-15 |
| 697 | 235.02 | 0.00015805 | 150 | 64 | 150 | 2 | 9 | 0 | 3.1e-15 | 2.2e-15 |
| 698 | 235.02 | 0.00015805 | 152 | 65 | 152 | 2 | 9 | 0 | 2.9e-15 | 2.7e-15 |
| 699 | 235.02 | 0.00015805 | 154 | 66 | 154 | 2 | 9 | 0 | 3.2e-15 | 2.6e-15 |
| 700 | 235.02 | 0.00015805 | 156 | 67 | 156 | 2 | 9 | 0 | 8.6e-15 | 2.9e-15 |
| 701 | 235.02 | 0.00015805 | 158 | 68 | 158 | 2 | 9 | 0 | 9.2e-15 | 3.5e-15 |
| 702 | 235.02 | 0.0003161 | 160 | 69 | 160 | 2 | 9 | 0 | 1.1e-14 | 5.5e-15 |
| 703 | 235.02 | 0.0003161 | 162 | 70 | 162 | 2 | 9 | 0 | 4.7e-15 | 6.4e-15 |

| | | | | | | | | | | |
|---|---|---|---|---|---|---|---|---|---|---|
| 704 | 235.02 | 0.0003161 | 164 | 71 | 164 | 2 | 9 | 0 | 6.8e-15 | 6.7e-15 |
| 705 | 235.03 | 0.0003161 | 166 | 72 | 166 | 2 | 9 | 0 | 5e-15 | 7.3e-15 |
| 706 | 235.03 | 0.0003161 | 168 | 73 | 168 | 2 | 9 | 0 | 4.2e-15 | 7.5e-15 |
| 707 | 235.03 | 0.0003161 | 170 | 74 | 170 | 2 | 9 | 0 | 3.6e-15 | 7.4e-15 |
| 708 | 235.03 | 0.0006322 | 172 | 75 | 172 | 2 | 9 | 0 | 5.8e-15 | 1.3e-14 |
| 709 | 235.03 | 0.0006322 | 174 | 76 | 174 | 2 | 9 | 0 | 1.2e-14 | 1.2e-14 |
| 710 | 235.03 | 0.0006322 | 176 | 77 | 176 | 2 | 9 | 0 | 5.3e-15 | 1.3e-14 |
| 711 | 235.03 | 0.0006322 | 178 | 78 | 178 | 2 | 9 | 0 | 9.4e-15 | 1.3e-14 |
| 712 | 235.03 | 0.0006322 | 180 | 79 | 180 | 2 | 9 | 0 | 5.3e-15 | 1.3e-14 |
| 713 | 235.03 | 0.0012644 | 182 | 80 | 182 | 2 | 9 | 0 | 1.4e-14 | 2.1e-14 |
| 714 | 235.03 | 0.0012644 | 184 | 81 | 184 | 2 | 9 | 0 | 1.1e-14 | 2.2e-14 |
| 715 | 235.03 | 0.0012644 | 186 | 82 | 186 | 2 | 9 | 0 | 1.4e-14 | 2.2e-14 |
| 716 | 235.03 | 0.0012644 | 188 | 83 | 188 | 2 | 9 | 0 | 7.8e-15 | 2.1e-14 |
| 717 | 235.04 | 0.0012644 | 190 | 84 | 190 | 2 | 9 | 0 | 1.1e-14 | 2.1e-14 |
| 718 | 235.04 | 0.0012644 | 192 | 85 | 192 | 2 | 9 | 0 | 6.9e-15 | 2.2e-14 |
| 719 | 235.04 | 0.0025288 | 194 | 86 | 194 | 2 | 9 | 0 | 6.7e-15 | 3.8e-14 |
| 720 | 235.04 | 0.0025288 | 196 | 87 | 196 | 2 | 9 | 0 | 6.9e-15 | 3.4e-14 |
| - | 235.04 | - | out | | | | | | | |
| 721 | 235.04 | 0.0025288 | 198 | 88 | 198 | 2 | 9 | 0 | 8e-15 | 3.4e-14 |
| 722 | 235.05 | 0.0025288 | 200 | 89 | 200 | 2 | 9 | 0 | 8.6e-15 | 3.2e-14 |
| 723 | 235.05 | 0.0025288 | 202 | 90 | 202 | 2 | 9 | 0 | 9.1e-15 | 3e-14 |
| 724 | 235.05 | 0.0025288 | 204 | 91 | 204 | 2 | 9 | 0 | 7.8e-15 | 2.9e-14 |
| 725 | 235.05 | 0.0025288 | 206 | 92 | 206 | 2 | 9 | 0 | 8.5e-15 | 2.9e-14 |
| 726 | 235.06 | 0.0025288 | 208 | 93 | 208 | 2 | 9 | 0 | 1e-14 | 2.8e-14 |
| 727 | 235.06 | 0.0050576 | 210 | 94 | 210 | 2 | 9 | 0 | 9.9e-15 | 4.7e-14 |
| 728 | 235.07 | 0.0050576 | 212 | 95 | 212 | 2 | 9 | 0 | 9.2e-15 | 4.7e-14 |
| - | 235.07 | - | out | | | | | | | |
| 729 | 235.07 | 0.0050576 | 214 | 96 | 214 | 2 | 9 | 0 | 8.8e-15 | 4.6e-14 |
| 730 | 235.08 | 0.0050576 | 216 | 97 | 216 | 2 | 9 | 0 | 1.1e-14 | 5e-14 |
| 731 | 235.08 | 0.0050576 | 218 | 98 | 218 | 2 | 9 | 0 | 1.4e-14 | 4.9e-14 |
| 732 | 235.09 | 0.010115 | 220 | 99 | 220 | 2 | 9 | 0 | 4e-14 | 7.1e-14 |
| - | 235.1 | - | out | | | | | | | |
| 733 | 235.11 | 0.02023 | 222 | 100 | 222 | 1 | 9 | 0 | 6e-14 | 1.7e-14 |
| - | 235.13 | - | out | | | | | | | |
| 734 | 235.13 | 0.02023 | 224 | 101 | 224 | 1 | 9 | 0 | 8.2e-14 | 1.2e-14 |
| - | 235.15 | - | out | | | | | | | |
| 735 | 235.15 | 0.02023 | 226 | 102 | 226 | 1 | 9 | 0 | 1e-13 | 1.2e-14 |
| - | 235.18 | - | out | | | | | | | |
| 736 | 235.19 | 0.04046 | 228 | 103 | 228 | 2 | 9 | 0 | 2.1e-13 | 1.4e-14 |
| - | 235.21 | - | out | | | | | | | |
| 737 | 235.23 | 0.04046 | 230 | 104 | 230 | 2 | 9 | 0 | 3.7e-13 | 7.5e-15 |
| - | 235.24 | - | out | | | | | | | |
| - | 235.26 | - | out | | | | | | | |
| - | 235.29 | - | out | | | | | | | |
| 738 | 235.31 | 0.080921 | 232 | 105 | 232 | 2 | 9 | 0 | 7.5e-13 | 1.4e-14 |
| - | 235.32 | - | out | | | | | | | |
| - | 235.35 | - | out | | | | | | | |
| - | 235.37 | - | out | | | | | | | |
| 739 | 235.38 | 0.067264 | 234 | 106 | 234 | 2 | 9 | 0 | 1.6e-13 | 1.3e-14 |
| - | 235.4 | - | out | | | | | | | |
| - | 235.43 | - | out | | | | | | | |
| 740 | 235.45 | 0.067264 | 236 | 107 | 236 | 2 | 9 | 0 | 3.7e-13 | 1.3e-14 |
| - | 235.46 | - | out | | | | | | | |
| - | 235.48 | - | out | | | | | | | |
| 741 | 235.51 | 0.060538 | 238 | 108 | 238 | 2 | 9 | 0 | 1.1e-12 | 1.3e-14 |
| - | 235.51 | - | out | | | | | | | |

```
   -      235.54           - out
   -      235.57           - out
 742      235.57    0.060538      240  109  240     2     9      0  1.4e-12  1.3e-14
   -      235.59           - out
   -      235.62           - out
 743      235.63    0.060538      242  110  242     2     9      0  2.6e-13  1.3e-14
   -      235.65           - out
   -      235.68           - out
 744      235.69    0.060538      244  111  244     2     9      0  1.2e-13  1.3e-14
   -      235.69           - out
 745      235.75    0.060538      246  112  246     2     9      0  1.5e-12  1.3e-14
Number of vertex elements: 3
Number of boundary elements: 112
Number of vertex elements: 3
Number of boundary elements: 112
Number of elements: 534
Minimum element quality: 0.5661
Number of vertex elements: 4
Number of boundary elements: 162
Number of vertex elements: 5
Number of boundary elements: 212
Number of vertex elements: 6
Number of boundary elements: 266
Minimum element quality: 0.5661
Geometry shape function: Linear Lagrange
Time interval 10
Time-dependent solver (BDF)
Number of degrees of freedom solved for: 11758 (plus 7124 internal DOFs).
Nonsymmetric matrix found.
Scales for dependent variables:
Concentration (comp1.cOx): 3.9
Concentration (comp1.cRed): 2.1
Spatial mesh displacement (comp1.spatial.disp): 6.9e-08
Step        Time    Stepsize      Res  Jac  Sol Order Tfail NLfail   LinErr   LinRes
   -      235.69           - out
   -       235.7           - out
 746      235.72      0.0275        2    2    2     1     0      0  9.7e-14  4.8e-15
   -      235.73           - out
 747      235.75      0.0275        4    3    4     1     0      0  2.6e-12  6.4e-15
   -      235.76           - out
   -      235.79           - out
 748       235.8       0.055        6    4    6     2     0      0  1.2e-11  3.6e-15
   -      235.81           - out
   -      235.84           - out
 749      235.86       0.055        8    5    8     2     0      0  4.5e-13  7.1e-15
   -      235.87           - out
   -       235.9           - out
 750      235.91       0.055       10    6   10     2     0      0  3.2e-12    9e-15
   -      235.92           - out
   -      235.95           - out
 751      235.97       0.055       12    7   12     2     0      0  1.1e-12  8.8e-15
   -      235.98           - out
   -      236.01           - out
 752      236.02       0.055       14    8   14     2     0      0  2.9e-13  8.7e-15
   -      236.03           - out
   -      236.06           - out
```

```
 753       236.08       0.055       16     9    16      2      0       0  2.5e-12  8.3e-15
   -       236.09           - out
   -       236.12           - out
 754       236.13       0.055       18    10    18      2      0       0  3.8e-12  8.9e-15
   -       236.14           - out
   -       236.17           - out
 755       236.19       0.055       20    11    20      2      0       0  4.1e-12  9.1e-15
   -        236.2           - out
   -       236.23           - out
 756       236.24       0.055       22    12    22      2      0       0  2.7e-12  8.3e-15
   -       236.25           - out
   -       236.28           - out
 757        236.3       0.055       24    13    24      2      0       0  2.3e-12  8.8e-15
   -       236.31           - out
   -       236.34           - out
 758       236.35       0.055       26    14    26      2      0       0  3.7e-12  1.1e-14
   -       236.36           - out
   -       236.39           - out
 759       236.41       0.055       28    15    28      2      0       0  8.3e-12  9.2e-15
   -       236.42           - out
   -       236.45           - out
 760       236.46       0.055       30    16    30      2      0       0  1.2e-11  9.8e-15
   -       236.47           - out
 761       236.49    0.027981       34    18    34      2      1       0  1.7e-12  8.6e-15
   -        236.5           - out
 762       236.51    0.016064       39    20    39      2      2       0  4.5e-12  6.4e-15
Time-stepping completed.
Geometry shape function: Linear Lagrange
Solution time: 1033 s. (17 minutes, 13 seconds)
Physical memory: 2.51 GB
Virtual memory: 2.62 GB
Ended at May 31, 2023 9:50:38 PM.
----- Time-Dependent Solver 1 in Study 5 (CV 39 to 43)/Solution 10 (sol10) ---->
```

**Advanced (aDef)**

### ASSEMBLY SETTINGS

| Description | Value |
|---|---|
| Reuse sparsity pattern | On |

**Fully Coupled 1 (fc1)**

### GENERAL

| Description | Value |
|---|---|
| Linear solver | Direct 1 |

### METHOD AND TERMINATION

| Description | Value |
|---|---|
| Damping factor | 0.9 |
| Jacobian update | Once per time step |
| Maximum number of iterations | 8 |

| Description | Value |
| --- | --- |
| Stabilization and acceleration | Anderson acceleration |
| Dimension of iteration space | 5 |

#### Automatic Remeshing 1 (ar1)

##### GENERAL

| Description | Value |
| --- | --- |
| Remesh in geometry | Geometry 1 |

##### CONDITION FOR REMESHING

| Description | Value |
| --- | --- |
| Condition type | Distortion |
| Stop when distortion exceeds | 1.2 |

##### OUTPUT

| Description | Value |
| --- | --- |
| Solution | Remeshed Solution 5 |
| Meshes | {mesh11, mesh12, mesh13, mesh14, mesh15, mesh16, mesh17, mesh18, mesh19} |

# 8 Results

## 8.1 DATASETS

### 8.1.1 No Solution

SOLUTION

| Description | Value |
| --- | --- |
| Component | Component 1 (comp1) |

### 8.1.2 Revolution 2D 2

DATA

| Description | Value |
| --- | --- |
| Dataset | No Solution |

AXIS DATA

| Description | Value |
| --- | --- |
| Axis entry method | Two points |
| Points | {{0, 0}, {0, 1}} |

REVOLUTION LAYERS

| Description | Value |
| --- | --- |
| Start angle | -90 |
| Revolution angle | 225 |

### 8.1.3 Grid 1D 1

DATA

| Description | Value |
| --- | --- |
| Function | All |
| Refresh | |

PARAMETER BOUNDS

| Description | Value |
| --- | --- |
| Name | t |
| Minimum | 0 |
| Maximum | 236.50000000000003 |

RESOLUTION

| Description | Value |
| --- | --- |
| Resolution | 10000 |

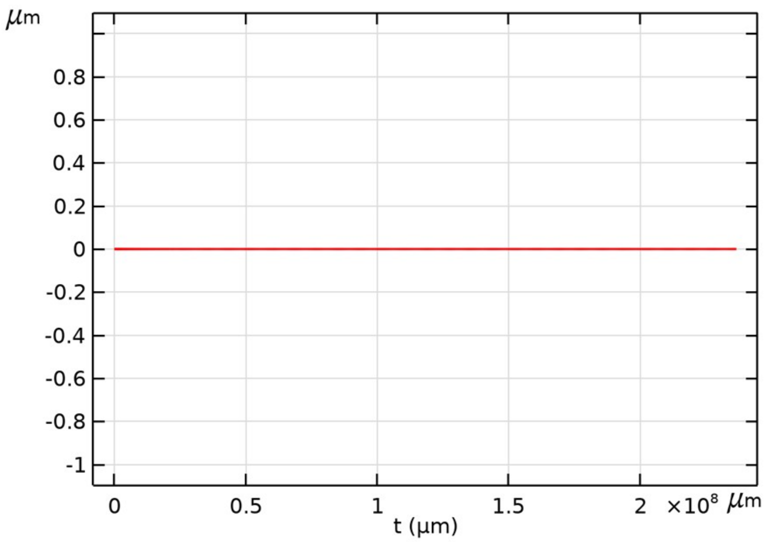


*Dataset: Grid 1D 1*

### 8.1.4 Study 1 (CV 1 to 39)/Solution 1

SOLUTION

| Description | Value |
| --- | --- |
| Solution | Solution 1 |
| Component | Component 1 (comp1) |

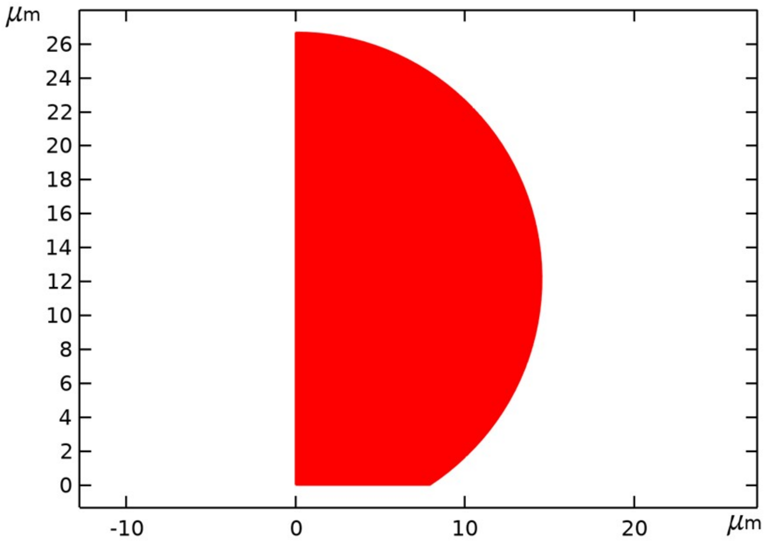


*Dataset: Study 1 (CV 1 to 39)/Solution 1*

### 8.1.5 Study 1 (CV 1 to 39)/Remeshed Solution 1

SOLUTION

| Description | Value |
|---|---|
| Solution | Remeshed Solution 1 |
| Component | Component 1 (comp1) |



*Dataset: Study 1 (CV 1 to 39)/Remeshed Solution 1*

### 8.1.6 Study 2 (CV 40 to 44)/Solution 4

SOLUTION

| Description | Value |
|---|---|
| Solution | Solution 4 |
| Component | Component 1 (comp1) |

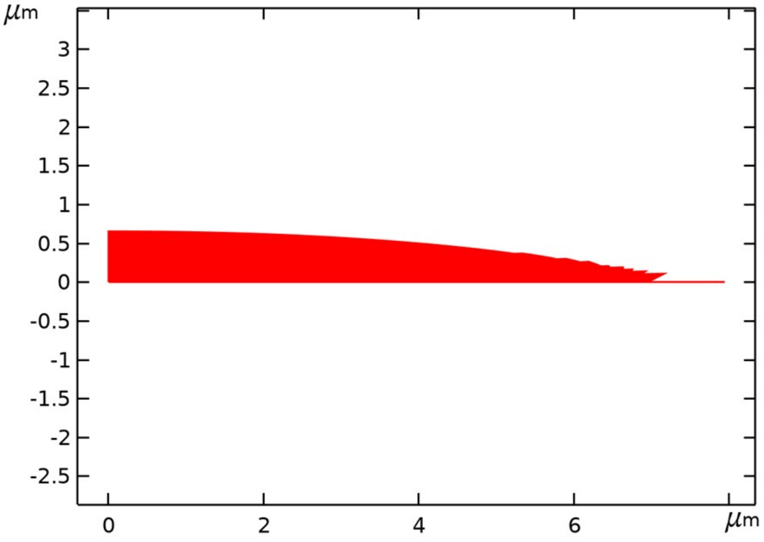


*Dataset: Study 2 (CV 40 to 44)/Solution 4*

### 8.1.7 Study 2 (CV 40 to 44)/Remeshed Solution 2

SOLUTION

| Description | Value |
|---|---|
| Solution | Remeshed Solution 2 |
| Component | Component 1 (comp1) |

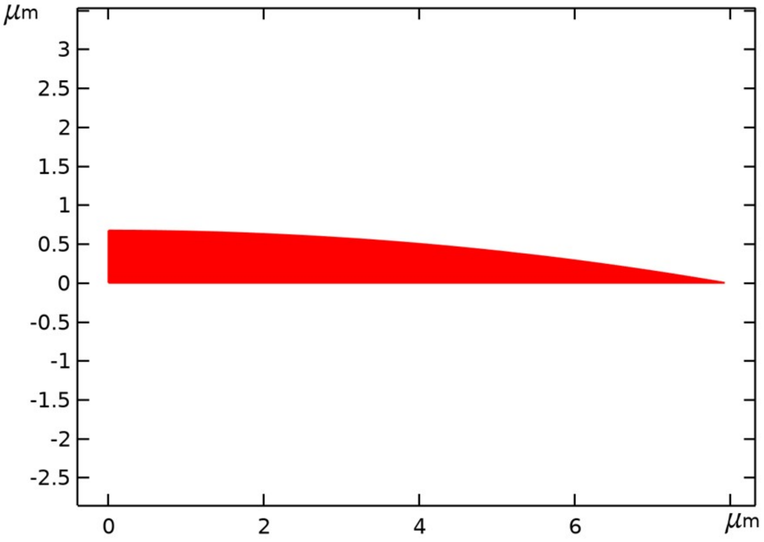


*Dataset: Study 2 (CV 40 to 44)/Remeshed Solution 2*

### 8.1.8 Revolution 2D 3

DATA

| Description | Value |
| --- | --- |
| Dataset | Study 2 (CV 40 to 44)/Remeshed Solution 2 |

AXIS DATA

| Description | Value |
| --- | --- |
| Axis entry method | Two points |
| Points | {{0, 0}, {0, 1}} |

REVOLUTION LAYERS

| Description | Value |
| --- | --- |
| Start angle | -90 |
| Revolution angle | 225 |

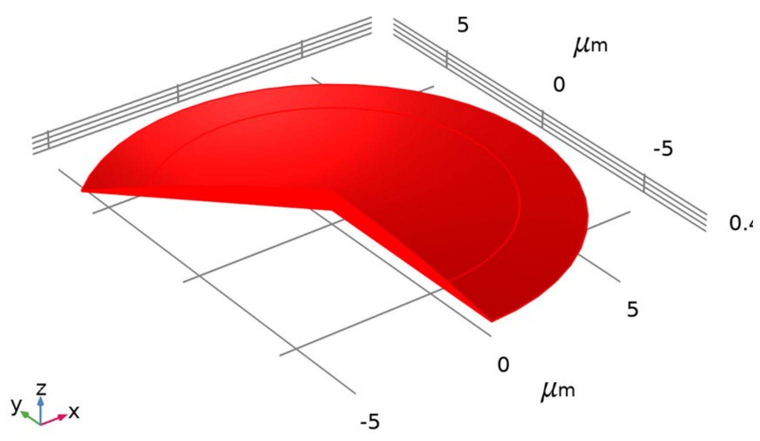


*Dataset: Revolution 2D 3*

### 8.1.9 Study 3 (CV 21 to 30)/Solution 6

SOLUTION

| Description | Value |
| --- | --- |
| Solution | Solution 6 |
| Component | Component 1 (comp1) |
| Geometry | Geometry 1 |

### 8.1.10 Revolution 2D 4

DATA

| Description | Value |
| --- | --- |

| Description | Value |
|---|---|
| Dataset | Study 3 (CV 21 to 30)/Solution 6 |

AXIS DATA

| Description | Value |
|---|---|
| Axis entry method | Two points |
| Points | {{0, 0}, {0, 1}} |

REVOLUTION LAYERS

| Description | Value |
|---|---|
| Start angle | -90 |
| Revolution angle | 225 |

### 8.1.11 Study 3 (CV 21 to 30)/Remeshed Solution 3

SOLUTION

| Description | Value |
|---|---|
| Solution | Remeshed Solution 3 |
| Component | Component 1 (comp1) |
| Geometry | Geometry 1 |

### 8.1.12 Revolution 2D 5

DATA

| Description | Value |
|---|---|
| Dataset | Study 3 (CV 21 to 30)/Remeshed Solution 3 |

AXIS DATA

| Description | Value |
|---|---|
| Axis entry method | Two points |
| Points | {{0, 0}, {0, 1}} |

REVOLUTION LAYERS

| Description | Value |
|---|---|
| Start angle | -90 |
| Revolution angle | 225 |

### 8.1.13 Study 4 (CV 31 to 38)/Solution 8

SOLUTION

| Description | Value |
|---|---|

| Description | Value |
|---|---|
| Solution | Solution 8 |
| Component | Component 1 (comp1) |
| Geometry | Geometry 1 |

### 8.1.14 Revolution 2D 6

DATA

| Description | Value |
|---|---|
| Dataset | Study 4 (CV 31 to 38)/Solution 8 |

AXIS DATA

| Description | Value |
|---|---|
| Axis entry method | Two points |
| Points | {{0, 0}, {0, 1}} |

REVOLUTION LAYERS

| Description | Value |
|---|---|
| Start angle | -90 |
| Revolution angle | 225 |

### 8.1.15 Study 4 (CV 31 to 38)/Remeshed Solution 4

SOLUTION

| Description | Value |
|---|---|
| Solution | Remeshed Solution 4 |
| Component | Component 1 (comp1) |
| Geometry | Geometry 1 |

### 8.1.16 Revolution 2D 7

DATA

| Description | Value |
|---|---|
| Dataset | Study 4 (CV 31 to 38)/Remeshed Solution 4 |

AXIS DATA

| Description | Value |
|---|---|
| Axis entry method | Two points |
| Points | {{0, 0}, {0, 1}} |

REVOLUTION LAYERS

| Description | Value |
| --- | --- |
| Start angle | -90 |
| Revolution angle | 225 |

### 8.1.17 Study 5 (CV 39 to 44)/Solution 10

SOLUTION

| Description | Value |
| --- | --- |
| Solution | Solution 10 |
| Component | Component 1 (comp1) |
| Geometry | Geometry 1 |

### 8.1.18 Study 5 (CV 39 to 44)/Remeshed Solution 5

SOLUTION

| Description | Value |
| --- | --- |
| Solution | Remeshed Solution 5 |
| Component | Component 1 (comp1) |
| Geometry | Geometry 1 |

## 8.2 DERIVED VALUES

### 8.2.1 Global Evaluation 1

OUTPUT

| | |
| --- | --- |
| Evaluated in | Table 43 |

DATA

| Description | Value |
| --- | --- |
| Dataset | Study 1 (CV 1 to 39)/Solution 1 |

EXPRESSIONS

| Expression | Unit | Description |
| --- | --- | --- |
| n_Ox+n_Red | mol | |

## 8.3 TABLES

### 8.3.1 CV-1

| E (V) | i (nA) |
| --- | --- |
| -0.34800 | 0.13920 |
| -0.34300 | -0.043410 |

| E (V) | i (nA) |
|---|---|
| -0.33800 | -0.0079200 |
| -0.33300 | -0.0031500 |
| -0.32800 | 0.0038200 |
| -0.32300 | 0.012560 |
| -0.31800 | -0.0026300 |
| -0.31300 | 0.010290 |
| -0.30800 | 0.0071100 |
| -0.30300 | 0.013600 |
| -0.29800 | 0.0063000 |
| -0.29300 | 0.0088100 |
| -0.28800 | 0.010100 |
| -0.28300 | 0.011650 |
| -0.27800 | 0.013050 |
| -0.27300 | 0.011560 |
| -0.26800 | 0.013620 |
| -0.26300 | 0.011430 |
| -0.25800 | 0.011560 |
| -0.25300 | 0.0093300 |
| -0.24800 | 0.012660 |
| -0.24300 | 0.012520 |
| -0.23800 | 0.011330 |
| -0.23300 | 0.010720 |
| -0.22800 | 0.012060 |
| -0.22300 | 0.012680 |
| -0.21800 | 0.011400 |
| -0.21300 | 0.013380 |
| -0.20800 | 0.014950 |
| -0.20300 | 0.015550 |
| -0.19800 | 0.014290 |
| -0.19300 | 0.014580 |
| -0.18800 | 0.014940 |
| -0.18300 | 0.014850 |
| -0.17800 | 0.012700 |
| -0.17300 | 0.012360 |
| -0.16800 | 0.013630 |

| E (V) | i (nA) |
|---|---|
| -0.16300 | 0.013430 |
| -0.15800 | 0.013050 |
| -0.15300 | 0.013040 |
| -0.14800 | 0.013750 |
| -0.14300 | 0.013480 |
| -0.13800 | 0.012220 |
| -0.13300 | 0.012050 |
| -0.12800 | 0.013540 |
| -0.12300 | 0.013620 |
| -0.11800 | 0.012920 |
| -0.11300 | 0.012810 |
| -0.10800 | 0.014730 |
| -0.10300 | 0.014850 |
| -0.098000 | 0.012530 |
| -0.093000 | 0.012060 |
| -0.088000 | 0.013520 |
| -0.083000 | 0.013000 |
| -0.078000 | 0.011720 |
| -0.073000 | 0.012330 |
| -0.068000 | 0.013770 |
| -0.063000 | 0.013460 |
| -0.058000 | 0.010180 |
| -0.053000 | 0.0094300 |
| -0.048000 | 0.011300 |
| -0.043000 | 0.013400 |
| -0.038000 | 0.012050 |
| -0.033000 | 0.011570 |
| -0.028000 | 0.012810 |
| -0.023000 | 0.013460 |
| -0.018000 | 0.012630 |
| -0.013000 | 0.012090 |
| -0.0080000 | 0.014450 |
| -0.0030000 | 0.014220 |
| 0.0020000 | 0.013110 |
| 0.0070000 | 0.012520 |

| E (V) | i (nA) |
|---|---|
| 0.012000 | 0.014870 |
| 0.017000 | 0.014610 |
| 0.022000 | 0.013350 |
| 0.027000 | 0.013590 |
| 0.032000 | 0.014880 |
| 0.037000 | 0.014890 |
| 0.042000 | 0.013140 |
| 0.047000 | 0.013880 |
| 0.052000 | 0.014270 |
| 0.057000 | 0.015250 |
| 0.062000 | 0.012830 |
| 0.067000 | 0.013580 |
| 0.072000 | 0.014650 |
| 0.077000 | 0.015180 |
| 0.082000 | 0.013160 |
| 0.087000 | 0.013660 |
| 0.092000 | 0.016040 |
| 0.097000 | 0.015160 |
| 0.10200 | 0.014640 |
| 0.10700 | 0.012450 |
| 0.11200 | 0.016300 |
| 0.11700 | 0.014350 |
| 0.12200 | 0.014930 |
| 0.12700 | 0.012800 |
| 0.13200 | 0.015690 |
| 0.13700 | 0.016180 |
| 0.14200 | 0.015040 |
| 0.14700 | 0.015120 |
| 0.15200 | 0.011910 |
| 0.15700 | 0.017280 |
| 0.16200 | 0.010210 |
| 0.16700 | 0.018710 |
| 0.17200 | 0.0095600 |
| 0.17700 | 0.020160 |
| 0.18200 | 0.010080 |

| E (V) | i (nA) |
|---|---|
| 0.18700 | 0.017110 |
| 0.19200 | 0.027850 |
| 0.19700 | -0.013690 |
| 0.19800 | 0.15587 |
| 0.19300 | -0.041320 |
| 0.18800 | 0.024630 |
| 0.18300 | 0.0073000 |
| 0.17800 | 0.0078400 |
| 0.17300 | 0.010710 |
| 0.16800 | 0.0094400 |
| 0.16300 | 0.013460 |
| 0.15800 | 0.0061300 |
| 0.15300 | 0.0087200 |
| 0.14800 | 0.0083200 |
| 0.14300 | 0.0096600 |
| 0.13800 | 0.0063000 |
| 0.13300 | 0.0067600 |
| 0.12800 | 0.0087700 |
| 0.12300 | 0.0082600 |
| 0.11800 | 0.0067700 |
| 0.11300 | 0.0063300 |
| 0.10800 | 0.0091900 |
| 0.10300 | 0.0076900 |
| 0.098000 | 0.0059100 |
| 0.093000 | 0.0057800 |
| 0.088000 | 0.0077600 |
| 0.083000 | 0.0064500 |
| 0.078000 | 0.0040800 |
| 0.073000 | 0.0038600 |
| 0.068000 | 0.0058100 |
| 0.063000 | 0.0062800 |
| 0.058000 | 0.0036100 |
| 0.053000 | 0.0038700 |
| 0.048000 | 0.0064000 |
| 0.043000 | 0.0050800 |

| E (V) | i (nA) |
|---|---|
| 0.038000 | 9.2841E-4 |
| 0.033000 | 0.0025500 |
| 0.028000 | 0.0062100 |
| 0.023000 | 0.0062300 |
| 0.018000 | 0.0036800 |
| 0.013000 | 0.0033200 |
| 0.0080000 | 0.0050900 |
| 0.0030000 | 0.0050200 |
| -0.0020000 | 0.0022700 |
| -0.0070000 | 0.0015000 |
| -0.012000 | 0.0034700 |
| -0.017000 | 0.0047500 |
| -0.022000 | 0.0027600 |
| -0.027000 | 0.0028900 |
| -0.032000 | 0.0058700 |
| -0.037000 | 0.0041100 |
| -0.042000 | 0.0059400 |
| -0.047000 | -0.0061100 |
| -0.052000 | 0.053100 |
| -0.057000 | 0.23175 |
| -0.062000 | 0.34425 |
| -0.067000 | 0.35204 |
| -0.072000 | 0.34757 |
| -0.077000 | 0.33564 |
| -0.082000 | 0.32163 |
| -0.087000 | 0.30727 |
| -0.092000 | 0.29292 |
| -0.097000 | 0.27682 |
| -0.10200 | 0.25850 |
| -0.10700 | 0.24298 |
| -0.11200 | 0.22890 |
| -0.11700 | 0.21320 |
| -0.12200 | 0.19702 |
| -0.12700 | 0.18495 |
| -0.13200 | 0.17338 |

| E (V) | i (nA) |
|---|---|
| -0.13700 | 0.15958 |
| -0.14200 | 0.14771 |
| -0.14700 | 0.13887 |
| -0.15200 | 0.12608 |
| -0.15700 | 0.11108 |
| -0.16200 | 0.099600 |
| -0.16700 | 0.089530 |
| -0.17200 | 0.078820 |
| -0.17700 | 0.068140 |
| -0.18200 | 0.056530 |
| -0.18700 | 0.048440 |
| -0.19200 | 0.044090 |
| -0.19700 | 0.036710 |
| -0.20200 | 0.027730 |
| -0.20700 | 0.022150 |
| -0.21200 | 0.020280 |
| -0.21700 | 0.014600 |
| -0.22200 | 0.0079000 |
| -0.22700 | 0.0046900 |
| -0.23200 | 0.0045900 |
| -0.23700 | 0.0018700 |
| -0.24200 | -0.0036100 |
| -0.24700 | -0.0058100 |
| -0.25200 | -0.0054500 |
| -0.25700 | -0.0066200 |
| -0.26200 | -0.012250 |
| -0.26700 | -0.013420 |
| -0.27200 | -0.013110 |
| -0.27700 | -0.013890 |
| -0.28200 | -0.018410 |
| -0.28700 | -0.018870 |
| -0.29200 | -0.017080 |
| -0.29700 | -0.018820 |
| -0.30200 | -0.020810 |
| -0.30700 | -0.024200 |

| E (V) | i (nA) |
|---|---|
| -0.31200 | -0.022270 |
| -0.31700 | -0.027440 |
| -0.32200 | -0.025530 |
| -0.32700 | -0.028650 |
| -0.33200 | -0.023930 |
| -0.33700 | -0.029090 |
| -0.34200 | -0.033270 |
| -0.34700 | -0.012170 |

### 8.3.2 CV-2

| E (V) | i (nA) |
|---|---|
| -0.34800 | -0.075420 |
| -0.34300 | -0.067570 |
| -0.33800 | -0.013970 |
| -0.33300 | -0.028080 |
| -0.32800 | -0.017170 |
| -0.32300 | -0.0085900 |
| -0.31800 | -0.010120 |
| -0.31300 | -0.0032100 |
| -0.30800 | 0.0026400 |
| -0.30300 | 0.018610 |
| -0.29800 | 0.021400 |
| -0.29300 | 0.020340 |
| -0.28800 | 0.022150 |
| -0.28300 | 0.021570 |
| -0.27800 | 0.012680 |
| -0.27300 | 0.012770 |
| -0.26800 | 0.021650 |
| -0.26300 | 0.023230 |
| -0.25800 | 0.020510 |
| -0.25300 | 0.020260 |
| -0.24800 | 0.025890 |
| -0.24300 | 0.029060 |
| -0.23800 | 0.029850 |
| -0.23300 | 0.035690 |

| E (V) | i (nA) |
|---|---|
| -0.22800 | 0.047190 |
| -0.22300 | 0.050350 |
| -0.21800 | 0.054090 |
| -0.21300 | 0.065280 |
| -0.20800 | 0.070410 |
| -0.20300 | 0.067740 |
| -0.19800 | 0.063130 |
| -0.19300 | 0.062240 |
| -0.18800 | 0.060380 |
| -0.18300 | 0.060890 |
| -0.17800 | 0.065580 |
| -0.17300 | 0.074160 |
| -0.16800 | 0.087430 |
| -0.16300 | 0.097440 |
| -0.15800 | 0.10416 |
| -0.15300 | 0.11516 |
| -0.14800 | 0.12893 |
| -0.14300 | 0.13691 |
| -0.13800 | 0.14280 |
| -0.13300 | 0.15327 |
| -0.12800 | 0.16610 |
| -0.12300 | 0.17396 |
| -0.11800 | 0.17523 |
| -0.11300 | 0.17725 |
| -0.10800 | 0.18446 |
| -0.10300 | 0.19239 |
| -0.098000 | 0.19687 |
| -0.093000 | 0.20097 |
| -0.088000 | 0.20528 |
| -0.083000 | 0.20704 |
| -0.078000 | 0.20594 |
| -0.073000 | 0.20816 |
| -0.068000 | 0.21389 |
| -0.063000 | 0.21508 |
| -0.058000 | 0.21291 |

| E (V) | i (nA) |
|---|---|
| -0.053000 | 0.21466 |
| -0.048000 | 0.21858 |
| -0.043000 | 0.21732 |
| -0.038000 | 0.21332 |
| -0.033000 | 0.21258 |
| -0.028000 | 0.21516 |
| -0.023000 | 0.21489 |
| -0.018000 | 0.21153 |
| -0.013000 | 0.21108 |
| -0.0080000 | 0.21351 |
| -0.0030000 | 0.21351 |
| 0.0020000 | 0.20998 |
| 0.0070000 | 0.20946 |
| 0.012000 | 0.21172 |
| 0.017000 | 0.21133 |
| 0.022000 | 0.20852 |
| 0.027000 | 0.20902 |
| 0.032000 | 0.21134 |
| 0.037000 | 0.21025 |
| 0.042000 | 0.20712 |
| 0.047000 | 0.20792 |
| 0.052000 | 0.21166 |
| 0.057000 | 0.21109 |
| 0.062000 | 0.20821 |
| 0.067000 | 0.20804 |
| 0.072000 | 0.21187 |
| 0.077000 | 0.21172 |
| 0.082000 | 0.20802 |
| 0.087000 | 0.20791 |
| 0.092000 | 0.21209 |
| 0.097000 | 0.21307 |
| 0.10200 | 0.20982 |
| 0.10700 | 0.21105 |
| 0.11200 | 0.21422 |
| 0.11700 | 0.21591 |

| E (V) | i (nA) |
|---|---|
| 0.12200 | 0.21432 |
| 0.12700 | 0.21713 |
| 0.13200 | 0.21970 |
| 0.13700 | 0.21965 |
| 0.14200 | 0.21678 |
| 0.14700 | 0.21596 |
| 0.15200 | 0.21991 |
| 0.15700 | 0.21866 |
| 0.16200 | 0.21725 |
| 0.16700 | 0.21383 |
| 0.17200 | 0.22087 |
| 0.17700 | 0.21739 |
| 0.18200 | 0.21721 |
| 0.18700 | 0.21636 |
| 0.19200 | 0.21958 |
| 0.19700 | 0.23171 |
| 0.19800 | 0.16349 |
| 0.19300 | 0.16700 |
| 0.18800 | 0.21852 |
| 0.18300 | 0.20687 |
| 0.17800 | 0.20527 |
| 0.17300 | 0.20724 |
| 0.16800 | 0.20826 |
| 0.16300 | 0.21209 |
| 0.15800 | 0.20606 |
| 0.15300 | 0.20877 |
| 0.14800 | 0.21006 |
| 0.14300 | 0.21098 |
| 0.13800 | 0.20590 |
| 0.13300 | 0.20529 |
| 0.12800 | 0.20794 |
| 0.12300 | 0.20659 |
| 0.11800 | 0.20297 |
| 0.11300 | 0.20150 |
| 0.10800 | 0.20377 |

| E (V) | i (nA) |
|---|---|
| 0.10300 | 0.20152 |
| 0.098000 | 0.19725 |
| 0.093000 | 0.19489 |
| 0.088000 | 0.19677 |
| 0.083000 | 0.19505 |
| 0.078000 | 0.19005 |
| 0.073000 | 0.18902 |
| 0.068000 | 0.19200 |
| 0.063000 | 0.19150 |
| 0.058000 | 0.18684 |
| 0.053000 | 0.18663 |
| 0.048000 | 0.18927 |
| 0.043000 | 0.18867 |
| 0.038000 | 0.18407 |
| 0.033000 | 0.18364 |
| 0.028000 | 0.18657 |
| 0.023000 | 0.18579 |
| 0.018000 | 0.18195 |
| 0.013000 | 0.18068 |
| 0.0080000 | 0.18309 |
| 0.0030000 | 0.18238 |
| -0.0020000 | 0.17788 |
| -0.0070000 | 0.17633 |
| -0.012000 | 0.17842 |
| -0.017000 | 0.17702 |
| -0.022000 | 0.17215 |
| -0.027000 | 0.17024 |
| -0.032000 | 0.17152 |
| -0.037000 | 0.16781 |
| -0.042000 | 0.15918 |
| -0.047000 | 0.15414 |
| -0.052000 | 0.15277 |
| -0.057000 | 0.14963 |
| -0.062000 | 0.14484 |
| -0.067000 | 0.14515 |

| E (V) | i (nA) |
|---|---|
| -0.072000 | 0.14917 |
| -0.077000 | 0.15030 |
| -0.082000 | 0.14781 |
| -0.087000 | 0.14634 |
| -0.092000 | 0.14613 |
| -0.097000 | 0.14004 |
| -0.10200 | 0.12831 |
| -0.10700 | 0.11857 |
| -0.11200 | 0.11212 |
| -0.11700 | 0.10177 |
| -0.12200 | 0.088020 |
| -0.12700 | 0.078370 |
| -0.13200 | 0.071650 |
| -0.13700 | 0.060260 |
| -0.14200 | 0.045750 |
| -0.14700 | 0.034970 |
| -0.15200 | 0.028150 |
| -0.15700 | 0.020470 |
| -0.16200 | 0.010070 |
| -0.16700 | 0.0031600 |
| -0.17200 | -0.0010300 |
| -0.17700 | -0.0083200 |
| -0.18200 | -0.018760 |
| -0.18700 | -0.025330 |
| -0.19200 | -0.027210 |
| -0.19700 | -0.032340 |
| -0.20200 | -0.040760 |
| -0.20700 | -0.045280 |
| -0.21200 | -0.045420 |
| -0.21700 | -0.049720 |
| -0.22200 | -0.055800 |
| -0.22700 | -0.058560 |
| -0.23200 | -0.057680 |
| -0.23700 | -0.059590 |
| -0.24200 | -0.064620 |

| E (V) | i (nA) |
|---|---|
| -0.24700 | -0.066030 |
| -0.25200 | -0.064640 |
| -0.25700 | -0.065080 |
| -0.26200 | -0.069830 |
| -0.26700 | -0.070060 |
| -0.27200 | -0.067700 |
| -0.27700 | -0.067650 |
| -0.28200 | -0.071990 |
| -0.28700 | -0.071470 |
| -0.29200 | -0.067880 |
| -0.29700 | -0.068370 |
| -0.30200 | -0.070210 |
| -0.30700 | -0.070950 |
| -0.31200 | -0.065100 |
| -0.31700 | -0.067290 |
| -0.32200 | -0.067150 |
| -0.32700 | -0.070010 |
| -0.33200 | -0.063330 |
| -0.33700 | -0.064380 |
| -0.34200 | -0.070150 |
| -0.34700 | -0.056390 |

### 8.3.3 CV-3

| E (V) | i (nA) |
|---|---|
| -0.34800 | -0.10491 |
| -0.34300 | -0.085240 |
| -0.33800 | -0.046030 |
| -0.33300 | -0.052220 |
| -0.32800 | -0.043790 |
| -0.32300 | -0.039920 |
| -0.31800 | -0.043260 |
| -0.31300 | -0.037880 |
| -0.30800 | -0.034180 |
| -0.30300 | -0.029950 |
| -0.29800 | -0.032390 |

| E (V) | i (nA) |
|---|---|
| -0.29300 | -0.029580 |
| -0.28800 | -0.025720 |
| -0.28300 | -0.023940 |
| -0.27800 | -0.025620 |
| -0.27300 | -0.024240 |
| -0.26800 | -0.018940 |
| -0.26300 | -0.018340 |
| -0.25800 | -0.020340 |
| -0.25300 | -0.019790 |
| -0.24800 | -0.014800 |
| -0.24300 | -0.013940 |
| -0.23800 | -0.015610 |
| -0.23300 | -0.013000 |
| -0.22800 | -0.0064100 |
| -0.22300 | -0.0035900 |
| -0.21800 | -0.0037600 |
| -0.21300 | 3.7406E-4 |
| -0.20800 | 0.0075900 |
| -0.20300 | 0.012410 |
| -0.19800 | 0.014250 |
| -0.19300 | 0.019720 |
| -0.18800 | 0.028510 |
| -0.18300 | 0.035030 |
| -0.17800 | 0.038350 |
| -0.17300 | 0.045340 |
| -0.16800 | 0.056360 |
| -0.16300 | 0.064110 |
| -0.15800 | 0.069190 |
| -0.15300 | 0.078210 |
| -0.14800 | 0.090530 |
| -0.14300 | 0.099220 |
| -0.13800 | 0.10581 |
| -0.13300 | 0.11540 |
| -0.12800 | 0.12751 |
| -0.12300 | 0.13653 |

| E (V) | i (nA) |
|---|---|
| -0.11800 | 0.14199 |
| -0.11300 | 0.15009 |
| -0.10800 | 0.16102 |
| -0.10300 | 0.16845 |
| -0.098000 | 0.17204 |
| -0.093000 | 0.17804 |
| -0.088000 | 0.18668 |
| -0.083000 | 0.19163 |
| -0.078000 | 0.19175 |
| -0.073000 | 0.19506 |
| -0.068000 | 0.20140 |
| -0.063000 | 0.20315 |
| -0.058000 | 0.20152 |
| -0.053000 | 0.20294 |
| -0.048000 | 0.20729 |
| -0.043000 | 0.20763 |
| -0.038000 | 0.20443 |
| -0.033000 | 0.20428 |
| -0.028000 | 0.20750 |
| -0.023000 | 0.20805 |
| -0.018000 | 0.20523 |
| -0.013000 | 0.20494 |
| -0.0080000 | 0.20825 |
| -0.0030000 | 0.20815 |
| 0.0020000 | 0.20389 |
| 0.0070000 | 0.20375 |
| 0.012000 | 0.20700 |
| 0.017000 | 0.20646 |
| 0.022000 | 0.20257 |
| 0.027000 | 0.20293 |
| 0.032000 | 0.20630 |
| 0.037000 | 0.20604 |
| 0.042000 | 0.20256 |
| 0.047000 | 0.20213 |
| 0.052000 | 0.20557 |

| E (V) | i (nA) |
|---|---|
| 0.057000 | 0.20528 |
| 0.062000 | 0.20180 |
| 0.067000 | 0.20117 |
| 0.072000 | 0.20448 |
| 0.077000 | 0.20458 |
| 0.082000 | 0.20120 |
| 0.087000 | 0.20092 |
| 0.092000 | 0.20447 |
| 0.097000 | 0.20446 |
| 0.10200 | 0.20058 |
| 0.10700 | 0.20129 |
| 0.11200 | 0.20435 |
| 0.11700 | 0.20455 |
| 0.12200 | 0.20079 |
| 0.12700 | 0.20146 |
| 0.13200 | 0.20413 |
| 0.13700 | 0.20395 |
| 0.14200 | 0.20044 |
| 0.14700 | 0.19985 |
| 0.15200 | 0.20396 |
| 0.15700 | 0.20295 |
| 0.16200 | 0.20071 |
| 0.16700 | 0.19925 |
| 0.17200 | 0.20532 |
| 0.17700 | 0.20296 |
| 0.18200 | 0.20197 |
| 0.18700 | 0.20113 |
| 0.19200 | 0.20369 |
| 0.19700 | 0.21050 |
| 0.19800 | 0.16269 |
| 0.19300 | 0.17323 |
| 0.18800 | 0.19313 |
| 0.18300 | 0.18793 |
| 0.17800 | 0.18417 |
| 0.17300 | 0.18390 |

| E (V) | i (nA) |
|---|---|
| 0.16800 | 0.18531 |
| 0.16300 | 0.18614 |
| 0.15800 | 0.18114 |
| 0.15300 | 0.18155 |
| 0.14800 | 0.18390 |
| 0.14300 | 0.18375 |
| 0.13800 | 0.17958 |
| 0.13300 | 0.17992 |
| 0.12800 | 0.18293 |
| 0.12300 | 0.18195 |
| 0.11800 | 0.17857 |
| 0.11300 | 0.17812 |
| 0.10800 | 0.18106 |
| 0.10300 | 0.18011 |
| 0.098000 | 0.17660 |
| 0.093000 | 0.17644 |
| 0.088000 | 0.17972 |
| 0.083000 | 0.17923 |
| 0.078000 | 0.17526 |
| 0.073000 | 0.17489 |
| 0.068000 | 0.17856 |
| 0.063000 | 0.17858 |
| 0.058000 | 0.17460 |
| 0.053000 | 0.17439 |
| 0.048000 | 0.17718 |
| 0.043000 | 0.17685 |
| 0.038000 | 0.17303 |
| 0.033000 | 0.17275 |
| 0.028000 | 0.17564 |
| 0.023000 | 0.17513 |
| 0.018000 | 0.17176 |
| 0.013000 | 0.17143 |
| 0.0080000 | 0.17425 |
| 0.0030000 | 0.17388 |
| -0.0020000 | 0.16977 |

| E (V) | i (nA) |
|---|---|
| -0.0070000 | 0.16867 |
| -0.012000 | 0.17185 |
| -0.017000 | 0.17114 |
| -0.022000 | 0.16611 |
| -0.027000 | 0.16495 |
| -0.032000 | 0.16726 |
| -0.037000 | 0.16493 |
| -0.042000 | 0.15912 |
| -0.047000 | 0.15733 |
| -0.052000 | 0.15789 |
| -0.057000 | 0.15471 |
| -0.062000 | 0.14816 |
| -0.067000 | 0.14428 |
| -0.072000 | 0.14321 |
| -0.077000 | 0.13840 |
| -0.082000 | 0.12908 |
| -0.087000 | 0.12239 |
| -0.092000 | 0.11868 |
| -0.097000 | 0.11072 |
| -0.10200 | 0.098810 |
| -0.10700 | 0.089810 |
| -0.11200 | 0.083630 |
| -0.11700 | 0.073130 |
| -0.12200 | 0.059750 |
| -0.12700 | 0.049740 |
| -0.13200 | 0.042320 |
| -0.13700 | 0.031750 |
| -0.14200 | 0.018130 |
| -0.14700 | 0.0082900 |
| -0.15200 | 0.0017300 |
| -0.15700 | -0.0076900 |
| -0.16200 | -0.019900 |
| -0.16700 | -0.028560 |
| -0.17200 | -0.033180 |
| -0.17700 | -0.040380 |

| E (V) | i (nA) |
|---|---|
| -0.18200 | -0.051120 |
| -0.18700 | -0.057700 |
| -0.19200 | -0.059630 |
| -0.19700 | -0.064850 |
| -0.20200 | -0.073120 |
| -0.20700 | -0.077190 |
| -0.21200 | -0.077030 |
| -0.21700 | -0.080440 |
| -0.22200 | -0.086400 |
| -0.22700 | -0.088950 |
| -0.23200 | -0.087960 |
| -0.23700 | -0.089460 |
| -0.24200 | -0.094280 |
| -0.24700 | -0.095860 |
| -0.25200 | -0.093780 |
| -0.25700 | -0.094460 |
| -0.26200 | -0.098940 |
| -0.26700 | -0.099400 |
| -0.27200 | -0.096880 |
| -0.27700 | -0.097320 |
| -0.28200 | -0.10159 |
| -0.28700 | -0.10169 |
| -0.29200 | -0.098300 |
| -0.29700 | -0.098920 |
| -0.30200 | -0.10226 |
| -0.30700 | -0.10294 |
| -0.31200 | -0.098770 |
| -0.31700 | -0.10011 |
| -0.32200 | -0.10255 |
| -0.32700 | -0.10390 |
| -0.33200 | -0.099620 |
| -0.33700 | -0.099800 |
| -0.34200 | -0.10449 |
| -0.34700 | -0.098670 |

### 8.3.4 CV-4

| E (V) | i (nA) |
|---|---|
| -0.34800 | -0.11322 |
| -0.34300 | -0.097870 |
| -0.33800 | -0.086120 |
| -0.33300 | -0.085950 |
| -0.32800 | -0.079650 |
| -0.32300 | -0.077390 |
| -0.31800 | -0.079410 |
| -0.31300 | -0.076750 |
| -0.30800 | -0.072490 |
| -0.30300 | -0.070640 |
| -0.29800 | -0.072750 |
| -0.29300 | -0.071050 |
| -0.28800 | -0.066790 |
| -0.28300 | -0.065280 |
| -0.27800 | -0.067240 |
| -0.27300 | -0.065830 |
| -0.26800 | -0.060660 |
| -0.26300 | -0.059200 |
| -0.25800 | -0.060940 |
| -0.25300 | -0.059250 |
| -0.24800 | -0.053460 |
| -0.24300 | -0.051380 |
| -0.23800 | -0.052440 |
| -0.23300 | -0.049550 |
| -0.22800 | -0.042950 |
| -0.22300 | -0.039450 |
| -0.21800 | -0.038980 |
| -0.21300 | -0.034790 |
| -0.20800 | -0.026960 |
| -0.20300 | -0.021600 |
| -0.19800 | -0.019270 |
| -0.19300 | -0.013670 |
| -0.18800 | -0.0037900 |
| -0.18300 | 0.0037400 |

| E (V) | i (nA) |
|---|---|
| -0.17800 | 0.0076900 |
| -0.17300 | 0.015860 |
| -0.16800 | 0.028070 |
| -0.16300 | 0.036870 |
| -0.15800 | 0.043000 |
| -0.15300 | 0.053080 |
| -0.14800 | 0.066810 |
| -0.14300 | 0.076980 |
| -0.13800 | 0.084040 |
| -0.13300 | 0.094820 |
| -0.12800 | 0.10868 |
| -0.12300 | 0.11905 |
| -0.11800 | 0.12568 |
| -0.11300 | 0.13479 |
| -0.10800 | 0.14725 |
| -0.10300 | 0.15591 |
| -0.098000 | 0.15979 |
| -0.093000 | 0.16650 |
| -0.088000 | 0.17621 |
| -0.083000 | 0.18142 |
| -0.078000 | 0.18231 |
| -0.073000 | 0.18625 |
| -0.068000 | 0.19269 |
| -0.063000 | 0.19507 |
| -0.058000 | 0.19402 |
| -0.053000 | 0.19574 |
| -0.048000 | 0.20006 |
| -0.043000 | 0.20059 |
| -0.038000 | 0.19786 |
| -0.033000 | 0.19832 |
| -0.028000 | 0.20195 |
| -0.023000 | 0.20199 |
| -0.018000 | 0.19849 |
| -0.013000 | 0.19862 |
| -0.0080000 | 0.20216 |

| E (V) | i (nA) |
|---|---|
| -0.0030000 | 0.20196 |
| 0.0020000 | 0.19836 |
| 0.0070000 | 0.19863 |
| 0.012000 | 0.20191 |
| 0.017000 | 0.20120 |
| 0.022000 | 0.19725 |
| 0.027000 | 0.19700 |
| 0.032000 | 0.20011 |
| 0.037000 | 0.19983 |
| 0.042000 | 0.19615 |
| 0.047000 | 0.19586 |
| 0.052000 | 0.19890 |
| 0.057000 | 0.19884 |
| 0.062000 | 0.19581 |
| 0.067000 | 0.19543 |
| 0.072000 | 0.19864 |
| 0.077000 | 0.19863 |
| 0.082000 | 0.19501 |
| 0.087000 | 0.19508 |
| 0.092000 | 0.19846 |
| 0.097000 | 0.19802 |
| 0.10200 | 0.19430 |
| 0.10700 | 0.19446 |
| 0.11200 | 0.19759 |
| 0.11700 | 0.19750 |
| 0.12200 | 0.19422 |
| 0.12700 | 0.19455 |
| 0.13200 | 0.19785 |
| 0.13700 | 0.19798 |
| 0.14200 | 0.19458 |
| 0.14700 | 0.19450 |
| 0.15200 | 0.19824 |
| 0.15700 | 0.19823 |
| 0.16200 | 0.19494 |
| 0.16700 | 0.19435 |

| E (V) | i (nA) |
|---|---|
| 0.17200 | 0.19867 |
| 0.17700 | 0.19788 |
| 0.18200 | 0.19485 |
| 0.18700 | 0.19453 |
| 0.19200 | 0.19805 |
| 0.19700 | 0.19831 |
| 0.19800 | 0.17960 |
| 0.19300 | 0.17781 |
| 0.18800 | 0.18375 |
| 0.18300 | 0.18212 |
| 0.17800 | 0.17796 |
| 0.17300 | 0.17734 |
| 0.16800 | 0.17960 |
| 0.16300 | 0.17938 |
| 0.15800 | 0.17478 |
| 0.15300 | 0.17471 |
| 0.14800 | 0.17751 |
| 0.14300 | 0.17668 |
| 0.13800 | 0.17279 |
| 0.13300 | 0.17294 |
| 0.12800 | 0.17599 |
| 0.12300 | 0.17514 |
| 0.11800 | 0.17139 |
| 0.11300 | 0.17124 |
| 0.10800 | 0.17440 |
| 0.10300 | 0.17391 |
| 0.098000 | 0.17069 |
| 0.093000 | 0.17066 |
| 0.088000 | 0.17347 |
| 0.083000 | 0.17286 |
| 0.078000 | 0.16906 |
| 0.073000 | 0.16884 |
| 0.068000 | 0.17199 |
| 0.063000 | 0.17182 |
| 0.058000 | 0.16814 |

| E (V) | i (nA) |
|---|---|
| 0.053000 | 0.16779 |
| 0.048000 | 0.17081 |
| 0.043000 | 0.17028 |
| 0.038000 | 0.16676 |
| 0.033000 | 0.16698 |
| 0.028000 | 0.16958 |
| 0.023000 | 0.16867 |
| 0.018000 | 0.16519 |
| 0.013000 | 0.16480 |
| 0.0080000 | 0.16757 |
| 0.0030000 | 0.16704 |
| -0.0020000 | 0.16240 |
| -0.0070000 | 0.16121 |
| -0.012000 | 0.16435 |
| -0.017000 | 0.16326 |
| -0.022000 | 0.15820 |
| -0.027000 | 0.15707 |
| -0.032000 | 0.15901 |
| -0.037000 | 0.15688 |
| -0.042000 | 0.15087 |
| -0.047000 | 0.14834 |
| -0.052000 | 0.14865 |
| -0.057000 | 0.14487 |
| -0.062000 | 0.13751 |
| -0.067000 | 0.13265 |
| -0.072000 | 0.13074 |
| -0.077000 | 0.12514 |
| -0.082000 | 0.11491 |
| -0.087000 | 0.10743 |
| -0.092000 | 0.10275 |
| -0.097000 | 0.093720 |
| -0.10200 | 0.080780 |
| -0.10700 | 0.070700 |
| -0.11200 | 0.063400 |
| -0.11700 | 0.052010 |

| E (V) | i (nA) |
|---|---|
| -0.12200 | 0.037020 |
| -0.12700 | 0.025770 |
| -0.13200 | 0.017710 |
| -0.13700 | 0.0058500 |
| -0.14200 | -0.0088400 |
| -0.14700 | -0.019580 |
| -0.15200 | -0.026620 |
| -0.15700 | -0.036700 |
| -0.16200 | -0.050120 |
| -0.16700 | -0.059270 |
| -0.17200 | -0.063840 |
| -0.17700 | -0.071250 |
| -0.18200 | -0.082100 |
| -0.18700 | -0.088910 |
| -0.19200 | -0.091120 |
| -0.19700 | -0.096350 |
| -0.20200 | -0.10463 |
| -0.20700 | -0.10879 |
| -0.21200 | -0.10873 |
| -0.21700 | -0.11185 |
| -0.22200 | -0.11784 |
| -0.22700 | -0.11999 |
| -0.23200 | -0.11862 |
| -0.23700 | -0.12025 |
| -0.24200 | -0.12484 |
| -0.24700 | -0.12609 |
| -0.25200 | -0.12386 |
| -0.25700 | -0.12450 |
| -0.26200 | -0.12875 |
| -0.26700 | -0.12899 |
| -0.27200 | -0.12568 |
| -0.27700 | -0.12617 |
| -0.28200 | -0.13018 |
| -0.28700 | -0.13003 |
| -0.29200 | -0.12635 |

| E (V) | i (nA) |
|---|---|
| -0.29700 | -0.12678 |
| -0.30200 | -0.12991 |
| -0.30700 | -0.12986 |
| -0.31200 | -0.12651 |
| -0.31700 | -0.12687 |
| -0.32200 | -0.12963 |
| -0.32700 | -0.13017 |
| -0.33200 | -0.12655 |
| -0.33700 | -0.12632 |
| -0.34200 | -0.13046 |
| -0.34700 | -0.12711 |

### 8.3.5 CV-5

| E (V) | i (nA) |
|---|---|
| -0.34800 | -0.12780 |
| -0.34300 | -0.11571 |
| -0.33800 | -0.11220 |
| -0.33300 | -0.10957 |
| -0.32800 | -0.10343 |
| -0.32300 | -0.10167 |
| -0.31800 | -0.10350 |
| -0.31300 | -0.10168 |
| -0.30800 | -0.097030 |
| -0.30300 | -0.095730 |
| -0.29800 | -0.098020 |
| -0.29300 | -0.096710 |
| -0.28800 | -0.091930 |
| -0.28300 | -0.090240 |
| -0.27800 | -0.092350 |
| -0.27300 | -0.091270 |
| -0.26800 | -0.086060 |
| -0.26300 | -0.084210 |
| -0.25800 | -0.086300 |
| -0.25300 | -0.084470 |
| -0.24800 | -0.078890 |

| E (V) | i (nA) |
|---|---|
| -0.24300 | -0.076880 |
| -0.23800 | -0.077640 |
| -0.23300 | -0.074840 |
| -0.22800 | -0.068780 |
| -0.22300 | -0.065470 |
| -0.21800 | -0.064720 |
| -0.21300 | -0.060700 |
| -0.20800 | -0.052940 |
| -0.20300 | -0.047420 |
| -0.19800 | -0.044960 |
| -0.19300 | -0.038640 |
| -0.18800 | -0.028210 |
| -0.18300 | -0.020700 |
| -0.17800 | -0.016150 |
| -0.17300 | -0.0076600 |
| -0.16800 | 0.0051300 |
| -0.16300 | 0.014760 |
| -0.15800 | 0.021240 |
| -0.15300 | 0.032340 |
| -0.14800 | 0.046830 |
| -0.14300 | 0.057730 |
| -0.13800 | 0.066050 |
| -0.13300 | 0.077880 |
| -0.12800 | 0.092890 |
| -0.12300 | 0.10441 |
| -0.11800 | 0.11208 |
| -0.11300 | 0.12232 |
| -0.10800 | 0.13567 |
| -0.10300 | 0.14529 |
| -0.098000 | 0.15013 |
| -0.093000 | 0.15774 |
| -0.088000 | 0.16803 |
| -0.083000 | 0.17361 |
| -0.078000 | 0.17536 |
| -0.073000 | 0.17997 |

| E (V) | i (nA) |
|---|---|
| -0.068000 | 0.18681 |
| -0.063000 | 0.18928 |
| -0.058000 | 0.18845 |
| -0.053000 | 0.19045 |
| -0.048000 | 0.19478 |
| -0.043000 | 0.19599 |
| -0.038000 | 0.19336 |
| -0.033000 | 0.19359 |
| -0.028000 | 0.19752 |
| -0.023000 | 0.19738 |
| -0.018000 | 0.19360 |
| -0.013000 | 0.19371 |
| -0.0080000 | 0.19708 |
| -0.0030000 | 0.19690 |
| 0.0020000 | 0.19336 |
| 0.0070000 | 0.19349 |
| 0.012000 | 0.19687 |
| 0.017000 | 0.19611 |
| 0.022000 | 0.19258 |
| 0.027000 | 0.19273 |
| 0.032000 | 0.19537 |
| 0.037000 | 0.19506 |
| 0.042000 | 0.19173 |
| 0.047000 | 0.19139 |
| 0.052000 | 0.19442 |
| 0.057000 | 0.19410 |
| 0.062000 | 0.19052 |
| 0.067000 | 0.19046 |
| 0.072000 | 0.19397 |
| 0.077000 | 0.19397 |
| 0.082000 | 0.19043 |
| 0.087000 | 0.19049 |
| 0.092000 | 0.19389 |
| 0.097000 | 0.19352 |
| 0.10200 | 0.18985 |

| E (V) | i (nA) |
|---|---|
| 0.10700 | 0.18999 |
| 0.11200 | 0.19324 |
| 0.11700 | 0.19288 |
| 0.12200 | 0.18944 |
| 0.12700 | 0.18934 |
| 0.13200 | 0.19259 |
| 0.13700 | 0.19278 |
| 0.14200 | 0.18933 |
| 0.14700 | 0.18909 |
| 0.15200 | 0.19277 |
| 0.15700 | 0.19319 |
| 0.16200 | 0.18940 |
| 0.16700 | 0.18919 |
| 0.17200 | 0.19304 |
| 0.17700 | 0.19268 |
| 0.18200 | 0.18931 |
| 0.18700 | 0.18933 |
| 0.19200 | 0.19303 |
| 0.19700 | 0.19096 |
| 0.19800 | 0.18057 |
| 0.19300 | 0.18012 |
| 0.18800 | 0.17590 |
| 0.18300 | 0.17641 |
| 0.17800 | 0.17186 |
| 0.17300 | 0.17102 |
| 0.16800 | 0.17416 |
| 0.16300 | 0.17365 |
| 0.15800 | 0.16963 |
| 0.15300 | 0.16898 |
| 0.14800 | 0.17223 |
| 0.14300 | 0.17137 |
| 0.13800 | 0.16756 |
| 0.13300 | 0.16728 |
| 0.12800 | 0.17042 |
| 0.12300 | 0.16998 |

| E (V) | i (nA) |
|---|---|
| 0.11800 | 0.16632 |
| 0.11300 | 0.16607 |
| 0.10800 | 0.16895 |
| 0.10300 | 0.16902 |
| 0.098000 | 0.16592 |
| 0.093000 | 0.16560 |
| 0.088000 | 0.16807 |
| 0.083000 | 0.16786 |
| 0.078000 | 0.16432 |
| 0.073000 | 0.16368 |
| 0.068000 | 0.16671 |
| 0.063000 | 0.16637 |
| 0.058000 | 0.16258 |
| 0.053000 | 0.16242 |
| 0.048000 | 0.16544 |
| 0.043000 | 0.16476 |
| 0.038000 | 0.16115 |
| 0.033000 | 0.16100 |
| 0.028000 | 0.16391 |
| 0.023000 | 0.16334 |
| 0.018000 | 0.15944 |
| 0.013000 | 0.15878 |
| 0.0080000 | 0.16136 |
| 0.0030000 | 0.16067 |
| -0.0020000 | 0.15611 |
| -0.0070000 | 0.15482 |
| -0.012000 | 0.15755 |
| -0.017000 | 0.15648 |
| -0.022000 | 0.15123 |
| -0.027000 | 0.14957 |
| -0.032000 | 0.15132 |
| -0.037000 | 0.14902 |
| -0.042000 | 0.14305 |
| -0.047000 | 0.14026 |
| -0.052000 | 0.14003 |

| E (V) | i (nA) |
|---|---|
| -0.057000 | 0.13589 |
| -0.062000 | 0.12816 |
| -0.067000 | 0.12287 |
| -0.072000 | 0.12007 |
| -0.077000 | 0.11326 |
| -0.082000 | 0.10241 |
| -0.087000 | 0.094220 |
| -0.092000 | 0.088740 |
| -0.097000 | 0.078790 |
| -0.10200 | 0.064040 |
| -0.10700 | 0.052880 |
| -0.11200 | 0.045130 |
| -0.11700 | 0.032680 |
| -0.12200 | 0.016670 |
| -0.12700 | 0.0042000 |
| -0.13200 | -0.0052000 |
| -0.13700 | -0.017850 |
| -0.14200 | -0.033510 |
| -0.14700 | -0.045370 |
| -0.15200 | -0.053180 |
| -0.15700 | -0.063870 |
| -0.16200 | -0.077460 |
| -0.16700 | -0.086840 |
| -0.17200 | -0.092140 |
| -0.17700 | -0.099940 |
| -0.18200 | -0.11039 |
| -0.18700 | -0.11703 |
| -0.19200 | -0.11955 |
| -0.19700 | -0.12515 |
| -0.20200 | -0.13302 |
| -0.20700 | -0.13647 |
| -0.21200 | -0.13708 |
| -0.21700 | -0.14043 |
| -0.22200 | -0.14594 |
| -0.22700 | -0.14784 |

| E (V) | i (nA) |
|---|---|
| -0.23200 | -0.14616 |
| -0.23700 | -0.14749 |
| -0.24200 | -0.15204 |
| -0.24700 | -0.15304 |
| -0.25200 | -0.14999 |
| -0.25700 | -0.15024 |
| -0.26200 | -0.15423 |
| -0.26700 | -0.15447 |
| -0.27200 | -0.15119 |
| -0.27700 | -0.15184 |
| -0.28200 | -0.15574 |
| -0.28700 | -0.15552 |
| -0.29200 | -0.15148 |
| -0.29700 | -0.15138 |
| -0.30200 | -0.15450 |
| -0.30700 | -0.15406 |
| -0.31200 | -0.15061 |
| -0.31700 | -0.14986 |
| -0.32200 | -0.15291 |
| -0.32700 | -0.15293 |
| -0.33200 | -0.14980 |
| -0.33700 | -0.14968 |
| -0.34200 | -0.15368 |
| -0.34700 | -0.15205 |

### 8.3.6 CV-6

| E (V) | i (nA) |
|---|---|
| -0.34800 | -0.14280 |
| -0.34300 | -0.13338 |
| -0.33800 | -0.13580 |
| -0.33300 | -0.13228 |
| -0.32800 | -0.12628 |
| -0.32300 | -0.12488 |
| -0.31800 | -0.12622 |
| -0.31300 | -0.12447 |

| E (V) | i (nA) |
|---|---|
| -0.30800 | -0.11964 |
| -0.30300 | -0.11855 |
| -0.29800 | -0.12033 |
| -0.29300 | -0.11904 |
| -0.28800 | -0.11425 |
| -0.28300 | -0.11261 |
| -0.27800 | -0.11501 |
| -0.27300 | -0.11432 |
| -0.26800 | -0.10936 |
| -0.26300 | -0.10776 |
| -0.25800 | -0.10979 |
| -0.25300 | -0.10751 |
| -0.24800 | -0.10184 |
| -0.24300 | -0.099690 |
| -0.23800 | -0.10091 |
| -0.23300 | -0.098110 |
| -0.22800 | -0.091380 |
| -0.22300 | -0.088010 |
| -0.21800 | -0.087350 |
| -0.21300 | -0.083110 |
| -0.20800 | -0.075220 |
| -0.20300 | -0.069860 |
| -0.19800 | -0.067510 |
| -0.19300 | -0.060950 |
| -0.18800 | -0.050040 |
| -0.18300 | -0.042290 |
| -0.17800 | -0.037460 |
| -0.17300 | -0.028230 |
| -0.16800 | -0.014680 |
| -0.16300 | -0.0043700 |
| -0.15800 | 0.0028400 |
| -0.15300 | 0.014440 |
| -0.14800 | 0.029940 |
| -0.14300 | 0.042010 |
| -0.13800 | 0.050820 |

| E (V) | i (nA) |
|---|---|
| -0.13300 | 0.063420 |
| -0.12800 | 0.079260 |
| -0.12300 | 0.091610 |
| -0.11800 | 0.10029 |
| -0.11300 | 0.11127 |
| -0.10800 | 0.12559 |
| -0.10300 | 0.13643 |
| -0.098000 | 0.14205 |
| -0.093000 | 0.15011 |
| -0.088000 | 0.16155 |
| -0.083000 | 0.16820 |
| -0.078000 | 0.17009 |
| -0.073000 | 0.17485 |
| -0.068000 | 0.18213 |
| -0.063000 | 0.18505 |
| -0.058000 | 0.18418 |
| -0.053000 | 0.18649 |
| -0.048000 | 0.19142 |
| -0.043000 | 0.19250 |
| -0.038000 | 0.19006 |
| -0.033000 | 0.19038 |
| -0.028000 | 0.19420 |
| -0.023000 | 0.19484 |
| -0.018000 | 0.19119 |
| -0.013000 | 0.19113 |
| -0.0080000 | 0.19504 |
| -0.0030000 | 0.19501 |
| 0.0020000 | 0.19124 |
| 0.0070000 | 0.19094 |
| 0.012000 | 0.19402 |
| 0.017000 | 0.19373 |
| 0.022000 | 0.19030 |
| 0.027000 | 0.19013 |
| 0.032000 | 0.19329 |
| 0.037000 | 0.19320 |

| E (V) | i (nA) |
|---|---|
| 0.042000 | 0.18947 |
| 0.047000 | 0.18900 |
| 0.052000 | 0.19221 |
| 0.057000 | 0.19219 |
| 0.062000 | 0.18831 |
| 0.067000 | 0.18769 |
| 0.072000 | 0.19087 |
| 0.077000 | 0.19075 |
| 0.082000 | 0.18691 |
| 0.087000 | 0.18724 |
| 0.092000 | 0.19112 |
| 0.097000 | 0.19051 |
| 0.10200 | 0.18700 |
| 0.10700 | 0.18717 |
| 0.11200 | 0.19025 |
| 0.11700 | 0.19001 |
| 0.12200 | 0.18650 |
| 0.12700 | 0.18617 |
| 0.13200 | 0.18939 |
| 0.13700 | 0.18939 |
| 0.14200 | 0.18596 |
| 0.14700 | 0.18567 |
| 0.15200 | 0.18922 |
| 0.15700 | 0.18955 |
| 0.16200 | 0.18559 |
| 0.16700 | 0.18604 |
| 0.17200 | 0.18934 |
| 0.17700 | 0.18923 |
| 0.18200 | 0.18582 |
| 0.18700 | 0.18613 |
| 0.19200 | 0.19010 |
| 0.19700 | 0.18661 |
| 0.19800 | 0.18187 |
| 0.19300 | 0.17727 |
| 0.18800 | 0.17257 |

| E (V) | i (nA) |
|---|---|
| 0.18300 | 0.17269 |
| 0.17800 | 0.16819 |
| 0.17300 | 0.16790 |
| 0.16800 | 0.17114 |
| 0.16300 | 0.17059 |
| 0.15800 | 0.16656 |
| 0.15300 | 0.16565 |
| 0.14800 | 0.16883 |
| 0.14300 | 0.16798 |
| 0.13800 | 0.16415 |
| 0.13300 | 0.16410 |
| 0.12800 | 0.16697 |
| 0.12300 | 0.16640 |
| 0.11800 | 0.16293 |
| 0.11300 | 0.16262 |
| 0.10800 | 0.16543 |
| 0.10300 | 0.16508 |
| 0.098000 | 0.16164 |
| 0.093000 | 0.16162 |
| 0.088000 | 0.16460 |
| 0.083000 | 0.16420 |
| 0.078000 | 0.16030 |
| 0.073000 | 0.16007 |
| 0.068000 | 0.16315 |
| 0.063000 | 0.16238 |
| 0.058000 | 0.15848 |
| 0.053000 | 0.15838 |
| 0.048000 | 0.16160 |
| 0.043000 | 0.16071 |
| 0.038000 | 0.15657 |
| 0.033000 | 0.15657 |
| 0.028000 | 0.15950 |
| 0.023000 | 0.15905 |
| 0.018000 | 0.15548 |
| 0.013000 | 0.15451 |

| E (V) | i (nA) |
|---|---|
| 0.0080000 | 0.15724 |
| 0.0030000 | 0.15677 |
| -0.0020000 | 0.15221 |
| -0.0070000 | 0.15098 |
| -0.012000 | 0.15315 |
| -0.017000 | 0.15192 |
| -0.022000 | 0.14687 |
| -0.027000 | 0.14498 |
| -0.032000 | 0.14639 |
| -0.037000 | 0.14364 |
| -0.042000 | 0.13757 |
| -0.047000 | 0.13469 |
| -0.052000 | 0.13377 |
| -0.057000 | 0.12923 |
| -0.062000 | 0.12114 |
| -0.067000 | 0.11517 |
| -0.072000 | 0.11177 |
| -0.077000 | 0.10415 |
| -0.082000 | 0.092100 |
| -0.087000 | 0.082950 |
| -0.092000 | 0.076460 |
| -0.097000 | 0.065420 |
| -0.10200 | 0.050100 |
| -0.10700 | 0.037780 |
| -0.11200 | 0.028570 |
| -0.11700 | 0.015240 |
| -0.12200 | -0.0017900 |
| -0.12700 | -0.015240 |
| -0.13200 | -0.025340 |
| -0.13700 | -0.038670 |
| -0.14200 | -0.055060 |
| -0.14700 | -0.067640 |
| -0.15200 | -0.076540 |
| -0.15700 | -0.087890 |
| -0.16200 | -0.10157 |

| E (V) | i (nA) |
|---|---|
| -0.16700 | -0.11160 |
| -0.17200 | -0.11726 |
| -0.17700 | -0.12541 |
| -0.18200 | -0.13616 |
| -0.18700 | -0.14242 |
| -0.19200 | -0.14490 |
| -0.19700 | -0.15041 |
| -0.20200 | -0.15831 |
| -0.20700 | -0.16183 |
| -0.21200 | -0.16170 |
| -0.21700 | -0.16472 |
| -0.22200 | -0.17015 |
| -0.22700 | -0.17172 |
| -0.23200 | -0.16992 |
| -0.23700 | -0.17111 |
| -0.24200 | -0.17559 |
| -0.24700 | -0.17630 |
| -0.25200 | -0.17284 |
| -0.25700 | -0.17275 |
| -0.26200 | -0.17679 |
| -0.26700 | -0.17716 |
| -0.27200 | -0.17336 |
| -0.27700 | -0.17339 |
| -0.28200 | -0.17678 |
| -0.28700 | -0.17656 |
| -0.29200 | -0.17301 |
| -0.29700 | -0.17308 |
| -0.30200 | -0.17634 |
| -0.30700 | -0.17589 |
| -0.31200 | -0.17231 |
| -0.31700 | -0.17213 |
| -0.32200 | -0.17514 |
| -0.32700 | -0.17448 |
| -0.33200 | -0.17122 |
| -0.33700 | -0.17084 |

| E (V) | i (nA) |
|---|---|
| -0.34200 | -0.17501 |
| -0.34700 | -0.17317 |

### 8.3.7 CV-7

| E (V) | i (nA) |
|---|---|
| -0.34800 | -0.16338 |
| -0.34300 | -0.15583 |
| -0.33800 | -0.15608 |
| -0.33300 | -0.15316 |
| -0.32800 | -0.14729 |
| -0.32300 | -0.14583 |
| -0.31800 | -0.14760 |
| -0.31300 | -0.14585 |
| -0.30800 | -0.14072 |
| -0.30300 | -0.13955 |
| -0.29800 | -0.14140 |
| -0.29300 | -0.14009 |
| -0.28800 | -0.13564 |
| -0.28300 | -0.13400 |
| -0.27800 | -0.13591 |
| -0.27300 | -0.13459 |
| -0.26800 | -0.12949 |
| -0.26300 | -0.12765 |
| -0.25800 | -0.12942 |
| -0.25300 | -0.12736 |
| -0.24800 | -0.12172 |
| -0.24300 | -0.11980 |
| -0.23800 | -0.12047 |
| -0.23300 | -0.11709 |
| -0.22800 | -0.11076 |
| -0.22300 | -0.10758 |
| -0.21800 | -0.10702 |
| -0.21300 | -0.10254 |
| -0.20800 | -0.094260 |
| -0.20300 | -0.088770 |

| E (V) | i (nA) |
|---|---|
| -0.19800 | -0.086260 |
| -0.19300 | -0.080060 |
| -0.18800 | -0.069110 |
| -0.18300 | -0.060730 |
| -0.17800 | -0.055760 |
| -0.17300 | -0.046320 |
| -0.16800 | -0.032640 |
| -0.16300 | -0.022130 |
| -0.15800 | -0.014110 |
| -0.15300 | -0.0019200 |
| -0.14800 | 0.013830 |
| -0.14300 | 0.026350 |
| -0.13800 | 0.036080 |
| -0.13300 | 0.049390 |
| -0.12800 | 0.065680 |
| -0.12300 | 0.078880 |
| -0.11800 | 0.088570 |
| -0.11300 | 0.10090 |
| -0.10800 | 0.11629 |
| -0.10300 | 0.12747 |
| -0.098000 | 0.13437 |
| -0.093000 | 0.14403 |
| -0.088000 | 0.15601 |
| -0.083000 | 0.16311 |
| -0.078000 | 0.16576 |
| -0.073000 | 0.17148 |
| -0.068000 | 0.17950 |
| -0.063000 | 0.18263 |
| -0.058000 | 0.18218 |
| -0.053000 | 0.18457 |
| -0.048000 | 0.18959 |
| -0.043000 | 0.19119 |
| -0.038000 | 0.18876 |
| -0.033000 | 0.18932 |
| -0.028000 | 0.19342 |

| E (V) | i (nA) |
|---|---|
| -0.023000 | 0.19413 |
| -0.018000 | 0.19094 |
| -0.013000 | 0.19085 |
| -0.0080000 | 0.19428 |
| -0.0030000 | 0.19409 |
| 0.0020000 | 0.19059 |
| 0.0070000 | 0.19045 |
| 0.012000 | 0.19334 |
| 0.017000 | 0.19265 |
| 0.022000 | 0.18906 |
| 0.027000 | 0.18899 |
| 0.032000 | 0.19183 |
| 0.037000 | 0.19158 |
| 0.042000 | 0.18804 |
| 0.047000 | 0.18776 |
| 0.052000 | 0.19111 |
| 0.057000 | 0.19086 |
| 0.062000 | 0.18680 |
| 0.067000 | 0.18663 |
| 0.072000 | 0.19021 |
| 0.077000 | 0.18972 |
| 0.082000 | 0.18562 |
| 0.087000 | 0.18553 |
| 0.092000 | 0.18900 |
| 0.097000 | 0.18860 |
| 0.10200 | 0.18515 |
| 0.10700 | 0.18521 |
| 0.11200 | 0.18784 |
| 0.11700 | 0.18759 |
| 0.12200 | 0.18475 |
| 0.12700 | 0.18455 |
| 0.13200 | 0.18768 |
| 0.13700 | 0.18779 |
| 0.14200 | 0.18437 |
| 0.14700 | 0.18432 |

| E (V) | i (nA) |
|---|---|
| 0.15200 | 0.18764 |
| 0.15700 | 0.18759 |
| 0.16200 | 0.18382 |
| 0.16700 | 0.18419 |
| 0.17200 | 0.18761 |
| 0.17700 | 0.18724 |
| 0.18200 | 0.18381 |
| 0.18700 | 0.18430 |
| 0.19200 | 0.18858 |
| 0.19700 | 0.18562 |
| 0.19800 | 0.17867 |
| 0.19300 | 0.17353 |
| 0.18800 | 0.17208 |
| 0.18300 | 0.17197 |
| 0.17800 | 0.16735 |
| 0.17300 | 0.16625 |
| 0.16800 | 0.16928 |
| 0.16300 | 0.16897 |
| 0.15800 | 0.16445 |
| 0.15300 | 0.16385 |
| 0.14800 | 0.16720 |
| 0.14300 | 0.16665 |
| 0.13800 | 0.16282 |
| 0.13300 | 0.16258 |
| 0.12800 | 0.16545 |
| 0.12300 | 0.16474 |
| 0.11800 | 0.16124 |
| 0.11300 | 0.16117 |
| 0.10800 | 0.16388 |
| 0.10300 | 0.16333 |
| 0.098000 | 0.15961 |
| 0.093000 | 0.15893 |
| 0.088000 | 0.16219 |
| 0.083000 | 0.16203 |
| 0.078000 | 0.15774 |

| E (V) | i (nA) |
|---|---|
| 0.073000 | 0.15750 |
| 0.068000 | 0.16092 |
| 0.063000 | 0.16055 |
| 0.058000 | 0.15659 |
| 0.053000 | 0.15617 |
| 0.048000 | 0.15941 |
| 0.043000 | 0.15891 |
| 0.038000 | 0.15490 |
| 0.033000 | 0.15441 |
| 0.028000 | 0.15717 |
| 0.023000 | 0.15688 |
| 0.018000 | 0.15316 |
| 0.013000 | 0.15219 |
| 0.0080000 | 0.15494 |
| 0.0030000 | 0.15435 |
| -0.0020000 | 0.14974 |
| -0.0070000 | 0.14863 |
| -0.012000 | 0.15100 |
| -0.017000 | 0.14945 |
| -0.022000 | 0.14434 |
| -0.027000 | 0.14252 |
| -0.032000 | 0.14329 |
| -0.037000 | 0.14016 |
| -0.042000 | 0.13383 |
| -0.047000 | 0.13049 |
| -0.052000 | 0.12936 |
| -0.057000 | 0.12414 |
| -0.062000 | 0.11515 |
| -0.067000 | 0.10877 |
| -0.072000 | 0.10479 |
| -0.077000 | 0.096410 |
| -0.082000 | 0.083620 |
| -0.087000 | 0.073320 |
| -0.092000 | 0.065750 |
| -0.097000 | 0.053670 |

| E (V) | i (nA) |
|---|---|
| -0.10200 | 0.037320 |
| -0.10700 | 0.024120 |
| -0.11200 | 0.013410 |
| -0.11700 | -0.0014200 |
| -0.12200 | -0.019160 |
| -0.12700 | -0.033410 |
| -0.13200 | -0.044760 |
| -0.13700 | -0.058910 |
| -0.14200 | -0.075540 |
| -0.14700 | -0.088680 |
| -0.15200 | -0.098100 |
| -0.15700 | -0.10997 |
| -0.16200 | -0.12404 |
| -0.16700 | -0.13386 |
| -0.17200 | -0.13974 |
| -0.17700 | -0.14818 |
| -0.18200 | -0.15920 |
| -0.18700 | -0.16562 |
| -0.19200 | -0.16772 |
| -0.19700 | -0.17290 |
| -0.20200 | -0.18062 |
| -0.20700 | -0.18408 |
| -0.21200 | -0.18351 |
| -0.21700 | -0.18579 |
| -0.22200 | -0.19112 |
| -0.22700 | -0.19281 |
| -0.23200 | -0.19067 |
| -0.23700 | -0.19159 |
| -0.24200 | -0.19586 |
| -0.24700 | -0.19648 |
| -0.25200 | -0.19336 |
| -0.25700 | -0.19328 |
| -0.26200 | -0.19678 |
| -0.26700 | -0.19687 |
| -0.27200 | -0.19322 |

| E (V) | i (nA) |
|---|---|
| -0.27700 | -0.19308 |
| -0.28200 | -0.19627 |
| -0.28700 | -0.19560 |
| -0.29200 | -0.19208 |
| -0.29700 | -0.19232 |
| -0.30200 | -0.19481 |
| -0.30700 | -0.19446 |
| -0.31200 | -0.19115 |
| -0.31700 | -0.19087 |
| -0.32200 | -0.19402 |
| -0.32700 | -0.19384 |
| -0.33200 | -0.19014 |
| -0.33700 | -0.18967 |
| -0.34200 | -0.19359 |
| -0.34700 | -0.19108 |

### 8.3.8 CV-8

| E (V) | i (nA) |
|---|---|
| -0.34800 | -0.18462 |
| -0.34300 | -0.17544 |
| -0.33800 | -0.17330 |
| -0.33300 | -0.17106 |
| -0.32800 | -0.16526 |
| -0.32300 | -0.16369 |
| -0.31800 | -0.16519 |
| -0.31300 | -0.16347 |
| -0.30800 | -0.15922 |
| -0.30300 | -0.15762 |
| -0.29800 | -0.15915 |
| -0.29300 | -0.15799 |
| -0.28800 | -0.15329 |
| -0.28300 | -0.15175 |
| -0.27800 | -0.15383 |
| -0.27300 | -0.15207 |
| -0.26800 | -0.14685 |

| E (V) | i (nA) |
|---|---|
| -0.26300 | -0.14527 |
| -0.25800 | -0.14704 |
| -0.25300 | -0.14480 |
| -0.24800 | -0.13910 |
| -0.24300 | -0.13736 |
| -0.23800 | -0.13835 |
| -0.23300 | -0.13518 |
| -0.22800 | -0.12875 |
| -0.22300 | -0.12550 |
| -0.21800 | -0.12499 |
| -0.21300 | -0.12077 |
| -0.20800 | -0.11257 |
| -0.20300 | -0.10687 |
| -0.19800 | -0.10415 |
| -0.19300 | -0.097570 |
| -0.18800 | -0.086450 |
| -0.18300 | -0.078080 |
| -0.17800 | -0.072860 |
| -0.17300 | -0.063160 |
| -0.16800 | -0.049290 |
| -0.16300 | -0.038340 |
| -0.15800 | -0.029830 |
| -0.15300 | -0.016990 |
| -0.14800 | -6.5498E-4 |
| -0.14300 | 0.012420 |
| -0.13800 | 0.023250 |
| -0.13300 | 0.037620 |
| -0.12800 | 0.054830 |
| -0.12300 | 0.068920 |
| -0.11800 | 0.079340 |
| -0.11300 | 0.092730 |
| -0.10800 | 0.10925 |
| -0.10300 | 0.12114 |
| -0.098000 | 0.12897 |
| -0.093000 | 0.13970 |

| E (V) | i (nA) |
|---|---|
| -0.088000 | 0.15229 |
| -0.083000 | 0.15995 |
| -0.078000 | 0.16341 |
| -0.073000 | 0.16988 |
| -0.068000 | 0.17849 |
| -0.063000 | 0.18240 |
| -0.058000 | 0.18239 |
| -0.053000 | 0.18490 |
| -0.048000 | 0.19008 |
| -0.043000 | 0.19178 |
| -0.038000 | 0.18977 |
| -0.033000 | 0.19047 |
| -0.028000 | 0.19419 |
| -0.023000 | 0.19459 |
| -0.018000 | 0.19140 |
| -0.013000 | 0.19121 |
| -0.0080000 | 0.19445 |
| -0.0030000 | 0.19393 |
| 0.0020000 | 0.19033 |
| 0.0070000 | 0.19084 |
| 0.012000 | 0.19398 |
| 0.017000 | 0.19314 |
| 0.022000 | 0.18946 |
| 0.027000 | 0.18943 |
| 0.032000 | 0.19246 |
| 0.037000 | 0.19201 |
| 0.042000 | 0.18814 |
| 0.047000 | 0.18754 |
| 0.052000 | 0.19080 |
| 0.057000 | 0.19096 |
| 0.062000 | 0.18713 |
| 0.067000 | 0.18678 |
| 0.072000 | 0.19034 |
| 0.077000 | 0.18990 |
| 0.082000 | 0.18595 |

| E (V) | i (nA) |
|---|---|
| 0.087000 | 0.18583 |
| 0.092000 | 0.18883 |
| 0.097000 | 0.18842 |
| 0.10200 | 0.18492 |
| 0.10700 | 0.18471 |
| 0.11200 | 0.18788 |
| 0.11700 | 0.18767 |
| 0.12200 | 0.18401 |
| 0.12700 | 0.18385 |
| 0.13200 | 0.18709 |
| 0.13700 | 0.18705 |
| 0.14200 | 0.18358 |
| 0.14700 | 0.18360 |
| 0.15200 | 0.18716 |
| 0.15700 | 0.18704 |
| 0.16200 | 0.18310 |
| 0.16700 | 0.18325 |
| 0.17200 | 0.18686 |
| 0.17700 | 0.18625 |
| 0.18200 | 0.18275 |
| 0.18700 | 0.18281 |
| 0.19200 | 0.18672 |
| 0.19700 | 0.18440 |
| 0.19800 | 0.17542 |
| 0.19300 | 0.16938 |
| 0.18800 | 0.16927 |
| 0.18300 | 0.16817 |
| 0.17800 | 0.16351 |
| 0.17300 | 0.16265 |
| 0.16800 | 0.16523 |
| 0.16300 | 0.16477 |
| 0.15800 | 0.15981 |
| 0.15300 | 0.15871 |
| 0.14800 | 0.16131 |
| 0.14300 | 0.16027 |

| E (V) | i (nA) |
|---|---|
| 0.13800 | 0.15651 |
| 0.13300 | 0.15656 |
| 0.12800 | 0.15983 |
| 0.12300 | 0.15961 |
| 0.11800 | 0.15629 |
| 0.11300 | 0.15649 |
| 0.10800 | 0.15986 |
| 0.10300 | 0.15993 |
| 0.098000 | 0.15630 |
| 0.093000 | 0.15541 |
| 0.088000 | 0.15851 |
| 0.083000 | 0.15816 |
| 0.078000 | 0.15351 |
| 0.073000 | 0.15221 |
| 0.068000 | 0.15451 |
| 0.063000 | 0.15341 |
| 0.058000 | 0.14903 |
| 0.053000 | 0.14846 |
| 0.048000 | 0.15108 |
| 0.043000 | 0.14973 |
| 0.038000 | 0.14510 |
| 0.033000 | 0.14346 |
| 0.028000 | 0.14416 |
| 0.023000 | 0.14230 |
| 0.018000 | 0.13912 |
| 0.013000 | 0.14069 |
| 0.0080000 | 0.14266 |
| 0.0030000 | 0.13552 |
| -0.0020000 | 0.12513 |
| -0.0070000 | 0.12161 |
| -0.012000 | 0.12199 |
| -0.017000 | 0.11691 |
| -0.022000 | 0.10715 |
| -0.027000 | 0.10229 |
| -0.032000 | 0.10219 |

| E (V) | i (nA) |
|---|---|
| -0.037000 | 0.098880 |
| -0.042000 | 0.093240 |
| -0.047000 | 0.091850 |
| -0.052000 | 0.092630 |
| -0.057000 | 0.088490 |
| -0.062000 | 0.079350 |
| -0.067000 | 0.071440 |
| -0.072000 | 0.065540 |
| -0.077000 | 0.055000 |
| -0.082000 | 0.038950 |
| -0.087000 | 0.024830 |
| -0.092000 | 0.014980 |
| -0.097000 | 0.0036300 |
| -0.10200 | -0.011990 |
| -0.10700 | -0.028050 |
| -0.11200 | -0.042460 |
| -0.11700 | -0.058910 |
| -0.12200 | -0.076790 |
| -0.12700 | -0.089800 |
| -0.13200 | -0.095070 |
| -0.13700 | -0.098270 |
| -0.14200 | -0.10275 |
| -0.14700 | -0.11069 |
| -0.15200 | -0.14309 |
| -0.15700 | -0.19179 |
| -0.16200 | -0.21545 |
| -0.16700 | -0.21642 |
| -0.17200 | -0.21316 |
| -0.17700 | -0.20764 |
| -0.18200 | -0.20717 |
| -0.18700 | -0.21007 |
| -0.19200 | -0.21093 |
| -0.19700 | -0.21270 |
| -0.20200 | -0.21564 |
| -0.20700 | -0.21464 |

| E (V) | i (nA) |
|---|---|
| -0.21200 | -0.20950 |
| -0.21700 | -0.20935 |
| -0.22200 | -0.21430 |
| -0.22700 | -0.21507 |
| -0.23200 | -0.21102 |
| -0.23700 | -0.20746 |
| -0.24200 | -0.20528 |
| -0.24700 | -0.20089 |
| -0.25200 | -0.19494 |
| -0.25700 | -0.19381 |
| -0.26200 | -0.19683 |
| -0.26700 | -0.19448 |
| -0.27200 | -0.18577 |
| -0.27700 | -0.18023 |
| -0.28200 | -0.17834 |
| -0.28700 | -0.17117 |
| -0.29200 | -0.16139 |
| -0.29700 | -0.15284 |
| -0.30200 | -0.14406 |
| -0.30700 | -0.14547 |
| -0.31200 | -0.15622 |
| -0.31700 | -0.16196 |
| -0.32200 | -0.15771 |
| -0.32700 | -0.14385 |
| -0.33200 | -0.13022 |
| -0.33700 | -0.12979 |
| -0.34200 | -0.13413 |
| -0.34700 | -0.13113 |

### 8.3.9 CV-9

| E (V) | i (nA) |
|---|---|
| -0.34800 | -0.13146 |
| -0.34300 | -0.14029 |
| -0.33800 | -0.13499 |
| -0.33300 | -0.14006 |

| E (V) | i (nA) |
|---|---|
| -0.32800 | -0.14238 |
| -0.32300 | -0.14646 |
| -0.31800 | -0.14915 |
| -0.31300 | -0.14675 |
| -0.30800 | -0.14028 |
| -0.30300 | -0.13403 |
| -0.29800 | -0.13172 |
| -0.29300 | -0.12851 |
| -0.28800 | -0.12391 |
| -0.28300 | -0.12076 |
| -0.27800 | -0.12054 |
| -0.27300 | -0.12167 |
| -0.26800 | -0.12332 |
| -0.26300 | -0.13175 |
| -0.25800 | -0.14411 |
| -0.25300 | -0.13355 |
| -0.24800 | -0.089000 |
| -0.24300 | -0.060180 |
| -0.23800 | -0.075850 |
| -0.23300 | -0.091790 |
| -0.22800 | -0.094850 |
| -0.22300 | -0.10469 |
| -0.21800 | -0.11385 |
| -0.21300 | -0.11716 |
| -0.20800 | -0.11799 |
| -0.20300 | -0.12086 |
| -0.19800 | -0.12357 |
| -0.19300 | -0.11507 |
| -0.18800 | -0.098420 |
| -0.18300 | -0.085480 |
| -0.17800 | -0.076930 |
| -0.17300 | -0.066040 |
| -0.16800 | -0.053200 |
| -0.16300 | -0.044180 |
| -0.15800 | -0.036530 |

| E (V) | i (nA) |
|---|---|
| -0.15300 | -0.022840 |
| -0.14800 | -0.0059000 |
| -0.14300 | 0.0074400 |
| -0.13800 | 0.016730 |
| -0.13300 | 0.028670 |
| -0.12800 | 0.047420 |
| -0.12300 | 0.063920 |
| -0.11800 | 0.075340 |
| -0.11300 | 0.091890 |
| -0.10800 | 0.11176 |
| -0.10300 | 0.12295 |
| -0.098000 | 0.12802 |
| -0.093000 | 0.13720 |
| -0.088000 | 0.14886 |
| -0.083000 | 0.15671 |
| -0.078000 | 0.16063 |
| -0.073000 | 0.16567 |
| -0.068000 | 0.17292 |
| -0.063000 | 0.17681 |
| -0.058000 | 0.17731 |
| -0.053000 | 0.18064 |
| -0.048000 | 0.18642 |
| -0.043000 | 0.18838 |
| -0.038000 | 0.18649 |
| -0.033000 | 0.18753 |
| -0.028000 | 0.19198 |
| -0.023000 | 0.19296 |
| -0.018000 | 0.19023 |
| -0.013000 | 0.19054 |
| -0.0080000 | 0.19367 |
| -0.0030000 | 0.19287 |
| 0.0020000 | 0.18893 |
| 0.0070000 | 0.18870 |
| 0.012000 | 0.19191 |
| 0.017000 | 0.19272 |

| E (V) | i (nA) |
|---|---|
| 0.022000 | 0.19067 |
| 0.027000 | 0.18975 |
| 0.032000 | 0.19052 |
| 0.037000 | 0.18891 |
| 0.042000 | 0.18492 |
| 0.047000 | 0.18500 |
| 0.052000 | 0.18845 |
| 0.057000 | 0.18841 |
| 0.062000 | 0.18493 |
| 0.067000 | 0.18433 |
| 0.072000 | 0.18734 |
| 0.077000 | 0.18720 |
| 0.082000 | 0.18382 |
| 0.087000 | 0.18406 |
| 0.092000 | 0.18726 |
| 0.097000 | 0.18673 |
| 0.10200 | 0.18386 |
| 0.10700 | 0.18420 |
| 0.11200 | 0.18703 |
| 0.11700 | 0.18686 |
| 0.12200 | 0.18344 |
| 0.12700 | 0.18333 |
| 0.13200 | 0.18659 |
| 0.13700 | 0.18671 |
| 0.14200 | 0.18330 |
| 0.14700 | 0.18416 |
| 0.15200 | 0.18781 |
| 0.15700 | 0.18594 |
| 0.16200 | 0.18126 |
| 0.16700 | 0.18155 |
| 0.17200 | 0.18565 |
| 0.17700 | 0.18551 |
| 0.18200 | 0.18214 |
| 0.18700 | 0.18194 |
| 0.19200 | 0.18618 |

| E (V) | i (nA) |
|---|---|
| 0.19700 | 0.18351 |
| 0.19800 | 0.17591 |
| 0.19300 | 0.16939 |
| 0.18800 | 0.17010 |
| 0.18300 | 0.16974 |
| 0.17800 | 0.16515 |
| 0.17300 | 0.16448 |
| 0.16800 | 0.16733 |
| 0.16300 | 0.16702 |
| 0.15800 | 0.16280 |
| 0.15300 | 0.16262 |
| 0.14800 | 0.16566 |
| 0.14300 | 0.16480 |
| 0.13800 | 0.16092 |
| 0.13300 | 0.16094 |
| 0.12800 | 0.16415 |
| 0.12300 | 0.16372 |
| 0.11800 | 0.16060 |
| 0.11300 | 0.16027 |
| 0.10800 | 0.16217 |
| 0.10300 | 0.16146 |
| 0.098000 | 0.15790 |
| 0.093000 | 0.15737 |
| 0.088000 | 0.16092 |
| 0.083000 | 0.16107 |
| 0.078000 | 0.15706 |
| 0.073000 | 0.15651 |
| 0.068000 | 0.15967 |
| 0.063000 | 0.15938 |
| 0.058000 | 0.15559 |
| 0.053000 | 0.15522 |
| 0.048000 | 0.15812 |
| 0.043000 | 0.15764 |
| 0.038000 | 0.15411 |
| 0.033000 | 0.15397 |

| E (V) | i (nA) |
|---|---|
| 0.028000 | 0.15651 |
| 0.023000 | 0.15586 |
| 0.018000 | 0.15218 |
| 0.013000 | 0.15130 |
| 0.0080000 | 0.15366 |
| 0.0030000 | 0.15231 |
| -0.0020000 | 0.14728 |
| -0.0070000 | 0.14621 |
| -0.012000 | 0.14845 |
| -0.017000 | 0.14638 |
| -0.022000 | 0.14041 |
| -0.027000 | 0.13785 |
| -0.032000 | 0.13859 |
| -0.037000 | 0.13543 |
| -0.042000 | 0.12844 |
| -0.047000 | 0.12387 |
| -0.052000 | 0.12188 |
| -0.057000 | 0.11620 |
| -0.062000 | 0.10598 |
| -0.067000 | 0.097910 |
| -0.072000 | 0.092220 |
| -0.077000 | 0.082430 |
| -0.082000 | 0.068070 |
| -0.087000 | 0.055650 |
| -0.092000 | 0.046100 |
| -0.097000 | 0.032250 |
| -0.10200 | 0.013890 |
| -0.10700 | -0.0013800 |
| -0.11200 | -0.013600 |
| -0.11700 | -0.029870 |
| -0.12200 | -0.049490 |
| -0.12700 | -0.065570 |
| -0.13200 | -0.078280 |
| -0.13700 | -0.094020 |
| -0.14200 | -0.11252 |

| E (V) | i (nA) |
|---|---|
| -0.14700 | -0.12683 |
| -0.15200 | -0.13698 |
| -0.15700 | -0.14946 |
| -0.16200 | -0.16457 |
| -0.16700 | -0.17527 |
| -0.17200 | -0.18110 |
| -0.17700 | -0.18949 |
| -0.18200 | -0.20051 |
| -0.18700 | -0.20699 |
| -0.19200 | -0.20911 |
| -0.19700 | -0.21388 |
| -0.20200 | -0.22087 |
| -0.20700 | -0.22393 |
| -0.21200 | -0.22389 |
| -0.21700 | -0.22650 |
| -0.22200 | -0.23133 |
| -0.22700 | -0.23272 |
| -0.23200 | -0.23049 |
| -0.23700 | -0.23097 |
| -0.24200 | -0.23452 |
| -0.24700 | -0.23460 |
| -0.25200 | -0.23119 |
| -0.25700 | -0.23098 |
| -0.26200 | -0.23422 |
| -0.26700 | -0.23354 |
| -0.27200 | -0.22950 |
| -0.27700 | -0.22969 |
| -0.28200 | -0.23299 |
| -0.28700 | -0.23208 |
| -0.29200 | -0.22836 |
| -0.29700 | -0.22815 |
| -0.30200 | -0.23075 |
| -0.30700 | -0.23051 |
| -0.31200 | -0.22658 |
| -0.31700 | -0.22606 |

| E (V) | i (nA) |
|---|---|
| -0.32200 | -0.22855 |
| -0.32700 | -0.22857 |
| -0.33200 | -0.22474 |
| -0.33700 | -0.22378 |
| -0.34200 | -0.22776 |
| -0.34700 | -0.22439 |

### 8.3.10 CV-10

| E(V) | i(nA) |
|---|---|
| -0.34800 | -0.22033 |
| -0.34300 | -0.21156 |
| -0.33800 | -0.20658 |
| -0.33300 | -0.20496 |
| -0.32800 | -0.19930 |
| -0.32300 | -0.19782 |
| -0.31800 | -0.19934 |
| -0.31300 | -0.19734 |
| -0.30800 | -0.19293 |
| -0.30300 | -0.19101 |
| -0.29800 | -0.19329 |
| -0.29300 | -0.19232 |
| -0.28800 | -0.18718 |
| -0.28300 | -0.18534 |
| -0.27800 | -0.18755 |
| -0.27300 | -0.18599 |
| -0.26800 | -0.18060 |
| -0.26300 | -0.17906 |
| -0.25800 | -0.18108 |
| -0.25300 | -0.17905 |
| -0.24800 | -0.17320 |
| -0.24300 | -0.17097 |
| -0.23800 | -0.17156 |
| -0.23300 | -0.16853 |
| -0.22800 | -0.16219 |
| -0.22300 | -0.15837 |

| E(V) | i(nA) |
|---|---|
| -0.21800 | -0.15734 |
| -0.21300 | -0.15320 |
| -0.20800 | -0.14494 |
| -0.20300 | -0.13914 |
| -0.19800 | -0.13625 |
| -0.19300 | -0.12884 |
| -0.18800 | -0.11753 |
| -0.18300 | -0.10930 |
| -0.17800 | -0.10324 |
| -0.17300 | -0.092310 |
| -0.16800 | -0.077440 |
| -0.16300 | -0.065870 |
| -0.15800 | -0.056480 |
| -0.15300 | -0.042210 |
| -0.14800 | -0.024470 |
| -0.14300 | -0.0099200 |
| -0.13800 | 0.0021900 |
| -0.13300 | 0.017860 |
| -0.12800 | 0.037020 |
| -0.12300 | 0.052990 |
| -0.11800 | 0.065320 |
| -0.11300 | 0.080580 |
| -0.10800 | 0.098760 |
| -0.10300 | 0.11291 |
| -0.098000 | 0.12280 |
| -0.093000 | 0.13484 |
| -0.088000 | 0.14888 |
| -0.083000 | 0.15836 |
| -0.078000 | 0.16362 |
| -0.073000 | 0.17115 |
| -0.068000 | 0.18017 |
| -0.063000 | 0.18493 |
| -0.058000 | 0.18627 |
| -0.053000 | 0.18954 |
| -0.048000 | 0.19480 |

| E(V) | i(nA) |
|---|---|
| -0.043000 | 0.19693 |
| -0.038000 | 0.19543 |
| -0.033000 | 0.19633 |
| -0.028000 | 0.20059 |
| -0.023000 | 0.20093 |
| -0.018000 | 0.19728 |
| -0.013000 | 0.19766 |
| -0.0080000 | 0.20124 |
| -0.0030000 | 0.20086 |
| 0.0020000 | 0.19734 |
| 0.0070000 | 0.19700 |
| 0.012000 | 0.19992 |
| 0.017000 | 0.19970 |
| 0.022000 | 0.19609 |
| 0.027000 | 0.19573 |
| 0.032000 | 0.19847 |
| 0.037000 | 0.19828 |
| 0.042000 | 0.19488 |
| 0.047000 | 0.19434 |
| 0.052000 | 0.19728 |
| 0.057000 | 0.19706 |
| 0.062000 | 0.19334 |
| 0.067000 | 0.19314 |
| 0.072000 | 0.19635 |
| 0.077000 | 0.19556 |
| 0.082000 | 0.19189 |
| 0.087000 | 0.19221 |
| 0.092000 | 0.19529 |
| 0.097000 | 0.19460 |
| 0.10200 | 0.19122 |
| 0.10700 | 0.19148 |
| 0.11200 | 0.19439 |
| 0.11700 | 0.19388 |
| 0.12200 | 0.19012 |
| 0.12700 | 0.19003 |

| E(V) | i(nA) |
|---|---|
| 0.13200 | 0.19341 |
| 0.13700 | 0.19337 |
| 0.14200 | 0.18962 |
| 0.14700 | 0.18939 |
| 0.15200 | 0.19304 |
| 0.15700 | 0.19283 |
| 0.16200 | 0.18920 |
| 0.16700 | 0.18933 |
| 0.17200 | 0.19267 |
| 0.17700 | 0.19221 |
| 0.18200 | 0.18950 |
| 0.18700 | 0.18944 |
| 0.19200 | 0.19275 |
| 0.19700 | 0.19085 |
| 0.19800 | 0.17934 |
| 0.19300 | 0.17866 |
| 0.18800 | 0.17576 |
| 0.18300 | 0.17587 |
| 0.17800 | 0.17123 |
| 0.17300 | 0.17076 |
| 0.16800 | 0.17385 |
| 0.16300 | 0.17303 |
| 0.15800 | 0.16910 |
| 0.15300 | 0.16900 |
| 0.14800 | 0.17202 |
| 0.14300 | 0.17123 |
| 0.13800 | 0.16730 |
| 0.13300 | 0.16689 |
| 0.12800 | 0.16987 |
| 0.12300 | 0.16912 |
| 0.11800 | 0.16552 |
| 0.11300 | 0.16520 |
| 0.10800 | 0.16798 |
| 0.10300 | 0.16792 |
| 0.098000 | 0.16431 |

| E(V) | i(nA) |
|---|---|
| 0.093000 | 0.16369 |
| 0.088000 | 0.16666 |
| 0.083000 | 0.16649 |
| 0.078000 | 0.16272 |
| 0.073000 | 0.16238 |
| 0.068000 | 0.16527 |
| 0.063000 | 0.16444 |
| 0.058000 | 0.16055 |
| 0.053000 | 0.16038 |
| 0.048000 | 0.16296 |
| 0.043000 | 0.16204 |
| 0.038000 | 0.15848 |
| 0.033000 | 0.15814 |
| 0.028000 | 0.16084 |
| 0.023000 | 0.16016 |
| 0.018000 | 0.15598 |
| 0.013000 | 0.15518 |
| 0.0080000 | 0.15806 |
| 0.0030000 | 0.15693 |
| -0.0020000 | 0.15175 |
| -0.0070000 | 0.15036 |
| -0.012000 | 0.15202 |
| -0.017000 | 0.14982 |
| -0.022000 | 0.14407 |
| -0.027000 | 0.14128 |
| -0.032000 | 0.14152 |
| -0.037000 | 0.13794 |
| -0.042000 | 0.13066 |
| -0.047000 | 0.12589 |
| -0.052000 | 0.12332 |
| -0.057000 | 0.11696 |
| -0.062000 | 0.10637 |
| -0.067000 | 0.097740 |
| -0.072000 | 0.091240 |
| -0.077000 | 0.080230 |

| E(V) | i(nA) |
|---|---|
| -0.082000 | 0.064960 |
| -0.087000 | 0.051880 |
| -0.092000 | 0.040860 |
| -0.097000 | 0.025600 |
| -0.10200 | 0.0061500 |
| -0.10700 | -0.010090 |
| -0.11200 | -0.023380 |
| -0.11700 | -0.041160 |
| -0.12200 | -0.061690 |
| -0.12700 | -0.078320 |
| -0.13200 | -0.092060 |
| -0.13700 | -0.10827 |
| -0.14200 | -0.12722 |
| -0.14700 | -0.14243 |
| -0.15200 | -0.15309 |
| -0.15700 | -0.16599 |
| -0.16200 | -0.18132 |
| -0.16700 | -0.19245 |
| -0.17200 | -0.19887 |
| -0.17700 | -0.20726 |
| -0.18200 | -0.21809 |
| -0.18700 | -0.22458 |
| -0.19200 | -0.22691 |
| -0.19700 | -0.23155 |
| -0.20200 | -0.23831 |
| -0.20700 | -0.24114 |
| -0.21200 | -0.24059 |
| -0.21700 | -0.24289 |
| -0.22200 | -0.24734 |
| -0.22700 | -0.24820 |
| -0.23200 | -0.24597 |
| -0.23700 | -0.24658 |
| -0.24200 | -0.25032 |
| -0.24700 | -0.25032 |
| -0.25200 | -0.24652 |

| E(V) | i(nA) |
|---|---|
| -0.25700 | -0.24635 |
| -0.26200 | -0.24944 |
| -0.26700 | -0.24876 |
| -0.27200 | -0.24458 |
| -0.27700 | -0.24396 |
| -0.28200 | -0.24694 |
| -0.28700 | -0.24621 |
| -0.29200 | -0.24241 |
| -0.29700 | -0.24216 |
| -0.30200 | -0.24473 |
| -0.30700 | -0.24460 |
| -0.31200 | -0.24103 |
| -0.31700 | -0.24050 |
| -0.32200 | -0.24328 |
| -0.32700 | -0.24278 |
| -0.33200 | -0.23934 |
| -0.33700 | -0.23875 |
| -0.34200 | -0.24230 |
| -0.34700 | -0.23922 |

### 8.3.11 CV-11

| E (V) | i (nA) |
|---|---|
| -0.34800 | -0.23168 |
| -0.34300 | -0.22349 |
| -0.33800 | -0.22128 |
| -0.33300 | -0.21899 |
| -0.32800 | -0.21350 |
| -0.32300 | -0.21220 |
| -0.31800 | -0.21388 |
| -0.31300 | -0.21194 |
| -0.30800 | -0.20728 |
| -0.30300 | -0.20569 |
| -0.29800 | -0.20741 |
| -0.29300 | -0.20621 |
| -0.28800 | -0.20127 |

| E (V) | i (nA) |
|---|---|
| -0.28300 | -0.19934 |
| -0.27800 | -0.20141 |
| -0.27300 | -0.19991 |
| -0.26800 | -0.19462 |
| -0.26300 | -0.19293 |
| -0.25800 | -0.19449 |
| -0.25300 | -0.19240 |
| -0.24800 | -0.18696 |
| -0.24300 | -0.18460 |
| -0.23800 | -0.18505 |
| -0.23300 | -0.18201 |
| -0.22800 | -0.17573 |
| -0.22300 | -0.17223 |
| -0.21800 | -0.17113 |
| -0.21300 | -0.16658 |
| -0.20800 | -0.15816 |
| -0.20300 | -0.15216 |
| -0.19800 | -0.14894 |
| -0.19300 | -0.14161 |
| -0.18800 | -0.13016 |
| -0.18300 | -0.12159 |
| -0.17800 | -0.11553 |
| -0.17300 | -0.10453 |
| -0.16800 | -0.089320 |
| -0.16300 | -0.077080 |
| -0.15800 | -0.067170 |
| -0.15300 | -0.053120 |
| -0.14800 | -0.034920 |
| -0.14300 | -0.019090 |
| -0.13800 | -0.0060600 |
| -0.13300 | 0.010280 |
| -0.12800 | 0.030010 |
| -0.12300 | 0.047010 |
| -0.11800 | 0.060440 |
| -0.11300 | 0.076520 |

| E (V) | i (nA) |
|---|---|
| -0.10800 | 0.095690 |
| -0.10300 | 0.11067 |
| -0.098000 | 0.12138 |
| -0.093000 | 0.13461 |
| -0.088000 | 0.14958 |
| -0.083000 | 0.15968 |
| -0.078000 | 0.16598 |
| -0.073000 | 0.17445 |
| -0.068000 | 0.18415 |
| -0.063000 | 0.18959 |
| -0.058000 | 0.19094 |
| -0.053000 | 0.19457 |
| -0.048000 | 0.20084 |
| -0.043000 | 0.20300 |
| -0.038000 | 0.20129 |
| -0.033000 | 0.20246 |
| -0.028000 | 0.20671 |
| -0.023000 | 0.20737 |
| -0.018000 | 0.20404 |
| -0.013000 | 0.20417 |
| -0.0080000 | 0.20792 |
| -0.0030000 | 0.20780 |
| 0.0020000 | 0.20431 |
| 0.0070000 | 0.20408 |
| 0.012000 | 0.20647 |
| 0.017000 | 0.20597 |
| 0.022000 | 0.20261 |
| 0.027000 | 0.20188 |
| 0.032000 | 0.20457 |
| 0.037000 | 0.20434 |
| 0.042000 | 0.20070 |
| 0.047000 | 0.20042 |
| 0.052000 | 0.20332 |
| 0.057000 | 0.20289 |
| 0.062000 | 0.19927 |

| E (V) | i (nA) |
|---|---|
| 0.067000 | 0.19920 |
| 0.072000 | 0.20226 |
| 0.077000 | 0.20144 |
| 0.082000 | 0.19761 |
| 0.087000 | 0.19759 |
| 0.092000 | 0.20038 |
| 0.097000 | 0.19969 |
| 0.10200 | 0.19663 |
| 0.10700 | 0.19682 |
| 0.11200 | 0.19946 |
| 0.11700 | 0.19907 |
| 0.12200 | 0.19567 |
| 0.12700 | 0.19540 |
| 0.13200 | 0.19857 |
| 0.13700 | 0.19869 |
| 0.14200 | 0.19541 |
| 0.14700 | 0.19501 |
| 0.15200 | 0.19793 |
| 0.15700 | 0.19791 |
| 0.16200 | 0.19447 |
| 0.16700 | 0.19460 |
| 0.17200 | 0.19751 |
| 0.17700 | 0.19706 |
| 0.18200 | 0.19433 |
| 0.18700 | 0.19440 |
| 0.19200 | 0.19791 |
| 0.19700 | 0.19574 |
| 0.19800 | 0.18711 |
| 0.19300 | 0.18322 |
| 0.18800 | 0.18161 |
| 0.18300 | 0.18165 |
| 0.17800 | 0.17688 |
| 0.17300 | 0.17619 |
| 0.16800 | 0.17887 |
| 0.16300 | 0.17807 |

| E (V) | i (nA) |
|---|---|
| 0.15800 | 0.17390 |
| 0.15300 | 0.17353 |
| 0.14800 | 0.17644 |
| 0.14300 | 0.17574 |
| 0.13800 | 0.17223 |
| 0.13300 | 0.17187 |
| 0.12800 | 0.17455 |
| 0.12300 | 0.17402 |
| 0.11800 | 0.17077 |
| 0.11300 | 0.17043 |
| 0.10800 | 0.17296 |
| 0.10300 | 0.17256 |
| 0.098000 | 0.16873 |
| 0.093000 | 0.16810 |
| 0.088000 | 0.17089 |
| 0.083000 | 0.17038 |
| 0.078000 | 0.16668 |
| 0.073000 | 0.16652 |
| 0.068000 | 0.16942 |
| 0.063000 | 0.16869 |
| 0.058000 | 0.16493 |
| 0.053000 | 0.16487 |
| 0.048000 | 0.16765 |
| 0.043000 | 0.16695 |
| 0.038000 | 0.16357 |
| 0.033000 | 0.16294 |
| 0.028000 | 0.16516 |
| 0.023000 | 0.16452 |
| 0.018000 | 0.16025 |
| 0.013000 | 0.15920 |
| 0.0080000 | 0.16174 |
| 0.0030000 | 0.16040 |
| -0.0020000 | 0.15550 |
| -0.0070000 | 0.15430 |
| -0.012000 | 0.15585 |

| E (V) | i (nA) |
|---|---|
| -0.017000 | 0.15344 |
| -0.022000 | 0.14786 |
| -0.027000 | 0.14510 |
| -0.032000 | 0.14456 |
| -0.037000 | 0.14040 |
| -0.042000 | 0.13294 |
| -0.047000 | 0.12771 |
| -0.052000 | 0.12421 |
| -0.057000 | 0.11670 |
| -0.062000 | 0.10555 |
| -0.067000 | 0.096180 |
| -0.072000 | 0.088650 |
| -0.077000 | 0.077000 |
| -0.082000 | 0.060530 |
| -0.087000 | 0.046660 |
| -0.092000 | 0.035200 |
| -0.097000 | 0.018570 |
| -0.10200 | -0.0018100 |
| -0.10700 | -0.019130 |
| -0.11200 | -0.034120 |
| -0.11700 | -0.052760 |
| -0.12200 | -0.074250 |
| -0.12700 | -0.092290 |
| -0.13200 | -0.10693 |
| -0.13700 | -0.12387 |
| -0.14200 | -0.14329 |
| -0.14700 | -0.15894 |
| -0.15200 | -0.17031 |
| -0.15700 | -0.18347 |
| -0.16200 | -0.19866 |
| -0.16700 | -0.20962 |
| -0.17200 | -0.21603 |
| -0.17700 | -0.22482 |
| -0.18200 | -0.23575 |
| -0.18700 | -0.24171 |

| E (V) | i (nA) |
|---|---|
| -0.19200 | -0.24405 |
| -0.19700 | -0.24903 |
| -0.20200 | -0.25582 |
| -0.20700 | -0.25869 |
| -0.21200 | -0.25773 |
| -0.21700 | -0.25951 |
| -0.22200 | -0.26398 |
| -0.22700 | -0.26474 |
| -0.23200 | -0.26208 |
| -0.23700 | -0.26231 |
| -0.24200 | -0.26571 |
| -0.24700 | -0.26586 |
| -0.25200 | -0.26219 |
| -0.25700 | -0.26169 |
| -0.26200 | -0.26481 |
| -0.26700 | -0.26407 |
| -0.27200 | -0.26024 |
| -0.27700 | -0.25964 |
| -0.28200 | -0.26198 |
| -0.28700 | -0.26170 |
| -0.29200 | -0.25810 |
| -0.29700 | -0.25754 |
| -0.30200 | -0.25972 |
| -0.30700 | -0.25902 |
| -0.31200 | -0.25575 |
| -0.31700 | -0.25549 |
| -0.32200 | -0.25797 |
| -0.32700 | -0.25761 |
| -0.33200 | -0.25406 |
| -0.33700 | -0.25356 |
| -0.34200 | -0.25744 |
| -0.34700 | -0.25419 |

### 8.3.12 CV-12

| E (V) | i (nA) |
|---|---|

| E (V) | i (nA) |
|---|---|
| -0.34800 | -0.24741 |
| -0.34300 | -0.23719 |
| -0.33800 | -0.23636 |
| -0.33300 | -0.23390 |
| -0.32800 | -0.22853 |
| -0.32300 | -0.22681 |
| -0.31800 | -0.22798 |
| -0.31300 | -0.22652 |
| -0.30800 | -0.22178 |
| -0.30300 | -0.21995 |
| -0.29800 | -0.22147 |
| -0.29300 | -0.22031 |
| -0.28800 | -0.21547 |
| -0.28300 | -0.21315 |
| -0.27800 | -0.21489 |
| -0.27300 | -0.21363 |
| -0.26800 | -0.20864 |
| -0.26300 | -0.20672 |
| -0.25800 | -0.20799 |
| -0.25300 | -0.20581 |
| -0.24800 | -0.20056 |
| -0.24300 | -0.19842 |
| -0.23800 | -0.19852 |
| -0.23300 | -0.19529 |
| -0.22800 | -0.18923 |
| -0.22300 | -0.18561 |
| -0.21800 | -0.18443 |
| -0.21300 | -0.17996 |
| -0.20800 | -0.17156 |
| -0.20300 | -0.16548 |
| -0.19800 | -0.16213 |
| -0.19300 | -0.15481 |
| -0.18800 | -0.14334 |
| -0.18300 | -0.13426 |
| -0.17800 | -0.12769 |

| E (V) | i (nA) |
|---|---|
| -0.17300 | -0.11674 |
| -0.16800 | -0.10151 |
| -0.16300 | -0.088820 |
| -0.15800 | -0.077960 |
| -0.15300 | -0.063110 |
| -0.14800 | -0.044880 |
| -0.14300 | -0.028460 |
| -0.13800 | -0.014330 |
| -0.13300 | 0.0029200 |
| -0.12800 | 0.023640 |
| -0.12300 | 0.041610 |
| -0.11800 | 0.056220 |
| -0.11300 | 0.073400 |
| -0.10800 | 0.093520 |
| -0.10300 | 0.10963 |
| -0.098000 | 0.12145 |
| -0.093000 | 0.13588 |
| -0.088000 | 0.15177 |
| -0.083000 | 0.16261 |
| -0.078000 | 0.16959 |
| -0.073000 | 0.17874 |
| -0.068000 | 0.18933 |
| -0.063000 | 0.19518 |
| -0.058000 | 0.19707 |
| -0.053000 | 0.20121 |
| -0.048000 | 0.20762 |
| -0.043000 | 0.21049 |
| -0.038000 | 0.20909 |
| -0.033000 | 0.20997 |
| -0.028000 | 0.21428 |
| -0.023000 | 0.21503 |
| -0.018000 | 0.21192 |
| -0.013000 | 0.21219 |
| -0.0080000 | 0.21507 |
| -0.0030000 | 0.21479 |

| E (V) | i (nA) |
|---|---|
| 0.0020000 | 0.21189 |
| 0.0070000 | 0.21116 |
| 0.012000 | 0.21357 |
| 0.017000 | 0.21338 |
| 0.022000 | 0.20995 |
| 0.027000 | 0.20953 |
| 0.032000 | 0.21185 |
| 0.037000 | 0.21127 |
| 0.042000 | 0.20785 |
| 0.047000 | 0.20746 |
| 0.052000 | 0.21016 |
| 0.057000 | 0.20962 |
| 0.062000 | 0.20603 |
| 0.067000 | 0.20589 |
| 0.072000 | 0.20864 |
| 0.077000 | 0.20807 |
| 0.082000 | 0.20473 |
| 0.087000 | 0.20458 |
| 0.092000 | 0.20725 |
| 0.097000 | 0.20682 |
| 0.10200 | 0.20376 |
| 0.10700 | 0.20347 |
| 0.11200 | 0.20590 |
| 0.11700 | 0.20584 |
| 0.12200 | 0.20267 |
| 0.12700 | 0.20199 |
| 0.13200 | 0.20503 |
| 0.13700 | 0.20551 |
| 0.14200 | 0.20224 |
| 0.14700 | 0.20199 |
| 0.15200 | 0.20503 |
| 0.15700 | 0.20482 |
| 0.16200 | 0.20152 |
| 0.16700 | 0.20176 |
| 0.17200 | 0.20462 |

| E (V) | i (nA) |
|---|---|
| 0.17700 | 0.20442 |
| 0.18200 | 0.20127 |
| 0.18700 | 0.20114 |
| 0.19200 | 0.20463 |
| 0.19700 | 0.20189 |
| 0.19800 | 0.19463 |
| 0.19300 | 0.19301 |
| 0.18800 | 0.18701 |
| 0.18300 | 0.18780 |
| 0.17800 | 0.18324 |
| 0.17300 | 0.18232 |
| 0.16800 | 0.18494 |
| 0.16300 | 0.18420 |
| 0.15800 | 0.18040 |
| 0.15300 | 0.17973 |
| 0.14800 | 0.18246 |
| 0.14300 | 0.18132 |
| 0.13800 | 0.17824 |
| 0.13300 | 0.17778 |
| 0.12800 | 0.17980 |
| 0.12300 | 0.17957 |
| 0.11800 | 0.17668 |
| 0.11300 | 0.17630 |
| 0.10800 | 0.17880 |
| 0.10300 | 0.17863 |
| 0.098000 | 0.17511 |
| 0.093000 | 0.17446 |
| 0.088000 | 0.17690 |
| 0.083000 | 0.17613 |
| 0.078000 | 0.17233 |
| 0.073000 | 0.17226 |
| 0.068000 | 0.17493 |
| 0.063000 | 0.17431 |
| 0.058000 | 0.17092 |
| 0.053000 | 0.17058 |

| E (V) | i (nA) |
|---|---|
| 0.048000 | 0.17319 |
| 0.043000 | 0.17246 |
| 0.038000 | 0.16920 |
| 0.033000 | 0.16866 |
| 0.028000 | 0.17070 |
| 0.023000 | 0.17025 |
| 0.018000 | 0.16652 |
| 0.013000 | 0.16506 |
| 0.0080000 | 0.16677 |
| 0.0030000 | 0.16577 |
| -0.0020000 | 0.16103 |
| -0.0070000 | 0.15910 |
| -0.012000 | 0.16037 |
| -0.017000 | 0.15773 |
| -0.022000 | 0.15204 |
| -0.027000 | 0.14944 |
| -0.032000 | 0.14837 |
| -0.037000 | 0.14408 |
| -0.042000 | 0.13698 |
| -0.047000 | 0.13103 |
| -0.052000 | 0.12605 |
| -0.057000 | 0.11728 |
| -0.062000 | 0.10509 |
| -0.067000 | 0.094960 |
| -0.072000 | 0.086720 |
| -0.077000 | 0.074160 |
| -0.082000 | 0.057020 |
| -0.087000 | 0.041520 |
| -0.092000 | 0.028150 |
| -0.097000 | 0.011070 |
| -0.10200 | -0.0099900 |
| -0.10700 | -0.028800 |
| -0.11200 | -0.045040 |
| -0.11700 | -0.064380 |
| -0.12200 | -0.086400 |

| E (V) | i (nA) |
|---|---|
| -0.12700 | -0.10549 |
| -0.13200 | -0.12151 |
| -0.13700 | -0.13916 |
| -0.14200 | -0.15874 |
| -0.14700 | -0.17492 |
| -0.15200 | -0.18679 |
| -0.15700 | -0.20001 |
| -0.16200 | -0.21503 |
| -0.16700 | -0.22613 |
| -0.17200 | -0.23331 |
| -0.17700 | -0.24229 |
| -0.18200 | -0.25267 |
| -0.18700 | -0.25825 |
| -0.19200 | -0.26014 |
| -0.19700 | -0.26418 |
| -0.20200 | -0.26903 |
| -0.20700 | -0.27241 |
| -0.21200 | -0.27526 |
| -0.21700 | -0.27800 |
| -0.22200 | -0.28148 |
| -0.22700 | -0.28280 |
| -0.23200 | -0.28036 |
| -0.23700 | -0.27976 |
| -0.24200 | -0.28240 |
| -0.24700 | -0.28215 |
| -0.25200 | -0.27864 |
| -0.25700 | -0.27843 |
| -0.26200 | -0.28107 |
| -0.26700 | -0.28013 |
| -0.27200 | -0.27667 |
| -0.27700 | -0.27663 |
| -0.28200 | -0.27843 |
| -0.28700 | -0.27726 |
| -0.29200 | -0.27404 |
| -0.29700 | -0.27352 |

| E (V) | i (nA) |
|---|---|
| -0.30200 | -0.27511 |
| -0.30700 | -0.27433 |
| -0.31200 | -0.27128 |
| -0.31700 | -0.27057 |
| -0.32200 | -0.27310 |
| -0.32700 | -0.27235 |
| -0.33200 | -0.26888 |
| -0.33700 | -0.26775 |
| -0.34200 | -0.27092 |
| -0.34700 | -0.26868 |

### 8.3.13 CV-13

| Column 1 | Column 2 |
|---|---|
| -0.34800 | -0.25828 |
| -0.34300 | -0.24373 |
| -0.33800 | -0.24765 |
| -0.33300 | -0.24342 |
| -0.32800 | -0.24346 |
| -0.32300 | -0.24812 |
| -0.31800 | -0.24854 |
| -0.31300 | -0.24401 |
| -0.30800 | -0.23782 |
| -0.30300 | -0.23630 |
| -0.29800 | -0.23783 |
| -0.29300 | -0.23713 |
| -0.28800 | -0.23252 |
| -0.28300 | -0.23073 |
| -0.27800 | -0.23175 |
| -0.27300 | -0.22957 |
| -0.26800 | -0.22479 |
| -0.26300 | -0.22306 |
| -0.25800 | -0.22401 |
| -0.25300 | -0.22104 |
| -0.24800 | -0.21557 |
| -0.24300 | -0.21316 |

| Column 1 | Column 2 |
|---|---|
| -0.23800 | -0.21293 |
| -0.23300 | -0.20971 |
| -0.22800 | -0.20415 |
| -0.22300 | -0.20028 |
| -0.21800 | -0.19843 |
| -0.21300 | -0.19509 |
| -0.20800 | -0.18789 |
| -0.20300 | -0.18194 |
| -0.19800 | -0.17907 |
| -0.19300 | -0.17341 |
| -0.18800 | -0.16329 |
| -0.18300 | -0.15181 |
| -0.17800 | -0.13602 |
| -0.17300 | -0.11726 |
| -0.16800 | -0.11271 |
| -0.16300 | -0.11687 |
| -0.15800 | -0.10560 |
| -0.15300 | -0.084640 |
| -0.14800 | -0.065600 |
| -0.14300 | -0.047590 |
| -0.13800 | -0.030930 |
| -0.13300 | -0.012010 |
| -0.12800 | 0.0095100 |
| -0.12300 | 0.028150 |
| -0.11800 | 0.043970 |
| -0.11300 | 0.062440 |
| -0.10800 | 0.083140 |
| -0.10300 | 0.099950 |
| -0.098000 | 0.11292 |
| -0.093000 | 0.12745 |
| -0.088000 | 0.14301 |
| -0.083000 | 0.15425 |
| -0.078000 | 0.16099 |
| -0.073000 | 0.16668 |
| -0.068000 | 0.17245 |

| Column 1 | Column 2 |
|---|---|
| -0.063000 | 0.17577 |
| -0.058000 | 0.17736 |
| -0.053000 | 0.18246 |
| -0.048000 | 0.19161 |
| -0.043000 | 0.20306 |
| -0.038000 | 0.21255 |
| -0.033000 | 0.21355 |
| -0.028000 | 0.21085 |
| -0.023000 | 0.20853 |
| -0.018000 | 0.20018 |
| -0.013000 | 0.19061 |
| -0.0080000 | 0.18878 |
| -0.0030000 | 0.19130 |
| 0.0020000 | 0.19390 |
| 0.0070000 | 0.19746 |
| 0.012000 | 0.20114 |
| 0.017000 | 0.20171 |
| 0.022000 | 0.19969 |
| 0.027000 | 0.19855 |
| 0.032000 | 0.19891 |
| 0.037000 | 0.19641 |
| 0.042000 | 0.19144 |
| 0.047000 | 0.19183 |
| 0.052000 | 0.19824 |
| 0.057000 | 0.20194 |
| 0.062000 | 0.20077 |
| 0.067000 | 0.20220 |
| 0.072000 | 0.20671 |
| 0.077000 | 0.20794 |
| 0.082000 | 0.20653 |
| 0.087000 | 0.20734 |
| 0.092000 | 0.21007 |
| 0.097000 | 0.21139 |
| 0.10200 | 0.20854 |
| 0.10700 | 0.20587 |

| Column 1 | Column 2 |
|---|---|
| 0.11200 | 0.20821 |
| 0.11700 | 0.20906 |
| 0.12200 | 0.20655 |
| 0.12700 | 0.20626 |
| 0.13200 | 0.20961 |
| 0.13700 | 0.21057 |
| 0.14200 | 0.20883 |
| 0.14700 | 0.21110 |
| 0.15200 | 0.21603 |
| 0.15700 | 0.21630 |
| 0.16200 | 0.21291 |
| 0.16700 | 0.21548 |
| 0.17200 | 0.21959 |
| 0.17700 | 0.21998 |
| 0.18200 | 0.21640 |
| 0.18700 | 0.21623 |
| 0.19200 | 0.21858 |
| 0.19700 | 0.21338 |
| 0.19800 | 0.21490 |
| 0.19300 | 0.20763 |
| 0.18800 | 0.19890 |
| 0.18300 | 0.19882 |
| 0.17800 | 0.19340 |
| 0.17300 | 0.19216 |
| 0.16800 | 0.20029 |
| 0.16300 | 0.21088 |
| 0.15800 | 0.22275 |
| 0.15300 | 0.22644 |
| 0.14800 | 0.23017 |
| 0.14300 | 0.23431 |
| 0.13800 | 0.24216 |
| 0.13300 | 0.26288 |
| 0.12800 | 0.27425 |
| 0.12300 | 0.25539 |
| 0.11800 | 0.22267 |

| Column 1 | Column 2 |
|---|---|
| 0.11300 | 0.20305 |
| 0.10800 | 0.19035 |
| 0.10300 | 0.17472 |
| 0.098000 | 0.16841 |
| 0.093000 | 0.17360 |
| 0.088000 | 0.17998 |
| 0.083000 | 0.18420 |
| 0.078000 | 0.18695 |
| 0.073000 | 0.19176 |
| 0.068000 | 0.19773 |
| 0.063000 | 0.19779 |
| 0.058000 | 0.19258 |
| 0.053000 | 0.18993 |
| 0.048000 | 0.19098 |
| 0.043000 | 0.19354 |
| 0.038000 | 0.19750 |
| 0.033000 | 0.20202 |
| 0.028000 | 0.21060 |
| 0.023000 | 0.21634 |
| 0.018000 | 0.21933 |
| 0.013000 | 0.22248 |
| 0.0080000 | 0.21539 |
| 0.0030000 | 0.21302 |
| -0.0020000 | 0.21799 |
| -0.0070000 | 0.21199 |
| -0.012000 | 0.20547 |
| -0.017000 | 0.19515 |
| -0.022000 | 0.17978 |
| -0.027000 | 0.13979 |
| -0.032000 | 0.053070 |
| -0.037000 | 0.049570 |
| -0.042000 | 0.13602 |
| -0.047000 | 0.14966 |
| -0.052000 | 0.13279 |
| -0.057000 | 0.13779 |

| Column 1 | Column 2 |
|---|---|
| -0.062000 | 0.12768 |
| -0.067000 | 0.12122 |
| -0.072000 | 0.11885 |
| -0.077000 | 0.11063 |
| -0.082000 | 0.096840 |
| -0.087000 | 0.081060 |
| -0.092000 | 0.067990 |
| -0.097000 | 0.051180 |
| -0.10200 | 0.025980 |
| -0.10700 | 0.0024100 |
| -0.11200 | -0.013910 |
| -0.11700 | -0.029160 |
| -0.12200 | -0.043980 |
| -0.12700 | -0.061710 |
| -0.13200 | -0.078610 |
| -0.13700 | -0.083860 |
| -0.14200 | -0.098170 |
| -0.14700 | -0.13277 |
| -0.15200 | -0.15996 |
| -0.15700 | -0.17874 |
| -0.16200 | -0.19481 |
| -0.16700 | -0.20420 |
| -0.17200 | -0.21233 |
| -0.17700 | -0.22042 |
| -0.18200 | -0.22578 |
| -0.18700 | -0.22449 |
| -0.19200 | -0.22146 |
| -0.19700 | -0.22425 |
| -0.20200 | -0.23265 |
| -0.20700 | -0.23811 |
| -0.21200 | -0.24154 |
| -0.21700 | -0.24655 |
| -0.22200 | -0.25349 |
| -0.22700 | -0.25807 |
| -0.23200 | -0.25519 |

| Column 1 | Column 2 |
|---|---|
| -0.23700 | -0.24938 |
| -0.24200 | -0.24544 |
| -0.24700 | -0.24380 |
| -0.25200 | -0.24044 |
| -0.25700 | -0.24138 |
| -0.26200 | -0.24170 |
| -0.26700 | -0.24339 |
| -0.27200 | -0.24218 |
| -0.27700 | -0.24492 |
| -0.28200 | -0.25038 |
| -0.28700 | -0.25662 |
| -0.29200 | -0.27651 |
| -0.29700 | -0.27992 |
| -0.30200 | -0.38197 |
| -0.30700 | -0.47342 |
| -0.31200 | -0.34292 |
| -0.31700 | -0.26855 |
| -0.32200 | -0.30780 |
| -0.32700 | -0.29254 |
| -0.33200 | -0.29427 |
| -0.33700 | -0.31722 |
| -0.34200 | -0.34199 |
| -0.34700 | -0.36099 |

### 8.3.14 CV-14

| Column 1 | Column 2 |
|---|---|
| -0.34800 | -0.34692 |
| -0.34300 | -0.28902 |
| -0.33800 | -0.30494 |
| -0.33300 | -0.29176 |
| -0.32800 | -0.27576 |
| -0.32300 | -0.26838 |
| -0.31800 | -0.26805 |
| -0.31300 | -0.26812 |
| -0.30800 | -0.26089 |

| Column 1 | Column 2 |
|---|---|
| -0.30300 | -0.25315 |
| -0.29800 | -0.25005 |
| -0.29300 | -0.25422 |
| -0.28800 | -0.25636 |
| -0.28300 | -0.25986 |
| -0.27800 | -0.26593 |
| -0.27300 | -0.26997 |
| -0.26800 | -0.27293 |
| -0.26300 | -0.27713 |
| -0.25800 | -0.28218 |
| -0.25300 | -0.28382 |
| -0.24800 | -0.28085 |
| -0.24300 | -0.27255 |
| -0.23800 | -0.26733 |
| -0.23300 | -0.26760 |
| -0.22800 | -0.26155 |
| -0.22300 | -0.25356 |
| -0.21800 | -0.25560 |
| -0.21300 | -0.25598 |
| -0.20800 | -0.24611 |
| -0.20300 | -0.23746 |
| -0.19800 | -0.23271 |
| -0.19300 | -0.22531 |
| -0.18800 | -0.21521 |
| -0.18300 | -0.20601 |
| -0.17800 | -0.19762 |
| -0.17300 | -0.18868 |
| -0.16800 | -0.17762 |
| -0.16300 | -0.16309 |
| -0.15800 | -0.15158 |
| -0.15300 | -0.14600 |
| -0.14800 | -0.13926 |
| -0.14300 | -0.13612 |
| -0.13800 | -0.13470 |
| -0.13300 | -0.12101 |

| Column 1 | Column 2 |
|---|---|
| -0.12800 | -0.096400 |
| -0.12300 | -0.067580 |
| -0.11800 | -0.045400 |
| -0.11300 | -0.030980 |
| -0.10800 | -0.0090900 |
| -0.10300 | 0.019970 |
| -0.098000 | 0.042790 |
| -0.093000 | 0.061080 |
| -0.088000 | 0.083700 |
| -0.083000 | 0.10580 |
| -0.078000 | 0.12308 |
| -0.073000 | 0.13586 |
| -0.068000 | 0.14949 |
| -0.063000 | 0.16755 |
| -0.058000 | 0.18106 |
| -0.053000 | 0.19032 |
| -0.048000 | 0.20062 |
| -0.043000 | 0.21200 |
| -0.038000 | 0.22000 |
| -0.033000 | 0.22015 |
| -0.028000 | 0.21976 |
| -0.023000 | 0.21798 |
| -0.018000 | 0.21229 |
| -0.013000 | 0.21304 |
| -0.0080000 | 0.21732 |
| -0.0030000 | 0.21863 |
| 0.0020000 | 0.22077 |
| 0.0070000 | 0.22567 |
| 0.012000 | 0.23194 |
| 0.017000 | 0.23643 |
| 0.022000 | 0.23837 |
| 0.027000 | 0.23898 |
| 0.032000 | 0.24033 |
| 0.037000 | 0.24069 |
| 0.042000 | 0.23840 |

| Column 1 | Column 2 |
|---|---|
| 0.047000 | 0.23722 |
| 0.052000 | 0.23853 |
| 0.057000 | 0.23884 |
| 0.062000 | 0.23723 |
| 0.067000 | 0.23859 |
| 0.072000 | 0.24115 |
| 0.077000 | 0.24078 |
| 0.082000 | 0.23946 |
| 0.087000 | 0.24145 |
| 0.092000 | 0.24607 |
| 0.097000 | 0.24978 |
| 0.10200 | 0.25380 |
| 0.10700 | 0.26088 |
| 0.11200 | 0.27046 |
| 0.11700 | 0.27853 |
| 0.12200 | 0.28482 |
| 0.12700 | 0.29195 |
| 0.13200 | 0.30284 |
| 0.13700 | 0.31185 |
| 0.14200 | 0.31737 |
| 0.14700 | 0.32148 |
| 0.15200 | 0.31959 |
| 0.15700 | 0.31152 |
| 0.16200 | 0.30087 |
| 0.16700 | 0.29249 |
| 0.17200 | 0.28220 |
| 0.17700 | 0.27531 |
| 0.18200 | 0.26959 |
| 0.18700 | 0.27064 |
| 0.19200 | 0.27259 |
| 0.19700 | 0.26206 |
| 0.19800 | 0.26890 |
| 0.19300 | 0.26402 |
| 0.18800 | 0.25062 |
| 0.18300 | 0.25949 |

| Column 1 | Column 2 |
|---|---|
| 0.17800 | 0.25909 |
| 0.17300 | 0.25839 |
| 0.16800 | 0.25629 |
| 0.16300 | 0.24763 |
| 0.15800 | 0.24510 |
| 0.15300 | 0.24518 |
| 0.14800 | 0.24163 |
| 0.14300 | 0.24198 |
| 0.13800 | 0.25038 |
| 0.13300 | 0.25091 |
| 0.12800 | 0.25165 |
| 0.12300 | 0.25387 |
| 0.11800 | 0.24247 |
| 0.11300 | 0.23256 |
| 0.10800 | 0.24105 |
| 0.10300 | 0.25002 |
| 0.098000 | 0.24109 |
| 0.093000 | 0.24251 |
| 0.088000 | 0.25579 |
| 0.083000 | 0.24502 |
| 0.078000 | 0.22259 |
| 0.073000 | 0.20646 |
| 0.068000 | 0.19414 |
| 0.063000 | 0.18128 |
| 0.058000 | 0.14611 |
| 0.053000 | 0.11832 |
| 0.048000 | 0.13667 |
| 0.043000 | 0.15056 |
| 0.038000 | 0.14069 |
| 0.033000 | 0.14631 |
| 0.028000 | 0.16561 |
| 0.023000 | 0.17791 |
| 0.018000 | 0.17799 |
| 0.013000 | 0.17448 |
| 0.0080000 | 0.17334 |

| Column 1 | Column 2 |
|---|---|
| 0.0030000 | 0.16783 |
| -0.0020000 | 0.15885 |
| -0.0070000 | 0.15812 |
| -0.012000 | 0.16800 |
| -0.017000 | 0.17584 |
| -0.022000 | 0.17622 |
| -0.027000 | 0.17997 |
| -0.032000 | 0.18699 |
| -0.037000 | 0.19050 |
| -0.042000 | 0.20221 |
| -0.047000 | 0.22014 |
| -0.052000 | 0.23274 |
| -0.057000 | 0.23946 |
| -0.062000 | 0.23394 |
| -0.067000 | 0.22287 |
| -0.072000 | 0.20940 |
| -0.077000 | 0.18442 |
| -0.082000 | 0.15298 |
| -0.087000 | 0.12544 |
| -0.092000 | 0.10483 |
| -0.097000 | 0.081000 |
| -0.10200 | 0.044590 |
| -0.10700 | -0.0091100 |
| -0.11200 | -0.069410 |
| -0.11700 | -0.10750 |
| -0.12200 | -0.12860 |
| -0.12700 | -0.15369 |
| -0.13200 | -0.17326 |
| -0.13700 | -0.19792 |
| -0.14200 | -0.23970 |
| -0.14700 | -0.25921 |
| -0.15200 | -0.25457 |
| -0.15700 | -0.26712 |
| -0.16200 | -0.29121 |
| -0.16700 | -0.30488 |

| Column 1 | Column 2 |
|---|---|
| -0.17200 | -0.31816 |
| -0.17700 | -0.33636 |
| -0.18200 | -0.34679 |
| -0.18700 | -0.35969 |
| -0.19200 | -0.37292 |
| -0.19700 | -0.36566 |
| -0.20200 | -0.35100 |
| -0.20700 | -0.34349 |
| -0.21200 | -0.34060 |
| -0.21700 | -0.33724 |
| -0.22200 | -0.32758 |
| -0.22700 | -0.31563 |
| -0.23200 | -0.30907 |
| -0.23700 | -0.30526 |
| -0.24200 | -0.30353 |
| -0.24700 | -0.30248 |
| -0.25200 | -0.30394 |
| -0.25700 | -0.31750 |
| -0.26200 | -0.32828 |
| -0.26700 | -0.32909 |
| -0.27200 | -0.32804 |
| -0.27700 | -0.32403 |
| -0.28200 | -0.32105 |
| -0.28700 | -0.31646 |
| -0.29200 | -0.31241 |
| -0.29700 | -0.31699 |
| -0.30200 | -0.32209 |
| -0.30700 | -0.32250 |
| -0.31200 | -0.32470 |
| -0.31700 | -0.33299 |
| -0.32200 | -0.34433 |
| -0.32700 | -0.35416 |
| -0.33200 | -0.36693 |
| -0.33700 | -0.37480 |
| -0.34200 | -0.37653 |

| Column 1 | Column 2 |
|---|---|
| -0.34700 | -0.36703 |

### 8.3.15 CV-15

| Column 1 | Column 2 |
|---|---|
| -0.34800 | -0.33835 |
| -0.34300 | -0.30134 |
| -0.33800 | -0.32341 |
| -0.33300 | -0.30983 |
| -0.32800 | -0.29971 |
| -0.32300 | -0.28583 |
| -0.31800 | -0.27385 |
| -0.31300 | -0.25484 |
| -0.30800 | -0.23860 |
| -0.30300 | -0.24450 |
| -0.29800 | -0.25070 |
| -0.29300 | -0.24951 |
| -0.28800 | -0.25359 |
| -0.28300 | -0.26306 |
| -0.27800 | -0.27249 |
| -0.27300 | -0.27656 |
| -0.26800 | -0.27004 |
| -0.26300 | -0.26338 |
| -0.25800 | -0.26541 |
| -0.25300 | -0.28120 |
| -0.24800 | -0.28909 |
| -0.24300 | -0.27379 |
| -0.23800 | -0.27085 |
| -0.23300 | -0.27147 |
| -0.22800 | -0.25002 |
| -0.22300 | -0.22765 |
| -0.21800 | -0.21467 |
| -0.21300 | -0.20765 |
| -0.20800 | -0.20602 |
| -0.20300 | -0.20856 |
| -0.19800 | -0.21743 |

| Column 1 | Column 2 |
|---|---|
| -0.19300 | -0.21888 |
| -0.18800 | -0.21042 |
| -0.18300 | -0.20935 |
| -0.17800 | -0.20701 |
| -0.17300 | -0.19877 |
| -0.16800 | -0.19041 |
| -0.16300 | -0.17837 |
| -0.15800 | -0.16177 |
| -0.15300 | -0.14258 |
| -0.14800 | -0.12246 |
| -0.14300 | -0.10363 |
| -0.13800 | -0.096390 |
| -0.13300 | -0.10216 |
| -0.12800 | -0.10684 |
| -0.12300 | -0.063430 |
| -0.11800 | 0.043970 |
| -0.11300 | 0.12080 |
| -0.10800 | 0.13997 |
| -0.10300 | 0.16553 |
| -0.098000 | 0.18642 |
| -0.093000 | 0.19874 |
| -0.088000 | 0.21891 |
| -0.083000 | 0.22395 |
| -0.078000 | 0.21929 |
| -0.073000 | 0.22170 |
| -0.068000 | 0.22665 |
| -0.063000 | 0.23413 |
| -0.058000 | 0.24411 |
| -0.053000 | 0.25203 |
| -0.048000 | 0.25681 |
| -0.043000 | 0.25534 |
| -0.038000 | 0.25210 |
| -0.033000 | 0.25030 |
| -0.028000 | 0.25038 |
| -0.023000 | 0.24791 |

| Column 1 | Column 2 |
|---|---|
| -0.018000 | 0.24734 |
| -0.013000 | 0.25093 |
| -0.0080000 | 0.26196 |
| -0.0030000 | 0.34871 |
| 0.0020000 | 0.42450 |
| 0.0070000 | 0.36591 |
| 0.012000 | 0.30833 |
| 0.017000 | 0.30626 |
| 0.022000 | 0.30030 |
| 0.027000 | 0.29660 |
| 0.032000 | 0.29076 |
| 0.037000 | 0.28537 |
| 0.042000 | 0.28194 |
| 0.047000 | 0.28186 |
| 0.052000 | 0.28854 |
| 0.057000 | 0.29474 |
| 0.062000 | 0.29224 |
| 0.067000 | 0.28166 |
| 0.072000 | 0.26930 |
| 0.077000 | 0.25939 |
| 0.082000 | 0.25663 |
| 0.087000 | 0.25151 |
| 0.092000 | 0.21604 |
| 0.097000 | 0.11825 |
| 0.10200 | 0.013130 |
| 0.10700 | -7.3961E-4 |
| 0.11200 | 0.035050 |
| 0.11700 | 0.038880 |
| 0.12200 | 0.037350 |
| 0.12700 | 0.051560 |
| 0.13200 | 0.055850 |
| 0.13700 | 0.056100 |
| 0.14200 | 0.064160 |
| 0.14700 | 0.067390 |
| 0.15200 | 0.070130 |

| Column 1 | Column 2 |
|---|---|
| 0.15700 | 0.078380 |
| 0.16200 | 0.081060 |
| 0.16700 | 0.081270 |
| 0.17200 | 0.084100 |
| 0.17700 | 0.092370 |
| 0.18200 | 0.099410 |
| 0.18700 | 0.093620 |
| 0.19200 | 0.094510 |
| 0.19700 | 0.12194 |
| 0.19800 | 0.12437 |
| 0.19300 | 0.12463 |
| 0.18800 | 0.10374 |
| 0.18300 | 0.11304 |
| 0.17800 | 0.11791 |
| 0.17300 | 0.12649 |
| 0.16800 | 0.13801 |
| 0.16300 | 0.14806 |
| 0.15800 | 0.15785 |
| 0.15300 | 0.16542 |
| 0.14800 | 0.17401 |
| 0.14300 | 0.17976 |
| 0.13800 | 0.18448 |
| 0.13300 | 0.18980 |
| 0.12800 | 0.19477 |
| 0.12300 | 0.19648 |
| 0.11800 | 0.19311 |
| 0.11300 | 0.18553 |
| 0.10800 | 0.18373 |
| 0.10300 | 0.19622 |
| 0.098000 | 0.21153 |
| 0.093000 | 0.22226 |
| 0.088000 | 0.22801 |
| 0.083000 | 0.21337 |
| 0.078000 | 0.19363 |
| 0.073000 | 0.20134 |

| Column 1 | Column 2 |
|---|---|
| 0.068000 | 0.22014 |
| 0.063000 | 0.22816 |
| 0.058000 | 0.22151 |
| 0.053000 | 0.21362 |
| 0.048000 | 0.21842 |
| 0.043000 | 0.22358 |
| 0.038000 | 0.22305 |
| 0.033000 | 0.22130 |
| 0.028000 | 0.21998 |
| 0.023000 | 0.21643 |
| 0.018000 | 0.21268 |
| 0.013000 | 0.21253 |
| 0.0080000 | 0.20937 |
| 0.0030000 | 0.20121 |
| -0.0020000 | 0.19647 |
| -0.0070000 | 0.19665 |
| -0.012000 | 0.19244 |
| -0.017000 | 0.18605 |
| -0.022000 | 0.18471 |
| -0.027000 | 0.18217 |
| -0.032000 | 0.17353 |
| -0.037000 | 0.16276 |
| -0.042000 | 0.15059 |
| -0.047000 | 0.13325 |
| -0.052000 | 0.11196 |
| -0.057000 | 0.094620 |
| -0.062000 | 0.087220 |
| -0.067000 | 0.085020 |
| -0.072000 | 0.079320 |
| -0.077000 | 0.067120 |
| -0.082000 | 0.051620 |
| -0.087000 | 0.037030 |
| -0.092000 | 0.020610 |
| -0.097000 | 7.5561E-4 |
| -0.10200 | -0.019310 |

| Column 1 | Column 2 |
|---|---|
| -0.10700 | -0.040320 |
| -0.11200 | -0.062510 |
| -0.11700 | -0.084950 |
| -0.12200 | -0.10837 |
| -0.12700 | -0.13224 |
| -0.13200 | -0.15511 |
| -0.13700 | -0.17584 |
| -0.14200 | -0.19598 |
| -0.14700 | -0.21492 |
| -0.15200 | -0.23036 |
| -0.15700 | -0.24381 |
| -0.16200 | -0.25670 |
| -0.16700 | -0.26827 |
| -0.17200 | -0.27809 |
| -0.17700 | -0.28758 |
| -0.18200 | -0.29675 |
| -0.18700 | -0.30449 |
| -0.19200 | -0.31048 |
| -0.19700 | -0.31388 |
| -0.20200 | -0.31495 |
| -0.20700 | -0.31643 |
| -0.21200 | -0.31892 |
| -0.21700 | -0.32085 |
| -0.22200 | -0.32156 |
| -0.22700 | -0.32037 |
| -0.23200 | -0.31866 |
| -0.23700 | -0.31684 |
| -0.24200 | -0.31499 |
| -0.24700 | -0.31254 |
| -0.25200 | -0.30998 |
| -0.25700 | -0.30902 |
| -0.26200 | -0.30731 |
| -0.26700 | -0.30408 |
| -0.27200 | -0.30080 |
| -0.27700 | -0.29868 |

| Column 1 | Column 2 |
|---|---|
| -0.28200 | -0.29713 |
| -0.28700 | -0.29551 |
| -0.29200 | -0.29285 |
| -0.29700 | -0.29005 |
| -0.30200 | -0.28896 |
| -0.30700 | -0.29221 |
| -0.31200 | -0.28606 |
| -0.31700 | -0.26576 |
| -0.32200 | -0.26156 |
| -0.32700 | -0.27044 |
| -0.33200 | -0.27587 |
| -0.33700 | -0.27725 |
| -0.34200 | -0.28180 |
| -0.34700 | -0.28134 |

### 8.3.16 CV-16

| Column 1 | Column 2 |
|---|---|
| -0.34800 | -0.26853 |
| -0.34300 | -0.24420 |
| -0.33800 | -0.26233 |
| -0.33300 | -0.25860 |
| -0.32800 | -0.26040 |
| -0.32300 | -0.26130 |
| -0.31800 | -0.25923 |
| -0.31300 | -0.25911 |
| -0.30800 | -0.25738 |
| -0.30300 | -0.25658 |
| -0.29800 | -0.25465 |
| -0.29300 | -0.25257 |
| -0.28800 | -0.24991 |
| -0.28300 | -0.24678 |
| -0.27800 | -0.24530 |
| -0.27300 | -0.24295 |
| -0.26800 | -0.24239 |
| -0.26300 | -0.24305 |

| Column 1 | Column 2 |
|---|---|
| -0.25800 | -0.24559 |
| -0.25300 | -0.25396 |
| -0.24800 | -0.26002 |
| -0.24300 | -0.31011 |
| -0.23800 | -0.33285 |
| -0.23300 | -0.25569 |
| -0.22800 | -0.22382 |
| -0.22300 | -0.23413 |
| -0.21800 | -0.21683 |
| -0.21300 | -0.20431 |
| -0.20800 | -0.19178 |
| -0.20300 | -0.18319 |
| -0.19800 | -0.17728 |
| -0.19300 | -0.17150 |
| -0.18800 | -0.16510 |
| -0.18300 | -0.15655 |
| -0.17800 | -0.14731 |
| -0.17300 | -0.13370 |
| -0.16800 | -0.12019 |
| -0.16300 | -0.10634 |
| -0.15800 | -0.091050 |
| -0.15300 | -0.073080 |
| -0.14800 | -0.053930 |
| -0.14300 | -0.033430 |
| -0.13800 | -0.011270 |
| -0.13300 | 0.011890 |
| -0.12800 | 0.036550 |
| -0.12300 | 0.060170 |
| -0.11800 | 0.082670 |
| -0.11300 | 0.10522 |
| -0.10800 | 0.12670 |
| -0.10300 | 0.14596 |
| -0.098000 | 0.16544 |
| -0.093000 | 0.18455 |
| -0.088000 | 0.20063 |

| Column 1 | Column 2 |
|---|---|
| -0.083000 | 0.21338 |
| -0.078000 | 0.22513 |
| -0.073000 | 0.23616 |
| -0.068000 | 0.24551 |
| -0.063000 | 0.25378 |
| -0.058000 | 0.26029 |
| -0.053000 | 0.26479 |
| -0.048000 | 0.26740 |
| -0.043000 | 0.26896 |
| -0.038000 | 0.26850 |
| -0.033000 | 0.26692 |
| -0.028000 | 0.26518 |
| -0.023000 | 0.26341 |
| -0.018000 | 0.26151 |
| -0.013000 | 0.27127 |
| -0.0080000 | 0.29490 |
| -0.0030000 | 0.29753 |
| 0.0020000 | 0.27281 |
| 0.0070000 | 0.25364 |
| 0.012000 | 0.24967 |
| 0.017000 | 0.24950 |
| 0.022000 | 0.24817 |
| 0.027000 | 0.24529 |
| 0.032000 | 0.24482 |
| 0.037000 | 0.25196 |
| 0.042000 | 0.26219 |
| 0.047000 | 0.26747 |
| 0.052000 | 0.26596 |
| 0.057000 | 0.26761 |
| 0.062000 | 0.27474 |
| 0.067000 | 0.27989 |
| 0.072000 | 0.28351 |
| 0.077000 | 0.28113 |
| 0.082000 | 0.27122 |
| 0.087000 | 0.26049 |

| Column 1 | Column 2 |
|---|---|
| 0.092000 | 0.24206 |
| 0.097000 | 0.21926 |
| 0.10200 | 0.21191 |
| 0.10700 | 0.20485 |
| 0.11200 | 0.17444 |
| 0.11700 | 0.15967 |
| 0.12200 | 0.17446 |
| 0.12700 | 0.17377 |
| 0.13200 | 0.16875 |
| 0.13700 | 0.17107 |
| 0.14200 | 0.17042 |
| 0.14700 | 0.17405 |
| 0.15200 | 0.17905 |
| 0.15700 | 0.18611 |
| 0.16200 | 0.19238 |
| 0.16700 | 0.19809 |
| 0.17200 | 0.20220 |
| 0.17700 | 0.20777 |
| 0.18200 | 0.20878 |
| 0.18700 | 0.21120 |
| 0.19200 | 0.21537 |
| 0.19700 | 0.21256 |
| 0.19800 | 0.22563 |
| 0.19300 | 0.22781 |
| 0.18800 | 0.20125 |
| 0.18300 | 0.20604 |
| 0.17800 | 0.20208 |
| 0.17300 | 0.20108 |
| 0.16800 | 0.20375 |
| 0.16300 | 0.20223 |
| 0.15800 | 0.20185 |
| 0.15300 | 0.19896 |
| 0.14800 | 0.19923 |
| 0.14300 | 0.19930 |
| 0.13800 | 0.19907 |

| Column 1 | Column 2 |
|---|---|
| 0.13300 | 0.19621 |
| 0.12800 | 0.19577 |
| 0.12300 | 0.19660 |
| 0.11800 | 0.19652 |
| 0.11300 | 0.19880 |
| 0.10800 | 0.20089 |
| 0.10300 | 0.20200 |
| 0.098000 | 0.20242 |
| 0.093000 | 0.20387 |
| 0.088000 | 0.20535 |
| 0.083000 | 0.20800 |
| 0.078000 | 0.21113 |
| 0.073000 | 0.21255 |
| 0.068000 | 0.21159 |
| 0.063000 | 0.21000 |
| 0.058000 | 0.20970 |
| 0.053000 | 0.20892 |
| 0.048000 | 0.20786 |
| 0.043000 | 0.20720 |
| 0.038000 | 0.20685 |
| 0.033000 | 0.20634 |
| 0.028000 | 0.20699 |
| 0.023000 | 0.21243 |
| 0.018000 | 0.21751 |
| 0.013000 | 0.21333 |
| 0.0080000 | 0.20559 |
| 0.0030000 | 0.19962 |
| -0.0020000 | 0.19346 |
| -0.0070000 | 0.18790 |
| -0.012000 | 0.18270 |
| -0.017000 | 0.17801 |
| -0.022000 | 0.17380 |
| -0.027000 | 0.16993 |
| -0.032000 | 0.16495 |
| -0.037000 | 0.15882 |

| Column 1 | Column 2 |
|---|---|
| -0.042000 | 0.15260 |
| -0.047000 | 0.14497 |
| -0.052000 | 0.13588 |
| -0.057000 | 0.12541 |
| -0.062000 | 0.11356 |
| -0.067000 | 0.10040 |
| -0.072000 | 0.085910 |
| -0.077000 | 0.068940 |
| -0.082000 | 0.049650 |
| -0.087000 | 0.030020 |
| -0.092000 | 0.0099400 |
| -0.097000 | -0.011490 |
| -0.10200 | -0.033880 |
| -0.10700 | -0.056810 |
| -0.11200 | -0.081600 |
| -0.11700 | -0.10605 |
| -0.12200 | -0.13004 |
| -0.12700 | -0.15386 |
| -0.13200 | -0.17700 |
| -0.13700 | -0.20097 |
| -0.14200 | -0.22612 |
| -0.14700 | -0.25923 |
| -0.15200 | -0.28213 |
| -0.15700 | -0.27748 |
| -0.16200 | -0.27788 |
| -0.16700 | -0.29409 |
| -0.17200 | -0.30641 |
| -0.17700 | -0.31482 |
| -0.18200 | -0.32126 |
| -0.18700 | -0.32578 |
| -0.19200 | -0.32932 |
| -0.19700 | -0.33224 |
| -0.20200 | -0.33521 |
| -0.20700 | -0.33736 |
| -0.21200 | -0.33887 |

| Column 1 | Column 2 |
|---|---|
| -0.21700 | -0.34045 |
| -0.22200 | -0.34328 |
| -0.22700 | -0.34467 |
| -0.23200 | -0.34497 |
| -0.23700 | -0.34654 |
| -0.24200 | -0.34824 |
| -0.24700 | -0.34703 |
| -0.25200 | -0.34471 |
| -0.25700 | -0.34411 |
| -0.26200 | -0.34313 |
| -0.26700 | -0.34221 |
| -0.27200 | -0.34083 |
| -0.27700 | -0.34034 |
| -0.28200 | -0.33854 |
| -0.28700 | -0.33733 |
| -0.29200 | -0.33653 |
| -0.29700 | -0.33484 |
| -0.30200 | -0.33434 |
| -0.30700 | -0.33361 |
| -0.31200 | -0.33388 |
| -0.31700 | -0.33202 |
| -0.32200 | -0.33256 |
| -0.32700 | -0.33049 |
| -0.33200 | -0.33133 |
| -0.33700 | -0.33000 |
| -0.34200 | -0.32925 |
| -0.34700 | -0.33123 |

### 8.3.17 CV-17

| Column 1 | Column 2 |
|---|---|
| -0.34800 | -0.30269 |
| -0.34300 | -0.29107 |
| -0.33800 | -0.31219 |
| -0.33300 | -0.30356 |
| -0.32800 | -0.30215 |

| Column 1 | Column 2 |
|---|---|
| -0.32300 | -0.30108 |
| -0.31800 | -0.29782 |
| -0.31300 | -0.29749 |
| -0.30800 | -0.29382 |
| -0.30300 | -0.29272 |
| -0.29800 | -0.29048 |
| -0.29300 | -0.28953 |
| -0.28800 | -0.28745 |
| -0.28300 | -0.28676 |
| -0.27800 | -0.28553 |
| -0.27300 | -0.28364 |
| -0.26800 | -0.28220 |
| -0.26300 | -0.28011 |
| -0.25800 | -0.27822 |
| -0.25300 | -0.27569 |
| -0.24800 | -0.27415 |
| -0.24300 | -0.27177 |
| -0.23800 | -0.26920 |
| -0.23300 | -0.26612 |
| -0.22800 | -0.26260 |
| -0.22300 | -0.25897 |
| -0.21800 | -0.25495 |
| -0.21300 | -0.25064 |
| -0.20800 | -0.24546 |
| -0.20300 | -0.23943 |
| -0.19800 | -0.23244 |
| -0.19300 | -0.22379 |
| -0.18800 | -0.21350 |
| -0.18300 | -0.20318 |
| -0.17800 | -0.19137 |
| -0.17300 | -0.17767 |
| -0.16800 | -0.16205 |
| -0.16300 | -0.14567 |
| -0.15800 | -0.12934 |
| -0.15300 | -0.11150 |

| Column 1 | Column 2 |
|---|---|
| -0.14800 | -0.092390 |
| -0.14300 | -0.070910 |
| -0.13800 | -0.047290 |
| -0.13300 | -0.022920 |
| -0.12800 | 0.0013100 |
| -0.12300 | 0.024800 |
| -0.11800 | 0.047670 |
| -0.11300 | 0.070980 |
| -0.10800 | 0.094210 |
| -0.10300 | 0.11621 |
| -0.098000 | 0.13824 |
| -0.093000 | 0.15970 |
| -0.088000 | 0.17884 |
| -0.083000 | 0.19620 |
| -0.078000 | 0.21191 |
| -0.073000 | 0.22478 |
| -0.068000 | 0.23652 |
| -0.063000 | 0.24674 |
| -0.058000 | 0.25426 |
| -0.053000 | 0.26031 |
| -0.048000 | 0.26564 |
| -0.043000 | 0.27120 |
| -0.038000 | 0.27490 |
| -0.033000 | 0.27665 |
| -0.028000 | 0.27834 |
| -0.023000 | 0.27899 |
| -0.018000 | 0.27903 |
| -0.013000 | 0.27946 |
| -0.0080000 | 0.27893 |
| -0.0030000 | 0.27798 |
| 0.0020000 | 0.27759 |
| 0.0070000 | 0.27640 |
| 0.012000 | 0.27493 |
| 0.017000 | 0.27358 |
| 0.022000 | 0.27265 |

| Column 1 | Column 2 |
|---|---|
| 0.027000 | 0.27167 |
| 0.032000 | 0.27087 |
| 0.037000 | 0.27027 |
| 0.042000 | 0.26944 |
| 0.047000 | 0.26900 |
| 0.052000 | 0.26824 |
| 0.057000 | 0.26675 |
| 0.062000 | 0.26489 |
| 0.067000 | 0.26402 |
| 0.072000 | 0.26317 |
| 0.077000 | 0.26237 |
| 0.082000 | 0.26189 |
| 0.087000 | 0.26182 |
| 0.092000 | 0.26144 |
| 0.097000 | 0.26095 |
| 0.10200 | 0.26034 |
| 0.10700 | 0.25858 |
| 0.11200 | 0.25843 |
| 0.11700 | 0.25838 |
| 0.12200 | 0.25845 |
| 0.12700 | 0.25747 |
| 0.13200 | 0.25633 |
| 0.13700 | 0.25566 |
| 0.14200 | 0.25547 |
| 0.14700 | 0.25548 |
| 0.15200 | 0.25488 |
| 0.15700 | 0.25418 |
| 0.16200 | 0.25284 |
| 0.16700 | 0.25501 |
| 0.17200 | 0.25312 |
| 0.17700 | 0.25390 |
| 0.18200 | 0.25329 |
| 0.18700 | 0.25518 |
| 0.19200 | 0.25758 |
| 0.19700 | 0.25070 |

| Column 1 | Column 2 |
|---|---|
| 0.19800 | 0.26735 |
| 0.19300 | 0.26240 |
| 0.18800 | 0.23717 |
| 0.18300 | 0.24187 |
| 0.17800 | 0.23943 |
| 0.17300 | 0.23803 |
| 0.16800 | 0.23901 |
| 0.16300 | 0.23727 |
| 0.15800 | 0.23742 |
| 0.15300 | 0.23596 |
| 0.14800 | 0.23675 |
| 0.14300 | 0.23562 |
| 0.13800 | 0.23624 |
| 0.13300 | 0.23670 |
| 0.12800 | 0.23694 |
| 0.12300 | 0.23789 |
| 0.11800 | 0.23848 |
| 0.11300 | 0.23905 |
| 0.10800 | 0.24059 |
| 0.10300 | 0.24396 |
| 0.098000 | 0.24470 |
| 0.093000 | 0.24210 |
| 0.088000 | 0.23738 |
| 0.083000 | 0.23302 |
| 0.078000 | 0.22964 |
| 0.073000 | 0.22742 |
| 0.068000 | 0.22519 |
| 0.063000 | 0.22210 |
| 0.058000 | 0.22023 |
| 0.053000 | 0.21949 |
| 0.048000 | 0.21920 |
| 0.043000 | 0.21899 |
| 0.038000 | 0.21881 |
| 0.033000 | 0.21832 |
| 0.028000 | 0.21779 |

| Column 1 | Column 2 |
|---|---|
| 0.023000 | 0.21662 |
| 0.018000 | 0.21467 |
| 0.013000 | 0.21295 |
| 0.0080000 | 0.21178 |
| 0.0030000 | 0.21029 |
| -0.0020000 | 0.20740 |
| -0.0070000 | 0.20398 |
| -0.012000 | 0.20030 |
| -0.017000 | 0.19597 |
| -0.022000 | 0.19092 |
| -0.027000 | 0.18546 |
| -0.032000 | 0.17893 |
| -0.037000 | 0.17114 |
| -0.042000 | 0.16308 |
| -0.047000 | 0.15315 |
| -0.052000 | 0.14215 |
| -0.057000 | 0.12986 |
| -0.062000 | 0.11569 |
| -0.067000 | 0.10053 |
| -0.072000 | 0.083200 |
| -0.077000 | 0.063630 |
| -0.082000 | 0.032080 |
| -0.087000 | -0.0010300 |
| -0.092000 | -0.014150 |
| -0.097000 | -0.025360 |
| -0.10200 | -0.047760 |
| -0.10700 | -0.074060 |
| -0.11200 | -0.10062 |
| -0.11700 | -0.12492 |
| -0.12200 | -0.14967 |
| -0.12700 | -0.17467 |
| -0.13200 | -0.19862 |
| -0.13700 | -0.22065 |
| -0.14200 | -0.24066 |
| -0.14700 | -0.25974 |

| Column 1 | Column 2 |
|---|---|
| -0.15200 | -0.27732 |
| -0.15700 | -0.29311 |
| -0.16200 | -0.30676 |
| -0.16700 | -0.31857 |
| -0.17200 | -0.32871 |
| -0.17700 | -0.33856 |
| -0.18200 | -0.34673 |
| -0.18700 | -0.35301 |
| -0.19200 | -0.35852 |
| -0.19700 | -0.36244 |
| -0.20200 | -0.36510 |
| -0.20700 | -0.36686 |
| -0.21200 | -0.36819 |
| -0.21700 | -0.36795 |
| -0.22200 | -0.36722 |
| -0.22700 | -0.36676 |
| -0.23200 | -0.36616 |
| -0.23700 | -0.36545 |
| -0.24200 | -0.36529 |
| -0.24700 | -0.36472 |
| -0.25200 | -0.36405 |
| -0.25700 | -0.36464 |
| -0.26200 | -0.36414 |
| -0.26700 | -0.36312 |
| -0.27200 | -0.36192 |
| -0.27700 | -0.36168 |
| -0.28200 | -0.36028 |
| -0.28700 | -0.35873 |
| -0.29200 | -0.35779 |
| -0.29700 | -0.35730 |
| -0.30200 | -0.35671 |
| -0.30700 | -0.35483 |
| -0.31200 | -0.35470 |
| -0.31700 | -0.35281 |
| -0.32200 | -0.35297 |

| Column 1 | Column 2 |
|---|---|
| -0.32700 | -0.35085 |
| -0.33200 | -0.35087 |
| -0.33700 | -0.34896 |
| -0.34200 | -0.34792 |
| -0.34700 | -0.34937 |

## 8.3.18 CV-18

| Column 1 | Column 2 |
|---|---|
| -0.34800 | -0.32360 |
| -0.34300 | -0.31778 |
| -0.33800 | -0.32982 |
| -0.33300 | -0.32338 |
| -0.32800 | -0.32160 |
| -0.32300 | -0.32036 |
| -0.31800 | -0.31768 |
| -0.31300 | -0.31696 |
| -0.30800 | -0.31412 |
| -0.30300 | -0.31314 |
| -0.29800 | -0.31133 |
| -0.29300 | -0.31028 |
| -0.28800 | -0.30772 |
| -0.28300 | -0.30587 |
| -0.27800 | -0.30459 |
| -0.27300 | -0.30272 |
| -0.26800 | -0.30174 |
| -0.26300 | -0.30140 |
| -0.25800 | -0.30096 |
| -0.25300 | -0.29927 |
| -0.24800 | -0.29750 |
| -0.24300 | -0.29480 |
| -0.23800 | -0.29186 |
| -0.23300 | -0.28893 |
| -0.22800 | -0.28499 |
| -0.22300 | -0.27978 |
| -0.21800 | -0.27491 |

| Column 1 | Column 2 |
|---|---|
| -0.21300 | -0.27017 |
| -0.20800 | -0.26371 |
| -0.20300 | -0.25623 |
| -0.19800 | -0.24841 |
| -0.19300 | -0.23960 |
| -0.18800 | -0.22853 |
| -0.18300 | -0.21576 |
| -0.17800 | -0.20049 |
| -0.17300 | -0.18455 |
| -0.16800 | -0.17045 |
| -0.16300 | -0.15558 |
| -0.15800 | -0.13826 |
| -0.15300 | -0.11856 |
| -0.14800 | -0.098230 |
| -0.14300 | -0.077420 |
| -0.13800 | -0.054050 |
| -0.13300 | -0.027930 |
| -0.12800 | 5.6696E-4 |
| -0.12300 | 0.030160 |
| -0.11800 | 0.057680 |
| -0.11300 | 0.082670 |
| -0.10800 | 0.10719 |
| -0.10300 | 0.12816 |
| -0.098000 | 0.14732 |
| -0.093000 | 0.16922 |
| -0.088000 | 0.18687 |
| -0.083000 | 0.20150 |
| -0.078000 | 0.21914 |
| -0.073000 | 0.23625 |
| -0.068000 | 0.25156 |
| -0.063000 | 0.26389 |
| -0.058000 | 0.27241 |
| -0.053000 | 0.27868 |
| -0.048000 | 0.28524 |
| -0.043000 | 0.29228 |

| Column 1 | Column 2 |
|---|---|
| -0.038000 | 0.29654 |
| -0.033000 | 0.29874 |
| -0.028000 | 0.29972 |
| -0.023000 | 0.29837 |
| -0.018000 | 0.29566 |
| -0.013000 | 0.29384 |
| -0.0080000 | 0.29270 |
| -0.0030000 | 0.29117 |
| 0.0020000 | 0.29072 |
| 0.0070000 | 0.29135 |
| 0.012000 | 0.29092 |
| 0.017000 | 0.28891 |
| 0.022000 | 0.28669 |
| 0.027000 | 0.28514 |
| 0.032000 | 0.28410 |
| 0.037000 | 0.28341 |
| 0.042000 | 0.27995 |
| 0.047000 | 0.27035 |
| 0.052000 | 0.26805 |
| 0.057000 | 0.27492 |
| 0.062000 | 0.27407 |
| 0.067000 | 0.27228 |
| 0.072000 | 0.27461 |
| 0.077000 | 0.27401 |
| 0.082000 | 0.27254 |
| 0.087000 | 0.27270 |
| 0.092000 | 0.27297 |
| 0.097000 | 0.27262 |
| 0.10200 | 0.27330 |
| 0.10700 | 0.27388 |
| 0.11200 | 0.27367 |
| 0.11700 | 0.27315 |
| 0.12200 | 0.27295 |
| 0.12700 | 0.27214 |
| 0.13200 | 0.27155 |

| Column 1 | Column 2 |
|---|---|
| 0.13700 | 0.27035 |
| 0.14200 | 0.26928 |
| 0.14700 | 0.26846 |
| 0.15200 | 0.26697 |
| 0.15700 | 0.26649 |
| 0.16200 | 0.26504 |
| 0.16700 | 0.26565 |
| 0.17200 | 0.26508 |
| 0.17700 | 0.26561 |
| 0.18200 | 0.26424 |
| 0.18700 | 0.26394 |
| 0.19200 | 0.26419 |
| 0.19700 | 0.25896 |
| 0.19800 | 0.26839 |
| 0.19300 | 0.26474 |
| 0.18800 | 0.25372 |
| 0.18300 | 0.25962 |
| 0.17800 | 0.26005 |
| 0.17300 | 0.26049 |
| 0.16800 | 0.25937 |
| 0.16300 | 0.25696 |
| 0.15800 | 0.25553 |
| 0.15300 | 0.25306 |
| 0.14800 | 0.25168 |
| 0.14300 | 0.25119 |
| 0.13800 | 0.25225 |
| 0.13300 | 0.25268 |
| 0.12800 | 0.25238 |
| 0.12300 | 0.25251 |
| 0.11800 | 0.25214 |
| 0.11300 | 0.25066 |
| 0.10800 | 0.24905 |
| 0.10300 | 0.24730 |
| 0.098000 | 0.24588 |
| 0.093000 | 0.24595 |

| Column 1 | Column 2 |
|---|---|
| 0.088000 | 0.24548 |
| 0.083000 | 0.24424 |
| 0.078000 | 0.24302 |
| 0.073000 | 0.24227 |
| 0.068000 | 0.24177 |
| 0.063000 | 0.24094 |
| 0.058000 | 0.23994 |
| 0.053000 | 0.23867 |
| 0.048000 | 0.23713 |
| 0.043000 | 0.23569 |
| 0.038000 | 0.23417 |
| 0.033000 | 0.23255 |
| 0.028000 | 0.23098 |
| 0.023000 | 0.22898 |
| 0.018000 | 0.22660 |
| 0.013000 | 0.22332 |
| 0.0080000 | 0.21940 |
| 0.0030000 | 0.21554 |
| -0.0020000 | 0.21236 |
| -0.0070000 | 0.20944 |
| -0.012000 | 0.20424 |
| -0.017000 | 0.19841 |
| -0.022000 | 0.19445 |
| -0.027000 | 0.19009 |
| -0.032000 | 0.18407 |
| -0.037000 | 0.17705 |
| -0.042000 | 0.16834 |
| -0.047000 | 0.15750 |
| -0.052000 | 0.14489 |
| -0.057000 | 0.13101 |
| -0.062000 | 0.11610 |
| -0.067000 | 0.099960 |
| -0.072000 | 0.081820 |
| -0.077000 | 0.060970 |
| -0.082000 | 0.038640 |

| Column 1 | Column 2 |
|---|---|
| -0.087000 | 0.014710 |
| -0.092000 | -0.011400 |
| -0.097000 | -0.037720 |
| -0.10200 | -0.064280 |
| -0.10700 | -0.091210 |
| -0.11200 | -0.11712 |
| -0.11700 | -0.14396 |
| -0.12200 | -0.17645 |
| -0.12700 | -0.21268 |
| -0.13200 | -0.24084 |
| -0.13700 | -0.25887 |
| -0.14200 | -0.27509 |
| -0.14700 | -0.28848 |
| -0.15200 | -0.29918 |
| -0.15700 | -0.31304 |
| -0.16200 | -0.32899 |
| -0.16700 | -0.34367 |
| -0.17200 | -0.35594 |
| -0.17700 | -0.36513 |
| -0.18200 | -0.37208 |
| -0.18700 | -0.37732 |
| -0.19200 | -0.38129 |
| -0.19700 | -0.38472 |
| -0.20200 | -0.38567 |
| -0.20700 | -0.38428 |
| -0.21200 | -0.38241 |
| -0.21700 | -0.37643 |
| -0.22200 | -0.36646 |
| -0.22700 | -0.36642 |
| -0.23200 | -0.37906 |
| -0.23700 | -0.38382 |
| -0.24200 | -0.37587 |
| -0.24700 | -0.37243 |
| -0.25200 | -0.37756 |
| -0.25700 | -0.38050 |

| Column 1 | Column 2 |
|---|---|
| -0.26200 | -0.37982 |
| -0.26700 | -0.38015 |
| -0.27200 | -0.38065 |
| -0.27700 | -0.38063 |
| -0.28200 | -0.37902 |
| -0.28700 | -0.37825 |
| -0.29200 | -0.37938 |
| -0.29700 | -0.37961 |
| -0.30200 | -0.37900 |
| -0.30700 | -0.37800 |
| -0.31200 | -0.37602 |
| -0.31700 | -0.37295 |
| -0.32200 | -0.36937 |
| -0.32700 | -0.36447 |
| -0.33200 | -0.36082 |
| -0.33700 | -0.35716 |
| -0.34200 | -0.35424 |
| -0.34700 | -0.34816 |

### 8.3.19 CV-19

| Column 1 | Column 2 |
|---|---|
| -0.34800 | -0.34091 |
| -0.34300 | -0.34016 |
| -0.33800 | -0.32247 |
| -0.33300 | -0.33573 |
| -0.32800 | -0.35645 |
| -0.32300 | -0.35843 |
| -0.31800 | -0.35570 |
| -0.31300 | -0.35609 |
| -0.30800 | -0.35135 |
| -0.30300 | -0.34653 |
| -0.29800 | -0.34195 |
| -0.29300 | -0.33753 |
| -0.28800 | -0.33416 |
| -0.28300 | -0.33188 |

| Column 1 | Column 2 |
|---|---|
| -0.27800 | -0.33018 |
| -0.27300 | -0.32730 |
| -0.26800 | -0.32478 |
| -0.26300 | -0.32206 |
| -0.25800 | -0.31925 |
| -0.25300 | -0.31632 |
| -0.24800 | -0.31331 |
| -0.24300 | -0.31022 |
| -0.23800 | -0.30657 |
| -0.23300 | -0.30253 |
| -0.22800 | -0.29778 |
| -0.22300 | -0.29305 |
| -0.21800 | -0.28863 |
| -0.21300 | -0.28307 |
| -0.20800 | -0.27561 |
| -0.20300 | -0.26780 |
| -0.19800 | -0.25938 |
| -0.19300 | -0.25049 |
| -0.18800 | -0.24170 |
| -0.18300 | -0.23191 |
| -0.17800 | -0.22055 |
| -0.17300 | -0.20775 |
| -0.16800 | -0.19364 |
| -0.16300 | -0.17735 |
| -0.15800 | -0.15841 |
| -0.15300 | -0.13721 |
| -0.14800 | -0.11475 |
| -0.14300 | -0.091120 |
| -0.13800 | -0.066430 |
| -0.13300 | -0.040970 |
| -0.12800 | -0.014520 |
| -0.12300 | 0.011980 |
| -0.11800 | 0.038060 |
| -0.11300 | 0.064370 |
| -0.10800 | 0.090480 |

| Column 1 | Column 2 |
|---|---|
| -0.10300 | 0.11537 |
| -0.098000 | 0.13941 |
| -0.093000 | 0.16262 |
| -0.088000 | 0.18450 |
| -0.083000 | 0.20474 |
| -0.078000 | 0.22295 |
| -0.073000 | 0.23910 |
| -0.068000 | 0.25319 |
| -0.063000 | 0.26543 |
| -0.058000 | 0.27575 |
| -0.053000 | 0.28396 |
| -0.048000 | 0.29090 |
| -0.043000 | 0.29665 |
| -0.038000 | 0.30056 |
| -0.033000 | 0.30409 |
| -0.028000 | 0.30649 |
| -0.023000 | 0.30743 |
| -0.018000 | 0.30857 |
| -0.013000 | 0.30950 |
| -0.0080000 | 0.30977 |
| -0.0030000 | 0.30929 |
| 0.0020000 | 0.30895 |
| 0.0070000 | 0.30867 |
| 0.012000 | 0.30820 |
| 0.017000 | 0.30770 |
| 0.022000 | 0.30672 |
| 0.027000 | 0.30580 |
| 0.032000 | 0.30553 |
| 0.037000 | 0.30502 |
| 0.042000 | 0.30377 |
| 0.047000 | 0.30307 |
| 0.052000 | 0.30270 |
| 0.057000 | 0.30172 |
| 0.062000 | 0.30060 |
| 0.067000 | 0.30009 |

| Column 1 | Column 2 |
|---|---|
| 0.072000 | 0.29910 |
| 0.077000 | 0.29812 |
| 0.082000 | 0.29790 |
| 0.087000 | 0.29732 |
| 0.092000 | 0.29630 |
| 0.097000 | 0.29587 |
| 0.10200 | 0.29729 |
| 0.10700 | 0.29954 |
| 0.11200 | 0.30398 |
| 0.11700 | 0.30614 |
| 0.12200 | 0.30340 |
| 0.12700 | 0.30477 |
| 0.13200 | 0.30719 |
| 0.13700 | 0.30045 |
| 0.14200 | 0.29124 |
| 0.14700 | 0.28760 |
| 0.15200 | 0.27820 |
| 0.15700 | 0.27555 |
| 0.16200 | 0.29683 |
| 0.16700 | 0.31114 |
| 0.17200 | 0.30562 |
| 0.17700 | 0.29668 |
| 0.18200 | 0.28365 |
| 0.18700 | 0.27648 |
| 0.19200 | 0.28291 |
| 0.19700 | 0.28132 |
| 0.19800 | 0.27620 |
| 0.19300 | 0.27087 |
| 0.18800 | 0.26771 |
| 0.18300 | 0.26775 |
| 0.17800 | 0.26569 |
| 0.17300 | 0.26540 |
| 0.16800 | 0.26410 |
| 0.16300 | 0.26293 |
| 0.15800 | 0.26172 |

| Column 1 | Column 2 |
|---|---|
| 0.15300 | 0.26222 |
| 0.14800 | 0.26113 |
| 0.14300 | 0.25996 |
| 0.13800 | 0.25915 |
| 0.13300 | 0.25830 |
| 0.12800 | 0.25772 |
| 0.12300 | 0.25665 |
| 0.11800 | 0.25570 |
| 0.11300 | 0.25508 |
| 0.10800 | 0.25465 |
| 0.10300 | 0.25389 |
| 0.098000 | 0.25315 |
| 0.093000 | 0.25239 |
| 0.088000 | 0.25191 |
| 0.083000 | 0.25072 |
| 0.078000 | 0.24976 |
| 0.073000 | 0.25014 |
| 0.068000 | 0.24967 |
| 0.063000 | 0.24899 |
| 0.058000 | 0.24891 |
| 0.053000 | 0.24779 |
| 0.048000 | 0.24643 |
| 0.043000 | 0.24597 |
| 0.038000 | 0.24490 |
| 0.033000 | 0.24327 |
| 0.028000 | 0.24143 |
| 0.023000 | 0.23915 |
| 0.018000 | 0.23749 |
| 0.013000 | 0.23640 |
| 0.0080000 | 0.23463 |
| 0.0030000 | 0.23189 |
| -0.0020000 | 0.22856 |
| -0.0070000 | 0.22521 |
| -0.012000 | 0.22141 |
| -0.017000 | 0.21596 |

| Column 1 | Column 2 |
|---|---|
| -0.022000 | 0.21044 |
| -0.027000 | 0.20469 |
| -0.032000 | 0.19713 |
| -0.037000 | 0.18880 |
| -0.042000 | 0.17913 |
| -0.047000 | 0.16752 |
| -0.052000 | 0.15443 |
| -0.057000 | 0.13972 |
| -0.062000 | 0.12356 |
| -0.067000 | 0.10548 |
| -0.072000 | 0.085430 |
| -0.077000 | 0.063820 |
| -0.082000 | 0.040830 |
| -0.087000 | 0.016670 |
| -0.092000 | -0.011320 |
| -0.097000 | -0.041130 |
| -0.10200 | -0.068140 |
| -0.10700 | -0.096460 |
| -0.11200 | -0.12766 |
| -0.11700 | -0.15715 |
| -0.12200 | -0.18396 |
| -0.12700 | -0.21047 |
| -0.13200 | -0.23634 |
| -0.13700 | -0.26029 |
| -0.14200 | -0.28285 |
| -0.14700 | -0.30312 |
| -0.15200 | -0.32121 |
| -0.15700 | -0.33828 |
| -0.16200 | -0.35309 |
| -0.16700 | -0.36463 |
| -0.17200 | -0.37457 |
| -0.17700 | -0.38285 |
| -0.18200 | -0.38883 |
| -0.18700 | -0.39370 |
| -0.19200 | -0.39889 |

| Column 1 | Column 2 |
| --- | --- |
| -0.19700 | -0.40396 |
| -0.20200 | -0.40712 |
| -0.20700 | -0.41007 |
| -0.21200 | -0.41272 |
| -0.21700 | -0.41348 |
| -0.22200 | -0.41374 |
| -0.22700 | -0.41372 |
| -0.23200 | -0.41218 |
| -0.23700 | -0.40948 |
| -0.24200 | -0.40653 |
| -0.24700 | -0.40391 |
| -0.25200 | -0.40216 |
| -0.25700 | -0.40245 |
| -0.26200 | -0.40348 |
| -0.26700 | -0.40144 |
| -0.27200 | -0.39876 |
| -0.27700 | -0.39711 |
| -0.28200 | -0.39587 |
| -0.28700 | -0.39454 |
| -0.29200 | -0.39277 |
| -0.29700 | -0.39102 |
| -0.30200 | -0.39036 |
| -0.30700 | -0.39089 |
| -0.31200 | -0.38942 |
| -0.31700 | -0.38983 |
| -0.32200 | -0.39096 |
| -0.32700 | -0.39294 |
| -0.33200 | -0.39337 |
| -0.33700 | -0.39361 |
| -0.34200 | -0.39095 |
| -0.34700 | -0.37024 |

### 8.3.20 CV-20

| Column 1 | Column 2 |
| --- | --- |
| -0.34800 | -0.36435 |

| Column 1 | Column 2 |
|---|---|
| -0.34300 | -0.36392 |
| -0.33800 | -0.34948 |
| -0.33300 | -0.36074 |
| -0.32800 | -0.36227 |
| -0.32300 | -0.36083 |
| -0.31800 | -0.35717 |
| -0.31300 | -0.35200 |
| -0.30800 | -0.34868 |
| -0.30300 | -0.34467 |
| -0.29800 | -0.34286 |
| -0.29300 | -0.33968 |
| -0.28800 | -0.33810 |
| -0.28300 | -0.33732 |
| -0.27800 | -0.33668 |
| -0.27300 | -0.33538 |
| -0.26800 | -0.33338 |
| -0.26300 | -0.33148 |
| -0.25800 | -0.32943 |
| -0.25300 | -0.32772 |
| -0.24800 | -0.32513 |
| -0.24300 | -0.32225 |
| -0.23800 | -0.32242 |
| -0.23300 | -0.33554 |
| -0.22800 | -0.35729 |
| -0.22300 | -0.36706 |
| -0.21800 | -0.35957 |
| -0.21300 | -0.33802 |
| -0.20800 | -0.30857 |
| -0.20300 | -0.28632 |
| -0.19800 | -0.27257 |
| -0.19300 | -0.25932 |
| -0.18800 | -0.24706 |
| -0.18300 | -0.23554 |
| -0.17800 | -0.22180 |
| -0.17300 | -0.20609 |

| Column 1 | Column 2 |
|---|---|
| -0.16800 | -0.19000 |
| -0.16300 | -0.17338 |
| -0.15800 | -0.15454 |
| -0.15300 | -0.13476 |
| -0.14800 | -0.11303 |
| -0.14300 | -0.089030 |
| -0.13800 | -0.064390 |
| -0.13300 | -0.036370 |
| -0.12800 | -0.010230 |
| -0.12300 | 0.038430 |
| -0.11800 | 0.10352 |
| -0.11300 | 0.11226 |
| -0.10800 | 0.093090 |
| -0.10300 | 0.12161 |
| -0.098000 | 0.17305 |
| -0.093000 | 0.20329 |
| -0.088000 | 0.22023 |
| -0.083000 | 0.24193 |
| -0.078000 | 0.26172 |
| -0.073000 | 0.27554 |
| -0.068000 | 0.28760 |
| -0.063000 | 0.29799 |
| -0.058000 | 0.30629 |
| -0.053000 | 0.31171 |
| -0.048000 | 0.31518 |
| -0.043000 | 0.31794 |
| -0.038000 | 0.32096 |
| -0.033000 | 0.32422 |
| -0.028000 | 0.32702 |
| -0.023000 | 0.32779 |
| -0.018000 | 0.32719 |
| -0.013000 | 0.32759 |
| -0.0080000 | 0.32744 |
| -0.0030000 | 0.32615 |
| 0.0020000 | 0.32589 |

| Column 1 | Column 2 |
|---|---|
| 0.0070000 | 0.32611 |
| 0.012000 | 0.32638 |
| 0.017000 | 0.32711 |
| 0.022000 | 0.32735 |
| 0.027000 | 0.32656 |
| 0.032000 | 0.32667 |
| 0.037000 | 0.32771 |
| 0.042000 | 0.32613 |
| 0.047000 | 0.32513 |
| 0.052000 | 0.32690 |
| 0.057000 | 0.32567 |
| 0.062000 | 0.31800 |
| 0.067000 | 0.31122 |
| 0.072000 | 0.31281 |
| 0.077000 | 0.31774 |
| 0.082000 | 0.31327 |
| 0.087000 | 0.29641 |
| 0.092000 | 0.27434 |
| 0.097000 | 0.25849 |
| 0.10200 | 0.26316 |
| 0.10700 | 0.23940 |
| 0.11200 | 0.18746 |
| 0.11700 | 0.19850 |
| 0.12200 | 0.23252 |
| 0.12700 | 0.23510 |
| 0.13200 | 0.24503 |
| 0.13700 | 0.26500 |
| 0.14200 | 0.28345 |
| 0.14700 | 0.30103 |
| 0.15200 | 0.31290 |
| 0.15700 | 0.31621 |
| 0.16200 | 0.31460 |
| 0.16700 | 0.31172 |
| 0.17200 | 0.30914 |
| 0.17700 | 0.30434 |

| Column 1 | Column 2 |
|---|---|
| 0.18200 | 0.30088 |
| 0.18700 | 0.29653 |
| 0.19200 | 0.29702 |
| 0.19700 | 0.29777 |
| 0.19800 | 0.28298 |
| 0.19300 | 0.28033 |
| 0.18800 | 0.28268 |
| 0.18300 | 0.28173 |
| 0.17800 | 0.28164 |
| 0.17300 | 0.28087 |
| 0.16800 | 0.27733 |
| 0.16300 | 0.27656 |
| 0.15800 | 0.27467 |
| 0.15300 | 0.27039 |
| 0.14800 | 0.26247 |
| 0.14300 | 0.25593 |
| 0.13800 | 0.25027 |
| 0.13300 | 0.24372 |
| 0.12800 | 0.23602 |
| 0.12300 | 0.22868 |
| 0.11800 | 0.22350 |
| 0.11300 | 0.22291 |
| 0.10800 | 0.22491 |
| 0.10300 | 0.22535 |
| 0.098000 | 0.22583 |
| 0.093000 | 0.22415 |
| 0.088000 | 0.22056 |
| 0.083000 | 0.20532 |
| 0.078000 | 0.18530 |
| 0.073000 | 0.17858 |
| 0.068000 | 0.17830 |
| 0.063000 | 0.18521 |
| 0.058000 | 0.21056 |
| 0.053000 | 0.26907 |
| 0.048000 | 0.35439 |

| Column 1 | Column 2 |
|---|---|
| 0.043000 | 0.42352 |
| 0.038000 | 0.43768 |
| 0.033000 | 0.40702 |
| 0.028000 | 0.36600 |
| 0.023000 | 0.32492 |
| 0.018000 | 0.29110 |
| 0.013000 | 0.26570 |
| 0.0080000 | 0.25065 |
| 0.0030000 | 0.25076 |
| -0.0020000 | 0.25276 |
| -0.0070000 | 0.25193 |
| -0.012000 | 0.24690 |
| -0.017000 | 0.23266 |
| -0.022000 | 0.20989 |
| -0.027000 | 0.18611 |
| -0.032000 | 0.17008 |
| -0.037000 | 0.15992 |
| -0.042000 | 0.14921 |
| -0.047000 | 0.13671 |
| -0.052000 | 0.12940 |
| -0.057000 | 0.12090 |
| -0.062000 | 0.094820 |
| -0.067000 | 0.059010 |
| -0.072000 | 0.025340 |
| -0.077000 | -0.0086000 |
| -0.082000 | -0.036130 |
| -0.087000 | -0.054060 |
| -0.092000 | -0.072390 |
| -0.097000 | -0.093160 |
| -0.10200 | -0.11893 |
| -0.10700 | -0.14775 |
| -0.11200 | -0.17133 |
| -0.11700 | -0.19803 |
| -0.12200 | -0.22686 |
| -0.12700 | -0.25026 |

| Column 1 | Column 2 |
|---|---|
| -0.13200 | -0.27421 |
| -0.13700 | -0.30126 |
| -0.14200 | -0.32546 |
| -0.14700 | -0.33574 |
| -0.15200 | -0.34162 |
| -0.15700 | -0.35125 |
| -0.16200 | -0.34975 |
| -0.16700 | -0.34217 |
| -0.17200 | -0.35659 |
| -0.17700 | -0.39593 |
| -0.18200 | -0.42504 |
| -0.18700 | -0.43472 |
| -0.19200 | -0.44175 |
| -0.19700 | -0.44728 |
| -0.20200 | -0.45022 |
| -0.20700 | -0.45122 |
| -0.21200 | -0.45037 |
| -0.21700 | -0.44398 |
| -0.22200 | -0.43130 |
| -0.22700 | -0.41791 |
| -0.23200 | -0.40622 |
| -0.23700 | -0.39623 |
| -0.24200 | -0.38768 |
| -0.24700 | -0.38355 |
| -0.25200 | -0.38479 |
| -0.25700 | -0.38657 |
| -0.26200 | -0.38553 |
| -0.26700 | -0.38645 |
| -0.27200 | -0.38721 |
| -0.27700 | -0.37817 |
| -0.28200 | -0.36390 |
| -0.28700 | -0.35014 |
| -0.29200 | -0.33288 |
| -0.29700 | -0.30245 |
| -0.30200 | -0.28926 |

| Column 1 | Column 2 |
|---|---|
| -0.30700 | -0.32795 |
| -0.31200 | -0.37771 |
| -0.31700 | -0.40370 |
| -0.32200 | -0.40530 |
| -0.32700 | -0.40304 |
| -0.33200 | -0.42122 |
| -0.33700 | -0.45254 |
| -0.34200 | -0.46729 |
| -0.34700 | -0.43938 |

### 8.3.21 CV-21

| Column 1 | Column 2 |
|---|---|
| -0.34800 | -0.41123 |
| -0.34300 | -0.30041 |
| -0.33800 | -0.24819 |
| -0.33300 | -0.26631 |
| -0.32800 | -0.30377 |
| -0.32300 | -0.31748 |
| -0.31800 | -0.31391 |
| -0.31300 | -0.30754 |
| -0.30800 | -0.32544 |
| -0.30300 | -0.35803 |
| -0.29800 | -0.37171 |
| -0.29300 | -0.35489 |
| -0.28800 | -0.33692 |
| -0.28300 | -0.33729 |
| -0.27800 | -0.34201 |
| -0.27300 | -0.33997 |
| -0.26800 | -0.33733 |
| -0.26300 | -0.33629 |
| -0.25800 | -0.33565 |
| -0.25300 | -0.33973 |
| -0.24800 | -0.34510 |
| -0.24300 | -0.34646 |
| -0.23800 | -0.34688 |

| Column 1 | Column 2 |
|---|---|
| -0.23300 | -0.35072 |
| -0.22800 | -0.35344 |
| -0.22300 | -0.34985 |
| -0.21800 | -0.34312 |
| -0.21300 | -0.34081 |
| -0.20800 | -0.34227 |
| -0.20300 | -0.34263 |
| -0.19800 | -0.33854 |
| -0.19300 | -0.32373 |
| -0.18800 | -0.30304 |
| -0.18300 | -0.28735 |
| -0.17800 | -0.27558 |
| -0.17300 | -0.25989 |
| -0.16800 | -0.24223 |
| -0.16300 | -0.22312 |
| -0.15800 | -0.19602 |
| -0.15300 | -0.16327 |
| -0.14800 | -0.13408 |
| -0.14300 | -0.11888 |
| -0.13800 | -0.11238 |
| -0.13300 | -0.099050 |
| -0.12800 | -0.078700 |
| -0.12300 | -0.058510 |
| -0.11800 | -0.036170 |
| -0.11300 | -0.0069800 |
| -0.10800 | 0.023020 |
| -0.10300 | 0.038770 |
| -0.098000 | 0.036970 |
| -0.093000 | 0.035250 |
| -0.088000 | 0.036230 |
| -0.083000 | 0.029240 |
| -0.078000 | 0.022150 |
| -0.073000 | 0.035020 |
| -0.068000 | 0.083050 |
| -0.063000 | 0.16589 |

| Column 1 | Column 2 |
|---|---|
| -0.058000 | 0.27054 |
| -0.053000 | 0.37502 |
| -0.048000 | 0.46542 |
| -0.043000 | 0.53886 |
| -0.038000 | 0.59138 |
| -0.033000 | 0.61886 |
| -0.028000 | 0.61484 |
| -0.023000 | 0.59004 |
| -0.018000 | 0.57297 |
| -0.013000 | 0.56888 |
| -0.0080000 | 0.55558 |
| -0.0030000 | 0.52347 |
| 0.0020000 | 0.47769 |
| 0.0070000 | 0.42593 |
| 0.012000 | 0.38724 |
| 0.017000 | 0.37142 |
| 0.022000 | 0.37021 |
| 0.027000 | 0.38135 |
| 0.032000 | 0.40508 |
| 0.037000 | 0.42629 |
| 0.042000 | 0.43240 |
| 0.047000 | 0.43049 |
| 0.052000 | 0.42060 |
| 0.057000 | 0.39631 |
| 0.062000 | 0.37241 |
| 0.067000 | 0.36453 |
| 0.072000 | 0.36372 |
| 0.077000 | 0.35828 |
| 0.082000 | 0.35101 |
| 0.087000 | 0.34069 |
| 0.092000 | 0.32775 |
| 0.097000 | 0.31866 |
| 0.10200 | 0.31238 |
| 0.10700 | 0.30526 |
| 0.11200 | 0.29541 |

| Column 1 | Column 2 |
|---|---|
| 0.11700 | 0.28718 |
| 0.12200 | 0.28202 |
| 0.12700 | 0.28070 |
| 0.13200 | 0.27939 |
| 0.13700 | 0.27525 |
| 0.14200 | 0.27459 |
| 0.14700 | 0.27116 |
| 0.15200 | 0.27388 |
| 0.15700 | 0.29683 |
| 0.16200 | 0.31900 |
| 0.16700 | 0.32659 |
| 0.17200 | 0.32504 |
| 0.17700 | 0.31922 |
| 0.18200 | 0.32290 |
| 0.18700 | 0.32380 |
| 0.19200 | 0.32243 |
| 0.19700 | 0.32272 |
| 0.19800 | 0.30336 |
| 0.19300 | 0.28586 |
| 0.18800 | 0.30141 |
| 0.18300 | 0.28866 |
| 0.17800 | 0.28133 |
| 0.17300 | 0.27359 |
| 0.16800 | 0.26432 |
| 0.16300 | 0.25857 |
| 0.15800 | 0.25766 |
| 0.15300 | 0.26477 |
| 0.14800 | 0.27528 |
| 0.14300 | 0.29103 |
| 0.13800 | 0.30344 |
| 0.13300 | 0.30819 |
| 0.12800 | 0.30673 |
| 0.12300 | 0.30518 |
| 0.11800 | 0.30603 |
| 0.11300 | 0.30630 |

| Column 1 | Column 2 |
|---|---|
| 0.10800 | 0.30570 |
| 0.10300 | 0.30570 |
| 0.098000 | 0.30721 |
| 0.093000 | 0.30803 |
| 0.088000 | 0.30889 |
| 0.083000 | 0.30854 |
| 0.078000 | 0.30769 |
| 0.073000 | 0.31080 |
| 0.068000 | 0.31690 |
| 0.063000 | 0.32308 |
| 0.058000 | 0.33281 |
| 0.053000 | 0.34156 |
| 0.048000 | 0.34210 |
| 0.043000 | 0.33619 |
| 0.038000 | 0.32537 |
| 0.033000 | 0.31385 |
| 0.028000 | 0.30576 |
| 0.023000 | 0.30748 |
| 0.018000 | 0.31269 |
| 0.013000 | 0.30925 |
| 0.0080000 | 0.30263 |
| 0.0030000 | 0.29404 |
| -0.0020000 | 0.28243 |
| -0.0070000 | 0.27144 |
| -0.012000 | 0.25956 |
| -0.017000 | 0.24721 |
| -0.022000 | 0.23597 |
| -0.027000 | 0.22606 |
| -0.032000 | 0.21657 |
| -0.037000 | 0.20344 |
| -0.042000 | 0.18631 |
| -0.047000 | 0.16834 |
| -0.052000 | 0.15053 |
| -0.057000 | 0.13250 |
| -0.062000 | 0.11390 |

| Column 1 | Column 2 |
|---|---|
| -0.067000 | 0.095030 |
| -0.072000 | 0.075780 |
| -0.077000 | 0.054610 |
| -0.082000 | 0.030990 |
| -0.087000 | 0.0055200 |
| -0.092000 | -0.023490 |
| -0.097000 | -0.056350 |
| -0.10200 | -0.090100 |
| -0.10700 | -0.12402 |
| -0.11200 | -0.15829 |
| -0.11700 | -0.19045 |
| -0.12200 | -0.22081 |
| -0.12700 | -0.25054 |
| -0.13200 | -0.28231 |
| -0.13700 | -0.31330 |
| -0.14200 | -0.34383 |
| -0.14700 | -0.40659 |
| -0.15200 | -0.49354 |
| -0.15700 | -0.55075 |
| -0.16200 | -0.57402 |
| -0.16700 | -0.57485 |
| -0.17200 | -0.56089 |
| -0.17700 | -0.55259 |
| -0.18200 | -0.55073 |
| -0.18700 | -0.53699 |
| -0.19200 | -0.51253 |
| -0.19700 | -0.49920 |
| -0.20200 | -0.49299 |
| -0.20700 | -0.48246 |
| -0.21200 | -0.47929 |
| -0.21700 | -0.48395 |
| -0.22200 | -0.48614 |
| -0.22700 | -0.48549 |
| -0.23200 | -0.48173 |
| -0.23700 | -0.47684 |

| Column 1 | Column 2 |
| --- | --- |
| -0.24200 | -0.47305 |
| -0.24700 | -0.47078 |
| -0.25200 | -0.47080 |
| -0.25700 | -0.47146 |
| -0.26200 | -0.47213 |
| -0.26700 | -0.47166 |
| -0.27200 | -0.47051 |
| -0.27700 | -0.46822 |
| -0.28200 | -0.46553 |
| -0.28700 | -0.46247 |
| -0.29200 | -0.45952 |
| -0.29700 | -0.45643 |
| -0.30200 | -0.45410 |
| -0.30700 | -0.45246 |
| -0.31200 | -0.45011 |
| -0.31700 | -0.45111 |
| -0.32200 | -0.45022 |
| -0.32700 | -0.45003 |
| -0.33200 | -0.44837 |
| -0.33700 | -0.44943 |
| -0.34200 | -0.45123 |
| -0.34700 | -0.45097 |

### 8.3.22 CV-24

| Column 1 | Column 2 |
| --- | --- |
| -0.34800 | -0.49796 |
| -0.34300 | -0.48804 |
| -0.33800 | -0.47528 |
| -0.33300 | -0.47388 |
| -0.32800 | -0.47038 |
| -0.32300 | -0.46696 |
| -0.31800 | -0.46427 |
| -0.31300 | -0.46094 |
| -0.30800 | -0.45906 |
| -0.30300 | -0.45701 |

| Column 1 | Column 2 |
|---|---|
| -0.29800 | -0.45586 |
| -0.29300 | -0.45309 |
| -0.28800 | -0.44991 |
| -0.28300 | -0.44694 |
| -0.27800 | -0.44439 |
| -0.27300 | -0.44135 |
| -0.26800 | -0.43803 |
| -0.26300 | -0.43537 |
| -0.25800 | -0.43235 |
| -0.25300 | -0.42825 |
| -0.24800 | -0.42362 |
| -0.24300 | -0.41999 |
| -0.23800 | -0.41664 |
| -0.23300 | -0.41279 |
| -0.22800 | -0.40753 |
| -0.22300 | -0.40239 |
| -0.21800 | -0.39753 |
| -0.21300 | -0.39070 |
| -0.20800 | -0.38230 |
| -0.20300 | -0.37386 |
| -0.19800 | -0.36527 |
| -0.19300 | -0.35496 |
| -0.18800 | -0.34355 |
| -0.18300 | -0.33121 |
| -0.17800 | -0.31714 |
| -0.17300 | -0.30132 |
| -0.16800 | -0.28278 |
| -0.16300 | -0.26152 |
| -0.15800 | -0.23899 |
| -0.15300 | -0.21522 |
| -0.14800 | -0.18960 |
| -0.14300 | -0.16166 |
| -0.13800 | -0.13195 |
| -0.13300 | -0.10030 |
| -0.12800 | -0.065780 |

| Column 1 | Column 2 |
|---|---|
| -0.12300 | -0.030590 |
| -0.11800 | 0.0047700 |
| -0.11300 | 0.041650 |
| -0.10800 | 0.079220 |
| -0.10300 | 0.11624 |
| -0.098000 | 0.15258 |
| -0.093000 | 0.18833 |
| -0.088000 | 0.22230 |
| -0.083000 | 0.25366 |
| -0.078000 | 0.28265 |
| -0.073000 | 0.30910 |
| -0.068000 | 0.33285 |
| -0.063000 | 0.35412 |
| -0.058000 | 0.37226 |
| -0.053000 | 0.38745 |
| -0.048000 | 0.40063 |
| -0.043000 | 0.41128 |
| -0.038000 | 0.41944 |
| -0.033000 | 0.42629 |
| -0.028000 | 0.43160 |
| -0.023000 | 0.43505 |
| -0.018000 | 0.43821 |
| -0.013000 | 0.44090 |
| -0.0080000 | 0.44215 |
| -0.0030000 | 0.44286 |
| 0.0020000 | 0.44299 |
| 0.0070000 | 0.44203 |
| 0.012000 | 0.44080 |
| 0.017000 | 0.43924 |
| 0.022000 | 0.43742 |
| 0.027000 | 0.43598 |
| 0.032000 | 0.43461 |
| 0.037000 | 0.43343 |
| 0.042000 | 0.43211 |
| 0.047000 | 0.43120 |

| Column 1 | Column 2 |
|---|---|
| 0.052000 | 0.43061 |
| 0.057000 | 0.42963 |
| 0.062000 | 0.42856 |
| 0.067000 | 0.42792 |
| 0.072000 | 0.42732 |
| 0.077000 | 0.42679 |
| 0.082000 | 0.42641 |
| 0.087000 | 0.42467 |
| 0.092000 | 0.42364 |
| 0.097000 | 0.41643 |
| 0.10200 | 0.40786 |
| 0.10700 | 0.41175 |
| 0.11200 | 0.41429 |
| 0.11700 | 0.41154 |
| 0.12200 | 0.41155 |
| 0.12700 | 0.41184 |
| 0.13200 | 0.41129 |
| 0.13700 | 0.41039 |
| 0.14200 | 0.40898 |
| 0.14700 | 0.40793 |
| 0.15200 | 0.40738 |
| 0.15700 | 0.40643 |
| 0.16200 | 0.40590 |
| 0.16700 | 0.40507 |
| 0.17200 | 0.40560 |
| 0.17700 | 0.40561 |
| 0.18200 | 0.40664 |
| 0.18700 | 0.40702 |
| 0.19200 | 0.40761 |
| 0.19700 | 0.40774 |
| 0.19800 | 0.38992 |
| 0.19300 | 0.38835 |
| 0.18800 | 0.38652 |
| 0.18300 | 0.38378 |
| 0.17800 | 0.38166 |

| Column 1 | Column 2 |
|---|---|
| 0.17300 | 0.38005 |
| 0.16800 | 0.37758 |
| 0.16300 | 0.37600 |
| 0.15800 | 0.37489 |
| 0.15300 | 0.37439 |
| 0.14800 | 0.37296 |
| 0.14300 | 0.37172 |
| 0.13800 | 0.37033 |
| 0.13300 | 0.36874 |
| 0.12800 | 0.36723 |
| 0.12300 | 0.36557 |
| 0.11800 | 0.36404 |
| 0.11300 | 0.36305 |
| 0.10800 | 0.36251 |
| 0.10300 | 0.36114 |
| 0.098000 | 0.35996 |
| 0.093000 | 0.35911 |
| 0.088000 | 0.35797 |
| 0.083000 | 0.35692 |
| 0.078000 | 0.35594 |
| 0.073000 | 0.35537 |
| 0.068000 | 0.35480 |
| 0.063000 | 0.35389 |
| 0.058000 | 0.35300 |
| 0.053000 | 0.35164 |
| 0.048000 | 0.34982 |
| 0.043000 | 0.34811 |
| 0.038000 | 0.34648 |
| 0.033000 | 0.34467 |
| 0.028000 | 0.34321 |
| 0.023000 | 0.34260 |
| 0.018000 | 0.34164 |
| 0.013000 | 0.33902 |
| 0.0080000 | 0.33505 |
| 0.0030000 | 0.32978 |

| Column 1 | Column 2 |
|---|---|
| -0.0020000 | 0.32180 |
| -0.0070000 | 0.31371 |
| -0.012000 | 0.30599 |
| -0.017000 | 0.29506 |
| -0.022000 | 0.28398 |
| -0.027000 | 0.27383 |
| -0.032000 | 0.26187 |
| -0.037000 | 0.24796 |
| -0.042000 | 0.23177 |
| -0.047000 | 0.21314 |
| -0.052000 | 0.19144 |
| -0.057000 | 0.16557 |
| -0.062000 | 0.13552 |
| -0.067000 | 0.10320 |
| -0.072000 | 0.069370 |
| -0.077000 | 0.033390 |
| -0.082000 | -0.0042000 |
| -0.087000 | -0.043370 |
| -0.092000 | -0.084130 |
| -0.097000 | -0.12591 |
| -0.10200 | -0.16789 |
| -0.10700 | -0.20950 |
| -0.11200 | -0.25079 |
| -0.11700 | -0.29087 |
| -0.12200 | -0.32907 |
| -0.12700 | -0.36534 |
| -0.13200 | -0.39911 |
| -0.13700 | -0.43049 |
| -0.14200 | -0.45905 |
| -0.14700 | -0.48413 |
| -0.15200 | -0.50621 |
| -0.15700 | -0.52575 |
| -0.16200 | -0.54250 |
| -0.16700 | -0.55576 |
| -0.17200 | -0.56686 |

| Column 1 | Column 2 |
|---|---|
| -0.17700 | -0.57610 |
| -0.18200 | -0.58290 |
| -0.18700 | -0.58814 |
| -0.19200 | -0.59152 |
| -0.19700 | -0.59363 |
| -0.20200 | -0.59516 |
| -0.20700 | -0.59557 |
| -0.21200 | -0.59522 |
| -0.21700 | -0.59418 |
| -0.22200 | -0.59230 |
| -0.22700 | -0.59053 |
| -0.23200 | -0.58830 |
| -0.23700 | -0.58578 |
| -0.24200 | -0.58320 |
| -0.24700 | -0.58001 |
| -0.25200 | -0.57725 |
| -0.25700 | -0.57448 |
| -0.26200 | -0.57123 |
| -0.26700 | -0.56820 |
| -0.27200 | -0.56580 |
| -0.27700 | -0.56337 |
| -0.28200 | -0.56074 |
| -0.28700 | -0.55816 |
| -0.29200 | -0.55569 |
| -0.29700 | -0.55312 |
| -0.30200 | -0.55123 |
| -0.30700 | -0.54935 |
| -0.31200 | -0.54611 |
| -0.31700 | -0.54411 |
| -0.32200 | -0.54156 |
| -0.32700 | -0.53949 |
| -0.33200 | -0.53744 |
| -0.33700 | -0.53558 |
| -0.34200 | -0.53434 |
| -0.34700 | -0.52927 |

### 8.3.23 CV-27

| Column 1 | Column 2 |
|---|---|
| -0.34800 | -0.61272 |
| -0.34300 | -0.59971 |
| -0.33800 | -0.58210 |
| -0.33300 | -0.58033 |
| -0.32800 | -0.57558 |
| -0.32300 | -0.57288 |
| -0.31800 | -0.57144 |
| -0.31300 | -0.56813 |
| -0.30800 | -0.56588 |
| -0.30300 | -0.56326 |
| -0.29800 | -0.56164 |
| -0.29300 | -0.55817 |
| -0.28800 | -0.55527 |
| -0.28300 | -0.55198 |
| -0.27800 | -0.54820 |
| -0.27300 | -0.54423 |
| -0.26800 | -0.53986 |
| -0.26300 | -0.53571 |
| -0.25800 | -0.53090 |
| -0.25300 | -0.52608 |
| -0.24800 | -0.52103 |
| -0.24300 | -0.51548 |
| -0.23800 | -0.50941 |
| -0.23300 | -0.50293 |
| -0.22800 | -0.49594 |
| -0.22300 | -0.48911 |
| -0.21800 | -0.48133 |
| -0.21300 | -0.47209 |
| -0.20800 | -0.46248 |
| -0.20300 | -0.45203 |
| -0.19800 | -0.43947 |
| -0.19300 | -0.42528 |
| -0.18800 | -0.41091 |
| -0.18300 | -0.39479 |

| Column 1 | Column 2 |
|---|---|
| -0.17800 | -0.37643 |
| -0.17300 | -0.35630 |
| -0.16800 | -0.33392 |
| -0.16300 | -0.30921 |
| -0.15800 | -0.28245 |
| -0.15300 | -0.25307 |
| -0.14800 | -0.22054 |
| -0.14300 | -0.18582 |
| -0.13800 | -0.14861 |
| -0.13300 | -0.10859 |
| -0.12800 | -0.066650 |
| -0.12300 | -0.023160 |
| -0.11800 | 0.022410 |
| -0.11300 | 0.069280 |
| -0.10800 | 0.11630 |
| -0.10300 | 0.16345 |
| -0.098000 | 0.21001 |
| -0.093000 | 0.25521 |
| -0.088000 | 0.29899 |
| -0.083000 | 0.34086 |
| -0.078000 | 0.37969 |
| -0.073000 | 0.41558 |
| -0.068000 | 0.44863 |
| -0.063000 | 0.47767 |
| -0.058000 | 0.50277 |
| -0.053000 | 0.52498 |
| -0.048000 | 0.54354 |
| -0.043000 | 0.55788 |
| -0.038000 | 0.56943 |
| -0.033000 | 0.57860 |
| -0.028000 | 0.58575 |
| -0.023000 | 0.59140 |
| -0.018000 | 0.59532 |
| -0.013000 | 0.59751 |
| -0.0080000 | 0.59888 |

| Column 1 | Column 2 |
|---|---|
| -0.0030000 | 0.60061 |
| 0.0020000 | 0.60127 |
| 0.0070000 | 0.60026 |
| 0.012000 | 0.59940 |
| 0.017000 | 0.59830 |
| 0.022000 | 0.59632 |
| 0.027000 | 0.59467 |
| 0.032000 | 0.59292 |
| 0.037000 | 0.59028 |
| 0.042000 | 0.58743 |
| 0.047000 | 0.58526 |
| 0.052000 | 0.58320 |
| 0.057000 | 0.58024 |
| 0.062000 | 0.57749 |
| 0.067000 | 0.57522 |
| 0.072000 | 0.57303 |
| 0.077000 | 0.57043 |
| 0.082000 | 0.56763 |
| 0.087000 | 0.56493 |
| 0.092000 | 0.56252 |
| 0.097000 | 0.56074 |
| 0.10200 | 0.55851 |
| 0.10700 | 0.55584 |
| 0.11200 | 0.55370 |
| 0.11700 | 0.55074 |
| 0.12200 | 0.54753 |
| 0.12700 | 0.54663 |
| 0.13200 | 0.54516 |
| 0.13700 | 0.54347 |
| 0.14200 | 0.54198 |
| 0.14700 | 0.54018 |
| 0.15200 | 0.53853 |
| 0.15700 | 0.53660 |
| 0.16200 | 0.53527 |
| 0.16700 | 0.53303 |

| Column 1 | Column 2 |
|---|---|
| 0.17200 | 0.53224 |
| 0.17700 | 0.53014 |
| 0.18200 | 0.52876 |
| 0.18700 | 0.52622 |
| 0.19200 | 0.52473 |
| 0.19700 | 0.52399 |
| 0.19800 | 0.50419 |
| 0.19300 | 0.50750 |
| 0.18800 | 0.50247 |
| 0.18300 | 0.49941 |
| 0.17800 | 0.49600 |
| 0.17300 | 0.49434 |
| 0.16800 | 0.49197 |
| 0.16300 | 0.49040 |
| 0.15800 | 0.48845 |
| 0.15300 | 0.48640 |
| 0.14800 | 0.48454 |
| 0.14300 | 0.48243 |
| 0.13800 | 0.47990 |
| 0.13300 | 0.47755 |
| 0.12800 | 0.47568 |
| 0.12300 | 0.47381 |
| 0.11800 | 0.47172 |
| 0.11300 | 0.47002 |
| 0.10800 | 0.46839 |
| 0.10300 | 0.46600 |
| 0.098000 | 0.46442 |
| 0.093000 | 0.46307 |
| 0.088000 | 0.46076 |
| 0.083000 | 0.45857 |
| 0.078000 | 0.45687 |
| 0.073000 | 0.45529 |
| 0.068000 | 0.45366 |
| 0.063000 | 0.45193 |
| 0.058000 | 0.44991 |

| Column 1 | Column 2 |
|---|---|
| 0.053000 | 0.44696 |
| 0.048000 | 0.44358 |
| 0.043000 | 0.44066 |
| 0.038000 | 0.43751 |
| 0.033000 | 0.43448 |
| 0.028000 | 0.43143 |
| 0.023000 | 0.42698 |
| 0.018000 | 0.42186 |
| 0.013000 | 0.41630 |
| 0.0080000 | 0.40914 |
| 0.0030000 | 0.40121 |
| -0.0020000 | 0.39258 |
| -0.0070000 | 0.38234 |
| -0.012000 | 0.37093 |
| -0.017000 | 0.35784 |
| -0.022000 | 0.34228 |
| -0.027000 | 0.32430 |
| -0.032000 | 0.30573 |
| -0.037000 | 0.28593 |
| -0.042000 | 0.26125 |
| -0.047000 | 0.23258 |
| -0.052000 | 0.20013 |
| -0.057000 | 0.16215 |
| -0.062000 | 0.12052 |
| -0.067000 | 0.077400 |
| -0.072000 | 0.032780 |
| -0.077000 | -0.014210 |
| -0.082000 | -0.064280 |
| -0.087000 | -0.11727 |
| -0.092000 | -0.17116 |
| -0.097000 | -0.22505 |
| -0.10200 | -0.27956 |
| -0.10700 | -0.33351 |
| -0.11200 | -0.38532 |
| -0.11700 | -0.43469 |

| Column 1 | Column 2 |
|---|---|
| -0.12200 | -0.48163 |
| -0.12700 | -0.52529 |
| -0.13200 | -0.56498 |
| -0.13700 | -0.60122 |
| -0.14200 | -0.63380 |
| -0.14700 | -0.66245 |
| -0.15200 | -0.68701 |
| -0.15700 | -0.70730 |
| -0.16200 | -0.72448 |
| -0.16700 | -0.73864 |
| -0.17200 | -0.75013 |
| -0.17700 | -0.75909 |
| -0.18200 | -0.76518 |
| -0.18700 | -0.76962 |
| -0.19200 | -0.77182 |
| -0.19700 | -0.77206 |
| -0.20200 | -0.77201 |
| -0.20700 | -0.77090 |
| -0.21200 | -0.76884 |
| -0.21700 | -0.76687 |
| -0.22200 | -0.76441 |
| -0.22700 | -0.76090 |
| -0.23200 | -0.75663 |
| -0.23700 | -0.75293 |
| -0.24200 | -0.74903 |
| -0.24700 | -0.74421 |
| -0.25200 | -0.74001 |
| -0.25700 | -0.73565 |
| -0.26200 | -0.73073 |
| -0.26700 | -0.72625 |
| -0.27200 | -0.72234 |
| -0.27700 | -0.71764 |
| -0.28200 | -0.71195 |
| -0.28700 | -0.70694 |
| -0.29200 | -0.70424 |

| Column 1 | Column 2 |
|---|---|
| -0.29700 | -0.70243 |
| -0.30200 | -0.69827 |
| -0.30700 | -0.69291 |
| -0.31200 | -0.68834 |
| -0.31700 | -0.68499 |
| -0.32200 | -0.68074 |
| -0.32700 | -0.67699 |
| -0.33200 | -0.67356 |
| -0.33700 | -0.67016 |
| -0.34200 | -0.66736 |
| -0.34700 | -0.66093 |

### 8.3.24 CV-28

| Column 1 | Column 2 |
|---|---|
| -0.34800 | -0.65716 |
| -0.34300 | -0.63965 |
| -0.33800 | -0.63312 |
| -0.33300 | -0.62728 |
| -0.32800 | -0.62180 |
| -0.32300 | -0.61705 |
| -0.31800 | -0.61241 |
| -0.31300 | -0.60748 |
| -0.30800 | -0.60210 |
| -0.30300 | -0.59735 |
| -0.29800 | -0.59214 |
| -0.29300 | -0.58643 |
| -0.28800 | -0.58132 |
| -0.28300 | -0.57690 |
| -0.27800 | -0.57255 |
| -0.27300 | -0.56814 |
| -0.26800 | -0.56359 |
| -0.26300 | -0.55843 |
| -0.25800 | -0.55332 |
| -0.25300 | -0.54805 |
| -0.24800 | -0.54225 |

| Column 1 | Column 2 |
|---|---|
| -0.24300 | -0.53667 |
| -0.23800 | -0.53182 |
| -0.23300 | -0.52633 |
| -0.22800 | -0.51972 |
| -0.22300 | -0.51288 |
| -0.21800 | -0.50379 |
| -0.21300 | -0.49241 |
| -0.20800 | -0.48115 |
| -0.20300 | -0.47116 |
| -0.19800 | -0.46203 |
| -0.19300 | -0.45033 |
| -0.18800 | -0.43523 |
| -0.18300 | -0.41808 |
| -0.17800 | -0.39775 |
| -0.17300 | -0.37590 |
| -0.16800 | -0.35351 |
| -0.16300 | -0.32764 |
| -0.15800 | -0.29774 |
| -0.15300 | -0.26444 |
| -0.14800 | -0.23078 |
| -0.14300 | -0.19950 |
| -0.13800 | -0.16339 |
| -0.13300 | -0.11810 |
| -0.12800 | -0.070220 |
| -0.12300 | -0.021440 |
| -0.11800 | 0.029280 |
| -0.11300 | 0.079620 |
| -0.10800 | 0.12941 |
| -0.10300 | 0.17736 |
| -0.098000 | 0.22853 |
| -0.093000 | 0.28510 |
| -0.088000 | 0.33518 |
| -0.083000 | 0.37553 |
| -0.078000 | 0.41290 |
| -0.073000 | 0.45353 |

| Column 1 | Column 2 |
|---|---|
| -0.068000 | 0.49403 |
| -0.063000 | 0.52846 |
| -0.058000 | 0.56080 |
| -0.053000 | 0.58719 |
| -0.048000 | 0.60408 |
| -0.043000 | 0.61848 |
| -0.038000 | 0.63442 |
| -0.033000 | 0.64938 |
| -0.028000 | 0.66005 |
| -0.023000 | 0.66799 |
| -0.018000 | 0.67324 |
| -0.013000 | 0.67517 |
| -0.0080000 | 0.67656 |
| -0.0030000 | 0.67853 |
| 0.0020000 | 0.67882 |
| 0.0070000 | 0.67829 |
| 0.012000 | 0.67985 |
| 0.017000 | 0.68109 |
| 0.022000 | 0.68104 |
| 0.027000 | 0.68055 |
| 0.032000 | 0.67852 |
| 0.037000 | 0.67563 |
| 0.042000 | 0.67297 |
| 0.047000 | 0.66974 |
| 0.052000 | 0.66526 |
| 0.057000 | 0.66047 |
| 0.062000 | 0.65657 |
| 0.067000 | 0.65365 |
| 0.072000 | 0.65106 |
| 0.077000 | 0.64907 |
| 0.082000 | 0.64613 |
| 0.087000 | 0.64245 |
| 0.092000 | 0.64104 |
| 0.097000 | 0.63980 |
| 0.10200 | 0.63582 |

| Column 1 | Column 2 |
|---|---|
| 0.10700 | 0.62989 |
| 0.11200 | 0.62352 |
| 0.11700 | 0.61884 |
| 0.12200 | 0.61717 |
| 0.12700 | 0.61450 |
| 0.13200 | 0.60907 |
| 0.13700 | 0.60505 |
| 0.14200 | 0.60419 |
| 0.14700 | 0.60503 |
| 0.15200 | 0.60525 |
| 0.15700 | 0.60412 |
| 0.16200 | 0.60192 |
| 0.16700 | 0.59971 |
| 0.17200 | 0.59801 |
| 0.17700 | 0.59699 |
| 0.18200 | 0.59570 |
| 0.18700 | 0.59315 |
| 0.19200 | 0.58916 |
| 0.19700 | 0.58261 |
| 0.19800 | 0.57266 |
| 0.19300 | 0.57143 |
| 0.18800 | 0.55574 |
| 0.18300 | 0.55601 |
| 0.17800 | 0.56085 |
| 0.17300 | 0.56295 |
| 0.16800 | 0.55338 |
| 0.16300 | 0.54132 |
| 0.15800 | 0.53533 |
| 0.15300 | 0.53175 |
| 0.14800 | 0.52940 |
| 0.14300 | 0.52777 |
| 0.13800 | 0.52711 |
| 0.13300 | 0.52643 |
| 0.12800 | 0.52555 |
| 0.12300 | 0.52295 |

| Column 1 | Column 2 |
|---|---|
| 0.11800 | 0.51808 |
| 0.11300 | 0.51212 |
| 0.10800 | 0.50603 |
| 0.10300 | 0.49982 |
| 0.098000 | 0.49365 |
| 0.093000 | 0.48944 |
| 0.088000 | 0.48637 |
| 0.083000 | 0.48189 |
| 0.078000 | 0.47707 |
| 0.073000 | 0.47326 |
| 0.068000 | 0.46921 |
| 0.063000 | 0.46540 |
| 0.058000 | 0.46323 |
| 0.053000 | 0.46181 |
| 0.048000 | 0.45839 |
| 0.043000 | 0.45511 |
| 0.038000 | 0.45388 |
| 0.033000 | 0.45134 |
| 0.028000 | 0.44856 |
| 0.023000 | 0.44765 |
| 0.018000 | 0.44660 |
| 0.013000 | 0.44266 |
| 0.0080000 | 0.43488 |
| 0.0030000 | 0.42356 |
| -0.0020000 | 0.40989 |
| -0.0070000 | 0.39558 |
| -0.012000 | 0.38101 |
| -0.017000 | 0.36534 |
| -0.022000 | 0.34799 |
| -0.027000 | 0.32999 |
| -0.032000 | 0.31138 |
| -0.037000 | 0.28892 |
| -0.042000 | 0.26082 |
| -0.047000 | 0.22957 |
| -0.052000 | 0.19480 |

| Column 1 | Column 2 |
|---|---|
| -0.057000 | 0.15410 |
| -0.062000 | 0.10782 |
| -0.067000 | 0.057880 |
| -0.072000 | 0.0044900 |
| -0.077000 | -0.051440 |
| -0.082000 | -0.10776 |
| -0.087000 | -0.16366 |
| -0.092000 | -0.21891 |
| -0.097000 | -0.27537 |
| -0.10200 | -0.32352 |
| -0.10700 | -0.36698 |
| -0.11200 | -0.42810 |
| -0.11700 | -0.49338 |
| -0.12200 | -0.54522 |
| -0.12700 | -0.59182 |
| -0.13200 | -0.63693 |
| -0.13700 | -0.67929 |
| -0.14200 | -0.71524 |
| -0.14700 | -0.74453 |
| -0.15200 | -0.77110 |
| -0.15700 | -0.79474 |
| -0.16200 | -0.81377 |
| -0.16700 | -0.82841 |
| -0.17200 | -0.84017 |
| -0.17700 | -0.84927 |
| -0.18200 | -0.85497 |
| -0.18700 | -0.85768 |
| -0.19200 | -0.85883 |
| -0.19700 | -0.85917 |
| -0.20200 | -0.85831 |
| -0.20700 | -0.85564 |
| -0.21200 | -0.85188 |
| -0.21700 | -0.84835 |
| -0.22200 | -0.84424 |
| -0.22700 | -0.83940 |

| Column 1 | Column 2 |
|---|---|
| -0.23200 | -0.83489 |
| -0.23700 | -0.83073 |
| -0.24200 | -0.82659 |
| -0.24700 | -0.82185 |
| -0.25200 | -0.81656 |
| -0.25700 | -0.81050 |
| -0.26200 | -0.80463 |
| -0.26700 | -0.79963 |
| -0.27200 | -0.79411 |
| -0.27700 | -0.78832 |
| -0.28200 | -0.78228 |
| -0.28700 | -0.77708 |
| -0.29200 | -0.77309 |
| -0.29700 | -0.76996 |
| -0.30200 | -0.76703 |
| -0.30700 | -0.76308 |
| -0.31200 | -0.75844 |
| -0.31700 | -0.75405 |
| -0.32200 | -0.75054 |
| -0.32700 | -0.74679 |
| -0.33200 | -0.74332 |
| -0.33700 | -0.73822 |
| -0.34200 | -0.73396 |
| -0.34700 | -0.72837 |

### 8.3.25 CV-30

Global Evaluation 1

| Column 1 | Column 2 |
|---|---|
| -0.34800 | -0.78331 |
| -0.34300 | -0.76491 |
| -0.33800 | -0.76125 |
| -0.33300 | -0.75329 |
| -0.32800 | -0.74672 |
| -0.32300 | -0.73982 |
| -0.31800 | -0.73272 |

| Column 1 | Column 2 |
|---|---|
| -0.31300 | -0.72587 |
| -0.30800 | -0.71874 |
| -0.30300 | -0.71284 |
| -0.29800 | -0.70592 |
| -0.29300 | -0.69919 |
| -0.28800 | -0.69253 |
| -0.28300 | -0.68592 |
| -0.27800 | -0.68039 |
| -0.27300 | -0.67442 |
| -0.26800 | -0.66769 |
| -0.26300 | -0.66111 |
| -0.25800 | -0.65487 |
| -0.25300 | -0.64832 |
| -0.24800 | -0.64093 |
| -0.24300 | -0.63326 |
| -0.23800 | -0.62696 |
| -0.23300 | -0.61969 |
| -0.22800 | -0.61130 |
| -0.22300 | -0.60325 |
| -0.21800 | -0.59634 |
| -0.21300 | -0.59869 |
| -0.20800 | -0.59356 |
| -0.20300 | -0.56506 |
| -0.19800 | -0.54045 |
| -0.19300 | -0.52654 |
| -0.18800 | -0.50849 |
| -0.18300 | -0.48692 |
| -0.17800 | -0.46261 |
| -0.17300 | -0.43679 |
| -0.16800 | -0.40925 |
| -0.16300 | -0.37927 |
| -0.15800 | -0.34709 |
| -0.15300 | -0.31114 |
| -0.14800 | -0.27128 |
| -0.14300 | -0.22857 |

| Column 1 | Column 2 |
|---|---|
| -0.13800 | -0.18327 |
| -0.13300 | -0.13465 |
| -0.12800 | -0.083100 |
| -0.12300 | -0.028570 |
| -0.11800 | 0.028470 |
| -0.11300 | 0.087270 |
| -0.10800 | 0.14804 |
| -0.10300 | 0.21029 |
| -0.098000 | 0.27265 |
| -0.093000 | 0.33365 |
| -0.088000 | 0.39325 |
| -0.083000 | 0.45132 |
| -0.078000 | 0.50613 |
| -0.073000 | 0.55662 |
| -0.068000 | 0.60316 |
| -0.063000 | 0.64508 |
| -0.058000 | 0.68228 |
| -0.053000 | 0.71487 |
| -0.048000 | 0.74203 |
| -0.043000 | 0.76459 |
| -0.038000 | 0.78358 |
| -0.033000 | 0.79908 |
| -0.028000 | 0.81059 |
| -0.023000 | 0.81867 |
| -0.018000 | 0.82444 |
| -0.013000 | 0.82827 |
| -0.0080000 | 0.83046 |
| -0.0030000 | 0.83125 |
| 0.0020000 | 0.83098 |
| 0.0070000 | 0.82988 |
| 0.012000 | 0.82816 |
| 0.017000 | 0.82577 |
| 0.022000 | 0.82320 |
| 0.027000 | 0.82087 |
| 0.032000 | 0.81814 |

| Column 1 | Column 2 |
|---|---|
| 0.037000 | 0.81458 |
| 0.042000 | 0.81048 |
| 0.047000 | 0.80609 |
| 0.052000 | 0.80146 |
| 0.057000 | 0.79729 |
| 0.062000 | 0.79370 |
| 0.067000 | 0.78944 |
| 0.072000 | 0.78421 |
| 0.077000 | 0.77935 |
| 0.082000 | 0.77488 |
| 0.087000 | 0.77039 |
| 0.092000 | 0.76629 |
| 0.097000 | 0.76248 |
| 0.10200 | 0.75846 |
| 0.10700 | 0.75459 |
| 0.11200 | 0.75094 |
| 0.11700 | 0.74665 |
| 0.12200 | 0.74271 |
| 0.12700 | 0.73873 |
| 0.13200 | 0.73373 |
| 0.13700 | 0.72868 |
| 0.14200 | 0.72399 |
| 0.14700 | 0.71916 |
| 0.15200 | 0.71475 |
| 0.15700 | 0.71006 |
| 0.16200 | 0.70482 |
| 0.16700 | 0.70039 |
| 0.17200 | 0.69602 |
| 0.17700 | 0.69385 |
| 0.18200 | 0.69189 |
| 0.18700 | 0.69060 |
| 0.19200 | 0.68923 |
| 0.19700 | 0.68381 |
| 0.19800 | 0.67395 |
| 0.19300 | 0.67322 |

| Column 1 | Column 2 |
|---|---|
| 0.18800 | 0.65585 |
| 0.18300 | 0.65390 |
| 0.17800 | 0.64860 |
| 0.17300 | 0.64493 |
| 0.16800 | 0.64126 |
| 0.16300 | 0.63813 |
| 0.15800 | 0.63702 |
| 0.15300 | 0.63503 |
| 0.14800 | 0.63302 |
| 0.14300 | 0.63025 |
| 0.13800 | 0.62749 |
| 0.13300 | 0.62476 |
| 0.12800 | 0.62317 |
| 0.12300 | 0.62057 |
| 0.11800 | 0.61695 |
| 0.11300 | 0.61416 |
| 0.10800 | 0.61116 |
| 0.10300 | 0.60783 |
| 0.098000 | 0.60467 |
| 0.093000 | 0.60184 |
| 0.088000 | 0.59857 |
| 0.083000 | 0.59559 |
| 0.078000 | 0.59281 |
| 0.073000 | 0.58948 |
| 0.068000 | 0.58596 |
| 0.063000 | 0.58270 |
| 0.058000 | 0.57903 |
| 0.053000 | 0.57508 |
| 0.048000 | 0.57135 |
| 0.043000 | 0.56652 |
| 0.038000 | 0.56128 |
| 0.033000 | 0.55620 |
| 0.028000 | 0.55002 |
| 0.023000 | 0.54248 |
| 0.018000 | 0.53474 |

| Column 1 | Column 2 |
|---|---|
| 0.013000 | 0.52686 |
| 0.0080000 | 0.51737 |
| 0.0030000 | 0.50622 |
| -0.0020000 | 0.49363 |
| -0.0070000 | 0.47883 |
| -0.012000 | 0.46168 |
| -0.017000 | 0.44150 |
| -0.022000 | 0.41716 |
| -0.027000 | 0.38969 |
| -0.032000 | 0.35976 |
| -0.037000 | 0.32605 |
| -0.042000 | 0.28760 |
| -0.047000 | 0.24418 |
| -0.052000 | 0.19581 |
| -0.057000 | 0.14313 |
| -0.062000 | 0.086280 |
| -0.067000 | 0.024580 |
| -0.072000 | -0.041130 |
| -0.077000 | -0.10952 |
| -0.082000 | -0.18041 |
| -0.087000 | -0.25330 |
| -0.092000 | -0.32693 |
| -0.097000 | -0.40002 |
| -0.10200 | -0.47232 |
| -0.10700 | -0.54301 |
| -0.11200 | -0.61023 |
| -0.11700 | -0.67394 |
| -0.12200 | -0.73403 |
| -0.12700 | -0.78868 |
| -0.13200 | -0.83816 |
| -0.13700 | -0.88333 |
| -0.14200 | -0.92299 |
| -0.14700 | -0.95673 |
| -0.15200 | -0.98514 |
| -0.15700 | -1.0089 |

| Column 1 | Column 2 |
|---|---|
| -0.16200 | -1.0281 |
| -0.16700 | -1.0427 |
| -0.17200 | -1.0534 |
| -0.17700 | -1.0608 |
| -0.18200 | -1.0661 |
| -0.18700 | -1.0694 |
| -0.19200 | -1.0695 |
| -0.19700 | -1.0677 |
| -0.20200 | -1.0651 |
| -0.20700 | -1.0609 |
| -0.21200 | -1.0552 |
| -0.21700 | -1.0493 |
| -0.22200 | -1.0432 |
| -0.22700 | -1.0363 |
| -0.23200 | -1.0299 |
| -0.23700 | -1.0236 |
| -0.24200 | -1.0161 |
| -0.24700 | -1.0080 |
| -0.25200 | -1.0006 |
| -0.25700 | -0.99370 |
| -0.26200 | -0.98616 |
| -0.26700 | -0.97863 |
| -0.27200 | -0.97138 |
| -0.27700 | -0.96436 |
| -0.28200 | -0.95739 |
| -0.28700 | -0.95049 |
| -0.29200 | -0.94315 |
| -0.29700 | -0.93613 |
| -0.30200 | -0.92938 |
| -0.30700 | -0.92238 |
| -0.31200 | -0.91588 |
| -0.31700 | -0.90933 |
| -0.32200 | -0.90290 |
| -0.32700 | -0.89612 |
| -0.33200 | -0.89053 |

| Column 1 | Column 2 |
|---|---|
| -0.33700 | -0.88373 |
| -0.34200 | -0.87771 |
| -0.34700 | -0.87089 |

### 8.3.26 CV-36

| Column 1 | Column 2 |
|---|---|
| -0.34800 | -1.3850 |
| -0.34300 | -1.3289 |
| -0.33800 | -1.3192 |
| -0.33300 | -1.2916 |
| -0.32800 | -1.2695 |
| -0.32300 | -1.2474 |
| -0.31800 | -1.2235 |
| -0.31300 | -1.2018 |
| -0.30800 | -1.1809 |
| -0.30300 | -1.1634 |
| -0.29800 | -1.1453 |
| -0.29300 | -1.1265 |
| -0.28800 | -1.1074 |
| -0.28300 | -1.0900 |
| -0.27800 | -1.0717 |
| -0.27300 | -1.0527 |
| -0.26800 | -1.0336 |
| -0.26300 | -1.0147 |
| -0.25800 | -0.99674 |
| -0.25300 | -0.97832 |
| -0.24800 | -0.95986 |
| -0.24300 | -0.94116 |
| -0.23800 | -0.92220 |
| -0.23300 | -0.90184 |
| -0.22800 | -0.88173 |
| -0.22300 | -0.86265 |
| -0.21800 | -0.84123 |
| -0.21300 | -0.81805 |
| -0.20800 | -0.79525 |

| Column 1 | Column 2 |
|---|---|
| -0.20300 | -0.77034 |
| -0.19800 | -0.74219 |
| -0.19300 | -0.71187 |
| -0.18800 | -0.67848 |
| -0.18300 | -0.64193 |
| -0.17800 | -0.60155 |
| -0.17300 | -0.55725 |
| -0.16800 | -0.50908 |
| -0.16300 | -0.45659 |
| -0.15800 | -0.39897 |
| -0.15300 | -0.33487 |
| -0.14800 | -0.26486 |
| -0.14300 | -0.18946 |
| -0.13800 | -0.10775 |
| -0.13300 | -0.019280 |
| -0.12800 | 0.075560 |
| -0.12300 | 0.17695 |
| -0.11800 | 0.28449 |
| -0.11300 | 0.39695 |
| -0.10800 | 0.51358 |
| -0.10300 | 0.63509 |
| -0.098000 | 0.75989 |
| -0.093000 | 0.88644 |
| -0.088000 | 1.0150 |
| -0.083000 | 1.1426 |
| -0.078000 | 1.2680 |
| -0.073000 | 1.3907 |
| -0.068000 | 1.5068 |
| -0.063000 | 1.6154 |
| -0.058000 | 1.7168 |
| -0.053000 | 1.8091 |
| -0.048000 | 1.8901 |
| -0.043000 | 1.9594 |
| -0.038000 | 2.0175 |
| -0.033000 | 2.0647 |

| Column 1 | Column 2 |
|---|---|
| -0.028000 | 2.1013 |
| -0.023000 | 2.1287 |
| -0.018000 | 2.1476 |
| -0.013000 | 2.1585 |
| -0.0080000 | 2.1627 |
| -0.0030000 | 2.1615 |
| 0.0020000 | 2.1554 |
| 0.0070000 | 2.1451 |
| 0.012000 | 2.1323 |
| 0.017000 | 2.1171 |
| 0.022000 | 2.0998 |
| 0.027000 | 2.0817 |
| 0.032000 | 2.0623 |
| 0.037000 | 2.0415 |
| 0.042000 | 2.0202 |
| 0.047000 | 1.9982 |
| 0.052000 | 1.9757 |
| 0.057000 | 1.9533 |
| 0.062000 | 1.9307 |
| 0.067000 | 1.9081 |
| 0.072000 | 1.8855 |
| 0.077000 | 1.8633 |
| 0.082000 | 1.8412 |
| 0.087000 | 1.8196 |
| 0.092000 | 1.7980 |
| 0.097000 | 1.7759 |
| 0.10200 | 1.7549 |
| 0.10700 | 1.7349 |
| 0.11200 | 1.7148 |
| 0.11700 | 1.6941 |
| 0.12200 | 1.6743 |
| 0.12700 | 1.6542 |
| 0.13200 | 1.6344 |
| 0.13700 | 1.6146 |
| 0.14200 | 1.5950 |

| Column 1 | Column 2 |
|---|---|
| 0.14700 | 1.5758 |
| 0.15200 | 1.5564 |
| 0.15700 | 1.5378 |
| 0.16200 | 1.5183 |
| 0.16700 | 1.5006 |
| 0.17200 | 1.4817 |
| 0.17700 | 1.4641 |
| 0.18200 | 1.4456 |
| 0.18700 | 1.4290 |
| 0.19200 | 1.4119 |
| 0.19700 | 1.3922 |
| 0.19800 | 1.3786 |
| 0.19300 | 1.3793 |
| 0.18800 | 1.3194 |
| 0.18300 | 1.3110 |
| 0.17800 | 1.2910 |
| 0.17300 | 1.2735 |
| 0.16800 | 1.2581 |
| 0.16300 | 1.2405 |
| 0.15800 | 1.2254 |
| 0.15300 | 1.2082 |
| 0.14800 | 1.1937 |
| 0.14300 | 1.1771 |
| 0.13800 | 1.1612 |
| 0.13300 | 1.1449 |
| 0.12800 | 1.1298 |
| 0.12300 | 1.1139 |
| 0.11800 | 1.0985 |
| 0.11300 | 1.0841 |
| 0.10800 | 1.0686 |
| 0.10300 | 1.0538 |
| 0.098000 | 1.0392 |
| 0.093000 | 1.0246 |
| 0.088000 | 1.0091 |
| 0.083000 | 0.99409 |

| Column 1 | Column 2 |
|---|---|
| 0.078000 | 0.97918 |
| 0.073000 | 0.96344 |
| 0.068000 | 0.94738 |
| 0.063000 | 0.93124 |
| 0.058000 | 0.91468 |
| 0.053000 | 0.89761 |
| 0.048000 | 0.87973 |
| 0.043000 | 0.86043 |
| 0.038000 | 0.84024 |
| 0.033000 | 0.81834 |
| 0.028000 | 0.79474 |
| 0.023000 | 0.76875 |
| 0.018000 | 0.74051 |
| 0.013000 | 0.70907 |
| 0.0080000 | 0.67355 |
| 0.0030000 | 0.63356 |
| -0.0020000 | 0.58840 |
| -0.0070000 | 0.53791 |
| -0.012000 | 0.48000 |
| -0.017000 | 0.41462 |
| -0.022000 | 0.34091 |
| -0.027000 | 0.25714 |
| -0.032000 | 0.16288 |
| -0.037000 | 0.058320 |
| -0.042000 | -0.056130 |
| -0.047000 | -0.18121 |
| -0.052000 | -0.31734 |
| -0.057000 | -0.46251 |
| -0.062000 | -0.61496 |
| -0.067000 | -0.77447 |
| -0.072000 | -0.93974 |
| -0.077000 | -1.1079 |
| -0.082000 | -1.2761 |
| -0.087000 | -1.4432 |
| -0.092000 | -1.6066 |

| Column 1 | Column 2 |
|---|---|
| -0.097000 | -1.7641 |
| -0.10200 | -1.9139 |
| -0.10700 | -2.0533 |
| -0.11200 | -2.1817 |
| -0.11700 | -2.2981 |
| -0.12200 | -2.4014 |
| -0.12700 | -2.4905 |
| -0.13200 | -2.5667 |
| -0.13700 | -2.6301 |
| -0.14200 | -2.6794 |
| -0.14700 | -2.7165 |
| -0.15200 | -2.7421 |
| -0.15700 | -2.7564 |
| -0.16200 | -2.7604 |
| -0.16700 | -2.7560 |
| -0.17200 | -2.7447 |
| -0.17700 | -2.7263 |
| -0.18200 | -2.7019 |
| -0.18700 | -2.6739 |
| -0.19200 | -2.6425 |
| -0.19700 | -2.6080 |
| -0.20200 | -2.5712 |
| -0.20700 | -2.5317 |
| -0.21200 | -2.4912 |
| -0.21700 | -2.4505 |
| -0.22200 | -2.4098 |
| -0.22700 | -2.3683 |
| -0.23200 | -2.3269 |
| -0.23700 | -2.2859 |
| -0.24200 | -2.2448 |
| -0.24700 | -2.2039 |
| -0.25200 | -2.1640 |
| -0.25700 | -2.1248 |
| -0.26200 | -2.0855 |
| -0.26700 | -2.0470 |

| Column 1 | Column 2 |
|---|---|
| -0.27200 | -2.0083 |
| -0.27700 | -1.9717 |
| -0.28200 | -1.9354 |
| -0.28700 | -1.8993 |
| -0.29200 | -1.8639 |
| -0.29700 | -1.8293 |
| -0.30200 | -1.7952 |
| -0.30700 | -1.7613 |
| -0.31200 | -1.7296 |
| -0.31700 | -1.6967 |
| -0.32200 | -1.6661 |
| -0.32700 | -1.6339 |
| -0.33200 | -1.6055 |
| -0.33700 | -1.5751 |
| -0.34200 | -1.5466 |
| -0.34700 | -1.5189 |

### 8.3.27 CV-38

| Column 1 | Column 2 |
|---|---|
| -0.34800 | -1.5214 |
| -0.34300 | -1.4579 |
| -0.33800 | -1.4386 |
| -0.33300 | -1.3990 |
| -0.32800 | -1.3646 |
| -0.32300 | -1.3323 |
| -0.31800 | -1.2987 |
| -0.31300 | -1.2681 |
| -0.30800 | -1.2360 |
| -0.30300 | -1.2066 |
| -0.29800 | -1.1765 |
| -0.29300 | -1.1483 |
| -0.28800 | -1.1201 |
| -0.28300 | -1.0921 |
| -0.27800 | -1.0646 |
| -0.27300 | -1.0379 |

| Column 1 | Column 2 |
|---|---|
| -0.26800 | -1.0121 |
| -0.26300 | -0.98582 |
| -0.25800 | -0.95971 |
| -0.25300 | -0.93345 |
| -0.24800 | -0.90831 |
| -0.24300 | -0.88295 |
| -0.23800 | -0.85716 |
| -0.23300 | -0.83055 |
| -0.22800 | -0.80404 |
| -0.22300 | -0.77704 |
| -0.21800 | -0.74850 |
| -0.21300 | -0.71909 |
| -0.20800 | -0.68808 |
| -0.20300 | -0.65513 |
| -0.19800 | -0.62016 |
| -0.19300 | -0.58282 |
| -0.18800 | -0.54138 |
| -0.18300 | -0.49637 |
| -0.17800 | -0.44779 |
| -0.17300 | -0.39376 |
| -0.16800 | -0.33432 |
| -0.16300 | -0.26940 |
| -0.15800 | -0.19765 |
| -0.15300 | -0.11934 |
| -0.14800 | -0.034250 |
| -0.14300 | 0.059260 |
| -0.13800 | 0.16131 |
| -0.13300 | 0.27200 |
| -0.12800 | 0.39088 |
| -0.12300 | 0.51823 |
| -0.11800 | 0.65421 |
| -0.11300 | 0.79816 |
| -0.10800 | 0.95015 |
| -0.10300 | 1.1096 |
| -0.098000 | 1.2754 |

| Column 1 | Column 2 |
|---|---|
| -0.093000 | 1.4470 |
| -0.088000 | 1.6225 |
| -0.083000 | 1.7987 |
| -0.078000 | 1.9757 |
| -0.073000 | 2.1518 |
| -0.068000 | 2.3225 |
| -0.063000 | 2.4851 |
| -0.058000 | 2.6379 |
| -0.053000 | 2.7782 |
| -0.048000 | 2.9033 |
| -0.043000 | 3.0127 |
| -0.038000 | 3.1041 |
| -0.033000 | 3.1770 |
| -0.028000 | 3.2328 |
| -0.023000 | 3.2722 |
| -0.018000 | 3.2959 |
| -0.013000 | 3.3063 |
| -0.0080000 | 3.3048 |
| -0.0030000 | 3.2926 |
| 0.0020000 | 3.2726 |
| 0.0070000 | 3.2453 |
| 0.012000 | 3.2117 |
| 0.017000 | 3.1738 |
| 0.022000 | 3.1329 |
| 0.027000 | 3.0889 |
| 0.032000 | 3.0426 |
| 0.037000 | 2.9946 |
| 0.042000 | 2.9450 |
| 0.047000 | 2.8958 |
| 0.052000 | 2.8461 |
| 0.057000 | 2.7961 |
| 0.062000 | 2.7462 |
| 0.067000 | 2.6964 |
| 0.072000 | 2.6474 |
| 0.077000 | 2.5988 |

| Column 1 | Column 2 |
|---|---|
| 0.082000 | 2.5494 |
| 0.087000 | 2.5014 |
| 0.092000 | 2.4541 |
| 0.097000 | 2.4069 |
| 0.10200 | 2.3610 |
| 0.10700 | 2.3154 |
| 0.11200 | 2.2708 |
| 0.11700 | 2.2269 |
| 0.12200 | 2.1844 |
| 0.12700 | 2.1416 |
| 0.13200 | 2.0994 |
| 0.13700 | 2.0585 |
| 0.14200 | 2.0183 |
| 0.14700 | 1.9788 |
| 0.15200 | 1.9395 |
| 0.15700 | 1.9018 |
| 0.16200 | 1.8638 |
| 0.16700 | 1.8281 |
| 0.17200 | 1.7911 |
| 0.17700 | 1.7570 |
| 0.18200 | 1.7213 |
| 0.18700 | 1.6882 |
| 0.19200 | 1.6560 |
| 0.19700 | 1.6180 |
| 0.19800 | 1.6028 |
| 0.19300 | 1.5757 |
| 0.18800 | 1.5016 |
| 0.18300 | 1.4776 |
| 0.17800 | 1.4433 |
| 0.17300 | 1.4129 |
| 0.16800 | 1.3836 |
| 0.16300 | 1.3525 |
| 0.15800 | 1.3245 |
| 0.15300 | 1.2948 |
| 0.14800 | 1.2680 |

| Column 1 | Column 2 |
|---|---|
| 0.14300 | 1.2394 |
| 0.13800 | 1.2125 |
| 0.13300 | 1.1861 |
| 0.12800 | 1.1603 |
| 0.12300 | 1.1342 |
| 0.11800 | 1.1090 |
| 0.11300 | 1.0849 |
| 0.10800 | 1.0600 |
| 0.10300 | 1.0356 |
| 0.098000 | 1.0113 |
| 0.093000 | 0.98729 |
| 0.088000 | 0.96349 |
| 0.083000 | 0.93985 |
| 0.078000 | 0.91531 |
| 0.073000 | 0.89148 |
| 0.068000 | 0.86759 |
| 0.063000 | 0.84231 |
| 0.058000 | 0.81630 |
| 0.053000 | 0.78975 |
| 0.048000 | 0.76206 |
| 0.043000 | 0.73206 |
| 0.038000 | 0.70085 |
| 0.033000 | 0.66723 |
| 0.028000 | 0.63009 |
| 0.023000 | 0.58943 |
| 0.018000 | 0.54482 |
| 0.013000 | 0.49441 |
| 0.0080000 | 0.43762 |
| 0.0030000 | 0.37411 |
| -0.0020000 | 0.30151 |
| -0.0070000 | 0.21901 |
| -0.012000 | 0.12625 |
| -0.017000 | 0.021390 |
| -0.022000 | -0.097400 |
| -0.027000 | -0.23011 |

| Column 1 | Column 2 |
|---|---|
| -0.032000 | -0.37807 |
| -0.037000 | -0.54189 |
| -0.042000 | -0.72107 |
| -0.047000 | -0.91521 |
| -0.052000 | -1.1230 |
| -0.057000 | -1.3431 |
| -0.062000 | -1.5729 |
| -0.067000 | -1.8092 |
| -0.072000 | -2.0495 |
| -0.077000 | -2.2899 |
| -0.082000 | -2.5269 |
| -0.087000 | -2.7576 |
| -0.092000 | -2.9775 |
| -0.097000 | -3.1846 |
| -0.10200 | -3.3762 |
| -0.10700 | -3.5485 |
| -0.11200 | -3.7018 |
| -0.11700 | -3.8348 |
| -0.12200 | -3.9453 |
| -0.12700 | -4.0335 |
| -0.13200 | -4.1012 |
| -0.13700 | -4.1482 |
| -0.14200 | -4.1739 |
| -0.14700 | -4.1817 |
| -0.15200 | -4.1721 |
| -0.15700 | -4.1467 |
| -0.16200 | -4.1083 |
| -0.16700 | -4.0577 |
| -0.17200 | -3.9970 |
| -0.17700 | -3.9285 |
| -0.18200 | -3.8529 |
| -0.18700 | -3.7718 |
| -0.19200 | -3.6865 |
| -0.19700 | -3.5988 |
| -0.20200 | -3.5096 |

| Column 1 | Column 2 |
|---|---|
| -0.20700 | -3.4189 |
| -0.21200 | -3.3281 |
| -0.21700 | -3.2378 |
| -0.22200 | -3.1495 |
| -0.22700 | -3.0612 |
| -0.23200 | -2.9730 |
| -0.23700 | -2.8872 |
| -0.24200 | -2.8037 |
| -0.24700 | -2.7223 |
| -0.25200 | -2.6428 |
| -0.25700 | -2.5647 |
| -0.26200 | -2.4887 |
| -0.26700 | -2.4154 |
| -0.27200 | -2.3432 |
| -0.27700 | -2.2745 |
| -0.28200 | -2.2076 |
| -0.28700 | -2.1419 |
| -0.29200 | -2.0785 |
| -0.29700 | -2.0176 |
| -0.30200 | -1.9580 |
| -0.30700 | -1.8996 |
| -0.31200 | -1.8443 |
| -0.31700 | -1.7893 |
| -0.32200 | -1.7373 |
| -0.32700 | -1.6855 |
| -0.33200 | -1.6381 |
| -0.33700 | -1.5891 |
| -0.34200 | -1.5441 |
| -0.34700 | -1.4992 |

### 8.3.28 CV-39

| Column 1 | Column 2 |
|---|---|
| -0.34800 | -1.4523 |
| -0.34300 | -1.3899 |
| -0.33800 | -1.3538 |

| Column 1 | Column 2 |
|---|---|
| -0.33300 | -1.3102 |
| -0.32800 | -1.2696 |
| -0.32300 | -1.2318 |
| -0.31800 | -1.1931 |
| -0.31300 | -1.1576 |
| -0.30800 | -1.1215 |
| -0.30300 | -1.0877 |
| -0.29800 | -1.0541 |
| -0.29300 | -1.0221 |
| -0.28800 | -0.99116 |
| -0.28300 | -0.96064 |
| -0.27800 | -0.93055 |
| -0.27300 | -0.90125 |
| -0.26800 | -0.87235 |
| -0.26300 | -0.84398 |
| -0.25800 | -0.81668 |
| -0.25300 | -0.78889 |
| -0.24800 | -0.76171 |
| -0.24300 | -0.73473 |
| -0.23800 | -0.70788 |
| -0.23300 | -0.68032 |
| -0.22800 | -0.65214 |
| -0.22300 | -0.62363 |
| -0.21800 | -0.59415 |
| -0.21300 | -0.56385 |
| -0.20800 | -0.53150 |
| -0.20300 | -0.49640 |
| -0.19800 | -0.45910 |
| -0.19300 | -0.41923 |
| -0.18800 | -0.37514 |
| -0.18300 | -0.32663 |
| -0.17800 | -0.27349 |
| -0.17300 | -0.21513 |
| -0.16800 | -0.15080 |
| -0.16300 | -0.079460 |

| Column 1 | Column 2 |
|---|---|
| -0.15800 | -6.0083E-4 |
| -0.15300 | 0.086340 |
| -0.14800 | 0.18153 |
| -0.14300 | 0.28562 |
| -0.13800 | 0.39991 |
| -0.13300 | 0.52418 |
| -0.12800 | 0.65792 |
| -0.12300 | 0.80273 |
| -0.11800 | 0.95852 |
| -0.11300 | 1.1246 |
| -0.10800 | 1.3011 |
| -0.10300 | 1.4875 |
| -0.098000 | 1.6824 |
| -0.093000 | 1.8851 |
| -0.088000 | 2.0951 |
| -0.083000 | 2.3088 |
| -0.078000 | 2.5242 |
| -0.073000 | 2.7395 |
| -0.068000 | 2.9504 |
| -0.063000 | 3.1537 |
| -0.058000 | 3.3464 |
| -0.053000 | 3.5243 |
| -0.048000 | 3.6836 |
| -0.043000 | 3.8230 |
| -0.038000 | 3.9397 |
| -0.033000 | 4.0313 |
| -0.028000 | 4.0999 |
| -0.023000 | 4.1452 |
| -0.018000 | 4.1682 |
| -0.013000 | 4.1727 |
| -0.0080000 | 4.1592 |
| -0.0030000 | 4.1307 |
| 0.0020000 | 4.0911 |
| 0.0070000 | 4.0405 |
| 0.012000 | 3.9812 |

| Column 1 | Column 2 |
| --- | --- |
| 0.017000 | 3.9151 |
| 0.022000 | 3.8444 |
| 0.027000 | 3.7710 |
| 0.032000 | 3.6941 |
| 0.037000 | 3.6152 |
| 0.042000 | 3.5360 |
| 0.047000 | 3.4565 |
| 0.052000 | 3.3766 |
| 0.057000 | 3.2969 |
| 0.062000 | 3.2171 |
| 0.067000 | 3.1379 |
| 0.072000 | 3.0603 |
| 0.077000 | 2.9849 |
| 0.082000 | 2.9101 |
| 0.087000 | 2.8356 |
| 0.092000 | 2.7627 |
| 0.097000 | 2.6918 |
| 0.10200 | 2.6227 |
| 0.10700 | 2.5550 |
| 0.11200 | 2.4889 |
| 0.11700 | 2.4236 |
| 0.12200 | 2.3606 |
| 0.12700 | 2.2985 |
| 0.13200 | 2.2380 |
| 0.13700 | 2.1790 |
| 0.14200 | 2.1213 |
| 0.14700 | 2.0655 |
| 0.15200 | 2.0102 |
| 0.15700 | 1.9573 |
| 0.16200 | 1.9047 |
| 0.16700 | 1.8550 |
| 0.17200 | 1.8049 |
| 0.17700 | 1.7572 |
| 0.18200 | 1.7091 |
| 0.18700 | 1.6636 |

| Column 1 | Column 2 |
|---|---|
| 0.19200 | 1.6211 |
| 0.19700 | 1.5719 |
| 0.19800 | 1.5495 |
| 0.19300 | 1.4942 |
| 0.18800 | 1.4290 |
| 0.18300 | 1.3925 |
| 0.17800 | 1.3512 |
| 0.17300 | 1.3122 |
| 0.16800 | 1.2744 |
| 0.16300 | 1.2375 |
| 0.15800 | 1.2018 |
| 0.15300 | 1.1666 |
| 0.14800 | 1.1330 |
| 0.14300 | 1.0989 |
| 0.13800 | 1.0671 |
| 0.13300 | 1.0359 |
| 0.12800 | 1.0050 |
| 0.12300 | 0.97470 |
| 0.11800 | 0.94535 |
| 0.11300 | 0.91683 |
| 0.10800 | 0.88864 |
| 0.10300 | 0.86072 |
| 0.098000 | 0.83297 |
| 0.093000 | 0.80579 |
| 0.088000 | 0.77901 |
| 0.083000 | 0.75275 |
| 0.078000 | 0.72533 |
| 0.073000 | 0.69789 |
| 0.068000 | 0.67107 |
| 0.063000 | 0.64304 |
| 0.058000 | 0.61370 |
| 0.053000 | 0.58326 |
| 0.048000 | 0.55121 |
| 0.043000 | 0.51726 |
| 0.038000 | 0.48062 |

| Column 1 | Column 2 |
|---|---|
| 0.033000 | 0.44048 |
| 0.028000 | 0.39623 |
| 0.023000 | 0.34690 |
| 0.018000 | 0.29218 |
| 0.013000 | 0.23001 |
| 0.0080000 | 0.15933 |
| 0.0030000 | 0.078470 |
| -0.0020000 | -0.014480 |
| -0.0070000 | -0.11900 |
| -0.012000 | -0.23748 |
| -0.017000 | -0.37275 |
| -0.022000 | -0.52567 |
| -0.027000 | -0.69677 |
| -0.032000 | -0.88718 |
| -0.037000 | -1.0973 |
| -0.042000 | -1.3259 |
| -0.047000 | -1.5725 |
| -0.052000 | -1.8355 |
| -0.057000 | -2.1114 |
| -0.062000 | -2.3968 |
| -0.067000 | -2.6881 |
| -0.072000 | -2.9803 |
| -0.077000 | -3.2698 |
| -0.082000 | -3.5515 |
| -0.087000 | -3.8206 |
| -0.092000 | -4.0733 |
| -0.097000 | -4.3054 |
| -0.10200 | -4.5146 |
| -0.10700 | -4.6975 |
| -0.11200 | -4.8526 |
| -0.11700 | -4.9799 |
| -0.12200 | -5.0770 |
| -0.12700 | -5.1442 |
| -0.13200 | -5.1839 |
| -0.13700 | -5.1978 |

| Column 1 | Column 2 |
|---|---|
| -0.14200 | -5.1868 |
| -0.14700 | -5.1514 |
| -0.15200 | -5.0947 |
| -0.15700 | -5.0201 |
| -0.16200 | -4.9298 |
| -0.16700 | -4.8256 |
| -0.17200 | -4.7099 |
| -0.17700 | -4.5867 |
| -0.18200 | -4.4572 |
| -0.18700 | -4.3214 |
| -0.19200 | -4.1827 |
| -0.19700 | -4.0447 |
| -0.20200 | -3.9059 |
| -0.20700 | -3.7676 |
| -0.21200 | -3.6325 |
| -0.21700 | -3.4990 |
| -0.22200 | -3.3677 |
| -0.22700 | -3.2400 |
| -0.23200 | -3.1166 |
| -0.23700 | -2.9969 |
| -0.24200 | -2.8808 |
| -0.24700 | -2.7684 |
| -0.25200 | -2.6597 |
| -0.25700 | -2.5557 |
| -0.26200 | -2.4557 |
| -0.26700 | -2.3582 |
| -0.27200 | -2.2643 |
| -0.27700 | -2.1756 |
| -0.28200 | -2.0898 |
| -0.28700 | -2.0067 |
| -0.29200 | -1.9278 |
| -0.29700 | -1.8531 |
| -0.30200 | -1.7799 |
| -0.30700 | -1.7098 |
| -0.31200 | -1.6436 |

| Column 1 | Column 2 |
|---|---|
| -0.31700 | -1.5798 |
| -0.32200 | -1.5183 |
| -0.32700 | -1.4590 |
| -0.33200 | -1.4030 |
| -0.33700 | -1.3479 |
| -0.34200 | -1.2981 |
| -0.34700 | -1.2456 |

### 8.3.29 CV-40

| Column 1 | Column 2 |
|---|---|
| -0.34800 | -1.2072 |
| -0.34300 | -1.1539 |
| -0.33800 | -1.0892 |
| -0.33300 | -1.0497 |
| -0.32800 | -1.0057 |
| -0.32300 | -0.96614 |
| -0.31800 | -0.92766 |
| -0.31300 | -0.88990 |
| -0.30800 | -0.85487 |
| -0.30300 | -0.82079 |
| -0.29800 | -0.78834 |
| -0.29300 | -0.75623 |
| -0.28800 | -0.72558 |
| -0.28300 | -0.69607 |
| -0.27800 | -0.66786 |
| -0.27300 | -0.63988 |
| -0.26800 | -0.61275 |
| -0.26300 | -0.58666 |
| -0.25800 | -0.56138 |
| -0.25300 | -0.53587 |
| -0.24800 | -0.51048 |
| -0.24300 | -0.48599 |
| -0.23800 | -0.46124 |
| -0.23300 | -0.43614 |
| -0.22800 | -0.41014 |

| Column 1 | Column 2 |
|---|---|
| -0.22300 | -0.38352 |
| -0.21800 | -0.35565 |
| -0.21300 | -0.32620 |
| -0.20800 | -0.29491 |
| -0.20300 | -0.26077 |
| -0.19800 | -0.22348 |
| -0.19300 | -0.18280 |
| -0.18800 | -0.13814 |
| -0.18300 | -0.088720 |
| -0.17800 | -0.033400 |
| -0.17300 | 0.028980 |
| -0.16800 | 0.098080 |
| -0.16300 | 0.17487 |
| -0.15800 | 0.26102 |
| -0.15300 | 0.35699 |
| -0.14800 | 0.46258 |
| -0.14300 | 0.57889 |
| -0.13800 | 0.70753 |
| -0.13300 | 0.84856 |
| -0.12800 | 1.0020 |
| -0.12300 | 1.1686 |
| -0.11800 | 1.3488 |
| -0.11300 | 1.5429 |
| -0.10800 | 1.7515 |
| -0.10300 | 1.9734 |
| -0.098000 | 2.2074 |
| -0.093000 | 2.4536 |
| -0.088000 | 2.7100 |
| -0.083000 | 2.9735 |
| -0.078000 | 3.2422 |
| -0.073000 | 3.5128 |
| -0.068000 | 3.7802 |
| -0.063000 | 4.0399 |
| -0.058000 | 4.2871 |
| -0.053000 | 4.5170 |

| Column 1 | Column 2 |
|---|---|
| -0.048000 | 4.7236 |
| -0.043000 | 4.9013 |
| -0.038000 | 5.0480 |
| -0.033000 | 5.1615 |
| -0.028000 | 5.2400 |
| -0.023000 | 5.2851 |
| -0.018000 | 5.2983 |
| -0.013000 | 5.2819 |
| -0.0080000 | 5.2405 |
| -0.0030000 | 5.1769 |
| 0.0020000 | 5.0946 |
| 0.0070000 | 4.9980 |
| 0.012000 | 4.8895 |
| 0.017000 | 4.7710 |
| 0.022000 | 4.6462 |
| 0.027000 | 4.5178 |
| 0.032000 | 4.3862 |
| 0.037000 | 4.2535 |
| 0.042000 | 4.1205 |
| 0.047000 | 3.9873 |
| 0.052000 | 3.8561 |
| 0.057000 | 3.7274 |
| 0.062000 | 3.5999 |
| 0.067000 | 3.4748 |
| 0.072000 | 3.3531 |
| 0.077000 | 3.2343 |
| 0.082000 | 3.1191 |
| 0.087000 | 3.0075 |
| 0.092000 | 2.8981 |
| 0.097000 | 2.7918 |
| 0.10200 | 2.6902 |
| 0.10700 | 2.5910 |
| 0.11200 | 2.4946 |
| 0.11700 | 2.4023 |
| 0.12200 | 2.3128 |

| Column 1 | Column 2 |
|---|---|
| 0.12700 | 2.2262 |
| 0.13200 | 2.1427 |
| 0.13700 | 2.0628 |
| 0.14200 | 1.9853 |
| 0.14700 | 1.9102 |
| 0.15200 | 1.8383 |
| 0.15700 | 1.7692 |
| 0.16200 | 1.7018 |
| 0.16700 | 1.6373 |
| 0.17200 | 1.5757 |
| 0.17700 | 1.5154 |
| 0.18200 | 1.4582 |
| 0.18700 | 1.4022 |
| 0.19200 | 1.3511 |
| 0.19700 | 1.2957 |
| 0.19800 | 1.2568 |
| 0.19300 | 1.1743 |
| 0.18800 | 1.1366 |
| 0.18300 | 1.0878 |
| 0.17800 | 1.0431 |
| 0.17300 | 1.0003 |
| 0.16800 | 0.95827 |
| 0.16300 | 0.91924 |
| 0.15800 | 0.88031 |
| 0.15300 | 0.84405 |
| 0.14800 | 0.80833 |
| 0.14300 | 0.77451 |
| 0.13800 | 0.74136 |
| 0.13300 | 0.71011 |
| 0.12800 | 0.67954 |
| 0.12300 | 0.64940 |
| 0.11800 | 0.62118 |
| 0.11300 | 0.59312 |
| 0.10800 | 0.56589 |
| 0.10300 | 0.53941 |

| Column 1 | Column 2 |
|---|---|
| 0.098000 | 0.51339 |
| 0.093000 | 0.48769 |
| 0.088000 | 0.46243 |
| 0.083000 | 0.43703 |
| 0.078000 | 0.41144 |
| 0.073000 | 0.38538 |
| 0.068000 | 0.35898 |
| 0.063000 | 0.33161 |
| 0.058000 | 0.30231 |
| 0.053000 | 0.27144 |
| 0.048000 | 0.23785 |
| 0.043000 | 0.20083 |
| 0.038000 | 0.16027 |
| 0.033000 | 0.11519 |
| 0.028000 | 0.063430 |
| 0.023000 | 0.0046600 |
| 0.018000 | -0.062080 |
| 0.013000 | -0.13935 |
| 0.0080000 | -0.22818 |
| 0.0030000 | -0.33158 |
| -0.0020000 | -0.45163 |
| -0.0070000 | -0.58814 |
| -0.012000 | -0.74414 |
| -0.017000 | -0.92270 |
| -0.022000 | -1.1246 |
| -0.027000 | -1.3509 |
| -0.032000 | -1.6018 |
| -0.037000 | -1.8775 |
| -0.042000 | -2.1766 |
| -0.047000 | -2.4959 |
| -0.052000 | -2.8337 |
| -0.057000 | -3.1844 |
| -0.062000 | -3.5421 |
| -0.067000 | -3.9028 |
| -0.072000 | -4.2594 |

| Column 1 | Column 2 |
|---|---|
| -0.077000 | -4.6051 |
| -0.082000 | -4.9337 |
| -0.087000 | -5.2391 |
| -0.092000 | -5.5165 |
| -0.097000 | -5.7609 |
| -0.10200 | -5.9702 |
| -0.10700 | -6.1403 |
| -0.11200 | -6.2689 |
| -0.11700 | -6.3577 |
| -0.12200 | -6.4058 |
| -0.12700 | -6.4139 |
| -0.13200 | -6.3844 |
| -0.13700 | -6.3210 |
| -0.14200 | -6.2264 |
| -0.14700 | -6.1026 |
| -0.15200 | -5.9560 |
| -0.15700 | -5.7886 |
| -0.16200 | -5.6027 |
| -0.16700 | -5.4058 |
| -0.17200 | -5.2000 |
| -0.17700 | -4.9869 |
| -0.18200 | -4.7721 |
| -0.18700 | -4.5568 |
| -0.19200 | -4.3412 |
| -0.19700 | -4.1293 |
| -0.20200 | -3.9234 |
| -0.20700 | -3.7234 |
| -0.21200 | -3.5299 |
| -0.21700 | -3.3440 |
| -0.22200 | -3.1647 |
| -0.22700 | -2.9934 |
| -0.23200 | -2.8304 |
| -0.23700 | -2.6751 |
| -0.24200 | -2.5279 |
| -0.24700 | -2.3883 |

| Column 1 | Column 2 |
|---|---|
| -0.25200 | -2.2567 |
| -0.25700 | -2.1320 |
| -0.26200 | -2.0137 |
| -0.26700 | -1.9009 |
| -0.27200 | -1.7957 |
| -0.27700 | -1.6973 |
| -0.28200 | -1.6050 |
| -0.28700 | -1.5175 |
| -0.29200 | -1.4355 |
| -0.29700 | -1.3586 |
| -0.30200 | -1.2839 |
| -0.30700 | -1.2137 |
| -0.31200 | -1.1477 |
| -0.31700 | -1.0875 |
| -0.32200 | -1.0302 |
| -0.32700 | -0.97779 |
| -0.33200 | -0.92710 |
| -0.33700 | -0.87959 |
| -0.34200 | -0.83620 |
| -0.34700 | -0.78893 |

### 8.3.30 CV-41

| Column 1 | Column 2 |
|---|---|
| -0.34800 | -0.76412 |
| -0.34300 | -0.73065 |
| -0.33800 | -0.65631 |
| -0.33300 | -0.62861 |
| -0.32800 | -0.59289 |
| -0.32300 | -0.56166 |
| -0.31800 | -0.53236 |
| -0.31300 | -0.50264 |
| -0.30800 | -0.47695 |
| -0.30300 | -0.45079 |
| -0.29800 | -0.42736 |
| -0.29300 | -0.40372 |

| Column 1 | Column 2 |
|---|---|
| -0.28800 | -0.38248 |
| -0.28300 | -0.36101 |
| -0.27800 | -0.34105 |
| -0.27300 | -0.32145 |
| -0.26800 | -0.30206 |
| -0.26300 | -0.28372 |
| -0.25800 | -0.26535 |
| -0.25300 | -0.24727 |
| -0.24800 | -0.22859 |
| -0.24300 | -0.21038 |
| -0.23800 | -0.19149 |
| -0.23300 | -0.17225 |
| -0.22800 | -0.15219 |
| -0.22300 | -0.13062 |
| -0.21800 | -0.10702 |
| -0.21300 | -0.081610 |
| -0.20800 | -0.053540 |
| -0.20300 | -0.021940 |
| -0.19800 | 0.013430 |
| -0.19300 | 0.053570 |
| -0.18800 | 0.098500 |
| -0.18300 | 0.14977 |
| -0.17800 | 0.20778 |
| -0.17300 | 0.27414 |
| -0.16800 | 0.34961 |
| -0.16300 | 0.43378 |
| -0.15800 | 0.52883 |
| -0.15300 | 0.63632 |
| -0.14800 | 0.75650 |
| -0.14300 | 0.89021 |
| -0.13800 | 1.0383 |
| -0.13300 | 1.2022 |
| -0.12800 | 1.3831 |
| -0.12300 | 1.5810 |
| -0.11800 | 1.7968 |

| Column 1 | Column 2 |
|---|---|
| -0.11300 | 2.0313 |
| -0.10800 | 2.2852 |
| -0.10300 | 2.5579 |
| -0.098000 | 2.8480 |
| -0.093000 | 3.1556 |
| -0.088000 | 3.4792 |
| -0.083000 | 3.8150 |
| -0.078000 | 4.1594 |
| -0.073000 | 4.5087 |
| -0.068000 | 4.8566 |
| -0.063000 | 5.1955 |
| -0.058000 | 5.5183 |
| -0.053000 | 5.8170 |
| -0.048000 | 6.0826 |
| -0.043000 | 6.3076 |
| -0.038000 | 6.4859 |
| -0.033000 | 6.6136 |
| -0.028000 | 6.6897 |
| -0.023000 | 6.7121 |
| -0.018000 | 6.6843 |
| -0.013000 | 6.6134 |
| -0.0080000 | 6.5027 |
| -0.0030000 | 6.3582 |
| 0.0020000 | 6.1885 |
| 0.0070000 | 5.9976 |
| 0.012000 | 5.7894 |
| 0.017000 | 5.5710 |
| 0.022000 | 5.3465 |
| 0.027000 | 5.1190 |
| 0.032000 | 4.8904 |
| 0.037000 | 4.6630 |
| 0.042000 | 4.4402 |
| 0.047000 | 4.2225 |
| 0.052000 | 4.0104 |
| 0.057000 | 3.8056 |

| Column 1 | Column 2 |
|---|---|
| 0.062000 | 3.6079 |
| 0.067000 | 3.4174 |
| 0.072000 | 3.2352 |
| 0.077000 | 3.0603 |
| 0.082000 | 2.8936 |
| 0.087000 | 2.7351 |
| 0.092000 | 2.5842 |
| 0.097000 | 2.4401 |
| 0.10200 | 2.3026 |
| 0.10700 | 2.1730 |
| 0.11200 | 2.0507 |
| 0.11700 | 1.9346 |
| 0.12200 | 1.8241 |
| 0.12700 | 1.7195 |
| 0.13200 | 1.6207 |
| 0.13700 | 1.5284 |
| 0.14200 | 1.4404 |
| 0.14700 | 1.3576 |
| 0.15200 | 1.2796 |
| 0.15700 | 1.2057 |
| 0.16200 | 1.1367 |
| 0.16700 | 1.0706 |
| 0.17200 | 1.0096 |
| 0.17700 | 0.95033 |
| 0.18200 | 0.89718 |
| 0.18700 | 0.84438 |
| 0.19200 | 0.79772 |
| 0.19700 | 0.74857 |
| 0.19800 | 0.70778 |
| 0.19300 | 0.62333 |
| 0.18800 | 0.61232 |
| 0.18300 | 0.56601 |
| 0.17800 | 0.53108 |
| 0.17300 | 0.49680 |
| 0.16800 | 0.46399 |

| Column 1 | Column 2 |
|---|---|
| 0.16300 | 0.43545 |
| 0.15800 | 0.40596 |
| 0.15300 | 0.38063 |
| 0.14800 | 0.35491 |
| 0.14300 | 0.33123 |
| 0.13800 | 0.30815 |
| 0.13300 | 0.28723 |
| 0.12800 | 0.26680 |
| 0.12300 | 0.24716 |
| 0.11800 | 0.22843 |
| 0.11300 | 0.20956 |
| 0.10800 | 0.19179 |
| 0.10300 | 0.17452 |
| 0.098000 | 0.15797 |
| 0.093000 | 0.14061 |
| 0.088000 | 0.12362 |
| 0.083000 | 0.10616 |
| 0.078000 | 0.087600 |
| 0.073000 | 0.067900 |
| 0.068000 | 0.046400 |
| 0.063000 | 0.022730 |
| 0.058000 | -0.0034300 |
| 0.053000 | -0.032360 |
| 0.048000 | -0.065920 |
| 0.043000 | -0.10492 |
| 0.038000 | -0.14995 |
| 0.033000 | -0.20247 |
| 0.028000 | -0.26578 |
| 0.023000 | -0.34065 |
| 0.018000 | -0.42718 |
| 0.013000 | -0.52853 |
| 0.0080000 | -0.64749 |
| 0.0030000 | -0.78787 |
| -0.0020000 | -0.95378 |
| -0.0070000 | -1.1450 |

| Column 1 | Column 2 |
|---|---|
| -0.012000 | -1.3623 |
| -0.017000 | -1.6096 |
| -0.022000 | -1.8891 |
| -0.027000 | -2.2020 |
| -0.032000 | -2.5475 |
| -0.037000 | -2.9226 |
| -0.042000 | -3.3239 |
| -0.047000 | -3.7484 |
| -0.052000 | -4.1909 |
| -0.057000 | -4.6415 |
| -0.062000 | -5.0922 |
| -0.067000 | -5.5353 |
| -0.072000 | -5.9596 |
| -0.077000 | -6.3560 |
| -0.082000 | -6.7161 |
| -0.087000 | -7.0312 |
| -0.092000 | -7.2963 |
| -0.097000 | -7.5071 |
| -0.10200 | -7.6598 |
| -0.10700 | -7.7517 |
| -0.11200 | -7.7818 |
| -0.11700 | -7.7540 |
| -0.12200 | -7.6715 |
| -0.12700 | -7.5381 |
| -0.13200 | -7.3591 |
| -0.13700 | -7.1392 |
| -0.14200 | -6.8839 |
| -0.14700 | -6.6004 |
| -0.15200 | -6.2948 |
| -0.15700 | -5.9733 |
| -0.16200 | -5.6437 |
| -0.16700 | -5.3099 |
| -0.17200 | -4.9757 |
| -0.17700 | -4.6460 |
| -0.18200 | -4.3267 |

| Column 1 | Column 2 |
|---|---|
| -0.18700 | -4.0196 |
| -0.19200 | -3.7243 |
| -0.19700 | -3.4444 |
| -0.20200 | -3.1810 |
| -0.20700 | -2.9337 |
| -0.21200 | -2.7021 |
| -0.21700 | -2.4861 |
| -0.22200 | -2.2864 |
| -0.22700 | -2.1025 |
| -0.23200 | -1.9334 |
| -0.23700 | -1.7781 |
| -0.24200 | -1.6351 |
| -0.24700 | -1.5039 |
| -0.25200 | -1.3836 |
| -0.25700 | -1.2731 |
| -0.26200 | -1.1728 |
| -0.26700 | -1.0798 |
| -0.27200 | -0.99454 |
| -0.27700 | -0.91652 |
| -0.28200 | -0.84547 |
| -0.28700 | -0.77991 |
| -0.29200 | -0.72017 |
| -0.29700 | -0.66553 |
| -0.30200 | -0.61429 |
| -0.30700 | -0.56835 |
| -0.31200 | -0.52616 |
| -0.31700 | -0.48832 |
| -0.32200 | -0.45134 |
| -0.32700 | -0.41996 |
| -0.33200 | -0.38880 |
| -0.33700 | -0.36204 |
| -0.34200 | -0.33760 |
| -0.34700 | -0.30939 |

### 8.3.31 CV-42

| Column 1 | Column 2 |
|---|---|
| -0.34800 | -0.29552 |
| -0.34300 | -0.27808 |
| -0.33800 | -0.23385 |
| -0.33300 | -0.22025 |
| -0.32800 | -0.20136 |
| -0.32300 | -0.18530 |
| -0.31800 | -0.17131 |
| -0.31300 | -0.15620 |
| -0.30800 | -0.14457 |
| -0.30300 | -0.13133 |
| -0.29800 | -0.12077 |
| -0.29300 | -0.11003 |
| -0.28800 | -0.10035 |
| -0.28300 | -0.090160 |
| -0.27800 | -0.081050 |
| -0.27300 | -0.072150 |
| -0.26800 | -0.062750 |
| -0.26300 | -0.053850 |
| -0.25800 | -0.044480 |
| -0.25300 | -0.034810 |
| -0.24800 | -0.024400 |
| -0.24300 | -0.013990 |
| -0.23800 | -0.0017000 |
| -0.23300 | 0.011890 |
| -0.22800 | 0.026760 |
| -0.22300 | 0.043310 |
| -0.21800 | 0.062750 |
| -0.21300 | 0.085270 |
| -0.20800 | 0.11109 |
| -0.20300 | 0.14188 |
| -0.19800 | 0.17704 |
| -0.19300 | 0.21808 |
| -0.18800 | 0.26635 |
| -0.18300 | 0.32207 |

| Column 1 | Column 2 |
|---|---|
| -0.17800 | 0.38614 |
| -0.17300 | 0.46051 |
| -0.16800 | 0.54621 |
| -0.16300 | 0.64392 |
| -0.15800 | 0.75497 |
| -0.15300 | 0.88121 |
| -0.14800 | 1.0233 |
| -0.14300 | 1.1825 |
| -0.13800 | 1.3613 |
| -0.13300 | 1.5595 |
| -0.12800 | 1.7800 |
| -0.12300 | 2.0247 |
| -0.11800 | 2.2932 |
| -0.11300 | 2.5872 |
| -0.10800 | 2.9080 |
| -0.10300 | 3.2560 |
| -0.098000 | 3.6304 |
| -0.093000 | 4.0309 |
| -0.088000 | 4.4550 |
| -0.083000 | 4.8990 |
| -0.078000 | 5.3581 |
| -0.073000 | 5.8251 |
| -0.068000 | 6.2903 |
| -0.063000 | 6.7431 |
| -0.058000 | 7.1709 |
| -0.053000 | 7.5600 |
| -0.048000 | 7.8967 |
| -0.043000 | 8.1661 |
| -0.038000 | 8.3571 |
| -0.033000 | 8.4636 |
| -0.028000 | 8.4813 |
| -0.023000 | 8.4117 |
| -0.018000 | 8.2605 |
| -0.013000 | 8.0370 |
| -0.0080000 | 7.7530 |

| Column 1 | Column 2 |
|---|---|
| -0.0030000 | 7.4215 |
| 0.0020000 | 7.0554 |
| 0.0070000 | 6.6636 |
| 0.012000 | 6.2575 |
| 0.017000 | 5.8482 |
| 0.022000 | 5.4418 |
| 0.027000 | 5.0443 |
| 0.032000 | 4.6597 |
| 0.037000 | 4.2920 |
| 0.042000 | 3.9430 |
| 0.047000 | 3.6136 |
| 0.052000 | 3.3048 |
| 0.057000 | 3.0163 |
| 0.062000 | 2.7488 |
| 0.067000 | 2.5016 |
| 0.072000 | 2.2739 |
| 0.077000 | 2.0634 |
| 0.082000 | 1.8705 |
| 0.087000 | 1.6950 |
| 0.092000 | 1.5349 |
| 0.097000 | 1.3889 |
| 0.10200 | 1.2559 |
| 0.10700 | 1.1354 |
| 0.11200 | 1.0267 |
| 0.11700 | 0.92847 |
| 0.12200 | 0.83916 |
| 0.12700 | 0.75897 |
| 0.13200 | 0.68610 |
| 0.13700 | 0.62128 |
| 0.14200 | 0.56243 |
| 0.14700 | 0.50939 |
| 0.15200 | 0.46268 |
| 0.15700 | 0.41977 |
| 0.16200 | 0.38215 |
| 0.16700 | 0.34787 |

| Column 1 | Column 2 |
|---|---|
| 0.17200 | 0.31881 |
| 0.17700 | 0.29038 |
| 0.18200 | 0.26772 |
| 0.18700 | 0.24596 |
| 0.19200 | 0.22786 |
| 0.19700 | 0.20972 |
| 0.19800 | 0.17619 |
| 0.19300 | 0.14532 |
| 0.18800 | 0.14748 |
| 0.18300 | 0.12794 |
| 0.17800 | 0.11542 |
| 0.17300 | 0.10404 |
| 0.16800 | 0.093220 |
| 0.16300 | 0.084640 |
| 0.15800 | 0.075240 |
| 0.15300 | 0.068160 |
| 0.14800 | 0.060870 |
| 0.14300 | 0.054280 |
| 0.13800 | 0.047570 |
| 0.13300 | 0.041580 |
| 0.12800 | 0.035180 |
| 0.12300 | 0.028650 |
| 0.11800 | 0.022480 |
| 0.11300 | 0.015410 |
| 0.10800 | 0.0074600 |
| 0.10300 | -8.1825E-4 |
| 0.098000 | -0.0096100 |
| 0.093000 | -0.020600 |
| 0.088000 | -0.032780 |
| 0.083000 | -0.046790 |
| 0.078000 | -0.063050 |
| 0.073000 | -0.082000 |
| 0.068000 | -0.10476 |
| 0.063000 | -0.13196 |
| 0.058000 | -0.16459 |

| Column 1 | Column 2 |
|---|---|
| 0.053000 | -0.20318 |
| 0.048000 | -0.24958 |
| 0.043000 | -0.30547 |
| 0.038000 | -0.37185 |
| 0.033000 | -0.45084 |
| 0.028000 | -0.54518 |
| 0.023000 | -0.65669 |
| 0.018000 | -0.78926 |
| 0.013000 | -0.94630 |
| 0.0080000 | -1.1292 |
| 0.0030000 | -1.3443 |
| -0.0020000 | -1.5962 |
| -0.0070000 | -1.8836 |
| -0.012000 | -2.2098 |
| -0.017000 | -2.5790 |
| -0.022000 | -2.9920 |
| -0.027000 | -3.4470 |
| -0.032000 | -3.9401 |
| -0.037000 | -4.4680 |
| -0.042000 | -5.0218 |
| -0.047000 | -5.5913 |
| -0.052000 | -6.1665 |
| -0.057000 | -6.7311 |
| -0.062000 | -7.2700 |
| -0.067000 | -7.7699 |
| -0.072000 | -8.2156 |
| -0.077000 | -8.5941 |
| -0.082000 | -8.8941 |
| -0.087000 | -9.1065 |
| -0.092000 | -9.2266 |
| -0.097000 | -9.2535 |
| -0.10200 | -9.1876 |
| -0.10700 | -9.0306 |
| -0.11200 | -8.7919 |
| -0.11700 | -8.4786 |

| Column 1 | Column 2 |
|---|---|
| -0.12200 | -8.0971 |
| -0.12700 | -7.6657 |
| -0.13200 | -7.1990 |
| -0.13700 | -6.7095 |
| -0.14200 | -6.2095 |
| -0.14700 | -5.7080 |
| -0.15200 | -5.2147 |
| -0.15700 | -4.7360 |
| -0.16200 | -4.2785 |
| -0.16700 | -3.8460 |
| -0.17200 | -3.4406 |
| -0.17700 | -3.0657 |
| -0.18200 | -2.7234 |
| -0.18700 | -2.4138 |
| -0.19200 | -2.1346 |
| -0.19700 | -1.8838 |
| -0.20200 | -1.6604 |
| -0.20700 | -1.4620 |
| -0.21200 | -1.2862 |
| -0.21700 | -1.1310 |
| -0.22200 | -0.99392 |
| -0.22700 | -0.87352 |
| -0.23200 | -0.76802 |
| -0.23700 | -0.67493 |
| -0.24200 | -0.59319 |
| -0.24700 | -0.52209 |
| -0.25200 | -0.45948 |
| -0.25700 | -0.40434 |
| -0.26200 | -0.35687 |
| -0.26700 | -0.31478 |
| -0.27200 | -0.27813 |
| -0.27700 | -0.24612 |
| -0.28200 | -0.21815 |
| -0.28700 | -0.19370 |
| -0.29200 | -0.17259 |

| Column 1 | Column 2 |
|---|---|
| -0.29700 | -0.15423 |
| -0.30200 | -0.13785 |
| -0.30700 | -0.12412 |
| -0.31200 | -0.11214 |
| -0.31700 | -0.10248 |
| -0.32200 | -0.092690 |
| -0.32700 | -0.085900 |
| -0.33200 | -0.078860 |
| -0.33700 | -0.073720 |
| -0.34200 | -0.070160 |
| -0.34700 | -0.061920 |

### 8.3.32 CV-43

| Column 1 | Column 2 |
|---|---|
| -0.29552 | -0.056750 |
| -0.27808 | -0.051030 |
| -0.23385 | -0.037870 |
| -0.22025 | -0.035390 |
| -0.20136 | -0.030250 |
| -0.18530 | -0.026400 |
| -0.17131 | -0.023500 |
| -0.15620 | -0.019820 |
| -0.14457 | -0.017210 |
| -0.13133 | -0.013930 |
| -0.12077 | -0.011630 |
| -0.11003 | -0.0089300 |
| -0.10035 | -0.0059100 |
| -0.090160 | -0.0025800 |
| -0.081050 | 5.5869E-4 |
| -0.072150 | 0.0042300 |
| -0.062750 | 0.0086500 |
| -0.053850 | 0.013150 |
| -0.044480 | 0.018470 |
| -0.034810 | 0.025140 |
| -0.024400 | 0.032490 |

| Column 1 | Column 2 |
|---|---|
| -0.013990 | 0.040470 |
| -0.0017000 | 0.050670 |
| 0.011890 | 0.062930 |
| 0.026760 | 0.077230 |
| 0.043310 | 0.093860 |
| 0.062750 | 0.11405 |
| 0.085270 | 0.13824 |
| 0.11109 | 0.16679 |
| 0.14188 | 0.20093 |
| 0.17704 | 0.24052 |
| 0.21808 | 0.28715 |
| 0.26635 | 0.34242 |
| 0.32207 | 0.40658 |
| 0.38614 | 0.48097 |
| 0.46051 | 0.56762 |
| 0.54621 | 0.66756 |
| 0.64392 | 0.78194 |
| 0.75497 | 0.91265 |
| 0.88121 | 1.0618 |
| 1.0233 | 1.2303 |
| 1.1825 | 1.4206 |
| 1.3613 | 1.6359 |
| 1.5595 | 1.8773 |
| 1.7800 | 2.1476 |
| 2.0247 | 2.4497 |
| 2.2932 | 2.7853 |
| 2.5872 | 3.1578 |
| 2.9080 | 3.5692 |
| 3.2560 | 4.0214 |
| 3.6304 | 4.5139 |
| 4.0309 | 5.0461 |
| 4.4550 | 5.6151 |
| 4.8990 | 6.2142 |
| 5.3581 | 6.8357 |
| 5.8251 | 7.4688 |

| Column 1 | Column 2 |
|---|---|
| 6.2903 | 8.0964 |
| 6.7431 | 8.6977 |
| 7.1709 | 9.2489 |
| 7.5600 | 9.7254 |
| 7.8967 | 10.101 |
| 8.1661 | 10.350 |
| 8.3571 | 10.453 |
| 8.4636 | 10.399 |
| 8.4813 | 10.189 |
| 8.4117 | 9.8311 |
| 8.2605 | 9.3429 |
| 8.0370 | 8.7524 |
| 7.7530 | 8.0883 |
| 7.4215 | 7.3834 |
| 7.0554 | 6.6670 |
| 6.6636 | 5.9540 |
| 6.2575 | 5.2666 |
| 5.8482 | 4.6226 |
| 5.4418 | 4.0291 |
| 5.0443 | 3.4901 |
| 4.6597 | 3.0062 |
| 4.2920 | 2.5777 |
| 3.9430 | 2.2010 |
| 3.6136 | 1.8730 |
| 3.3048 | 1.5906 |
| 3.0163 | 1.3475 |
| 2.7488 | 1.1397 |
| 2.5016 | 0.96370 |
| 2.2739 | 0.81517 |
| 2.0634 | 0.68974 |
| 1.8705 | 0.58423 |
| 1.6950 | 0.49630 |
| 1.5349 | 0.42328 |
| 1.3889 | 0.36245 |
| 1.2559 | 0.31246 |

| Column 1 | Column 2 |
|---|---|
| 1.1354 | 0.27115 |
| 1.0267 | 0.23663 |
| 0.92847 | 0.20908 |
| 0.83916 | 0.18674 |
| 0.75897 | 0.16831 |
| 0.68610 | 0.15330 |
| 0.62128 | 0.14168 |
| 0.56243 | 0.13202 |
| 0.50939 | 0.12421 |
| 0.46268 | 0.11815 |
| 0.41977 | 0.11254 |
| 0.38215 | 0.10812 |
| 0.34787 | 0.10449 |
| 0.31881 | 0.10149 |
| 0.29038 | 0.097440 |
| 0.26772 | 0.094740 |
| 0.24596 | 0.091720 |
| 0.22786 | 0.089770 |
| 0.20972 | 0.083560 |
| 0.17619 | 0.067430 |
| 0.14532 | 0.051300 |
| 0.14748 | 0.051360 |
| 0.12794 | 0.042790 |
| 0.11542 | 0.036750 |
| 0.10404 | 0.030540 |
| 0.093220 | 0.025400 |
| 0.084640 | 0.020680 |
| 0.075240 | 0.015330 |
| 0.068160 | 0.010460 |
| 0.060870 | 0.0055000 |
| 0.054280 | 6.1494E-4 |
| 0.047570 | -0.0051400 |
| 0.041580 | -0.010830 |
| 0.035180 | -0.017510 |
| 0.028650 | -0.025310 |

| Column 1 | Column 2 |
|---|---|
| 0.022480 | -0.033180 |
| 0.015410 | -0.042740 |
| 0.0074600 | -0.054210 |
| -8.1825E-4 | -0.067310 |
| -0.0096100 | -0.082610 |
| -0.020600 | -0.10072 |
| -0.032780 | -0.12206 |
| -0.046790 | -0.14735 |
| -0.063050 | -0.17693 |
| -0.082000 | -0.21085 |
| -0.10476 | -0.25038 |
| -0.13196 | -0.29691 |
| -0.16459 | -0.35064 |
| -0.20318 | -0.41238 |
| -0.24958 | -0.48458 |
| -0.30547 | -0.56930 |
| -0.37185 | -0.66960 |
| -0.45084 | -0.78917 |
| -0.54518 | -0.93255 |
| -0.65669 | -1.1050 |
| -0.78926 | -1.3112 |
| -0.94630 | -1.5573 |
| -1.1292 | -1.8457 |
| -1.3443 | -2.1861 |
| -1.5962 | -2.5832 |
| -1.8836 | -3.0281 |
| -2.2098 | -3.5269 |
| -2.5790 | -4.0846 |
| -2.9920 | -4.6943 |
| -3.4470 | -5.3483 |
| -3.9401 | -6.0373 |
| -4.4680 | -6.7473 |
| -5.0218 | -7.4578 |
| -5.5913 | -8.1503 |
| -6.1665 | -8.7980 |

| Column 1 | Column 2 |
|---|---|
| -6.7311 | -9.3755 |
| -7.2700 | -9.8666 |
| -7.7699 | -10.245 |
| -8.2156 | -10.488 |
| -8.5941 | -10.588 |
| -8.8941 | -10.535 |
| -9.1065 | -10.330 |
| -9.2266 | -9.9830 |
| -9.2535 | -9.5052 |
| -9.1876 | -8.9222 |
| -9.0306 | -8.2637 |
| -8.7919 | -7.5573 |
| -8.4786 | -6.8309 |
| -8.0971 | -6.1041 |
| -7.6657 | -5.3989 |
| -7.1990 | -4.7367 |
| -6.7095 | -4.1297 |
| -6.2095 | -3.5807 |
| -5.7080 | -3.0890 |
| -5.2147 | -2.6562 |
| -4.7360 | -2.2774 |
| -4.2785 | -1.9468 |
| -3.8460 | -1.6611 |
| -3.4406 | -1.4146 |
| -3.0657 | -1.2029 |
| -2.7234 | -1.0220 |
| -2.4138 | -0.86731 |
| -2.1346 | -0.73560 |
| -1.8838 | -0.62380 |
| -1.6604 | -0.52846 |
| -1.4620 | -0.44787 |
| -1.2862 | -0.37976 |
| -1.1310 | -0.32198 |
| -0.99392 | -0.27317 |
| -0.87352 | -0.23246 |

| Column 1 | Column 2 |
|---|---|
| -0.76802 | -0.19836 |
| -0.67493 | -0.16936 |
| -0.59319 | -0.14530 |
| -0.52209 | -0.12570 |
| -0.45948 | -0.10931 |
| -0.40434 | -0.095600 |
| -0.35687 | -0.084650 |
| -0.31478 | -0.075540 |
| -0.27813 | -0.067930 |
| -0.24612 | -0.061890 |
| -0.21815 | -0.057070 |
| -0.19370 | -0.052940 |
| -0.17259 | -0.049850 |
| -0.15423 | -0.047420 |
| -0.13785 | -0.045410 |
| -0.12412 | -0.044130 |
| -0.11214 | -0.042820 |
| -0.10248 | -0.042140 |
| -0.092690 | -0.041620 |
| -0.085900 | -0.041580 |
| -0.078860 | -0.041380 |
| -0.073720 | -0.041690 |
| -0.070160 | -0.042740 |
| -0.061920 | -0.040250 |

### 8.3.33 Evaluation 2D

Interactive 2D values

| x | y | Value |
|---|---|---|
| 7.0488 | 0.17889 | 0.93308 |
| 1.8028 | 0.86134 | 0.87319 |

### 8.3.34 Table 42

Global Evaluation 1

### 8.3.35 Table 43

Global Evaluation 1

| Time | n_Ox+n_Red (mol) |
|---|---|
| 0.0000 | 8.4824E-14 |

### 8.3.36 Table 44

| Column 1 | Column 2 |
|---|---|
| 2.7500 | NaN |
| 5.5000 | NaN |
| 8.2500 | -0.11000 |
| 11.000 | NaN |
| 13.750 | -0.10300 |
| 16.500 | NaN |
| 19.250 | -0.14300 |
| 22.000 | NaN |
| 24.750 | -0.13800 |
| 27.500 | NaN |
| 30.250 | -0.13800 |
| 33.000 | NaN |
| 35.750 | -0.13800 |
| 38.500 | NaN |
| 41.250 | -0.13300 |
| 44.000 | NaN |
| 46.750 | -0.13500 |
| 49.500 | NaN |
| 52.250 | -0.13300 |
| 55.000 | NaN |
| 57.750 | -0.13000 |
| 60.500 | NaN |
| 63.250 | -0.12800 |
| 66.000 | NaN |
| 68.750 | -0.12800 |
| 71.500 | NaN |
| 74.250 | -0.098000 |
| 77.000 | NaN |
| 79.750 | -0.11300 |
| 82.500 | NaN |
| 85.250 | -0.12300 |

| Column 1 | Column 2 |
|---|---|
| 88.000 | NaN |
| 90.750 | -0.12300 |
| 93.500 | NaN |
| 96.250 | -0.12300 |
| 99.000 | NaN |
| 101.75 | -0.12300 |
| 104.50 | NaN |
| 107.25 | -0.12300 |
| 110.00 | NaN |
| 112.75 | -0.11800 |
| 115.50 | NaN |
| 118.25 | -0.11600 |
| 121.00 | NaN |
| 123.75 | -0.11400 |
| 126.50 | NaN |
| 129.25 | -0.11400 |
| 132.00 | NaN |
| 134.75 | -0.11300 |
| 137.50 | NaN |
| 140.25 | -0.11000 |
| 143.00 | NaN |
| 145.75 | -0.11300 |
| 148.50 | NaN |
| 151.25 | -0.11500 |
| 154.00 | NaN |
| 156.75 | -0.11200 |
| 159.50 | NaN |
| 162.25 | -0.11000 |
| 165.00 | NaN |
| 167.75 | -0.10800 |
| 170.50 | NaN |
| 173.25 | -0.11000 |
| 176.00 | NaN |
| 178.75 | -0.088000 |
| 181.50 | NaN |

| Column 1 | Column 2 |
|---|---|
| 184.25 | -0.070000 |
| 187.00 | NaN |
| 189.75 | -0.085000 |
| 192.50 | NaN |
| 195.25 | -0.081000 |
| 198.00 | NaN |
| 200.75 | -0.079000 |
| 203.50 | NaN |
| 206.25 | -0.077000 |
| 209.00 | NaN |
| 211.75 | -0.072000 |
| 214.50 | NaN |
| 217.25 | -0.065000 |
| 220.00 | NaN |
| 222.75 | -0.067000 |
| 225.50 | NaN |
| 228.25 | -0.062000 |
| 231.00 | NaN |
| 233.75 | -0.050000 |
| 236.50 | NaN |
| 239.25 | -0.034000 |
| 242.00 | NaN |

## 8.4 PLOT GROUPS

### 8.4.1 voltamogramm

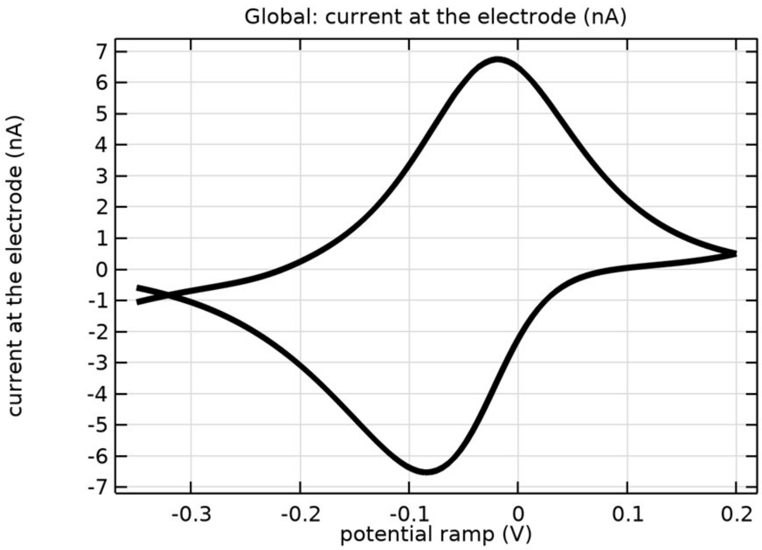


*Global: current at the electrode (nA)*

### 8.4.2 droplet's volume vs time

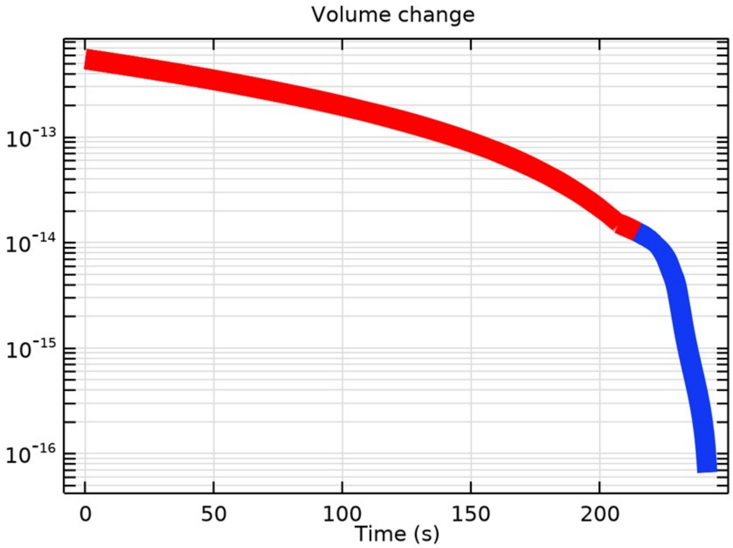


*Volume change*

### 8.4.3 Conservation of matter

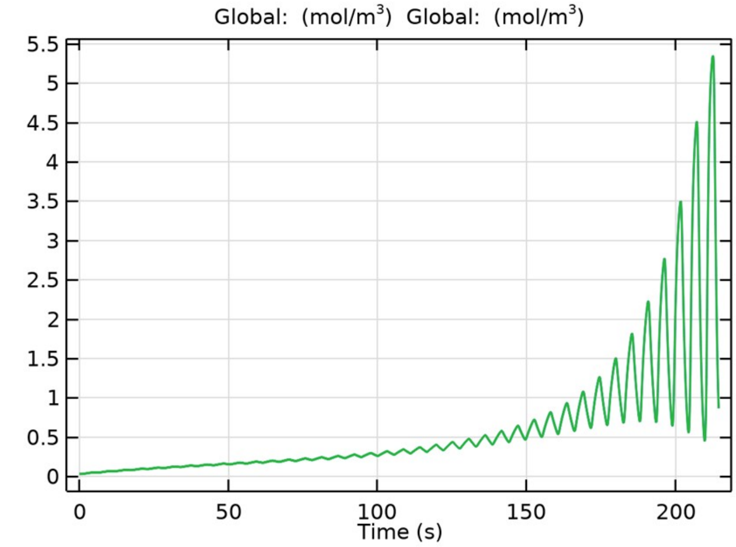


*Global: (mol/m$^3$) Global: (mol/m$^3$) Global: (mol) Global: (mol) Global: (mol)*

### 8.4.4 E0'

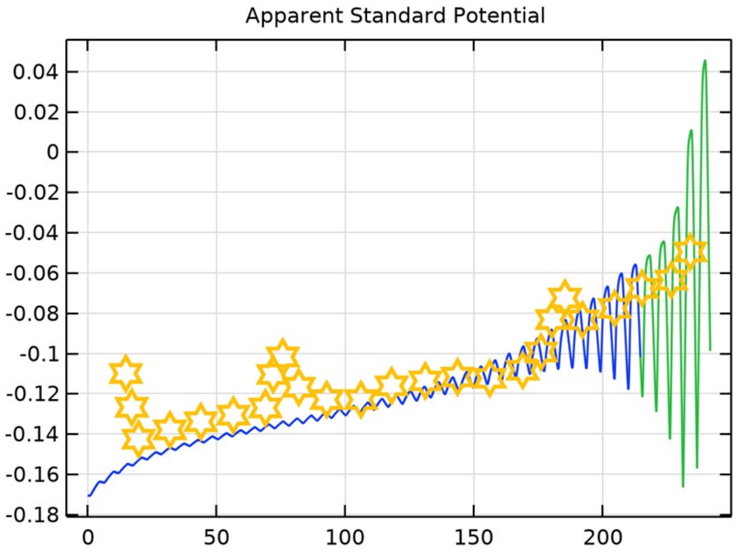


*Apparent Standard Potential*

### 8.4.5 contact radius

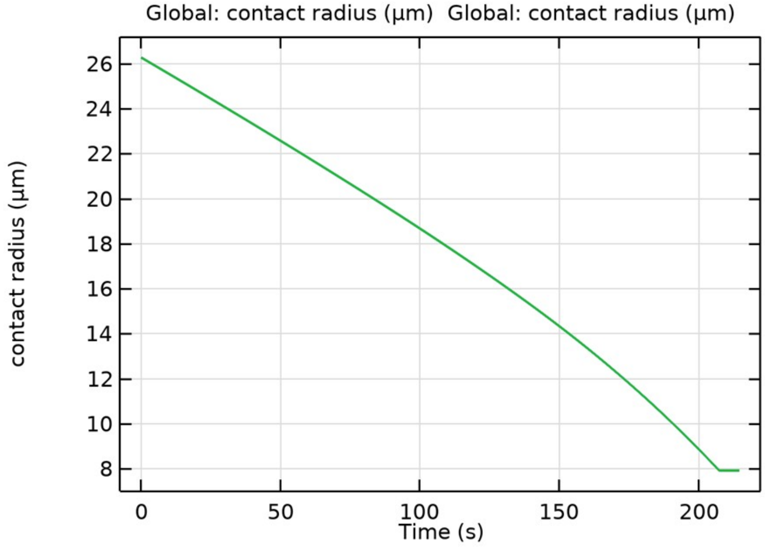


*Global: contact radius (µm) Global: contact radius (µm)*

### 8.4.6 contact angle

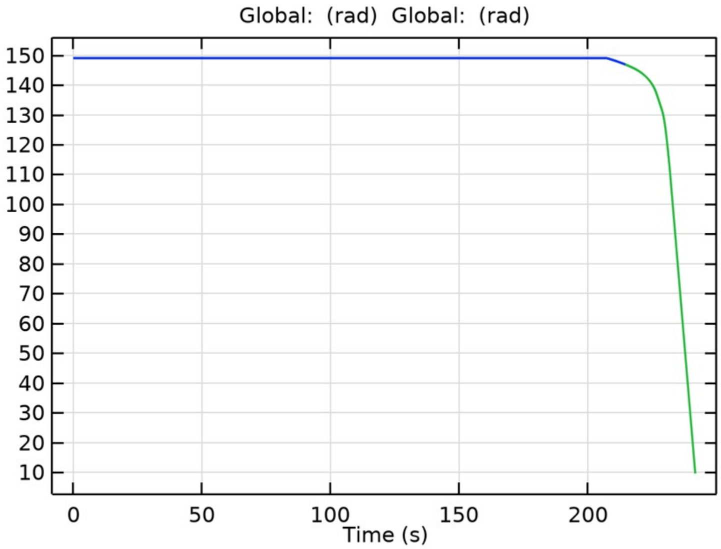


*Global: (rad) Global: (rad) Global: (rad) Global: (rad) Global: (rad)*

### 8.4.7 radius

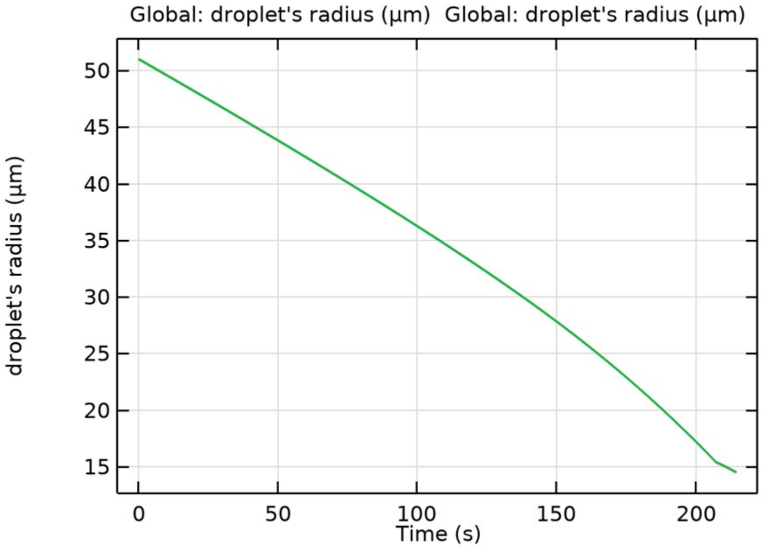


*Global: droplet's radius (µm) Global: droplet's radius (µm)*

### 8.4.8 Vang

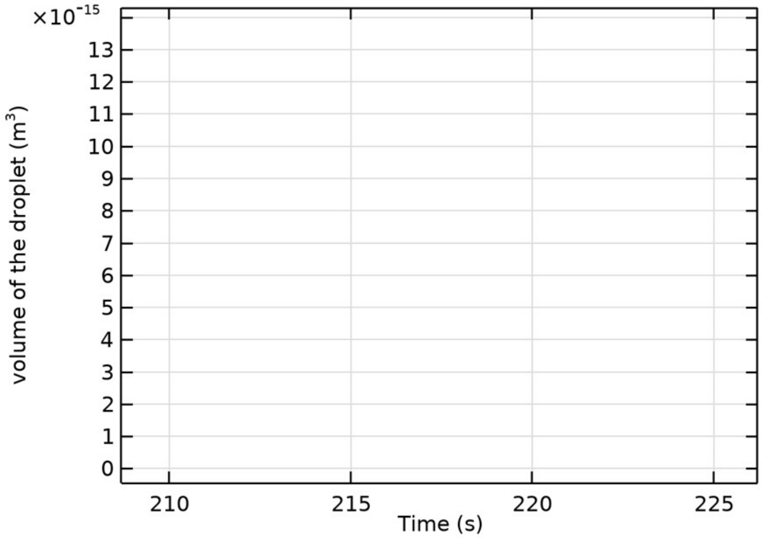


*Point Graph: volume of the droplet ($m^3$)*

## 8.4.9 E1/2

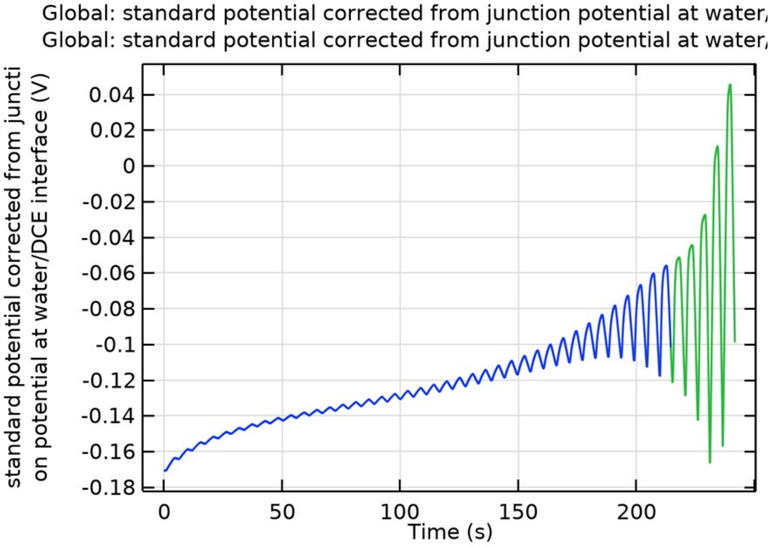


*Global: standard potential corrected from junction potential at water/DCE interface (V) Global: standard potential corrected from junction potential at water/DCE interface (V)*

## 8.4.10 Mesh

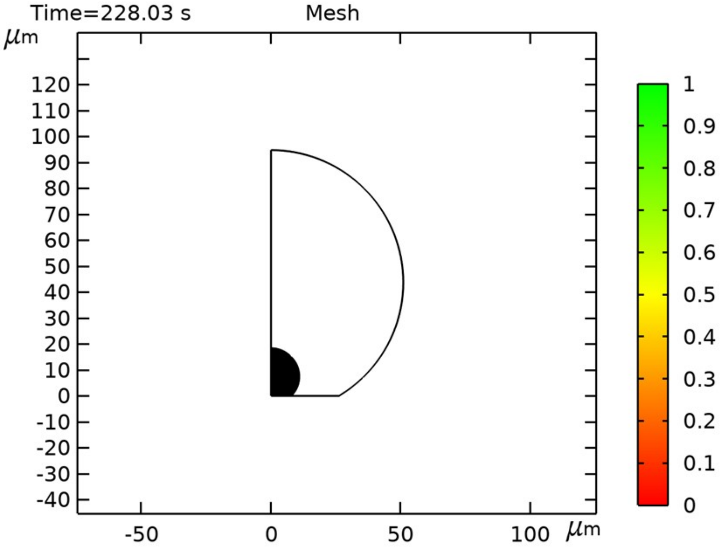


*Mesh*

### 8.4.11 Concentration, Ox (tds)

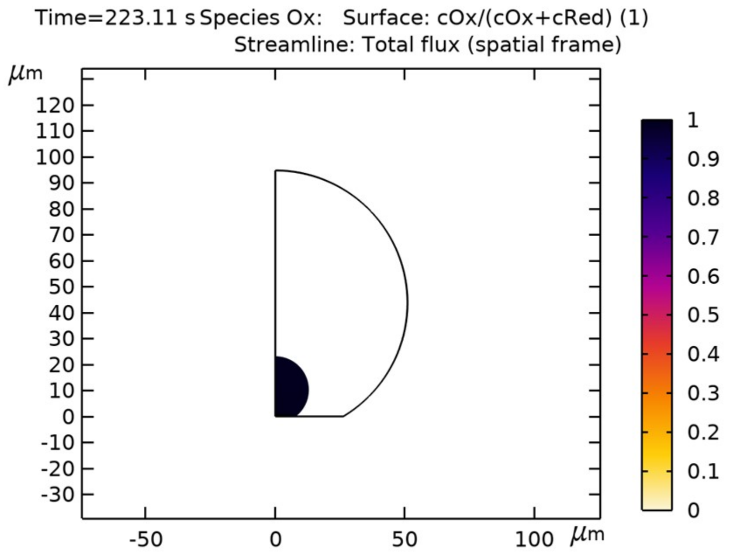


*Species Ox: Surface: cOx/(cOx+cRed) (1) Streamline: Total flux (spatial frame)*

### 8.4.12 Concentration, Ox, 3D (tds)

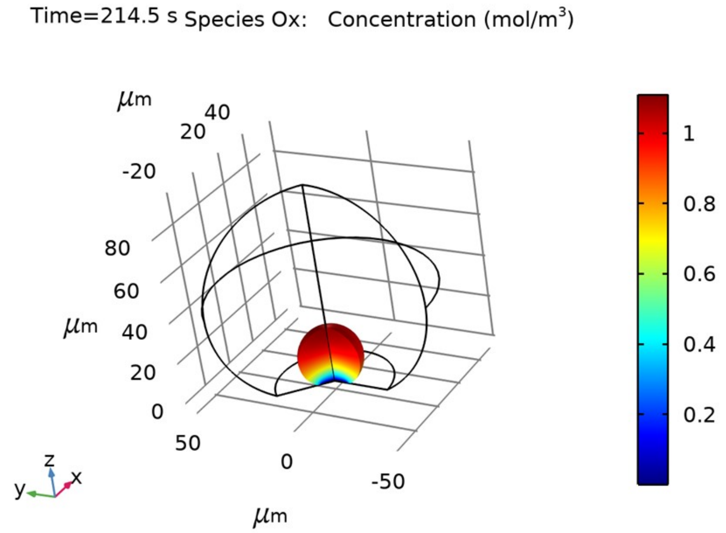


*Species Ox: Concentration (mol/m$^3$)*

## 8.4.13 Concentration, Red (tds)

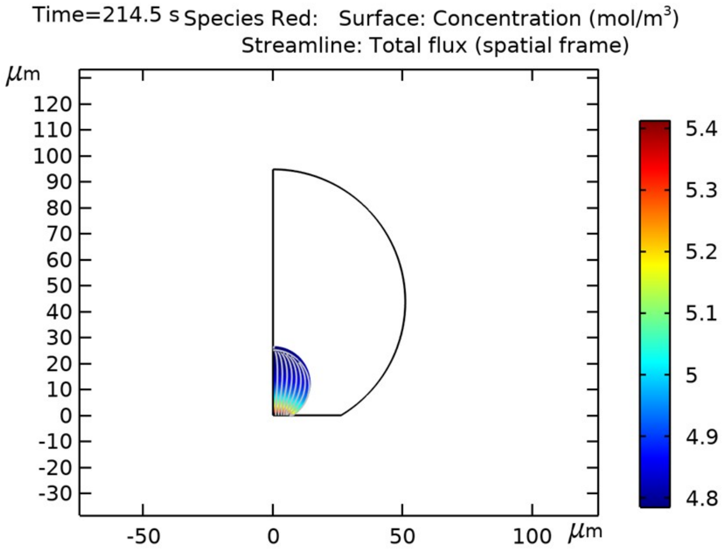


*Species Red: Surface: Concentration (mol/m$^3$) Streamline: Total flux (spatial frame)*

## 8.4.14 Concentration, Red, 3D (tds)

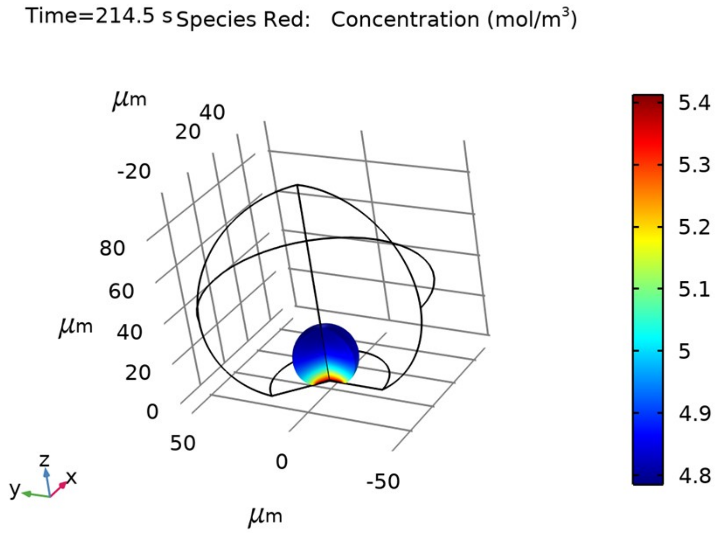


*Species Red: Concentration (mol/m$^3$)*

*Species Red: Surface: Concentration (mol/m$^3$) Streamline: Total flux (spatial frame)*

### 8.4.15 voltamogramm 1

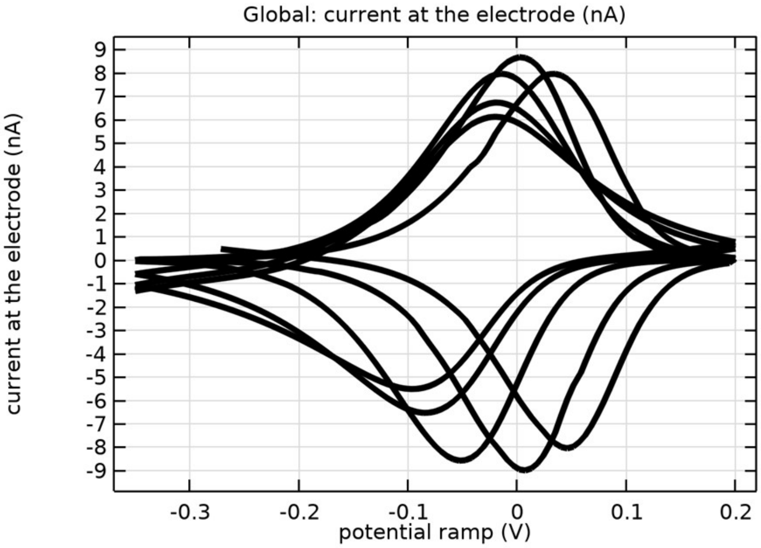


*Global: current at the electrode (nA)*
