# Supplementary material for: Understanding dynamic voltammetry in a dissolving microdroplet
Source: Analyst. 2024 Apr 30;149(15):3939–50. doi: 10.1039/d4an00299g (PMC11262062; doi:10.1039/d4an00299g)
Supplement: AN-149-D4AN00299G-s005 [file AN-149-D4AN00299G-s005.pdf]

Electronic Supplementary Material (ESI) for Analyst.
This journal is © The Royal Society of Chemistry 2024

Supplementary Information

Understanding Dynamic Voltammetry in a Dissolving Microdroplet

Ashutosh Rana[a], Christophe Renault[a*] and Jeffrey E. Dick[a,b*]

[a]Department of Chemistry, Purdue University, West Lafayette, IN, 47907, USA
[b]Elmore Family School of Electrical and Computer Engineering, Purdue University, West Lafayette, IN, 47907, USA
*Corresponding Author(s)
Jeffrey E. Dick (jdick@purdue.edu)
Christophe Renault (crenaulat@purdue.edu)

Sl. no	Figure/ Methods	Pg. no
1	Experimental Section	S2
2	**Figure S1:** Optical micrograph showing the size of the Au disk UME	S3
3	**Figure S2:** Experimental and simulated voltammogram of 155 μM $(Cp^*)_2Fe^{(II/III)}$ in DCE.	S4
4	**Figure S3:** Cyclic voltammetry of 1 mM $(Cp^*)_2Fe^{(II/III)}$ in bulk DCE phase with 10 mM tertbutyl ammonium perchlorate.	S5
5	Bond Number Calculation	S6
6	**Figure S4:** Experimental data and noise filtered data using Savitzky-Golay filter for the purple voltammogram shown in Fig. 3 (h)	S7
7	**Method:** Contact Angle Measurement (**Figure S5**)	S8
8	**Method:** Geometrical Description of Droplet (**Figure S6**)	S9
9	Rate of dissolution of a sessile droplet under diffusion limited condition	S10
10	Calculating diffusion coefficient of DCE in water for CCA and CCR modes, rate of decrease of contact angle vs. $K(\theta)$ (red points) (**Figure S7**)	S11-S12
11	Derivation of the Mesh Velocity	S13-S14
12	Calculation of Junction Potential ($\Delta\phi^{w/o}$), Partitioning of perchlorate ions across the oil-water interface during the oxidation and the reduction (**Figure S8**)	S15
13	Details of the Numerical Simulation	S16-S19
14	Method: Theoretical Lifetime of Droplet, **Figure S9:** Meshed geometry for the simulation, **Figure S10:** Simulated voltammograms for "bulk-like" condition and thin-layer-like" condition, **Figure S11:** Total number of moles of $(Cp^*)_2Fe^{(II)}$ and $(Cp^*)_2Fe^{(III)}$ as a function of time during the entire simulation.	S20-S22
15	**Figure S12.** Cyclic voltammetry measurements for 1 mM of $(Cp^*)_2Fe^{(II)}$) droplets within a bulk aqueous phase devoid of salts.	S23

Experimental Section

All aqueous solutions were prepared in ultra-pure water (18.20 MΩ.cm) from a GenPure water purification system (Millipore). 1,2-dichloroethane (DCE) was obtained from Sigma Aldrich (Ward-Hill, MA). Decamethyl Ferrocene $(Cp^*)_2Fe^{(II)}$) was obtained from Aldrich (St. Louis, MO). To prepare the aqueous continuous phase, we used 10 mM of sodium perchlorate (Sigma Aldrich) in ultra-pure water, and the droplet contained varying concentrations of $(Cp^*)_2Fe^{(II)}$ in DCE. All reagents were of analytical grade and were used without purification. Glassware was thoroughly cleaned before experimentation with mQ water, acetone (99.9%, Sigma-Aldrich, Ward-Hill, MA), and finally with the respective solvent of interest for that solution. Gold (12.5 µm diameter) working electrodes were obtained from CH Instruments (Austin, TX). The Ag/AgCl, 1 M KCl aqueous reference electrode was purchased from CH Instruments (Austin, TX) and was used as the counter/reference electrode. The working electrodes were polished before use with 0.3 µm alumina powder suspension (Electron Microscopy Sciences, Hatfield, PA) on micro-cloth polishing pads (Buehler, Lake Bluff, IL) in water, followed by dipping them in piranha solution (a mixture of concentrated sulfuric acid with hydrogen peroxide in a ratio of 3:1). The lab-made electrochemical cell was built out of Teflon and cleaned in Piranha solution to remove any trace impurities. The microinjection experiments were performed using a micro-injector (FemtoJet 4i Eppendorf) and microinjection capillart tips of an orifice diameter of 10 um (Eppendorf Femtotips). The position of the microinjector was controlled using an XYZ micro-positioning system (InjectMan 4) and monitored using an optical microscope (mini-2.5X\ 500 FL-Optem Fusion lens system). The optical microscope was equipped with a high-resolution sCMOS camera (C15440 Orca Fusion BT, from Hamamatsu, Japan). All electrochemical experiments were performed using on a CHI 6284E potentiostat (CH Instruments, Austin, TX). The reference electrode was placed in a compartment containing 1 M KCl (Fisher Bioreagents, Fair Lawn, NJ) and connected to the cell containing the continuous phase by a salt bridge. The salt bridge was made by filling a glass tube with 3% agarose (99.9%, Sigma-Aldrich, Ward-Hill, MA) containing 1 M potassium chloride (Fischer Bioreagents, Fair Lawn, NJ). The reference electrode served as both the reference and counter electrode.

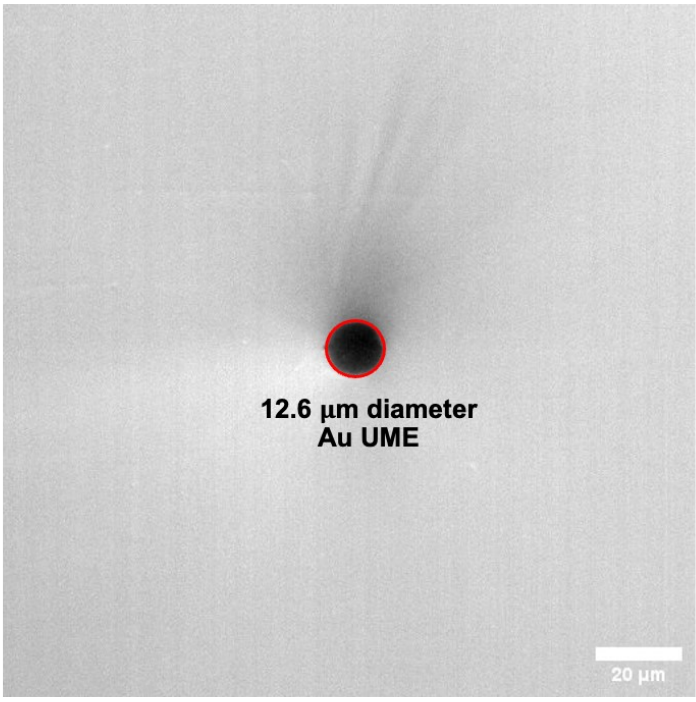

Figure S1. Optical micrograph showing the size of the Au disk UME used. The diameter of the exposed disc was found to be 12.6 µm which corresponds to a radius of 6.3 µm.

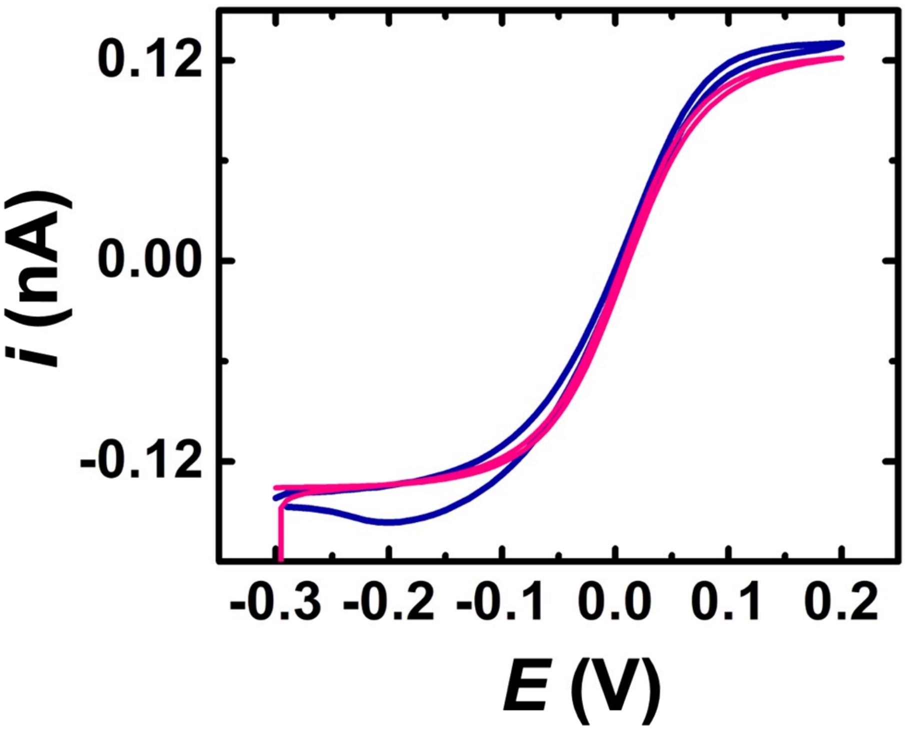

Figure S*2*. Cyclic voltammetry of 155 μM $(Cp^*)_2Fe^{(II/III)}$ in DCE. The cyclic voltammograms were obtained at 0.05 V/s with a 12.3 μm diameter Au disk UME as the working electrode, and Ag/AgCl (1 M KCl) reference served as the reference as well as the counter. The blue curve shows the experimental data and the red curve shows the simulated voltammogram using COMSOL Multiphysics. The simulation incorporated finite redox kinetics ($k_0 = 0.01\ m/s$), a transfer coefficient of 0.5, and an electrode radius of 6.35 μm at room temperature (298 K). The diffusion coefficients for oxidized and reduced forms were 5.3 x 10^{-6} m^2/s and 8.8 x 10^{-6} m^2/s, respectively.

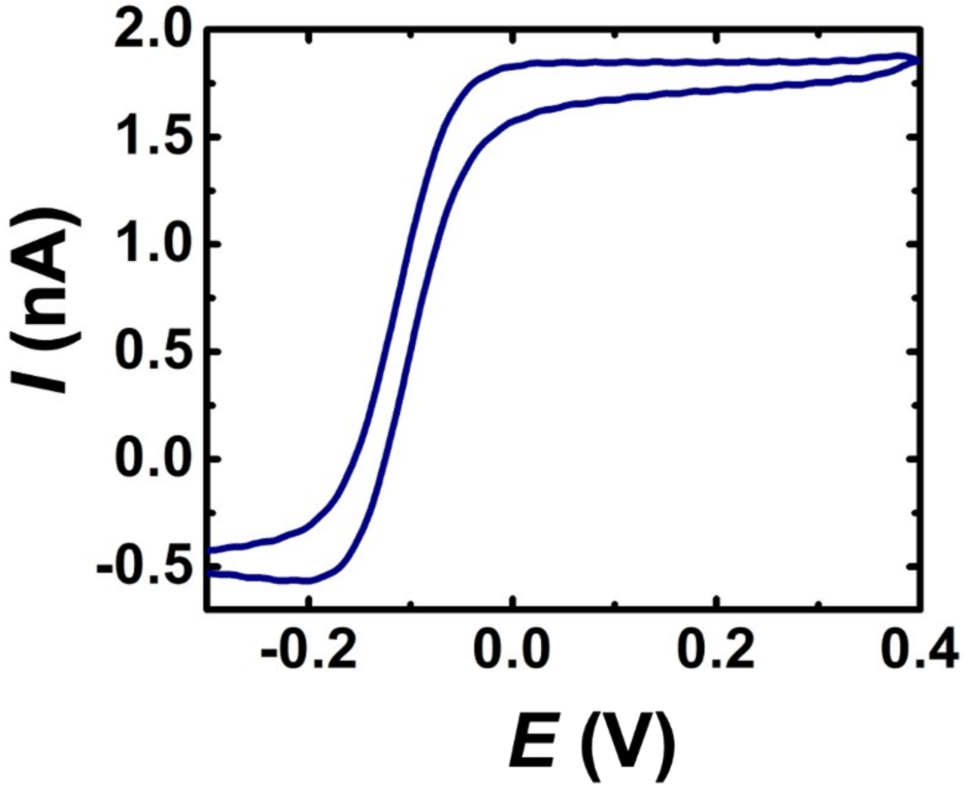

Figure S3: Cyclic voltammetry of 1 mM $(Cp^*)_2Fe^{(II/III)}$ in bulk DCE phase with 10 mM tertbutyl ammonium perchlorate. The cyclic voltammograms were obtained at 0.2 V/s with a 12.3 μm diameter Au disk UME as the working electrode, and a Ag/AgCl (1 M KCl) reference served as the reference as well as the counter. The Ag/AgCl 1 M aqueous reference electrode used in the experiments were separated from the cell by a salt bridge. The $E_{1/2}$ value was found to be -0.1 V vs. Ag/AgCl (1 M KCl) reference.

◊ Bond Number Calculation (B_o)

In fluid mechanics, B_o is a dimensionless number measuring the importance of gravitational forces compared to the surface tension:[1]

$$B_o = \frac{\Delta\rho.g.L^2}{\gamma} \qquad [S1]$$

Where, $\Delta\rho$ is the difference in the density of the two phases (kg/m^3), g is the gravitational acceleration (9.8 m/s^2), L is the radius of the curvature of the droplet (m), γ is the surface tension (N/m).

For a DCE droplet of $L = 51\ \mu m$, the difference in density $(\Delta\rho) = 0.33\ g/mL$ ($\rho_w = 1\ g/mL$ and $\rho_{DCE} = 1.33\ g/mL$ and the surface tension of DCE|water interface is found to $28.2\ mN/m$.[2] Using these values and **Eq. S1**, we obtain, $B_o = 2.98$ x 10^{-4}. The value of $B_o \ll 1$, which implies the affect to gravitational force is negligible in our system.

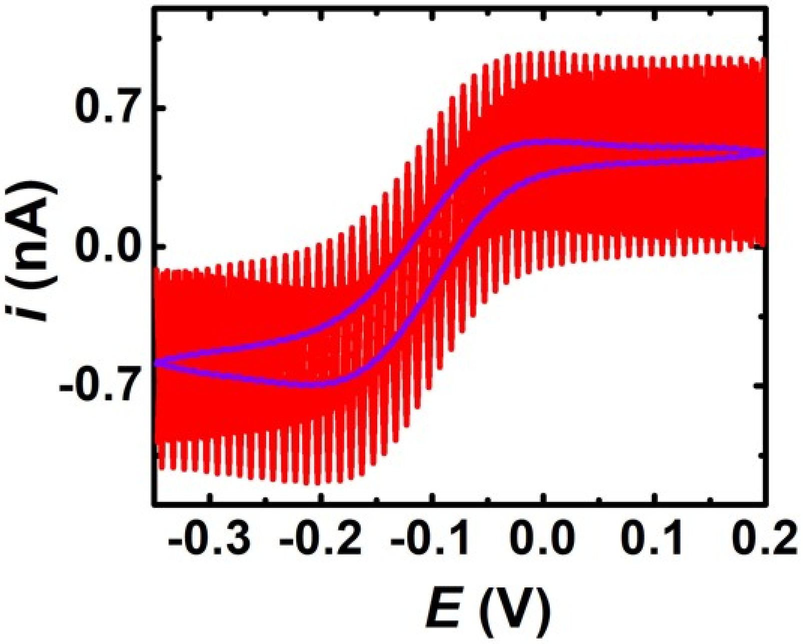

Figure. S4 Experimental data (red) and noise filtered data (purple) using Savitzky-Golay filter for the purple voltammogram shown in Fig. 3 (h). The experiments were conducted without the presence of a Faraday cage, which likely contributed to the observed noise in the system.

◊ Measurement of Contact Angle

An emulsion comprising 1,2 dichloroethane droplets dispersed within a continuous water phase was prepared using a horn sonicator and subsequently transferred into a glass cuvette. The emulsion was allowed to settle, enabling the oil droplets to descend freely onto the underlying glass substrate. Subsequently, side-view optical micrographs at the bottom of the cuvette were captured, as shown in **Fig. S5**.

(a)

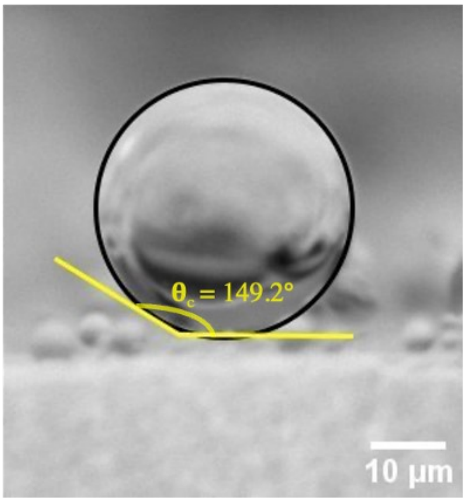

(b)

Sl no.	θ_c
1	149°
2	151°
3	145°
4	149°
5	156°

Std. deviation (σ) = 4°

Figure S5. (a) Contact angle measurement for 1,2 dichloroethane droplets on a glass substrate surrounded by a bulk water phase. The contact angle was measured to be 149 ± 4° (b) Table of measured contact angles for five different droplets, the standard deviation was calculated to be 4°.

◊ Geometrical description of a droplet on a substrate.

A droplet sitting on top of a flat substrate is depicted in **Fig. S6**. Under our experimental conditions gravity is much weaker than surface tension and thus the droplet adopts a spherical curvature. In 2-D the surface of the droplet can be described by the equation of a circle (considering y-axis as the rotational axis of symmetry), which in reality is a sphere in 3-D.

$$(x - x_0)^2 + (y - y_0)^2 = R^2 \qquad [S2]$$

where, R, x_0 and y_0 are the radius of the sphere and the coordinates of the center of the sphere, respectively. Since the droplet does not translate along the x axis, a value of $x_0 = 0$ is used at all time. The intersection between the sphere and the plane is a disk having a radius R_w. This quantity will be referred as the contact radius of the droplet. Therefore, in 3-D the sessile droplet resembles a spherical cap.

The angle θ corresponds to the angle between the tangent of the sphere at $y = 0$ and the substrate. This angle is referred as the contact angle between the droplet and the substrate. These two quantities can be used to derive all the other geometric parameters of this system. We provide below relations between these two quantities and other parameters used in the simulation.

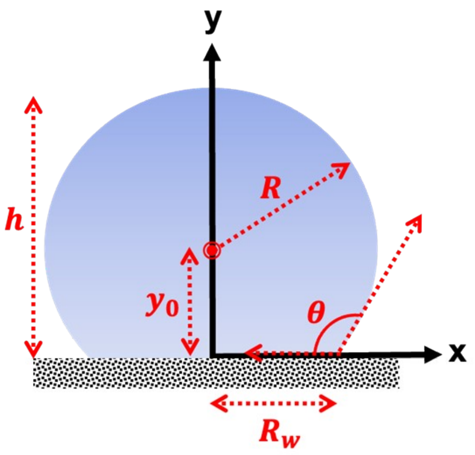

Figure S6. Geometry of a droplet on a substrate. The y axis is a rotational axis of symmetry for the droplet.

Volume of the droplet (V):

$$V = \frac{\pi R_w^3}{3}\left(\frac{sin\theta(2 + cos\theta)}{(1 + cos\theta)^2}\right) \qquad [S3]$$

Height of the droplet (h):

$$h = R_w\left(\frac{1 - \cos\theta}{sin\theta}\right) \qquad [S4]$$

Position of the center of the circle (x_0, y_0)

$$x_0 = 0 \qquad y_0 = \frac{R_w}{\tan\theta} \qquad [S5]$$

Radius of the circle (R):

$$R = \frac{R_w}{\sin\theta} \qquad [S6]$$

◊ Rate of dissolution of a sessile droplet under diffusion limited condition

The rate-limiting process varies depending on the specific conditions, encompassing factors such as vapor transport, phase transition at the droplet's free surface, heat transfer within the droplet, heat conduction through the substrate, or their combination. Among these scenarios, the diffusion-limited model is extensively studied for situations where the diffusive transport of vapor from the droplet into the atmosphere governs the evaporation process.[3] Extensive theoretical, numerical, and experimental efforts have been dedicated to exploring this research domain.[4–6] Evaporation of a droplet occurs due to a negative concentration gradient of its constituents, which diffuses from the droplet interface towards the surrounding medium. Initially, Epstein and Plesset described this for free spherical air bubble in water.[7] This solution was adapted for sessile droplets, considering the modified geometry and boundary conditions, particularly the absence of flux through the substrate, giving the solution for evaporation of sessile droplets. In 2005, Popov et al. extended this formulation to evaporating sessile droplets in air. The rate of volume change for such droplets is given by:[8]

$$\frac{dV}{dt} = -\frac{\pi R_w D M (c_{sat} - c_\infty)}{\rho} \frac{g(\theta)}{(1 + cos\theta)^2} \qquad [S7]$$

D is the diffusion coefficient of the molecules constituting the droplet phase (DCE in our case) in the bulk phase (water in our case). ρ is the density of the dissolving phase (the DCE droplet) defined here in units of volume per unit of mass. M is the molecular weight of the molecules constituting the droplet phase. c_{sat} is the concentration at the interface between the droplet and the bulk phase. It is taken as the maximum concentration of DCE that can be dissolved in water (i.e., the concentration at saturation). C_∞ is the concentration far from the interface. Since the cell is opened and DCE in water can evaporate this value is kept at zero at all time. Finally, the function $g(\theta)$ in **Eq. 7** is given by

$$g(\theta) = (1 + cos\theta)^2 \left\{ tan\left(\frac{\theta}{2}\right) + 8\int_0^\infty \frac{cosh^2(\theta\varepsilon)}{sinh(\theta\varepsilon)} tanh(\varepsilon(\pi - \theta)) d\varepsilon \right\} \qquad [S8]$$

This function accounts for the substrate hindering the diffusion of DCE into water. When a spherical droplet is considered (i.e., in absence of a substrate) the term $\frac{g(\theta)}{(1 + cos\theta)^2}$ in **Eq. [7]** is equal to 1.

◊ Calculating diffusion coefficient of DCE in water for CCA and CCR modes

Part A: **CCA Mode of Dissolution**

As discussed previously, the contact angle (θ_c) remains constant during CCA mode. Differentiating **Eq. S3** with the constraint of a constant θ_c, and plugging in the value of $\frac{dV}{dt}$, into **Eq. S7** yields:

$$\frac{dR}{dt} = -\left\{\frac{D(c_{sat} - c_\infty)M}{\rho} . \frac{1}{\sin^3\theta(2 + \cos\theta)} . g(\theta)\right\} . \frac{1}{R} \qquad [S9]$$

Therefore, a plot of $\frac{dR}{dt}$ vs. $\frac{1}{R}$ has a constant slope:

$$m_{CCA} = -\left\{\frac{(c_{sat} - c_\infty)M}{\rho} . \frac{1}{\sin^3\theta(2 + \cos\theta)} . g(\theta)\right\} D \qquad [S10]$$

Using the value of m from the fit, the value of D for DCE in water can be calculated. From the fit shown in **Fig. 3 (e)** in the main text, the value of m_{CCA} was found to be -2.4 cm^2/s, which gives a value of D for DCE in water as 4.5 x 10^{-6} cm^2/s.

Part A: **CCR Mode of Dissolution**

For the CCR mode of dissolution, R_w remains constant and θ_c decreases. Similar to the last case, differentiating **Eq. S3** with the constraint of a constant R_w, and plugging in the value of $\frac{dV}{dt}$, into **Eq. S7** yields:

$$\frac{d\theta}{dt} = -\left\{\frac{D(c_{sat} - c_\infty)M}{\rho . R_w^2}\right\} . g(\theta) . (1 + cos\theta)^2 \qquad [S11]$$

We define $g(\theta).(1 + cos\theta)^2$ as $Q(\theta)$ to obtain the more compact formula:

$$\frac{d\theta}{dt} = -\left\{\frac{D(c_{sat} - c_\infty)M}{\rho . R_w^2}\right\} . K(\theta) \qquad [S12]$$

A plot of $\frac{d\theta}{dt}$ vs. $Q(\theta)$ is has a constant slope:

$$m_{CCR} = -\left\{\frac{(c_{sat} - c_\infty)M}{\rho R_w^2}\right\} D \qquad [S13]$$

Using the value of m_{CCR} from the fit, the value of D for DCE in water can be calculated. From the fit shown below, **Fig. S7**, the value of m_{CCR} was found to be -3.8 cm^2/s, which gives a value of D for DCE in water as 3 x 10^{-6} cm^2/s.

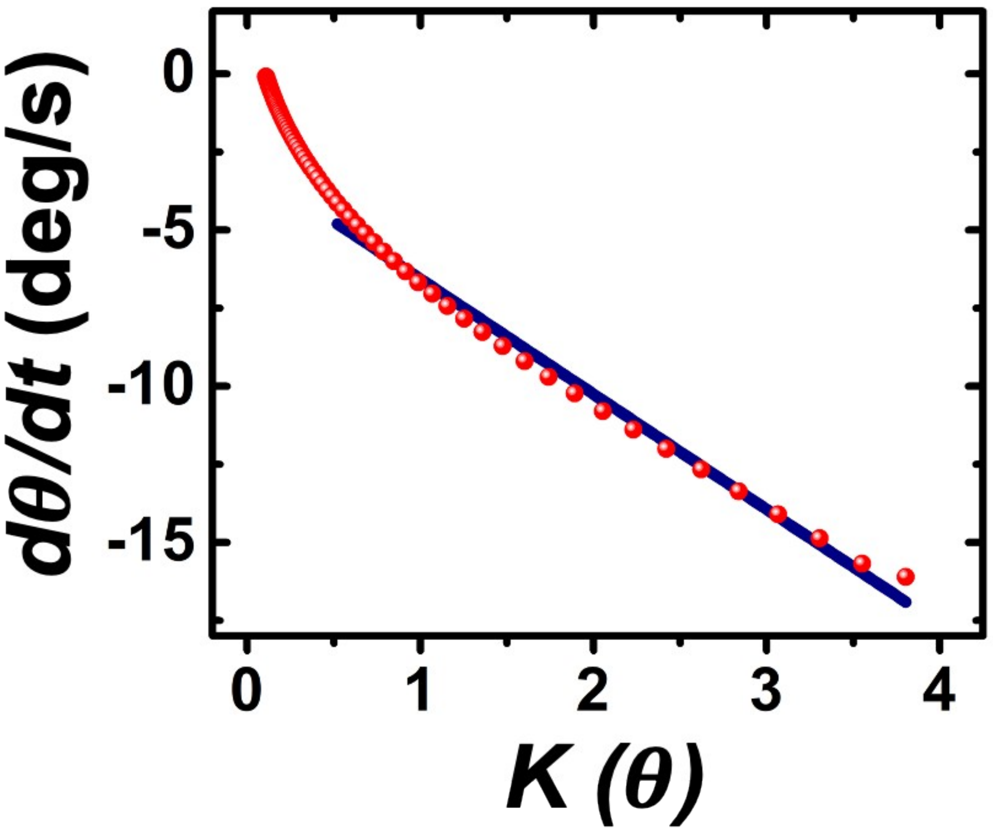

Figure S7. Rate of contact angle decrease vs. $K(\theta)$ (red points). The solid navy curve is a linear fit on the data, slope = -3.8 ± 0.2 cm^2 /s, R^2 = 0.986.

◊ Derivation of the Mesh Velocity

Part A: CCR Mode of Dissolution

The circle defining the boundary between the droplet and the bulk phase can be expressed as a function of the contact angle and the contact radius by combining **Eqs. S4** and S5 to obtain:

$$x^2 + \left(y + \frac{R_w}{tan\theta}\right)^2 = \left(\frac{R_w}{sin\theta}\right)^2 \qquad [S14]$$

The derivative of the **Eq. S11** with respect to time is

$$2x\left(\frac{dx}{dt}\right) + 2\left(y + \frac{R_w}{tan\theta}\right)\left(\frac{dy}{dt} - \frac{R_w}{sin^2\theta}.\frac{d\theta}{dt}\right) =- \frac{2R_w^2}{tan\theta.sin^2\theta}\left(\frac{d\theta}{dt}\right) \qquad [S15]$$

Note that there are no time derivatives for R_w as it is a constant during CCR mode of dissolution. For the point $(0,y = h)$, **Eq. S12** can be written as:

$$2\left(h + \frac{R_w}{tan\theta}\right)\left(\frac{dy}{dt} - \frac{R_w}{tan\theta}\right) = -\frac{2R_w^2}{tan\theta.sin^2\theta}\left(\frac{d\theta}{dt}\right)$$

$$\left.\frac{dy}{dt}\right|_{x=0} = \frac{h}{\sin\theta}\left(\frac{d\theta}{dt}\right) \qquad [S16]$$

The problem of deriving the rest of points is the parameterizations of the circumference of the circle.[9] We can define a scaling variable $\breve{y}$, such that it goes from 0 to 1 and is independent of θ. The expression for $\breve{y}$ is given as:

$$y = \breve{y}.y(x = 0) = \breve{y}.h \qquad [S17]$$

Using **Eq. S13** and S**14**, we can write an expression for (dy/dt) for all other points on the circumference of the circle. Differentiating **Eq. S13** and substituting in **Eq. S14** we get:

$$\left(\frac{dy}{dt}\right) = \frac{y}{sin\theta}\left(\frac{d\theta}{dt}\right) \qquad [S18]$$

Now that we have the expression for all the velocity along the *y* axis, we can substitute **Eq. [15]** back into **Eq. S12** to calculate the velocity along the *x* axis, which yields the following:

$$\left(\frac{dx}{dt}\right) = \left(\frac{h - y}{x}\right)\left(\frac{dy}{dx}\right) + \frac{y}{sin\theta}\left(\frac{d\theta}{dt}\right) \qquad [S19]$$

Eq. S15 and **S16** describe the *x* and *y* components of velocity of the droplet boundary during the CCR mode of dissolution of the droplet.

Part B: CCA Mode of Dissolution

To derive equation for the velocity of the droplet boundary along the *x* and *y* axis, a similar approach as part A can be taken. **Eq. S11** can be written in terms of the radius of the circle (R).

$$x^2 + (y + R\cos\theta)^2 = R^2 \qquad [S20]$$

This equation of circle described above can be differentiated w.r.t time to give the following expression:

$$2x\left(\frac{dx}{dt}\right) + 2(y + Rcos\theta)\left(\frac{dy}{dt} + cos\theta\frac{dR}{dt}\right) = 2R\left(\frac{dR}{dt}\right) \qquad [S21]$$

Note that θ is a constant during CCR mode of dissolution. For the point $(0,y = h)$, **Eq. S18** can be written as:

$$2(h + Rcos\theta)\left(\frac{dy}{dt}\bigg|_{x=0} + cos\theta\frac{dR}{dt}\right) = 2R\frac{dR}{dt} \qquad [S22]$$

$$\frac{dy}{dt}\bigg|_{x=0} = (1 - cos\theta)\frac{dR}{dt} \qquad [S23]$$

Defining a scaling variable $\breve{y}$ like the previous case, we can represent the velocity of all of the points along the y axis as the following:

$$y = \breve{y}.y(x = 0) = \breve{y}.h \qquad [S24]$$

Using **Eq. S20** and **S21**, we can write an expression for (dy/dt) for all other on the circumference of the circle. Differentiating **Eq. S21** and substituting **Eq. S20** we obtain:

$$\frac{dy}{dt} = \frac{y}{R}\frac{dR}{dt} \qquad [S25]$$

Now that we have the expression for all the velocity along the *y* axis (**Eq. S22**), we can substitute **Eq. S22** into **Eq. S18** to calculate the velocity along the *x* axis, which yields the following:

$$\frac{dx}{dt} = \frac{\left[R^2 - (y + Rcos\theta)^2\right]dR}{Rx \qquad dt} \qquad [S26]$$

To further simplify **Eq. S23,** we can substitute results from **Eq. S17** to derive the following expression:

$$\frac{dx}{dt} = \frac{x}{R}\frac{dR}{dt} \qquad [S27]$$

Eq. S21 and **S24**, can accurately describe the velocity of the droplet boundary during the CCA mode of dissolution of the droplet.

◊ Calculation of Junction Potential ($\Delta\phi^{w/o}$)

Junction potential at the oil|water interface occurs as a result of the difference of solubility of the ion between the two immiscible liquids. The electrical potential difference across the interface, $\Delta\phi^{w/o}$, is calculated using:[10]

$$\Delta\phi^{w/o} = \frac{\Delta G^{o,\ w\to o}_{ClO_4}}{F} + \frac{RT}{F}\ln\left(\frac{a^{DCE}_{ClO_4}}{a^{water}_{ClO_4}}\right) \qquad [S28]$$

The first term on the right side is the standard potential difference of ion transfer for ClO_4^- from water to DCE. The second term on the right depends on the ratio of ClO_4^- in both phases. This equation assumes that only ClO_4^- will partition between DCE and water (i.e. Na^+ partitioning is neglected). This is a good approximation considering that $\left|\Delta G^{o,\ w\to o}_{ClO_4}\right|(-170\ mV) < \left|\Delta G^{o,\ w\to o}_{Na}\right|(591\ mV)$.[11,12] Maintaining electro-neutrality inside the DCE droplet imposes that $[anion]_{DCE} = [cation]_{DCE}$. Since we assumed that ClO_4^- is the only anion that can be present in DCE then, for each $(Cp^*)_2Fe^{(II)}$ being oxidized to $(Cp^*)_2Fe^{(III)}$ there has to be one ClO_4^- entering the droplet and

$$[ClO_4^-]_{DCE} = [(Cp^*)_2Fe^{(III)}]_{DCE} \qquad [S29]$$

This process is depicted in **Fig. S8**.

Figure S8. Partitioning of perchlorate ions across the oil-water interface during the oxidation and the reduction of $(Cp^*)_2Fe^{(II)}$ and $(Cp^*)_2Fe^{(III)}$, respectively. For the sake of clarity $(Cp^*)_2Fe^{(III)}$ is denoted as Cp^{*+} and $(Cp^*)_2Fe^{(II)}$ is denoted as Cp*.

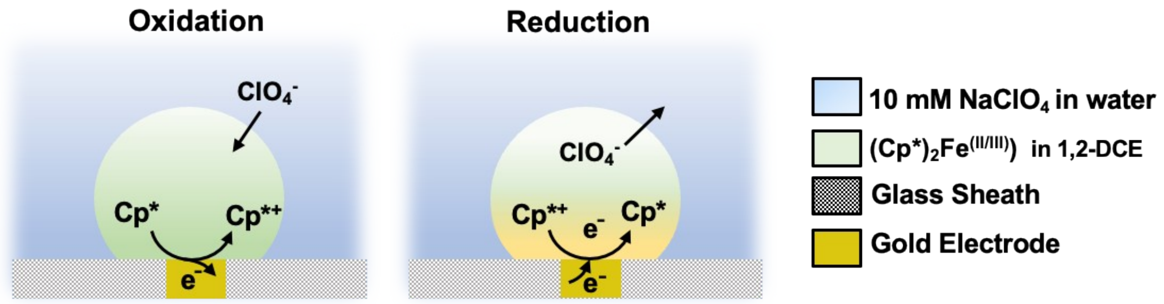

The total potential loss between the working electrode and the reference electrode is the summation of all the potential loss along the electrical path. In addition to the potential loss at the DCE|water interface we should consider potential loss at the salt bridge and the frit of the reference, for example. These last two sources of potential loss are assumed to be constant as the composition of the solution at each of these interfaces is not changing. Hence, the total potential loss becomes:

$$\Delta\phi = \frac{\Delta G^{o,\ w\to o}_{ClO_4}}{F} + \left(\frac{RT}{F}\right)\ln\left(\frac{[ClO_4^-]^{DCE}}{[ClO_4^-]^{water}}\right) + cste \qquad [S30]$$

where *cste* corresponds to constant sources of potential loss (salt bridge and frite of the reference electrode). The value of $[ClO_4^-]^{water}$ is 10 mM and constant with time as the aqueous phase acts as an infinite reservoir of perchlorate ions. $[ClO_4^-]^{DCE}$ is assumed to be equal to $[(Cp^*)_2Fe^{(III)}]$. The $\Delta\phi^{w/o}$ vs. time curve in the main file was adjusted for a potential of 19 mV, which is attributed to the junction potential loss at the salt bridge-water interface.

◊ Numerical Simulation

This section describes the overarching design of the simulation and provides explanation about the choice of the boundaries, important parameters to consider and how was tested the simulation. A full COMSOL report is provided in a separate file in SI.

Simulations were performed using a Finite Element Method with a commercial software, COMSOL Multiphysics 6.0. A 2D axial symmetry was used with a time-dependent solver. Simulations were run on a PC equipped with an intel Xeon processor, 64-bit operating system, x64-based processor and 32 GBs of RAM. A typical simulation takes about 45 min which lead to approximately 11 seconds of simulation per second of experiment. A sequence of 44 voltammograms represents 242 s under our experimental conditions.

The goal of the simulations is to predict the electrochemical response of the $(Cp^*)_2Fe^{(III)}$, $/(Cp^*)_2Fe^{(II)}$, contained in a droplet during the dissolution of this latter. More precisely, the simulation solves for the diffusion of the redox molecules within the droplet in presence of a UME biased with a ramp of potential. The dissolution of the droplet is accounted in the simulation by deforming the mesh with an Arbitrary Lagrangian-Eulerian (ALE) method. The rate of deformation of the mesh is an adjustable parameter of the simulation. To ease the adjustment of the simulation on the experiment, the simulation was cut into 5 separate time segments (called "Study" in COMSOL) with the "n+1" study starting from the final solution of the "n" study.

Geometry & Mesh Deformation

The meshed geometry used for the simulation is shown in **Fig. S9**. Only the volume inside the droplet is simulated. The electrode sits at the bottom of the droplet. A small volume surrounding the electrode is finely mesh to capture precisely the gradients of concentration. Meshing is critical to avoid significantly distorted elements. The initial mesh is designed to follow the deformation, like a conformal mapping. An automatic remeshing node was used in the solver to prevent very large distortions. The initial mesh was optimized by manually increasing the number of elements in the mesh. A number of 3,500 elements was chosen.

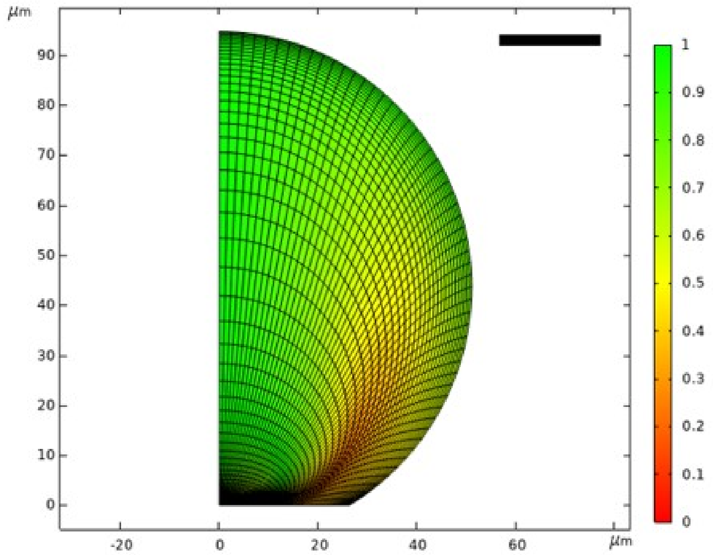

Figure S9. Meshed geometry for the simulation. The scale bar is 20 µm.

Since gravitational forces are weak in comparison to surface tension the droplet is assumed to adopt a spherical curvature at all time. The simulation reflects this shape by using the equation of circle to draw the initial droplet and subsequently using specific rates equations for the mesh deformation that ensure the conservation of the curvature. Two cases were considered, constant contact angle (CCA) and constant contact radius (CCR). The equations for both cases are given in Table S1. A node "Prescribed Mesh Velocity" in the ALE module was used to apply a deformation on the mesh. The derivation of the rate equations is given on **Pg. no S13-14**.

Table S1. Rate equations for the « *r* » and « *z* » velocity components of the mesh at the water|DCE interface.

Velocity	CCA	CCR
V_r	**Eq. S27**	**Eq. S19**
V_z	**Eq. S25**	**Eq. S18**

The dissolution of the droplet can alternate between these two modes in the so-called "stick & slip" mode. The transition between CCA and CCR mode is determined in the simulation by adding a conditional statement in the mesh velocity node. If the contact radius is larger than 1.25 times the radius of the electrode (i.e., 8 µm) then, the dissolution follows a CCA mode. Otherwise, mesh displacement obeys a CCR mode. This reflects our experimental observations. The contact line between the droplet and the glass is pinned when reaching the gold/glass boundary. A value of 1.25 (and not 1) is used to avoid the disappearance of a boundary (the glass sheath) during the simulation.

Physics

The diffusion of the species $(Cp^*)_2Fe^{(III)}$ and $(Cp^*)_2Fe^{(II)}$ inside the droplet during the course of the voltammetric experiment is simulated by solving the second Fick's law:

$$\frac{\partial C_i}{\partial t} + D_i \nabla C_i = 0$$

where D_i, and C_i are the diffusion coefficients and concentrations of the species "i", respectively. Briefly, a flux boundary is used at the surface of the electrode to reflect the consumption/production of the species upon exchange of an electron. The Butler-Volmer law for current-potential is used to set the flux. The flux is controlled by the difference of potential between a reference (taken here as the potential of the solution far away from the electrode) and the surface of the electrode. This potential difference is expressed as the sum of three terms:

$$\Delta E = E_{WE} + Ri + \Delta\phi$$

Where, the potential ramp applied at the electrode, E_{WE}, the potential loss in solution, Ri, and the junction potential at the oil/water interface. The first term is the applied potential ramp shown in **Fig. 3 (f)** in the main text. The potential loss in solution (aka ohmic drop) is defined as the product of the current passing through the electrode, i, and the resistance, R, of the solution:

$$R = R_O \frac{C_{ox,\ ini}}{C_{Ox}}$$

Where, R_0, $C_{ox,ini}$ and C_{ox} are the initial resistance of the solution, the initial concentration of $(Cp^*)_2Fe^{(III)}$ and the concentration of $(Cp^*)_2Fe^{(III)}$, respectively. Since water contains 10 mM of supporting electrolyte but the DCE droplet does not have any added salt we assume that most of the resistance originates from the droplet. The resistance is proportional (in first approximation) to the concentration of ions and thus the resistance of the droplet will vary as a function of concentration of $(Cp^*)_2Fe^{(III)}$. The initial resistance was found by adjusting the first CVs in **Fig. 3 (g)**. A value of 100 MΩ was determined. The value of $C_{ox,ini}$ is set as 20% of the initial concentration of ferrocenyl species.

Validation of the model

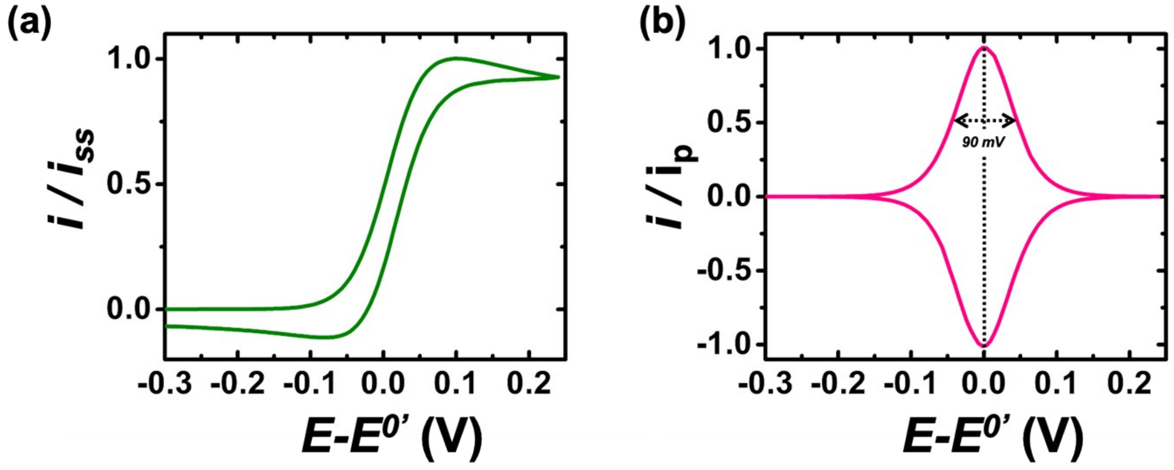

Figure S10. (a) Simulated voltammograms for "bulk-like" condition using a droplet of $51\ \mu m$ radius and a contact angle of 149° **(b)** Simulated voltammograms for "thin-layer-like" condition using a droplet of $8\ \mu m$ radius and a contact angle of 60°.

First, we simulated cyclic voltammograms in two limiting cases, "bulk-like" condition and "thin layer-like" condition. These two simulations are given in **Fig. S10 (a) and (b)**, respectively. For both, **Fig. S9 (a) and (b)**, a scan rate of $20\ mV/s$ a formal potential of -$40\ mV$ was used. In **Fig. S9 (a)**, the current on the y axis was normalized with respect to the steady state current given by the following equation:[10]

$$i_{ss} = 4nFCDr \qquad [S31]$$

In **Fig. S9 (b)**, the current on the y axis was normalized with respect to the value of the peak current (i_p) for thin-layer voltammetry given by the following equation:[10]

$$i_p = \frac{n^2F^2vCV}{4RT} \qquad [S32]$$

In **Eqs. S31** and **S32**, denoted the no of electrons, is the Faraday's constant, C is the concentration of the redox species (155 uM Cred), v is the scan rate, D is the diffusion coefficient, R is the universal gas constant, r is the radius of the electrode (6.3 um), T is the temperature (298 K) and V is the volume of the solution. For, **Fig. S10 (b)**, the droplet volume was calculated using **Eq. S3** with $\theta_c = 60°$ and droplet radius of $8\ \mu m$. The product of CV in **Eq. 32** was found to be 5.19 x 10^{-17} moles. The FWHM is indicated with the dotted line and corresponds to value of $90\ mV$, which is typical of voltammetry in thin-layer electrochemistry. A good agreement is observed. It was observed that in the absence of any ion-transfer across the oil-water interface, the simulation struggled to conserve the mass of ferrocenyl molecules. We attribute this to systematic errors made by the simulation during the droplet dissolution process. To address this, a compensation for mass loss function was introduced in the form of an interpolation function, with values manually determined to ensure the conservation of overall mass within the droplet. This was implemented before introducing any partitioning kinetics across the oil-water interface. Further details on the mass loss function can be found in the COMSOL report. Then, we ensured that mass is being conserved (in absence of our first order kinetic of ion transfer). **Fig. S11** shows the number of moles of species (normalized with respect to the initial amount) during the entire simulation. In order to enforce mass conservation, we used a manually parametrized function that accounts for systematic errors made while adjusting the movement of the water|DCE boundary and the flux of species at that same boundary. These errors are kept below $\pm 2\ \%$ at all times. A Lagrange multiplier approach was also tested to maintain mass conservation. However, due to the prohibitive increase of simulation time (about 10X) we favored manual parametrization that would take about one day to perform and then could be used for all the simulations.

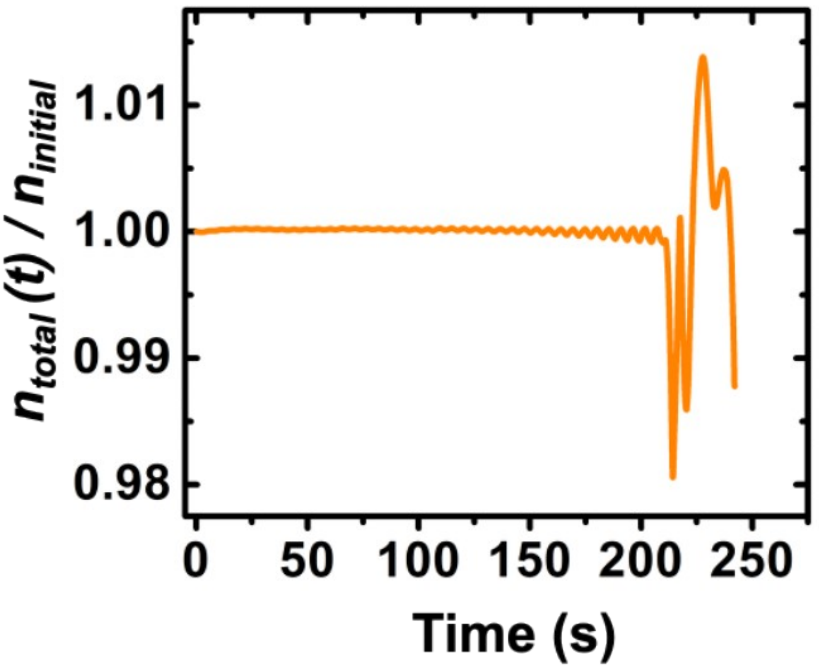

Figure S11. Total number of moles of $(Cp^*)_2Fe^{(II)}$ and $(Cp^*)_2Fe^{(III)}$ normalized with respect to the initial total amount, ~83 fmol as a function of time during the entire simulation.

◊ Theoretical lifetime of a droplet

The lifetime (τ) of a droplet is defined as the time between the initial volume, V_0, of the droplet (defined as the volume at t = 0 s) and the final volume (V_f) = 0. Depending on the mode of dissolution (CCR or CCA) different sets of equation are used to determine τ. Under our experimental conditions the droplet initially dissolves in a CCA mode starting with an initial contact radius $R_w(t=0)$ until reaching at a certain time t_ca radius $R_w(t_c)$. Then, the droplet follows CCR mode of dissolution. The total lifetime of the droplet (τ) is the sum of the times in CCA (τ_{CCA}) and CCR τ_{CCR} modes: $\tau = \tau_{CCA} + \tau_{CCR}$. The following section presents the equations of τ_{CCA} and τ_{CCR}.[4]

Part A: τ_{CCA} CCA Mode of Dissolution

$$\tau_{CCA} = \frac{\rho}{D(c_{sat} - c_\infty)M}.\frac{1}{g(\theta)}.(sin\theta(2 + cos\theta)).\left[R_w(t=0)^2 - R_w(t_c)^2\right] \qquad [S33]$$

Part B: τ_{CCA} CCR Mode of Dissolution

$$\tau_{CCR} = \frac{\rho}{D(c_{sat} - c_\infty)M}.\left(\frac{3V_0}{2\pi}\right)^{\frac{2}{3}}\left(\frac{2(1 + cos\theta_o)^2}{sin\theta_0(2 + cos\theta_o)}\right)^{\frac{2}{3}}\int_0^{\theta_0}\frac{2}{g(\theta)}d\theta \qquad [S34]$$

where θ_0 is the initial contact angle ($149°$), V_0 is the initial droplet volume that can be calculated using **Eq. S3**. The lifetime calculation was done using numerical integration on MATLAB. The code is provided below.

```
close all;
clear;
clc;

%%%%% DEFINE ALL THE TRIGO FUNCTIONS   %%%%%%%%%%%%%%%%%%%%

f = @(x, tetha) ( tanh( x*(pi-tetha) ).* (cosh(tetha*x).^2) ./ sinh(2*pi*x));

%%%% Parameterization of the model %%%%%

R0 = 51E-4;                  % apparent radius of droplet at t = 0 s measured by ImageJ
tetha0= 149*pi/180;          % initial contact angle in "degree", converted in radians
R0_CR = 8E-4;                % initial contact radius in CR mode in "cm"
```

```
%%%% Physical constants   %%%%%%%%

R0_CA = R0*sin(tetha0);     % initial contact radius in CA mode in "cm"
rho = 1.25;                 % density in g/cm3
M = 98.96;                  % molecular weight in g/mol
D = 9.9E-6;                 % diffusion coefficient of DCE in water in "cm2/s"
Csat = 87.5E-6;             % saturation concentration of DCE in water in "mol/cm3"



%%%%%%%%% CR mode %%%%%%%%%%%%%%%%%%%%%%
%%%%%%%%%%%%%%%%%%%%%%%%%%%%%%%%%%%%%%%%%

cste = (rho*(R0_CR^2))/(M*D*Csat);         % constante in "s"

%%%%  Computes the function "g"

n=100;                                % define the number of contact angles
tetha_final =0;                       % minimum value of teha for computation
tetha = linspace(tetha_final,tetha0,n);     % creates an array of equaly spaced tetha values by dividing the initial tetha value into 100 steps
g=zeros(0,n);                         % preallocates an array with hundred values to store the solution of "g"
p=zeros(0,n);

for m=1:n
p(m) = integral( @(x) f(x,tetha(m)),0,12);     % the convergence of this integral reaches 0.001% at 12
g(m) = ( (1+cos(tetha(m)))^2 ) *(tan( tetha(m)/2 )+ 8*p(m));
end


%%%% Computes the lifetime in CA mode for a 100 different initial contact angles

I = zeros(size(g));
t_CR = zeros(size(g));
%Q = zeros(size(g));   %another way to perform the integration with trapezoidal method

for m=1:n
    dx = (tetha0-tetha_final)/n;
    I(m) = (1./g(m))*dx;
    t_CR(m) = cste*sum(I);           % integration with the rectangle method
    %Q(m)= cste*trapz(1./g(1:m))*dx;  %another way to perform the integration with trapezoidal method

end

%%%%%%%%%%%%%%%%%%   CA mode   %%%%%%%%%%%%%%%%%%%%%%%%%%%%%%%
%%%%%%%%%%%%%%%%%%%%%%%%%%%%%%%%%%%%%%%%%%%%%%%%%%%%%%%%%%%%%%%%%


%%%% Internal constants for the model %%%%%

p1 = integral( @(x) f(x,tetha0),0,12);     % the convergence of this integral reaches 0.001% at 12
g1 = ( (1+cos(tetha0))^2 ) *(tan( tetha0/2 )+ 8*p1);
cste1 = sin(tetha0)*(2+cos(tetha0))/(g1);
r = (R0_CR:0.1E-4:R0_CA);                 % array of radii from the initial contact radius to the final before switching to CR mode
t_CA = ((rho)/(2*M*D*Csat))*cste1*r.^2;     % array of lifetime to go from initial R0 to values of R in the array "r"
```

```
%V = -1E4*(1./(r))*(D*Csat*M/rho)*g1/(sin(tetha0)*(2+cos(tetha0))); %normal velocity of the DCE/water interface in CA mode from the %analytical expression
V2 = -1./( diff(t_CA)/0.1 );               % normal velocity of DCE/water interface in CA mode computed from the t_CA computed previously

%%%%% Plotting results   %%%%
%%%%%%%%%%%%%%%%%%%%%%%%%%%%%%%%

subplot(2,2,1);                         % CR mode lifetime as a function of tetha
plot(180*tetha/pi,t_CR);
xlim([min(180*tetha/pi) max(180*tetha/pi)]);
title('Constant Radius Mode');
xlabel('contact angle (degree)');
ylabel('time (s)');

subplot(2,2,2);
plot(1E4*r,t_CA);                       % CA mode lifetime as a function of contact radius
xlim([min(1E4*r) max(1E4*r)]);
title('Constant Angle Mode');
xlabel('contact radius (µm)');
ylabel('time (s)');

subplot(2,2,4);
t_CAf=flip(t_CA);
t_CAf(end)=[];
plot(t_CAf,V2);                         % normal velocity in CA mode as a function of time
xlim([min(t_CA) max(t_CA)]);
title('Constant Angle Mode');
xlabel('time (s)');
ylabel('normal velocity (µm/s)');

%%%%%% Provides values of lifetime in different modes  %%%%%

T_CA = ((rho)/(2*M*D*Csat))*cste1*R0_CA^2   -  ((rho)/(2*M*D*Csat))*cste1*R0_CR^2
T_CR = max(t_CR) - min(t_CR)
T_tot =T_CR+T_CA


%%%%%%%%%%%%%%%%%%%%%%%%%%%%%%%%%%%
```

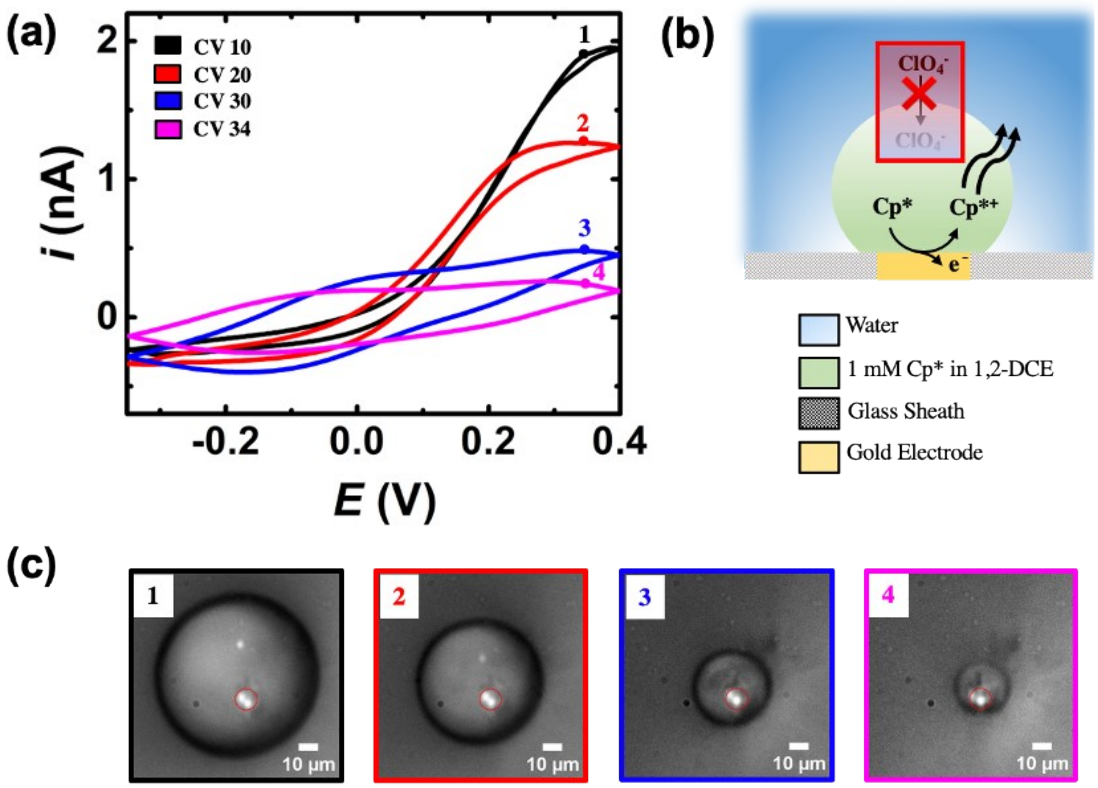

Figure S12. In-situ observation of droplet size dynamics correlated with cyclic voltammetry measurements over time for 1 mM of $(Cp^*)_2Fe^{(II)}$ droplets within a bulk aqueous phase devoid of salts. The cyclic voltammetry measurements were continuously performed as the droplets underwent shrinkage. Panel (a) illustrates voltammograms recorded in different colors, denoted as 1 to 4, corresponding to distinct droplet sizes. Panel (b) showcases color-coded optical micrographs captured at points 1 to 4, corresponding to the voltammograms in panel (a). Panel (c) provides a schematic depiction of the ion partitioning mechanism across the oil-water boundary. The voltammograms presented above were processed using an adjacent weighted averaging technique, employing a window size of 19 points and applying a periodic boundary condition.

References

1. Li, S., Liu, M., Hanaor, D. & Gan, Y. Dynamics of Viscous Entrapped Saturated Zones in Partially Wetted Porous Media. *Transp Porous Media* ***125**, 193–210 (2018)*. DOI: 10.1007/s11242-018-1113-3

2. Hou, B. *et al.* Ion distributions at the water/1,2-dichloroethane interface: Potential of mean force approach to analyzing X-ray reflectivity and interfacial tension measurements. *J. Phys. Chem. B 2013, 117, 17, 5365–5378.* DOI: 10.1021/jp401892y

3. Wilson, S. K.; Ambrosio, H.-M. D. Evaporation of Sessile Droplets. *Annu. Rev. Fluid Mech.* ***2022**, 55, 481–509*. DOI :10.1146/annurev-fluid-031822-013213.

4. Stauber, J. M.; Wilson, S. K.; Duffy, B. R.; Sefiane, K. On the Lifetimes of Evaporating Droplets. *J Fluid Mech* ***2014**, 744*. DOI: 10.1017/jfm.2014.94.

5. Adachi, E. ; Dimitro. A.S. ; Nagayama, K. Stripe Patterns Formed on a Glass Surface during Droplet Evaporation. *Langmuir* 1995, 11, 4, 1057–1060. DOI : 10.1021/la00004a003

6. Gelderblom, H. *et al.* How water droplets evaporate on a superhydrophobic substrate. *Phys Rev E* ***2011**, Stat Nonlin Soft Matter Phys* 83. DOI: 10.1103/PhysRevE.83.026306

7. Epstein, P. S.; Plesset, M. S. On the Stability of Gas Bubbles in Liquid-Gas Solutions. *J Chem Phys 1950, 18 (11), 1505–1509*. DOI: 10.1063/1.1747520.

8. Popov, Y. O. Evaporative Deposition Patterns: Spatial Dimensions of the Deposit. *Phys Rev E Stat Nonlin Soft Matter Phys* ***2005**, 71 (3)*. DOI: 10.1103/PhysRevE.71.036313.

9. Manfredo, R. : & Guilizzoni, G. Comparison of drop evaporation models on hot surface Adrì a González Esteve. Politecnico di Milano (2014).

10. Bard, A. J.; Faulkner, L. R. Electrochemical Methods : Fundamentals and Applications; John Wiley & Sons: New York, 2001

11. Sabela, A., Marecek, V., Samec, Z., Fuocot, R. & Heyrovsky, J. Standard Gibbs Energies Of Transfer Of Univalent Ions From Water To 1,2-Dichloroethane. *Electrochimica. Am* (1992), vol. 37. DOI: 10.1016/0013-4686(92)85008-9

12. Wandlowski, T. *et al. Galvani Potential Scales for Water-Nitrobenzene And Water-1,2-Dichloroethane Interfaces*. *Electrochimica Acta* ***1990***, vol. 35. DOI: 10.1016/0013-4686(90)80035-M
